# The immune‐related biomarker TEK inhibits the development of clear cell renal cell carcinoma (ccRCC) by regulating AKT phosphorylation

**DOI:** 10.1186/s12935-021-01830-1

**Published:** 2021-02-18

**Authors:** Siming Chen, Mengxue Yu, Lingao Ju, Gang Wang, Kaiyu Qian, Yu Xiao, Xinghuan Wang

**Affiliations:** 1 Department of Urology, Zhongnan Hospital of Wuhan University, Wuhan, China; 2 Department of Biological Repositories, Zhongnan Hospital of Wuhan University, Wuhan, China; 3 Human Genetics Resource Preservation Center of Hubei Province, Wuhan, China; 4 Wuhan Research Center for Infectious Diseases and Cancer, Chinese Academy of Medical Sciences, Wuhan, China; 5 Laboratory of Precision Medicine, Zhongnan Hospital of Wuhan University, Wuhan, China; 6 Medical Research Institute, Wuhan University, Wuhan, China

**Keywords:** Clear cell renal cell carcinoma, Tumor microenvironment, Survival prognosis, Tumor‐infiltrating immune cells, TEK

## Abstract

**Background:**

High immunogenicity is an important feature of ccRCC, but its underlying immune-related molecular mechanisms remain unclear. This study aimed to investigate the effect of immune-related gene TEK on ccRCC and its prognostic value.

**Methods:**

The immune-related differentially expressed genes (DEGs) and transcription factors (TFs) in ccRCC were screened based on The Cancer Genome Atlas (TCGA) database, and a regulatory network of TF was constructed. Prognostic-related immune genes were screened by univariate Cox regression analysis and functional annotation was performed. Univariate and multivariate Cox regression analyses were performed to construct the immune gene risk model and identify the hub gene TEK that independently affected the prognosis of ccRCC. The effectiveness of the TEK was verified by external microarray datasets. The relationship between TEK and immune cells in ccRCC was evaluated based on Tumor Immune Estimation Resource (TIMER). The expression of TEK in clinical specimens was verified by qRT-PCR and immunohistochemical (IHC) staining. MTT and cloning formation assay were used to evaluate cell proliferation. Transwell assays were used to assess cell migration. Apoptosis was assessed by flow cytometry, and the expression of related proteins was detected by Western blot and immunofluorescence.

**Results:**

We constructed a prognostic model consisting of 12 hub genes and performed risk scores to determine the relationship between these scores and prognosis. Through Cox regression analysis and survival analysis, TEK, an immune marker highly related to survival prognosis, was obtained and validated. *In vitro* experiments showed that knockdown of *TEK* promoted the proliferation and migration of ccRCC cells, and we found that TEK promoted apoptosis by regulating the phosphorylation of AKT, thereby inhibiting cell proliferation.

**Conclusions:**

TEK plays an important role in risk assessment and survival prediction for ccRCC patients as a new immune gene and maybe an emerging target for immunotherapy for ccRCC patients.

## Introduction

Renal cell carcinoma (RCC) is a relatively common urogenital malignancy with high mortality [1, 2], and the global incidence of RCC is on the rise. ccRCC is the most common pathologic type and the leading cause of death in renal cancer patients [3, 4]. Clinical and basic research on renal cancer is now extensive and in-depth, we can treat the tumor through radical resection, immunotherapy and targeted therapy [5–7]. Moreover, there is growing evidence that checkpoint inhibitor immunotherapy could achieve anti-tumor effects by stimulating an immune response [8]. However, the pathogenesis and mechanism of renal cancer are still not well understood, and we need to find a biomarker to detect the survival prognosis of the immune response in ccRCC patients so as to promote diagnosis and treatment. Tumors are always in a complex tissue microenvironment, and changes in the immune microenvironment might affect the occurrence, development and metastasis of tumors in different ways. Tumor immune escape and immunosuppression are the key factors of tumor development [9]. It is worth noting that many pieces of evidence showed that ccRCC was highly immunogenic [10]. It has high responsiveness and timeliness for immunotherapy [11].

Tumor microenvironment (TME) contains a large number of extracellular matrix, vascular structure, and tumor cells, as well as a large number of infiltrating immune cells, such as tumor-related macrophages, neutrophils, and dendritic cells [12]. TME is not only closely related to tumor growth and migration but also has a profound impact on the therapeutic effect [13]. Now there is increasing evidence that signaling pathways such as MAPK [14], PI3K [15, 16] and Wnt/β-catenin [17] in various types of cancer can impair immune function in the TME and thus resist immunotherapy. Therefore, understanding the composition and function of immune cells and molecules in TME is of great value and significance for tumor diagnosis, prognosis and treatment.

With the rapid development of science and technology, bioinformatics has been widely used in various fields [18–20]. Moreover, it now plays an important role in screening differentially expressed genes [21], discovering and identifying biomarkers, etc. in tumors [22, 23]. In view of this, we can start from the immune direction, find immune-related molecular markers that can predict clinical prognosis and provide clinical treatment directions, and build an effective clinical prognostic model.

In this study, we collected microarray data and relevant information on ccRCC patients from a public database and constructed a 12-gene predictive risk model. We have obtained a hub molecule, TEK, which may play a key role in immunotherapy and survival prediction. In addition, we also selected other data sets to further verify our results. Finally, we confirmed the high expression of TEK in ccRCC and its tumor-promoting mechanism through cellular functional experiments.

## Materials and methods

### Human kidney tissue samples

Postoperative cancer and adjacent tissues of ccRCC patients in Zhongnan Hospital were obtained, and informed consent of all subjects was obtained. The samples were histopathologically confirmed by two pathologists independently. The inclusion criteria are as follows: (1) the histopathological type is confirmed as ccRCC, (2) not received anti-cancer treatment before nephrectomy, (3) underwent radical nephrectomy or nephron sparing partial nephrectomy, (4) no history of other malignant tumors. Exclusion criteria: (1) other pathological types of RCC, (2) metastatic ccRCC or other merge tumors, (3) patients who did not undergo surgery, (4) clinical pathological data is incomplete. The Ethics Committee of Zhongnan Hospital has passed the ethical approval of this study (approval number: 2,020,102).

### Obtained raw biological microarray data and immune‐related genes

First, in this study, data from the public domain was used. We downloaded 611 samples of HTSeq-FPKM transcriptome data from the TCGA database, including 539 ccRCC samples and 72 normal samples that matched [24]. And we through the GDC portal website to obtain the corresponding clinical information of each patient, including histological grade, pathological stage and a large number of follow-up information. We searched for DEGs using RNA-seq data and constructed risk prediction models with clinical information. We collected the chip dataset GSE53757 from the GEO database. The dataset GSE53757 includes 72 ccRCC tissues and 72 corresponding para-cancer tissues, as well as clinical information, to further verify our hub genes. We collected 2498 immune genes from the ImmPort database (https://immport.niaid.nih.gov*)* for the next analysis.

### Screening of immune‐related DEGs

The expression matrix consisted of a raw count of each mRNA of each sample. First of all, we standardized the microarray, eliminated the bias of the data, ensured the homogeneity and integrity of the data, and finally got 20,589 genes. Next, we executed the “limma” R package to screen for DEG in ccRCC tissues and normal kidney tissues [25]. The thresholds for screening DEGs were FDR < 0.05 and |log2 FC| > 1. The obtained DEGs intersected with 2498 immune-related genes to obtain co-existing 682 immune-related DEGs.

### Univariate cox prognostic analysis and construction of multivariate cox risk model

Using the obtained 682 immune-related DEGs for univariate cox prognosis analysis and plotting forest plots (p < 0.001), 35 prognostic-related immune differential genes were obtained. Then a multivariate cox model was constructed for the prognostic-related DEGs screened by Univariate cox, which was used the Coxp function to build and step function to optimize. Finally, the 12 hub genes were determined to construct the model. The risk score calculation formula: risk score =$\sum _{i=1}^{n}(\text{coefi*}\hspace{0.17em}\text{Expri})$, Expri represented the expression of the patient’s gene i, and coef i represented the Cox coefficient of gene i. Then, we performed Cox regression model analysis to screen for the key factors affecting patient survival, including age (ref. low), gender (ref. female), grade (ref. grade1), stage (ref. stage1), pT stage (ref. T1), pM stage (ref. M0), risk score (ref. low) and expression of each hub gene (ref. low).

### Functional annotation of prognostic related immune DEGs

To further determine the biological function of prognostic-related immune DEGs, we used the “clusterProfiler” [26] software package in R for Gene Ontology (GO) function annotation [27] and the Kyoto Encyclopedia of Genes and Genomes (KEGG) pathway enrichment analysis [28]. Thresholds were p < 0.05 and FDR < 0.05.

### Construction of TF regulation network in ccRCC

We collected 318 tumor-related TFs from the Cistrome cancer database and performed differential expression analysis using the “limma” R package. Thresholds were |log2 FC| > 1 and FDR < 0.05. Correlation analysis was then performed using the obtained TFs and the immune-related DEGs in the ccRCC samples, with cor > 0.4 and p < 0.05 as the cutoff criteria. Finally, the molecular interaction network was visualized using Cytoscape [29].

### Risk curve, survival analysis and ROC curve

Based on the risk score, we plotted risk value graphs, survival status charts, and survival heat maps. In order to determine survival differences among groups, we executed an intergroup survival analysis. In addition, we determined the accuracy of the model by drawing the ROC curve and calculated the AUC value.

### Identification and validation of hub genes

We identified genes with p-values less than 0.05 in both univariate and multivariate cox analysis as hub genes that could be used as clinical prognostic markers. The expression of hub gene was determined as a binary variable (high and low), which meant that the median value of each hub gene was used as the threshold value to distinguish. We removed samples with a survival time of fewer than 90 days, and then performed survival analysis and correlation analysis of clinicopathological features. The clinical characteristics included age, gender, grade, stage, pM stage and pT stage. We use an independent data set, GSE53757, for external validation. Based on TIMER database (https://cistrome.shinyapps.io/timer/*)*, we analyzed the connection between the hub genes and immune infiltrating cells.

### Cell culture and transfection

ACHN and Caki-1 cell lines were cultured in MEM and McCoy’s 5A medium containing 10 % FBS. We purchased siTEK from GenePharma. The corresponding sense sequences of TEK are as follows: siTEK-1(si-1): 5’-AGCUUGCUCCUUUCUGGAATT-3’, siTEK-2 (si-2): 5’-GCCGCUACCUACUAAUGAATT-3’, siTEK-3 (si-3): 5’-CCCAGAUCCUACAAUUUAUTT-3’. Lipofectamine 3000 was used for cell transfection.

### RNA isolation and qRT-PCR

According to the kit instructions, we used the RNeasy Mini Kit (Cat. #74,101, Qiagen) to extract total RNA. Then the concentration of RNA was measured and reverse transcribed into cDNA. Finally, qRT-PCR analysis of cDNA was performed by iQ™ SYBR®Green Supermix (Bio-RAD). The primer sequences were as follows: TEK: 5’-TTAGCCAGCTTAGTTCTCTGTGG-3’, 5’-AGCATCAGATACAAGAGGTAGGG-3’; FAS: 5’-TCTGGTTCTTACGTCTGTTGC-3’, 5’-CTGTGCAGTCCCTAGCTTTCC-3’; BAX: 5’-CCCGAGAGGTCTTTTTCCGAG-3’, 5’-CCAGCCCATGATGGTTCTGAT-3’; C-MYC: 5’-CGTCCTCGGATTCTCTGCTC-3’, 5’-GATTTCTTCCTCATCTTCTTGTTC-3’; GAPDH: 5’-GGAGCGAGATCCCTCCAAAAT-3’, 5’-GGCTGTTGTCATACTTCTCATGG-3’.

### MTT assay, transwell assay, and cloning formation assay

For MTT assay, after 36 h of transfection, cells were planted into 96-well plates in different groups, then absorbance measurements were used to assess cell viability at different times. For the cloning formation assay, 1000 transfected cells were grown in a six-well culture dish, fixed and stained 14 days later. For transwell assay, the transfected cells were seeded into the upper chambers (Corning), and 600 µL medium containing 10 % FBS was added into the lower chambers. After incubation for 24 h, the cells were fixed and stained.

### Western blot analysis

The collected cells were lysed in a mixture of RIPA buffer (Sigma-Aldrich, USA), phosphatase inhibitors and protease inhibitors in the ratio of 50:1:1 for 30 minutes on ice. We used a 10 % SDS / PAGE gel to separate proteins and transfer them to a PVDF membrane (Millipore), then sealed them with 5 % skimmed milk and incubated with primary and secondary antibodies. The primary antibodies were as follows: anti-TEK, 1:1000 (Abcam); anti-GAPDH, 1:1000 (Santa Cruz); anti-N-cadherin, 1:500 (CST); anti-E-cadherin, 1:500 (CST); anti-Vimentin, 1:1000 (CST); anti-β-catenin, 1:1000 (CST); anti-AKT(phosphoThr308), 1:1000 (CST); anti-AKT, 1:1000 (CST); anti-Bcl-xL, 1:1000 (CST); anti-Bcl-2, 1:1000 (CST).

### Analysis of apoptosis by flow cytometry

The collected ccRCC cells were centrifuged and washed with cold PBS to prepare the experimental samples. Annexin V FITC Apoptosis Assay Kit I (BD Biosciences, USA) was used for staining in accordance with the instructions and analysis by flow cytometry.

### Immunofluorescence and IHC staining

For immunofluorescence, the cells seeded on the coverslips were washed with PBS and fixed with 4 % PFA, then treated with 0.1 % Triton X-100. After blocking with goat serum, the cells were treated with primary antibody and FITC-labeled or Cy3-labeled secondary antibody, respectively. The nuclei were labeled with DAPI and visualized by confocal microscope. For IHC, put the paraffin sections after deparaffinization in citrate buffer for antigen retrieval, and then blocked with 3 % H_2_O_2_. They were then incubated with primary and secondary antibodies, and finally with the DAB chromogen solution and HRP substrate solution. The primary antibodies were as follows: anti-Ki-67, 1:200 (Novus); anti-TEK, 1:200 (Abcam).

### Statistical analysis

All results were performed more than 3 independent experiments. We used R software and GraphPad Prism 7 (USA) statistical software to analyze the differences between the groups by two-tailed t-test. We reckoned p < 0.05 to be statistically significant.

## Results

### Screening of immune‐related DEGs and TF network construction

**Fig. 1:**
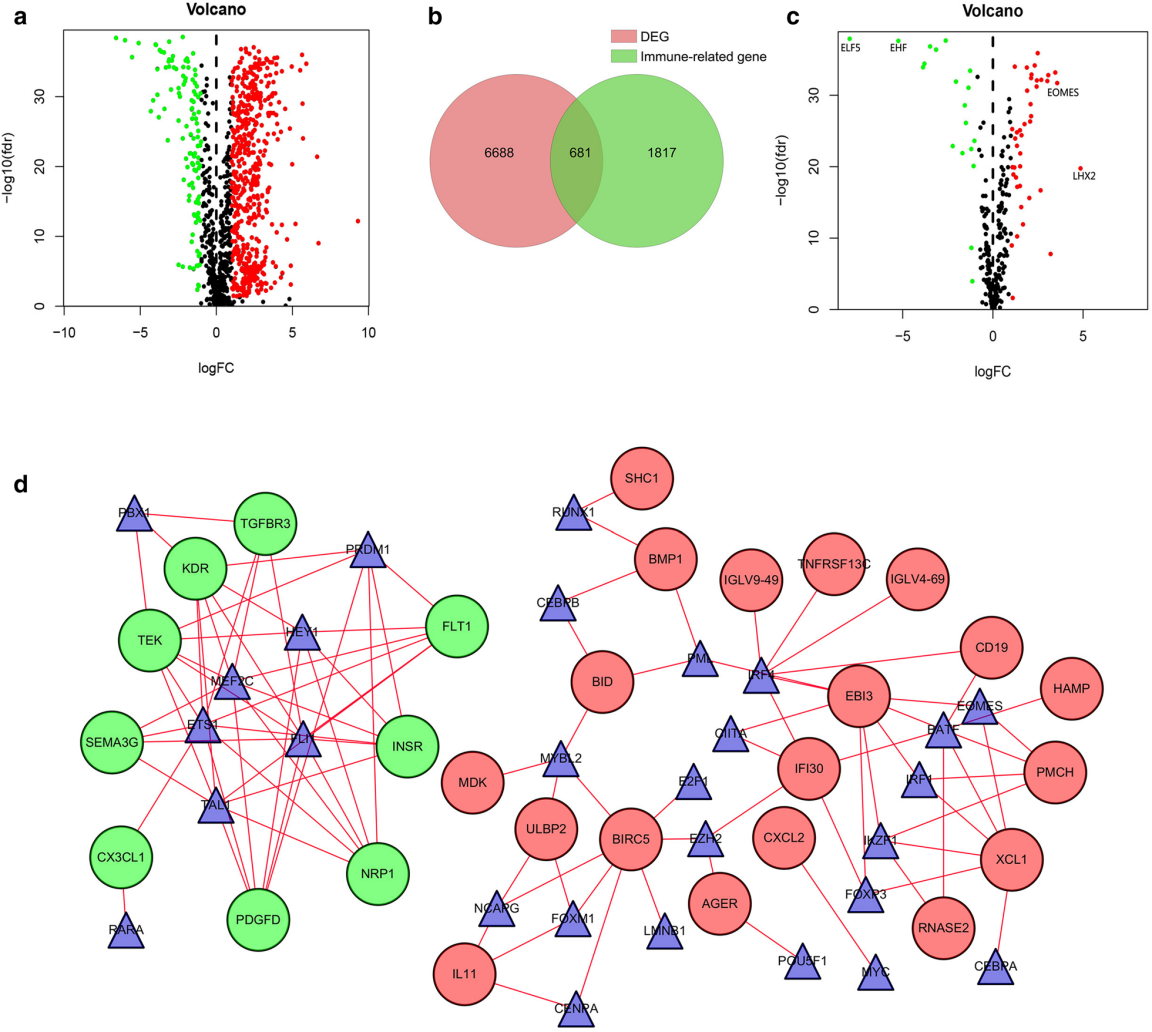
Screening of ccRCC immune-related DEGs and construction of TF regulatory network.** a** The volcano plot visualized 1817 immune-related genes based on the TCGA database. Red indicated high expression and green indicated low expression. **b** Using Venn algorithm to obtain 681 immune-related DEGs in ccRCC. **c** The volcano plot of 318 TFs based on the TCGA database. Red indicated high expression and green indicated low expression. **d** A TF regulatory network was constructed using differentially expressed TFs. Blue triangles represented TFs, red circles represented positively regulated genes, and green circles represented negatively regulated genes

All 611 samples in the data set consisted of 539 ccRCC samples and 72 adjacent non-tumor kidney samples. |log2FC| > 1 and FDR < 0.05 were used as thresholds. We screened a total of 7,369 DEGs, including 5467 up-regulated genes and 1902 down-regulated genes (Additional file 1: Table S1). The volcano plot was performed to represent DEGs between ccRCC tissues and normal tissues significantly (Additional file 1: Fig. S1). Then it overlapped with the list containing 2498 immune genes downloaded from the Immport database. As shown in Fig. 1a, the volcano plot showed all immune-related DEGs. Figure 1b showed 681 immune-related DEGs, of which 565 were up-regulated and 116 were down-regulated (Additional file 1: Table S2).

The interaction between TFs and genes is an essential step in the regulation of gene expression. In abnormal tissues, the interaction between TFs and genes may change significantly. Therefore, in order to define a common set of diagnostic goals for ccRCC, we downloaded tumor-related TFs, and then extracted the expression values of these 318 TFs from the TCGA cohort. Using |log2FC| > 1 and FDR < 0.05 as thresholds, and doing a propensity analysis to get a volcano plot (Fig. 1c), 60 differentially expressed TFs were obtained, including 41 up-regulated TFs and 19 down-regulated TFs. Cistrome cancer was a comprehensive database of predicted TF targets and enhancer profiles from TCGA expression profiling cancers. To study the regulatory relationship between immune-related DEGs and differentially expressed TFs previously obtained in ccRCC, correlation analysis was performed on them, with cor > 0.4 and p < 0.05 as the cutoff criteria. The results were imported into Cytoscape for visualization (Fig. 1d). It can be seen that in this study, 20 up-regulated TFs that were highly related to immune-related DEGs were BATF, CEBPA, E2F1, EOMES, EZH2, FOXM1 and so on. There were 8 down-regulated TFs such as PBX1, PRDM1, HEY1. In short, the construction of a regulatory network of immune genes and TFs provided a reference for further research on immune genes. They provided predictions for the mechanisms or pathways of immune genes causing or inhibiting tumor, which is conducive to exploring tumor immune regulation.

### Acquisition of prognostic‐related immune genes and their GO enrichment and KEGG pathway analysis

After integrating 681 immune-related DEGs mRNA expression profiles and clinical information, we screened 35 immune genes highly correlated with prognosis by univariate regression analysis (Additional file 1: Fig. S2a). The boxplot showed differences in expression levels of these genes between tumor tissues and normal tissues (Additional file 1: Fig. S2b).

**Fig. 2:**
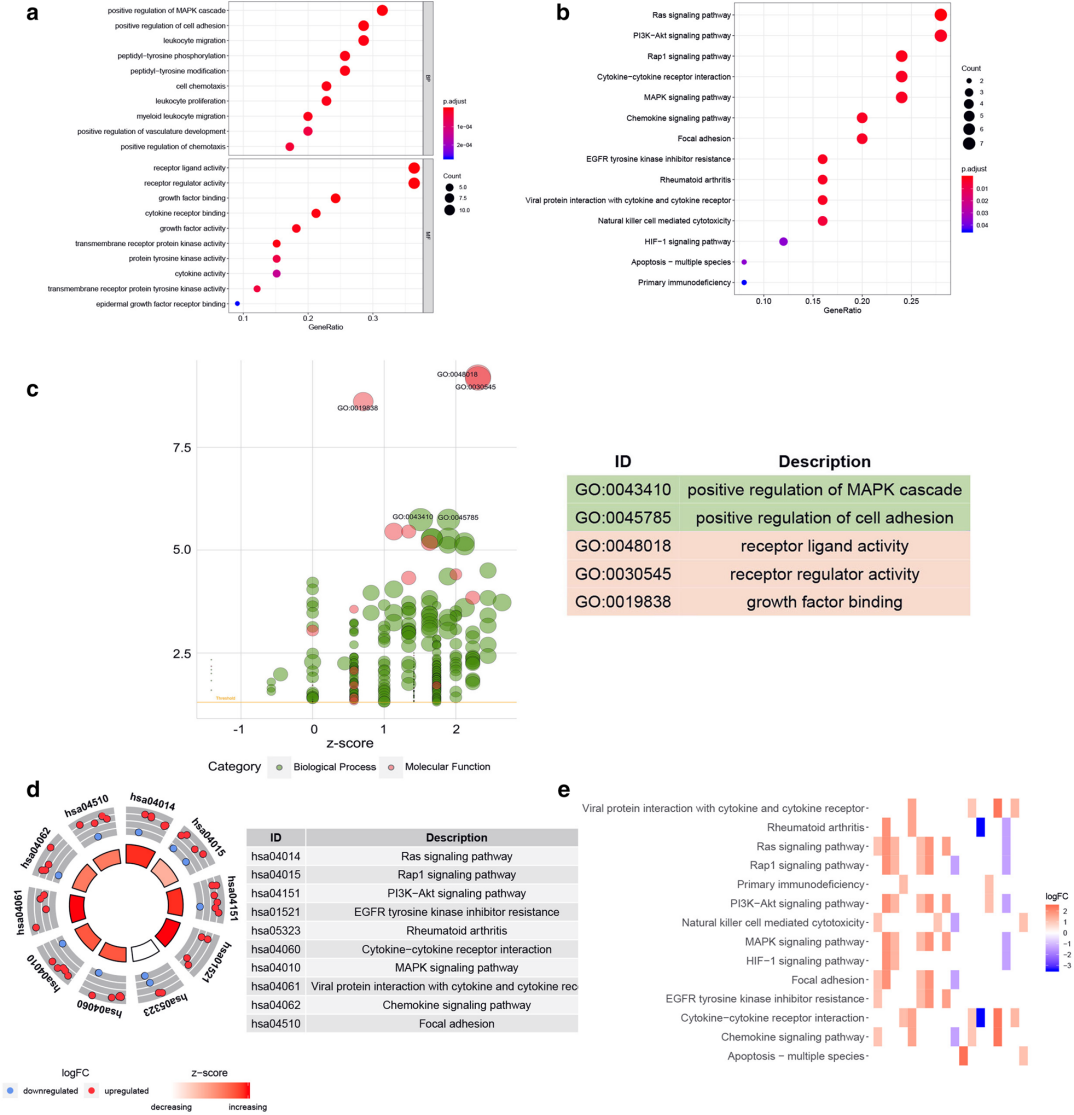
Functional annotation of prognosis-related immune genes.** a** Performed GO analysis on immune genes related to prognosis. **b** KEGG pathway analysis of immune genes related to prognosis. **c** The x-axis represented the z-score, the y-axis represented the negative logarithm of the P-value, and the size of the circle was proportional to the number of genes. Green circles correspond to the biological process and red indicated the molecular function. **d** The outer circle showed a scatter plot for each term of the logFC of the assigned genes. Red displayed increase, and blue displayed decrease. **e** The heatmap showed the correlation between prognostic immune genes and pathways

We performed GO analysis and KEGG pathway enrichment on the aforementioned immune genes related to prognosis to study the immune-related biological processes in ccRCC. The biological process of gene ontology analysis was mainly related to “positive regulation of MAPK cascade”, “positive regulation of cell adhesion”, “leukocyte migration” and so on. The molecular functions were mainly concentrated in “receptor ligand activity”, “receptor regulator activity” and “growth factor binding” (Fig. 2a). The results of KEGG pathway enrichment at the top of several pathways were related to immune pathways, such as “Ras signaling pathway”, “PI3K-Akt signaling pathway”, “Rap1 signaling pathway” and “EGFR tyrosine kinase inhibitor resistance” (Fig. 2b). All of these indicated that the selected immune genes related to prognosis were highly represented in the immune pathway, providing an accurate pathway basis for the further study of immunity. As shown in Fig. 2c, d, the Z-score greater than zero indicated a greater likelihood of enrichment in these pathways; on the contrary, a Z-score less than zero indicated a less likelihood of enrichment in these pathways. In addition, the heatmap showed the relationship between immune-related genes and pathways (Fig. 2e). The threshold was p < 0.05. Information on function notes was listed in Additional file 1 Tables S3 and S4.

### Construction of risk model of immune‐related genes

**Fig. 3:**
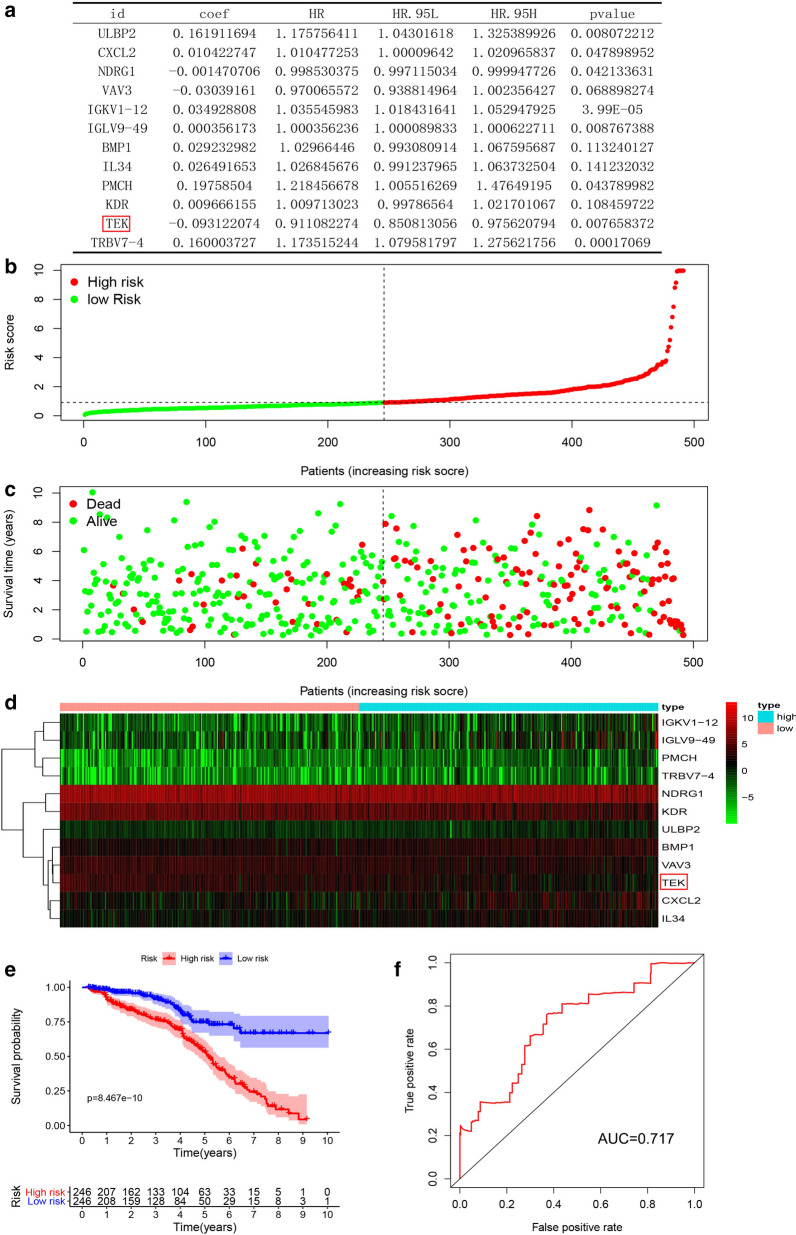
Construction of immune genes risk model.** a** Multivariate analysis of the 12 immune genes that made up the risk model. **b** The risk index distribution of ccRCC patients in the training data set. **c** The survival status chart of ccRCC patients is based on the TCGA cohort. **d** The heatmap of 12 hub immune genes is based on the TCGA cohort. **e** Risk model survival curve analysis. **f** ROC curve to verify the ability of the risk model to predict prognosis

In order to improve the robustness, we continued to analyze the 35 prognostic-associated immune genes that were previously screened. We used the “survival” package of R software, built the model with Coxp function, and optimized the model with step function. Finally, we obtained a multivariate cox model consisting of 12 prognostic-related immune genes (Fig. 3a) and calculated the patient’s risk score. Based on the risk score, we plotted risk value graphs, survival status charts, and survival heat maps (Fig. 3b–d). As can be clearly seen in the survival chart, the number of patients and survival time decreased significantly as the risk score increased. The survival heat map also showed that these 12 immune genes significantly correlated with the risk score. The survival curve showed an increase in the immune risk score indicated a poorer survival rate (Fig. 3e; p < 0.001). In order to evaluate the prognostic accuracy of the established ccRCC patient model, we performed a ROC curve analysis. The AUC of the risk model was 0.717 (Fig. 3f), which showed the accuracy of the immune gene risk model in the TCGA data set in predicting survival.

### Clinical independent prognostic analysis and screening and validation of hub genes

**Fig. 4:**
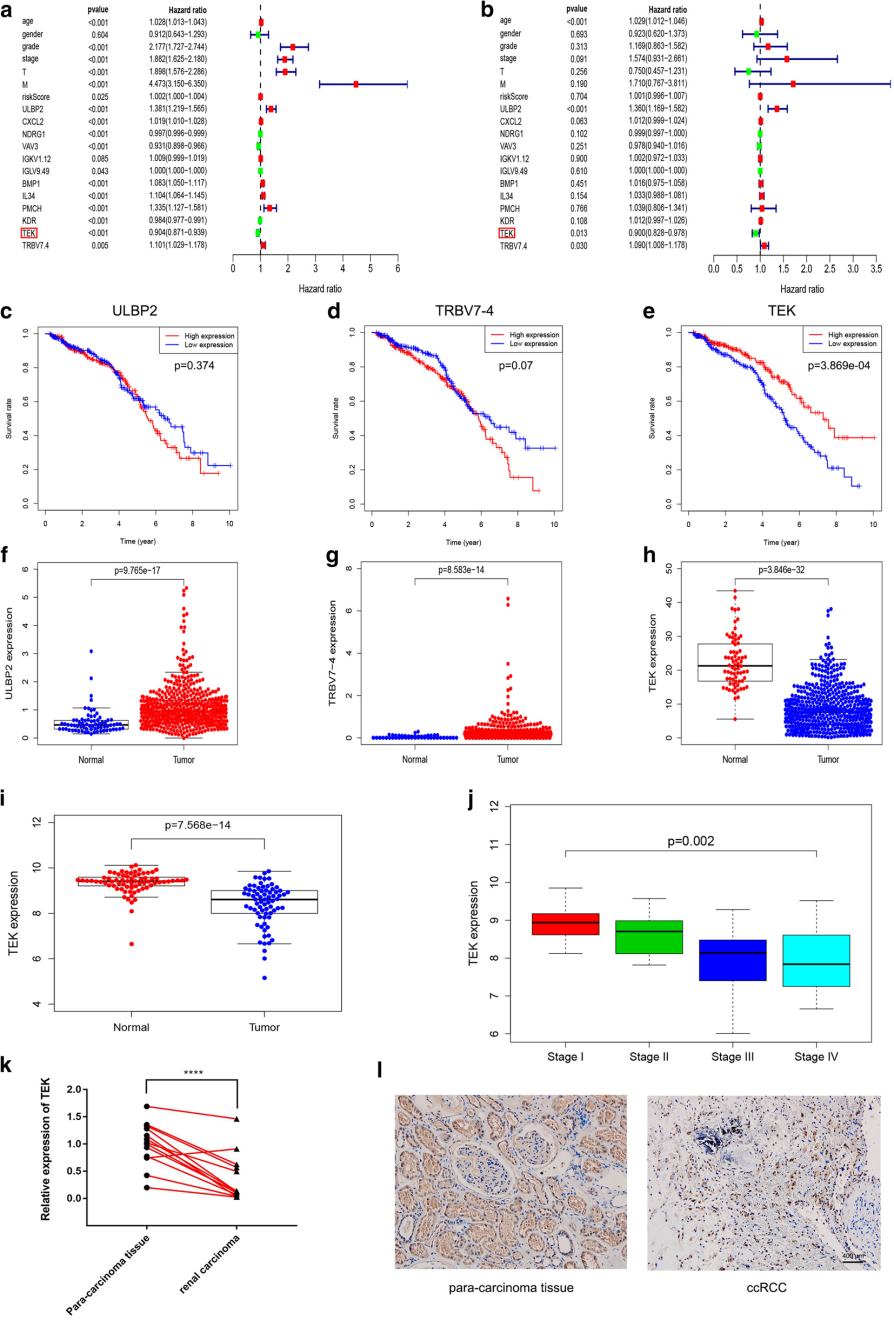
Screening and validation of hub genes.** a**,** b** Univariate and multivariate analysis of overall survival rate of ccRCC patients. **c–h** Tissue expression difference analysis and survival analysis of three hub genes based on the TCGA cohort. **i** TEK mRNA expression difference was analyzed based on GSE53757 dataset. **j** Correlation analysis between *TEK* and pathological stages (based on GSE53757 dataset). **k**,** l** Analyze the differential expression of TEK in tumor and adjacent tissues by qPCR and IHC experiments

First of all, univariate Cox analysis was executed on the TCGA cohort after integrating the mRNA expression profiles and clinical information of 12 hub genes (ULBP2, CXCL2, NDRG1, VAV3, IGKV1-12, IGLV9-49, BMP1, IL34, PMCH, KDR, TEK, TRBV7-4). As shown in Fig. 4a, age (ref. low), grade (ref. grade1), stage (ref. stage1), pT (ref. T1), pM (ref. M0), riskscore (ref. low), ULBP2, CXCL2, NDRG1, VAV3, IGLV9-49, BMP1, IL34, PMCH, KDR, TEK, TRBV7-4 (ref. low) had been proven to be an important predictor for patients with ccRCC (p < 0.05). Multivariate Cox analysis indicated that poor prognosis was significantly related to age (ref. low; HR = 1.029, p < 0.001), ULBP2 expression (ref. low; HR = 1.360, p < 0.001), TEK expression (ref. low; HR = 0.900, p = 0.013) and TRBV7-4 (ref. low; HR = 1.090, p = 0.030) (Fig. 4b). We then selected ULBP2, TEK, and TRBV7-4, three immune genes with significant differences in univariate and multivariate cox analysis for further analysis. As shown in Fig. 4c–h, among these three hub genes, compared with normal kidney tissues, the expression of *ULBP2* and *TRBV-4* mRNA found in ccRCC tissues was significantly increased, while *TEK* mRNA expression in ccRCC tissues was significantly reduced. Survival analysis showed that the decrease in *TEK* mRNA expression was significantly associated with low survival rates (p < 0.001). However, there was no significant correlation between *ULBP2* and *TRBV7-4* mRNA expression and survival.

After extracting the *TEK* mRNA expression profile and clinical information, we performed a clinical correlation analysis in the TCGA cohort. As demonstrated in Additional file 1: Fig. S3, TEK was significantly related to age (p = 0.018), grade (p < 0.001), stage (p < 0.001), pT stage (p < 0 0.001), and pM stage (p < 0.001). In addition, according to the GEPIA database, high expression of TEK significantly increases the survival rate of patients (Additional file 1: Fig. S4a, b), indicating that TEK was a prognostic biomarker for ccRCC. It can be known from Additional file 1: Fig. S4c, d that the protein expression of TEK gene in ccRCC tissues was significantly lower than that in normal tissues, which were provided and confirmed by The Human Protein Atlas database. The GSE53757 dataset was then used to validate the expression level and clinical stage of *TEK* gene, demonstrating that TEK expression in ccRCC tissues was down-regulated (p < 0.001) and significantly correlated with clinical stage (p = 0.002) (Fig. 4i, j). In addition, the qPCR and IHC results of clinical tissue samples collected in our hospital further confirmed the above conclusions (Fig. 4k, l).

### Immune infiltration of TEK

**Fig. 5:**
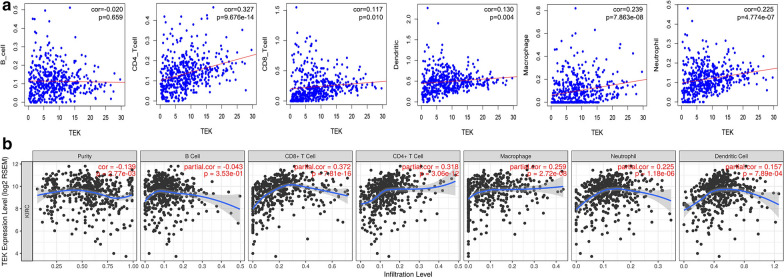
Immune infiltration of TEK. ** a** Correlation analysis between TEK and immune infiltration level of ccRCC was performed. **b** Based on the Timer database, the correlation between TEK and ccRCC immune infiltration level was shown. The generation of scatterplot had partial Spearman correlation and statistical significance

After identifying the prognostic value and clinical relevance of TEK, we investigated TEK’s immune infiltration. From TIMER data, we downloaded the content of immune cells in each sample in TCGA and performed correlation analysis with *TEK* expression. Increased *TEK* expression was significantly associated with the infiltration of CD4^+^ T cells, CD8^+^ T cells, macrophages, neutrophils, and dendritic cells (p < 0.05) (Fig. 5a). Moreover, we analyzed the TEK gene in the TIMER database, and *TEK* expression was significantly related to purity (correlation coefficients were − 0.139). Analysis of TIMER database showed that the increase of *TEK* expression was strongly correlated with the infiltration of B cells, CD4^+^ T cells, CD8^+^ T cells, macrophages, neutrophils, and dendritic cells (p < 0.05) (Fig. 5b).

### Knockdown of TEK promoted ccRCC cell proliferation and migration

**Fig. 6:**
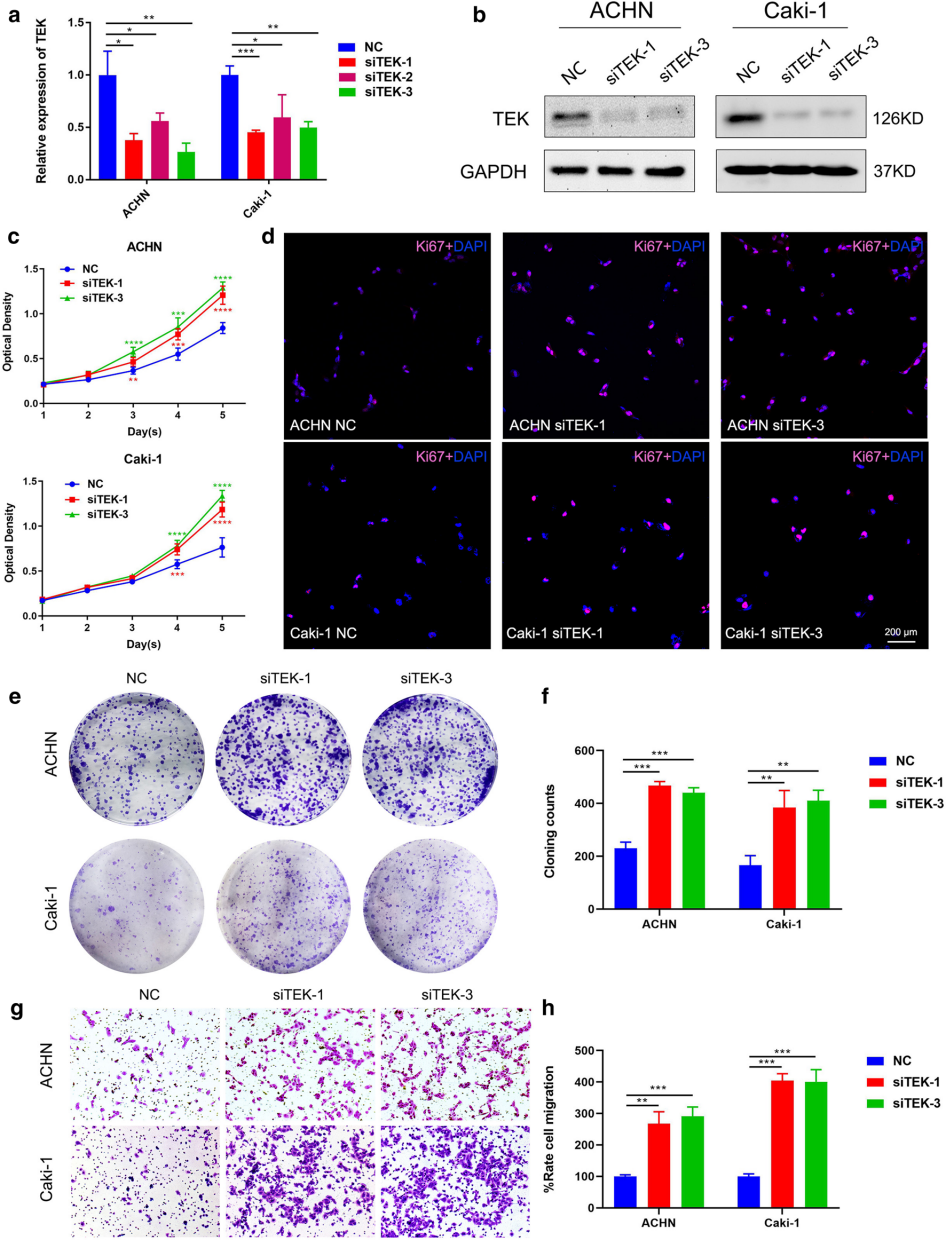
TEK knockdown promoted the proliferation and migration of ccRCC cells.** a**,** b** Verification of TEK-siRNA silencing efficacy on the mRNA and protein levels of ACHN cells and Caki-1 cells. **c** MTT assay detected the effect of *TEK* silencing on cell proliferation. **d** Immunofluorescence staining of Ki-67. **e** The cloning formation assay evaluated the effect of silencing *TEK* on cloning formation ability. **f** Statistical analysis of cloning formation assay. **g** The effect of *TEK* silencing on cell migration was evaluated by transwell assay. **h** Statistical analysis of transwell assay

First of all, *TEK* knockdown efficiency was verified, and the results showed that siTEK-1 and siTEK-3 showed higher knockdown efficiency (Fig. 6a, b). MTT assay showed that *TEK* knockdown significantly promoted the proliferation of ccRCC cells (Fig. 6c). The immunofluorescence staining of the proliferation marker Ki-67 also proved that the knockdown of *TEK* could increase the number of Ki-67 positive cells (Fig. 6d). In addition, the results of the cloning formation assay indicated that knockdown of *TEK* could increase the cloning ability of ccRCC cells (Fig. 6e, f). Transwell assay was then performed to evaluate the effect of TEK on cell migration, and the results showed that *TEK* knockdown significantly improved the migration of ccRCC cells compared with the control group (Fig. 6g, h). In short, *TEK* knockdown could promote the proliferation and migration of ccRCC cells.

### TEK knockdown promoted the EMT and inhibited apoptosis in ccRCC cells by promoting AKT phosphorylation

**Fig. 7:**
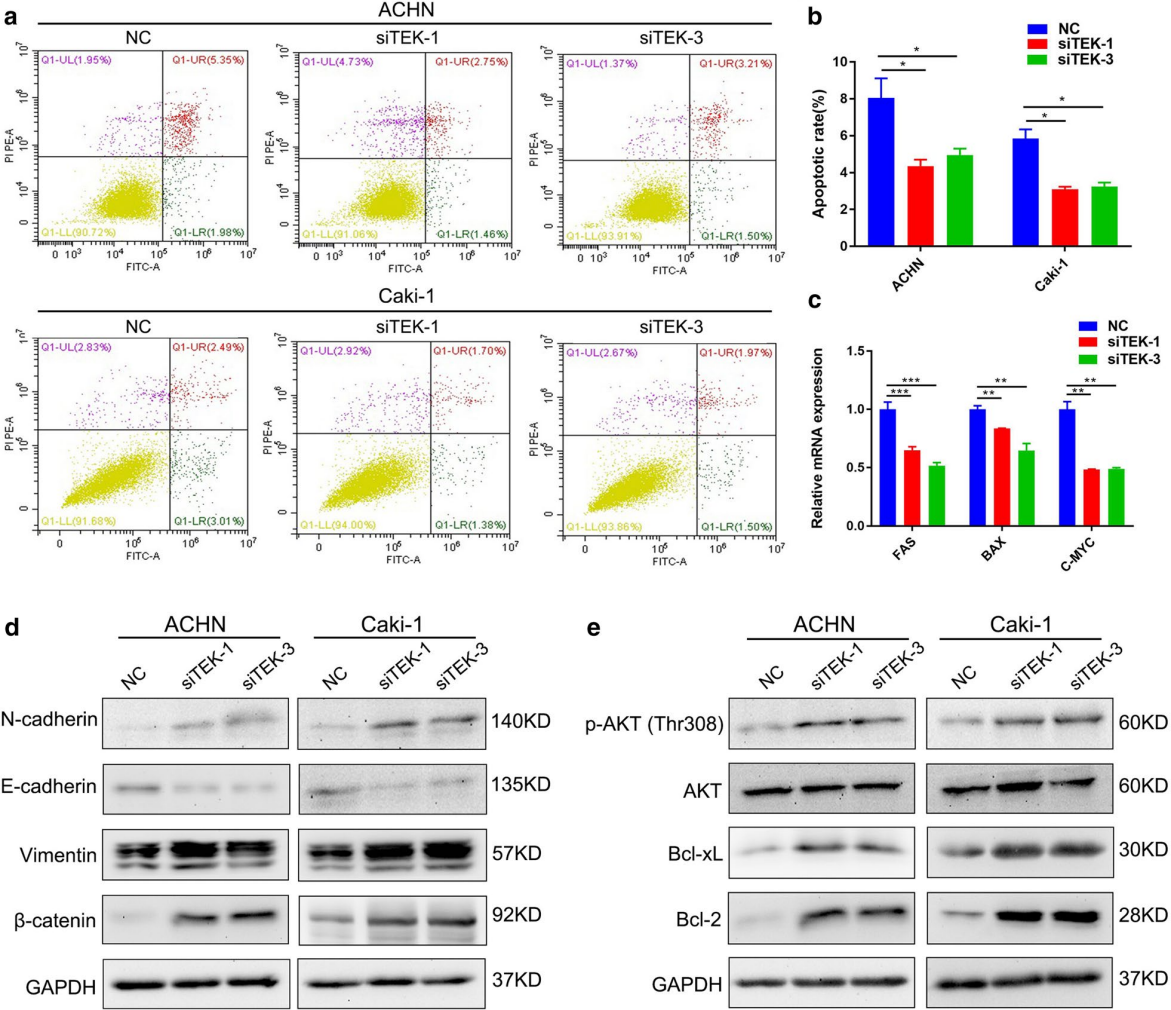
TEK could affect the EMT and apoptosis of ccRCC cells by regulating AKT phosphorylation. **a** Flow cytometry analyzed the effect of *TEK* knockdown on ccRCC cell apoptosis. **b** Statistical analysis of Flow cytometry analysis. **c** Analysis of mRNA levels of apoptosis-related genes in ccRCC cells after *TEK* knockdown. **d** Western blot analysis detected EMT-related proteins in *TEK* knockdown ccRCC cells. **e** Western blot analysis was performed to detect apoptosis-related proteins and phosphorylated AKT in *TEK* knockdown ccRCC cells

Next, we evaluated the effect of TEK on apoptosis of ccRCC cells by flow cytometry, and the results showed that *TEK* knockdown could effectively inhibit apoptosis (Fig. 7a, b). qRT-PCR also confirmed the reduction of apoptosis-related genes in the *TEK* knockdown group (Fig. 7c). In order to explain the changes in cell phenotypes caused by *TEK* knockdown, Western blot analysis was performed. First, we detected EMT-related proteins, and the results showed that *TEK* knockdown significantly increased the expression of N-cadherin, Vimentin, β-catenin, and decreased the expression of E-cadherin (Fig. 7d). Consistent with flow cytometry analysis, Western blot results showed that *TEK* knockdown increased the expression of c-Myc, Bcl-2 and Bcl-xL. In addition, down-regulation of *TEK* strongly induced phosphorylated AKT in ccRCC cells, which was consistent with our results of KEGG pathway analysis (Fig. 7e).

## Discussion

More and more evidence shows that immune cells in the TME significantly affect tumor occurrence and development [30]. RCC is characterized by high immunogenicity, accompanied by obvious infiltration of immune cells. [31]. Tumor CD8^+^ T and NK cells are differentiated effector cells with lysed particles [32]. In addition, some CD8^+^ T cells express tumor-reactive T-cell receptor (TCR) when analyzed *in vitro* after exposure to IL-2 [33] and mediate anti-tumor reactivity [32]. The high frequency of NK cells in lymphocyte infiltration seems to herald a better prognosis [34]. Nevertheless, tumors are growing despite potential tumor-reactive cytotoxic effector cell infiltration, suggesting that their anti-tumor activity is impaired within the TME.

In this study, bioinformatics methods were used to screen specific immune-related prognostic markers of ccRCC based on the TCGA database. On this basis, we screened 35 immune genes that are closely associated with prognosis through univariate cox prognostic analysis, performed GO and KEGG analysis and constructed TF networks to further explore the molecular mechanism of these genes. Then multivariate cox model was used to identify 12 hub genes related to the progress and prognosis of ccRCC patients, and the corresponding risk model was constructed. In addition, clinically related univariate, multivariate cox analysis and survival analysis indicated that TEK was an independent prognostic factor for ccRCC patients and further validated it. The clinical correlation analysis further indicated that TEK was closely related to the clinicopathological traits of ccRCC. Importantly, through the Person’s correlation analysis, we demonstrated a high correlation between *TEK* expression and immune cell infiltration.

The Ang–Tie signaling system regulates blood and lymphatic growth. Angiopoietin-1 activates the tyrosine kinase receptor TEK (also known as Tie2) and is mainly expressed on endothelial cells. Activation and phosphorylation of TEK lead to downstream signaling, promoting vascular maturation and endothelial cell survival [35]. Angiopoietin-1/TEK signaling is important for vascular integrity, TEK knockout results in a significant increase in metastatic tumor cells in the lung [36]. Furthermore, TEK activation normalizes the structure and function of tumor vessels, thereby delaying tumor growth, slowing the metastatic process and enhancing the response to concomitant cytotoxic treatments [37]. Hence, we could speculate that the decrease of *TEK* expression in ccRCC might be the main reason for tumor hypoxia and subsequent invasion and metastasis, because the lack of TEK can cause the instability of blood vessels, resulting in impeded blood perfusion and increased permeability and leakage of blood vessels.

Other studies have shown that TEK activation favorably changes TME and immune infiltration, polarizes tumor-associated macrophages (TAM) toward M1-like phenotypes and reduces regulatory T cell (Treg) infiltration [38]. According to the general concept, polarization towards the M1-like phenotype has anti-tumor effects [39], Tregs hinder defense against tumors [40]. In addition, TEK could enhance mast cell adhesion to VCAM-1, mediating mast cell activation and contributing to the occurrence of immune response [41]. It can be seen that TEK may be used as a new immune-related biomarker to regulate the occurrence and development of ccRCC. Interestingly, HA et al. performed a survival analysis of TEK and found that TEK in ccRCC can be used as a prognostic marker, consistent with our findings [42]. We have proved that TEK was significantly down-regulated in ccRCC tissues through qPCR and IHC experiments (Fig. 4k, l). In addition, *in vitro* experiments showed that *TEK* knockdown could promote the proliferation and migration of ccRCC cells, suggesting that TEK may be a good prognostic marker for ccRCC. Flow cytometry analysis showed that the down-regulation of *TEK* inhibited cell apoptosis (Fig. 7a, b). Then we performed a correlation analysis of apoptosis-related genes in the GEPIA database (Additional file 1: Fig. S5) and performed a qRT-PCR experiment (Fig. 7c). The results showed TEK gene is significantly related to apoptosis-related genes FAS, BAX, and c-Myc. To explain the effect of TEK on the migration of ccRCC cells, we completed Western blot assay and found that the down-regulation of *TEK* significantly affected the expression of EMT-related proteins (Fig. 7d). According to the previous analysis of the KEGG pathway, the results showed that TEK is significantly related to immune-related pathways, such as “Ras signaling pathway”, “PI3K-AKT signaling pathway” and “Rap1 signaling pathway”, which provided a certain basis for TEK to regulate immune-related pathways of ccRCC. In order to explore the regulatory effect of TEK on cell apoptosis, Western blot analysis showed that down-regulation of *TEK* could promote AKT phosphorylation and affect downstream apoptosis-related proteins, thereby inhibiting cell apoptosis (Fig. 7e). The above results indicated that *TEK* knockdown promoted the proliferation and migration of ccRCC cells, and affected cell apoptosis by regulating the phosphorylation of AKT.

The major limitation of our study is the lack of sufficient clinical cohorts to validate the accuracy of the risk model. Our next research plan is to validate the model through prospective studies and clarify TEK’s mechanism of action on ccRCC progress through molecular biology experiments. In conclusion, we have constructed a risk model and identified TEK, an immune-related gene associated with the progression of ccRCC, which could play an important role in the risk assessment and prognosis prediction of ccRCC patients.

## Supplementary Information

**Additional file 1:** Additional Figures and Tables.

## Data Availability

The data that support the findings of this study are openly available in Gene Expression Omnibus (GEO) database at http://www.ncbi.nlm.nih.gov/geo/, and The Cancer Genome Atlas (TCGA) database at https://genomecancer.ucsc.edu/.

## Abbreviations

ccRCC: Clear cell renal cell carcinoma
DEG: Differentially expressed gene
GO: Gene ontology
IHC: Immunohistochemistry
KEGG: Kyoto encyclopedia of genes and genomes
RCC: Renal cell carcinoma
TCGA: The Cancer Genome Atlas
TF: Transcription factor
TIMER: Tumor Immune Estimation Resource
TME: Tumor microenvironment

## References

[1] Capitanio U, Bensalah K, Bex A, Boorjian SA, Bray F, Coleman J, Gore JL, Sun M, Wood C, Russo P (2019). Epidemiology of Renal Cell Carcinoma. Eur Urol.

[2] Siegel RL, Miller KD, Jemal A (2018). Cancer statistics, 2018. CA Cancer J Clin.

[3] Wong MCS, Goggins WB, Yip BHK, Fung FDH, Leung C, Fang Y, Wong SYS, Ng CF (2017). Incidence and mortality of kidney cancer: temporal patterns and global trends in 39 countries. Sci Rep.

[4] Hsieh JJ, Purdue MP, Signoretti S, Swanton C, Albiges L, Schmidinger M, Heng DY, Larkin J, Ficarra V (2017). Renal cell carcinoma. Nat Rev Dis Primers.

[5] Klatte T, Stewart GD (2018). Renal cell carcinoma: standards and controversies. World J Urol.

[6] Li JR, Ou YC, Yang CK, Wang SS, Chen CS, Ho HC, Cheng CL, Yang CR, Chen CC, Wang SC (2018). The Impact of Local Intervention Combined with Targeted Therapy on Metastatic Renal Cell Carcinoma. Anticancer Res.

[7] George S, Rini BI, Hammers HJ (2019). Emerging Role of Combination Immunotherapy in the First-line Treatment of Advanced Renal Cell Carcinoma: A Review. JAMA Oncol.

[8] Rijnders M, de Wit R, Boormans JL, Lolkema MPJ, van der Veldt AAM (2017). Systematic Review of Immune Checkpoint Inhibition in Urological Cancers. Eur Urol.

[9] Quail DF, Joyce JA (2013). Microenvironmental regulation of tumor progression and metastasis. Nat Med.

[10] Senbabaoglu Y, Gejman RS, Winer AG, Liu M, Van Allen EM, de Velasco G, Miao D, Ostrovnaya I, Drill E, Luna A (2016). Tumor immune microenvironment characterization in clear cell renal cell carcinoma identifies prognostic and immunotherapeutically relevant messenger RNA signatures. Genome Biol.

[11] Escudier B (2012). Emerging immunotherapies for renal cell carcinoma. Ann Oncol.

[12] Hui L, Chen Y (2015). Tumor microenvironment: Sanctuary of the devil. Cancer Lett.

[13] Wu T, Dai Y (2017). Tumor microenvironment and therapeutic response. Cancer Lett.

[14] Liu C, Peng W, Xu C, Lou Y, Zhang M, Wargo JA, Chen JQ, Li HS, Watowich SS, Yang Y (2013). BRAF inhibition increases tumor infiltration by T cells and enhances the antitumor activity of adoptive immunotherapy in mice. Clin Cancer Res.

[15] Peng W, Chen JQ, Liu C, Malu S, Creasy C, Tetzlaff MT, Xu C, McKenzie JA, Zhang C, Liang X (2016). Loss of PTEN Promotes Resistance to T Cell-Mediated Immunotherapy. Cancer Discov.

[16] Parsa AT, Waldron JS, Panner A, Crane CA, Parney IF, Barry JJ, Cachola KE, Murray JC, Tihan T, Jensen MC (2007). Loss of tumor suppressor PTEN function increases B7-H1 expression and immunoresistance in glioma. Nat Med.

[17] Spranger S, Bao R, Gajewski TF (2015). Melanoma-intrinsic beta-catenin signalling prevents anti-tumour immunity. Nature.

[18] Gu P, Chen H (2014). Modern bioinformatics meets traditional Chinese medicine. Brief Bioinform.

[19] Huang MD, Huang AH (2015). Bioinformatics Reveal Five Lineages of Oleosins and the Mechanism of Lineage Evolution Related to Structure/Function from Green Algae to Seed Plants. Plant Physiol.

[20] Blattner M, Lee DJ, O’Reilly C, Park K, MacDonald TY, Khani F, Turner KR, Chiu YL, Wild PJ, Dolgalev I (2014). SPOP mutations in prostate cancer across demographically diverse patient cohorts. Neoplasia.

[21] Chen L, Yuan L, Wang G, Cao R, Peng J, Shu B, Qian G, Wang X, Xiao Y (2017). Identification and bioinformatics analysis of miRNAs associated with human muscle invasive bladder cancer. Mol Med Rep.

[22] Dahinden C, Ingold B, Wild P, Boysen G, Luu VD, Montani M, Kristiansen G, Sulser T, Buhlmann P, Moch H (2010). Mining tissue microarray data to uncover combinations of biomarker expression patterns that improve intermediate staging and grading of clear cell renal cell cancer. Clin Cancer Res.

[23] Gerlinger M, Horswell S, Larkin J, Rowan AJ, Salm MP, Varela I, Fisher R, McGranahan N, Matthews N, Santos CR (2014). Genomic architecture and evolution of clear cell renal cell carcinomas defined by multiregion sequencing. Nat Genet.

[24] Tomczak K, Czerwinska P, Wiznerowicz M (2015). The Cancer Genome Atlas (TCGA): an immeasurable source of knowledge. Contemp Oncol (Pozn).

[25] Ritchie ME, Phipson B, Wu D, Hu Y, Law CW, Shi W, Smyth GK (2015). limma powers differential expression analyses for RNA-sequencing and microarray studies. Nucleic Acids Res.

[26] Yu G, Wang LG, Han Y, He QY (2012). clusterProfiler: an R package for comparing biological themes among gene clusters. OMICS.

[27] Ashburner M, Ball CA, Blake JA, Botstein D, Butler H, Cherry JM, Davis AP, Dolinski K, Dwight SS, Eppig JT (2000). Gene ontology: tool for the unification of biology. The Gene Ontology Consortium. Nat Genet.

[28] Kanehisa M. The KEGG database. Novartis Foundation symposium 2002, 247:91–101; discussion 101–103, 119–128, 244 – 152. 12539951

[29] Smoot ME, Ono K, Ruscheinski J, Wang PL, Ideker T (2011). Cytoscape 2.8: new features for data integration and network visualization. Bioinformatics.

[30] Gajewski TF, Schreiber H, Fu YX (2013). Innate and adaptive immune cells in the tumor microenvironment. Nat Immunol.

[31] Noessner E, Brech D, Mendler AN, Masouris I, Schlenker R, Prinz PU (2012). Intratumoral alterations of dendritic-cell differentiation and CD8(+) T-cell anergy are immune escape mechanisms of clear cell renal cell carcinoma. Oncoimmunology.

[32] Prinz PU, Mendler AN, Masouris I, Durner L, Oberneder R, Noessner E (2012). High DGK-alpha and disabled MAPK pathways cause dysfunction of human tumor-infiltrating CD8 + T cells that is reversible by pharmacologic intervention. J Immunol.

[33] Leisegang M, Turqueti-Neves A, Engels B, Blankenstein T, Schendel DJ, Uckert W, Noessner E (2010). T-cell receptor gene-modified T cells with shared renal cell carcinoma specificity for adoptive T-cell therapy. Clin Cancer Res.

[34] Eckl J, Buchner A, Prinz PU, Riesenberg R, Siegert SI, Kammerer R, Nelson PJ, Noessner E (2012). Transcript signature predicts tissue NK cell content and defines renal cell carcinoma subgroups independent of TNM staging. J Mol Med.

[35] Eklund L, Saharinen P (2013). Angiopoietin signaling in the vasculature. Exp Cell Res.

[36] Michael IP, Orebrand M, Lima M, Pereira B, Volpert O, Quaggin SE, Jeansson M (2017). Angiopoietin-1 deficiency increases tumor metastasis in mice. BMC Cancer.

[37] Goel S, Gupta N, Walcott BP, Snuderl M, Kesler CT, Kirkpatrick ND, Heishi T, Huang Y, Martin JD, Ager E (2013). Effects of vascular-endothelial protein tyrosine phosphatase inhibition on breast cancer vasculature and metastatic progression. J Natl Cancer Inst.

[38] Park JS, Kim IK, Han S, Park I, Kim C, Bae J, Oh SJ, Lee S, Kim JH, Woo DC (2016). Normalization of Tumor Vessels by Tie2 Activation and Ang2 Inhibition Enhances Drug Delivery and Produces a Favorable Tumor Microenvironment. Cancer Cell.

[39] Peterson TE, Kirkpatrick ND, Huang Y, Farrar CT, Marijt KA, Kloepper J, Datta M, Amoozgar Z, Seano G, Jung K (2016). Dual inhibition of Ang-2 and VEGF receptors normalizes tumor vasculature and prolongs survival in glioblastoma by altering macrophages. Proc Natl Acad Sci USA.

[40] Facciabene A, Motz GT, Coukos G (2012). T-regulatory cells: key players in tumor immune escape and angiogenesis. Cancer Res.

[41] Kanemaru K, Noguchi E, Tokunaga T, Nagai K, Hiroyama T, Nakamura Y, Tahara-Hanaoka S, Shibuya A (2015). Tie2 Signaling Enhances Mast Cell Progenitor Adhesion to Vascular Cell Adhesion Molecule-1 (VCAM-1) through α4β1 Integrin. PloS one.

[42] Ha M, Son YR, Kim J, Park SM, Hong CM, Choi D, Kang W, Kim JH, Lee KJ, Park D (2019). TEK is a novel prognostic marker for clear cell renal cell carcinoma. Eur Rev Med Pharmacol Sci.

