# Supplementary material for: The immune‐related biomarker TEK inhibits the development of clear cell renal cell carcinoma (ccRCC) by regulating AKT phosphorylation
Source: Cancer Cell Int. 2021 Feb 18;21:119. doi: 10.1186/s12935-021-01830-1 (PMC7890987; doi:10.1186/s12935-021-01830-1)
Supplement: Supplementary file 1 — Additional file 1: Additional Figures and Tables. [file 12935_2021_1830_MOESM1_ESM.pdf]

Supplementary Data

The immune-related biomarker TEK inhibits the development of clear cell renal cell carcinoma (ccRCC) by regulating AKT phosphorylation

Siming Chen[1], Mengxue Yu[2,3,4], Lingao Ju[2,3,4,5], Gang Wang[2,3,4,5], Kaiyu Qian[2,3,4,5], Yu Xiao[1,2,3,4,5], Xinghuan Wang[1,4,6*]

[1]Department of Urology, Zhongnan Hospital of Wuhan University, Wuhan, China

[2]Department of Biological Repositories, Zhongnan Hospital of Wuhan University, Wuhan, China

[3]Human Genetics Resource Preservation Center of Hubei Province, Wuhan, China

[4]Wuhan Research Center for Infectious Diseases and Cancer, Chinese Academy of Medical Sciences, Wuhan, China

[5]Laboratory of Precision Medicine, Zhongnan Hospital of Wuhan University, Wuhan, China

[6]Medical Research Institute, Wuhan University, Wuhan, China

***Corresponding author:** Dr. Xinghuan Wang, Email: wangxinghuan@whu.edu.cn, Tel. +86-27-6781-3104.

Running title: TEK inhibits ccRCC progress

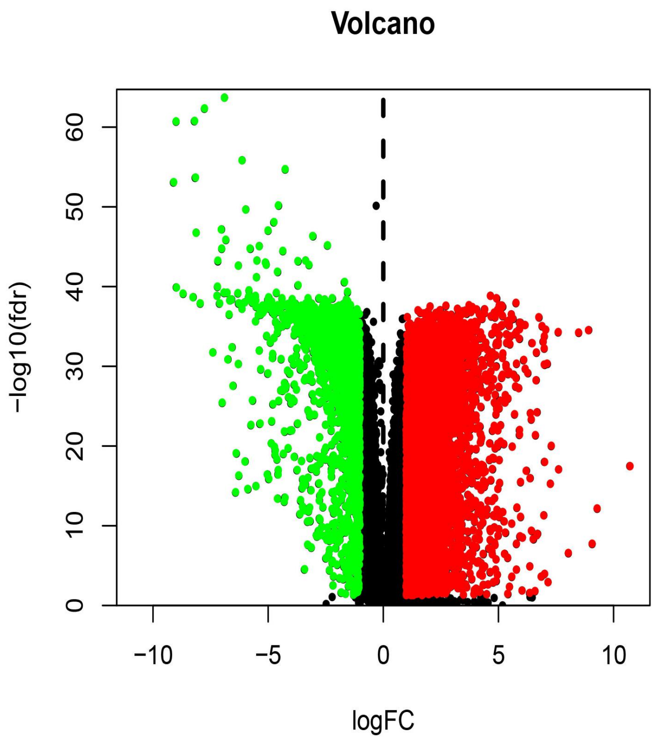

Supplementary Figure 1. Screening of differentially expressed genes (DEGs) between ccRCC and normal specimens. The volcano plot visualizes the differentially expressed genes in TCGA.

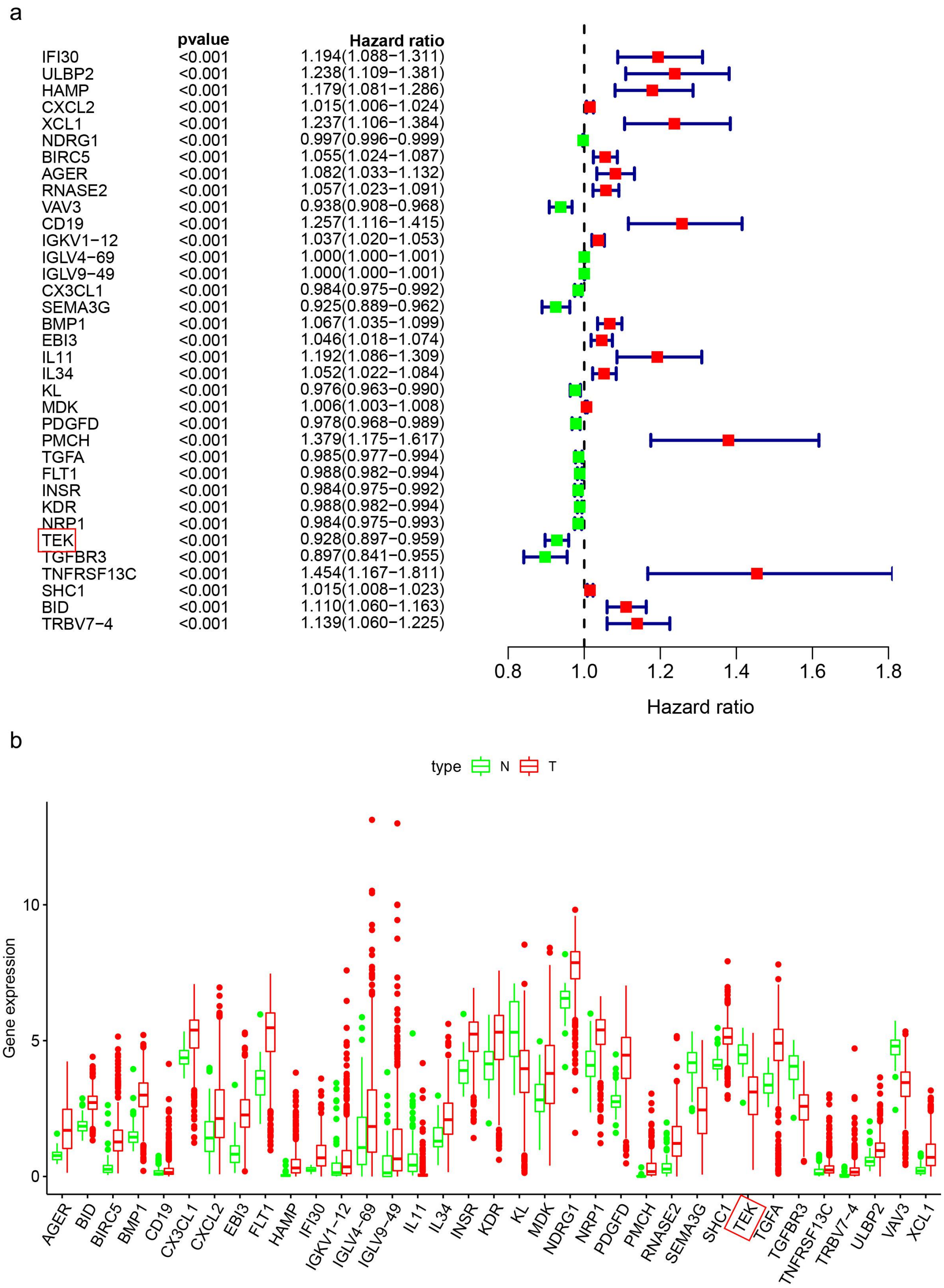

Supplementary Figure 2. Prognosis-related immune genes. (a) Forest map of 35 immune differential genes related to prognosis. **(b)** The expression patterns of 35 prognostic-related immune genes in ccRCC and matched normal samples. The red dots represented tumor samples, and green represented normal samples.

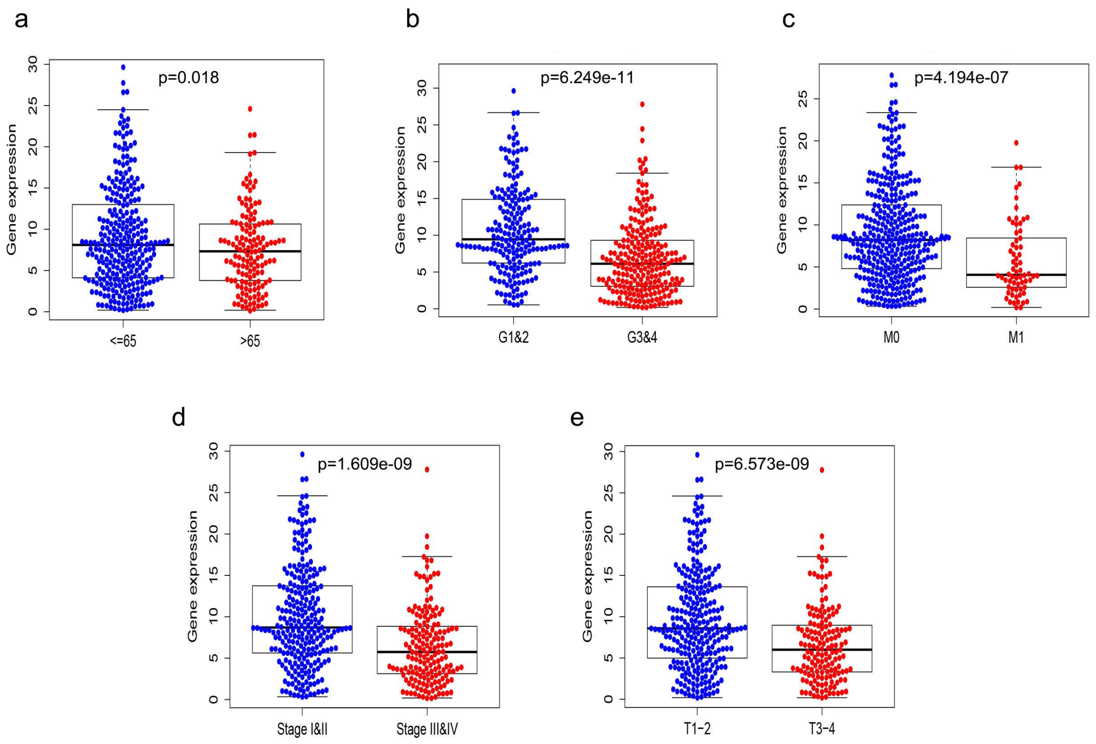

Supplementary Figure 3. The clinicopathological significance of TEK. The value of TEK varies in **(a)** age, **(b)** pathological stage, **(c)** M stage, **(d)** clinical stage, and **(e)** T stage.

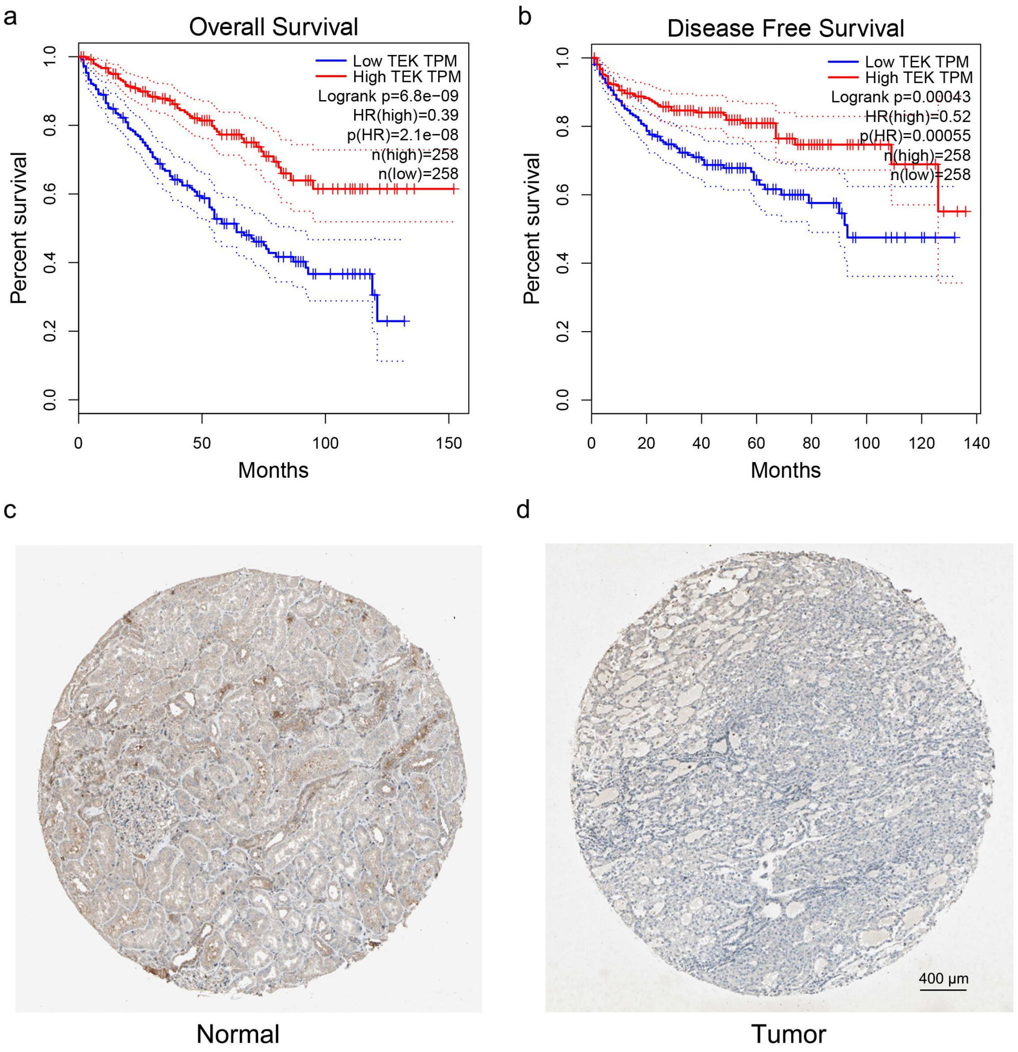

Supplementary Figure 4. Expression and survival analysis of TEK. (a-b) TEK's OS and DFS were displayed based on the GEPIA database. **(c-d)** According to the Human Protein Atlas database to analyze TEK protein level expression differences.

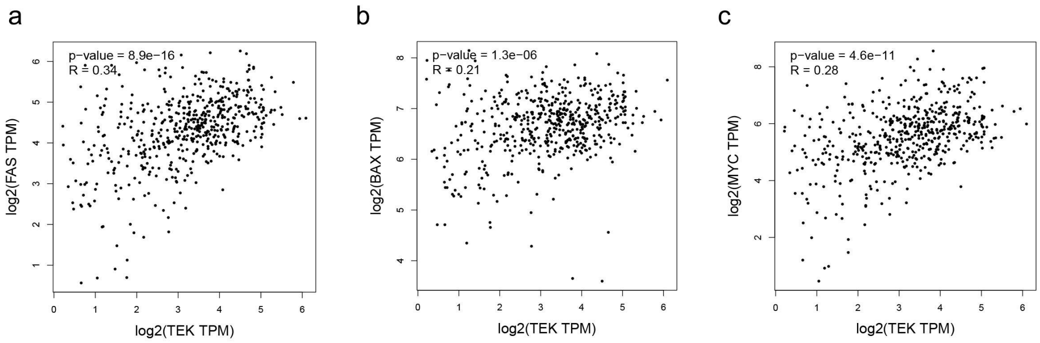

Supplementary Figure 5. Verify the correlation between FAS, BAX and MYC expression levels and TEK expression levels (based on TCGA data in GEPIA). (a) FAS, **(b)** BAX, **(c)** MYC.

Table S1. The differentially expressed genes(DEGs) were screened out in TCGA

ID	conMean	treatMean	logFC	pValue	FDR
GPR18	0.146794618	0.693482736	2.240060894	9.12E-25	4.88E-24
FAM102A	20.81446349	9.545372492	-1.124712969	7.37E-30	6.44E-29
AL135999.3	1.545612011	0.458942028	-1.751794378	9.49E-39	7.18E-37
AC007216.3	0.062909036	0.293995291	2.22445388	1.83E-16	5.28E-16
CA8	5.054649058	0.489813877	-3.367305378	2.44E-38	1.61E-36
AC073842.2	0.198957502	0.483187968	1.280124234	3.13E-08	5.39E-08
SLC23A1	11.61591379	4.028049759	-1.527949241	1.75E-05	2.53E-05
RPL35	115.7821596	242.1192943	1.06430508	5.51E-26	3.27E-25
CTSV	2.577228031	0.372850582	-2.789150692	2.04E-28	1.52E-27
LEF1	1.373004663	2.936015603	1.09652311	0.021097949	0.024538986
AC064807.2	3.8841067	1.398757052	-1.473437429	4.41E-23	2.05E-22
GRM8	0.358724128	1.490924662	2.055260669	4.93E-13	1.14E-12
AP000757.1	8.871118132	1.183177989	-2.906448837	1.56E-36	5.28E-35
IGHV4-28	0.940814095	5.01157033	2.41328115	0.000101513	0.000139507
AC040977.1	2.882706806	8.47776831	1.556260429	5.30E-33	7.58E-32
AL512770.1	0.086839521	0.374746255	2.109490393	1.06E-21	4.44E-21
LINC01320	9.481668125	24.13059716	1.347650816	1.16E-18	3.87E-18
SEMA3G	18.17338861	6.023849073	-1.593069921	5.15E-30	4.60E-29
AC010261.2	0.041846596	0.226296068	2.435029355	2.85E-15	7.62E-15
AC008083.2	0.134397147	0.3550637	1.401575368	2.27E-13	5.38E-13
AC007220.1	0.13348511	0.301095415	1.173541912	2.34E-07	3.81E-07
AL353801.3	0.088063481	0.401032331	2.187102776	1.87E-14	4.72E-14
PRG4	0.186333577	0.383416962	1.04102649	0.003667361	0.004522529
AL022067.1	0.094362041	0.32477799	1.783175331	9.67E-12	2.07E-11
MYO7A	1.176536643	2.421973827	1.041637021	4.43E-11	9.07E-11
SNORA60	0.497408982	1.617834807	1.70155984	2.11E-09	3.91E-09
TNNT2	2.292270072	0.085305232	-4.747998989	1.11E-35	2.89E-34
ORC6	0.205240259	0.525470379	1.356295691	4.73E-26	2.83E-25
AC011472.1	0.705543357	2.137253438	1.598951347	2.40E-13	5.66E-13
UGT1A10	0.066599692	0.467911281	2.812647612	0.001147985	0.001470461
PHYHIP	1.118194497	0.32076167	-1.80159749	7.08E-12	1.52E-11
KRR1P1	0.096589537	0.338571169	1.809520301	7.47E-10	1.42E-09
RNF150	8.595075058	0.989696965	-3.118451479	2.26E-40	3.88E-38
SIX4	0.910923261	0.186472412	-2.288367316	1.89E-35	4.60E-34
AP000866.4	3.290994344	1.496393156	-1.137034275	2.30E-15	6.18E-15
CENPK	0.182479053	0.703768503	1.947370079	6.52E-34	1.11E-32
RARA	7.262338653	15.19483995	1.065075367	8.71E-27	5.52E-26
AC106897.1	0.45086649	1.212784049	1.427550492	3.01E-19	1.05E-18
AC008957.1	0.08807267	0.327478725	1.894634876	4.74E-17	1.42E-16
RN7SL832P	0.713108685	0.248929532	-1.518384579	2.11E-24	1.09E-23
LMO7-AS1	3.659250473	0.681240102	-2.425312901	3.78E-32	4.58E-31
LMNTD2	0.595623692	2.444870214	2.037284837	2.80E-24	1.43E-23
AC020659.1	0.187215248	0.775801102	2.050988882	1.11E-06	1.73E-06
CDKN2B	1.077422874	3.489958453	1.695625263	1.72E-29	1.45E-28
MIR621	16.3406876	7.713759003	-1.082962713	4.29E-31	4.41E-30
LUC7L	2.691615222	5.594674973	1.055582134	3.14E-12	6.92E-12
PTMAP1	0.092679068	0.306989004	1.727871531	3.19E-09	5.85E-09
RPL7AP50	0.435403506	0.949441225	1.124725668	3.15E-14	7.86E-14
LINC01705	0.019412782	0.453374623	4.545624877	3.00E-14	7.49E-14
KLHL31	0.098753783	0.327206487	1.72829343	1.09E-23	5.30E-23
PTGER1	9.081212405	0.265394724	-5.096673324	9.60E-41	2.16E-38
JPH2	0.9676099	3.040657713	1.651885986	5.93E-20	2.18E-19
AHNAK2	0.450615143	7.55171597	4.066836801	1.05E-36	3.80E-35
FADS3	2.141894982	5.340856589	1.3181834	5.28E-29	4.19E-28
DCN	37.8382284	6.456298469	-2.551065363	3.60E-34	6.49E-33

Table S1. The differentially expressed genes(DEGs) were screened out in TCGA

ID	conMean	treatMean	logFC	pValue	FDR
AC005746.2	0.823585772	0.363347983	-1.180567009	5.61E-23	2.59E-22
MYO3A	0.144442468	1.491503332	3.368200321	4.63E-32	5.53E-31
AC079210.1	0.08712425	0.269910272	1.631333641	0.000139362	0.000189874
CYP17A1	13.60738757	3.803805891	-1.838874584	2.61E-32	3.28E-31
NEURL1B	4.733859569	10.52522799	1.15276267	1.57E-18	5.20E-18
BGN	204.7638106	428.3880594	1.064957508	1.51E-11	3.19E-11
AC008735.4	0.226317542	0.572765601	1.339596439	1.99E-14	5.01E-14
RAB39A	0.209429234	0.591218648	1.497228933	3.94E-12	8.63E-12
CDT1	0.380483511	1.822894458	2.260325197	1.37E-35	3.47E-34
GCH1	8.123429958	3.89354356	-1.061005239	4.83E-20	1.79E-19
BPHL	10.42599381	3.653792286	-1.512718382	1.49E-21	6.15E-21
CLEC4F	0.122448114	0.366599785	1.582035392	4.19E-12	9.15E-12
TREX2	0.333426857	1.252323939	1.909165572	1.69E-14	4.29E-14
AC078778.1	0.178513947	0.607967354	1.767957065	4.71E-08	8.02E-08
SOD3	50.24434181	17.101312	-1.554854127	5.04E-32	6.00E-31
CCDC188	0.138070449	0.473324341	1.777424537	2.68E-18	8.74E-18
RN7SL23P	0.080234088	0.325616296	2.020885689	1.92E-11	4.03E-11
NPIPB14P	0.057734765	0.22349632	1.952738882	1.04E-16	3.05E-16
STPG3	0.19322221	0.717862374	1.893446348	4.34E-07	6.95E-07
GPT2	7.45080825	3.420571386	-1.123159596	2.93E-18	9.51E-18
THBS4	0.536691164	2.64445917	2.30080866	0.000172947	0.00023419
DPYSL4	0.159381894	0.739621986	2.214300363	0.001906214	0.002399874
CCDC102B	0.921465085	2.542422905	1.46420262	2.96E-26	1.80E-25
TBX5	0.001234323	0.240517073	7.606275203	2.62E-18	8.53E-18
AC243562.2	0.223181226	0.761618421	1.770852697	1.12E-07	1.86E-07
CHL1-AS2	1.097583107	0.105749349	-3.375609489	5.86E-47	5.24E-44
AL353898.3	0.114663192	0.376761949	1.716250917	2.03E-13	4.82E-13
CCNB2	0.573388583	1.901121406	1.729265581	1.54E-33	2.42E-32
ARHGEF1	4.633667903	10.6500702	1.200636386	2.54E-31	2.70E-30
TBC1D9	20.48664399	9.190858364	-1.156412158	1.57E-32	2.06E-31
FTH1P22	0.09501723	1.461906877	3.943518449	3.13E-32	3.86E-31
HAO2	64.62680813	12.40163215	-2.381602737	3.14E-10	6.10E-10
NFKBID	0.603899051	2.798481684	2.212264994	4.46E-27	2.90E-26
KIF4A	0.26490767	1.223594342	2.207563821	2.27E-34	4.22E-33
TMEM116	4.406298417	1.873106019	-1.234134646	9.00E-33	1.23E-31
AC090286.1	0.142599533	0.346899331	1.282547797	5.22E-09	9.43E-09
MAGI3	5.32290669	1.544040017	-1.785504132	3.38E-37	1.47E-35
RPS15AP24	0.121618251	0.382536158	1.653236378	5.75E-21	2.27E-20
C1S	24.811175	60.26297901	1.280281933	5.43E-08	9.21E-08
LRAT	0.136894504	0.655736692	2.2600521	1.50E-18	4.98E-18
HRASLS2	5.213910625	1.324025005	-1.977435484	6.31E-09	1.14E-08
NR4A2	12.34924099	5.541268422	-1.156134215	3.53E-08	6.07E-08
MIR4639	0.154574097	0.42907156	1.472919701	1.59E-05	2.30E-05
COX7A2P1	4.159563642	0.941825515	-2.142900478	5.03E-27	3.26E-26
TNFSF13B	1.005474456	5.701772058	2.503533935	1.57E-31	1.71E-30
GACAT2	0.076221918	3.076454337	5.334918867	1.11E-31	1.24E-30
LAT2	0.871756264	3.916974376	2.16774296	2.92E-35	6.78E-34
CD22	0.342610763	0.811314312	1.243690464	2.79E-10	5.44E-10
WASF3	5.221801861	1.784134941	-1.549322981	4.05E-37	1.71E-35
CD37	1.638342547	7.275088653	2.150727799	7.53E-33	1.04E-31
TMC8	0.651497248	3.826700659	2.554270062	6.87E-35	1.46E-33
TICRR	0.063202576	0.250010358	1.983932613	2.77E-32	3.44E-31
SYNPO	21.81873318	48.08980586	1.140163762	6.88E-18	2.18E-17
PIPOX	26.87580397	3.573386495	-2.910944044	8.50E-10	1.61E-09
ANG	5.349920968	17.26753471	1.690472639	1.47E-25	8.39E-25

Table S1. The differentially expressed genes(DEGs) were screened out in TCGA

ID	conMean	treatMean	logFC	pValue	FDR
AFAP1L1	3.461961028	9.932583723	1.520579565	5.97E-28	4.22E-27
USP2	21.11981061	5.947915357	-1.828140875	1.87E-27	1.26E-26
TRAV16	0.12231083	0.661238388	2.43461833	4.26E-19	1.47E-18
AC109479.1	0.089551533	0.243883665	1.445403091	1.37E-12	3.08E-12
ANXA13	1.672101258	6.047417996	1.854657086	7.13E-10	1.36E-09
LARGE2	8.575646321	3.275072544	-1.388718544	8.19E-27	5.21E-26
DLG2	0.523576555	0.233507482	-1.164931718	2.88E-30	2.66E-29
GJA4	13.88610468	37.81382938	1.445272005	2.50E-19	8.79E-19
VN1R83P	0.628035346	1.387547249	1.143619239	3.11E-09	5.71E-09
BMP8B	0.629849776	1.481873568	1.234342682	3.49E-09	6.39E-09
SELPLG	3.126343449	13.11657302	2.068842653	9.32E-33	1.27E-31
FN1	32.84256128	113.2576303	1.785969702	6.78E-22	2.88E-21
ADGRG6	3.930049618	8.603998064	1.130459673	5.62E-10	1.08E-09
KLHL17	0.807064456	1.776355957	1.138164903	6.01E-14	1.47E-13
SPINK13	0.370466883	5.803573523	3.96952502	8.20E-25	4.40E-24
LZTS3	14.71820363	2.958505495	-2.31466112	1.26E-38	9.29E-37
INSR	15.25112535	38.0406436	1.318625955	2.49E-25	1.39E-24
AC011476.3	0.779179049	1.565386721	1.006492321	1.24E-05	1.81E-05
AL022322.1	0.196393332	1.376384687	2.809065894	8.38E-24	4.12E-23
PPARGC1A	14.43103276	4.334165749	-1.735348319	4.60E-34	8.05E-33
AC009542.2	0.119693804	1.622238799	3.760565827	5.63E-33	8.00E-32
NPIPA1	0.169593602	0.57378574	1.758430366	6.12E-22	2.61E-21
SYNJ2BP	16.61969164	7.141309614	-1.218633041	8.85E-37	3.34E-35
RNY1P9	0.067170243	0.401303212	2.578798562	9.36E-09	1.67E-08
AC002044.2	0.040551311	0.233044502	2.522785021	6.27E-13	1.44E-12
GPS2	0.650231104	1.854846437	1.512275276	9.15E-18	2.87E-17
AL359510.2	0.083097031	0.241301546	1.53796833	6.73E-12	1.45E-11
PDLIM1	27.92281167	87.90733683	1.654539359	1.24E-33	1.98E-32
AC044840.1	0.034367148	0.36437406	3.40631824	4.00E-13	9.34E-13
MIR4768	1.881322578	6.035138076	1.681639539	1.34E-05	1.95E-05
AL049776.1	0.143580238	0.408791446	1.50950781	4.48E-18	1.44E-17
KRT223P	0.33876326	9.315739501	4.781320975	2.18E-12	4.85E-12
AC008957.3	0.017745285	0.259322862	3.869241773	4.83E-25	2.64E-24
AC090589.1	0.219372157	0.815535509	1.894367269	5.51E-14	1.35E-13
WHAMMP3	0.437482967	0.93055027	1.088857242	6.25E-23	2.87E-22
STX1B	0.202938824	0.712610634	1.812069123	9.25E-22	3.89E-21
AC087623.2	0.582914106	0.276770416	-1.074593572	2.96E-20	1.11E-19
GSAP	1.89271519	4.438525555	1.229623169	3.43E-27	2.25E-26
AC003092.1	0.230722777	0.974585857	2.07862885	3.68E-10	7.12E-10
NOTCH3	14.63377529	37.3008257	1.349905556	1.08E-18	3.62E-18
DHRS11	3.530430692	1.295011505	-1.446879279	2.83E-36	8.65E-35
LAX1	0.220904992	0.937900384	2.086008679	2.19E-21	8.98E-21
AL121748.2	0.133102725	0.309263845	1.21629808	3.14E-12	6.92E-12
NEIL3	0.06360382	0.418741059	2.718873069	6.93E-35	1.47E-33
KLRK1	0.029883865	0.326691405	3.450489826	7.38E-29	5.76E-28
AC087468.1	0.115020676	2.031027506	4.142244648	2.54E-31	2.70E-30
GTSE1	0.177278698	0.887581877	2.323861021	4.68E-34	8.17E-33
EDARADD	0.740378775	0.360155858	-1.039642168	3.43E-25	1.90E-24
ADAMTS10	0.720304188	3.318377704	2.203799909	2.63E-27	1.75E-26
SPHK1	0.60873218	1.930841201	1.665349977	6.24E-19	2.13E-18
AC018755.4	0.460884291	1.392503397	1.595204346	5.28E-19	1.81E-18
LINC02328	0.16953704	0.553991658	1.708263746	9.33E-25	4.98E-24
DNMBP-AS1	0.726244099	0.244411314	-1.571143465	6.75E-34	1.14E-32
AC063961.1	0.194010069	0.652331733	1.749474278	6.14E-05	8.56E-05
AC021683.1	0.163391647	0.428356817	1.390478822	7.82E-08	1.31E-07

Table S1. The differentially expressed genes(DEGs) were screened out in TCGA

ID	conMean	treatMean	logFC	pValue	FDR
ABCA12	0.177234322	0.977853194	2.463959876	2.47E-24	1.27E-23
AC009159.1	0.021385306	0.273619902	3.677481417	1.31E-24	6.95E-24
PLB1	0.253432466	0.639511942	1.335369947	1.04E-23	5.09E-23
IGLC7	0.960335685	6.227441905	2.697028965	7.99E-06	1.18E-05
TNFAIP2	8.306021236	20.42612479	1.298186059	4.89E-21	1.95E-20
MEIS3P1	7.512314403	3.644207986	-1.043652143	2.66E-24	1.36E-23
IGKV4-1	22.3848872	97.57261536	2.123951251	4.39E-06	6.60E-06
RN7SL233P	0.055133876	0.223204743	2.017356758	7.67E-06	1.14E-05
FTH1P20	2.072454469	4.204934208	1.020742821	2.38E-19	8.37E-19
AC079140.3	0.174973861	0.351058709	1.004572899	1.06E-06	1.67E-06
FLI1	2.562854778	6.039956961	1.236786538	1.99E-25	1.12E-24
AGAP2-AS1	1.581780747	5.362099825	1.761248438	4.16E-27	2.71E-26
AC011377.1	0.162166561	0.356517921	1.136498231	0.000299069	0.000398553
LINC02437	8.152251411	0.029143737	-8.127868857	1.22E-50	1.68E-47
AL078604.2	0.034753459	0.355462745	3.354469882	4.46E-29	3.57E-28
MUT	27.66262458	13.38728595	-1.047074535	1.02E-35	2.69E-34
AC127070.4	0.060436082	0.244573509	2.016786108	7.07E-14	1.72E-13
BCL6B	4.773137278	12.16503939	1.349731257	1.69E-18	5.59E-18
GCNT4	3.427825563	1.129813953	-1.601208471	1.28E-30	1.24E-29
U47924.3	0.101494313	0.437019335	2.106298217	6.76E-17	2.00E-16
ATP6V1B1	54.5326197	6.96854969	-2.968189127	7.60E-36	2.07E-34
Z99916.2	0.047990124	0.279260643	2.540802803	4.08E-19	1.41E-18
AC004485.1	2.360905758	0.757422675	-1.640169936	3.20E-08	5.50E-08
JPH4	1.132185136	0.375809416	-1.591036768	6.41E-35	1.37E-33
AC012236.1	0.062926022	0.43360171	2.784641791	2.08E-18	6.84E-18
AC108062.1	0.413451458	1.532030312	1.889654979	4.15E-24	2.09E-23
AP000866.3	0.276587304	0.593024241	1.100356145	1.67E-06	2.57E-06
TRBV5-4	0.135794445	0.662669958	2.286866049	6.29E-18	2.00E-17
CYBA	17.38570603	40.70283459	1.227227613	3.87E-22	1.67E-21
HIST1H2AG	0.205808301	0.707338037	1.781098669	4.09E-08	6.99E-08
N4BP2L1	1.702255244	4.46146328	1.390069588	3.54E-34	6.39E-33
CASP1	2.518213747	8.464012536	1.748941019	2.10E-33	3.21E-32
DYSF	6.811971028	19.42914683	1.512078347	4.99E-28	3.55E-27
NMU	0.1332798	0.995020698	2.9002684	1.86E-09	3.46E-09
STK19B	0.474415655	1.743404859	1.877684113	2.54E-13	5.98E-13
HOXA11	2.285468482	0.805869011	-1.503872662	5.49E-27	3.54E-26
RGS17	0.086348455	0.254016633	1.556680704	1.03E-09	1.94E-09
GRPEL2-AS1	0.060939216	0.483846619	2.989106944	4.29E-25	2.35E-24
AC004854.2	0.314321172	0.764529684	1.282333064	1.98E-22	8.74E-22
AC116036.2	1.1691855	0.381791097	-1.614648476	1.69E-32	2.21E-31
AC103563.7	5.17369644	0.597977582	-3.113032104	1.51E-36	5.13E-35
LINC02391	0.32194975	0.653049696	1.020357253	2.72E-05	3.88E-05
GAPDHP1	3.904729111	12.36488748	1.662954741	1.47E-06	2.28E-06
AC010457.1	0.082443346	0.452768206	2.457297691	1.94E-15	5.24E-15
AL031275.1	0.91051505	0.235134916	-1.953194075	1.13E-22	5.10E-22
AL162578.1	0.025335165	0.237294937	3.22746818	2.48E-06	3.79E-06
TNFRSF11B	30.39567186	13.77588677	-1.141720719	4.21E-21	1.69E-20
CD300A	1.121980594	8.980388604	3.000730152	3.95E-38	2.43E-36
APOD	10.8744237	3.910502721	-1.475512954	8.00E-23	3.64E-22
ITGB2-AS1	0.223036171	1.665270729	2.900407134	2.01E-29	1.67E-28
AC233300.1	0.088627751	0.438261927	2.305962946	7.07E-20	2.59E-19
AC020907.4	0.134377623	1.094545189	3.025966698	1.54E-25	8.78E-25
TMEM245	27.69070431	13.24710836	-1.063724273	1.09E-34	2.19E-33
CLSPN	0.143628893	0.449519532	1.646037806	1.80E-28	1.35E-27
LRRC25	1.648140349	8.083466858	2.294135071	2.61E-32	3.28E-31

Table S1. The differentially expressed genes(DEGs) were screened out in TCGA

ID	conMean	treatMean	logFC	pValue	FDR
AL031429.2	7.9942565	2.014980741	-1.988197813	6.33E-30	5.60E-29
HEPACAM2	11.08079207	5.284465014	-1.068231682	7.08E-36	1.94E-34
MYBL2	0.405437421	2.607001958	2.68484051	3.97E-34	7.08E-33
SIRPB1	0.17449623	1.080339326	2.630216747	1.20E-29	1.03E-28
CCL4L2	1.021498395	6.129015295	2.584968368	9.51E-22	3.99E-21
RNVU1-19	0.05218902	0.220210977	2.077068167	1.17E-05	1.71E-05
SH3GL3	2.022772624	0.039532294	-5.677158668	2.90E-27	1.92E-26
LRRC41	9.500479556	20.7531276	1.127256531	1.44E-17	4.48E-17
B4GALNT3	5.299483011	1.053523503	-2.330629125	1.37E-36	4.74E-35
AC091390.3	0.351979311	1.401581811	1.993493422	8.23E-22	3.48E-21
SNORD1B	0.077854961	0.294573933	1.919768892	4.47E-07	7.16E-07
AC093157.2	0.112826197	0.231611561	1.037605179	3.34E-12	7.34E-12
AC022509.1	0.049703376	1.30890708	4.718875024	6.21E-33	8.73E-32
SORCS2	7.197522139	2.810207982	-1.356823414	7.76E-20	2.83E-19
AC025430.1	0.08078156	0.238864647	1.564095425	2.87E-09	5.28E-09
TEPSIN	0.953810742	2.144401887	1.168800374	1.28E-22	5.75E-22
FOXP3	0.366837174	1.168878911	1.671913732	5.20E-13	1.20E-12
NCCRP1	1.738726015	0.225455437	-2.947116404	2.07E-32	2.65E-31
NCOA7	21.43788489	9.915912845	-1.112345077	3.64E-24	1.85E-23
GFI1	0.164254187	0.947006789	2.527444623	1.98E-30	1.86E-29
HLA-F-AS1	0.33338156	1.13960865	1.773292265	2.12E-39	2.22E-37
IGSF6	0.972910704	6.311128988	2.697518807	5.94E-37	2.35E-35
AL158151.4	0.035789896	0.307705279	3.10392494	1.48E-20	5.67E-20
AC090971.3	0.083883455	0.311712105	1.893756	3.46E-20	1.29E-19
NCAPG	0.215689766	0.934882554	2.115827412	1.97E-34	3.74E-33
HLA-DPA1	33.19723619	121.3011518	1.869458208	1.23E-27	8.40E-27
AC116348.1	0.115886668	0.497746367	2.102696185	0.001806322	0.002278711
MTND4P20	0.08228534	0.300272388	1.867564484	0.012269856	0.014527103
TOX3	6.512128418	1.443172972	-2.173884925	2.21E-34	4.14E-33
SNORD51	0.066855486	0.414425902	2.63199632	6.87E-11	1.39E-10
AC016957.2	0.274332098	0.887658692	1.694081629	9.53E-29	7.36E-28
TRBV23-1	0.018425888	0.311711993	4.080407585	3.26E-16	9.24E-16
PCED1B	1.170480503	3.836522289	1.712698234	5.08E-32	6.04E-31
AL355802.1	0.239009587	0.628675811	1.395247765	1.59E-29	1.34E-28
AC108749.1	1.098309368	0.105363746	-3.381834034	2.39E-16	6.84E-16
GZMA	2.447645085	21.84271956	3.157686208	1.27E-34	2.53E-33
MISP3	8.414979389	2.579467593	-1.705886417	1.23E-32	1.65E-31
CATSPERG	0.168024106	0.528706904	1.653799934	2.81E-15	7.51E-15
MYL4	0.171086951	0.51073126	1.577834637	7.56E-29	5.89E-28
CLIC3	0.534747398	1.644326152	1.620567022	8.36E-16	2.31E-15
CLEC7A	0.744322863	3.934988063	2.402358804	2.90E-31	3.06E-30
MICAL1	2.304040715	6.496947219	1.495595773	1.13E-31	1.26E-30
CD28	0.401188881	1.092552612	1.445349226	1.22E-16	3.56E-16
TDGF1	4.941875346	0.500182846	-3.304531135	2.11E-36	6.72E-35
LINC01983	2.205005878	0.035134937	-5.971732362	1.14E-53	2.14E-50
AC011352.3	0.093314493	3.739439559	5.32457709	5.36E-32	6.35E-31
AC090181.3	0.077182312	0.285442722	1.886859119	1.28E-10	2.55E-10
SBK1	1.449117403	0.531116101	-1.448075311	7.00E-31	6.99E-30
MS4A6A	3.065113829	14.92628491	2.283842572	3.62E-32	4.41E-31
SPSB1	5.927885083	14.26971733	1.267367372	1.50E-15	4.08E-15
AC009119.1	0.103826994	0.343635301	1.726696674	8.64E-28	5.98E-27
SLC2A1	16.14210251	63.5368224	1.976764438	6.96E-30	6.12E-29
TRAV12-3	0.169048066	1.006449563	2.573769456	3.13E-19	1.09E-18
RACK1	119.6126604	273.8791294	1.195169232	1.90E-35	4.63E-34
CHKB	0.561143344	1.851868177	1.722540145	7.36E-20	2.69E-19

Table S1. The differentially expressed genes(DEGs) were screened out in TCGA

ID	conMean	treatMean	logFC	pValue	FDR
LYPD6B	3.542795703	0.472465929	-2.906606074	9.34E-37	3.47E-35
AC021078.1	0.830892818	2.949858335	1.82791138	9.41E-28	6.50E-27
PROSER2	6.066920778	2.732986472	-1.150486154	1.60E-16	4.64E-16
AC015819.2	0.090652528	0.645324545	2.831605749	8.37E-26	4.90E-25
HPDL	0.383607927	0.188960728	-1.021546102	1.89E-14	4.77E-14
RPS5P2	0.10990824	0.27792809	1.338412108	0.003013141	0.003737471
CDC25C	0.070735089	0.448310259	2.663999554	2.16E-34	4.07E-33
MYO9B	4.1783465	9.482977543	1.182407983	4.86E-33	7.00E-32
IGFALS	0.57918144	0.182622971	-1.665147129	2.33E-20	8.81E-20
TOP2A	1.030254579	3.844895731	1.899943598	2.30E-32	2.92E-31
AC034105.1	0.069168885	0.389465566	2.493300669	0.001563501	0.00198308
PFN1P1	0.37696868	0.853329206	1.178657763	3.47E-11	7.16E-11
AC009542.1	0.158357276	0.334794708	1.080093569	2.71E-06	4.13E-06
SNORD36C	0.117936847	0.392459438	1.734529021	1.14E-07	1.90E-07
E2F7	0.079399376	0.336965231	2.085400157	1.85E-30	1.75E-29
AC079209.1	0.030613193	0.228008878	2.896864578	7.99E-29	6.21E-28
CTSW	1.093540138	9.081308296	3.05389398	2.93E-33	4.37E-32
XRCC2	0.140246306	0.435195803	1.633701871	6.26E-30	5.53E-29
CCDC8	13.25770105	5.341038502	-1.311638437	2.80E-18	9.11E-18
PCYT2	9.196652833	3.660777587	-1.328958756	1.14E-30	1.11E-29
ABCC3	4.103354433	20.70616958	2.335185015	1.10E-31	1.23E-30
ACAT1	65.91870276	28.64223753	-1.202543651	4.68E-28	3.34E-27
AL356801.1	0.261990644	0.731487089	1.481317106	2.43E-08	4.21E-08
INHBA-AS1	0.031580925	0.246114993	2.962207218	8.31E-15	2.15E-14
PML	4.127491986	9.57467159	1.21395759	3.33E-36	9.89E-35
EMILIN2	1.160915785	3.687760389	1.6674816	2.18E-30	2.04E-29
TYMS	2.853996469	12.37620179	2.11651317	1.43E-36	4.93E-35
RNU6-322P	0.742568339	1.622344107	1.127484146	3.08E-06	4.67E-06
OAT	33.61081736	14.82165811	-1.181218775	1.11E-36	3.97E-35
PRRT3-AS1	0.35529605	0.745503494	1.069193465	3.26E-09	5.97E-09
FFAR2	0.07535404	0.264411352	1.81102734	1.24E-20	4.80E-20
H1FX-AS1	0.215542041	0.468042793	1.118671152	3.54E-09	6.47E-09
ZNF354B	1.303508158	2.910415271	1.158825406	3.49E-23	1.63E-22
MT-TL1	8.945711522	4.30451976	-1.055343945	1.48E-13	3.53E-13
ERMP1	45.6789176	8.080264609	-2.499054022	1.13E-39	1.36E-37
UPP1	4.363011333	8.786879672	1.010026717	1.52E-15	4.15E-15
CD99	22.64103736	54.36078267	1.263626167	6.39E-31	6.42E-30
LINC00887	0.253411989	10.45881041	5.367090177	4.62E-35	1.03E-33
PSPC1-AS2	0.382395338	0.839438346	1.134359432	1.44E-10	2.87E-10
IL13RA2	2.898230885	1.347450712	-1.104940028	2.07E-08	3.61E-08
LTB4R2	0.168818613	0.802658086	2.249311586	9.46E-34	1.56E-32
RF00409	0.052169991	0.338122772	2.696255105	2.84E-21	1.15E-20
DOC2B	5.248147289	2.168867865	-1.274866048	1.16E-20	4.48E-20
AL391422.1	0.368929315	0.782583958	1.084901108	2.86E-14	7.15E-14
DUSP9	46.69976037	0.385074571	-6.922133483	1.23E-42	6.05E-40
ASS1P1	1.133058591	0.523692861	-1.113429623	0.006889265	0.008323231
AC098613.1	0.255871983	1.128615107	2.141059473	9.83E-30	8.48E-29
GIMAP2	3.195176779	7.97355115	1.319326642	2.02E-25	1.14E-24
NPHS1	13.98247244	0.237648489	-5.878646447	9.17E-16	2.53E-15
TRBV4-2	0.117070998	1.417207799	3.59759569	1.48E-21	6.13E-21
AC011510.1	0.049217096	0.413731336	3.071462784	8.77E-21	3.42E-20
AC007216.1	0.049884767	0.431083861	3.111297319	8.27E-14	2.01E-13
AC025569.1	0.140288037	0.340466168	1.279119455	3.62E-08	6.21E-08
LINC02417	1.58550095	0.1245831	-3.66975846	3.51E-32	4.28E-31
CAMK2N2	0.102393013	0.374546558	1.871027793	2.18E-14	5.49E-14

Table S1. The differentially expressed genes(DEGs) were screened out in TCGA

ID	conMean	treatMean	logFC	pValue	FDR
AP001429.1	0.076358203	0.270341492	1.823927897	1.73E-06	2.67E-06
SCNN1B	28.31457642	1.66903298	-4.08446058	5.36E-40	7.56E-38
AF117829.1	0.30925959	0.721053528	1.221288028	1.23E-18	4.12E-18
PFKFB2	8.036807147	2.444758198	-1.716930683	9.19E-35	1.88E-33
TAS2R19	0.077389849	0.258648084	1.740774264	3.12E-14	7.78E-14
GSDMB	0.363560933	1.345897775	1.888299752	6.04E-18	1.92E-17
AC093797.1	0.130756709	0.436611166	1.739464049	2.20E-17	6.76E-17
AC003989.1	0.015323724	0.250205775	4.029276196	0.006249043	0.007569294
TCEA1P4	0.106796302	0.216720066	1.020971053	3.91E-09	7.12E-09
AL138963.1	0.075702177	0.403033081	2.412491563	6.52E-09	1.17E-08
RPS15AP10	0.225288538	0.587611311	1.38308825	7.20E-11	1.45E-10
LINGO1	1.026240961	4.062510184	1.985001915	2.05E-26	1.26E-25
PIF1	0.066551359	0.327408946	2.298553708	1.86E-31	2.02E-30
AC007405.3	4.075373236	1.980841337	-1.040818865	2.07E-20	7.86E-20
SNORA73B	0.64666156	98.86331895	7.256280675	1.77E-16	5.10E-16
MYORG	9.071014431	3.200948652	-1.502764365	2.64E-36	8.13E-35
KCCAT333	1.232244084	23.47148678	4.251549272	1.15E-15	3.17E-15
CYP4F60P	0.073044896	0.728426155	3.317927346	9.19E-16	2.53E-15
YEATS2	2.785090514	6.017884044	1.111532093	7.86E-31	7.80E-30
CPVL	61.52470986	23.30797139	-1.400342504	1.44E-25	8.27E-25
ZNF726	0.485492023	0.179742871	-1.433513038	3.85E-31	3.98E-30
IGKV2D-30	0.068049783	0.267724693	1.976087753	0.008832521	0.010584014
IGKV1D-42	0.118195626	0.339532339	1.522372348	0.024474073	0.028328116
AP000317.1	0.587630886	0.268794843	-1.12840477	7.97E-12	1.71E-11
C16orf74	0.501583044	3.99209426	2.9925853	6.07E-23	2.80E-22
PCSK1N	22.2394353	7.159968765	-1.635094957	3.86E-26	2.32E-25
GPAT2	0.169482984	0.443956494	1.389277875	5.72E-16	1.60E-15
QRFPR	0.877919125	6.781851231	2.949519189	1.48E-25	8.45E-25
AC145285.3	0.064640032	0.262411413	2.021330652	3.06E-12	6.76E-12
WNT2B	0.377446475	0.95375364	1.337344587	7.32E-06	1.09E-05
SNORA7B	0.092811257	2.937469443	4.984130233	9.67E-10	1.83E-09
AC018695.4	0.120264572	0.430601555	1.840141825	4.80E-16	1.35E-15
RF00003	0.060821144	0.254693346	2.066116407	6.56E-08	1.11E-07
TMEM200B	2.387168067	5.559907593	1.219760764	3.64E-20	1.36E-19
RBP2	2.037824387	0.139397015	-3.869758154	4.08E-36	1.18E-34
AL133230.1	0.080193864	0.317512581	1.985250003	2.12E-13	5.03E-13
PDCD1LG2	1.039202081	2.282631426	1.135221704	7.34E-14	1.79E-13
AC138305.1	0.88356457	0.330300651	-1.419555757	9.71E-32	1.10E-30
PBX4	0.347426898	0.965349606	1.474342065	1.25E-14	3.20E-14
ITGAL	1.221017113	7.198869878	2.559687022	1.35E-33	2.13E-32
ALG1L13P	0.11590607	0.234528635	1.016807962	2.38E-07	3.89E-07
MLC1	0.053651164	0.253570775	2.240707108	2.41E-30	2.24E-29
SH3BP2	4.034804486	13.43978336	1.735939211	1.37E-34	2.68E-33
GCAT	8.686227681	4.068903248	-1.094089793	3.77E-23	1.76E-22
SCIMP	0.48444316	1.939949194	2.001619561	3.13E-28	2.28E-27
HPCAL4	2.760841413	0.177628789	-3.958170691	1.71E-27	1.16E-26
GRK5	1.765715779	4.299069073	1.283771154	7.85E-26	4.60E-25
AC074029.3	0.117005294	0.340218397	1.539887355	3.21E-11	6.63E-11
LINC01738	0.100749715	1.516500645	3.911898453	6.73E-30	5.93E-29
TRAV2	0.119579648	0.705460227	2.56059288	1.16E-19	4.18E-19
KLKB1	0.646815801	1.766200451	1.449222259	3.31E-08	5.70E-08
GALNT6	2.51012825	1.092048044	-1.200724749	5.95E-15	1.55E-14
SNORD67	0.074217632	0.491783777	2.72819026	0.001260938	0.001611033
LILRB5	0.319792113	1.086771141	1.764841898	2.09E-17	6.41E-17
FREM2	5.036462653	1.644065175	-1.615143323	4.60E-34	8.05E-33

Table S1. The differentially expressed genes(DEGs) were screened out in TCGA

ID	conMean	treatMean	logFC	pValue	FDR
AF124730.1	0.101309308	0.270407731	1.416369662	5.73E-07	9.12E-07
NKD2	0.927640203	3.286598973	1.824958179	0.00044277	0.000582736
PLA2G5	0.049894417	0.344077579	2.785783578	4.29E-26	2.57E-25
PDHA1	38.48976486	17.02476874	-1.176839657	1.81E-35	4.42E-34
AC010319.4	0.211860238	0.424270523	1.001871603	1.54E-09	2.88E-09
MAPK10	3.527225085	1.70749035	-1.046656217	5.05E-35	1.11E-33
COL4A2	50.27486917	145.4251536	1.532367501	4.56E-26	2.73E-25
FP671120.1	0.119648011	0.275717745	1.204395712	4.63E-21	1.85E-20
TSPYL2	4.138505625	9.413518347	1.18562412	9.86E-18	3.09E-17
IGLV9-49	0.825740822	24.64058859	4.899203879	1.07E-06	1.67E-06
DSP	27.98280819	10.3091232	-1.440619115	6.60E-32	7.71E-31
SEMA4A	9.113818778	3.051167014	-1.578694532	9.06E-22	3.81E-21
AL034550.1	0.134330037	0.34148936	1.346058696	2.62E-10	5.12E-10
RPL23AP64	0.478601727	1.823033579	1.929443628	6.70E-16	1.86E-15
AC011005.4	0.15805598	0.438513535	1.472185748	1.38E-07	2.28E-07
GRHL2	4.631645056	0.333207364	-3.797032505	1.05E-36	3.80E-35
HMGCR	11.052227	5.441092878	-1.022368739	4.56E-34	8.00E-33
SH2D3C	4.957674847	10.73453102	1.114523603	1.65E-19	5.89E-19
GLYCTK	7.931479182	3.875845214	-1.03307899	4.81E-06	7.21E-06
AC022400.5	0.224676664	0.581952087	1.373050091	2.67E-16	7.60E-16
A4GNT	0.213508376	0.582024418	1.446787012	1.19E-07	1.98E-07
TMSB4XP4	0.536806257	1.19988467	1.160422353	4.99E-14	1.23E-13
AC090286.3	0.526459611	2.305750411	2.130841596	5.14E-11	1.05E-10
FA2H	3.169834805	0.86258338	-1.877671832	3.63E-31	3.77E-30
AL133415.1	0.133377109	0.84456853	2.662703405	1.12E-34	2.24E-33
SNHG1	2.656819181	8.303979614	1.644102859	1.93E-31	2.09E-30
GATM	209.6253164	49.7876089	-2.073954326	4.09E-08	6.99E-08
TMEM156	0.307700508	0.855053315	1.474487554	9.57E-20	3.47E-19
NUSAP1	1.626359465	7.233556383	2.15305896	1.33E-34	2.62E-33
RPL9P29	0.446995	1.052377665	1.235321936	6.84E-11	1.38E-10
CDH23	0.107752377	0.573128409	2.411138717	3.21E-25	1.78E-24
AC005326.1	0.034659342	0.353092764	3.3487311	2.66E-10	5.20E-10
AL354877.1	0.125743814	0.259824393	1.047049454	0.000253829	0.000340024
TRAV26-1	0.078861069	0.468924188	2.571969524	7.88E-21	3.08E-20
CREG2	0.122122549	0.482505367	1.982215384	7.57E-15	1.96E-14
FAM111B	0.264237417	1.205421254	2.189630731	4.76E-34	8.30E-33
C3AR1	3.346978871	12.57674676	1.909827438	5.02E-28	3.58E-27
AP000355.1	0.439309797	1.590784216	1.856427574	1.11E-12	2.51E-12
AL021328.1	0.173607002	2.369806387	3.770872154	3.07E-30	2.83E-29
ANK2	17.19651207	4.063837527	-2.081201347	6.86E-34	1.16E-32
RTEL1-TNFR	0.077287971	0.443098514	2.519311685	1.95E-29	1.62E-28
C1QA	30.67384269	188.6313927	2.620488976	8.09E-34	1.35E-32
RPS19P1	0.150901162	0.36846091	1.287907653	2.43E-11	5.05E-11
AC007566.1	0.641806218	2.09545086	1.707051018	6.93E-18	2.19E-17
RNU6-658P	0.220777793	0.5537892	1.326741854	3.06E-07	4.95E-07
AL590004.3	0.045680603	0.341215207	2.901028337	1.32E-17	4.09E-17
AC131953.1	0.131731295	0.341431349	1.373997408	9.15E-09	1.63E-08
FER1L4	0.059214027	2.789463836	5.557905057	3.06E-39	2.92E-37
CSPG4	2.881595844	19.60701756	2.766430192	1.87E-33	2.90E-32
COLCA1	3.252070764	1.055254527	-1.623767633	7.27E-28	5.08E-27
LCP2	1.60631535	7.624742511	2.246933469	1.79E-36	5.86E-35
MIR23A	0.988055201	2.224557499	1.170854838	0.030131311	0.034584569
AC032044.2	0.116953042	0.55098703	2.236088977	2.69E-10	5.25E-10
CLEC14A	15.4126056	39.53430939	1.358994444	3.04E-18	9.86E-18
DEGS2	6.733143993	0.794410569	-3.083323596	9.17E-30	7.93E-29

Table S1. The differentially expressed genes(DEGs) were screened out in TCGA

ID	conMean	treatMean	logFC	pValue	FDR
LYPLAL1-DT	1.191361415	0.230203997	-2.371626348	7.24E-37	2.78E-35
MT1G	532.7081502	24.31696708	-4.453310148	1.42E-28	1.08E-27
AC091965.4	0.81290199	0.180198654	-2.173493186	1.07E-34	2.16E-33
HLA-S	0.509047074	1.30855167	1.362099913	9.16E-11	1.84E-10
AGR2	4.524751441	0.944386749	-2.260388838	7.35E-35	1.55E-33
AC108704.1	0.200310682	0.912947227	2.188292107	6.19E-29	4.88E-28
FAM198A	0.60730271	0.220096368	-1.464280465	5.36E-29	4.26E-28
AL009178.2	0.554802881	0.242690748	-1.192856171	1.81E-25	1.03E-24
MAP3K15	1.456948697	0.199355109	-2.86953759	1.01E-37	5.30E-36
ALG1L	0.796861857	0.367117573	-1.118087469	5.30E-16	1.48E-15
BMPR1B	6.925853413	1.525887457	-2.182343294	1.89E-36	6.15E-35
C9orf50	0.218483385	0.457371744	1.065843666	0.000190408	0.000257051
MOB1B	13.82833544	6.623388718	-1.06198607	1.81E-33	2.81E-32
TRAV12-2	0.218301273	1.35452236	2.633391757	5.45E-19	1.86E-18
AC002128.2	0.123364493	0.30365243	1.299493695	8.21E-06	1.21E-05
RNU7-75P	0.103852439	0.697939929	2.748567773	8.52E-11	1.72E-10
SLC7A13	7.676463792	0.273152616	-4.812662735	6.87E-33	9.58E-32
RNU7-49P	0.559304349	2.073553924	1.890400113	3.80E-14	9.43E-14
RNU1-14P	2.082746217	11.01866014	2.403389841	6.93E-16	1.93E-15
CD109	4.704063008	10.09567106	1.10175752	2.99E-14	7.46E-14
TNFAIP6	0.569658114	35.32574177	5.954479713	5.81E-38	3.37E-36
DNAH11	0.108037348	2.824726231	4.708509015	4.60E-29	3.68E-28
DOK1	2.091258869	4.974958104	1.250312717	8.21E-32	9.39E-31
RPL21P13	0.027472491	0.326355104	3.570382976	2.58E-16	7.36E-16
KLK6	7.000276228	0.167178771	-5.38794829	6.17E-34	1.06E-32
SLC1A7	0.197012157	0.411091676	1.061175503	0.001355409	0.001727984
TTK	0.203862099	0.599042881	1.555065698	2.70E-30	2.50E-29
ZYG11A	0.604346732	0.26813081	-1.172439502	3.39E-09	6.20E-09
CES3	3.94590223	14.38109304	1.865746218	3.96E-12	8.67E-12
AC007731.2	0.170217514	0.474824141	1.480013796	3.82E-16	1.08E-15
STPG3-AS1	0.204214319	0.67880704	1.732917497	1.09E-05	1.60E-05
AC002563.1	0.199930047	0.442917365	1.147542254	0.032405425	0.037091393
TRABD2A	0.122796511	0.399607113	1.702312697	1.99E-11	4.17E-11
EGFR	10.23830246	28.39874681	1.471850735	8.44E-24	4.15E-23
AC117503.2	0.117871586	0.358500462	1.604758988	1.58E-11	3.33E-11
AFM	13.44497647	0.557824857	-4.591111197	1.59E-14	4.05E-14
RNU6-828P	0.083698907	0.286043048	1.772951592	5.39E-07	8.60E-07
PHKA2-AS1	0.171816153	1.216439595	2.823727101	4.87E-35	1.08E-33
PEBP1	663.8088472	285.8428593	-1.215545607	9.00E-33	1.23E-31
MARVELD3	3.483021264	1.273554928	-1.451478097	2.88E-32	3.57E-31
LINC01559	0.385118022	2.269257528	2.558847803	0.02663308	0.030711429
DNHD1	0.214978321	0.669063306	1.637951544	1.39E-17	4.32E-17
ACSM3	9.623633819	3.672314881	-1.389891982	4.57E-29	3.66E-28
NBPF8	0.260077852	1.284331605	2.304002294	9.71E-34	1.60E-32
AC051619.3	0.048252895	0.670219514	3.795946297	1.06E-25	6.17E-25
SNORD99	0.534826967	6.052408191	3.500365175	2.98E-25	1.66E-24
TAGLN3	2.34926915	0.261931528	-3.16495038	1.35E-37	6.79E-36
AC087741.1	0.311494034	1.167836086	1.906561358	1.35E-13	3.23E-13
RHCG	62.55005826	20.79701356	-1.588634855	1.13E-36	4.04E-35
BAX	8.618243083	20.25855879	1.233065847	9.01E-36	2.42E-34
RNU6-118P	0.226133081	0.891823771	1.979586598	2.02E-10	3.97E-10
LILRA6	0.102931937	0.594055662	2.528907434	4.39E-33	6.37E-32
S100A14	12.84185961	2.839144884	-2.177325754	5.27E-35	1.15E-33
AC104794.4	6.585028931	1.4649556	-2.168332839	3.65E-39	3.31E-37
PHLDA3	6.157462569	18.64748827	1.598573461	8.64E-28	5.98E-27

Table S1. The differentially expressed genes(DEGs) were screened out in TCGA

ID	conMean	treatMean	logFC	pValue	FDR
CCR5	0.782307784	5.954670484	2.92821345	9.31E-32	1.06E-30
CMTM4	27.75082024	7.570558575	-1.874058759	2.03E-37	9.54E-36
AC099336.2	3.086296126	6.826212712	1.145208878	5.18E-10	9.94E-10
FLRT3	16.04999568	7.366223006	-1.12357593	9.41E-24	4.61E-23
FXYD4	287.305672	2.830038334	-6.665621069	1.73E-41	5.32E-39
ACOX2	11.18087994	3.173125389	-1.817057296	5.29E-11	1.08E-10
AC079922.1	0.9746661	3.070877361	1.655670926	7.14E-19	2.42E-18
MAB21L4	5.350334356	0.388980104	-3.781860784	3.23E-33	4.79E-32
RNU2-22P	0.151161561	0.304805151	1.011795965	3.81E-05	5.39E-05
MAPK8IP3	1.665459139	7.235707175	2.119214065	6.31E-27	4.04E-26
AL451007.1	0.329300453	0.662867585	1.00931621	7.40E-16	2.05E-15
AL353572.1	0.142278328	0.300595039	1.079105279	0.003565009	0.004400528
CDH6	7.953147674	27.66590364	1.798511183	1.55E-22	6.91E-22
IFFO1	1.449382218	5.079429094	1.809228254	5.76E-36	1.62E-34
PILRB	0.318904555	1.759513662	2.463980105	4.48E-21	1.79E-20
PLEKHG4	0.560673496	2.442771068	2.123285886	5.79E-28	4.10E-27
ADK	11.93990931	5.263814317	-1.181611375	3.17E-37	1.39E-35
TBC1D24	4.893219097	1.893010339	-1.370101592	2.96E-33	4.40E-32
HEY1	2.223286863	6.654564612	1.581650172	6.65E-26	3.92E-25
ELOVL2	0.148109133	1.400430385	3.24113776	3.88E-31	4.01E-30
UMOD	2746.672191	6.621687491	-8.696270098	1.69E-42	7.90E-40
RNA5SP383	0.359829795	1.768077902	2.296795285	1.70E-16	4.92E-16
AC000067.1	0.051510998	0.495312068	3.265385386	4.78E-29	3.82E-28
GAS8-AS1	0.156197006	0.41205733	1.399478279	3.79E-07	6.10E-07
LINC01359	0.064567435	0.292444003	2.179281787	2.26E-17	6.92E-17
IL2RA	0.334480075	1.620337851	2.276302479	9.76E-19	3.28E-18
NUPR2	7.460512792	1.344576663	-2.472122781	7.44E-38	4.13E-36
KIF20A	0.388391498	1.941276177	2.321421852	4.63E-32	5.53E-31
RHOXF1P1	0.012162792	0.392087547	5.01062945	1.31E-10	2.60E-10
SAMD13	0.569538561	0.205645668	-1.469632845	1.35E-31	1.49E-30
IGHV2-70	1.656294636	12.97103667	2.969262547	0.000891782	0.001150881
AC096992.2	0.557621089	1.149312149	1.043413653	6.90E-24	3.41E-23
AC104564.1	0.114588489	0.340977812	1.573215738	7.54E-10	1.43E-09
AC090204.1	1.961060361	7.559274151	1.94661377	1.59E-09	2.97E-09
AC009506.1	2.726282389	1.231779013	-1.146191551	9.76E-29	7.51E-28
PPM1K	5.446468014	1.921652678	-1.502973353	2.00E-36	6.44E-35
NFKBIE	6.618898931	15.23765777	1.202978012	2.66E-26	1.62E-25
RNU4-22P	0.110118053	0.236174833	1.100804231	0.029879378	0.034308791
AC129510.1	0.363975128	1.039035257	1.513332836	1.12E-11	2.39E-11
AC015722.2	0.451007842	5.78125781	3.680158985	5.29E-09	9.57E-09
SLIT2	4.282229282	1.86475961	-1.199372382	9.51E-14	2.30E-13
AC015912.3	0.244971541	0.58046463	1.244594002	2.26E-07	3.69E-07
TYRO3	3.584852328	0.776616983	-2.206638529	2.73E-09	5.03E-09
CXCR1	0.210486932	0.458931935	1.124549533	6.15E-08	1.04E-07
MYO5C	4.177330732	1.727216898	-1.274132108	7.49E-30	6.53E-29
AC073172.1	5.011077792	0.038402231	-7.027787015	4.22E-51	6.68E-48
RGN	14.52103692	5.564391136	-1.383848737	6.63E-20	2.43E-19
PFN1P2	0.085580625	0.299502024	1.807209641	2.82E-18	9.17E-18
LINC01355	0.246016616	1.126133675	2.194550428	1.08E-20	4.17E-20
AL596223.2	0.062467901	0.262059073	2.068705089	4.44E-08	7.57E-08
GPR34	2.830308182	9.108638451	1.686276266	2.33E-20	8.81E-20
PLK1	0.307266584	1.247110122	2.021026077	1.81E-32	2.35E-31
HIST1H1B	0.009020555	0.494500548	5.776612109	2.30E-05	3.29E-05
TRAV38-1	0.050240006	0.288100635	2.5196643	1.08E-12	2.44E-12
LRRC37B	0.559228093	1.177956812	1.074777904	5.15E-29	4.10E-28

Table S1. The differentially expressed genes(DEGs) were screened out in TCGA

ID	conMean	treatMean	logFC	pValue	FDR
HOGA1	21.11118324	5.717697668	-1.884501198	2.11E-31	2.27E-30
AL355987.4	0.094667293	0.317601892	1.746281539	3.89E-10	7.51E-10
TRBV7-9	0.379669498	2.827487652	2.896704718	1.59E-23	7.65E-23
MNS1	6.072130254	2.916028498	-1.058197919	5.98E-15	1.56E-14
SNORD19	0.299609889	0.770660432	1.363010078	8.77E-07	1.38E-06
AC125807.1	0.166468785	0.334650301	1.007402634	1.39E-08	2.45E-08
AL031577.1	0.085848429	0.313183988	1.867146814	2.16E-12	4.82E-12
LINC01871	0.447713907	2.41537707	2.431599392	1.27E-29	1.09E-28
MIR7152	0.071693903	0.720423248	3.328922402	5.95E-10	1.14E-09
RCSD1	2.568773028	6.211168027	1.273785175	6.99E-25	3.78E-24
KCNJ10	26.30641911	0.373563246	-6.137918551	3.74E-40	5.79E-38
SUCLG1	64.30233056	19.33853684	-1.733392383	1.47E-36	5.04E-35
RPL22P2	0.161800867	0.338646058	1.065558865	0.025906101	0.029915015
NDRG1	92.62849931	239.8847846	1.372813608	1.15E-26	7.21E-26
KCNK7	0.086428862	0.280360638	1.69769875	3.64E-24	1.85E-23
AC008875.1	0.045316393	0.307309455	2.761587203	5.88E-25	3.19E-24
TTC39A	2.354269958	5.278784082	1.164925896	2.28E-15	6.12E-15
AL158196.1	0.078930766	0.236104233	1.580764245	7.08E-11	1.43E-10
BEST4	0.215099418	1.350160675	2.650055576	1.25E-15	3.43E-15
GPRC5A	7.360292208	3.055431543	-1.268386886	1.29E-28	9.81E-28
AL590762.1	0.059879243	0.408805494	2.771286705	9.71E-22	4.07E-21
AL157395.1	0.220131867	0.610592637	1.471842182	3.75E-17	1.13E-16
ADAM19	0.823084474	2.493044472	1.598796211	2.68E-24	1.37E-23
POSTN	7.891605806	23.75144517	1.589624499	1.25E-12	2.82E-12
LINC01704	0.033101467	0.309539735	3.225157545	5.23E-33	7.49E-32
PSORS1C1	0.154431672	1.203787813	2.962540547	1.27E-36	4.46E-35
PDK1	1.321960119	5.058250405	1.935959804	7.51E-35	1.57E-33
MMP7	110.6706324	54.39604269	-1.024698835	5.09E-11	1.04E-10
AC008667.1	0.047834265	0.261689384	2.451739078	4.44E-17	1.33E-16
AC084876.1	0.0829491	0.572314339	2.786509521	1.30E-24	6.85E-24
CDC37L1-DT	1.998020522	0.957912555	-1.060605533	6.12E-25	3.32E-24
ASLP1	0.411354244	0.951687987	1.210107333	1.89E-06	2.91E-06
LINC02244	0.258086935	0.961574116	1.897540949	3.37E-15	8.95E-15
CTSH	60.76937139	25.10300574	-1.275484251	2.99E-35	6.94E-34
DDX41	12.31289335	25.13412427	1.02947761	1.72E-33	2.68E-32
PRSS51	0.113934046	0.459849657	2.012963347	6.53E-17	1.93E-16
AL589739.1	0.094179395	0.244754711	1.377853262	3.71E-05	5.24E-05
LDHAP5	0.194425847	0.500979823	1.365532475	9.80E-18	3.07E-17
PFKFB4	0.961428575	5.080213919	2.40163766	2.61E-34	4.79E-33
ADH6	9.490228603	1.618544615	-2.551745706	1.09E-09	2.05E-09
RPS20P33	0.221810671	0.582086805	1.39190554	4.32E-13	1.01E-12
LINC00863	1.045556932	0.501965108	-1.058612629	5.35E-28	3.80E-27
IPO13	15.2066731	7.386499087	-1.041741906	3.13E-35	7.19E-34
TRBV20-1	1.141252884	3.140158488	1.46021887	4.18E-22	1.80E-21
USP30-AS1	0.394299421	2.072622248	2.394093699	1.15E-28	8.81E-28
UNC5B-AS1	0.11012297	0.404775825	1.878007707	1.01E-06	1.59E-06
GRIN2D	0.101210758	0.388634528	1.941051442	2.35E-25	1.32E-24
FOXJ1	2.582773893	0.28889677	-3.160295369	3.67E-35	8.29E-34
PSORS1C3	1.595966915	15.21931104	3.253400401	2.98E-29	2.44E-28
AL136368.1	0.213745951	0.550508281	1.364867176	1.79E-21	7.37E-21
LCAT	1.370049468	4.805940794	1.81059089	4.20E-23	1.96E-22
TYROBP	15.28945163	96.42195363	2.656824997	1.62E-36	5.42E-35
TRAF2	2.88729175	5.931370671	1.03864864	3.19E-33	4.74E-32
AC130469.1	0.057492006	0.584919001	3.346803578	2.14E-19	7.57E-19
INO80C	2.136129792	4.341637814	1.023240071	1.19E-25	6.85E-25

Table S1. The differentially expressed genes(DEGs) were screened out in TCGA

ID	conMean	treatMean	logFC	pValue	FDR
HAPLN2	0.132875689	0.303311861	1.190724745	3.53E-08	6.07E-08
GEMIN8P4	1.14873664	0.567605258	-1.017088223	9.76E-29	7.51E-28
PLA2G1B	0.283904363	0.64208825	1.177366578	2.04E-05	2.93E-05
AL353751.1	2.176800043	0.978551064	-1.153489848	4.62E-19	1.59E-18
IGLV4-69	4.600324245	36.05767816	2.970499046	0.000373714	0.000494411
IL1RL1	8.227516492	2.075214184	-1.987196768	1.62E-22	7.19E-22
SUSD4	10.24236959	0.79297523	-3.691129911	7.50E-39	5.87E-37
PRR4	0.196089791	0.422960476	1.109008428	3.15E-15	8.39E-15
COL4A4	11.2928144	2.604077625	-2.116560721	1.76E-39	1.92E-37
LINC01508	1.247707283	3.608484867	1.532113692	1.81E-14	4.57E-14
AC011498.7	0.145778768	0.595105995	2.02936604	2.74E-23	1.29E-22
PHYH	37.20635557	16.68414104	-1.157071669	1.16E-19	4.17E-19
SLC5A4	0.295514144	1.068480623	1.854261656	5.39E-19	1.84E-18
IGKV2D-29	1.152520884	6.769730069	2.554305417	6.51E-06	9.69E-06
TRBV28	1.116786232	8.664480758	2.955760234	2.11E-30	1.98E-29
AC022973.4	0.086001746	0.244464802	1.507188901	1.02E-11	2.17E-11
NPIPB3	0.098969769	0.44901974	2.181719052	2.44E-24	1.26E-23
ITK	0.260597012	1.165764617	2.161384071	3.29E-26	1.99E-25
PRDX3P1	0.172835894	0.363300777	1.071761584	3.25E-06	4.94E-06
KCNK9	0.046907578	1.113727643	4.569431666	1.55E-28	1.17E-27
DDX39A	5.899424569	11.85008443	1.006251192	3.43E-31	3.58E-30
SLN	0.322082094	1.848364372	2.520748826	0.018950631	0.022116411
VGLL1	5.806579646	0.073854644	-6.296856142	3.31E-46	2.27E-43
RDH10	25.37267486	7.968975777	-1.670809403	2.05E-36	6.57E-35
RNU6-50P	0.098265905	0.454446756	2.209348429	1.99E-12	4.43E-12
CYBB	5.948485569	21.40530795	1.847374268	1.71E-20	6.53E-20
IGHV4-61	0.880962227	3.013112678	1.774102557	0.001377879	0.001755214
IGHA1	110.5818066	382.7663462	1.791349943	1.13E-08	2.00E-08
NETO2	1.020722718	10.1677074	3.316331506	3.05E-38	1.95E-36
AP006623.1	0.196932677	0.93836278	2.252443273	3.04E-20	1.14E-19
TUBBP5	0.814199362	0.275494317	-1.563359534	4.31E-12	9.41E-12
ZHX3	14.31925232	5.88670429	-1.282424101	1.67E-34	3.22E-33
AC005682.1	0.192191314	1.129398828	2.55494	2.91E-32	3.60E-31
AC092597.1	0.512674085	0.246553744	-1.056139811	3.22E-17	9.75E-17
RELB	4.108267917	8.908311619	1.116621757	1.70E-26	1.05E-25
TNNT1	0.231251107	1.199889323	2.375369161	5.48E-15	1.44E-14
TMEM246-AS	3.836072087	1.068066228	-1.844628723	6.19E-12	1.34E-11
DTX2P1-UPK	0.082123084	0.289376934	1.81709021	2.98E-26	1.81E-25
CHRNA6	0.030612507	0.255630475	3.061866717	4.13E-25	2.27E-24
EPS8L3	0.134585474	1.491126405	3.469807954	0.000160301	0.000217424
RN7SL124P	0.003968255	0.406173429	6.677447317	1.01E-25	5.84E-25
AC130371.2	4.471458525	0.744195501	-2.58699192	4.69E-36	1.34E-34
SMIM10L2B	3.039244736	1.042249146	-1.544012663	4.00E-34	7.13E-33
ACTG1P17	0.078803524	0.232229241	1.559217585	1.26E-17	3.92E-17
OVOL1	3.546528335	0.689473556	-2.362840349	1.18E-36	4.21E-35
SVEP1	3.704366904	1.228410829	-1.592433864	1.30E-22	5.83E-22
CCDC181	2.103362587	0.261561039	-3.007478008	2.66E-39	2.66E-37
TRBV12-3	0.085789612	0.472627531	2.461828806	6.04E-17	1.79E-16
PSD2	0.051766686	0.282810553	2.449740076	4.20E-23	1.96E-22
AC092296.1	0.692633397	0.195727952	-1.823242149	2.21E-07	3.61E-07
SLC22A8	36.29032022	0.563868794	-6.008081474	2.37E-19	8.35E-19
AC069185.1	0.485633755	0.178681834	-1.442475733	1.26E-22	5.63E-22
COL9A2	6.876993875	1.50120491	-2.195657147	6.29E-34	1.07E-32
3-Mar	0.313182803	0.865184127	1.466002202	4.06E-30	3.68E-29
TRPC2	0.063280854	0.912948132	3.850691929	4.18E-17	1.26E-16

Table S1. The differentially expressed genes(DEGs) were screened out in TCGA

ID	conMean	treatMean	logFC	pValue	FDR
LINC01127	1.073906914	4.993360994	2.217142263	0.000195861	0.000264194
MIR4653	0.590973042	1.59432913	1.431785261	1.27E-12	2.86E-12
LDLRAD3	1.786375321	4.943504661	1.468498967	5.78E-31	5.84E-30
AL021407.1	0.029023622	0.230397698	2.98882682	3.62E-18	1.17E-17
NKD1	0.944592378	0.129040163	-2.871871726	1.68E-36	5.56E-35
AC243732.1	0.063514948	0.265728701	2.064785979	3.86E-24	1.95E-23
SH3GL2	6.929802973	0.996888801	-2.797309843	8.95E-34	1.48E-32
MIR4645	0.633917863	2.005794383	1.661805895	2.10E-10	4.13E-10
IGLV8-61	4.347945808	20.09799394	2.208645644	2.13E-05	3.06E-05
MIR8083	0.059227161	0.381834337	2.688616003	3.49E-06	5.28E-06
AC046185.3	0.46498034	1.00762808	1.115721608	1.76E-20	6.75E-20
CLDN10-AS1	1.514802259	0.280193245	-2.434635396	4.78E-33	6.89E-32
AC092813.2	3.771409921	0.115020279	-5.035143815	1.24E-17	3.86E-17
GAPDHP2	0.075626262	0.228777572	1.596986417	6.65E-24	3.30E-23
AC005831.1	0.239123886	1.071510317	2.163815589	2.13E-31	2.29E-30
PLCB4	2.049854081	4.345284259	1.083929343	4.37E-06	6.57E-06
HCST	2.506624654	9.894549697	1.98088807	1.59E-27	1.08E-26
IRF9	0.563746139	1.906369183	1.757709983	2.67E-19	9.36E-19
AP001992.1	0.188532858	0.812645846	2.107810774	9.76E-19	3.28E-18
AC011481.2	0.173586929	0.497653643	1.519483684	5.88E-23	2.71E-22
SAMD12	3.19535639	0.881558896	-1.857847987	1.26E-36	4.42E-35
RAB42	0.50168346	14.45848814	4.84899552	2.97E-37	1.32E-35
AC002091.2	0.206906069	0.703430353	1.765431625	1.35E-20	5.20E-20
CEACAM1	9.381274944	4.287132586	-1.129770968	4.59E-25	2.51E-24
IRF4	0.192470378	0.781507344	2.021623002	8.01E-17	2.36E-16
AC017104.1	0.252635922	0.657047316	1.378937477	2.51E-23	1.19E-22
ADAMTS18	0.13645773	0.386673367	1.502661276	8.30E-13	1.90E-12
AC087645.2	0.129727861	0.355541245	1.454528579	8.90E-19	3.00E-18
TRAV23DV6	0.112269687	0.428993963	1.933988895	3.94E-15	1.04E-14
C2CD4C	0.275868908	0.754172187	1.450911079	1.24E-13	2.99E-13
AC122710.2	0.024552503	0.224256705	3.191209129	3.67E-28	2.66E-27
RAET1E	0.890929207	0.255746995	-1.800593513	2.61E-16	7.43E-16
AC009495.2	0.078292324	0.441091062	2.494133757	1.51E-16	4.38E-16
AC026401.3	1.351287322	3.591197765	1.410130638	1.23E-32	1.65E-31
CDCA3	0.23019797	0.736696491	1.678195258	1.47E-29	1.24E-28
C21orf62	5.731995479	1.247830136	-2.199615915	1.63E-31	1.78E-30
AC005006.1	0.066921196	0.373100215	2.479028058	3.27E-16	9.27E-16
PTPRR	0.13341116	0.400996282	1.587709511	1.73E-11	3.63E-11
BNIP3P26	0.060690061	0.226976171	1.903008666	7.10E-10	1.35E-09
PARP6	3.098685861	6.226735974	1.006819603	1.78E-25	1.01E-24
AL157871.3	0.114066558	0.39996468	1.809996714	3.28E-07	5.30E-07
AC127070.1	0.550970137	0.220776136	-1.319389892	3.87E-22	1.67E-21
PI16	2.674226382	0.563014786	-2.247876883	1.82E-21	7.51E-21
RF00100	2.072775651	240.0493575	6.855623291	0.000175916	0.000238132
DDX11	0.904536835	2.189761946	1.275522881	7.04E-21	2.76E-20
OCLN	5.049051958	1.205095843	-2.066864634	1.05E-35	2.76E-34
LUCAT1	0.03662434	1.489655172	5.34603183	3.36E-36	9.95E-35
AF127936.1	0.1192279	0.604294188	2.341529197	1.02E-22	4.59E-22
AC105446.1	0.233053782	1.497607159	2.683924406	1.19E-22	5.33E-22
MIR210HG	1.204069344	11.55013316	3.261919097	8.61E-37	3.26E-35
CHST13	1.531017755	6.216933418	2.021712114	5.29E-22	2.26E-21
DNAJC5B	0.038939513	0.519577129	3.738031172	3.25E-34	5.87E-33
AC006128.1	0.341817386	1.677452632	2.294974343	4.86E-19	1.67E-18
AL357078.3	0.043064464	0.264383725	2.618063582	2.08E-17	6.38E-17
ESRRG	9.957923889	1.152864747	-3.110621721	5.61E-41	1.41E-38

Table S1. The differentially expressed genes(DEGs) were screened out in TCGA

ID	conMean	treatMean	logFC	pValue	FDR
AC137932.3	0.086061301	0.38460449	2.159939047	8.46E-22	3.57E-21
TRBV24-1	0.099810743	0.523539388	2.391031074	6.28E-19	2.14E-18
NDUFS1	20.33792392	9.020242729	-1.172934256	8.93E-36	2.40E-34
RPL39P38	0.237425852	0.798983069	1.750687901	1.29E-16	3.75E-16
CIART	1.53947355	3.938089617	1.355058862	6.16E-22	2.62E-21
HCLS1	4.375098833	16.71255883	1.933545126	7.17E-34	1.21E-32
MIR324	0.104733558	0.788007903	2.911486331	1.85E-17	5.70E-17
AC008569.2	0.056332537	0.251062942	2.156008736	1.51E-19	5.38E-19
U62317.2	1.890257183	6.762733048	1.839023869	1.62E-28	1.22E-27
SLC9A3	57.94358244	5.313743508	-3.446848388	1.73E-34	3.32E-33
RNF139-AS1	0.128615294	0.258603535	1.00767979	7.27E-06	1.08E-05
ITGA10	0.509532919	1.152932879	1.178061263	1.65E-08	2.90E-08
IL11	1.442833319	0.134805611	-3.419952193	6.57E-30	5.79E-29
CPA6	0.079085163	0.451020925	2.511715403	1.16E-28	8.84E-28
TTYH2	0.93637589	2.067042399	1.142408288	1.34E-19	4.81E-19
MAPT-AS1	0.118242614	0.266138322	1.1704262	0.003325584	0.004112633
IGHV1-69	1.61452826	9.229171318	2.515088421	7.28E-05	0.000101045
AC009095.1	0.06693367	0.223331941	1.73838558	2.95E-17	8.99E-17
AL359697.1	0.174767724	0.398858149	1.190436978	5.82E-06	8.68E-06
MYZAP	2.186091749	0.767622731	-1.509884612	1.30E-33	2.07E-32
TFCP2L1	38.44710229	4.12019332	-3.222090933	2.26E-37	1.05E-35
TLR8	0.398471467	1.794289803	2.170864597	2.55E-23	1.21E-22
NBL1	5.508348625	11.66995813	1.083107609	9.26E-06	1.37E-05
TRBV11-2	0.139708356	0.863098204	2.627106406	5.48E-19	1.88E-18
TNFRSF4	0.570296188	5.561031829	3.285569301	6.99E-38	3.89E-36
SH2D1B	0.235735878	0.56113457	1.251175452	1.94E-17	5.96E-17
MIR378A	0.217944184	0.541482223	1.31295527	5.03E-05	7.06E-05
MIR4668	0.251692397	1.156256614	2.199728074	2.18E-12	4.85E-12
AL683807.1	0.02567042	0.591022164	4.52503342	4.31E-34	7.60E-33
TMEM100	0.880249875	2.144307105	1.284526519	0.006120712	0.007416469
NRL	1.118936979	0.486626098	-1.201243182	2.59E-36	8.02E-35
AL683842.1	0.043379087	0.432960587	3.319164122	4.89E-25	2.67E-24
GIPC3	2.011482443	4.90397621	1.285692835	1.78E-19	6.31E-19
ARC	4.00972511	0.939595361	-2.09339184	2.28E-10	4.46E-10
CAVIN3	6.530876361	31.40651909	2.265715551	4.31E-33	6.27E-32
AC096642.1	0.071196627	0.310014922	2.122456852	6.13E-20	2.25E-19
PA2G4P4	0.069492349	0.266114641	1.937121832	1.83E-24	9.55E-24
AC092809.2	0.144747899	0.353254512	1.287165581	4.43E-10	8.53E-10
APBA2	0.235244325	0.551278252	1.228620766	1.00E-06	1.57E-06
MTCL1	0.662110686	3.213721341	2.279100521	2.72E-32	3.39E-31
MCOLN3	1.259686615	0.441798555	-1.511604258	7.80E-28	5.44E-27
EVL	2.491277997	8.103930572	1.701735796	2.16E-37	1.01E-35
AC084048.1	0.016598668	0.254649187	3.939371775	0.031486334	0.036077507
SEC61G	14.13858258	30.23772851	1.096712272	2.45E-36	7.63E-35
HIST1H4H	3.257064269	7.366496292	1.177406409	2.63E-08	4.55E-08
AC005387.1	0.085842017	0.450948958	2.393208269	1.84E-18	6.05E-18
LRRN2	6.521923876	1.189875536	-2.454486931	7.09E-33	9.85E-32
AC027796.1	0.080155765	0.230650849	1.524832417	1.59E-05	2.30E-05
DHRS9	0.14599854	0.553026016	1.921393407	1.57E-24	8.24E-24
VNN2	1.094486039	3.860287269	1.81845466	5.05E-19	1.73E-18
AC010175.1	0.247627247	0.507440309	1.035068062	6.23E-08	1.05E-07
CCER2	0.072077471	0.453540799	2.653612032	6.15E-16	1.72E-15
IGKV2-30	0.783076669	1.979691728	1.338050325	0.000794108	0.001028116
LINC01976	1.911128415	0.059512246	-5.005094333	6.44E-51	9.47E-48
AL138693.1	0.318990567	0.982916411	1.623554972	2.65E-15	7.09E-15

Table S1. The differentially expressed genes(DEGs) were screened out in TCGA

ID	conMean	treatMean	logFC	pValue	FDR
NDC80	0.315854479	1.294332188	2.034875996	6.24E-34	1.06E-32
MARCO	0.513380503	2.577398728	2.327815329	7.27E-11	1.47E-10
CPA4	0.193966124	1.446292202	2.898482442	0.004411008	0.005403243
AC254562.1	0.213898801	0.540813872	1.338203763	3.87E-06	5.84E-06
KIF13B	15.28129669	6.018791947	-1.344221116	5.47E-37	2.21E-35
MYL3	9.070032205	3.906237016	-1.215328184	4.40E-16	1.24E-15
AC007546.1	0.176552719	0.591982441	1.745455342	1.03E-11	2.19E-11
AC015819.1	0.225566245	0.766739407	1.765185141	2.52E-29	2.07E-28
AL731569.1	0.145928478	0.371474343	1.34800112	3.27E-10	6.35E-10
SLC14A2	12.24156341	0.132961566	-6.524634734	3.45E-29	2.80E-28
CADM3	0.819236038	4.931648471	2.589718882	1.85E-11	3.88E-11
RNU1-16P	0.080123852	0.244955529	1.612216165	3.18E-05	4.52E-05
STMN3	7.105869833	31.17910834	2.133496504	1.23E-29	1.05E-28
AC025171.5	0.151054002	0.450048807	1.575017061	1.02E-20	3.96E-20
AL662797.1	0.164724244	0.387608723	1.234548128	2.08E-20	7.91E-20
AC007216.2	0.066103292	0.231464753	1.807998498	4.26E-09	7.74E-09
RALGPS1	2.674809842	0.69178597	-1.951038672	1.14E-35	2.94E-34
AC090907.1	0.225473657	0.495996357	1.137370638	3.64E-11	7.50E-11
AC011461.1	0.062127226	0.288901464	2.217279974	4.07E-13	9.49E-13
TRAV20	0.092756593	0.475867866	2.359039294	9.24E-14	2.24E-13
CD180	0.324302843	1.435832085	2.146473465	9.05E-28	6.26E-27
AC005740.3	0.06267081	0.284944327	2.184814534	1.00E-14	2.58E-14
MTHFD2P1	0.08121303	0.571417784	2.814762823	1.24E-06	1.93E-06
TEX29	0.098517918	0.351736245	1.836035965	5.25E-13	1.22E-12
AC023421.1	10.3351166	0.109043989	-6.566500613	2.25E-34	4.19E-33
UBAP1L	0.154287469	0.735236811	2.252588108	1.05E-24	5.59E-24
BAZ1A	2.120259778	4.877858391	1.202006839	4.45E-30	4.00E-29
RN7SL368P	0.070209149	0.414291435	2.560915054	2.75E-12	6.09E-12
AP002907.1	0.159508453	0.54597421	1.775199927	6.88E-15	1.79E-14
AP000424.2	0.94571865	0.399804344	-1.242116902	4.88E-15	1.28E-14
SNORD73B	0.179871367	0.485860528	1.433576685	0.000255873	0.000342651
AP1M2	36.51333944	6.079679493	-2.586356446	8.52E-40	1.10E-37
RNU7-159P	1.965865924	0.043700314	-5.491377477	6.61E-47	5.67E-44
HIST1H3D	0.489141903	1.501251259	1.617840489	2.84E-07	4.60E-07
AC107021.2	0.347370031	2.442888311	2.81404271	7.27E-36	1.99E-34
PERM1	0.265677277	2.254864083	3.085293726	6.74E-19	2.29E-18
SMTNL2	15.94501939	7.027830481	-1.181954555	7.79E-26	4.57E-25
MIR146B	0.053660121	0.442588382	3.044043372	1.28E-08	2.26E-08
AL022328.2	0.640800372	2.063638931	1.687243678	2.97E-17	9.04E-17
APOO	22.04685331	9.295915636	-1.245903877	1.49E-34	2.90E-33
AC016027.5	0.061779282	0.303070683	2.294459302	4.64E-11	9.47E-11
AC005387.2	0.076183328	0.48807851	2.679566005	2.08E-23	9.95E-23
RN7SL209P	0.041212979	0.298990371	2.858928373	8.14E-21	3.18E-20
LINC01615	0.062430013	0.827937884	3.729210855	5.52E-33	7.87E-32
AC091057.1	0.079306576	0.297423205	1.907004817	4.39E-33	6.37E-32
PPP1R1A	50.03813301	7.079538523	-2.821300735	8.85E-32	1.01E-30
CATSPER1	0.062505563	0.25256355	2.014589944	9.23E-29	7.14E-28
LAT	0.025849011	0.275403271	3.413364728	3.03E-29	2.48E-28
KCNIP2	0.178899345	0.613359249	1.777584213	1.18E-21	4.91E-21
AC091180.4	0.047962231	0.223186734	2.218280597	3.70E-13	8.64E-13
PARD6A	6.492252083	2.632609051	-1.302225725	9.30E-29	7.19E-28
NTN4	90.44181	28.77264223	-1.652292153	2.06E-32	2.63E-31
ERVMER34-1	4.557869131	0.177435501	-4.682992905	2.02E-40	3.58E-38
PDZRN4	0.600891836	0.223037683	-1.429817844	2.04E-26	1.25E-25
AC018413.1	0.679717436	0.33943727	-1.00179015	3.74E-29	3.02E-28

Table S1. The differentially expressed genes(DEGs) were screened out in TCGA

ID	conMean	treatMean	logFC	pValue	FDR
AC068792.1	0.226520147	0.576236966	1.347022843	5.95E-21	2.35E-20
SLC2A5	5.262929739	18.04480087	1.777645183	4.95E-17	1.48E-16
AC025917.1	0.206882977	0.615589517	1.573153723	6.52E-14	1.59E-13
APOM	38.40334376	16.86765048	-1.186972899	0.00079868	0.001033839
BARX2	2.129813957	13.56773695	2.671380785	9.90E-31	9.73E-30
AC245128.3	0.143240538	0.307066672	1.100112093	5.84E-07	9.28E-07
SORD	13.67269904	3.156407983	-2.114942466	1.93E-24	1.00E-23
ARL4D	33.24288156	4.751545976	-2.806576549	3.10E-36	9.31E-35
AP000445.1	0.554602745	2.255348534	2.023823737	2.15E-18	7.04E-18
AC010359.1	1.354879143	0.515147598	-1.395106416	2.55E-28	1.88E-27
ABHD17C	9.568681139	2.25346936	-2.086172256	4.45E-38	2.70E-36
LINC01503	9.189198282	3.606180712	-1.349467304	1.32E-26	8.24E-26
AC015660.3	0.089215582	0.267705957	1.585281636	6.92E-08	1.17E-07
VCAM1	29.0877245	103.6318576	1.832985227	8.23E-22	3.48E-21
PLXNA3	1.441355619	3.711254294	1.364480528	2.58E-29	2.12E-28
TAGAP	0.799857706	3.178739016	1.990639299	2.03E-29	1.68E-28
RNY1P16	0.205965311	0.639770391	1.635152849	3.02E-05	4.29E-05
AC078883.1	0.372244354	1.589411205	2.094170548	6.83E-39	5.41E-37
CEMIP2	5.911299542	13.94990272	1.238707829	1.98E-29	1.65E-28
AP001330.5	0.886319618	1.778865362	1.005058368	3.56E-21	1.43E-20
CP	5.26941831	67.03234319	3.669141749	1.15E-24	6.10E-24
PAPPA2	7.125206577	0.665734173	-3.419913712	2.84E-35	6.62E-34
IGKV2OR22-4	0.197062208	0.914330154	2.214064075	0.003315848	0.004101333
KDM4A-AS1	0.134700932	0.367634373	1.448511834	1.92E-15	5.18E-15
CHST1	1.472567306	3.989618213	1.437917118	1.68E-06	2.60E-06
TSPAN32	0.154131487	0.642675757	2.059929439	1.82E-30	1.72E-29
AC008781.3	0.107344848	0.414423765	1.948853791	3.07E-16	8.71E-16
NEURL3	2.956253514	1.455666243	-1.022090383	1.30E-09	2.44E-09
CNTNAP1	0.773412464	2.399863273	1.633642295	2.10E-25	1.18E-24
CCDC74A	1.416309654	3.969114231	1.486680361	1.12E-22	5.06E-22
HOXB7	23.16635154	11.35267813	-1.028998178	4.35E-25	2.39E-24
CATIP-AS2	1.148341592	0.378829991	-1.599929402	4.61E-30	4.15E-29
FAM151A	57.19443873	3.926809124	-3.864445497	6.41E-20	2.35E-19
AC116407.1	0.612525028	0.223748986	-1.452887647	3.85E-27	2.51E-26
PPP2R2B	0.950009132	0.19622742	-2.275414726	5.89E-39	4.85E-37
SOST	8.345863233	0.077795032	-6.745239445	9.48E-33	1.29E-31
HSD11B2	206.4807	27.00716632	-2.934592757	4.49E-38	2.70E-36
AC012181.2	0.51326245	1.485844042	1.533514072	1.45E-15	3.94E-15
UQCRFS1	37.26782847	15.40110064	-1.274897301	1.48E-35	3.71E-34
RN7SL431P	0.059403953	0.300717317	2.339777113	5.81E-13	1.34E-12
FDFT1	23.19094181	11.39448641	-1.025225511	1.13E-37	5.83E-36
CXCR4	13.70918847	94.82623863	2.790143139	4.33E-39	3.76E-37
ADAM11	0.054528636	0.238834573	2.130925718	1.57E-25	8.94E-25
RNA5SP217	0.180299276	0.564463376	1.646486371	4.35E-09	7.90E-09
CICP14	1.007750621	2.508695231	1.315798544	7.46E-14	1.82E-13
SDR42E1	1.390640193	0.575634417	-1.272524435	8.28E-32	9.45E-31
CDCA7	0.261865057	1.069732386	2.030354458	5.11E-29	4.06E-28
TGFBR3	15.92464507	5.356685893	-1.571848612	1.99E-34	3.77E-33
GLIPR2	3.714377196	7.536702188	1.020813057	5.09E-21	2.02E-20
HS3ST2	0.088621338	2.57976382	4.863441071	6.44E-37	2.51E-35
MFSD6L	2.58066242	0.221457737	-3.542638129	5.62E-37	2.26E-35
GJC2	0.616661364	1.579862504	1.357248641	2.30E-11	4.80E-11
SERPINB9P1	0.63220218	2.841475856	2.168182542	1.36E-21	5.64E-21
CFD	5.613680972	12.94402018	1.20526678	1.78E-13	4.23E-13
TPTEP1	1.90681709	0.39160884	-2.283681222	2.13E-33	3.26E-32

Table S1. The differentially expressed genes(DEGs) were screened out in TCGA

ID	conMean	treatMean	logFC	pValue	FDR
VENTX	0.17365028	0.678147674	1.965414731	6.10E-24	3.04E-23
RPL26P27	0.080144208	0.254086094	1.664647256	3.29E-10	6.38E-10
PEBP1P2	0.582720751	0.231082755	-1.334395087	2.26E-20	8.53E-20
RPL35P2	0.284118974	0.623069527	1.132897977	6.40E-19	2.18E-18
CYP27B1	5.799186388	0.492092382	-3.558849421	2.10E-19	7.44E-19
IPO5P1	4.101562917	1.94219448	-1.078486088	4.60E-29	3.68E-28
IL18R1	1.058179985	2.621489478	1.308801721	1.73E-20	6.62E-20
CHGB	7.505851206	0.56755816	-3.725175547	2.27E-39	2.34E-37
TNS4	1.063752366	0.205528647	-2.371750942	2.48E-34	4.57E-33
SLC52A3	7.860796167	0.926636243	-3.084600425	2.39E-40	3.97E-38
QPRT	39.26529278	17.63692326	-1.154655748	7.63E-22	3.23E-21
DNA2	0.293911138	0.835864797	1.50788957	2.94E-21	1.19E-20
AL158071.3	0.089309709	0.305014679	1.771989745	1.43E-15	3.90E-15
AC010519.1	0.056486838	0.413089523	2.87046782	7.44E-24	3.67E-23
RNF152	20.80206882	9.55677838	-1.122130747	1.09E-16	3.18E-16
CD33	0.283466928	1.113944592	1.974425142	1.34E-30	1.29E-29
AL031123.2	0.85046066	0.229017389	-1.892787359	2.88E-32	3.57E-31
COL15A1	8.31187869	22.55457991	1.440173912	1.39E-17	4.32E-17
AL391840.1	0.147756938	0.372471734	1.333905076	0.006337139	0.007673295
NPM1P26	0.126342944	0.27644854	1.129665858	0.000587863	0.00076744
AL133330.1	0.043470985	0.241123775	2.471649203	8.02E-15	2.08E-14
AC124017.1	4.182311133	0.042685007	-6.614427172	1.43E-41	4.45E-39
HNRNPA1P2	0.109964533	0.664512247	2.595257507	5.03E-26	3.00E-25
HS6ST1P1	1.514442026	0.269082608	-2.4926653	4.18E-21	1.67E-20
FBLN1	16.56879004	5.448985631	-1.60440866	2.38E-25	1.33E-24
ZDHHC11B	0.327275326	1.039894523	1.667860457	1.96E-07	3.22E-07
SLC39A14	15.33448104	51.30762502	1.742393903	3.30E-27	2.17E-26
FRMD1	1.282117849	0.338503478	-1.921286314	1.01E-12	2.30E-12
AC087500.2	0.323473991	0.681875334	1.07585828	1.74E-09	3.25E-09
AL096799.1	0.226582897	5.78237663	4.67355171	4.33E-31	4.45E-30
FBP1	122.671715	25.50755822	-2.265805934	4.77E-26	2.85E-25
ENHO	0.703398515	0.232181354	-1.59909017	1.33E-23	6.45E-23
ZDHHC2	18.55405651	5.227870612	-1.827439301	2.89E-36	8.76E-35
MPC1	84.37310833	27.62483053	-1.610817638	2.53E-33	3.82E-32
TRIB1	25.07911036	12.34636964	-1.022399281	2.69E-21	1.09E-20
RNU6-26P	0.254321776	0.685112199	1.429685276	4.03E-06	6.07E-06
FBXL8	0.709039438	2.693148207	1.925355846	6.47E-36	1.80E-34
AL139246.2	0.044376466	0.36833398	3.053147798	2.11E-21	8.66E-21
AC138207.6	0.05887014	0.241793042	2.038164763	1.79E-30	1.70E-29
LRRC37A15P	0.077409195	0.265325007	1.777183799	1.12E-19	4.05E-19
CCDC178	1.152512514	0.418963368	-1.459886402	8.06E-30	6.99E-29
EVC	22.58294536	9.979299643	-1.178223186	1.97E-23	9.46E-23
AL109659.2	0.08474623	0.3250274	1.939340252	1.99E-19	7.05E-19
AJAP1	2.044710165	0.921870133	-1.149260924	4.06E-19	1.40E-18
AC110285.7	0.103919824	0.293813742	1.499430984	2.22E-22	9.76E-22
IGHV1-14	0.073585689	0.357964556	2.282319629	0.000966239	0.001244319
SLC44A4	29.69985932	8.055449093	-1.882419171	3.52E-31	3.66E-30
CARMIL1	9.38288075	3.42424646	-1.454244386	5.31E-40	7.56E-38
HOXB-AS3	5.586956483	0.940967277	-2.569846125	6.02E-36	1.69E-34
PLAU	72.7867979	15.84399196	-2.199740923	1.85E-25	1.05E-24
AL645939.4	0.05451231	0.512490354	3.232870882	1.20E-21	4.98E-21
NR0B2	12.87589489	1.486644849	-3.114540763	9.70E-38	5.16E-36
AC073410.1	0.081814345	0.283543684	1.793145292	3.09E-12	6.81E-12
COL1A2	25.30513908	83.16353519	1.716520683	4.66E-16	1.31E-15
FMNL1	1.03265516	4.96354021	2.265010912	3.33E-35	7.61E-34

Table S1. The differentially expressed genes(DEGs) were screened out in TCGA

ID	conMean	treatMean	logFC	pValue	FDR
WAS	1.768378444	8.080171018	2.191958774	4.20E-33	6.12E-32
RNU6-44P	0.070063296	0.682692545	3.284505238	4.67E-09	8.46E-09
MIR587	0.501192033	1.544078726	1.623310924	0.016673419	0.019524079
AL162724.1	0.024456312	0.472565953	4.272236917	2.37E-28	1.75E-27
RNU6-1016P	0.840441776	3.055479543	1.86217904	1.30E-12	2.93E-12
RNF182	1.334912956	0.560443124	-1.252105797	2.81E-22	1.23E-21
MIR4258	0.237018122	0.565134745	1.25359752	3.32E-11	6.85E-11
AC068580.3	0.830549708	1.848468753	1.154192235	4.64E-16	1.30E-15
AL121574.1	0.604693601	0.187214729	-1.691510371	1.01E-23	4.96E-23
AP001273.1	0.281378972	0.619303285	1.138131584	5.07E-17	1.52E-16
AC024267.3	0.145109344	0.390901423	1.429664411	1.08E-10	2.16E-10
ITGA2B	0.165621092	0.410019869	1.307807413	0.04173843	0.047373659
RTP2	0.006550877	0.258093904	5.300064099	5.99E-28	4.24E-27
BRCA2	0.130868212	0.439986619	1.74934494	2.63E-32	3.30E-31
AC026780.2	0.060739253	0.312210993	2.361820254	3.96E-07	6.36E-07
AC020916.1	3.358283921	12.75118897	1.924835665	9.40E-13	2.14E-12
SLC16A1-AS1	0.065275	0.610393011	3.225135987	4.00E-40	6.14E-38
NSUN5P1	0.627663686	2.109712935	1.748983059	4.72E-15	1.24E-14
PLSCR3	0.094858615	0.289108285	1.607759237	1.77E-27	1.19E-26
ADAMTS14	0.066169847	0.675236999	3.35114811	1.87E-30	1.77E-29
NDUFA4L2	13.10351732	580.0550561	5.468163815	6.20E-38	3.55E-36
SCN4A	0.29201623	0.951316624	1.703877033	1.59E-08	2.78E-08
VSIR	7.326332611	17.01410665	1.215568298	4.72E-29	3.77E-28
PCDH10	0.836712994	2.740933863	1.711862772	0.006529787	0.007899128
EGFLAM	0.925767089	2.621092148	1.501446894	3.73E-16	1.06E-15
RNU6-94P	0.205818528	0.724806525	1.816223084	1.70E-09	3.17E-09
AC008555.1	0.381394983	0.944955228	1.30896011	3.70E-10	7.16E-10
TOX2	0.931117296	3.140214458	1.753828265	3.74E-26	2.26E-25
TRIM55	3.609656213	7.424757365	1.040482441	7.29E-12	1.57E-11
DNAJB5-DT	0.079374046	0.278804003	1.812512018	6.24E-12	1.35E-11
AL158163.2	0.13578472	0.388466221	1.516468012	8.84E-20	3.21E-19
NFKB2	7.662133431	17.83473026	1.218871342	3.30E-27	2.17E-26
MDM4	1.999776215	4.018825008	1.006935195	5.42E-14	1.33E-13
AC008760.2	0.19677516	6.251811183	4.989654192	3.73E-35	8.40E-34
TRAV3	0.116137831	0.748555373	2.688271044	5.58E-19	1.91E-18
ARAP3	3.677318542	7.357245327	1.000511546	1.07E-15	2.95E-15
PYGL	3.956743111	16.08835989	2.023631952	1.28E-35	3.25E-34
AC024941.2	0.628825404	0.281462583	-1.159716358	4.22E-21	1.69E-20
CYP39A1	3.964899986	1.254855272	-1.659763493	1.12E-31	1.25E-30
SLC17A3	19.7777427	67.07067627	1.761804373	1.26E-09	2.36E-09
IGFL2-AS1	0.021451053	0.82743995	5.269534234	2.01E-07	3.30E-07
RN7SL809P	0.165499822	0.548611778	1.728955928	8.95E-11	1.80E-10
SPATA18	3.055894821	10.28690348	1.75114198	3.70E-27	2.43E-26
DPP9-AS1	0.027380636	0.253833116	3.212652415	3.88E-32	4.70E-31
AC005261.3	1.385184403	3.062944949	1.144841389	7.04E-29	5.52E-28
NMNAT2	0.24856373	0.783576293	1.656457955	0.000557802	0.000729284
PCP4	17.94134801	3.034350321	-2.563828729	3.64E-36	1.07E-34
PMCH	0.011450176	0.323610897	4.820818461	5.80E-27	3.73E-26
ETS1	20.11911801	45.90594895	1.190114063	3.37E-21	1.36E-20
SELE	3.96314371	1.125186636	-1.81648096	1.93E-14	4.88E-14
SGO1	0.132327331	0.272646243	1.042919205	5.13E-20	1.89E-19
TRAV4	0.155301446	1.013204457	2.705782163	1.48E-21	6.13E-21
AC002553.2	0.371951738	1.035501346	1.477142084	8.72E-14	2.11E-13
AC005840.1	0.252539177	0.538529978	1.092519442	1.07E-10	2.13E-10
C1RL-AS1	1.378691882	4.238105339	1.620119375	1.84E-21	7.57E-21

Table S1. The differentially expressed genes(DEGs) were screened out in TCGA

ID	conMean	treatMean	logFC	pValue	FDR
ANKZF1	2.868032944	8.053115346	1.489485401	2.64E-29	2.17E-28
OR51E1	0.423612902	2.478632567	2.548725986	2.08E-31	2.24E-30
RNA5SP498	0.109179076	1.070315845	3.29326829	1.10E-17	3.45E-17
PTTG1	0.751440964	3.737554144	2.314362809	2.23E-34	4.17E-33
AC079015.1	0.069813338	0.732073011	3.390412942	1.14E-33	1.84E-32
KNG1	288.1662501	2.998896111	-6.586326023	1.30E-41	4.16E-39
AC018410.2	0.078971944	0.347141635	2.136112301	6.72E-18	2.13E-17
TRAV22	0.118260256	0.360959194	1.60987044	1.05E-07	1.74E-07
HLA-DQB1	14.35841368	71.73125957	2.320705595	4.98E-30	4.46E-29
PHKA2	3.605396111	16.81318925	2.221363735	1.61E-35	3.99E-34
ALKBH6	0.208363367	0.68840122	1.724148003	3.06E-28	2.23E-27
IFI44L	2.052316403	5.665500426	1.464950227	7.19E-21	2.82E-20
SPINK5	0.21438913	0.944449288	2.139241573	1.19E-07	1.97E-07
FAM182B	0.099039882	0.236244759	1.254200828	1.43E-16	4.16E-16
AL096855.1	0.15891962	0.421685338	1.407869613	4.29E-12	9.37E-12
ALDH1A3	7.05628835	2.501162224	-1.496310884	1.87E-05	2.70E-05
METTL26	13.5204699	39.33778121	1.540770294	1.25E-31	1.39E-30
AL590648.3	0.154676573	0.409487874	1.404566019	1.24E-20	4.78E-20
PPP1R18	6.110836583	19.19803279	1.651516681	1.73E-33	2.70E-32
AC004836.1	0.1572689	0.663780891	2.077473693	1.10E-17	3.43E-17
MFHAS1	8.761978153	4.139312407	-1.08186548	2.33E-30	2.17E-29
FBXO17	8.267283444	24.7774875	1.583544646	4.33E-32	5.19E-31
TPT1P5	0.19946955	0.909946695	2.189613506	4.45E-20	1.65E-19
TIAF1	0.253710764	0.598590365	1.238384328	1.21E-14	3.08E-14
AC064799.1	0.463211335	0.98471745	1.088039268	3.90E-12	8.55E-12
KCNAB3	0.140194601	0.379040695	1.434921955	1.63E-08	2.86E-08
TOB2P1	0.322198734	0.870966898	1.434667066	2.69E-21	1.09E-20
IFNAR2	2.667835708	5.754906838	1.109122749	2.94E-29	2.40E-28
AP000864.1	0.032410171	0.701472455	4.435867925	1.17E-30	1.14E-29
AL162430.1	0.083907732	0.253796347	1.596795646	3.30E-22	1.44E-21
PARP10	8.04703	16.35611095	1.023301438	6.75E-26	3.97E-25
RN7SKP80	0.170387993	1.158571771	2.765451841	3.31E-12	7.28E-12
LSP1	2.140109313	10.15848706	2.246929158	4.37E-32	5.23E-31
AC138470.1	0.095035199	0.269020731	1.501183493	3.82E-14	9.48E-14
ATP2B2	0.16075763	1.697238912	3.40023054	9.06E-26	5.28E-25
TRBV18	0.158751505	0.818320916	2.365896461	1.42E-18	4.73E-18
NPIPB15	0.394023681	1.349177929	1.77572638	2.18E-05	3.12E-05
ACADSB	31.33014843	8.133489794	-1.945605205	2.12E-35	5.09E-34
LINC00460	0.030224813	0.994363976	5.03996869	1.74E-19	6.19E-19
ECHS1	227.5192168	91.84253814	-1.308753986	2.96E-25	1.64E-24
GLYAT	53.81034066	20.01598803	-1.426730608	1.37E-05	2.00E-05
TNIP3	0.033819218	0.436745959	3.690879135	2.59E-30	2.40E-29
AC127496.5	0.089695119	0.233194547	1.37843267	6.43E-17	1.91E-16
AC009955.4	0.138058858	0.373451086	1.435635842	3.20E-07	5.17E-07
LINC00106	0.595310683	2.735853403	2.200276237	1.03E-11	2.20E-11
LIPG	2.432585054	0.981768137	-1.309035998	2.55E-23	1.21E-22
LGI2	6.551814993	0.661253716	-3.308618789	1.01E-34	2.05E-33
EFHD1	97.58812811	15.57314109	-2.647645686	2.18E-40	3.80E-38
ATP5MFP5	0.050273701	0.30215737	2.587424336	0.016047863	0.018810829
PCDH1	19.62311604	9.759049995	-1.007741533	6.88E-28	4.82E-27
RN7SL381P	0.266330975	1.19799645	2.169331502	1.34E-07	2.21E-07
IGHV3-43	1.233913283	4.604851501	1.899913624	8.90E-05	0.000122652
AC068790.4	0.142136757	0.464043894	1.706981591	4.53E-12	9.87E-12
SLC22A13	4.29512116	0.62710593	-2.775917762	2.87E-14	7.17E-14
AC093249.3	0.216815468	0.450601146	1.0553833	0.002781249	0.003457751

Table S1. The differentially expressed genes(DEGs) were screened out in TCGA

ID	conMean	treatMean	logFC	pValue	FDR
AC022532.1	0.064638127	0.310948939	2.266220401	2.43E-26	1.49E-25
GCM1	0.656977023	0.173973177	-1.916978024	6.21E-26	3.67E-25
TAPBP	27.74980472	84.23953798	1.602019905	2.87E-37	1.28E-35
ISCA1P4	1.175166085	0.464325266	-1.339656975	4.40E-16	1.24E-15
AC006369.1	0.068966135	0.404364626	2.551696777	1.10E-26	6.91E-26
AC087379.2	7.33464733	1.378487024	-2.411641912	6.60E-26	3.90E-25
Z84480.1	0.063204588	0.237496424	1.909804602	1.84E-15	4.97E-15
TUBAP2	0.109639026	0.248636169	1.181274766	1.40E-18	4.66E-18
FCGR1CP	0.101521983	0.786443502	2.953550974	1.79E-32	2.33E-31
LINC01428	0.190741621	2.727427881	3.837849459	1.82E-28	1.36E-27
AC005845.1	0.088022943	0.232265065	1.399820654	7.47E-16	2.07E-15
PLPP4	3.884623211	0.630034783	-2.624271286	8.00E-37	3.05E-35
TPSB2	1.704389531	5.54143091	1.701003463	2.88E-17	8.78E-17
LINC01186	1.943661324	0.836731606	-1.215940019	5.75E-07	9.14E-07
HAUS7	0.456368492	1.733783817	1.925652927	1.16E-19	4.19E-19
APOL1	25.99648115	107.0530704	2.041937914	5.05E-25	2.76E-24
AL033381.1	0.167476934	0.460496965	1.459229231	0.025628088	0.029607266
ALOX5AP	3.809420556	8.71263291	1.193537191	2.67E-15	7.15E-15
AL033519.1	0.015430386	0.287601568	4.220225486	4.86E-11	9.92E-11
AC007969.1	3.174462546	7.739096314	1.285652758	2.37E-15	6.36E-15
RF00190	0.12979396	0.725270573	2.482296068	1.21E-19	4.33E-19
TIMP1	73.32822694	291.5273354	1.991190605	5.51E-26	3.27E-25
AC120114.3	0.170486944	0.894307293	2.391109377	4.29E-26	2.57E-25
DGKA	0.815325156	1.795447383	1.138895941	7.12E-20	2.61E-19
NGFR	2.313789089	8.467775684	1.871725689	9.76E-19	3.28E-18
SLC12A1	255.6958626	0.835677082	-8.257267531	4.99E-42	2.03E-39
AC105339.5	0.060303246	0.238036236	1.98087364	1.32E-12	2.98E-12
TRGV4	0.064596539	0.307302827	2.25013227	1.03E-20	3.98E-20
MC1R	0.264908213	0.799431296	1.59348148	5.41E-20	1.99E-19
PF4V1	0.720211486	2.991160317	2.054212723	8.05E-10	1.53E-09
C1orf162	2.351763361	11.95347687	2.345615505	2.70E-34	4.94E-33
PDIA5	5.542858264	18.6799957	1.752792101	5.34E-32	6.34E-31
ARID5A	3.583940444	9.507311987	1.40749084	7.47E-27	4.76E-26
SCGB1D2	7.228524489	2.573808947	-1.489796223	7.06E-13	1.62E-12
VASH1	2.009656497	9.254050724	2.203136079	1.43E-35	3.60E-34
CCDC68	0.859401415	1.734885942	1.01343676	3.29E-09	6.02E-09
PRDM1	1.253641832	6.253365249	2.318507559	2.00E-36	6.44E-35
AL590666.2	0.486509766	3.37347872	2.793696391	3.37E-31	3.52E-30
VPS9D1-AS1	0.229626455	0.75562897	1.718389153	5.67E-11	1.15E-10
NCR3	0.203123655	0.730691176	1.846903528	4.46E-26	2.67E-25
FAM171A2	0.981014417	0.461319659	-1.088507564	1.28E-13	3.07E-13
KLF7-IT1	0.193726489	0.493576922	1.349253711	3.54E-05	5.00E-05
NIPA2P1	0.003460519	0.345481324	6.641475592	5.94E-30	5.26E-29
FLT1	11.79856785	45.57086422	1.94949998	7.10E-25	3.83E-24
COL1A1	16.98836258	114.9473076	2.758353964	2.10E-23	1.01E-22
AL391422.4	1.363021236	3.392335316	1.315470741	3.98E-29	3.21E-28
AC020558.2	0.191130927	0.566317682	1.567050336	9.48E-12	2.03E-11
ADAMTS2	1.123038863	4.229426687	1.913054262	2.35E-25	1.32E-24
AC103810.3	0.566058615	2.473615909	2.127598146	2.42E-16	6.91E-16
TRAV21	0.168999613	1.258429278	2.896532294	1.19E-21	4.95E-21
POLE2	0.295709241	0.760690455	1.363130173	2.61E-31	2.76E-30
MIR31HG	0.81821282	0.312817315	-1.387155772	2.45E-17	7.50E-17
EPHB3	2.862107953	1.33623825	-1.098900827	9.20E-12	1.97E-11
AC119674.1	0.106076697	0.295813789	1.479581547	4.28E-19	1.48E-18
CDH4	0.253601562	4.342345418	4.097838956	2.22E-18	7.28E-18

Table S1. The differentially expressed genes(DEGs) were screened out in TCGA

ID	conMean	treatMean	logFC	pValue	FDR
IGLV3-19	14.18982428	123.4008551	3.120423763	7.61E-07	1.20E-06
APLN	6.064477011	33.99312623	2.486787907	5.53E-33	7.87E-32
NRBP2	3.968318847	11.25848348	1.504412656	2.68E-24	1.37E-23
DOK2	1.319769275	4.914704685	1.896818994	1.54E-29	1.30E-28
UCN	0.219488887	1.32043169	2.588789867	5.39E-33	7.70E-32
NKAIN1	0.087297619	0.902048153	3.369190245	5.32E-31	5.40E-30
AL135999.1	0.211246046	0.893068678	2.079846786	7.64E-17	2.25E-16
SLC7A11	0.114650648	0.388249872	1.759740939	1.52E-25	8.69E-25
SLC25A33	4.438026464	1.33797974	-1.729862	3.22E-38	2.05E-36
AL671277.2	0.466013775	1.742432827	1.902658535	9.77E-27	6.17E-26
AC092017.1	0.341971666	0.733584126	1.101085623	1.77E-08	3.09E-08
AL445222.1	0.2085552	0.730726383	1.808902016	3.59E-29	2.90E-28
AC002550.2	0.412610028	0.988443296	1.260379321	1.49E-18	4.95E-18
AP001351.1	0.070871625	0.25333622	1.837773328	3.33E-25	1.85E-24
AL355877.1	1.902548204	0.437114804	-2.121848862	1.20E-37	6.14E-36
ANKRD9	4.677594694	2.007749958	-1.220187251	4.45E-30	4.00E-29
MEGF11	0.410981206	0.861106794	1.067119748	1.29E-07	2.13E-07
CCL11	2.321754935	0.411467152	-2.496366534	4.10E-28	2.95E-27
AC003072.1	1.326956728	3.043336402	1.197532489	1.00E-18	3.36E-18
APOBEC3F	1.222887569	2.648046756	1.114636825	3.68E-30	3.36E-29
AC068051.1	0.054396318	0.348146862	2.67811512	5.73E-15	1.50E-14
ACSL6	0.78189695	0.181689791	-2.10550112	9.22E-34	1.53E-32
LOX	5.912242657	63.54692831	3.426045002	3.63E-31	3.77E-30
GMDS	6.360934319	3.142846245	-1.017166996	8.07E-32	9.26E-31
MCF2L	1.440241615	3.336591008	1.212063996	7.36E-20	2.69E-19
PODXL	118.0175738	48.69259505	-1.277227409	4.80E-13	1.12E-12
AC099568.2	1.935018079	0.578785479	-1.741246413	1.59E-28	1.19E-27
RAPGEF3	7.533435167	2.285044075	-1.721085876	1.67E-36	5.54E-35
PIP5K1B	3.304663321	1.041505637	-1.665832649	6.40E-33	8.98E-32
IFITM9P	0.122924958	0.422544545	1.781325575	1.59E-09	2.97E-09
APBB1IP	2.817833142	15.2176924	2.433091516	4.33E-32	5.19E-31
GATA3	25.44814668	2.867507597	-3.149691379	4.57E-39	3.94E-37
AC105910.1	0.009541414	0.271886951	4.832660035	1.42E-14	3.63E-14
DENND1B	2.978026556	1.436179221	-1.052120824	2.20E-30	2.06E-29
RNU6-341P	0.1373297	0.655321074	2.25455826	3.26E-06	4.94E-06
AL031709.1	0.140392904	0.540365565	1.944465719	5.01E-15	1.32E-14
SNORA70F	0.994048033	0.396280515	-1.326793534	3.80E-19	1.32E-18
AC243962.1	0.028647656	0.321506163	3.488357402	1.26E-30	1.22E-29
AC084036.1	1.959146381	3.954125661	1.013133526	7.57E-09	1.36E-08
AFF2	0.107304963	0.248794359	1.213236974	7.83E-15	2.03E-14
LOXL1-AS1	2.236166771	1.11315472	-1.006373656	3.97E-21	1.59E-20
HMGN5	5.817919583	2.718307554	-1.097794661	2.14E-36	6.82E-35
AC007993.2	2.418381238	0.043957725	-5.781781148	1.79E-48	1.76E-45
AC004890.2	0.180973627	0.613974307	1.762398815	2.84E-10	5.53E-10
AC114744.1	0.103398094	0.268332497	1.375812199	1.07E-12	2.42E-12
AL591806.2	0.10056994	0.363560618	1.853996777	3.09E-08	5.33E-08
CLDN16	29.83053597	0.535628503	-5.799413355	1.22E-41	4.10E-39
COL21A1	0.430954178	2.071087579	2.264782176	1.12E-18	3.75E-18
AC020978.3	0.139303573	0.588476248	2.078751925	2.66E-16	7.58E-16
AP000695.1	0.582864603	1.556323916	1.416909664	1.64E-12	3.67E-12
MMP25-AS1	0.31537941	1.680744971	2.413940454	8.09E-36	2.19E-34
SORCS3	0.355196651	3.352937965	3.238735906	0.004895646	0.005976374
TMEM200A	3.30866263	8.516555809	1.364021913	2.17E-19	7.67E-19
AC009078.3	0.097151716	0.250753243	1.367956969	3.76E-09	6.86E-09
PXDN	4.119729611	13.12571666	1.671774637	7.10E-25	3.83E-24

Table S1. The differentially expressed genes(DEGs) were screened out in TCGA

ID	conMean	treatMean	logFC	pValue	FDR
LCN2	8.599858558	3.208513648	-1.422407812	8.73E-21	3.41E-20
AL662844.3	0.115057083	0.989206459	3.103921859	2.20E-28	1.63E-27
ANGPTL2	9.848470819	25.9743834	1.399117863	6.80E-18	2.15E-17
ZNF37CP	0.181999683	2.785514859	3.935936167	4.04E-31	4.17E-30
KIAA1211	0.580439959	0.246338163	-1.236506694	3.57E-20	1.33E-19
SLC6A13	18.22763463	39.93324295	1.13146288	3.75E-07	6.04E-07
ANK3	7.225486125	2.985830338	-1.274962464	9.90E-31	9.73E-30
AC016831.4	0.019025655	0.36144352	4.247752196	3.09E-06	4.69E-06
LOXL4	8.232749111	3.631325794	-1.18087789	4.37E-11	8.94E-11
AC022144.1	1.099055503	5.979006996	2.443641654	1.59E-28	1.19E-27
AL049840.1	0.645291486	1.845984376	1.516367448	2.55E-12	5.66E-12
LGR6	0.632048805	0.210063396	-1.589211175	7.28E-19	2.47E-18
RNU1-47P	0.086700914	0.819308348	3.240287401	2.82E-19	9.84E-19
AC018521.5	1.243988728	0.579176101	-1.102899435	2.72E-32	3.39E-31
CALCA	8.634989161	1.194736171	-2.853502298	2.69E-38	1.76E-36
FAM43B	1.165653957	0.373494866	-1.641979246	1.72E-21	7.09E-21
INSYN1	2.05563104	0.839966485	-1.291177672	3.60E-31	3.74E-30
AL031727.1	1.033221788	2.818616319	1.447837135	6.27E-18	1.99E-17
NCKAP1L	1.048964601	5.351423428	2.350956693	7.42E-32	8.58E-31
MIR6797	0.123608046	0.75477466	2.610273334	7.58E-17	2.24E-16
IGHG1	45.43358152	363.3513878	2.999534474	4.06E-07	6.52E-07
SLC20A2	12.20490826	4.655861438	-1.390341423	1.44E-38	1.01E-36
TM4SF19	0.02969557	0.285459178	3.264964797	4.11E-24	2.07E-23
COL5A3	0.538646386	6.329459402	3.554671905	7.68E-40	1.01E-37
DDC	63.70149566	20.87503853	-1.609548386	1.80E-05	2.60E-05
ITGA4	1.272222754	4.459601974	1.809563658	5.48E-25	2.98E-24
AC011611.3	0.03887101	0.221581098	2.511068308	2.43E-14	6.11E-14
BDH1	3.506545168	0.941491531	-1.897030289	5.52E-34	9.52E-33
RNU6-35P	0.6739363	0.240979897	-1.483699438	1.33E-20	5.13E-20
ENTPD3-AS1	2.166550347	0.990115086	-1.129731632	5.77E-33	8.16E-32
LINC01480	0.101911092	0.678030913	2.734039975	1.08E-30	1.05E-29
ADSSL1	2.430510576	13.95030413	2.520965258	4.48E-21	1.79E-20
TLCD1	6.521000056	2.605691959	-1.323426691	3.95E-29	3.18E-28
AC016769.3	0.031979412	0.604939504	4.24157554	1.48E-18	4.93E-18
AC008060.1	0.00221274	0.27821923	6.974244306	2.17E-12	4.84E-12
STOX1	1.882333121	0.567703606	-1.729312156	6.23E-31	6.26E-30
DDB2	2.728486625	11.14791891	2.030601536	1.72E-40	3.24E-38
LINC01693	0.057528593	0.23535881	2.032510761	8.80E-09	1.57E-08
AP000941.1	0.154207844	0.396861335	1.363758862	2.63E-09	4.85E-09
SORT1	37.95197806	13.23821091	-1.51946692	9.04E-39	6.89E-37
GLDC	16.88547622	3.381029429	-2.320248389	2.37E-36	7.41E-35
EMX1	14.03607081	2.292337375	-2.614247837	9.41E-41	2.16E-38
TUBB6	7.763664708	17.16056881	1.144287656	9.14E-24	4.48E-23
CRP	0.039230023	4.083626221	6.701748816	5.53E-10	1.06E-09
UPP2	9.156492904	0.480300644	-4.252785479	7.74E-29	6.03E-28
HLA-B	290.8432708	1113.972107	1.937399279	1.20E-39	1.42E-37
DPEP1	84.10286769	4.305901897	-4.287767638	3.23E-23	1.52E-22
PRDX4	19.73624175	52.36921108	1.407871579	2.61E-36	8.08E-35
C1orf54	4.916049013	10.35624053	1.074929174	2.10E-23	1.01E-22
AC010999.1	0.081502844	0.255947326	1.650924632	2.02E-13	4.78E-13
RN7SL138P	0.738028744	3.747057668	2.344009269	1.82E-36	5.95E-35
CCDC142	0.730609724	1.585522314	1.117785322	1.73E-28	1.30E-27
AP000344.1	1.008316956	0.21050915	-2.259994364	4.99E-18	1.59E-17
AL022238.1	0.039723908	0.223010223	2.489030391	3.48E-10	6.75E-10
VKORC1	13.38587731	29.04751399	1.117703001	5.19E-30	4.63E-29

Table S1. The differentially expressed genes(DEGs) were screened out in TCGA

ID	conMean	treatMean	logFC	pValue	FDR
AL161935.3	0.235629934	0.933232814	1.985714208	7.29E-29	5.70E-28
CD5	0.624199294	2.728489097	2.128023648	3.32E-26	2.01E-25
PLEKHS1	0.458023357	0.210195094	-1.12369217	4.97E-08	8.45E-08
NOS1	1.580688498	0.08064948	-4.292744047	1.54E-29	1.30E-28
RPS20P15	0.026550539	0.821642444	4.951697653	0.000101674	0.00013971
PPM1F	3.499926667	7.31964063	1.064448125	2.33E-27	1.55E-26
SNORD83A	0.189347022	0.870061088	2.200083965	2.08E-12	4.64E-12
EXOC3L4	0.918692661	4.06288031	2.144848655	1.48E-24	7.79E-24
AL118508.4	0.116447827	0.29619961	1.346886023	1.66E-14	4.22E-14
HLA-H	17.05623982	57.10324799	1.743273179	2.33E-32	2.96E-31
AC011458.2	0.085787909	0.230567063	1.426340197	8.14E-08	1.37E-07
GPR35	0.293800333	1.599365655	2.444591878	2.90E-16	8.24E-16
HMGB3P10	0.095591667	0.263028429	1.460261969	1.18E-12	2.67E-12
SNORD100	0.687117465	2.078739601	1.597080387	1.37E-09	2.57E-09
NOL12	0.581369131	1.226715821	1.077274701	2.32E-21	9.47E-21
NDNF	23.21243953	4.446979875	-2.384000371	1.09E-28	8.35E-28
KCNF1	0.373770963	1.106831103	1.566208689	1.10E-06	1.71E-06
AC022509.4	0.096918171	0.241580671	1.317665936	2.18E-10	4.28E-10
GJB2	5.232077065	13.06994102	1.320796935	7.03E-06	1.04E-05
LINC01556	0.111325828	0.371889196	1.740084489	3.14E-12	6.92E-12
AL157392.4	0.134044345	0.56613771	2.078442663	1.63E-15	4.41E-15
AC100854.1	0.359447124	1.105694727	1.621101658	2.35E-25	1.32E-24
TPPP3	7.410807617	14.82996417	1.000812433	6.46E-11	1.31E-10
RPL37P1	0.019256936	0.229082903	3.572419703	8.39E-17	2.47E-16
SOX11	0.017447551	0.749261977	5.424373749	2.94E-38	1.90E-36
GYG2	1.261204424	0.542807894	-1.216288528	6.89E-24	3.41E-23
TBC1D4	23.52759981	8.556760379	-1.459217554	1.62E-35	4.02E-34
AC104964.3	0.191484267	0.818304624	2.095412145	2.36E-17	7.21E-17
DTL	0.312437717	1.368807148	2.131278668	1.10E-33	1.78E-32
AC008870.2	0.14045925	0.60869582	2.11556982	5.25E-22	2.25E-21
CYSLTR1	0.476301081	1.090941784	1.195628388	7.06E-18	2.23E-17
CALHM6	1.536161393	12.14379716	2.982817896	2.78E-33	4.17E-32
CLCN5	20.84059503	8.099722102	-1.363452153	2.87E-28	2.10E-27
BAIAP3	3.294646683	1.515653396	-1.120183887	1.99E-25	1.12E-24
LINC00240	0.186624332	0.698194921	1.903492763	1.00E-26	6.32E-26
LMTK3	1.428098524	0.498654714	-1.51798242	3.99E-21	1.60E-20
IGHV1-18	8.956996947	55.70513736	2.636723366	2.05E-06	3.15E-06
IGLC6	0.119058536	0.601370793	2.336583745	3.22E-05	4.57E-05
TMEM92-AS1	0.063029065	0.48206275	2.935131793	7.00E-24	3.46E-23
PRELID1P4	0.152574175	0.382150667	1.324630759	2.68E-17	8.18E-17
AC004241.3	0.600067369	0.27971225	-1.101181042	1.54E-25	8.81E-25
TRIM46	0.131881852	0.508832042	1.947943472	1.05E-18	3.53E-18
ZC3H12D	0.172030263	0.535988113	1.63953862	4.32E-22	1.86E-21
CCDC78	0.077735483	0.538048185	2.791090193	5.72E-14	1.40E-13
GLYATL2	0.316180022	0.834083822	1.399446164	5.45E-13	1.26E-12
TNFAIP3	6.156998153	15.3819319	1.320937669	3.51E-25	1.94E-24
IGLV2-23	14.18486224	75.80777859	2.417993751	1.19E-06	1.85E-06
PKMYT1	0.087302557	0.527427126	2.594875966	1.84E-35	4.49E-34
CASP12	0.302154394	0.719462187	1.251632943	4.62E-09	8.38E-09
AC133552.5	3.985295065	1.805599424	-1.14220868	1.85E-25	1.05E-24
ACO1	25.11522347	12.09053945	-1.054683497	1.86E-24	9.68E-24
FLRT2	0.387488169	0.180849168	-1.099365296	1.45E-17	4.50E-17
AC005586.2	0.276198374	0.554670749	1.005926816	0.041099781	0.046675181
GRHL1	1.034578497	0.404817668	-1.353698949	3.77E-29	3.04E-28
AC007292.1	0.441183501	0.99714951	1.176430994	2.88E-13	6.78E-13

Table S1. The differentially expressed genes(DEGs) were screened out in TCGA

ID	conMean	treatMean	logFC	pValue	FDR
SLC34A3	7.508846262	0.772720889	-3.28057195	1.38E-08	2.43E-08
MPEG1	6.038608163	15.38146585	1.348905032	1.33E-18	4.43E-18
SOWAHB	6.960876361	2.842061975	-1.292330935	5.32E-27	3.43E-26
PWWP2B	5.537118681	2.734881967	-1.017656874	5.56E-21	2.20E-20
TMEM164	11.35632103	4.023300171	-1.497044254	1.44E-39	1.66E-37
HRH2	0.92316926	7.033842333	2.929645906	2.68E-25	1.50E-24
SLC39A4	7.309359653	2.503666145	-1.545702826	5.69E-31	5.75E-30
AC007743.1	0.341307518	0.955309994	1.484896764	3.06E-16	8.68E-16
VIM	109.0103189	639.5199219	2.552524595	4.86E-37	2.00E-35
HOXD13	0.026006922	0.258126209	3.311109087	2.32E-27	1.55E-26
SCGB2A1	2.459267677	0.520661085	-2.239812285	1.97E-27	1.32E-26
AC010333.1	0.122505248	0.2948617	1.267194884	0.025695602	0.029680265
ACSL4	37.34497875	13.09191682	-1.512237936	2.99E-38	1.92E-36
ANKRD29	1.173588663	2.455627122	1.06516467	2.80E-17	8.52E-17
ADTRP	3.606491113	1.116644583	-1.691425805	8.97E-31	8.84E-30
BAAT	0.092684061	4.417637677	5.574810024	2.55E-11	5.29E-11
NNMT	18.31632946	268.4626517	3.87351907	9.25E-36	2.47E-34
SNORD60	0.165953001	1.068878575	2.687251344	4.97E-17	1.49E-16
AC027796.4	0.224249392	1.066627439	2.249880365	1.74E-17	5.35E-17
POLR3B	4.618367056	1.981166385	-1.221032791	5.94E-37	2.35E-35
AP005432.2	13.71497378	0.141972591	-6.593995623	1.25E-41	4.15E-39
AC006026.3	2.111253449	0.707639774	-1.577012735	2.55E-24	1.31E-23
AC093458.1	0.107254215	0.231580698	1.110480659	0.000131423	0.000179462
LINC00865	0.690019631	0.324000456	-1.090641565	4.94E-14	1.21E-13
AC107959.3	0.290349772	0.978538707	1.752837017	1.88E-16	5.42E-16
AC026403.1	1.777309651	6.045366115	1.766134657	3.95E-17	1.19E-16
AL133406.2	0.080592015	0.300502303	1.898667235	9.32E-22	3.91E-21
ADAMTS7	0.340721421	1.895045366	2.475567829	2.91E-36	8.83E-35
FRAS1	5.967982806	2.560852492	-1.220619225	1.16E-27	7.97E-27
MIR3657	0.0774727	0.288102633	1.894822921	1.08E-06	1.68E-06
HEY2	2.067680793	5.174990201	1.32354265	3.24E-18	1.05E-17
RAD51	0.275551534	0.835361447	1.600078402	7.68E-32	8.84E-31
IGHE	0.09999659	0.353360308	1.821189196	0.002436529	0.003043702
RF02126	0.124039694	0.303265945	1.289781626	0.00088134	0.001138476
RN7SL118P	0.029090063	0.240621454	3.048166972	8.78E-10	1.66E-09
ACAD11	0.706895113	1.668150987	1.238681803	4.26E-09	7.74E-09
CYTH4	1.108724722	4.447694386	2.004156447	9.32E-33	1.27E-31
HLA-DPB1	44.02344333	186.7294374	2.084605486	7.30E-32	8.46E-31
FOXL1	0.652856172	1.344571145	1.042308996	2.23E-11	4.67E-11
MRNIP	1.38076694	3.261979997	1.240278108	2.67E-18	8.68E-18
GZMM	0.599017491	2.540450229	2.084414166	2.72E-28	1.99E-27
COX4I2	2.715824143	19.34008474	2.832132147	3.65E-30	3.34E-29
AC012645.3	0.047133073	0.428087156	3.183092906	1.21E-29	1.04E-28
SLC27A3	2.442062149	6.898973392	1.49828178	8.14E-32	9.33E-31
TRPA1	0.12684434	1.177720178	3.214865746	9.75E-32	1.10E-30
AL157871.5	0.134547868	0.344312933	1.35560084	1.22E-07	2.02E-07
AC105339.3	0.067399108	0.245551122	1.865222006	9.69E-10	1.83E-09
ST3GAL4	15.43631657	6.453408047	-1.258195382	7.10E-25	3.83E-24
SCARNA8	0.054421215	0.543763665	3.320738681	1.54E-13	3.68E-13
PLPPR1	8.164899235	0.404576817	-4.334949524	1.44E-38	1.01E-36
PIWIL4	0.348006944	0.889854624	1.354453566	2.59E-23	1.23E-22
TCN1	0.076218819	0.681919131	3.161381508	0.000101671	0.00013971
AC007991.2	0.043205717	1.357644529	4.973739767	2.21E-23	1.06E-22
COL4A2-AS1	0.140709301	0.553167068	1.974997574	1.78E-16	5.14E-16
ATP5MC1P4	0.079867404	0.33561703	2.071137203	3.57E-17	1.08E-16

Table S1. The differentially expressed genes(DEGs) were screened out in TCGA

ID	conMean	treatMean	logFC	pValue	FDR
KCNJ12	1.966168072	0.39195177	-2.326638607	9.35E-35	1.91E-33
RNU6-856P	0.195576228	0.438957347	1.166349741	0.000968959	0.001247665
MSI2	8.722535319	4.260062175	-1.033873048	2.86E-33	4.26E-32
AC018529.1	0.141920327	0.292409824	1.042910546	2.56E-05	3.66E-05
AC019181.1	1.40129884	0.569945204	-1.297869531	1.12E-22	5.03E-22
PLXDC1	0.79305821	5.88724404	2.892093764	6.26E-38	3.57E-36
FCGR1A	0.358387101	3.044816572	3.0867647	2.66E-36	8.19E-35
CD40LG	0.375650774	1.065211627	1.503676102	2.32E-18	7.59E-18
LINC02015	0.355904689	2.235593755	2.651095206	4.83E-19	1.66E-18
LINC01176	0.548287228	2.119976966	1.951044818	2.84E-21	1.15E-20
PPP1R3B	8.881540542	22.10895908	1.315749258	4.54E-28	3.25E-27
MIR6730	0.183237918	0.671382605	1.873417083	7.99E-11	1.61E-10
AC005082.1	6.499592926	0.791732799	-3.037263841	1.00E-34	2.04E-33
PLEKHO2	9.279880028	19.24005386	1.051934779	2.53E-25	1.41E-24
AC079380.1	0.052990525	0.233624699	2.140386476	8.99E-18	2.82E-17
IGKV3-11	16.25485653	79.24572673	2.285462318	1.83E-06	2.82E-06
PCDHB8	0.329667093	0.702360535	1.091201899	0.000267719	0.00035798
HIST1H3E	0.921124033	2.126286268	1.206868505	2.07E-08	3.61E-08
C5orf67	1.976399426	0.30011945	-2.719265817	2.85E-28	2.09E-27
AL031710.1	10.23423708	1.697657815	-2.59178596	5.68E-33	8.03E-32
AC090197.1	0.111106422	0.503296887	2.179467472	7.57E-35	1.58E-33
MYEOV	0.056720445	7.543812285	7.055281122	6.10E-37	2.41E-35
HIST1H3H	0.456986592	2.772094947	2.600752931	5.64E-21	2.23E-20
GRAMD4	5.542686083	18.89700809	1.769500629	7.19E-30	6.29E-29
BATF	0.477197401	3.853560308	3.01353388	6.08E-34	1.04E-32
AL357992.1	0.093405572	0.412875497	2.144126282	2.80E-07	4.53E-07
SLC2A3	5.570609986	22.7955221	2.032843235	1.51E-26	9.42E-26
FAP	0.453600052	1.00200623	1.143398766	1.14E-14	2.92E-14
SLC12A3	73.30503179	2.99324404	-4.614130419	9.91E-38	5.25E-36
LINC01160	0.086834663	0.226275262	1.381735901	1.33E-11	2.81E-11
AC012645.4	0.058530354	0.338718391	2.532829414	1.68E-17	5.19E-17
SARDH	4.996210983	2.202010597	-1.182012989	5.11E-08	8.69E-08
AC015849.3	0.466717133	1.71093629	1.874165705	3.75E-20	1.40E-19
AP001107.8	0.065399632	0.397667216	2.6042072	1.52E-12	3.41E-12
ANGPTL7	0.444157062	0.212461096	-1.063871234	2.38E-13	5.63E-13
PLCB2	0.808865414	3.622450668	2.162994463	4.12E-31	4.25E-30
AC100823.1	0.237025927	0.625845801	1.400762366	0.00037971	0.00050215
GMIP	1.874372654	5.701016745	1.604811427	7.66E-33	1.06E-31
ARRDC5	0.025069434	0.36263952	3.854534831	1.69E-34	3.25E-33
HP	0.15478228	22.07178204	7.155818989	0.000889275	0.00114794
CYS1	128.2017628	30.65217619	-2.064354691	2.56E-40	4.21E-38
SH3GL1P1	0.054448781	0.310310922	2.510742822	1.56E-30	1.49E-29
RNU4-5P	0.04106436	0.239023358	2.54119288	1.37E-12	3.08E-12
AC092295.2	1.515496647	0.736782978	-1.040479024	1.53E-30	1.46E-29
AL356056.3	0.499036514	0.248037455	-1.008587388	4.21E-15	1.11E-14
AC003070.1	0.283998638	1.390484124	2.291631357	2.55E-23	1.21E-22
SIGLEC10	0.588047027	4.743193466	3.011855275	6.32E-37	2.48E-35
AGPAT4-IT1	0.126485266	0.255233265	1.012847036	5.08E-06	7.60E-06
PRSS36	0.270068699	0.565108794	1.0652022	1.45E-23	7.02E-23
AL359853.1	0.033341461	0.342614162	3.361195576	3.98E-30	3.61E-29
PLP2	32.88957722	70.38799593	1.097698947	2.30E-13	5.43E-13
NPM2	1.706402461	0.806504866	-1.081202808	2.18E-23	1.04E-22
KAAG1	0.779391489	0.376125563	-1.051133818	8.56E-21	3.34E-20
LGALS12	0.207157898	2.365075111	3.513083275	3.24E-24	1.65E-23
P2RX7	0.568997307	3.722196398	2.709660451	1.53E-37	7.51E-36

Table S1. The differentially expressed genes(DEGs) were screened out in TCGA

ID	conMean	treatMean	logFC	pValue	FDR
NID2	3.515416139	7.152124139	1.024676298	2.97E-09	5.45E-09
CDKL2	4.504130528	1.504957873	-1.581525533	1.57E-28	1.19E-27
CACNA2D3	1.874950892	0.436264911	-2.103576463	8.17E-38	4.46E-36
AC005086.2	0.045484732	0.238313234	2.389404821	0.002996051	0.003716945
AC004918.1	1.559502676	4.310374283	1.46672712	6.28E-24	3.12E-23
LINC01874	6.198979607	0.912968108	-2.763394389	1.83E-22	8.12E-22
AL844908.1	0.546569851	1.171249241	1.099570324	0.000976891	0.001257176
C3	22.99807194	235.7467393	3.357652995	2.34E-33	3.56E-32
SLC5A11	1.551706779	0.198199633	-2.968829765	2.44E-18	7.98E-18
LBHD1	0.481538508	1.000807954	1.055442082	1.79E-21	7.37E-21
TOX	4.555773763	1.986685612	-1.197332518	2.67E-21	1.09E-20
GOLGA8A	0.516654217	3.861288915	2.901811555	6.98E-17	2.06E-16
FGF7	1.747524878	0.800011909	-1.127219613	6.85E-17	2.03E-16
MUC15	28.05434097	0.18735572	-7.22630019	3.02E-42	1.36E-39
TGM2	28.41593422	67.49115931	1.247998388	3.00E-18	9.74E-18
AP001767.4	0.302337569	0.724697579	1.261218815	4.93E-07	7.88E-07
ACKR3	8.768886819	42.47723586	2.276224274	1.34E-31	1.48E-30
ZNF582-AS1	2.039757036	0.802189067	-1.346383109	1.45E-31	1.60E-30
AC008759.2	0.111313096	0.25275364	1.183108537	0.00020717	0.000278954
HAPLN3	0.602839985	3.170850215	2.395022714	3.22E-32	3.95E-31
AC008556.1	1.932685154	0.639892982	-1.594704085	1.44E-13	3.44E-13
PLA2G6	0.954664944	2.340319577	1.293639158	3.84E-11	7.90E-11
LINC01659	0.765055267	0.16044609	-2.253475342	2.26E-17	6.92E-17
TM6SF2	0.798655661	1.804933633	1.176300264	1.37E-05	2.00E-05
IGHV3-48	1.24586632	9.322350761	2.903544519	1.91E-06	2.94E-06
CCDC85B	5.374663751	14.15027453	1.396583638	6.19E-18	1.97E-17
RAP1GAP	54.51348382	8.597448778	-2.664632602	7.93E-37	3.03E-35
ITGA5	7.631388542	29.35314983	1.943497838	9.55E-32	1.08E-30
AC103591.3	0.275541812	0.999781431	1.859341475	4.34E-15	1.14E-14
SNORA55	0.349013507	1.079014501	1.628359479	6.36E-09	1.14E-08
HPCA	0.124252004	0.842036195	2.760613134	4.86E-31	4.96E-30
CLDN10	38.11023033	7.829517671	-2.283182988	5.36E-40	7.56E-38
CETP	2.257560383	5.432347509	1.266811196	2.15E-18	7.06E-18
SGPP1	23.75221722	9.93914416	-1.256868658	2.51E-38	1.65E-36
WWTR1-IT1	0.081702743	0.388166828	2.248220415	6.69E-09	1.20E-08
KIR2DL4	0.071827739	0.384075366	2.418776434	3.36E-28	2.44E-27
AC126755.1	0.119332308	0.262168613	1.135510287	3.96E-12	8.67E-12
B3GNT8	3.554914364	1.36308227	-1.382942167	1.57E-24	8.24E-24
BLOC1S5-TX	0.729780425	0.198290467	-1.879847132	2.11E-15	5.69E-15
MAP3K7CL	1.753906256	7.416105473	2.080090123	3.23E-30	2.96E-29
SPN	0.684516422	3.083263994	2.171301362	2.71E-29	2.22E-28
AC007224.2	0.078824584	0.297728144	1.917278057	2.91E-11	6.03E-11
SNHG20	1.119141617	2.613429903	1.223551857	3.19E-25	1.77E-24
SMPDL3A	12.82003822	46.65686771	1.863688893	6.23E-31	6.26E-30
ODF3L1	0.241377558	0.484579905	1.005443035	1.68E-10	3.33E-10
HES5	0.021555276	0.312271753	3.856689166	2.35E-27	1.57E-26
AL627309.6	0.25032963	0.918761832	1.875861863	5.30E-08	9.00E-08
AC131971.1	0.038571792	0.241308557	2.645260993	1.26E-09	2.37E-09
GDF15	79.55718229	34.44815336	-1.207565533	1.94E-16	5.58E-16
ASAP3	7.348217	3.347026746	-1.134514151	2.86E-33	4.26E-32
AC013553.3	0.125077088	0.492833906	1.978283979	9.77E-22	4.10E-21
AC109826.1	0.089014179	0.65232491	2.873483652	1.83E-37	8.77E-36
PPM1E	0.922726528	0.323920601	-1.51026291	2.61E-32	3.28E-31
AC138356.2	2.318784028	0.976440248	-1.247764785	4.52E-32	5.40E-31
TNFRSF10B	7.282567361	20.89170975	1.520411519	2.66E-37	1.22E-35

Table S1. The differentially expressed genes(DEGs) were screened out in TCGA

ID	conMean	treatMean	logFC	pValue	FDR
KCNJ15	35.48048363	14.53452865	-1.287541388	2.06E-19	7.30E-19
TIMD4	1.461899504	4.944896481	1.75809618	1.85E-16	5.33E-16
GPR27	3.575302901	1.011471444	-1.821609882	1.12E-30	1.09E-29
TDRD10	0.34652835	0.790732507	1.190216348	1.42E-10	2.83E-10
RGS19	2.453337944	8.046626505	1.713638093	3.22E-36	9.59E-35
RN7SL333P	0.156997919	0.421127418	1.423511374	1.30E-11	2.77E-11
AL133243.3	0.147665547	0.297547904	1.010788698	3.69E-12	8.10E-12
RPL21P16	2.249154601	5.462976527	1.280304392	1.65E-08	2.89E-08
FOXJ3	8.211005361	26.07583724	1.667082793	2.66E-24	1.36E-23
EDN1	9.967852014	34.23102498	1.779949937	4.86E-18	1.55E-17
FUT2	0.93987692	0.254673434	-1.883823369	3.48E-09	6.36E-09
RGCC	28.49081399	86.71599104	1.605801221	6.65E-24	3.30E-23
EVPL	6.259346236	2.102499113	-1.573906789	6.96E-30	6.12E-29
MAP6D1	0.406413233	0.858819051	1.079406815	6.74E-22	2.86E-21
PDGFRA	7.540041465	1.449602308	-2.3789153	9.55E-32	1.08E-30
JAG2	2.464757322	7.855815598	1.672315458	2.89E-27	1.92E-26
AC124798.1	1.111123411	2.544946923	1.195616504	1.43E-13	3.42E-13
AC022432.1	0.039475432	0.639032419	4.01686216	3.74E-27	2.45E-26
AC023024.1	0.244518768	1.823466286	2.898666419	3.37E-31	3.52E-30
ASB2	0.308500828	0.840147932	1.445369015	8.24E-18	2.59E-17
MAN1A1	58.70055556	21.54610194	-1.445947273	7.66E-38	4.22E-36
AC124944.3	0.175001827	1.050264643	2.58531101	7.89E-23	3.59E-22
AC114284.1	0.059152957	0.226879308	1.939402838	7.63E-23	3.48E-22
AC010789.1	0.279944814	0.748072196	1.418035057	0.025922636	0.02993243
MIR34AHG	0.087719431	0.276289166	1.655210638	3.29E-21	1.33E-20
KCNMA1	0.704355198	3.483006472	2.305958102	5.69E-25	3.09E-24
GRAP2	0.204742758	0.68192207	1.735794455	3.09E-25	1.72E-24
HAVCR1	6.007565563	27.91057405	2.215959404	2.90E-16	8.24E-16
IDO1	1.222919186	12.36748266	3.338150901	2.43E-36	7.58E-35
CYP4A11	60.44897469	20.9747067	-1.527067232	2.57E-07	4.18E-07
TRIP13	0.459293692	1.571612808	1.774756957	3.39E-32	4.15E-31
RUSC1-AS1	0.482382394	1.460941908	1.598649654	1.31E-16	3.81E-16
AC092296.3	0.689801607	0.240266017	-1.521548877	1.48E-06	2.29E-06
TRBV9	0.190927867	1.639053492	3.10176335	5.33E-23	2.46E-22
LY6E	33.87197125	108.7793846	1.683241314	6.10E-24	3.04E-23
AC020913.3	0.074073049	0.61797341	3.060524138	1.27E-13	3.04E-13
AL157373.2	2.742082392	0.618913586	-2.147462024	3.94E-35	8.82E-34
SIGLEC17P	0.100319781	0.349272618	1.799747441	1.52E-26	9.49E-26
KLHL14	5.137352938	0.361725161	-3.828059331	6.06E-41	1.48E-38
AC073487.1	0.133786008	0.604039306	2.174715197	2.05E-19	7.27E-19
AC107021.1	0.222419802	1.197723969	2.428938314	1.58E-24	8.27E-24
NME1-NME2	0.199332324	0.405114842	1.023155265	4.55E-17	1.36E-16
TMEM174	42.01655936	9.471136813	-2.149348521	4.99E-11	1.02E-10
TRAV19	0.158428723	1.430488225	3.174601803	4.24E-25	2.33E-24
LINC01358	0.244871587	3.127218933	3.674782937	7.41E-32	8.58E-31
AL136040.1	1.023321563	0.372421409	-1.458251645	2.42E-29	2.00E-28
PTGER3	36.22309219	8.233594074	-2.137315479	1.06E-33	1.73E-32
DGKH	1.061966836	2.133349009	1.006381292	3.91E-18	1.26E-17
GAPDHP35	1.073384467	2.827620574	1.397421629	9.29E-12	1.99E-11
APEH	33.74179671	14.1742646	-1.251262911	6.65E-36	1.84E-34
CARD11	0.949591299	4.033692469	2.086722476	6.80E-24	3.36E-23
RPS14	88.95319097	183.6508046	1.045846952	1.08E-29	9.28E-29
EGF	74.50253505	1.589919098	-5.550264254	3.88E-38	2.40E-36
AL355102.4	2.675754908	0.422136817	-2.664163409	1.53E-32	2.02E-31
CD244	0.212198227	1.08118021	2.3491225	1.61E-32	2.11E-31

Table S1. The differentially expressed genes(DEGs) were screened out in TCGA

ID	conMean	treatMean	logFC	pValue	FDR
RNU6-388P	0.135559044	0.343546784	1.341585206	1.40E-05	2.03E-05
CRIP3	0.35303149	0.759888053	1.10599002	1.77E-05	2.56E-05
TNFSF8	0.475100429	1.746131819	1.877858061	1.89E-25	1.07E-24
GAL3ST3	3.218246006	0.155703104	-4.369404999	9.80E-39	7.39E-37
RIC3	1.235257306	0.605618591	-1.028330191	1.02E-21	4.27E-21
AC023906.5	0.126820873	0.476635068	1.910092889	8.17E-20	2.98E-19
ANXA1	17.07867731	42.6919013	1.321766168	9.46E-25	5.05E-24
AL353593.3	0.229393571	0.746300554	1.701931799	1.84E-09	3.42E-09
BIN2	1.10576349	4.505740075	2.026721247	2.81E-35	6.57E-34
RF00275	0.102669382	0.369845992	1.848918632	6.68E-12	1.44E-11
LINC00864	3.000613271	0.065796591	-5.511100753	1.07E-44	6.87E-42
AC016734.1	0.327819577	0.760878033	1.214763198	8.93E-13	2.03E-12
FAS	4.339167014	10.13104214	1.223292565	1.32E-26	8.24E-26
DPRXP4	0.107680444	0.518838141	2.26852828	5.05E-24	2.53E-23
AC093458.2	0.258029969	0.667630255	1.371510699	4.29E-11	8.78E-11
ENPEP	29.36913641	58.85286841	1.002811888	3.42E-10	6.64E-10
PTPRN	0.164265237	1.186600894	2.852735669	2.86E-33	4.26E-32
TMEM176A	62.54046147	155.3229183	1.312408949	6.95E-24	3.43E-23
AC073486.1	5.333237005	54.77972377	3.360558644	1.56E-28	1.18E-27
AC019117.1	0.131656975	0.788094498	2.581584676	6.95E-13	1.60E-12
AC002064.2	0.292878027	0.86220948	1.557738465	8.61E-09	1.54E-08
ITPKA	0.09901447	1.438261707	3.860543031	2.01E-27	1.35E-26
CD68	0.22119933	1.935416552	3.129225182	3.70E-37	1.59E-35
FFAR4	0.06821887	0.476398208	2.803925232	6.91E-31	6.91E-30
NUDT4	14.57659069	4.183296724	-1.800941091	4.48E-36	1.29E-34
AC099677.1	1.318743608	4.291603918	1.702352831	2.78E-11	5.77E-11
EGR3	6.343968006	2.993250131	-1.083672652	4.58E-05	6.44E-05
TNNT3	0.09647057	1.188196383	3.6225406	5.14E-07	8.22E-07
AL049780.1	0.141855743	0.570798577	2.008557176	4.22E-14	1.04E-13
AP000812.1	0.081465681	0.28189464	1.790891727	1.10E-17	3.43E-17
NPTX2	1.978962318	67.65095748	5.095294306	1.76E-39	1.92E-37
TKFC	11.84667872	5.187293183	-1.191428831	2.19E-25	1.24E-24
EHD3	17.39404682	7.552602992	-1.203547765	3.39E-10	6.58E-10
AC044781.1	0.009184703	0.226240156	4.622478132	1.06E-21	4.44E-21
TMEM25	7.513849292	3.245368238	-1.211169996	2.29E-35	5.46E-34
AP003469.2	0.071256052	0.730920074	3.358629201	5.73E-35	1.24E-33
BX679664.1	0.111591715	0.325926244	1.546315601	4.58E-08	7.81E-08
SFXN2	9.008744128	1.860068199	-2.275970481	1.03E-30	1.01E-29
RNU6-88P	0.200936085	1.121783265	2.48098539	8.67E-14	2.10E-13
TDO2	0.149940492	0.619882968	2.047605828	3.02E-08	5.20E-08
SMOX	3.338215635	7.1817972	1.105267765	2.10E-20	7.96E-20
SLC37A2	0.485046509	3.289541516	2.761691526	4.29E-36	1.24E-34
TESPA1	0.195959615	1.094556215	2.481717786	2.25E-30	2.11E-29
HOXD8	40.30395319	11.82344717	-1.769270632	1.64E-40	3.18E-38
IRF6	16.37202504	5.311937999	-1.623922566	1.14E-34	2.28E-33
PLA2G7	0.377565883	4.702069367	3.638495509	1.20E-36	4.28E-35
AC016949.1	0.090029061	0.343306084	1.931032742	3.61E-17	1.09E-16
AL136115.2	0.075701617	0.271264446	1.841303939	3.51E-06	5.32E-06
USP53	7.581357681	2.91940628	-1.376781234	1.54E-35	3.84E-34
MIR3142HG	0.115701526	0.552081466	2.254473278	4.13E-21	1.65E-20
SLC43A1	5.120039725	1.976245468	-1.373392849	4.09E-24	2.06E-23
IGHV3-21	4.432468157	31.22557114	2.816545788	2.22E-06	3.40E-06
CCDC84	0.964298764	3.305318058	1.777237	4.14E-23	1.93E-22
CD3G	0.354834962	2.149054049	2.598481698	6.83E-28	4.79E-27
RN7SL221P	0.176136654	0.365825988	1.054462398	0.000311545	0.000414641

Table S1. The differentially expressed genes(DEGs) were screened out in TCGA

ID	conMean	treatMean	logFC	pValue	FDR
CR2	2.50528243	0.183293138	-3.77274857	1.14E-28	8.71E-28
FSTL1	24.33877467	62.9156698	1.370162843	6.50E-21	2.56E-20
AL121894.2	0.267122849	0.581679101	1.122720085	3.95E-15	1.04E-14
BNIP3P15	0.06137006	0.306205204	2.318891906	3.21E-09	5.87E-09
SERPINF2	46.09460572	95.70308854	1.053967557	6.55E-08	1.10E-07
RNU4-89P	0.089000495	0.362601343	2.026498998	1.02E-06	1.59E-06
ZDHHC20-IT	0.097635697	0.286261883	1.551854961	2.66E-14	6.66E-14
BTN2A3P	0.511304397	1.045100729	1.031387661	3.71E-16	1.05E-15
MIR548P	0.011306763	0.321272553	4.828539931	2.73E-12	6.03E-12
CCR7	0.523290493	1.614489439	1.625394051	1.00E-19	3.62E-19
PLA2G4C	1.71507365	3.575800503	1.059995719	1.61E-20	6.18E-20
RF00134	0.121167056	0.628173621	2.374165866	1.67E-13	3.97E-13
RPL26P35	0.017367597	0.267742917	3.946378379	4.30E-12	9.39E-12
GLIPR1	1.906767725	5.421736673	1.507625934	2.59E-23	1.23E-22
UBA52P7	0.095170169	0.321917656	1.758110369	1.06E-08	1.88E-08
MIF	23.23782518	51.43787337	1.146355944	1.44E-20	5.55E-20
SLC11A1	0.304216049	1.604221745	2.398705405	1.83E-33	2.83E-32
AC074033.1	0.65665951	0.147381221	-2.155592794	1.07E-37	5.57E-36
PRR7-AS1	0.03829638	0.382321865	3.319507784	1.99E-33	3.06E-32
CYP1B1	23.12736461	10.32415432	-1.163577267	1.52E-23	7.37E-23
AC004918.3	1.140033119	2.304305472	1.015256244	8.81E-15	2.28E-14
OSMR-AS1	0.209446442	0.499302905	1.253333925	4.20E-17	1.26E-16
RF00154	0.36463123	1.838376607	2.333922311	1.07E-19	3.86E-19
TPRG1	0.064556699	0.381203682	2.561923333	4.18E-29	3.36E-28
SMTNL1	0.051976402	0.407928257	2.972386766	1.53E-25	8.75E-25
ARHGAP24	25.16775225	8.285874178	-1.602850556	1.57E-39	1.77E-37
AC241644.2	1.403160831	0.132846593	-3.400847245	1.13E-37	5.83E-36
AC020978.4	0.763127212	0.190394738	-2.002929968	5.80E-36	1.63E-34
NAPSA	16.14601221	7.577385027	-1.091405926	3.89E-16	1.10E-15
HIST1H2APS	0.496474485	0.199505504	-1.315291026	0.000670164	0.00087132
NELL1	8.886163536	0.154001832	-5.850541278	4.63E-42	1.98E-39
ARHGAP27P	0.31731414	1.367695025	2.107762852	9.89E-23	4.48E-22
AL731567.1	0.103841281	0.831823464	3.00189729	1.53E-25	8.75E-25
SLC47A1P2	0.097415891	0.655742694	2.750900796	5.16E-21	2.05E-20
CDO1	1.754360218	0.56376283	-1.637784737	9.38E-34	1.54E-32
MIR5587	0.340544201	1.427350464	2.067425638	4.42E-09	8.03E-09
TRAV10	0.048122876	0.335127569	2.799915593	8.40E-15	2.17E-14
SEPT1	0.907773419	3.300159965	1.862131806	8.90E-34	1.48E-32
DPP9	4.052760097	10.89226839	1.426327754	1.26E-37	6.36E-36
IGHV1-67	0.185484984	0.771117159	2.055647673	0.000167899	0.000227519
CALB1	53.47279118	0.31468416	-7.408756613	1.11E-33	1.79E-32
PDXDC2P-NI	0.252384983	0.796080532	1.657288313	5.66E-16	1.58E-15
AL109947.1	0.077280058	0.245354056	1.666697032	3.25E-06	4.93E-06
AC010201.2	0.34135982	1.024145501	1.585055536	1.02E-12	2.31E-12
PTAFR	1.975882161	5.364454696	1.440934618	2.14E-19	7.57E-19
AL645939.2	0.050749796	0.332269615	2.710880437	2.69E-17	8.21E-17
G6PC	29.90980479	2.819185278	-3.407268294	4.61E-13	1.07E-12
AP003064.2	0.066738311	0.244467486	1.873055532	1.77E-08	3.10E-08
FUT6	10.75231009	4.6021329	-1.224272099	9.76E-06	1.44E-05
SUCNR1	17.03261574	4.083018258	-2.060592086	3.30E-22	1.44E-21
SNORD12B	0.260109582	1.217535684	2.226772605	1.24E-15	3.39E-15
LINC00528	0.078201521	0.397998823	2.347495584	1.12E-34	2.25E-33
SERPINE2	7.025307389	16.96775747	1.272162648	3.84E-10	7.42E-10
LPL	18.19948001	7.88252081	-1.207168252	2.99E-09	5.49E-09
ENTPD1	3.469673056	7.055871642	1.024024591	3.97E-28	2.86E-27

Table S1. The differentially expressed genes(DEGs) were screened out in TCGA

ID	conMean	treatMean	logFC	pValue	FDR
PPL	14.98075406	5.063256254	-1.564972837	3.38E-18	1.09E-17
ARL11	0.224860272	0.865995408	1.945330583	5.21E-33	7.46E-32
AC093151.2	0.090825672	0.322229266	1.826915484	2.33E-09	4.31E-09
AL163636.2	0.203478468	0.471588027	1.212650958	8.33E-15	2.15E-14
OAS2	3.813517181	9.641639198	1.338156244	2.45E-25	1.37E-24
AP000345.2	0.320649481	0.679099995	1.082626948	0.014434704	0.01699152
TTC36	3.104165213	0.427300864	-2.860881203	2.24E-33	3.42E-32
AC010761.1	0.345711257	1.065560814	1.623973447	4.06E-19	1.40E-18
SEC62-AS1	0.119914924	0.291289826	1.280444088	2.01E-08	3.51E-08
AC090912.2	0.11188335	0.260778219	1.220828024	0.001521335	0.001931802
RPL28	37.06324458	77.21067196	1.058811086	1.81E-29	1.51E-28
C1QL4	0.496236658	4.16423225	3.068950316	4.03E-10	7.78E-10
TRGV7	0.313894805	1.206958911	1.943023505	4.03E-25	2.21E-24
AC091564.1	0.049896461	0.246097576	2.302221041	1.45E-16	4.21E-16
AC105384.1	1.693074351	0.060408466	-4.808750773	1.89E-33	2.92E-32
AC007347.1	0.069902336	0.446118619	2.674014787	3.59E-18	1.16E-17
MIR4312	0.113390118	0.448596935	1.984124847	4.91E-08	8.36E-08
LAMB4	0.585920408	0.219031702	-1.419565004	1.51E-33	2.38E-32
AL159163.1	0.091585242	0.427084014	2.221332853	3.96E-28	2.86E-27
PTCSC3	4.18756292	0.349949276	-3.580893141	1.40E-38	1.01E-36
AC034213.1	0.078500398	0.255476125	1.702416598	0.00128209	0.001637144
AL096701.1	0.073557564	0.380780096	2.372012458	7.35E-20	2.69E-19
IGHV3OR16-(	0.049234406	0.266705362	2.437508075	0.004213759	0.005170633
AL445433.1	0.113416865	0.266623292	1.233167635	2.50E-08	4.33E-08
TMEM145	0.043538696	0.859505075	4.303136062	4.90E-30	4.40E-29
TRIM15	1.915216392	4.752751019	1.311255421	3.98E-12	8.71E-12
RANBP3L	19.46878908	0.490052256	-5.312083747	9.27E-40	1.19E-37
MAGI2	2.910392611	0.96474223	-1.592998361	1.66E-37	8.06E-36
AC078922.1	0.069260199	0.285650911	2.044154701	9.86E-08	1.65E-07
AL132657.1	0.280932633	0.978174418	1.799867516	1.83E-28	1.37E-27
UNC5A	0.056414074	0.587547082	3.380577428	6.07E-28	4.29E-27
AC026740.1	0.249513248	0.531345955	1.090535074	2.10E-10	4.13E-10
SLC5A8	3.18239975	9.818593799	1.625401351	0.005550213	0.006751021
TRBV19	0.319424665	2.130600632	2.73771257	1.23E-22	5.51E-22
LPIN2	20.93879567	10.24070129	-1.03186395	4.63E-30	4.17E-29
AL445248.1	0.076766094	0.245366598	1.676397725	3.57E-12	7.85E-12
MIR7848	0.052277588	0.719554979	3.782840452	1.72E-18	5.68E-18
IGHV3-19	0.14152334	1.512142331	3.417482034	0.006781431	0.008197281
MMP2-AS1	0.020771671	0.620347168	4.900386635	9.41E-32	1.07E-30
PRRT2	0.211253089	1.18761652	2.491024728	6.31E-26	3.73E-25
SLC6A19	43.41474775	10.47344008	-2.051449818	3.52E-10	6.82E-10
AL513327.3	0.086487319	0.23178849	1.422248414	1.46E-07	2.41E-07
VEPH1	11.1272921	3.966368181	-1.488212037	1.97E-33	3.04E-32
EPHA3	0.854871614	2.921112756	1.772738373	1.42E-20	5.48E-20
RNASE4	0.982363558	2.028999201	1.046439348	1.61E-14	4.09E-14
AC090004.1	0.075303686	0.308365584	2.033849373	1.16E-21	4.84E-21
LGI4	0.924436335	21.86502865	4.563907463	4.00E-30	3.63E-29
GGT4P	0.472648144	1.438478078	1.60570474	3.73E-06	5.65E-06
AC011389.2	0.047000466	0.274870155	2.548003311	1.73E-09	3.23E-09
ADH1C	15.59172784	1.413523316	-3.463413227	3.42E-39	3.20E-37
AC092162.3	0.270544144	0.864878032	1.676632681	1.13E-19	4.09E-19
RELT	0.328711904	1.41369825	2.104578605	9.91E-38	5.25E-36
SNORA71A	0.204155151	2.271975998	3.476209723	3.65E-17	1.10E-16
AC015911.3	0.100496825	0.821778501	3.031599658	7.92E-31	7.85E-30
AC090616.6	0.094940498	0.352983243	1.894504178	1.56E-25	8.87E-25

Table S1. The differentially expressed genes(DEGs) were screened out in TCGA

ID	conMean	treatMean	logFC	pValue	FDR
PEG3	2.886837717	0.568261782	-2.344862413	5.67E-40	7.89E-38
TTC3P1	2.211994278	0.908048337	-1.284506652	1.34E-31	1.48E-30
ERVE-1	3.415181925	0.098634548	-5.113725565	1.27E-46	9.67E-44
AC008750.5	0.081667123	0.277560328	1.764974069	1.53E-13	3.65E-13
GPR183	3.441182786	9.549883258	1.472578571	3.04E-20	1.14E-19
DOCK6	3.584907708	8.287723747	1.209039937	6.17E-25	3.34E-24
AC120057.4	0.155292965	0.656382502	2.079544301	4.95E-11	1.01E-10
IGKV1D-39	0.06654096	0.620356125	3.220782062	0.000371867	0.000492062
CAPN11	0.14195949	0.646903657	2.188071576	4.06E-34	7.22E-33
CARD9	0.263058502	0.747432322	1.506559276	1.14E-22	5.12E-22
PANO1	0.092043448	0.265765411	1.529766423	2.07E-25	1.17E-24
C1QTNF6	0.693557922	3.347356726	2.270934028	9.60E-37	3.54E-35
MIR570	2.855735589	10.53021831	1.882601034	1.02E-15	2.82E-15
C3orf70	0.725644515	2.121469403	1.547729004	1.22E-22	5.48E-22
RPS26P31	0.203414457	0.455562329	1.163226234	0.000618433	0.000805791
NR4A3	7.697664926	3.264947522	-1.23736107	7.83E-09	1.40E-08
AL449212.1	0.465918425	1.321462386	1.503986073	1.64E-14	4.15E-14
IL1RAP	0.788852025	1.612824186	1.031762573	1.46E-15	3.99E-15
ZNF503-AS2	2.694723569	0.838852464	-1.683648287	8.20E-35	1.69E-33
DNER	9.593789833	1.542210449	-2.637101188	1.49E-36	5.10E-35
CYP4F3	5.655357784	1.225016479	-2.206817142	3.06E-11	6.34E-11
NPIPB11	0.065136228	0.337381343	2.37284812	9.34E-21	3.63E-20
SERPINE1	26.71965024	121.9353135	2.190142975	1.69E-15	4.58E-15
PROX1	6.693235572	0.318916123	-4.391454849	2.08E-37	9.78E-36
AQP7P1	1.063368198	0.213133942	-2.318808954	1.57E-19	5.61E-19
AL133342.1	0.255520559	0.591895246	1.211902496	1.38E-13	3.30E-13
RASSF5	1.457845893	4.860576768	1.737289295	9.68E-29	7.47E-28
AC004486.1	0.156162079	0.572229531	1.87354979	2.51E-11	5.23E-11
CD53	9.384634139	32.94409817	1.811647627	4.78E-30	4.30E-29
TRPM3	2.045051509	1.005392538	-1.024378294	1.43E-11	3.02E-11
AP006284.1	0.309567291	4.084617159	3.721875914	7.15E-25	3.85E-24
MMP13	0.015097142	0.441209196	4.869115487	2.22E-05	3.18E-05
RPL22L1	3.563499847	9.217157221	1.371026997	1.91E-30	1.81E-29
AL627309.7	0.42856317	1.256962068	1.552361336	2.79E-05	3.97E-05
LINC01619	0.121932142	0.31760781	1.381167912	6.79E-14	1.66E-13
ERBB4	5.04758368	0.34427227	-3.873971037	1.75E-38	1.21E-36
AC000089.1	0.402306081	0.830288481	1.045319141	9.67E-12	2.07E-11
C2orf27A	0.669176872	1.786289748	1.416506625	5.00E-22	2.15E-21
VAMP5	29.29337208	66.9035207	1.191507855	1.96E-29	1.64E-28
AC073655.2	0.13464933	0.510042223	1.921409629	2.04E-20	7.76E-20
PLA2G2D	0.10591699	1.17059791	3.466239676	2.11E-19	7.47E-19
TRBV5-1	0.240400225	1.300121923	2.43513677	2.86E-21	1.16E-20
CCL3	1.57382809	5.668468198	1.848680963	8.77E-18	2.75E-17
AL358394.1	0.013271119	0.238688848	4.168769203	2.88E-09	5.30E-09
OPA1-AS1	0.062135175	0.237333204	1.93343184	7.76E-08	1.30E-07
AC018742.1	0.164483765	1.866376861	3.504223227	1.98E-06	3.04E-06
NLRC5	1.011753493	4.915886327	2.280593726	1.78E-39	1.92E-37
F11-AS1	2.07185343	0.10841946	-4.25622631	1.54E-38	1.07E-36
AC008050.1	0.076283648	0.30441559	1.996596508	4.99E-15	1.31E-14
ADAM18	0.032297591	0.55225607	4.095838916	1.14E-33	1.84E-32
HIST1H4PS1	0.070470797	0.339651082	2.268956017	2.31E-13	5.45E-13
AC138969.1	0.069035446	0.247351472	1.841153277	1.67E-09	3.12E-09
TMEM121B	0.09166189	0.258732155	1.49706543	1.55E-23	7.46E-23
ATP5S	5.713454486	2.821232472	-1.018037746	4.10E-30	3.71E-29
IGLV1-51	14.28230035	57.20029646	2.001794261	8.59E-08	1.44E-07

Table S1. The differentially expressed genes(DEGs) were screened out in TCGA

ID	conMean	treatMean	logFC	pValue	FDR
MATN1-AS1	0.194796772	0.520804462	1.418772038	4.29E-22	1.85E-21
KLHDC8A	0.262609357	0.836617986	1.671650694	1.59E-13	3.79E-13
AL049629.1	2.086014158	0.406784251	-2.358413219	2.06E-33	3.17E-32
NFASC	5.810665203	1.877161087	-1.630150873	3.93E-16	1.11E-15
SERPINA4	3.617165533	0.69144386	-2.387175596	6.87E-33	9.58E-32
AC009509.4	0.625893089	1.369603414	1.129770052	3.24E-15	8.62E-15
OR5BA1P	0.029969154	0.388261	3.695476527	4.04E-26	2.43E-25
SALL3	0.65200688	0.14056505	-2.213649258	4.86E-34	8.45E-33
HIF1A	77.61493319	33.72896895	-1.202346037	6.60E-24	3.27E-23
AL121672.1	0.502227056	0.220846509	-1.185295726	4.91E-23	2.28E-22
AC010501.1	1.006588373	0.185455042	-2.440332446	6.04E-39	4.95E-37
RPL10P1	0.089376659	0.239800437	1.42386427	1.49E-14	3.79E-14
AC073610.1	0.121997644	0.47124084	1.949611285	5.83E-26	3.45E-25
ANKRD61	0.154828466	0.38341638	1.308241232	4.88E-13	1.13E-12
IL7R	2.286850316	5.217229036	1.189921831	6.31E-14	1.54E-13
CCRL2	0.87396559	2.426985188	1.47351692	3.50E-29	2.84E-28
GARS-DT	0.361001413	1.171895885	1.698768014	2.32E-24	1.20E-23
AL645939.1	0.024869746	0.543143741	4.448870369	4.96E-35	1.09E-33
SIGLEC9	0.429428	2.155460318	2.327507835	5.48E-34	9.46E-33
RUNX3	0.905378113	5.939608304	2.713775459	7.06E-35	1.49E-33
ZNF767P	1.230112125	2.571757121	1.063964576	3.30E-10	6.41E-10
RNASE6	6.939145097	20.2870591	1.547729902	2.05E-24	1.06E-23
LY9	0.087430479	0.379791438	2.118999175	1.31E-25	7.53E-25
SMIM5	7.909648513	0.765626949	-3.368900069	4.86E-37	2.00E-35
RAP1GAP2	6.036854846	2.600048292	-1.215258693	7.30E-27	4.65E-26
LHX1	3.020999611	0.296317473	-3.349810393	1.49E-38	1.05E-36
IGFN1	0.0204823	0.911985952	5.476561984	0.003403868	0.004207676
RNU7-47P	0.161939031	0.485339599	1.583543828	1.10E-06	1.72E-06
CASP17P	0.239359198	1.632083566	2.769465778	1.47E-37	7.33E-36
CKMT1B	0.541732671	0.24504801	-1.14451667	1.98E-29	1.65E-28
SAP30	2.616934819	14.65200164	2.485147871	1.83E-38	1.26E-36
Z94721.1	0.119882273	0.866926493	2.854291324	7.31E-29	5.71E-28
RRM2	0.665280767	2.89982108	2.123928657	2.81E-32	3.49E-31
WDR90	1.133551067	2.924645672	1.367412463	5.45E-27	3.51E-26
AC104316.2	0.035198692	0.513354308	3.866361176	6.29E-28	4.43E-27
CTSZ	69.80638611	165.7212512	1.247327688	7.54E-31	7.51E-30
GRAMD1B	3.175829943	1.391293907	-1.190706444	6.55E-11	1.33E-10
TRBV14	0.083606474	0.464931186	2.475330631	8.50E-17	2.50E-16
ADAMDEC1	0.134989204	2.665691681	4.303593988	7.61E-32	8.78E-31
AC103724.4	0.057850022	0.25321157	2.129953906	1.07E-18	3.57E-18
HIST1H4E	0.262708031	3.556245817	3.75882284	2.09E-07	3.41E-07
FKBP1AP1	0.09252248	0.217658267	1.234188979	0.000208944	0.00028131
AP001636.3	0.054284778	0.236897369	2.125642559	3.60E-30	3.29E-29
RERG-IT1	0.231745942	1.159428306	2.322797634	9.08E-16	2.50E-15
KRT7	35.15174872	10.83917463	-1.697341558	1.11E-31	1.24E-30
KLHDC7A	19.75846771	9.858132312	-1.003084819	8.39E-20	3.05E-19
DOCK2	0.635168029	3.143769683	2.307285326	1.22E-29	1.04E-28
SNORA65	1.180308515	3.072045445	1.38003555	2.12E-07	3.47E-07
LRRC17	1.137970015	3.561319569	1.645949355	3.91E-21	1.57E-20
RNU6-343P	0.303801844	1.064779323	1.809351931	2.53E-12	5.61E-12
ZBED5-AS1	10.73954844	5.094726197	-1.075856815	2.76E-33	4.14E-32
AC005519.1	0.195832194	0.735491067	1.909089863	9.31E-14	2.25E-13
TF	0.247983323	4.357376627	4.135144811	1.88E-06	2.89E-06
AL139246.3	0.304542784	0.678555268	1.155821407	1.64E-10	3.24E-10
APOL6	5.881236125	12.52485652	1.090602757	4.66E-24	2.34E-23

Table S1. The differentially expressed genes(DEGs) were screened out in TCGA

ID	conMean	treatMean	logFC	pValue	FDR
SLC16A1	4.252015376	18.25550476	2.102112841	1.06E-35	2.77E-34
AC025178.1	0.083500441	0.343700725	2.04129718	2.56E-20	9.64E-20
AGFG2	10.2761841	4.429681081	-1.214029902	7.10E-10	1.35E-09
DDX43P3	0.011118173	0.380032373	5.095130724	4.79E-24	2.40E-23
MIR509-3	0.045156225	0.472379984	3.386951048	1.10E-13	2.66E-13
WIPF1	4.435878472	12.01389424	1.437412123	8.38E-34	1.40E-32
PCDHB9	0.37329261	1.120707173	1.586030515	2.59E-23	1.23E-22
SLC16A12	46.06132819	21.61515644	-1.091512734	2.47E-19	8.68E-19
PPDPFL	0.685562165	4.780564735	2.801821659	5.24E-20	1.94E-19
APBB3	1.563731728	4.423714624	1.500265293	6.37E-22	2.71E-21
C10orf95	0.554663892	0.216551339	-1.356904715	1.91E-20	7.29E-20
SLC17A1	34.18064074	16.45820176	-1.054372727	0.004470638	0.005474773
LINC02038	2.406519517	0.232967204	-3.368749347	5.50E-41	1.40E-38
RNU4-21P	0.03179485	0.259275398	3.027620319	1.47E-08	2.58E-08
AC004801.2	0.121958301	0.356325065	1.546806014	6.66E-12	1.44E-11
PI4KAP1	0.359875067	1.054419324	1.550880659	1.90E-11	3.99E-11
RPL5P24	0.11315026	0.228579284	1.014454756	3.86E-08	6.62E-08
SKA3	0.189742325	0.803104476	2.08154615	4.54E-35	1.01E-33
SLA	1.537339118	5.434049576	1.821592287	3.15E-27	2.08E-26
HPX	0.048201714	0.775773465	4.008479067	1.25E-23	6.11E-23
NME3	13.35129682	26.83313767	1.007035884	1.02E-21	4.27E-21
CD7	0.477295232	3.960605369	3.052767131	1.00E-31	1.13E-30
SLC1A4	1.440338522	5.588999879	1.956182216	2.03E-38	1.37E-36
TBC1D10C	0.398918212	2.37154956	2.571665124	2.81E-33	4.20E-32
ANKHD1	0.361342331	0.745301581	1.044458046	8.25E-30	7.16E-29
RN7SL172P	0.042553106	0.329834557	2.954406201	4.48E-19	1.54E-18
AL137145.1	0.239620001	0.530893049	1.14767292	3.15E-09	5.78E-09
GAS2L3	0.7374928	6.660265313	3.174878779	3.49E-36	1.03E-34
IGKV3-7	0.517787288	1.660191768	1.680918443	2.58E-06	3.94E-06
HYPK	0.161296591	0.438972842	1.444415742	4.74E-17	1.42E-16
MIR4685	0.079473606	0.329100863	2.049982106	9.15E-07	1.44E-06
AC022126.1	0.090916625	0.214764547	1.240139816	5.12E-11	1.04E-10
PCGF1	2.875081167	5.915278406	1.040843386	8.31E-39	6.46E-37
BMX	0.376925734	1.104408092	1.550921162	3.29E-19	1.14E-18
CCL3L1	0.782775898	2.583180855	1.722477412	1.35E-10	2.68E-10
AP003071.4	1.116453966	0.169823319	-2.716817289	1.67E-33	2.61E-32
LINC01614	0.107100236	0.735019609	2.778821081	1.51E-22	6.75E-22
ARHGAP9	0.499732457	3.005668544	2.588458095	1.97E-35	4.77E-34
ZRANB2-AS1	0.426401815	0.181392954	-1.233095159	2.93E-19	1.02E-18
ASMTL-AS1	0.500563539	3.275899338	2.710265911	2.21E-15	5.95E-15
SMIM22	3.495290079	0.980954555	-1.833153985	3.69E-33	5.42E-32
AL691432.1	0.263678383	0.685244196	1.377838904	5.94E-12	1.29E-11
AC006017.1	0.165420176	0.462988477	1.484841081	7.41E-09	1.33E-08
IGKV1-12	0.736419297	1.767910023	1.263445516	0.000522011	0.000684054
RPL7AP10	0.10419246	0.323257893	1.633434714	6.90E-10	1.31E-09
Z98884.2	0.493666825	1.243942034	1.333309657	1.53E-21	6.32E-21
AC018638.3	0.148146667	0.403336523	1.444957885	0.000548066	0.000717192
RNU6-593P	0.060170333	0.282567474	2.23147117	0.000412221	0.000543712
GUSBP11	0.13302359	0.312179051	1.230691619	2.32E-10	4.54E-10
L1CAM	21.7755553	2.402221388	-3.180268489	2.76E-36	8.44E-35
AC127024.4	0.250425028	1.055792991	2.075876332	3.21E-17	9.74E-17
SIGLEC7	0.3954582	1.735314715	2.133600216	8.91E-28	6.17E-27
AC008897.1	0.291317218	0.872613272	1.582751442	2.95E-17	8.99E-17
AC239800.2	0.045557701	0.225144471	2.305084187	8.93E-10	1.69E-09
UGT3A2	1.079251286	0.441517634	-1.289487848	1.30E-32	1.74E-31

Table S1. The differentially expressed genes(DEGs) were screened out in TCGA

ID	conMean	treatMean	logFC	pValue	FDR
JAKMIP1	0.046943229	0.577018503	3.619628589	4.44E-34	7.83E-33
ZNF44	6.800551958	2.746508964	-1.308052845	1.76E-33	2.75E-32
IGKV1-27	3.305946873	12.32980267	1.899014265	8.85E-05	0.000122034
P2RY1	0.436379634	2.44693325	2.48731907	1.46E-34	2.86E-33
FYB2	2.458147796	0.532220499	-2.207475676	1.90E-35	4.63E-34
IGSF11	1.535052745	0.219585815	-2.805431463	6.03E-31	6.08E-30
AP001021.1	0.089276812	0.256122391	1.520475972	1.79E-08	3.14E-08
MILR1	0.7388053	2.976665446	2.010430962	1.31E-30	1.26E-29
KIFC2	1.132188507	2.672005039	1.238808545	3.40E-12	7.49E-12
LINC00645	7.724902255	0.404416369	-4.255603382	4.19E-39	3.67E-37
AC111000.5	0.102159456	0.651808054	2.673624429	2.65E-23	1.25E-22
RN7SL587P	0.055205498	0.261264268	2.242625972	0.000453032	0.000595861
HMGN1P13	0.034014298	0.633976739	4.220216704	8.30E-22	3.51E-21
MIR3186	0.200235478	0.558325037	1.479407637	1.62E-05	2.34E-05
AC019080.1	5.948519454	0.532585864	-3.481444594	2.09E-16	6.01E-16
NRXN3	0.324220056	0.840848319	1.374872239	2.92E-10	5.68E-10
RN7SL674P	0.057858105	1.300918548	4.490867764	0.030449607	0.034940169
MT1DP	1.108845122	2.42524997	1.129075582	1.14E-05	1.67E-05
ODAM	0.974284077	0.116593312	-3.062857451	3.60E-50	4.64E-47
AC018638.7	0.248297049	0.730513764	1.556844342	1.12E-12	2.54E-12
ULBP2	0.562564232	1.14788055	1.028882787	9.77E-17	2.87E-16
AC026412.3	0.159989171	0.501250074	1.647556292	6.40E-25	3.46E-24
BTNL9	2.535815522	11.89983962	2.23042043	2.96E-20	1.11E-19
ZNF385D	0.881023946	0.355640471	-1.308761721	1.25E-23	6.07E-23
UNC5B	5.123127689	17.84339698	1.800293543	4.23E-28	3.04E-27
SNORD62B	0.619861683	1.891764494	1.609714267	4.96E-14	1.22E-13
CDKN1C	25.72729126	9.240980666	-1.477181703	5.04E-27	3.26E-26
AC130650.2	0.128327451	0.276841578	1.109230817	1.01E-08	1.80E-08
AC087623.1	0.184244466	0.670013843	1.862569616	1.27E-12	2.86E-12
ICAM1	11.38388336	29.8352402	1.390024607	7.81E-25	4.20E-24
AC117383.1	0.061232478	0.364532804	2.573679677	1.73E-20	6.64E-20
LINC002481	0.106715727	0.303743815	1.509082229	5.79E-21	2.29E-20
AC015977.2	0.014170638	0.837433691	5.884998393	6.48E-29	5.11E-28
NCAPH	0.261502444	0.994792243	1.927570827	3.90E-34	6.96E-33
UHRF1	0.175342857	0.999957169	2.511687642	2.35E-34	4.35E-33
STRA8	0.277139073	2.941844627	3.408039022	1.32E-30	1.27E-29
HLA-DPA3	0.023220086	0.31058697	3.741552063	1.57E-22	6.99E-22
IL10RB-DT	0.316998606	0.70279179	1.148620844	5.26E-26	3.13E-25
TRAV8-6	0.15330211	0.827524867	2.43242511	1.64E-19	5.83E-19
HMGB1P14	0.139750989	0.280183118	1.003511537	0.032414653	0.037099893
RNY4P10	0.552204486	2.401061084	2.120397593	3.72E-18	1.20E-17
OCLNP1	0.144169822	0.32704843	1.181735082	0.021717554	0.025243959
GPR19	0.049171793	0.227858184	2.212233335	3.74E-33	5.50E-32
EYA4	2.782415578	0.246837565	-3.49470404	1.95E-38	1.32E-36
SLC26A7	16.02201235	3.761266146	-2.09076506	9.26E-37	3.45E-35
TRBC1	0.107523008	0.793897945	2.884308154	5.32E-31	5.40E-30
FCRL5	0.055348842	0.254621552	2.201729486	4.72E-08	8.04E-08
RNU6-476P	0.154216283	0.405244081	1.393836015	1.41E-06	2.18E-06
AL359881.2	0.096125846	0.271196255	1.496340957	1.05E-06	1.64E-06
PTGDR	0.125898207	0.600976753	2.255051445	7.67E-30	6.68E-29
TRAJ1	0.021891544	0.437100626	4.319519799	9.74E-13	2.22E-12
LGALS4	1.349302087	16.59473613	3.620440403	0.00388106	0.004774906
AL031186.1	0.224111997	0.606647044	1.436637502	1.47E-11	3.10E-11
MTMR10	15.08177143	6.755550631	-1.15866062	6.38E-37	2.50E-35
AL139289.1	0.164707018	0.465144431	1.49777673	3.35E-14	8.34E-14

Table S1. The differentially expressed genes(DEGs) were screened out in TCGA

ID	conMean	treatMean	logFC	pValue	FDR
LINC02427	0.45483795	0.172556827	-1.398281083	2.88E-16	8.20E-16
EHD2	19.15319929	90.67564636	2.243129729	3.22E-33	4.78E-32
AC036108.3	0.124982375	0.278068321	1.153714731	4.21E-18	1.35E-17
SIRLNT	1.948538931	0.046489317	-5.389349724	7.57E-49	8.20E-46
C3orf85	1.672702609	0.173523796	-3.268975544	3.10E-17	9.42E-17
AP001024.1	0.273213398	0.663310398	1.279655908	3.00E-10	5.84E-10
RGS5	29.31606369	179.5032984	2.614247045	4.61E-28	3.30E-27
AC025857.2	0.766397057	2.93842087	1.938877123	2.30E-20	8.70E-20
ELF3	21.53043453	9.825140282	-1.131827525	1.93E-11	4.05E-11
AL049552.1	0.211944165	0.704279811	1.732464478	2.94E-14	7.34E-14
PTPN3	13.43100863	5.646858894	-1.250047164	6.86E-34	1.16E-32
AL022341.1	0.23863542	0.484837946	1.022694419	1.11E-07	1.85E-07
KCNMB2	1.154601965	0.262183158	-2.138748666	5.87E-33	8.29E-32
BTN3A3	3.689437306	10.43176266	1.499510245	6.23E-31	6.26E-30
AC091849.2	0.138060852	0.622669615	2.173162591	1.40E-18	4.66E-18
CD247	0.498860564	3.068987214	2.621054103	1.52E-34	2.95E-33
UBAC2-AS1	0.894845754	0.388974433	-1.201963693	1.83E-31	1.99E-30
SCIN	23.36804397	5.544212033	-2.07548264	2.39E-32	3.03E-31
ORAOV1P1	0.417128785	2.602291587	2.641217848	2.21E-25	1.24E-24
CD99P1	0.380865357	1.041995553	1.451996148	3.68E-30	3.36E-29
AL158212.3	2.681801743	1.084576163	-1.306071219	6.65E-29	5.23E-28
CNTFR	1.18102445	0.46929796	-1.331462739	1.14E-20	4.43E-20
AC079140.2	0.38340225	0.909391549	1.246042795	5.14E-10	9.86E-10
IGKV1OR2-1(	0.522034729	3.167538683	2.601144546	0.001096191	0.001405867
LINC01020	2.262612016	0.028810844	-6.295233491	4.22E-32	5.09E-31
HTR1F	0.217808437	0.67250603	1.626487367	8.96E-15	2.31E-14
IGLV3-1	10.99084213	38.42420146	1.805713347	5.28E-07	8.42E-07
DHRS4-AS1	14.17352183	6.918501356	-1.034666814	4.02E-29	3.23E-28
CST2	0.063156238	0.477178146	2.917530837	2.21E-09	4.10E-09
MAPK11	2.818133111	6.46832241	1.198651834	1.79E-26	1.10E-25
AL354811.1	0.233232463	0.488324448	1.066071404	3.46E-14	8.61E-14
PRAP1	40.49086025	18.0439105	-1.166084259	0.004896781	0.005977406
PLXNB1	19.20313639	7.242743757	-1.406733721	5.13E-36	1.46E-34
SEMA3F-AS1	0.254400602	0.54769314	1.106265726	2.61E-11	5.42E-11
BLNK	9.552324958	4.287332507	-1.155771605	2.01E-29	1.67E-28
SERPINF1	9.420696061	19.11711542	1.020959287	8.14E-06	1.20E-05
IGLV10-54	1.894357916	7.256751497	1.93761493	9.55E-05	0.000131496
TAS2R15P	0.177666165	0.406211449	1.193061944	1.59E-05	2.31E-05
OCIAD1-AS1	0.353832424	0.880055543	1.314528322	5.72E-15	1.49E-14
RBCK1	14.29818396	32.08218256	1.165940373	1.22E-35	3.13E-34
MND1	0.252720219	0.72236309	1.515183086	2.45E-28	1.81E-27
HS1BP3-IT1	0.413624005	1.166090217	1.495287589	4.75E-10	9.13E-10
COL6A2	23.43663035	90.9595535	1.956460014	6.67E-28	4.69E-27
AC090607.2	0.271769295	0.834130505	1.617890655	5.30E-16	1.48E-15
MIR1254-1	0.709766756	2.271262653	1.678077644	1.39E-12	3.13E-12
ARHGEF3	11.06614122	5.411104612	-1.032157202	3.53E-32	4.32E-31
IGFBP2	25.08906847	7.10265177	-1.820629247	1.56E-33	2.46E-32
POLG2	1.29673565	2.882698611	1.152535605	2.01E-26	1.23E-25
HILPDA	4.372160292	112.6098692	4.686843168	6.58E-39	5.27E-37
AC055713.1	0.153333498	0.396365769	1.370159465	4.88E-11	9.96E-11
NEK2	0.248114188	0.848628824	1.774129445	1.32E-29	1.13E-28
PADI1	0.062343248	3.396826756	5.76781051	2.91E-30	2.68E-29
MIR513C	0.016679322	0.376634347	4.497032003	3.47E-13	8.13E-13
ZMAT1	1.833548493	4.176359505	1.187607483	1.42E-11	3.00E-11
RNASE2	0.376079281	2.019287882	2.424737872	1.50E-26	9.35E-26

Table S1. The differentially expressed genes(DEGs) were screened out in TCGA

ID	conMean	treatMean	logFC	pValue	FDR
SLC22A15	2.219463849	0.87531387	-1.342338873	1.35E-29	1.15E-28
LINC01571	3.224633306	0.024914233	-7.016021123	1.75E-48	1.76E-45
ITM2C	141.0551547	53.86792114	-1.388761094	1.21E-32	1.63E-31
OSTM1-AS1	0.004165584	2.009911219	8.914397199	6.74E-37	2.61E-35
TRPM2	0.35163474	2.409790999	2.776758511	3.05E-37	1.35E-35
PSMD10P1	0.096606309	0.473264538	2.292457517	1.91E-14	4.82E-14
TUBA1B	28.00522028	59.12063956	1.0779661	1.83E-26	1.13E-25
LINC01126	0.137911907	0.52035866	1.91575933	1.16E-19	4.17E-19
AL731563.3	0.124384739	0.331001314	1.412027452	2.65E-20	9.96E-20
UBE2Q2P1	0.277760907	0.651628353	1.230205815	7.76E-13	1.78E-12
ENPP6	6.668230133	0.221967216	-4.908885381	4.54E-35	1.01E-33
AL359715.3	1.319978869	0.555996093	-1.247368185	4.50E-33	6.52E-32
SAMHD1	9.371830194	25.43711337	1.440532242	1.28E-25	7.37E-25
AMPH	1.783234529	0.198144619	-3.169870765	6.58E-39	5.27E-37
SNORA38B	0.074171202	1.249112669	4.073900647	2.67E-06	4.07E-06
PIK3CG	0.760539903	1.849939691	1.282382391	1.84E-14	4.65E-14
EEF1DP4	0.127419005	0.310377832	1.284445044	0.008717613	0.010450578
AL353625.1	0.32598156	0.743597628	1.189731812	4.95E-17	1.48E-16
AC119044.1	0.159306454	0.91761334	2.526081651	1.10E-32	1.48E-31
SPOCK2	40.85220172	20.04741825	-1.026997379	3.93E-17	1.18E-16
ZP1	0.028479287	0.332307903	3.544535695	8.03E-27	5.11E-26
CTSL3P	0.408633627	3.27304904	3.001755385	1.24E-20	4.80E-20
MROH2A	0.055007389	0.440000778	2.999808736	0.001697095	0.002146707
PSMB10	7.361736236	18.93288573	1.362776357	4.98E-30	4.46E-29
MIR5690	0.119263054	1.897629911	3.991979563	2.08E-09	3.87E-09
CENPI	0.125928687	0.385372228	1.61364563	3.97E-30	3.60E-29
TMSB10P1	0.292893075	1.341456525	2.19535431	1.42E-24	7.49E-24
MAP7D2	1.6170332	8.557678396	2.403870163	2.77E-10	5.41E-10
FCHO1	0.184007747	1.127399557	2.615160491	5.86E-32	6.92E-31
IL10	0.200087059	0.50553959	1.337196218	3.56E-12	7.82E-12
AC010261.1	0.041590909	0.332178171	2.997617159	3.21E-17	9.73E-17
E2F2	0.079670582	0.409862349	2.363020441	3.67E-34	6.58E-33
CETN4P	0.88187175	0.241878598	-1.866285739	4.83E-32	5.76E-31
TLR3	5.539796903	16.01348697	1.531382501	3.44E-24	1.74E-23
CHIT1	0.077507872	2.431162175	4.971159477	1.23E-21	5.10E-21
SNORA71C	0.189753187	1.043018488	2.45856871	5.12E-17	1.53E-16
FAM219A	4.706011333	10.03365622	1.092270713	2.51E-35	5.90E-34
AC068338.3	1.486563329	0.686806209	-1.114005938	4.24E-24	2.14E-23
HLA-T	0.078138046	0.226968832	1.538397116	2.40E-12	5.33E-12
CTLA4	0.114287353	0.907691279	2.98953593	1.86E-27	1.25E-26
AC073257.2	0.125382719	1.172503457	3.225181755	1.84E-33	2.85E-32
AC138123.1	0.906416282	0.204915971	-2.145141348	1.41E-32	1.88E-31
MOB3A	9.203178167	19.64100457	1.093664657	2.84E-32	3.51E-31
LINC01004	0.648160246	1.718184903	1.406462859	7.52E-11	1.52E-10
TSPYL4	16.26668456	7.945529237	-1.03370501	1.44E-34	2.82E-33
SNORA12	0.186623819	4.971950714	4.735606961	2.09E-07	3.42E-07
FPR1	1.842032599	5.525870812	1.584903241	4.13E-17	1.24E-16
MEF2B	0.067171471	0.280599977	2.062594367	6.34E-28	4.46E-27
DLL4	3.828581707	20.8556227	2.445554437	7.87E-32	9.04E-31
PRR22	0.469087036	1.28305087	1.451650835	1.53E-18	5.07E-18
PTGR1	51.32649625	20.62718435	-1.315156876	2.38E-33	3.62E-32
AC073850.1	0.062646858	0.642528592	3.358446603	3.04E-19	1.06E-18
AC078942.1	0.095735213	0.237621822	1.311545748	0.000887889	0.001146504
EPCAM	147.6145346	19.71968476	-2.904126387	5.95E-40	8.16E-38
EBLN2	0.183402937	0.463147692	1.336455584	5.42E-13	1.25E-12

Table S1. The differentially expressed genes(DEGs) were screened out in TCGA

ID	conMean	treatMean	logFC	pValue	FDR
ORM1	0.132598528	3.962466698	4.90126214	0.000614203	0.000800583
AL139161.1	0.150467577	0.311781837	1.051084239	4.32E-09	7.86E-09
C15orf48	1.413460164	4.141319402	1.550859252	1.65E-15	4.48E-15
AC243960.1	0.190792211	0.905843615	2.247259733	2.19E-28	1.63E-27
MSC	2.398311154	19.57436037	3.028874416	2.35E-32	2.98E-31
AKT3-IT1	0.039740841	0.228090257	2.520910503	1.27E-09	2.39E-09
NLGN1-AS1	0.003573108	0.297364789	6.378910362	3.51E-17	1.06E-16
IQCH-AS1	1.797153022	0.884828431	-1.022243608	3.25E-33	4.81E-32
MYRFL	0.222257071	0.881603362	1.987900408	9.54E-09	1.70E-08
ARHGAP11A	0.444274358	1.166353045	1.392481764	8.88E-30	7.68E-29
UBASH3A	0.154910564	1.108511247	2.839115972	1.32E-30	1.27E-29
GBGT1	1.353878774	2.727191009	1.010317183	2.06E-21	8.47E-21
KCNJ16	83.59537583	28.69949732	-1.542397673	2.60E-41	7.55E-39
AL442128.2	0.096116367	0.22602998	1.233660112	3.08E-08	5.31E-08
STAC3	0.440972936	2.161548379	2.293303105	6.65E-39	5.30E-37
AC124312.2	2.647263175	1.186153641	-1.158210734	6.40E-25	3.46E-24
LAMC2	9.558824882	3.190328548	-1.583128267	3.19E-22	1.39E-21
KLRD1	0.084454012	0.438419828	2.37607518	1.76E-36	5.78E-35
PNMA2	2.686043901	15.43642934	2.522784286	6.08E-31	6.13E-30
PDZRN3	3.83829972	1.742249163	-1.139516409	3.00E-26	1.83E-25
FBXO41	0.301115941	1.192639593	1.985767144	3.00E-18	9.74E-18
NUS1P2	1.846026783	7.430384381	2.00901336	3.15E-05	4.48E-05
CELF2	1.958342142	3.957759983	1.015051283	8.26E-17	2.43E-16
TAS2R4	0.10803253	0.315483386	1.54609824	1.02E-13	2.47E-13
RASGRP4	0.268728011	0.926070755	1.784975716	7.29E-31	7.27E-30
PTPN7	0.664340028	2.307957304	1.796622786	1.91E-19	6.76E-19
PLCXD2	2.924746104	0.994981408	-1.555569917	1.51E-30	1.44E-29
FAM3D-AS1	0.813637887	0.147351838	-2.465121806	1.77E-26	1.09E-25
AC135983.2	0.362519957	0.993357709	1.454252909	6.32E-21	2.49E-20
ST6GALNAC	3.575632299	1.623709044	-1.138905249	1.72E-12	3.86E-12
AC025165.4	0.21262203	0.47163318	1.149374137	1.62E-11	3.42E-11
TAS2R5	0.171041228	0.676939957	1.984683757	1.11E-23	5.42E-23
OR2I1P	2.471635189	36.05138876	3.866517112	1.33E-26	8.30E-26
FRK	7.056410417	2.95226508	-1.257112209	2.32E-35	5.49E-34
AMT	7.996424444	3.817660885	-1.066666095	2.89E-28	2.12E-27
RPS6KA6	5.018454776	0.633164016	-2.986592043	2.14E-40	3.76E-38
AP001363.1	0.087840368	0.533056	2.601331108	1.60E-16	4.62E-16
RASSF2	2.203617176	10.21528677	2.212784186	2.59E-34	4.76E-33
IGKV1D-13	0.561049656	1.47929134	1.398705846	0.004004301	0.004920355
CKMT1A	0.518990773	0.235902842	-1.137516087	6.11E-28	4.31E-27
ENO1-AS1	0.535005874	0.155833734	-1.779547159	1.85E-20	7.09E-20
DNAJC11	16.01264797	6.444798671	-1.313004706	9.23E-32	1.05E-30
BCL2L15	1.031817271	0.265193145	-1.960072109	4.81E-14	1.18E-13
AL117328.1	0.143187946	0.422381336	1.560636036	2.10E-14	5.30E-14
TOM1L2	16.71482408	8.178493791	-1.031221095	1.59E-33	2.49E-32
ARHGAP33	0.533823811	2.370905437	2.15100256	1.93E-24	1.00E-23
KCNJ2	1.090567771	3.974674312	1.865757224	1.31E-24	6.92E-24
SEMA5B	2.082418883	27.6203172	3.729397684	2.77E-32	3.44E-31
FYB1	2.609953703	8.63751124	1.726591467	3.92E-22	1.70E-21
AC027702.1	0.290416508	0.932654071	1.683218609	3.25E-27	2.14E-26
RPS2P5	17.5769104	39.40909676	1.164847182	5.32E-22	2.28E-21
YTHDF3-AS1	1.567280154	0.761401555	-1.041533665	3.32E-22	1.45E-21
FOXD4	0.173076877	0.381926712	1.141882832	2.31E-10	4.52E-10
ALDH1L2	0.352507251	0.878311024	1.317078976	4.36E-12	9.51E-12
TRAF1	1.992486882	4.009239513	1.008758381	9.35E-15	2.41E-14

Table S1. The differentially expressed genes(DEGs) were screened out in TCGA

ID	conMean	treatMean	logFC	pValue	FDR
AC106772.2	2.733021082	0.080478581	-5.085747917	2.42E-46	1.78E-43
RNU6-190P	0.07713921	0.40188139	2.381233502	3.07E-10	5.97E-10
NPIPA5	0.33014369	0.805377134	1.28657044	4.16E-07	6.68E-07
C4orf47	0.914447017	5.236238429	2.517559304	5.52E-34	9.52E-33
AC084880.1	1.472826144	7.585566004	2.364669691	5.54E-29	4.39E-28
TRAV9-2	0.147797347	0.720451599	2.285281142	4.12E-18	1.33E-17
AC090114.3	0.531068977	1.170088554	1.139646559	7.73E-10	1.47E-09
SLC28A1	11.12268132	44.0771582	1.986526594	1.97E-24	1.02E-23
F8	5.030398847	10.52304579	1.064807641	3.12E-05	4.44E-05
AL031846.1	0.032092918	0.427136159	3.734369168	8.01E-34	1.34E-32
CPNE5	0.734589456	2.583561551	1.814351162	7.72E-23	3.52E-22
CNTN1	5.280299988	0.422711713	-3.6428739	6.24E-39	5.08E-37
L3MBTL1	0.329488467	0.685751623	1.057458165	6.57E-08	1.11E-07
CFAP46	0.410921681	0.200888697	-1.032467059	1.84E-21	7.57E-21
LINC00511	0.158663073	0.411295962	1.374210514	1.02E-07	1.70E-07
GPNMB	16.99379919	55.02679707	1.695125934	5.40E-07	8.60E-07
HERC2P2	0.77392344	2.547209883	1.718655077	1.30E-12	2.93E-12
FDX1	16.71874568	7.663216499	-1.125444644	9.64E-38	5.14E-36
AC022558.1	0.12288348	0.3631507	1.563277379	1.47E-12	3.30E-12
GPR174	0.23297248	1.358328198	2.543600652	3.02E-21	1.22E-20
MTDHP3	1.905147479	0.553665066	-1.782817281	4.83E-25	2.64E-24
SDC3	8.28777425	17.34777311	1.065693868	1.44E-23	6.97E-23
ARL6IP4	0.273682172	0.854984085	1.643396106	1.66E-23	8.00E-23
ALX1	1.673991251	0.188210374	-3.152873929	1.09E-30	1.06E-29
LONRF2	1.437745035	0.395287688	-1.862832931	3.98E-31	4.11E-30
LINC00921	0.279117056	0.779767616	1.482173956	1.09E-30	1.06E-29
LINC01896	1.292712161	0.279952006	-2.207149655	1.22E-36	4.32E-35
UBE2L4	0.084354607	0.223487783	1.405657199	3.29E-05	4.66E-05
NLRP1	1.135399042	2.674270392	1.235945913	1.91E-20	7.29E-20
HSPB6	14.21254867	6.921540461	-1.038000224	4.06E-14	1.00E-13
AC098851.1	0.254308089	0.516757127	1.022909027	9.91E-05	0.000136329
Z96811.1	0.072274722	0.299639462	2.051664571	3.20E-11	6.62E-11
RNASET2	3.348983639	33.49208626	3.322025013	5.76E-36	1.62E-34
TMPRSS4	3.195945704	0.2410174	-3.729033687	6.20E-38	3.55E-36
FCGR3B	0.343330288	0.977381146	1.509324139	5.29E-07	8.44E-07
RNU6-117P	0.477739751	1.888190579	1.982707557	2.52E-09	4.65E-09
HLA-DOA	8.590774556	23.0765818	1.425569424	3.07E-14	7.66E-14
GAPDHP40	0.173724772	0.447476711	1.365009115	2.67E-21	1.09E-20
AC055855.1	0.133914978	0.511256362	1.932729559	9.50E-19	3.20E-18
PABPC4L	0.880310667	2.17809448	1.306981882	3.91E-16	1.10E-15
PON3	0.716357046	0.30437273	-1.234839726	1.38E-21	5.72E-21
FOXI1	20.6866531	7.522189967	-1.459475604	6.30E-37	2.48E-35
LINC01772	0.180998189	0.481812198	1.412495656	1.42E-19	5.09E-19
AP000897.2	0.070933636	0.489897744	2.787938848	2.70E-19	9.44E-19
CAT	123.5289486	51.07843203	-1.274063028	2.08E-33	3.19E-32
MIR6793	0.055139079	0.396389091	2.84577018	1.82E-10	3.60E-10
AC008440.1	0.084400214	0.263188539	1.640778112	3.31E-10	6.43E-10
ZFHX2	0.905388688	0.27375043	-1.72567605	1.20E-37	6.14E-36
XAF1	0.609519072	2.512464032	2.043359673	3.12E-25	1.73E-24
CPT1B	0.184735431	0.680381837	1.880884035	2.94E-10	5.73E-10
RNU1-103P	0.184997814	0.697977604	1.915672524	9.12E-12	1.95E-11
RNU6-577P	0.153842471	0.325164483	1.079715846	0.021411071	0.024889116
AC007496.1	0.012437263	0.23401342	4.233850386	1.17E-13	2.82E-13
ACSF2	25.56426811	2.721778388	-3.231507213	4.86E-30	4.36E-29
RPL10P6	3.010269059	10.44209538	1.794446894	4.05E-15	1.07E-14

Table S1. The differentially expressed genes(DEGs) were screened out in TCGA

ID	conMean	treatMean	logFC	pValue	FDR
BRD9P2	0.107244397	0.23937762	1.158386002	0.007780562	0.009358311
SPDEF	0.069527114	0.433682778	2.640992545	6.35E-18	2.01E-17
PIK3C2G	3.18925628	0.209681767	-3.926946714	2.84E-40	4.56E-38
LRRC36	0.171985925	0.635806182	1.886296539	6.19E-24	3.08E-23
AC010307.4	0.716867117	0.149844047	-2.25824395	2.29E-32	2.91E-31
PLEKHA3P1	0.12334752	0.257884688	1.063997407	4.20E-11	8.62E-11
AC087071.2	0.507167011	0.240650208	-1.075523238	9.85E-21	3.83E-20
LINC01936	1.747031824	0.67050778	-1.38157991	1.88E-27	1.27E-26
GRB10	6.636349833	19.1403704	1.528156904	1.74E-27	1.18E-26
GSTM5	0.856967107	0.35743376	-1.261563926	2.92E-17	8.89E-17
BTN3A1	4.190421903	15.89480638	1.923388032	6.04E-37	2.39E-35
AC107081.2	0.067362323	0.309622893	2.200498356	1.85E-19	6.54E-19
TPM1-AS	0.146790096	0.472097363	1.68532979	1.26E-16	3.66E-16
NLRP6	0.614899793	1.704630251	1.471035615	4.05E-10	7.82E-10
AC116552.1	0.065841347	0.324134164	2.299525334	3.53E-14	8.77E-14
RFTN1	7.390498125	14.94461601	1.015882317	1.64E-22	7.29E-22
ABCB5	0.01011622	0.236498168	4.547086721	0.001749748	0.00221005
AC087257.2	0.045238422	0.230682613	2.35028876	5.37E-15	1.41E-14
DDX39B	3.716356333	11.56706923	1.638062629	1.67E-19	5.93E-19
THBS2	8.586893875	22.59929661	1.396069603	7.09E-07	1.12E-06
AGAP6	0.564174238	1.745870308	1.629733699	8.87E-17	2.61E-16
RNU6-759P	0.041457168	0.5250602	3.662789374	3.46E-09	6.33E-09
POPDC2	1.385426447	4.176575524	1.591990407	3.03E-27	2.00E-26
AC010997.3	0.675294214	0.297919583	-1.180593236	4.23E-26	2.54E-25
ZGRF1	0.161284933	0.40170399	1.316521121	1.03E-24	5.47E-24
RN7SL600P	0.09030702	0.480130983	2.410517986	2.80E-12	6.20E-12
AC113143.1	0.057174157	0.222412576	1.959803277	0.00033073	0.000439306
AC108704.2	0.107932879	1.003006723	3.21612496	2.54E-34	4.68E-33
TRIM14	4.671600971	10.58024683	1.179384329	6.28E-21	2.48E-20
AL512306.2	0.12046523	0.358205831	1.572172021	2.49E-07	4.04E-07
ARG2	37.12929571	9.539183227	-1.960620301	2.69E-27	1.79E-26
SYNE2	14.84510535	6.660405529	-1.156305406	3.15E-24	1.60E-23
RPS15AP38	0.092832743	0.239003772	1.364327733	2.16E-09	4.01E-09
MIR378H	0.383511097	0.939233825	1.292216041	1.96E-05	2.82E-05
AC021744.1	1.716990777	15.52936538	3.177044679	8.18E-19	2.76E-18
RSPH4A	0.495451512	0.246293354	-1.008366172	2.00E-17	6.14E-17
RPS12P16	0.010943952	0.244384028	4.480944311	1.72E-12	3.85E-12
AL109615.3	0.62872963	4.433096658	2.81780316	2.98E-34	5.41E-33
RNU7-40P	0.233198582	1.551485173	2.734018989	8.23E-14	2.00E-13
GGT2	0.138012491	0.414069004	1.58507237	0.00356821	0.004404215
AC098869.2	0.186934628	0.454815231	1.282746725	2.37E-07	3.86E-07
AC084759.3	1.596975883	0.237425552	-2.749795413	6.34E-30	5.60E-29
AC109460.2	0.216084534	0.790538593	1.871240079	7.14E-16	1.98E-15
NCF1	0.251276641	1.252014292	2.316902564	1.34E-31	1.48E-30
UQCRC1	83.75180597	39.0218911	-1.101836604	2.77E-32	3.44E-31
SHISA3	28.26140581	6.635045083	-2.090655058	7.87E-32	9.04E-31
MAP3K12	0.880634292	2.282930324	1.3742719	2.09E-27	1.40E-26
AC023825.2	0.01586742	0.26004914	4.03464477	8.88E-25	4.75E-24
C1orf116	6.549097431	0.887432857	-2.88358622	1.43E-35	3.60E-34
SPINT1-AS1	9.524572819	3.958528225	-1.266690251	1.02E-30	1.00E-29
AC005154.2	0.228774068	0.724188804	1.662442346	1.59E-15	4.31E-15
TRIM2	26.69296478	7.629951613	-1.806713741	2.29E-35	5.46E-34
CLK4	1.822402832	5.295318708	1.538875624	5.82E-30	5.17E-29
RN7SL535P	0.060859746	0.313521606	2.365004656	1.45E-17	4.51E-17
AL627389.1	0.086079593	0.225485377	1.389290711	1.31E-18	4.35E-18

Table S1. The differentially expressed genes(DEGs) were screened out in TCGA

ID	conMean	treatMean	logFC	pValue	FDR
CXCR2P1	0.083254558	2.038551653	4.613871437	8.02E-31	7.95E-30
AC104237.3	5.981177639	0.248642781	-4.588283116	2.08E-45	1.38E-42
AC118344.2	0.197144922	0.535519975	1.441683841	2.50E-11	5.20E-11
LINC00843	0.126403785	0.339213074	1.424152114	3.41E-10	6.61E-10
TCAF2	0.625867418	1.991993867	1.670284227	1.58E-33	2.47E-32
TRGV3	0.136668263	0.466451619	1.771049186	5.45E-18	1.74E-17
DLEU7	0.087213831	0.292946653	1.748009122	6.95E-21	2.73E-20
CXCR3	0.371623525	3.382527768	3.18618804	3.52E-31	3.66E-30
ACAP2-IT1	0.180356552	0.443900917	1.299385855	6.70E-08	1.13E-07
EHF	19.91221951	0.525110237	-5.244889908	1.22E-40	2.46E-38
AC009269.5	0.133608751	0.34311089	1.360660417	9.50E-05	0.000130753
AC245884.10	0.030319894	0.239226194	2.980038757	1.42E-16	4.13E-16
EGLN3	4.107289625	79.23944973	4.269960274	9.17E-36	2.46E-34
ZNF469	0.10559336	0.450659405	2.093518389	7.00E-32	8.15E-31
CCNE2	0.147261585	0.442864299	1.588483561	1.13E-33	1.83E-32
CSPG4P13	0.05881242	0.367797036	2.644717081	6.58E-21	2.59E-20
RPL10P3	0.341681747	1.018850855	1.576217793	1.27E-14	3.24E-14
TUBB3	0.089289797	0.249026016	1.47972923	8.29E-09	1.48E-08
AP007216.2	0.039121196	0.425048567	3.441605311	1.23E-27	8.44E-27
AC139769.1	2.007245099	0.479426569	-2.065835022	9.51E-37	3.52E-35
AL591866.1	0.104986003	0.280555422	1.418088801	1.97E-06	3.02E-06
AC079760.1	0.111734849	0.426652582	1.932982567	2.78E-10	5.43E-10
LINC01055	9.400381382	0.112579448	-6.383703907	2.10E-20	7.97E-20
AC040970.1	0.8662459	2.006772236	1.212028359	1.55E-26	9.63E-26
SNORD53B	0.144463111	0.487298034	1.754103256	6.64E-07	1.05E-06
AC093627.7	2.051749119	0.162971142	-3.654165902	2.65E-32	3.32E-31
PROC	5.261353549	1.422607443	-1.886896381	2.92E-21	1.18E-20
P2RY10	0.409902844	1.636432905	1.997200545	3.36E-19	1.17E-18
TRG-AS1	0.110213554	0.712890984	2.693379825	1.82E-35	4.46E-34
TMEM262	0.099752466	0.298375424	1.580704306	1.61E-24	8.41E-24
TFR2	0.048947392	0.754157325	3.945561616	5.32E-29	4.22E-28
AC091271.1	2.864474608	1.371242915	-1.062786383	6.41E-21	2.53E-20
ST6GALNAC	2.497369397	0.903703096	-1.466488463	1.05E-32	1.42E-31
AC008982.2	0.147223165	0.604220638	2.037070767	1.18E-17	3.67E-17
SQOR	7.979041611	19.25597784	1.271019011	3.77E-37	1.62E-35
EXOC3L1	1.931327681	5.710508641	1.564026292	2.94E-26	1.79E-25
CENPA	0.131554968	0.704125775	2.420167428	4.13E-33	6.03E-32
CLDN8	70.92163653	5.737231068	-3.627799298	2.01E-40	3.58E-38
PRSS8	44.46200105	13.2332334	-1.748407268	1.87E-32	2.42E-31
AL033381.2	0.584899944	1.74070007	1.573405885	1.94E-05	2.80E-05
AC074138.1	0.122540259	0.280014795	1.192247257	2.08E-08	3.62E-08
KNDC1	0.347793145	1.074996977	1.628031201	1.23E-05	1.80E-05
SNHG28	0.773819251	0.313936073	-1.301525808	5.70E-28	4.04E-27
LINC02343	1.984954556	0.084774488	-4.549332002	2.74E-54	6.27E-51
MIR99AHG	1.903442258	0.763131988	-1.3186063	1.95E-31	2.10E-30
FBXO21	21.66202903	10.69090545	-1.018784338	1.42E-31	1.56E-30
GSTM3	60.00433532	5.961968865	-3.331205993	4.21E-41	1.14E-38
AC091133.5	0.138598709	0.360128691	1.37759872	1.79E-10	3.53E-10
PRND	0.17529766	1.657114571	3.240794707	1.02E-17	3.18E-17
RN7SL113P	0.050338546	0.307341317	2.610106278	4.69E-17	1.40E-16
AC010976.2	0.325060491	1.117177548	1.781078362	1.13E-07	1.88E-07
CAPN12	0.563160472	5.200120577	3.206927095	9.63E-32	1.09E-30
NEK6	7.426096165	30.81438683	2.052928181	1.28E-34	2.54E-33
FOLH1B	1.678498696	0.226809007	-2.887621576	1.08E-09	2.03E-09
PAM16	1.464392424	2.988883157	1.029304285	2.38E-25	1.33E-24

Table S1. The differentially expressed genes(DEGs) were screened out in TCGA

ID	conMean	treatMean	logFC	pValue	FDR
TRIM9	0.273619809	2.002291953	2.871407761	1.05E-33	1.71E-32
SLC4A11	13.40310863	0.575731736	-4.5410271	3.70E-37	1.59E-35
AL391280.1	0.09068524	0.234193481	1.368761249	4.27E-17	1.28E-16
MYO15B	3.354788324	10.8815231	1.697586862	2.18E-21	8.93E-21
AL121890.5	0.105799903	0.233856704	1.144286476	1.51E-06	2.34E-06
BSPRY	11.76628496	2.661294087	-2.144459031	2.73E-36	8.38E-35
HADH	52.33342792	15.4898121	-1.756413117	6.35E-40	8.61E-38
PRSS35	4.475548983	0.971317928	-2.204049163	1.25E-27	8.52E-27
AC005534.2	0.12837751	0.332460793	1.372791726	1.17E-08	2.08E-08
AL109809.1	0.026992176	0.261042729	3.273672788	1.49E-33	2.35E-32
OR7E47P	0.535337151	3.257646792	2.605310508	1.34E-24	7.07E-24
AC025766.1	0.122195837	0.545379563	2.158065508	8.07E-18	2.54E-17
TGFBI	31.25246186	219.1229792	2.809699041	4.43E-24	2.23E-23
AC007842.1	0.300455705	0.821949961	1.451898253	2.08E-19	7.34E-19
ADGRA2	4.183515931	10.73574461	1.359634423	3.55E-19	1.23E-18
ADAP2	1.960937588	7.322837378	1.900859139	2.89E-37	1.29E-35
AC148477.2	0.62095348	0.1488674	-2.060457337	9.06E-29	7.01E-28
PTCHD4	0.493627612	1.048110807	1.086296246	6.69E-06	9.94E-06
LINC01801	0.279712795	0.57717388	1.045059763	0.000624417	0.000813381
HLA-DPB2	0.304300758	1.970213627	2.694782234	4.89E-21	1.95E-20
LINC02285	0.100155606	0.365721441	1.868502036	1.37E-27	9.33E-27
AC009690.2	0.17513302	0.475816452	1.44195404	4.45E-18	1.43E-17
AL158152.1	0.072020403	0.22281879	1.629393319	0.000646346	0.000841042
AC127024.6	0.154771665	0.367386644	1.247157803	1.62E-09	3.03E-09
RN7SL417P	0.122975301	0.367531067	1.579497617	0.000237075	0.000318119
GOLGA7B	0.058314108	0.810004702	3.796013417	9.34E-37	3.47E-35
KLHL13	7.601908514	1.672901179	-2.184009438	5.16E-40	7.48E-38
THBS3	2.824244819	6.733152869	1.253417066	3.20E-27	2.11E-26
AC016590.2	0.192845072	0.511099379	1.40616156	1.75E-06	2.70E-06
NAV2	6.161523861	2.043306737	-1.592381406	9.42E-36	2.50E-34
H2AFY2	11.82278849	4.995257029	-1.242939527	4.01E-32	4.85E-31
MEI1	0.208586488	0.542857916	1.379928937	8.83E-13	2.01E-12
AC137932.2	0.071188117	0.368174783	2.370682473	4.18E-32	5.05E-31
FSTL4	1.11280488	0.194508112	-2.516298421	1.27E-34	2.53E-33
LINC01943	0.084571732	0.766338206	3.179733805	4.25E-38	2.60E-36
AC092809.4	0.057033867	0.339361894	2.572933821	9.38E-34	1.54E-32
AL596442.2	0.150026935	1.039194333	2.792172025	9.52E-28	6.57E-27
AL357079.1	0.710026919	1.590742289	1.1637545	1.11E-16	3.26E-16
AC005274.1	0.049091158	0.254390025	2.373507011	3.66E-14	9.10E-14
PABPC1L	0.912772401	4.341193613	2.249764691	2.18E-16	6.26E-16
SNORD14A	0.824523808	3.752927079	2.186383201	1.72E-19	6.11E-19
RNA5SP201	0.124874732	0.419099597	1.746811549	2.23E-08	3.88E-08
SUSD2	14.77576522	5.425145705	-1.445499058	0.000286828	0.000382711
RASSF6	3.222481125	7.79379063	1.274153268	1.76E-21	7.28E-21
AC005332.1	0.054997057	0.306993464	2.480781619	6.54E-21	2.57E-20
RALGDS	3.841135347	8.493245242	1.144783108	2.98E-32	3.68E-31
GSN-AS1	0.099350257	0.238379881	1.262666875	8.11E-05	0.000112143
PTGS2	5.727144401	2.5058454	-1.19251857	2.04E-14	5.15E-14
P2RY8	2.062411426	7.683752404	1.89747887	1.68E-27	1.13E-26
RNU7-186P	0.342620088	0.849128762	1.309373602	0.000206812	0.000278491
DNAJC28	1.549196669	0.711158115	-1.123278044	5.90E-29	4.66E-28
AL161452.1	0.058053673	0.246043353	2.083453281	1.59E-19	5.66E-19
IGHV1-45	0.275815979	1.177627542	2.094105374	0.000269995	0.000360928
TCF4	2.421118306	5.597280726	1.20905253	2.84E-18	9.22E-18
IL2RB	0.763775293	6.259662302	3.034864672	4.52E-37	1.88E-35

Table S1. The differentially expressed genes(DEGs) were screened out in TCGA

ID	conMean	treatMean	logFC	pValue	FDR
AC006033.2	0.1086273	0.542931742	2.321384106	3.91E-33	5.73E-32
ADGRF3	1.530784345	0.130491041	-3.552248384	3.59E-39	3.28E-37
AL451060.1	0.466240498	0.182371872	-1.354191091	6.39E-23	2.94E-22
SCML4	0.10433144	0.269414173	1.368651769	4.17E-15	1.10E-14
BIK	1.812636838	0.694838813	-1.383339661	1.38E-30	1.32E-29
CLMN	9.709497292	3.850642045	-1.334297586	3.39E-35	7.72E-34
RN7SKP22	0.054283025	0.249910879	2.202840694	3.34E-07	5.39E-07
AC138956.1	0.2669516	0.903600354	1.759106639	7.10E-17	2.10E-16
RHBG	5.482258416	1.582699103	-1.792383332	2.40E-36	7.49E-35
AC018541.1	1.588500415	0.146668823	-3.437031325	6.49E-32	7.59E-31
SLC9A3-AS1	1.743309179	14.67071627	3.073038947	8.87E-19	2.99E-18
AL135960.1	0.063127817	0.244164479	1.951505558	1.49E-10	2.95E-10
SLFNL1-AS1	0.079033619	0.361954744	2.195270948	1.53E-22	6.81E-22
TRBV29-1	0.375992816	2.167340172	2.527148604	5.79E-21	2.29E-20
TRAV8-1	0.078945269	0.38536212	2.287290054	1.50E-13	3.58E-13
AC007639.1	0.065388545	0.503103809	2.943746282	5.68E-19	1.94E-18
RPS2	116.2507789	313.8427391	1.43280145	2.30E-36	7.23E-35
RPS27AP10	0.032926683	0.317158388	3.267874425	3.20E-10	6.22E-10
PIK3R5	0.361425004	2.584320304	2.838016668	1.00E-37	5.28E-36
AC018816.1	0.359561465	1.112675037	1.629721988	2.15E-24	1.11E-23
APOC2	0.088198234	0.442778635	2.327763932	8.96E-16	2.47E-15
NNT	29.40260222	12.24080903	-1.264244931	2.96E-33	4.40E-32
ZFPM2-AS1	0.396056096	1.701299423	2.102860385	6.01E-19	2.05E-18
VN1R85P	0.604632137	0.276403918	-1.129279597	2.53E-22	1.11E-21
TM7SF2	11.3594711	5.460770678	-1.056719186	4.95E-29	3.95E-28
LINC02544	0.078981742	0.334781267	2.083627705	7.73E-13	1.77E-12
AP003774.1	0.106766339	0.259899728	1.28349825	4.48E-17	1.34E-16
LINC01235	0.507236878	4.217569472	3.055680289	5.88E-28	4.16E-27
IGHV3-7	0.800938598	2.448886886	1.612362587	0.002168932	0.002718824
AC011374.2	0.65405485	1.516890777	1.213633676	1.09E-21	4.56E-21
AC005730.3	0.060561211	0.248296163	2.035596005	7.69E-10	1.46E-09
AC008750.1	0.018696014	0.310398634	4.05331959	7.45E-30	6.50E-29
AC073335.2	0.866312332	2.634192653	1.604401702	8.71E-18	2.74E-17
FBXO2	12.82711423	2.0297757	-2.659804421	8.60E-34	1.43E-32
HPGD	10.93876474	2.548996387	-2.101448597	1.99E-29	1.66E-28
P4HA1	16.37654519	53.63917231	1.711655938	2.77E-34	5.06E-33
AL353622.1	0.656716151	1.36634076	1.05697549	0.000336192	0.000446261
PAQR4	0.73026084	2.427552069	1.733018466	7.92E-31	7.85E-30
EBF3	0.35716588	1.150825616	1.688003066	5.02E-15	1.32E-14
HMGN1P17	1.090400898	0.139149509	-2.970150928	1.57E-34	3.05E-33
AL158151.1	0.116150172	0.308073828	1.407284833	2.95E-18	9.56E-18
TMEM273	1.481625446	3.707658496	1.323327586	5.86E-23	2.70E-22
TRBV5-6	0.114617708	0.736621355	2.684093268	1.11E-19	4.00E-19
POU2F2	0.37479018	1.434952726	1.936848152	6.12E-28	4.32E-27
AL359881.1	0.22930959	0.83003986	1.855883927	5.26E-08	8.93E-08
AC105052.2	0.20876527	0.43714477	1.066229415	6.17E-13	1.42E-12
IYD	4.423878008	0.238376528	-4.213997512	3.37E-29	2.74E-28
VWA8	9.027156764	4.199925602	-1.103907889	5.92E-33	8.35E-32
AC096733.2	1.7752	0.632467944	-1.488917308	1.26E-32	1.68E-31
PRSS22	2.439815783	0.244630086	-3.31809847	1.54E-36	5.24E-35
PGF	1.895384371	29.09362359	3.940140642	1.71E-35	4.23E-34
AC009237.8	2.240744128	0.703462564	-1.67143236	1.64E-21	6.80E-21
AC009549.1	0.273816426	1.40451562	2.358791766	1.63E-31	1.78E-30
WASH7P	0.325540332	0.87989231	1.43449067	1.79E-20	6.84E-20
TRBC2	2.576459104	18.20364254	2.820765564	5.03E-29	4.00E-28

Table S1. The differentially expressed genes(DEGs) were screened out in TCGA

ID	conMean	treatMean	logFC	pValue	FDR
ATP6V0E2-A	1.195025093	0.498374701	-1.261738172	1.62E-26	1.00E-25
IGLON5	0.448483657	2.641338327	2.55814179	1.32E-21	5.49E-21
AC139100.2	0.276792594	0.989477556	1.83786164	5.48E-17	1.63E-16
AC005154.4	0.110775601	0.419301689	1.920348488	1.74E-16	5.03E-16
SLC10A6	0.140673225	0.908405767	2.690989102	9.38E-29	7.24E-28
BLOC1S1-RD	0.272422906	1.129101781	2.051255621	2.19E-34	4.11E-33
ATAD3B	0.543577647	1.479137052	1.444197698	7.57E-16	2.10E-15
COL6A3	4.669706067	12.03402667	1.365715812	5.96E-11	1.21E-10
KRBA1	1.820098521	12.81021815	2.815206595	4.16E-37	1.75E-35
ANO4	0.154154371	2.038379574	3.724975027	6.75E-28	4.74E-27
CILP	0.885668187	0.425556052	-1.057417131	6.72E-20	2.46E-19
KCNN4	0.325663654	0.927194318	1.50948901	4.65E-20	1.72E-19
PLA2G4F	6.118355944	1.057549357	-2.532419044	2.09E-36	6.67E-35
FGR	1.999401647	5.741111935	1.521761869	1.98E-28	1.47E-27
HLA-DMA	20.48890526	57.94869739	1.499933329	3.00E-30	2.76E-29
CYP24A1	7.117859816	2.884707878	-1.303018289	4.15E-17	1.25E-16
AC027601.3	0.159391575	0.330296218	1.051185074	1.26E-07	2.08E-07
DNASE1	6.917358171	1.228918906	-2.492831441	5.91E-32	6.98E-31
HMOX1	24.87096883	128.4014111	2.368126444	1.97E-32	2.54E-31
SNORD11	0.138390061	0.489885	1.823702783	1.32E-06	2.05E-06
ALDH1B1	33.73385857	14.48256156	-1.21988055	2.00E-17	6.14E-17
MARVELD2	11.71952297	2.21833284	-2.401366098	4.75E-39	4.02E-37
AL121760.1	0.068338329	0.384618514	2.492661336	1.14E-25	6.60E-25
ABCG2	2.185107588	4.447830453	1.02539748	3.19E-06	4.84E-06
AC087286.2	0.260310658	0.593216748	1.188324947	2.98E-07	4.82E-07
AC004263.1	0.03976167	0.266426599	2.744287858	2.39E-16	6.84E-16
AC073636.1	0.079442661	0.280968039	1.822420181	1.92E-16	5.54E-16
NAP1L2	7.8309385	1.674522319	-2.225435612	1.57E-37	7.68E-36
RASGEF1A	0.781237038	1.608797951	1.042150897	3.01E-05	4.28E-05
GRIK4	0.094151587	0.295100256	1.64814785	0.000226616	0.000304542
AL031719.2	0.333552738	1.262468026	1.920260062	3.28E-28	2.39E-27
AC005632.2	0.315213718	0.823502964	1.38544352	3.70E-25	2.04E-24
AP005019.1	0.112589153	0.353161506	1.649260257	1.19E-19	4.27E-19
LNCTAM34A	0.366132159	1.55309642	2.084710995	2.27E-35	5.43E-34
TNNC1	5.631231013	0.540819838	-3.380230359	7.86E-39	6.13E-37
ATP6V1C2	7.384937193	1.767208491	-2.063113396	2.16E-36	6.88E-35
AC004908.2	0.510546921	1.043389972	1.031163013	6.00E-08	1.02E-07
ATP6V0E2	21.10343333	8.494383992	-1.312896498	1.42E-31	1.56E-30
NFAM1	0.668158324	2.910535405	2.123022665	5.15E-34	8.92E-33
NLRP3	0.569211841	1.490087104	1.388359088	2.17E-20	8.21E-20
C5AR1	3.605503958	9.359148153	1.376176299	3.16E-23	1.49E-22
LINC01780	0.850135904	0.244153942	-1.799902419	2.65E-19	9.29E-19
MSX1	0.870650244	2.215667161	1.347575991	6.37E-24	3.17E-23
AC127024.5	0.722971213	1.541385173	1.092217311	3.93E-11	8.08E-11
AL451050.2	0.0839342	0.246672464	1.555265995	5.27E-09	9.53E-09
AC080013.3	0.089547583	0.296640168	1.727987571	6.32E-10	1.21E-09
MAPK12	0.772506765	1.928050374	1.319523273	2.34E-07	3.81E-07
ICOS	0.068278474	0.728396481	3.415221227	1.60E-31	1.75E-30
FCGR1B	0.069804065	0.506991688	2.860579146	1.18E-35	3.03E-34
AC145423.3	0.344614693	0.756802161	1.134931996	1.05E-06	1.64E-06
AC112484.1	0.149920898	0.330024369	1.138371054	4.56E-12	9.94E-12
SEC31B	0.327714919	1.075291458	1.714214498	4.50E-11	9.20E-11
ENTPD8	1.445470081	0.43740238	-1.724505773	2.62E-25	1.46E-24
HOXB8	17.58115351	3.680588199	-2.256021482	4.52E-34	7.94E-33
LINC01730	0.055357133	0.26712062	2.270650227	7.35E-18	2.32E-17

Table S1. The differentially expressed genes(DEGs) were screened out in TCGA

ID	conMean	treatMean	logFC	pValue	FDR
C5	1.98548505	0.974544859	-1.026690998	2.91E-32	3.60E-31
C5orf38	3.068163514	0.499474362	-2.61889284	7.31E-34	1.23E-32
AC093535.1	0.2093187	0.560924637	1.422105744	3.46E-14	8.61E-14
PRAME	0.581748643	3.950837208	2.763690558	0.000466427	0.000613009
SHMT2	11.77514142	46.41954134	1.978987881	1.22E-37	6.22E-36
SHC3	0.59571092	0.209852883	-1.505234119	1.71E-25	9.76E-25
IGLV2-28	0.351235892	0.831140377	1.242651888	0.000376258	0.000497681
SLC16A3	3.891058964	31.64135822	3.023576782	2.24E-37	1.04E-35
MMP25	0.159609838	0.757231273	2.246184413	8.90E-34	1.48E-32
APOBR	1.117356768	3.537443977	1.662617393	8.53E-26	4.98E-25
S100A5	0.95986352	0.320581968	-1.582136007	1.95E-28	1.45E-27
AC018648.1	0.059203504	0.25742098	2.12037518	1.33E-17	4.14E-17
RNU6-431P	0.123169206	0.48117558	1.965921817	1.39E-12	3.12E-12
LINC02555	0.063150592	0.362904438	2.522721526	6.19E-14	1.52E-13
ACAA1	20.18380894	6.799000942	-1.56980378	7.74E-32	8.91E-31
BX547991.1	2.65848761	0.332068375	-3.001053505	2.29E-34	4.25E-33
LINC00893	0.168802925	0.800961054	2.246392189	9.65E-17	2.83E-16
XCL1	0.196233068	1.000537621	2.350135332	2.24E-23	1.07E-22
COL9A3	0.527573412	0.203424129	-1.374881045	9.92E-21	3.86E-20
PRKCQ-AS1	2.779532613	0.917097311	-1.599695582	2.80E-23	1.32E-22
SLC15A4	4.914226486	17.68454055	1.847452483	5.57E-39	4.64E-37
HAMP	0.042262231	0.565979605	3.74330925	2.84E-29	2.33E-28
AC110749.1	0.087058455	0.38947601	2.161478149	6.85E-10	1.31E-09
AACSP1	0.20234816	0.672706112	1.733136633	4.33E-11	8.86E-11
MS4A14	0.119300338	1.1070036	3.213989882	4.52E-36	1.30E-34
PPEF1	0.044016187	0.286455638	2.702205654	3.13E-28	2.28E-27
AC007342.4	1.316497929	0.173546817	-2.923308443	9.65E-37	3.55E-35
PROZ	4.27465719	0.243415444	-4.134316116	4.72E-21	1.88E-20
AC007283.2	0.205348121	0.892044764	2.119044365	7.02E-16	1.95E-15
SMCO3	1.524416775	0.695489438	-1.132156879	4.99E-19	1.71E-18
DNASE1L3	10.32917564	2.346363889	-2.138226441	5.14E-35	1.13E-33
AL645929.1	2.079993101	6.34009256	1.607925159	4.48E-21	1.79E-20
AL021154.1	0.173317843	0.441543001	1.349133762	3.20E-11	6.61E-11
AC015660.2	0.303284273	1.177231154	1.956655033	6.67E-16	1.86E-15
AATK	0.349075151	0.939531148	1.428403329	3.54E-23	1.66E-22
AL035420.3	0.613909382	0.252772902	-1.280183909	1.17E-20	4.53E-20
CCNYL2	0.083645897	0.620798665	2.891758779	7.70E-18	2.43E-17
NOX4	13.94177603	5.867594815	-1.248573201	0.003417254	0.004223461
ARL4AP4	0.077466334	0.248901636	1.683934347	1.14E-05	1.67E-05
CLCNKA	71.58846046	1.768176081	-5.339393199	1.73E-39	1.90E-37
AC097641.2	0.191838032	0.448573126	1.225454431	3.47E-11	7.16E-11
METTL27	2.939905196	6.192419188	1.074733504	3.21E-09	5.87E-09
AC093520.2	0.007361274	0.251314751	5.09339615	6.36E-22	2.71E-21
ASIC3	0.110392172	0.45563984	2.045256023	9.38E-20	3.40E-19
AC087239.1	0.123115105	0.428898151	1.800627322	6.87E-14	1.68E-13
C9orf66	7.224883804	3.452072416	-1.065511656	0.006028647	0.007310076
AC091390.4	0.27786212	0.651427433	1.229235304	1.59E-21	6.58E-21
AP003774.4	0.049876446	0.313073229	2.650069577	1.90E-25	1.07E-24
AL671277.1	5.187417128	14.56326379	1.489245427	2.97E-09	5.45E-09
NABP1	0.775289272	1.799856428	1.215075222	1.72E-16	4.97E-16
CENPU	0.648318814	2.289110771	1.820011932	2.39E-34	4.42E-33
IGHG4	25.72455506	57.58469046	1.162539182	0.001218361	0.001558182
RPS6P25	0.20194271	0.415725833	1.041686333	1.51E-13	3.60E-13
CFAP58-DT	0.286627775	0.642542017	1.164612378	2.21E-13	5.24E-13
RN7SL541P	0.043282614	0.596650087	3.785025556	2.47E-24	1.27E-23

Table S1. The differentially expressed genes(DEGs) were screened out in TCGA

ID	conMean	treatMean	logFC	pValue	FDR
ADGRV1	0.80948881	0.125403046	-2.690438748	6.71E-36	1.85E-34
BX539320.1	0.200132513	0.698220653	1.802727466	3.18E-23	1.49E-22
AC105105.1	0.036781249	0.405513804	3.462708645	3.24E-26	1.96E-25
DNAJC22	4.911636639	10.98375696	1.161095868	3.56E-21	1.43E-20
MIR5188	0.087557131	0.378108294	2.110502918	8.22E-13	1.88E-12
TWIST1	0.287428117	0.650246359	1.177785217	1.12E-08	1.98E-08
TRPC7-AS1	1.779276338	0.499540343	-1.832617493	4.71E-10	9.05E-10
RNU4-78P	0.144739417	0.447346096	1.627933557	8.37E-07	1.32E-06
AC073218.2	0.106479487	3.861052261	5.180346648	5.96E-33	8.39E-32
TROAP	0.122923936	0.853685216	2.795938327	1.47E-35	3.69E-34
CGREF1	0.912822861	7.771962178	3.089872051	2.93E-31	3.08E-30
CASP5	0.03871163	0.30118713	2.959821148	3.19E-32	3.92E-31
TNFRSF14	5.158948625	23.1763763	2.167506029	1.00E-38	7.50E-37
CREB3L3	1.220201108	8.002731616	2.713373581	2.74E-19	9.59E-19
AC005034.4	0.749171785	0.227147565	-1.721666728	1.01E-28	7.75E-28
AP000523.1	0.03204746	0.283348377	3.144294995	1.86E-29	1.55E-28
AC141557.2	0.034163917	0.358754161	3.392450264	6.74E-22	2.86E-21
LINC00626	0.13962712	2.035359276	3.865632388	1.68E-07	2.76E-07
RAD54B	0.24522626	0.513721591	1.066873233	1.40E-12	3.15E-12
Z97192.2	0.103600399	0.215235684	1.054887726	2.42E-05	3.46E-05
AC004951.3	0.095118798	0.339192163	1.834300455	4.42E-19	1.52E-18
IRX3	8.751928	24.73171345	1.498689421	5.75E-21	2.27E-20
ODF3B	1.017547711	11.73498163	3.527647236	6.74E-38	3.80E-36
HRG	10.93005619	2.354339139	-2.21490676	7.84E-42	2.83E-39
NCR1	0.050670735	0.327064827	2.690351947	8.14E-32	9.33E-31
IQGAP3	0.260562429	1.461545535	2.487793796	4.99E-33	7.17E-32
DARS	24.29280306	51.16707017	1.07468666	7.12E-32	8.27E-31
PPP1R14D	1.857774729	7.596455524	2.031750846	0.005797606	0.007041123
ACAN	0.723247813	4.431471681	2.615223933	2.78E-24	1.42E-23
PNPLA1	1.718769045	0.322536761	-2.413840195	7.73E-11	1.56E-10
AP003555.2	0.027965087	0.437592764	3.967890182	8.79E-35	1.81E-33
TNFAIP8L2	1.337980286	6.368755729	2.25095468	2.21E-34	4.14E-33
EIF5AP3	0.028420873	0.33066148	3.540332227	1.84E-25	1.05E-24
FCMR	1.434301263	4.047076591	1.496532071	9.13E-22	3.84E-21
GLTP	48.31644542	23.18606296	-1.059256453	1.83E-24	9.55E-24
PYCARD	2.459117529	9.994204781	2.022951091	1.14E-30	1.11E-29
ABCA1	3.708622333	11.2231736	1.597525424	2.81E-31	2.97E-30
BTLA	0.124086365	0.34512765	1.475785466	8.91E-15	2.30E-14
BMS1P1	0.36112876	0.729698012	1.014786204	9.46E-09	1.68E-08
AD001527.1	0.127593695	0.576003892	2.174521519	4.91E-13	1.14E-12
RPS14P8	0.472891084	1.062518495	1.167908108	4.16E-05	5.87E-05
AL137009.1	0.478427786	0.210880055	-1.181878527	4.72E-14	1.16E-13
LY6H	0.117695956	1.230300631	3.385874235	6.33E-22	2.70E-21
IDSP1	0.081542266	0.302084381	1.88933163	1.00E-06	1.57E-06
AC093690.1	0.061226114	0.261658656	2.095466971	2.48E-21	1.01E-20
UBD	2.812574586	26.43713419	3.232602535	1.02E-26	6.46E-26
AL590822.1	0.088636204	0.768604924	3.116274215	3.93E-12	8.60E-12
FGF1	26.41248142	1.479408148	-4.158127816	3.23E-31	3.40E-30
ACR	0.121711299	0.307961216	1.33928556	1.58E-14	4.02E-14
AC087289.2	0.107232841	0.292999522	1.450151496	5.98E-15	1.56E-14
TNFSF14	0.114871977	1.55979764	3.763260072	4.76E-34	8.30E-33
KIF18A	0.205855315	0.601444387	1.546800644	1.07E-29	9.21E-29
AC005776.1	0.035039633	0.333274108	3.249649648	2.13E-14	5.36E-14
PRKX	11.59000861	5.77867531	-1.004070923	3.31E-11	6.83E-11
AIM2	0.159185024	1.32448773	3.056657959	3.08E-28	2.25E-27

Table S1. The differentially expressed genes(DEGs) were screened out in TCGA

ID	conMean	treatMean	logFC	pValue	FDR
AC138230.1	0.183758259	0.484032719	1.397295477	1.32E-08	2.33E-08
AC010247.2	0.075203435	0.244391859	1.700325756	2.77E-24	1.42E-23
ACRBP	0.662511444	1.625963537	1.295277623	1.53E-23	7.41E-23
AC131009.3	0.472820813	1.231992601	1.381628145	1.60E-20	6.12E-20
LINC00115	0.300086003	0.742111658	1.30626024	3.84E-10	7.42E-10
AC097534.2	0.493840268	3.709262762	2.909016086	3.21E-35	7.37E-34
JMY	4.9709135	2.202805873	-1.174168639	5.87E-34	1.01E-32
SCGB3A1	0.2821962	0.891585871	1.659675191	2.78E-13	6.54E-13
TTC21B-AS1	0.037142902	13.27978033	8.481929016	1.68E-36	5.56E-35
AC073316.2	0.262737228	0.875169623	1.735942032	5.57E-10	1.07E-09
LGALS17A	0.016765179	0.509799721	4.926390772	3.59E-26	2.17E-25
ANLN	0.522617155	2.123145237	2.022376677	1.69E-30	1.61E-29
FAM83F	1.09597734	0.52126547	-1.07212777	3.50E-20	1.30E-19
OACYLP	0.023599548	2.564611281	6.763837138	9.12E-39	6.93E-37
LAG3	0.321975235	3.78157404	3.553965234	5.32E-29	4.22E-28
RDM1P3	0.071581151	0.245077208	1.775584666	5.94E-11	1.21E-10
AC124319.1	0.111509303	0.428196457	1.941108789	4.64E-08	7.90E-08
NXPH2	2.683930411	0.205499622	-3.707139619	7.63E-47	6.05E-44
CADM4	17.45104154	5.452549978	-1.67831015	1.98E-29	1.65E-28
RNA5SP527	0.104913051	0.331680217	1.660598808	3.02E-07	4.88E-07
RBM11	1.038053208	0.176092954	-2.559471306	1.25E-35	3.20E-34
AC016596.1	3.820450167	8.531426684	1.159044377	4.32E-16	1.22E-15
HM13-IT1	0.27159886	1.359948026	2.324002186	6.03E-26	3.57E-25
GLYATL1	28.64842948	11.43953843	-1.32442721	2.71E-05	3.87E-05
AC090181.2	0.20037267	0.50301025	1.327902053	3.91E-09	7.12E-09
AC009119.2	0.156303631	0.438230686	1.487339219	2.23E-16	6.41E-16
SLCO1C1	0.070529577	0.406953682	2.528564315	7.02E-30	6.15E-29
RNU6-353P	0.087261901	0.337702651	1.952329701	0.014604355	0.017181397
SMIM32	14.89402588	6.309669622	-1.239097398	1.27E-09	2.39E-09
ANKRD37	1.911317899	4.828163202	1.336906689	1.56E-27	1.06E-26
MAMDC4	0.855645818	2.995246688	1.807589185	3.06E-17	9.31E-17
FUT11	4.624923431	13.74948659	1.571876363	1.52E-34	2.95E-33
ACAD8	7.28976025	3.498253484	-1.059236536	3.27E-36	9.74E-35
IGHV2-70D	0.404168767	3.132870012	2.954455167	1.67E-07	2.75E-07
AC103563.3	0.077983211	0.331891647	2.089476858	0.021986862	0.025540068
IL4R	4.881285833	13.64068417	1.482582868	6.75E-35	1.44E-33
NCMAP	1.250661357	0.313875857	-1.994425235	9.12E-27	5.78E-26
FPR2	0.163058047	0.412641538	1.339503416	1.95E-11	4.09E-11
AL161669.1	0.218977187	1.05455292	2.267779015	1.76E-17	5.42E-17
AL122020.1	0.525168843	1.118981651	1.091333146	7.52E-13	1.72E-12
AC002091.1	0.2307892	0.802366979	1.79768652	4.34E-25	2.38E-24
SYTL3	2.178595792	5.022064227	1.20488193	1.37E-21	5.68E-21
GPC5-AS1	0.413017039	0.205997779	-1.003572519	6.51E-35	1.39E-33
FRG2C	2.011799035	0.098879493	-4.346671036	7.72E-29	6.01E-28
ATP6V0D2	42.30824335	14.59175086	-1.535785783	1.79E-35	4.39E-34
LYZ	20.62648308	114.1078376	2.467828128	1.72E-24	8.96E-24
HAS2	0.835206191	0.406326596	-1.039492608	3.41E-09	6.23E-09
PHBP9	0.259996196	0.674951232	1.37629275	4.46E-13	1.04E-12
BLM	0.177353708	0.422022941	1.250691934	5.55E-26	3.29E-25
SAA2-SAA4	0.092875216	3.962203899	5.414865661	0.028172342	0.032417403
SDS	0.257938154	5.425802914	4.394739548	1.86E-39	1.99E-37
NRXN2	1.134871013	3.371971907	1.571064184	6.61E-13	1.52E-12
VEGFC	3.189453496	6.994936381	1.133001693	1.55E-17	4.81E-17
RTN4RL2	0.993085525	2.01762223	1.022666202	6.55E-08	1.10E-07
HSPE1P11	0.052828104	0.289619386	2.454780633	0.001462352	0.001859714

Table S1. The differentially expressed genes(DEGs) were screened out in TCGA

ID	conMean	treatMean	logFC	pValue	FDR
AC092032.1	0.132134298	0.399114618	1.594798125	2.13E-12	4.74E-12
P2RY2	1.143417576	0.419736465	-1.445796664	2.43E-23	1.16E-22
IGKV3-20	36.32660977	160.4613739	2.14312742	3.99E-05	5.62E-05
PADI3	0.125578809	1.037830567	3.046905992	1.82E-17	5.62E-17
PPP1R35	5.031191444	10.11860369	1.008038228	3.05E-22	1.33E-21
AC116366.1	0.244980328	1.022029135	2.060698511	7.87E-25	4.23E-24
RPS28P7	145.406802	300.7078053	1.048267557	1.18E-09	2.22E-09
GJA1	17.40098624	60.51947384	1.798230369	2.30E-25	1.29E-24
AC008731.1	0.129552914	0.424071741	1.710766881	2.96E-10	5.76E-10
PBK	0.378974348	1.304672361	1.78351545	2.33E-30	2.17E-29
HTR7	0.115943217	0.400627176	1.788841871	5.48E-20	2.02E-19
RPL13AP25	2.367730482	6.275784185	1.406290872	7.66E-15	1.99E-14
LILRB3	0.191575925	1.056059909	2.462703506	3.19E-35	7.31E-34
IGHJ2	0.929418008	5.149062181	2.469910189	0.000138464	0.000188725
TRDV1	0.137387798	0.746249974	2.441405095	6.49E-20	2.38E-19
AC002059.1	0.089059067	0.529891689	2.572863097	2.61E-20	9.82E-20
SEMA3A	0.189572838	0.41963461	1.146381405	0.002418253	0.003021606
LILRB1	0.386682366	2.985894924	2.948942517	3.59E-39	3.28E-37
SH3GL1	12.44718822	25.71262339	1.046656933	3.72E-32	4.51E-31
HNRNPA1P4	0.090898561	0.295004595	1.698408068	1.44E-12	3.23E-12
NPDC1	7.526885069	19.71118544	1.388889696	5.59E-24	2.80E-23
AL008582.1	0.135247671	0.577301808	2.093721998	2.57E-16	7.33E-16
RNU4-82P	0.078375234	0.279491529	1.834334814	1.38E-08	2.43E-08
MIR579	0.11871469	0.376530248	1.665267296	1.81E-05	2.62E-05
AC087442.1	0.049063755	0.230458299	2.23177617	2.46E-07	4.01E-07
AC010422.4	0.154836327	0.354913864	1.196724942	0.000481215	0.000631921
RBM47	32.41202653	15.25272568	-1.08746215	9.42E-36	2.50E-34
SPARC	157.9616897	569.4551487	1.850007512	2.04E-32	2.61E-31
AL359397.1	1.086088829	0.342991112	-1.662899008	5.40E-22	2.31E-21
SNORC	0.209656909	0.686175812	1.710547899	1.34E-14	3.42E-14
AL157931.1	1.605201349	9.353443464	2.542743317	7.63E-08	1.28E-07
DAO	12.55426448	1.382285341	-3.183050145	1.16E-11	2.47E-11
SBNO2	5.164889278	10.42104984	1.012691298	3.89E-24	1.97E-23
AC129507.4	1.895806248	0.539725507	-1.812513752	2.09E-24	1.08E-23
SPTBN5	0.185392419	0.464092879	1.323831312	0.000313154	0.000416675
AC119751.6	0.015862134	0.325389335	4.358508181	8.34E-17	2.46E-16
CD52	6.109696011	26.21698756	2.101329418	1.57E-28	1.19E-27
CELF4	0.117240508	0.24832717	1.082770996	0.008782524	0.010525328
AC016700.2	0.261544812	0.640643061	1.292462622	3.48E-10	6.75E-10
AL513320.1	0.326674521	0.944454675	1.531627628	4.18E-06	6.30E-06
DIAPH2	2.3341645	5.17513028	1.148688943	5.14E-28	3.66E-27
KRT20	0.044700591	0.819207406	4.195862944	0.016586126	0.019425175
SNORA53	0.312724092	28.53008392	6.511449814	2.55E-09	4.70E-09
RAPGEF4	1.796226444	3.679776594	1.034648943	2.66E-13	6.28E-13
CTF1	7.684911203	3.378299894	-1.185731187	1.26E-26	7.89E-26
WNT4	0.811476534	0.215655529	-1.911820673	1.74E-19	6.19E-19
AC005165.2	0.130204789	0.43972502	1.755819108	1.70E-10	3.36E-10
CRACR2B	1.761232851	3.942594574	1.162559703	2.93E-13	6.88E-13
AC106782.6	0.595328908	1.199866023	1.011114468	3.18E-17	9.65E-17
SULT2A1	0.018168638	0.369390249	4.345623584	0.001983486	0.002494564
AC004253.1	0.148081443	0.831291728	2.488963995	5.64E-22	2.41E-21
AC005837.3	0.045822233	0.319215692	2.800411898	1.59E-10	3.16E-10
IFI30	0.192021464	0.970552	2.337537931	3.39E-26	2.05E-25
HSPA6	0.654532543	2.747032921	2.069337371	2.90E-31	3.06E-30
AC011676.1	0.786472168	0.256174366	-1.618269591	7.19E-12	1.55E-11

Table S1. The differentially expressed genes(DEGs) were screened out in TCGA

ID	conMean	treatMean	logFC	pValue	FDR
AQP6	17.70035548	7.611376706	-1.217549005	1.77E-38	1.22E-36
HLA-DOB	0.636122774	2.937564404	2.20724334	2.20E-20	8.32E-20
TREML1	0.059384478	0.469524638	2.983043061	1.73E-34	3.32E-33
PLAT	26.08385588	7.754012856	-1.750142121	2.32E-35	5.49E-34
MIR5685	0.161398326	0.487502799	1.594784882	3.45E-08	5.92E-08
AC074286.1	0.131842682	0.49110517	1.897214516	1.14E-18	3.80E-18
CNP	27.86898042	11.13392783	-1.323697586	9.30E-34	1.54E-32
RN7SL130P	0.269914912	0.844187494	1.645058776	3.21E-13	7.52E-13
MKI67	0.406896226	1.886047409	2.212633135	1.31E-31	1.46E-30
IGHV3-60	0.095580371	0.262736471	1.458830214	0.026524125	0.030590929
AC005740.4	0.253465327	0.566824288	1.161113172	2.64E-16	7.52E-16
AL031726.1	2.981812449	0.069165083	-5.430001803	4.13E-39	3.64E-37
CCL18	0.240432572	12.12416365	5.656109063	1.08E-30	1.06E-29
TRPM6	1.109150587	0.148155525	-2.904270921	4.77E-18	1.53E-17
RNU6-611P	0.175350911	1.024266305	2.546274027	1.15E-11	2.45E-11
MIR647	1.437939718	6.738405274	2.228404005	5.30E-16	1.48E-15
LINC00323	1.036565602	0.425140913	-1.285798416	5.19E-21	2.06E-20
PTPN4	4.347065708	1.858788437	-1.225679328	9.95E-37	3.63E-35
AC092078.2	2.525482063	0.022051541	-6.839535523	1.07E-49	1.30E-46
PC	13.14827386	5.074872195	-1.373430016	6.36E-15	1.66E-14
SOD2	39.52757972	90.10933722	1.188816986	4.61E-15	1.21E-14
RNU6-720P	0.256413729	0.609728592	1.24969369	0.00026551	0.00035514
PDE1A	25.68797689	3.46947926	-2.888302225	2.89E-39	2.80E-37
PABPC1P4	1.284807288	0.528633357	-1.281212614	1.50E-27	1.02E-26
MIR421	0.234096458	0.698461685	1.577077869	3.87E-05	5.46E-05
PTPRD	5.150893151	2.418181667	-1.090899981	8.07E-08	1.35E-07
GOT1	84.95069431	40.84269312	-1.05654774	4.68E-28	3.34E-27
TRGV2	0.052945686	0.356171188	2.749985772	4.09E-25	2.25E-24
SCTR	4.773160885	1.749431184	-1.448059049	4.01E-14	9.93E-14
LDHA	90.01795639	283.8685387	1.656938245	7.71E-35	1.60E-33
AC103563.2	1.603528419	0.289782957	-2.46820527	2.54E-32	3.21E-31
TSGA10	1.089284418	0.530246345	-1.038646023	4.72E-28	3.37E-27
AL161747.1	0.072011156	0.301232841	2.064586738	1.05E-11	2.23E-11
AC010970.1	0.246680712	1.426183786	2.531443088	0.001762441	0.002225126
CAV2	6.180266861	24.30287911	1.975386197	3.44E-38	2.17E-36
AC007000.1	0.246233351	0.509847701	1.050040178	4.58E-11	9.37E-11
BDNF-AS	1.609535926	0.794672428	-1.018212585	1.92E-32	2.48E-31
AC134879.2	0.127182235	0.425727897	1.743034461	0.004687972	0.005731012
MYCN	1.312991738	0.371646926	-1.820853259	2.79E-21	1.13E-20
CAPN5	10.17730922	4.869008717	-1.063656189	1.02E-32	1.39E-31
PAG1	1.443285083	4.876323406	1.756437516	6.18E-34	1.06E-32
HIST4H4	0.419419956	0.865022876	1.044342781	1.74E-07	2.85E-07
AC063976.2	0.038549317	0.265454173	2.783685615	1.39E-26	8.68E-26
GAL3ST1	6.995447208	53.04403877	2.922702432	2.39E-30	2.23E-29
CXCL5	0.211824868	5.253037935	4.632208128	1.39E-10	2.77E-10
AC016747.2	0.095317946	0.252480525	1.405352345	7.22E-08	1.22E-07
AC099850.3	0.640911625	2.18794834	1.771381333	3.39E-24	1.72E-23
FCAMR	9.941867563	3.608018646	-1.462310091	3.72E-05	5.25E-05
ALDH1L1	15.76332322	7.277239312	-1.115108555	3.33E-10	6.46E-10
WFIKKN1	0.320360779	0.771844669	1.268613005	1.40E-05	2.04E-05
AC016026.1	0.033276527	0.238546728	2.841695119	1.33E-29	1.14E-28
LPCAT1	7.316948347	42.07852589	2.523770185	3.13E-36	9.37E-35
HJURP	0.175017236	1.042089992	2.573910959	4.14E-34	7.36E-33
SYNM	2.819065014	5.995743799	1.088721988	7.26E-25	3.91E-24
AC008760.1	0.424249434	0.86778965	1.032432644	7.19E-07	1.14E-06

Table S1. The differentially expressed genes(DEGs) were screened out in TCGA

ID	conMean	treatMean	logFC	pValue	FDR
AC120498.3	0.066387444	0.352439608	2.408393747	2.15E-05	3.09E-05
IRX1	9.419194583	0.396656897	-4.569640162	9.65E-41	2.16E-38
LINC02541	0.043188738	0.267704167	2.631912537	7.20E-21	2.82E-20
SIGLEC16	0.14958101	0.368353138	1.300162501	4.12E-11	8.46E-11
CTH	5.554299703	1.53365613	-1.856629981	1.54E-37	7.56E-36
ZNF683	0.115487873	1.209809755	3.388966927	3.29E-33	4.87E-32
TRAM1L1	7.006953542	2.464245189	-1.507641521	8.06E-29	6.27E-28
IGKV3OR2-2(	0.142940505	1.554909878	3.443344267	0.00010245	0.000140748
AC136475.5	0.110998273	0.242860053	1.129587978	1.77E-09	3.30E-09
SNORD104	1.413838704	8.331461407	2.558952037	7.17E-22	3.04E-21
AP003469.4	0.907437374	2.184245148	1.267264801	1.58E-24	8.29E-24
AC024361.3	0.045829807	0.356237808	2.958482514	5.51E-20	2.03E-19
HOXB9	24.15763419	2.374513435	-3.346775351	5.57E-37	2.24E-35
CIDEC	0.326088786	0.88057837	1.43318658	0.002331261	0.002915917
MID1IP1-AS1	2.164669724	0.828685085	-1.385251061	1.48E-30	1.42E-29
ALKAL2	2.460337583	8.03056499	1.706645211	9.98E-10	1.88E-09
RN7SL67P	0.042388856	0.426426292	3.330539458	1.23E-14	3.14E-14
C5orf46	0.444219216	13.49408672	4.924911725	4.00E-37	1.69E-35
RPL31P52	0.073388158	0.3043666	2.052190851	1.82E-14	4.61E-14
AL136126.1	0.114406201	0.272992199	1.25469447	8.42E-13	1.92E-12
AL139287.1	2.075306869	5.486949279	1.40267956	2.36E-14	5.92E-14
AC009120.2	0.521049923	1.716232632	1.719751609	1.15E-17	3.59E-17
CORO2B	5.227825319	1.304071421	-2.003188051	1.97E-37	9.30E-36
AC002470.1	1.412147882	0.585148571	-1.271016297	9.77E-26	5.67E-25
AL391832.2	0.108319647	0.218769577	1.014117188	1.09E-13	2.64E-13
CCL22	0.319994012	0.673709132	1.074080948	5.63E-12	1.22E-11
SH2D1A	0.376502135	2.149816285	2.513483427	9.19E-27	5.82E-26
VXN	0.32265325	1.042066362	1.691390692	4.93E-13	1.14E-12
HYAL1	24.93277394	11.76320343	-1.083762405	3.70E-31	3.83E-30
MIR378B	0.013860365	0.435407652	4.97332958	8.16E-16	2.26E-15
RPL10P13	0.152357853	0.544312865	1.836972276	5.55E-09	1.00E-08
EDAR	0.548990449	0.253459016	-1.115028566	4.27E-19	1.47E-18
SIRPB2	0.30079028	1.351912262	2.168171671	3.46E-31	3.61E-30
ARHGEF26	1.418960936	0.554307684	-1.35607596	1.19E-19	4.29E-19
HCK	2.050127786	8.59168049	2.067226506	3.51E-33	5.17E-32
AL163051.1	0.564558999	0.244077215	-1.209786732	4.06E-24	2.05E-23
ALPI	0.763874302	5.040047415	2.722030143	0.024883035	0.028780445
AC239803.3	0.069057336	0.306794199	2.151404628	1.14E-16	3.34E-16
AC087289.5	0.111147604	0.296692251	1.416490403	8.88E-18	2.79E-17
AC136632.1	1.624702597	5.77391277	1.829373656	1.63E-22	7.24E-22
PPM1J	0.693380361	0.299887959	-1.209223377	1.49E-26	9.28E-26
AP1G2	1.875617753	4.217318889	1.168960275	3.62E-16	1.03E-15
GGACT	11.30962573	2.332852925	-2.277383927	9.19E-25	4.91E-24
AC016747.3	0.119158707	0.378848627	1.668737151	6.32E-13	1.45E-12
AIF1	14.58156717	30.7601602	1.076917234	7.27E-16	2.02E-15
IGLV1-44	11.27860858	48.7341877	2.111345102	2.07E-06	3.17E-06
GALM	40.66423201	18.38678727	-1.145090948	7.58E-22	3.21E-21
CHST15	5.040470694	16.10054749	1.675479379	3.49E-23	1.63E-22
AC022558.3	0.118206197	0.383756613	1.698885936	3.16E-16	8.98E-16
AC012676.5	0.246254406	0.869672326	1.820322391	9.92E-18	3.11E-17
AC069234.5	0.253576775	0.695522762	1.455675115	2.66E-25	1.49E-24
CYP51A1	1.791744011	0.812039224	-1.141743211	1.68E-30	1.60E-29
MIR3176	0.405845286	1.697479296	2.064392217	1.79E-12	4.00E-12
AL391987.4	0.369217546	0.177452033	-1.057042012	7.58E-17	2.24E-16
LMNB1	1.941582879	4.905947053	1.337298372	2.43E-26	1.49E-25

Table S1. The differentially expressed genes(DEGs) were screened out in TCGA

ID	conMean	treatMean	logFC	pValue	FDR
FAM163A	0.060743942	0.571592853	3.234175437	7.82E-34	1.31E-32
AL353803.5	0.148096053	0.504178583	1.767401641	3.02E-09	5.54E-09
BX293535.1	0.142735605	0.326458769	1.193555541	3.56E-06	5.39E-06
CD80	0.0603403	0.340053775	2.494569123	1.89E-29	1.58E-28
CDK18	9.204546861	48.65050373	2.402036138	3.75E-32	4.55E-31
SYCE2	0.225065085	0.477981679	1.086613053	6.83E-12	1.47E-11
AL391845.2	0.016768096	0.590421715	5.137955007	1.03E-40	2.25E-38
IGHV3-72	1.79127637	5.567548948	1.636054394	0.00260664	0.003247927
BNIP3P1	0.110459855	0.321717745	1.542273375	6.74E-22	2.86E-21
TJP3	4.784781764	0.648501051	-2.883272307	6.62E-37	2.57E-35
STK32A	1.491644093	0.562583191	-1.406764996	6.77E-28	4.75E-27
TCAIM	6.850039833	3.306462686	-1.050823757	2.24E-36	7.09E-35
HLA-F	6.785350208	37.5298872	2.46754477	6.29E-40	8.58E-38
LINC02273	0.08570194	0.279799367	1.70699293	5.22E-15	1.37E-14
GAREM2	0.719129524	1.697322318	1.23893701	3.52E-20	1.31E-19
CD1A	0.039832605	0.278854276	2.807489666	3.80E-14	9.43E-14
CHSY3	0.327480898	1.513704245	2.20860069	2.65E-32	3.32E-31
NFE4	0.027847185	0.483342128	4.117441358	9.97E-13	2.27E-12
LINC01762	3.893641994	0.161101776	-4.595075936	4.96E-40	7.29E-38
AC093915.1	0.040442217	0.512529181	3.663700165	3.90E-20	1.45E-19
HIST1H2BE	0.258368618	0.98281541	1.927489634	1.34E-06	2.08E-06
EDA2R	1.111160688	5.478233544	2.301643308	3.41E-37	1.48E-35
IGKV1D-27	0.109540928	0.426100141	1.959722528	0.004699614	0.005744561
C5orf56	0.570751476	1.44442429	1.339559995	4.01E-26	2.41E-25
SLC4A9	5.238483251	0.541384469	-3.274423749	3.61E-37	1.56E-35
PCDHB2	0.565923194	1.252837783	1.146521455	0.006002573	0.007281033
HYKK	1.821707118	0.461538218	-1.980769009	6.66E-40	8.96E-38
COBL	4.386521764	1.912183399	-1.197856526	3.29E-29	2.68E-28
S100A8	3.659235781	10.48543314	1.518772175	2.03E-15	5.48E-15
CAPN6	14.07829831	5.494610519	-1.357383836	1.24E-15	3.39E-15
MIR320B2	0.072832029	0.266543467	1.871725886	1.18E-08	2.08E-08
AL359314.1	0.016203699	0.339825844	4.390400501	4.42E-16	1.24E-15
AC084824.5	0.602468371	1.483501864	1.300049332	8.09E-18	2.55E-17
ADHFE1	4.49728644	2.038163023	-1.141785325	1.04E-24	5.55E-24
RN7SL15P	0.05752235	0.274193797	2.253001421	1.59E-07	2.62E-07
BGLAP	0.12450433	0.631912616	2.343529155	8.76E-21	3.42E-20
NDRG2	33.77022325	13.21895484	-1.353143602	7.42E-32	8.58E-31
LINC01625	0.745975386	0.273594968	-1.44708633	8.17E-23	3.72E-22
CDCA8	0.663871956	1.820878278	1.455657571	5.14E-28	3.66E-27
AC245052.4	0.090430671	0.261874188	1.533989788	4.43E-15	1.17E-14
AC109460.1	0.127675322	0.339055241	1.409040651	1.39E-11	2.95E-11
AL133371.2	0.693636516	1.822381365	1.393573145	1.24E-17	3.85E-17
SLC51B	8.016260278	2.836177256	-1.49898165	8.25E-06	1.22E-05
MICE	0.150387182	0.87214647	2.535888843	2.27E-35	5.43E-34
AL136531.2	0.108910552	0.258924219	1.249386172	1.34E-13	3.21E-13
AL096865.1	0.068664485	0.399413322	2.540246448	4.09E-21	1.64E-20
IGHV4-31	2.635738927	15.81837258	2.585321798	6.65E-05	9.25E-05
MET	20.76852771	46.29848048	1.156565898	1.96E-24	1.02E-23
AC083862.2	0.826666775	3.469092316	2.069180424	8.32E-38	4.52E-36
ZMYND15	0.558497018	1.872433833	1.745293256	2.43E-33	3.67E-32
ATAD5	0.251822376	0.629828104	1.322551655	1.60E-26	9.90E-26
CD2	1.81832654	15.03387378	3.047533585	1.83E-32	2.37E-31
AL034397.3	0.246558054	0.945240897	1.938754671	2.37E-21	9.66E-21
PAQR5	24.64039447	7.270684679	-1.760862219	1.39E-38	9.98E-37
KNTC1	0.706844743	1.632347947	1.207483341	2.19E-28	1.63E-27

Table S1. The differentially expressed genes(DEGs) were screened out in TCGA

ID	conMean	treatMean	logFC	pValue	FDR
IGKV2D-24	0.15442559	0.568533003	1.88033226	0.000181221	0.000245071
FCF1P7	0.052581146	0.27893082	2.407289875	7.24E-09	1.30E-08
DTX2	1.158550882	2.844438565	1.295822515	4.09E-37	1.72E-35
CD101	0.237885686	0.65811769	1.468077139	8.63E-24	4.24E-23
KIFC1	0.467072225	1.813281674	1.956885488	3.63E-33	5.34E-32
HOTAIR	0.163085144	0.389775864	1.257019386	7.44E-12	1.60E-11
RPL36AP43	0.239485396	0.856996862	1.839352237	5.97E-18	1.90E-17
AC016405.3	0.288723805	1.655008079	2.519076294	2.74E-23	1.29E-22
SHISA6	0.834881871	0.265613266	-1.652244879	7.41E-24	3.66E-23
AC004771.5	0.166579987	0.343103246	1.042427693	7.45E-09	1.33E-08
CRYGS	0.298278892	1.537256569	2.365624178	1.01E-25	5.88E-25
L3MBTL4-AS	0.056628053	0.412246474	2.863918322	6.77E-38	3.81E-36
C6orf141	0.113380179	0.48558174	2.098545716	1.36E-05	1.99E-05
AC004908.1	0.538013468	1.27094802	1.240190835	2.92E-10	5.68E-10
CA2	150.9875032	57.10456339	-1.402751201	1.75E-32	2.27E-31
AL359182.1	0.148687713	0.472457208	1.667898235	1.18E-16	3.44E-16
AL596220.1	0.109025317	0.249927141	1.196844403	1.38E-08	2.43E-08
CTD-3080P12	0.535036427	0.251778657	-1.087481124	0.000574411	0.000750379
GUSBP2	0.137838934	0.424303461	1.622112997	1.21E-20	4.66E-20
HIGD1A	56.28117056	22.25637019	-1.338434013	1.69E-31	1.84E-30
AC048382.2	0.179138951	0.644482009	1.847061019	2.58E-15	6.91E-15
TRAV13-1	0.276330233	1.49609232	2.436733886	3.73E-19	1.29E-18
LMO3	1.48910374	0.14533913	-3.356949184	8.48E-38	4.58E-36
ACE	2.372841294	5.402977995	1.187139196	1.18E-15	3.24E-15
RPS26P28	0.097339975	0.238914634	1.295390917	0.001273762	0.001626712
AC004034.1	0.060675346	0.286162741	2.237653508	5.28E-18	1.69E-17
AC010530.1	0.078999766	0.286894834	1.860601703	1.46E-14	3.71E-14
LINC01239	0.105175219	0.272002355	1.370824319	4.15E-09	7.55E-09
KIF15	0.176386192	0.412722161	1.226433279	1.61E-23	7.78E-23
STAG3	0.255987608	0.641418715	1.325192474	2.30E-19	8.11E-19
AC007996.1	2.845346489	0.878342458	-1.695748897	2.25E-34	4.19E-33
FAM167A	7.074714942	0.626412058	-3.49748813	2.73E-39	2.70E-37
AC011899.2	0.091457122	0.624373049	2.771240845	2.25E-34	4.19E-33
AC018695.6	0.032841499	0.60090292	4.193540036	6.27E-24	3.12E-23
ARL4A	2.486525847	5.376916156	1.112647548	6.97E-27	4.46E-26
MSH5	0.160756603	0.771716197	2.263192391	3.27E-23	1.54E-22
PVT1	0.124390722	3.131098603	4.653718156	3.04E-42	1.36E-39
AL022316.1	0.202243716	0.585042507	1.532446573	7.35E-16	2.04E-15
THSD1	1.260970438	2.5754147	1.030270304	1.75E-14	4.43E-14
PLXNA4	1.030777932	0.183567644	-2.48934986	4.03E-33	5.89E-32
IRF7	3.246384853	10.86632612	1.742958307	1.53E-29	1.29E-28
LINC01389	0.156305425	0.522615172	1.741381159	4.61E-16	1.29E-15
LINC00707	0.160311446	1.100786842	2.779585792	1.53E-21	6.32E-21
AC011825.2	0.128156525	0.412288736	1.685748109	5.16E-11	1.05E-10
MIR616	0.694913239	1.405163316	1.015833047	0.024794481	0.028682855
SEZ6L2	4.18342761	20.48151179	2.291564832	3.70E-28	2.67E-27
MIR593	0.076113486	0.354390781	2.219117071	1.10E-09	2.08E-09
EIF4A1	0.425400621	1.931569211	2.182879328	2.49E-27	1.66E-26
CLDN14	3.549157783	0.457722301	-2.954932223	6.21E-37	2.45E-35
MTTP	2.230482404	0.655611941	-1.766441731	7.28E-26	4.28E-25
SYTL1	0.372823383	1.091240262	1.549404527	6.52E-11	1.32E-10
SNORA13	0.294125792	1.499149015	2.349638589	1.40E-10	2.78E-10
AC100803.2	0.47944809	1.33449457	1.476846907	1.12E-11	2.39E-11
GPD1	42.52470183	13.81676874	-1.62188086	3.88E-05	5.48E-05
SEPT5	1.023569332	3.39916611	1.731572038	2.08E-28	1.54E-27

Table S1. The differentially expressed genes(DEGs) were screened out in TCGA

ID	conMean	treatMean	logFC	pValue	FDR
AC012254.4	0.077551819	0.260368243	1.747320969	6.92E-09	1.24E-08
AC011726.3	0.127549163	0.288007693	1.175053915	3.15E-06	4.78E-06
LRRC2	3.562984708	0.32798995	-3.441362775	1.12E-39	1.36E-37
TRAV24	0.068604758	0.681325397	3.311963443	3.45E-16	9.77E-16
AL592211.1	0.077969001	0.24008405	1.622567013	1.34E-07	2.22E-07
PNP	43.28292014	15.98834704	-1.436777042	3.13E-35	7.19E-34
AC108463.3	0.034094906	0.455254239	3.739044354	1.54E-34	2.98E-33
ALOX12	0.42439849	1.025930398	1.273441429	2.06E-24	1.07E-23
GOLGA8VP	0.077264523	0.680351597	3.138402456	1.32E-34	2.61E-33
CYP8B1	5.545651242	1.998815599	-1.472211508	8.43E-08	1.41E-07
AC015922.2	13.50772403	6.351987822	-1.088504558	2.46E-26	1.51E-25
RPL7AP64	0.078852287	0.382058297	2.276568281	1.26E-23	6.13E-23
OGN	3.634924457	1.646940747	-1.142136731	6.32E-16	1.76E-15
TRAT1	0.215105315	1.13876781	2.404358538	2.03E-24	1.05E-23
AP001178.1	0.078271207	0.316856328	2.017275227	7.84E-12	1.69E-11
RNU6-553P	0.110007565	0.435183182	1.984020063	0.000130339	0.000178028
SNHG22	0.226258552	0.790850408	1.805432506	2.57E-14	6.45E-14
ARMH4	7.352378422	0.628665983	-3.547845416	1.16E-40	2.41E-38
RF00271	0.068700657	1.191303668	4.116073504	2.73E-16	7.77E-16
AC004000.1	0.040751675	0.331218312	3.022851188	1.36E-16	3.95E-16
COL4A1	47.82685222	175.3824953	1.874612015	1.08E-28	8.29E-28
ATP6V0A4	54.10487869	10.95331979	-2.304390493	8.07E-36	2.18E-34
RNU6-323P	0.095901231	0.285262768	1.572670227	2.14E-05	3.07E-05
NICN1	10.67157544	5.12770258	-1.057388687	1.13E-33	1.82E-32
AL606534.1	0.058091993	0.225937647	1.959513438	7.38E-18	2.33E-17
SEMA6B	2.768146275	8.425802233	1.605893874	1.53E-25	8.75E-25
HACD1	1.253044563	0.572055384	-1.131210987	1.67E-18	5.53E-18
AL592295.1	0.076919283	0.256702541	1.738680362	2.41E-28	1.78E-27
RPL36	68.96026042	159.0903759	1.206009436	1.69E-31	1.84E-30
CAPS	8.11307964	2.232825295	-1.861379277	1.58E-29	1.33E-28
PRAM1	0.231288849	1.287315347	2.476597886	2.63E-34	4.83E-33
AC078850.2	0.835273908	0.283142977	-1.560718628	4.58E-21	1.82E-20
MIR6719	0.041702235	0.409065868	3.294136564	6.78E-14	1.66E-13
AC016526.1	1.963628987	0.016483172	-6.896384648	9.07E-69	1.87E-64
AL135786.1	0.930436078	0.440130972	-1.079974141	1.43E-22	6.37E-22
ZNF121	1.772190925	3.544772564	1.000159026	1.92E-23	9.20E-23
AC004921.1	0.137649601	0.70423988	2.355066504	2.66E-34	4.86E-33
AC093591.1	0.128917485	0.272345404	1.078989574	1.56E-07	2.58E-07
LARS2	8.510364861	3.239198496	-1.393584107	2.69E-35	6.31E-34
AC008514.1	0.585409759	0.265199491	-1.142368791	3.72E-23	1.74E-22
CD300E	0.593308554	1.262101966	1.088973983	2.01E-13	4.77E-13
AL109811.3	1.022019646	2.404352882	1.234225724	1.57E-10	3.11E-10
EP300-AS1	0.400995168	0.860205447	1.101096416	2.14E-13	5.06E-13
PPIAP51	0.041375373	0.23971207	2.534458319	7.94E-11	1.60E-10
CXCL13	0.265823874	5.028927296	4.241708112	1.86E-26	1.14E-25
RNU6-1010P	0.155114885	0.955465163	2.622866133	5.09E-08	8.64E-08
CD163L1	0.458637876	1.333879941	1.540201412	1.34E-13	3.21E-13
NOP16	2.895474014	6.291194857	1.119534498	5.53E-33	7.87E-32
KC877373.1	0.12917878	0.656768932	2.346016782	4.68E-21	1.86E-20
PCED1A	9.694210139	19.58607128	1.014632779	1.30E-28	9.89E-28
GRAP	0.498581603	1.058952844	1.086736791	9.63E-19	3.24E-18
CKLF	2.729053529	5.792647343	1.085822144	2.45E-30	2.28E-29
AL021707.4	0.165215133	0.373676535	1.177444137	6.12E-08	1.04E-07
RRAD	11.97482158	42.68068958	1.833579325	2.57E-18	8.37E-18
TRIM29	0.742175035	0.333630763	-1.153507153	3.32E-16	9.40E-16

Table S1. The differentially expressed genes(DEGs) were screened out in TCGA

ID	conMean	treatMean	logFC	pValue	FDR
AC116533.1	6.67236825	17.8562086	1.420154968	5.86E-25	3.18E-24
RASAL1	9.719001458	4.640343723	-1.066576424	2.72E-09	5.01E-09
AC010273.2	0.79562018	0.142504719	-2.481070176	1.22E-27	8.37E-27
AQP7	4.311704508	2.12444437	-1.021172744	9.82E-19	3.30E-18
SERPINA5	93.15671236	2.961056878	-4.975475618	5.71E-42	2.26E-39
C8G	0.339359283	1.155214376	1.767275217	3.66E-09	6.69E-09
ADGRE5	8.435460417	18.32753712	1.119474208	4.42E-25	2.42E-24
AP000240.1	0.411010175	0.919047824	1.160965826	3.94E-09	7.18E-09
HOMER1	4.600688806	0.983313921	-2.226125903	3.77E-38	2.35E-36
TRIM7	0.233698838	0.645107282	1.464888541	2.20E-05	3.15E-05
FAHD2B	2.710464181	1.267267061	-1.096819354	4.10E-26	2.46E-25
C11orf21	0.091988453	0.598313302	2.701376461	4.95E-32	5.90E-31
AL023881.1	0.177253684	0.536215737	1.596997948	1.68E-18	5.54E-18
CD14	18.80520831	62.83544718	1.740446365	1.94E-27	1.31E-26
MICB	0.94848986	2.373876505	1.323540629	6.65E-29	5.23E-28
ABCB6	0.641922867	2.206010897	1.780968058	7.72E-33	1.07E-31
ABCD1	3.263849139	6.661305298	1.02923053	1.62E-31	1.77E-30
MIR590	0.547023436	2.027765338	1.890216158	3.00E-14	7.48E-14
C21orf58	0.21484195	0.528200215	1.297809167	1.98E-22	8.74E-22
FOXD2-AS1	0.512954235	1.517886158	1.565161571	2.09E-29	1.74E-28
LINC01786	0.196841294	1.039864447	2.401290667	1.06E-18	3.55E-18
AC073548.1	0.229846181	0.907966817	1.981970875	7.68E-17	2.27E-16
AC138649.2	0.131129049	0.407555681	1.636009858	4.74E-14	1.17E-13
AC068722.2	0.076708431	0.27382581	1.835801388	2.60E-14	6.52E-14
KCNJ4	0.126142146	0.388570157	1.623124724	1.15E-08	2.04E-08
EPHX2	37.34381275	14.85866984	-1.329564261	1.70E-26	1.05E-25
AL450311.2	0.039974938	0.496412706	3.634372335	2.31E-23	1.10E-22
BCL2A1	0.734244986	4.224732377	2.52452654	3.66E-31	3.80E-30
SPDYE6	0.133246409	0.267955036	1.007894272	2.92E-10	5.70E-10
RN7SKP239	0.116488179	0.352910214	1.599117628	8.87E-09	1.58E-08
CTAGE9	0.020096938	0.368114611	4.195107404	3.71E-29	2.99E-28
NLGN1	0.863635864	3.606112446	2.061949324	6.46E-28	4.55E-27
MTAPP2	0.046660908	0.266429886	2.513469627	3.51E-11	7.24E-11
IGHV1-58	0.889514594	3.047616159	1.776591029	0.024968837	0.028874819
AC021188.1	0.113572811	0.398757986	1.811895913	1.40E-24	7.36E-24
JMJD7-PLA2(	0.175024358	0.531440104	1.602351382	1.14E-13	2.75E-13
AC114316.2	0.029381612	0.339201182	3.529155736	3.10E-18	1.00E-17
RNU6-850P	0.522957382	2.393153533	2.194147673	1.51E-15	4.11E-15
AVPR1B	0.132594746	1.831644103	3.788043694	3.26E-26	1.98E-25
GJC1	0.734173093	5.01368415	2.771678966	2.36E-35	5.57E-34
AF111167.2	1.213197501	0.441720877	-1.457607508	3.43E-31	3.58E-30
HLA-DRB6	11.64693354	37.90984239	1.702622294	2.02E-20	7.67E-20
HMMR	0.416237058	1.063885127	1.353865063	1.69E-24	8.84E-24
ATP8B1	10.21866057	4.339966684	-1.235450232	7.04E-28	4.93E-27
UBTFL6	0.086737092	0.581771542	2.745731753	3.82E-27	2.50E-26
CKS1BP1	1.571136254	0.565092302	-1.47524986	5.19E-21	2.06E-20
SH2D4A	8.939492347	3.9428945	-1.180937798	3.83E-24	1.94E-23
AL031432.2	0.272858007	0.666660683	1.288802265	1.66E-05	2.41E-05
SIRPG	0.206470924	2.189064102	3.406303666	1.01E-29	8.72E-29
SNORA80D	0.134128016	0.463900331	1.790204263	0.007433859	0.008960672
TSPOAP1-AS	0.08763831	0.261557493	1.577494536	1.14E-21	4.75E-21
MIR6814	0.067683469	0.326414898	2.269831477	1.99E-08	3.46E-08
TYMP	4.304598149	33.13173079	2.944263104	4.69E-37	1.94E-35
NMI	3.739740986	7.611059127	1.025158875	1.52E-28	1.15E-27
DLX4	0.047157219	0.2955714	2.647956119	3.46E-24	1.76E-23

Table S1. The differentially expressed genes(DEGs) were screened out in TCGA

ID	conMean	treatMean	logFC	pValue	FDR
AC016737.2	0.131868446	0.315227121	1.257292269	3.57E-07	5.76E-07
AC011462.3	0.006300091	0.249144799	5.305468084	3.67E-10	7.10E-10
PLXNB3	0.12341833	0.500386446	2.019486028	8.29E-15	2.14E-14
RNU6-195P	0.095393936	0.290009791	1.604132139	9.56E-05	0.000131583
COX6B1P5	0.076609555	0.306002067	1.997945159	2.14E-13	5.06E-13
AC145422.1	0.091010783	0.32453466	1.834263173	3.36E-22	1.46E-21
DCST2	0.063241944	0.263138779	2.056870242	2.58E-21	1.05E-20
TSC22D3	35.17360778	78.12438941	1.151279689	2.71E-13	6.38E-13
HIST1H2BN	0.195867342	0.434498837	1.149475451	2.71E-22	1.19E-21
SNORA47	0.212425038	2.052478161	3.27234114	5.46E-06	8.17E-06
AL078604.1	0.101798503	0.264260881	1.376246532	0.000135016	0.000184221
AP002414.2	0.090934694	0.261578387	1.524340616	0.003249254	0.004021137
SREBF2	28.61649194	11.93171555	-1.262045335	1.42E-35	3.57E-34
CGN	13.68303333	2.540734984	-2.429070285	4.75E-39	4.02E-37
AL365181.3	0.45737711	2.678763996	2.550111412	1.72E-28	1.29E-27
RFX2	0.584435593	1.650532238	1.497815369	1.30E-26	8.13E-26
TEDC1	0.364369845	0.792476783	1.120965102	1.58E-18	5.23E-18
FOXC1	21.0564401	4.6665463	-2.173834433	3.95E-38	2.43E-36
BIRC5	0.401040877	2.170156096	2.435977619	9.38E-34	1.54E-32
CBR3	1.333108338	3.058347952	1.197958525	4.62E-18	1.48E-17
AC073912.1	0.053622199	0.230550012	2.104177437	1.20E-12	2.71E-12
AC107057.1	4.639346409	0.066037213	-6.13449853	3.24E-60	1.33E-56
AOX1	36.75379622	14.05488769	-1.386821345	5.09E-08	8.65E-08
AC069200.1	0.216003082	0.506278344	1.228878878	9.13E-15	2.35E-14
GBP1P1	0.7993546	2.322642102	1.538859324	5.24E-18	1.67E-17
AC022079.1	0.133258471	0.590490315	2.147686157	0.004796084	0.005860047
IGHV3-53	2.274194994	10.78057039	2.245005647	0.000946178	0.0012194
BHLHA15	0.221811852	1.214245792	2.452652126	2.64E-16	7.52E-16
STAG3L5P-P'	0.25937769	0.989454774	1.931579369	1.11E-18	3.73E-18
EOMES	0.180121103	2.13525873	3.567371772	1.25E-33	1.99E-32
CTSS	10.84665618	41.53150929	1.936955946	2.94E-29	2.40E-28
SERPINB9	3.436899518	9.028936257	1.393448353	1.78E-29	1.49E-28
RF01293	0.121500606	0.315041745	1.374579497	2.68E-08	4.64E-08
AC017002.3	0.067984681	0.342852448	2.334306212	4.16E-28	3.00E-27
PILRA	2.016723426	6.333978652	1.651098759	5.36E-30	4.78E-29
ATP5F1A	95.36683153	39.83353698	-1.259503998	1.78E-34	3.39E-33
LINC00672	0.235154671	0.530694902	1.1742727	1.28E-19	4.58E-19
C9orf92	0.134011949	0.294482057	1.135818093	2.41E-06	3.69E-06
RNU6-1280P	0.157872763	0.936596579	2.568665475	6.25E-13	1.44E-12
MYC	12.39717535	35.53526388	1.519239967	3.09E-23	1.45E-22
STC2	1.485266486	23.58730573	3.989216928	4.29E-38	2.62E-36
LRRC37A4P	0.697724151	0.324102468	-1.106206767	1.52E-17	4.70E-17
SAMD14	0.407015226	1.27276279	1.644808892	7.13E-30	6.25E-29
MGAT3-AS1	0.112894884	0.340259204	1.59165408	4.19E-11	8.60E-11
AC010655.2	0.069154883	2.866902601	5.373517969	5.41E-38	3.17E-36
AC142381.1	0.125853967	0.476533867	1.920828059	0.010286533	0.012255029
AC011753.4	0.258602439	0.652233441	1.334652532	4.73E-07	7.57E-07
BMP5	0.843819914	0.349110677	-1.273250655	9.95E-07	1.56E-06
AP003390.1	0.138547098	0.289435604	1.062865913	1.19E-07	1.97E-07
RN7SL81P	0.124909661	0.595919181	2.254231616	4.58E-17	1.37E-16
PKP1	0.395648573	1.676896228	2.083501953	0.015341231	0.018015365
PCDHGA1	0.137991355	0.360742152	1.386390128	7.55E-11	1.52E-10
AC005785.1	0.121053028	0.610457021	2.334250555	9.01E-29	6.97E-28
AC245014.3	0.130700251	0.309988046	1.245950677	2.20E-06	3.37E-06
C17orf49	0.301929227	0.609863672	1.014276362	1.15E-25	6.65E-25

Table S1. The differentially expressed genes(DEGs) were screened out in TCGA

ID	conMean	treatMean	logFC	pValue	FDR
CSF3R	0.361273153	2.497306914	2.789211182	4.99E-36	1.42E-34
TTLL3	0.590351371	1.842802471	1.642255646	5.53E-13	1.28E-12
U62317.4	0.056263419	0.352408609	2.646980041	7.80E-20	2.85E-19
AL355488.1	0.557591263	1.825068095	1.710670434	1.18E-16	3.45E-16
UBE2Q1-AS1	0.096730846	0.431441603	2.157117384	6.45E-19	2.20E-18
PIGR	126.4306192	45.10173928	-1.487090925	5.71E-24	2.86E-23
AP001029.1	0.054674511	0.544214524	3.315235132	3.22E-17	9.75E-17
IGSF3	13.53368979	6.129332416	-1.142753371	9.39E-32	1.06E-30
RAB25	24.559332	1.952179182	-3.653113938	4.41E-37	1.84E-35
CASR	24.75163581	0.537988906	-5.523803641	1.70E-40	3.24E-38
GFOD1	0.884287365	1.907179594	1.108853524	3.86E-14	9.57E-14
SNHG25	0.347610737	1.310574896	1.914655252	1.77E-16	5.10E-16
LY6K	0.556455536	0.21208346	-1.3916343	2.72E-26	1.66E-25
KLRA1P	0.310575245	1.112915896	1.841329826	4.38E-23	2.04E-22
ANKRD23	0.076018372	0.238487592	1.64949417	7.76E-19	2.62E-18
HSPB7	14.64025027	1.421711648	-3.364239424	4.29E-34	7.58E-33
SCARNA12	0.111366675	2.97258363	4.738327904	2.28E-18	7.46E-18
ZNF436-AS1	0.579544875	1.294033553	1.158882745	4.77E-18	1.53E-17
NADK2-AS1	0.219760282	0.472340478	1.103896508	3.58E-08	6.15E-08
AC073115.2	0.025104265	2.518519716	6.648499744	1.11E-33	1.79E-32
ALAD	26.21698472	11.20367232	-1.226530074	1.69E-32	2.21E-31
SPARCL1	54.94232603	168.6541579	1.618077991	5.60E-21	2.22E-20
LINC01230	2.447812963	0.748823498	-1.708795714	2.60E-39	2.61E-37
RNU6-282P	0.216498194	0.679551388	1.650227659	1.79E-08	3.13E-08
COL11A1	0.110262257	1.165161854	3.401519426	0.000391286	0.000516948
MRPL33	34.77965833	16.2979883	-1.09354986	2.57E-37	1.18E-35
RNA5SP160	0.083272469	0.280885895	1.754072677	0.004215928	0.005172985
GOLGA8M	0.063319622	0.265518763	2.068089267	1.36E-16	3.95E-16
CDCA2	0.30070131	5.890745275	4.292047113	1.41E-38	1.01E-36
AC026471.4	1.584314883	3.727187667	1.234228361	4.03E-18	1.30E-17
WT1	5.661493042	0.999446581	-2.501981205	1.21E-19	4.35E-19
AC116158.1	0.07993446	0.236465109	1.564737838	8.70E-09	1.55E-08
AC104667.2	1.149316144	0.400936751	-1.519329128	1.63E-26	1.01E-25
AL356299.2	0.079288104	0.278488203	1.812439889	1.63E-12	3.65E-12
AC010168.2	0.390521513	0.900633931	1.205538806	9.29E-12	1.99E-11
AL356361.2	0.202588884	0.41618998	1.038687216	0.004833105	0.005904929
PRC1	0.993881574	2.532202408	1.349246867	3.81E-30	3.47E-29
TRGJP2	0.115550072	0.363523907	1.653532084	4.30E-06	6.47E-06
ANKK1	0.062672516	0.292614868	2.22309826	3.62E-23	1.69E-22
AC011479.1	0.53367501	0.253684763	-1.072924586	6.04E-18	1.92E-17
DCLK1	0.495332021	2.182194352	2.139311805	6.89E-25	3.72E-24
KCNMB1	0.721313458	1.622350237	1.169387058	5.49E-19	1.88E-18
AC022306.3	0.252977245	0.55730951	1.139471148	1.03E-11	2.19E-11
BRICD5	0.515928306	1.117340702	1.114826657	1.24E-05	1.82E-05
AC016737.1	0.114970535	0.346130068	1.590050104	4.40E-10	8.47E-10
TBX19	0.492261961	1.488782934	1.596635256	7.11E-31	7.10E-30
KCCAT198	0.281170306	2.621190686	3.220706163	3.28E-28	2.39E-27
HIBCH	12.27786082	5.346094034	-1.199502102	3.47E-20	1.30E-19
AC090152.1	1.603297864	0.564576993	-1.505800233	3.26E-28	2.37E-27
FGFR2	11.88838857	4.92208734	-1.272211013	9.34E-28	6.45E-27
GAB3	0.56600116	1.789048151	1.660315303	3.06E-33	4.55E-32
LURAP1	3.753048014	1.096671007	-1.774931954	3.87E-34	6.91E-33
AC092755.1	0.08794661	0.260835793	1.568441989	5.58E-05	7.80E-05
AP003170.3	0.1200589	0.445710867	1.892365784	7.08E-10	1.35E-09
CTDSPL	24.56505213	10.71440904	-1.197055021	7.53E-36	2.06E-34

Table S1. The differentially expressed genes(DEGs) were screened out in TCGA

ID	conMean	treatMean	logFC	pValue	FDR
U2AF1L4	1.304752518	2.900273057	1.152412548	2.47E-16	7.06E-16
CACNA2D2	1.648251247	0.340372415	-2.275750149	2.08E-39	2.19E-37
COL5A2	4.043147458	16.81573731	2.056261311	1.72E-28	1.29E-27
U91328.3	0.17870165	0.427265766	1.257580771	3.44E-07	5.54E-07
AC016586.1	0.119978526	0.317253471	1.40285974	2.41E-08	4.17E-08
HLA-C	265.9387185	677.5405253	1.349213403	1.28E-32	1.71E-31
LINC02585	0.269198933	0.702718029	1.384273221	8.69E-11	1.75E-10
AC010378.1	0.047632456	0.414771367	3.122299462	1.21E-15	3.32E-15
HMGN2P47	0.155827437	1.330675826	3.094137966	1.03E-23	5.02E-23
CBLC	4.100831594	0.988518325	-2.052576883	3.19E-34	5.78E-33
KLRC1	0.061161691	0.233621995	1.933475905	1.68E-20	6.45E-20
AC108463.1	0.698628984	3.719427627	2.412482224	5.56E-35	1.21E-33
AL157834.1	0.108922803	0.254612033	1.224994591	0.005065048	0.006176215
BIRC7	0.071508884	9.151525041	6.999745885	4.94E-32	5.88E-31
PIEZO2	0.508548284	2.37200824	2.221652358	4.78E-27	3.10E-26
RN7SL648P	0.02225423	0.683920905	4.941677999	4.28E-13	9.96E-13
AC099791.2	0.058577733	0.233224535	1.993295292	5.99E-10	1.14E-09
BBIP1P1	0.0951179	0.246963311	1.376507963	0.00019034	0.000256999
ATF3	46.77667497	20.46374967	-1.192718793	1.17E-09	2.20E-09
AL035446.1	0.139378666	0.699667281	2.32765928	2.69E-17	8.21E-17
MYOT	0.235480579	0.666819881	1.501689037	3.93E-06	5.93E-06
AC000078.1	0.103484055	0.400117095	1.951013777	4.23E-17	1.27E-16
CDHR3	0.469942198	1.335962468	1.507324254	8.92E-25	4.77E-24
SNX22	0.306342049	0.730311745	1.253369027	1.21E-21	5.04E-21
AL159169.2	0.141663383	0.4179915	1.561006702	2.31E-14	5.79E-14
SCARF1	2.366696394	7.057962935	1.576377208	7.62E-29	5.94E-28
ZG16B	0.118213526	0.254448019	1.105975842	1.50E-08	2.64E-08
MIR155HG	0.252438202	2.736779781	3.438477195	5.68E-33	8.03E-32
AGAP12P	0.116816954	0.427128794	1.87042149	2.72E-06	4.14E-06
RHOH	0.232214088	1.114850157	2.263322408	2.23E-28	1.65E-27
IGLV1-36	1.152643858	4.21611198	1.870966366	0.000194511	0.000262442
LINC00265	0.58954685	1.234460668	1.0662025	1.47E-07	2.43E-07
SIM2	5.444617059	0.239558702	-4.506379457	1.10E-39	1.35E-37
IGKV5-2	0.547297696	5.30014141	3.275633164	0.000453944	0.000597023
AC112907.2	0.535356871	0.223415455	-1.260771929	6.14E-23	2.83E-22
MAP4K4	5.792544083	13.50943053	1.221697838	1.42E-31	1.56E-30
MIR4292	0.837193114	2.560691853	1.612901303	5.44E-13	1.26E-12
RTP4	3.341271958	11.26588039	1.753490741	6.81E-27	4.36E-26
C2orf40	9.485873918	2.445620058	-1.955580403	1.89E-24	9.82E-24
MPPED2	3.329367488	0.576930047	-2.528779814	6.99E-38	3.89E-36
SLC4A8	0.825652725	0.128299124	-2.686023779	7.99E-30	6.94E-29
AC092375.2	0.07211339	0.279618851	1.955122556	3.10E-14	7.73E-14
AC008554.1	0.809076076	1.642379152	1.021439951	3.42E-09	6.25E-09
AP002490.1	0.115635287	0.305464432	1.401422691	1.63E-15	4.43E-15
FUT3	2.890358762	0.618730985	-2.223864387	3.12E-13	7.32E-13
MST1L	5.617494215	0.833366217	-2.752904212	2.98E-33	4.43E-32
AC092919.2	0.267139227	0.568344674	1.089174281	5.72E-17	1.70E-16
PBX1	7.006295639	2.743029466	-1.352881746	6.62E-33	9.26E-32
BUB1B	0.386532038	0.950255533	1.297727523	1.33E-23	6.45E-23
AC141002.1	0.183337668	0.541174402	1.561590372	6.20E-14	1.52E-13
KITLG	21.52851915	6.954415999	-1.630247813	3.34E-31	3.50E-30
NAALADL2	1.875741896	0.504957008	-1.893228859	1.00E-38	7.50E-37
AP001381.1	0.026085639	0.229153372	3.134985826	8.46E-13	1.93E-12
AC087277.2	0.046342678	0.226936451	2.291875054	4.83E-07	7.73E-07
SVIP	11.43453382	5.692939156	-1.006151963	1.08E-33	1.75E-32

Table S1. The differentially expressed genes(DEGs) were screened out in TCGA

ID	conMean	treatMean	logFC	pValue	FDR
WNT9B	3.030977214	0.160114388	-4.242608143	7.69E-41	1.82E-38
AC136475.10	0.280333681	1.361817251	2.280316123	1.68E-23	8.11E-23
AC079310.1	2.567111602	0.005007551	-9.00182507	3.58E-65	1.84E-61
AC114402.1	0.085288147	0.416867369	2.28917128	2.62E-15	7.03E-15
AC079203.1	0.124592013	0.429984928	1.787074497	4.62E-12	1.01E-11
MEF2C	3.684021653	9.267532839	1.330903781	2.35E-24	1.21E-23
AJM1	0.428488755	1.840859533	2.103050298	1.59E-35	3.96E-34
IGHA2	13.1693102	57.92937063	2.137115211	1.60E-08	2.81E-08
TMEM61	8.100909502	0.931356391	-3.120678653	7.55E-36	2.06E-34
STAMBPL1	0.455687776	2.585089948	2.504096903	1.59E-36	5.35E-35
CYP2D6	0.186867523	0.527322535	1.496669796	1.65E-06	2.56E-06
IGHV1OR15-(	0.218250844	0.881339321	2.013710341	0.000182888	0.000247212
AC026979.3	0.31732303	0.650743349	1.03613643	2.90E-17	8.83E-17
TDRD9	1.368749538	0.671781802	-1.026793857	3.74E-20	1.39E-19
MZT2B	14.92501633	32.29747328	1.113688793	8.54E-25	4.57E-24
AL162274.1	0.059669646	0.455871669	2.933558634	7.55E-30	6.58E-29
C16orf54	0.588778888	2.061638855	1.807993787	2.51E-25	1.40E-24
RNVU1-3	0.159660863	0.746680368	2.225480087	1.34E-13	3.22E-13
LINC02348	0.133107015	7.995378152	5.908507757	1.23E-35	3.15E-34
SULF1	6.141353536	13.40203291	1.125823294	2.94E-10	5.73E-10
AC079250.1	0.468076097	1.07010001	1.192930636	2.43E-21	9.92E-21
C1QTNF1	5.973205875	13.32443387	1.157496885	1.59E-11	3.35E-11
CDKN3	0.414006472	1.510654293	1.867448318	1.85E-31	2.01E-30
CARMIL2	0.089130701	0.515061262	2.530749673	4.72E-28	3.37E-27
AC019206.1	0.030126716	0.224953746	2.900513069	1.02E-15	2.80E-15
DDN	8.607555135	0.110987796	-6.277130564	1.65E-17	5.09E-17
ZP3	0.67016821	1.945932893	1.537866801	7.81E-19	2.64E-18
IGLV1-40	13.55344794	177.9843242	3.715018364	3.31E-07	5.35E-07
MAN1C1	7.811620074	1.671384156	-2.224578419	1.08E-36	3.89E-35
MYBPC2	0.136365314	0.274356372	1.00857436	0.002699977	0.003359966
KRT86	0.244828783	0.700315552	1.516231951	8.05E-13	1.84E-12
AL136295.2	0.186265177	0.643394003	1.788344508	2.34E-18	7.64E-18
RIN1	0.472756317	1.888164688	1.997815964	6.36E-30	5.62E-29
ZNF296	0.322364889	0.926108443	1.522486518	1.44E-24	7.57E-24
RN7SL827P	0.038007997	0.279570986	2.878839735	0.00130917	0.001670686
RPL18AP3	6.639690333	13.73166218	1.048318407	1.01E-16	2.97E-16
AC005262.1	0.024696438	0.26332904	3.414491723	2.96E-11	6.14E-11
RRM2P3	0.170691252	0.576209689	1.755204794	1.61E-12	3.60E-12
CERS3-AS1	0.046829572	0.261087029	2.479039023	2.38E-16	6.81E-16
AP001893.1	0.142670722	0.373387435	1.387984075	1.55E-09	2.90E-09
GABRD	0.240042364	8.697100451	5.179173548	1.67E-40	3.21E-38
CD207	0.311498513	0.872375333	1.485723701	1.06E-09	2.00E-09
NARF-IT1	0.09580311	0.423671537	2.144801808	1.37E-17	4.26E-17
AC090578.2	0.058568884	0.371912978	2.666758789	1.14E-09	2.14E-09
RN7SKP287	0.08254428	0.231892563	1.490216396	6.45E-06	9.59E-06
TAS2R6P	0.06481485	0.280608522	2.114162522	2.10E-19	7.42E-19
AL138689.1	0.114618391	0.358602639	1.645547558	4.00E-08	6.84E-08
AC004771.1	0.128572671	0.534126832	2.05459834	1.76E-23	8.46E-23
RNU6-767P	0.045716456	0.284108972	2.635658932	6.59E-06	9.79E-06
REELD1	0.114162678	0.6019447	2.398539873	2.47E-15	6.61E-15
C8orf34-AS1	0.53785169	0.212104788	-1.342431227	1.03E-07	1.71E-07
TTC34	0.15396174	0.409779575	1.412276193	9.11E-14	2.20E-13
HCG27	0.202245276	1.578450712	2.964331305	3.72E-32	4.51E-31
AC007014.2	0.085436667	0.240164737	1.491097064	0.001873956	0.002360561
TET3	1.00310026	2.081579382	1.053212767	1.40E-27	9.54E-27

Table S1. The differentially expressed genes(DEGs) were screened out in TCGA

ID	conMean	treatMean	logFC	pValue	FDR
LDLRAD2	0.427261588	0.978568471	1.195553182	9.06E-16	2.50E-15
ENPP7P4	0.054145798	0.221583111	2.032926627	1.78E-15	4.81E-15
STBD1	0.658204442	1.337823978	1.023280641	1.20E-22	5.37E-22
THSD4	5.258344167	1.327032486	-1.986404883	1.57E-35	3.90E-34
ACY1	8.587370308	1.968624897	-2.12502816	2.08E-14	5.23E-14
AC022146.2	0.071906969	0.365103135	2.344100553	4.75E-08	8.09E-08
AC017083.1	0.119154114	0.28328898	1.249445718	8.72E-07	1.37E-06
LOH12CR2	2.218925494	0.832062664	-1.415097137	7.67E-36	2.09E-34
ABCA17P	0.073018171	0.397766159	2.4455931	4.36E-26	2.61E-25
STX4	6.536388778	14.75849637	1.174980043	1.79E-36	5.86E-35
SLC25A48	1.683597485	0.839941552	-1.003186414	6.45E-14	1.58E-13
GGT3P	0.109141111	0.382796071	1.810381392	5.51E-05	7.70E-05
AC120349.1	0.091197574	0.461079749	2.337948952	2.76E-06	4.20E-06
KIF21B	0.185122881	0.835087992	2.173445003	6.06E-32	7.13E-31
AP000439.2	0.151633392	11.75354171	6.276364246	6.46E-31	6.48E-30
MTURN	43.96015165	5.315204534	-3.047999249	5.26E-40	7.56E-38
AC060780.2	0.298835864	0.825658009	1.466191034	1.60E-11	3.37E-11
CASC2	0.802866151	0.375632532	-1.095837478	8.16E-34	1.36E-32
CD300LF	0.324951005	2.637871036	3.021079917	1.03E-37	5.39E-36
HOXD10	10.65641832	4.208876471	-1.340215549	9.20E-26	5.35E-25
AC040162.1	0.1188522	0.524543404	2.141893542	2.67E-31	2.83E-30
ADAM12	0.474845348	1.693797274	1.834731586	5.60E-12	1.21E-11
AC009237.15	1.808554378	0.679507988	-1.412274561	2.71E-17	8.27E-17
ZNF812P	0.265407519	0.801803052	1.595038667	8.18E-14	1.99E-13
SNORA22	0.125396237	1.770395218	3.819505502	2.91E-12	6.43E-12
RRN3P1	0.370900363	1.238297516	1.739254398	9.34E-26	5.43E-25
RFLNB	5.885368681	16.43264048	1.481359619	8.12E-23	3.69E-22
C20orf204	0.049870941	0.221980375	2.154160794	1.24E-29	1.06E-28
RF00586	0.102581515	0.443255314	2.111367149	2.09E-15	5.63E-15
AP000525.1	0.029578894	0.315738456	3.416089953	7.40E-25	3.98E-24
AC055822.1	0.68425456	1.398370649	1.03114176	3.80E-08	6.52E-08
AL135818.1	0.11022747	0.382355217	1.794429758	3.36E-24	1.71E-23
XCL2	0.332617371	1.964680643	2.562359401	1.73E-27	1.17E-26
CD72	0.362359888	2.408438665	2.732603018	1.21E-36	4.31E-35
IGHV3OR16-[illegible]	0.139533964	0.593129785	2.08773149	0.001259202	0.001609016
GRIK5	3.975263275	0.47426275	-3.067291946	1.26E-35	3.23E-34
IGLV1-47	6.388097263	36.16840818	2.50127192	5.42E-07	8.63E-07
HVCN1	1.153553765	3.074663402	1.414343233	4.40E-31	4.51E-30
BCAS1	1.135993156	0.265547225	-2.096913782	1.25E-27	8.52E-27
IQCH	0.771928465	0.350523242	-1.138957052	9.79E-32	1.10E-30
AURKB	0.239296783	1.492426949	2.64078741	1.21E-34	2.41E-33
ASPHD2	3.037128517	1.506172095	-1.011821336	8.10E-15	2.10E-14
IDNK	9.539671139	4.362789364	-1.128688711	1.70E-21	7.04E-21
AC026470.2	0.087652962	0.2343489	1.418783268	0.000395277	0.000522099
AC023510.2	0.389696731	0.822939011	1.078433686	2.93E-09	5.38E-09
AP001542.1	0.130251227	0.557855375	2.098594187	8.33E-10	1.58E-09
AC092802.2	0.098922764	0.273020706	1.46463592	2.41E-13	5.69E-13
KLF8	1.073093207	2.580757368	1.26601912	5.49E-21	2.17E-20
RIMKLA	0.73349814	3.772760422	2.362755276	2.25E-29	1.86E-28
HIST1H2BJ	0.334983721	0.77177479	1.204088929	8.13E-07	1.28E-06
PRIMA1	1.306181793	10.53584956	3.011879042	4.16E-07	6.68E-07
AC010320.3	0.103723629	0.278460344	1.424727301	4.27E-10	8.23E-10
DDX11-AS1	0.094439867	0.214951235	1.186541481	2.43E-20	9.15E-20
SLC16A8	0.098107919	0.426491983	2.120077126	2.98E-21	1.21E-20
CYP2C8	0.410565796	1.538445777	1.90578825	6.34E-11	1.28E-10

Table S1. The differentially expressed genes(DEGs) were screened out in TCGA

ID	conMean	treatMean	logFC	pValue	FDR
TLR10	0.142757554	0.527868606	1.886611782	3.06E-20	1.15E-19
UGT3A1	12.02704756	4.955951983	-1.279048413	4.07E-21	1.63E-20
AL031600.1	0.195917986	0.820075824	2.065507457	1.04E-12	2.35E-12
FAM198B-AS	0.162115403	0.357786613	1.142078241	3.01E-10	5.86E-10
AKR7A3	27.92207006	9.749449963	-1.51801317	7.07E-11	1.43E-10
CPA3	2.487805381	6.519299786	1.389843389	2.64E-15	7.07E-15
TMEM255B	0.968235996	2.804660538	1.53439553	5.51E-26	3.27E-25
AC108010.1	0.899406101	1.824884558	1.020760626	4.00E-10	7.71E-10
IGHV3OR16-	0.145689188	0.784173812	2.428279652	0.000151861	0.000206424
AP000688.2	2.14459959	0.75867584	-1.499152812	5.90E-22	2.52E-21
RPL35P5	1.347575649	2.709464708	1.007641592	2.66E-13	6.28E-13
LINC01569	1.178833046	2.364011198	1.003877461	5.50E-15	1.44E-14
SNRPGP4	0.252143734	0.976330704	1.953123526	2.28E-05	3.27E-05
AC083837.1	0.074267851	0.244609852	1.719672775	3.17E-20	1.19E-19
CDKN2A	0.063563294	1.927830869	4.922640777	7.21E-42	2.76E-39
MXD3	0.167789047	0.994298896	2.567031061	7.34E-36	2.01E-34
MIR1270	0.414501792	4.529858341	3.450015692	1.62E-25	9.23E-25
AP002807.1	0.311513802	0.848759094	1.446059045	3.83E-09	6.97E-09
C3orf52	2.357702238	0.598380986	-1.978245289	2.58E-31	2.74E-30
LINC02061	6.409707789	0.701613794	-3.19150957	8.42E-20	3.06E-19
ST8SIA4	0.6926729	8.452596733	3.609148485	2.24E-39	2.33E-37
TRGV10	0.161556216	1.184493175	2.874161723	1.45E-29	1.23E-28
AC073210.1	0.155735206	0.368908534	1.24416804	8.03E-05	0.00011106
ERCC6L	0.086382909	0.250520941	1.536113393	1.58E-30	1.51E-29
AC027514.2	0.12401347	0.317380409	1.355716248	3.51E-07	5.66E-07
NFE2L3	3.722489306	9.644068239	1.373374155	2.68E-22	1.17E-21
AP5M1	6.512073167	3.12324117	-1.060072934	3.24E-36	9.67E-35
FGF14-AS2	6.540842881	2.856416962	-1.195269969	1.39E-27	9.47E-27
UBE2L6	25.02473708	53.18577449	1.08768551	1.31E-30	1.26E-29
VWA5B1	0.702445221	0.193473568	-1.860249244	1.81E-36	5.92E-35
PTPRJ-AS1	0.030315566	0.556486641	4.198216412	8.05E-05	0.000111392
HCP5	9.270478486	22.60989337	1.286238479	6.51E-28	4.58E-27
GBP4	10.09476213	24.64491333	1.287682992	7.02E-16	1.95E-15
LINC00996	0.069649995	0.226331137	1.700239918	1.16E-17	3.63E-17
FCHSD1	1.168174426	2.889019307	1.306324139	4.51E-23	2.10E-22
AC040162.3	0.083759987	0.390618159	2.22142589	2.31E-25	1.30E-24
SLC35G2	1.014886201	2.726154997	1.425549622	7.62E-29	5.94E-28
CCL28	2.527795814	7.263620979	1.522808991	1.43E-15	3.90E-15
AL021707.7	0.123280223	0.576657021	2.225772127	5.35E-17	1.60E-16
KCNK13	5.168706111	0.823880061	-2.649296941	2.35E-39	2.42E-37
AC005281.1	1.762453478	0.262359388	-2.747968855	8.05E-35	1.67E-33
LTB4R	0.519578019	3.04305765	2.550109361	4.49E-38	2.70E-36
AC243829.1	0.027803995	0.295618653	3.410373207	4.57E-21	1.82E-20
CNR1	0.120202125	0.394957521	1.716235091	2.02E-14	5.09E-14
CYFIP2	87.888915	18.27109236	-2.266118327	5.17E-36	1.47E-34
FZD1	9.894522569	24.27144524	1.294558015	2.28E-25	1.28E-24
ZNF32-AS2	0.396491139	0.935665438	1.23870414	8.30E-13	1.90E-12
HMGA1P4	0.423841251	0.952360644	1.167983997	0.000448726	0.000590354
SCN1B	1.072960089	4.328219516	2.012177258	1.92E-35	4.66E-34
ANKRD36C	0.100477279	0.357829696	1.832403812	2.54E-16	7.27E-16
SNORA75	0.166460722	1.0910353	2.71244408	1.27E-16	3.70E-16
TREM2	1.409428622	23.41532467	4.054270721	4.52E-40	6.84E-38
XBP1P1	0.09655031	0.278844265	1.530106798	1.99E-12	4.44E-12
LILRB2	0.712980169	3.528278768	2.307030696	3.02E-35	6.99E-34
AC132872.3	0.365955634	2.019289163	2.464106857	1.40E-21	5.80E-21

Table S1. The differentially expressed genes(DEGs) were screened out in TCGA

ID	conMean	treatMean	logFC	pValue	FDR
ZDHHC11	0.162525058	0.454670964	1.484160709	4.27E-07	6.85E-07
AC027288.3	1.896463498	0.285271068	-2.73290626	6.41E-20	2.35E-19
FGF14	0.145720211	0.318787333	1.12939332	0.031064472	0.035611968
TAS2R2P	0.527392786	0.173025386	-1.607894113	2.94E-25	1.63E-24
MYO6	27.24522153	11.78605164	-1.208922729	2.18E-38	1.46E-36
AC012186.2	0.147331077	0.460884319	1.645342912	1.54E-14	3.93E-14
IL16	0.589597089	2.210966514	1.906875869	8.63E-32	9.84E-31
CEACAM4	0.149628548	0.537953748	1.846096683	5.22E-21	2.07E-20
LINC01094	0.277250445	1.963631265	2.824262361	6.62E-37	2.57E-35
RNA5SP118	0.08206058	0.315622291	1.943437839	1.95E-06	3.00E-06
QTRT1	5.345294319	12.21137121	1.191883917	1.33E-26	8.30E-26
MS4A2	0.216930038	0.561876675	1.373023679	6.51E-16	1.81E-15
LRRK1	0.686337219	2.153168658	1.649471832	1.55E-29	1.31E-28
LYNX1	4.687950375	2.026407721	-1.210032819	3.50E-26	2.11E-25
CSF1R	7.452381472	23.2148752	1.639276095	1.55E-24	8.13E-24
LINC00113	2.020253757	0.859227137	-1.233425052	1.30E-16	3.78E-16
RNU2-27P	4.004503504	1.416726529	-1.499062081	2.50E-19	8.79E-19
HIST2H2BE	14.70718447	6.366502967	-1.207948043	9.01E-15	2.32E-14
RPL21P119	0.351830885	0.760759171	1.112557686	4.63E-08	7.88E-08
AC147651.1	5.260377449	11.1339188	1.081723241	3.19E-06	4.84E-06
AGBL3	0.531623663	1.583935244	1.57503613	4.10E-26	2.46E-25
AC134043.2	0.388085373	0.959787159	1.306340453	2.40E-20	9.04E-20
TSPAN1	148.5260861	66.62756024	-1.156525367	3.97E-21	1.59E-20
IRF1	5.463305597	12.01400087	1.136870644	3.27E-20	1.22E-19
AC012618.3	1.197002397	0.508110573	-1.236211651	4.49E-26	2.69E-25
EPB41L4A-A:	4.630928222	12.26937211	1.40568812	7.10E-28	4.97E-27
FAM183A	0.455262274	1.577635122	1.792993756	1.70E-10	3.37E-10
STMN2	0.045156298	0.641166603	3.827700156	1.31E-07	2.17E-07
CCDC13-AS1	0.52312596	0.160503533	-1.704553314	1.49E-30	1.43E-29
RNF175	0.083770215	0.437738403	2.385559674	6.22E-32	7.30E-31
SPTBN2	32.95805167	1.476465481	-4.480411427	5.84E-40	8.06E-38
PRKAR2B	7.447831625	1.567726298	-2.24814675	3.09E-40	4.86E-38
SFXN3	8.174946556	19.44758902	1.250310107	4.93E-34	8.57E-33
RPL21P10	0.088549415	0.265379354	1.583501459	6.69E-12	1.44E-11
IGHV3-41	0.145657728	0.367832543	1.336466871	0.014387	0.016937303
ARNT2	16.22907947	4.725729053	-1.779972351	4.33E-34	7.64E-33
IGLV1-50	0.15383399	0.667502541	2.117399024	0.000311541	0.000414641
CADM1	10.31925156	3.99332561	-1.36967572	2.79E-25	1.55E-24
AC245100.4	0.338447143	1.109618612	1.713061447	9.09E-21	3.54E-20
IGKV1D-12	0.143719423	0.569367598	1.986105347	0.031252317	0.035821328
FCGR2A	2.956442069	10.95811033	1.890065121	2.18E-30	2.04E-29
AL024508.2	0.603267806	0.176453586	-1.77350984	9.03E-35	1.85E-33
MIRLET7D	0.779391415	2.252634653	1.531193401	8.97E-06	1.32E-05
PIM2	4.676008986	11.31879483	1.275370747	6.40E-19	2.18E-18
AL158212.2	0.213800664	0.436791174	1.030677369	0.001096527	0.001406211
SCART1	0.070071317	0.347844668	2.311547278	8.01E-19	2.71E-18
SPTB	0.891068136	0.146329619	-2.606313938	3.19E-38	2.04E-36
ITPR2	9.920941875	4.310921658	-1.202480749	7.03E-33	9.77E-32
B3GNT4	0.467202585	1.644622184	1.815636032	2.06E-16	5.90E-16
IGKV1D-43	0.095152802	0.647564283	2.766705364	0.009653354	0.011530707
GPR84	0.148641791	0.633879364	2.092368515	2.24E-21	9.16E-21
GRPR	0.070903849	0.27461279	1.953462976	0.000110741	0.000151846
ADAM1B	0.651020824	0.269499461	-1.272421302	2.47E-30	2.29E-29
PRSS27	0.124396594	0.283700603	1.189422231	2.41E-16	6.90E-16
AC068888.2	0.066524203	0.282294995	2.085252315	8.44E-14	2.05E-13

Table S1. The differentially expressed genes(DEGs) were screened out in TCGA

ID	conMean	treatMean	logFC	pValue	FDR
AC006449.5	0.54046313	0.182564402	-1.565790715	4.03E-33	5.89E-32
IKZF3	0.331399566	2.030108258	2.614913046	1.80E-28	1.35E-27
MIR581	0.065399915	0.539247768	3.043587627	6.00E-06	8.95E-06
AC008991.1	0.032605993	0.525439788	4.010316373	2.99E-14	7.47E-14
AC093063.1	0.006020191	0.445473202	6.209385683	3.70E-18	1.19E-17
TNFRSF17	0.223611994	0.678031667	1.600355082	1.98E-07	3.24E-07
EPHB2	1.353783521	0.657578499	-1.041762029	3.37E-19	1.17E-18
SNORA80E	0.135121741	0.809366227	2.582532826	0.00136161	0.001735779
BCL9	6.666360319	3.256424153	-1.033610673	2.11E-36	6.72E-35
AC000068.1	0.584852053	0.184171528	-1.667021678	1.63E-34	3.14E-33
CD5L	0.038950162	1.215745921	4.964068607	2.45E-21	1.00E-20
GZMH	0.977967646	7.902790277	3.01450348	5.56E-35	1.21E-33
AC068790.6	0.07655601	0.303491126	1.987066788	1.74E-08	3.05E-08
UXS1	19.94383839	7.255615106	-1.458773266	1.02E-34	2.06E-33
CRNDE	2.506750071	6.166878792	1.298722328	3.48E-17	1.05E-16
SNCB	0.024548203	0.403611708	4.039278705	2.49E-08	4.31E-08
RASSF4	6.40596505	21.56521928	1.751218554	1.62E-30	1.55E-29
HIST1H2BO	0.051967681	0.356193988	2.776976586	2.87E-14	7.18E-14
MATK	0.298408072	1.447925052	2.278628459	2.25E-34	4.19E-33
AC145207.9	0.166395745	0.952999761	2.51785731	1.10E-19	3.96E-19
HTATIP2	7.837779042	17.66357696	1.172260719	2.67E-32	3.34E-31
AC110995.1	0.194480309	0.650764654	1.742511803	3.35E-21	1.35E-20
RNU6-97P	0.048644199	0.515727471	3.406269233	3.57E-07	5.75E-07
C19orf67	0.047955529	0.701440282	3.870551207	9.89E-28	6.81E-27
OR7E14P	5.362358708	2.164085498	-1.309110232	3.73E-31	3.86E-30
CHTF18	0.475129856	1.394323007	1.553171044	1.48E-27	1.01E-26
RARRES2	42.75623274	127.4521886	1.5757495	9.09E-21	3.54E-20
LINC00160	0.012243723	0.238290277	4.282605866	6.18E-19	2.11E-18
NR3C2	11.14803619	2.762160559	-2.012920503	1.39E-37	6.95E-36
LINC01767	0.04520809	0.235749603	2.382602472	3.41E-19	1.18E-18
AC131009.4	0.054949045	0.278329513	2.340627593	4.10E-13	9.56E-13
AC018665.1	0.661448371	1.69598847	1.358425905	3.62E-10	7.00E-10
ARL10	0.876762	2.24257344	1.354898055	2.92E-21	1.18E-20
SACS	0.774690667	1.952263719	1.333455687	2.64E-26	1.61E-25
AP006621.4	0.235820988	1.001369746	2.086210744	3.25E-12	7.16E-12
KRT14	0.100330903	0.316131993	1.655761009	0.00016495	0.000223597
RPS20P31	0.011448385	0.287009666	4.647883342	3.09E-09	5.67E-09
TMEM45A	0.973257596	8.021134611	3.04291272	3.83E-29	3.08E-28
IGKV3D-15	0.737091759	2.109140634	1.516739162	0.002103146	0.002639253
ACSM5	5.294032918	10.82986957	1.032576797	4.07E-11	8.35E-11
AL161668.4	1.775058229	0.103734943	-4.096892498	4.24E-39	3.70E-37
FAAHP1	0.164335821	0.69019275	2.070352338	1.75E-13	4.17E-13
CYP2B6	4.553810907	0.187378102	-4.603050117	1.70E-36	5.62E-35
PPP1R1B	1.593889782	0.111119008	-3.842374342	5.75E-37	2.30E-35
AC002525.1	0.496464388	0.247500021	-1.004261586	2.74E-07	4.45E-07
RDH12	4.006883572	0.99469439	-2.010155345	4.97E-06	7.45E-06
TFF1	1.679151313	0.69772493	-1.267001953	4.59E-06	6.89E-06
ZNF425	1.577574838	0.679426901	-1.2153182	1.37E-35	3.47E-34
AC022509.3	0.602419953	1.8686019	1.63311778	6.37E-24	3.17E-23
DUXAP8	0.036679161	0.250957731	2.774411845	1.60E-27	1.08E-26
GOLGA8N	0.15157701	0.342623556	1.176573391	1.35E-11	2.86E-11
KLHL21	16.29519586	7.120619411	-1.194372043	1.35E-20	5.20E-20
SLC25A25	12.28048744	3.37535946	-1.863254771	6.69E-35	1.43E-33
KIF26B	1.40845845	0.572350065	-1.29914729	4.96E-21	1.97E-20
CLDN7	34.69009389	14.75621772	-1.233200764	1.12E-23	5.45E-23

Table S1. The differentially expressed genes(DEGs) were screened out in TCGA

ID	conMean	treatMean	logFC	pValue	FDR
IDH2-DT	1.404985816	0.201796626	-2.799581607	1.61E-38	1.12E-36
AC007000.2	0.063539968	0.219667511	1.78958525	1.80E-07	2.96E-07
AL390729.1	0.030276578	0.318187126	3.393601381	3.25E-31	3.41E-30
AL807757.2	0.06858333	0.240921276	1.812631957	1.06E-08	1.88E-08
AC019069.1	0.206618079	2.132048327	3.367201737	4.95E-37	2.02E-35
AC093495.1	0.209244655	0.450530733	1.106434751	1.27E-12	2.86E-12
AC037487.3	0.211473271	0.774873387	1.87348527	2.14E-10	4.20E-10
AC080162.1	0.252461864	0.607492128	1.266800238	1.01E-11	2.15E-11
AC119424.1	1.130563176	0.165090533	-2.775712311	6.22E-27	3.99E-26
GPRC5B	24.06027486	10.38286162	-1.212449004	6.82E-32	7.97E-31
AC245884.3	0.576801096	2.221839945	1.945609082	2.85E-16	8.10E-16
SHROOM1	1.991107331	4.351390366	1.127905456	4.94E-24	2.48E-23
RPL12P47	0.071924713	0.249425926	1.79405197	4.52E-05	6.35E-05
IRF2BPL	23.29480036	8.39093805	-1.473103958	1.81E-33	2.81E-32
VAV3	27.17348351	10.44541001	-1.379330401	3.61E-35	8.17E-34
TEK	22.50718069	8.632241494	-1.382578221	3.85E-32	4.66E-31
AL592166.1	0.146076379	0.362584805	1.311595557	4.00E-23	1.86E-22
AL162377.1	1.471023506	0.601049765	-1.291263949	6.03E-34	1.03E-32
SLC29A4	1.873210439	8.375827693	2.160718781	1.49E-27	1.01E-26
LYL1	1.1346881	2.40287913	1.082468292	2.88E-21	1.17E-20
RF01241	0.181164626	0.395194706	1.125262336	1.20E-07	2.00E-07
MIR3942	0.182116018	0.504456138	1.469871015	1.87E-07	3.06E-07
ENTPD3	1.0429266	0.111807125	-3.221553591	9.12E-38	4.90E-36
GPR15	0.085963491	0.39091357	2.18505369	4.88E-10	9.37E-10
AL672207.1	0.969931827	0.310124963	-1.645033692	1.44E-13	3.44E-13
KRT8P33	0.213987491	0.470296191	1.136043182	1.97E-21	8.09E-21
DCDC2	23.81228676	7.71032063	-1.626843413	5.36E-30	4.78E-29
ACLY	33.71824625	88.47434425	1.391729663	1.39E-30	1.33E-29
AP000569.1	0.777144846	1.946962699	1.324969823	5.02E-06	7.53E-06
RN7SKP16	0.403985548	1.262591649	1.644012525	1.05E-14	2.71E-14
MIR4420	0.037886468	0.526033294	3.795399559	9.99E-15	2.57E-14
PPP4R1L	0.604765596	1.289049062	1.0918592	1.82E-19	6.47E-19
SYCE1L	0.597844489	1.72240252	1.52658017	5.92E-10	1.13E-09
AHRR	0.206329636	0.572329816	1.471895713	2.48E-10	4.86E-10
PLEKHG4B	0.858621018	0.191054602	-2.168036481	2.72E-13	6.41E-13
C1orf210	17.95728328	6.83541158	-1.39346899	7.66E-31	7.62E-30
PLCL1	16.17935939	2.606743224	-2.633834103	7.59E-38	4.20E-36
MSTO2P	0.552142124	1.55389077	1.492773518	7.23E-18	2.29E-17
AL606970.1	1.333563238	0.614441763	-1.117938056	1.73E-05	2.50E-05
ENAM	4.079121432	1.221067202	-1.740115854	5.21E-33	7.46E-32
CDH2	8.922311486	21.32867839	1.257305154	2.78E-23	1.31E-22
HSPA2	52.34832846	3.354695578	-3.963889711	2.22E-40	3.84E-38
AP3S1	8.510536542	17.38741598	1.03072155	3.89E-33	5.71E-32
RNU6-1160P	0.047059413	0.469974637	3.320027688	5.91E-14	1.45E-13
IL18BP	2.916749444	8.354901034	1.518261279	1.23E-30	1.19E-29
AC103810.1	0.090473723	0.281575877	1.637953007	6.52E-11	1.32E-10
AP002812.5	0.068616694	0.360178985	2.39208249	1.68E-22	7.48E-22
TRIM22	6.872980139	18.10588291	1.397450834	3.65E-27	2.39E-26
NCS1	10.76023706	4.607247909	-1.223732727	1.26E-30	1.22E-29
LHX2	0.008705954	0.252054896	4.855591839	4.41E-21	1.76E-20
CST7	2.115860032	18.83098504	3.15379237	2.68E-34	4.90E-33
CLDN9	0.774206285	0.279125312	-1.471805061	2.58E-11	5.37E-11
MIR8058	0.019963806	0.386407229	4.274663422	2.12E-12	4.73E-12
SCG5	0.694136437	2.055365449	1.566103764	1.16E-06	1.81E-06
ACBD3-AS1	0.139406209	0.420900858	1.594185632	5.49E-15	1.44E-14

Table S1. The differentially expressed genes(DEGs) were screened out in TCGA

ID	conMean	treatMean	logFC	pValue	FDR
AC092171.4	0.198323965	0.710168814	1.84030299	3.82E-11	7.86E-11
ADM	12.07183515	80.42273438	2.735958378	4.95E-37	2.02E-35
AL365356.5	0.079285061	0.308751319	1.961324344	2.32E-16	6.65E-16
NAP1L3	2.718359639	1.118912351	-1.280639309	5.23E-30	4.67E-29
ARRDC2	4.970225778	20.02854001	2.010673965	1.89E-34	3.59E-33
ABLIM3	4.395845149	18.01005537	2.034590146	2.13E-29	1.76E-28
TGFA	10.33891817	32.95195043	1.672278627	4.01E-25	2.21E-24
AC005264.1	0.014622459	0.237162394	4.019617426	1.96E-35	4.74E-34
SNORD101	0.597122204	1.42197577	1.25179876	1.06E-06	1.66E-06
CX3CR1	2.027509138	6.70631854	1.725812594	1.66E-17	5.13E-17
RAB29	26.46742503	12.50887588	-1.081265698	2.00E-22	8.80E-22
C1QC	25.38847476	173.9455879	2.776390454	1.61E-34	3.11E-33
RASD1	123.4203774	20.70849255	-2.575286168	7.31E-37	2.80E-35
BTG2	193.3563436	44.83398722	-2.108597384	3.05E-30	2.81E-29
MIR3685	1.690418329	4.455604915	1.398240997	3.90E-12	8.54E-12
PRKY	0.297945313	0.813703267	1.449455229	0.007704262	0.009271414
CACNA1C-A!	0.112867605	0.314416194	1.478044056	7.51E-19	2.55E-18
PXDC1	11.83476299	26.83896298	1.181298115	1.61E-28	1.21E-27
ANXA2R	0.471497819	1.925064552	2.029583819	8.55E-33	1.17E-31
PGGHG	2.031994943	24.24549482	3.576747981	3.89E-29	3.13E-28
AC012409.1	2.697267542	1.278088186	-1.077511246	1.20E-18	4.01E-18
AP001267.3	0.858788686	0.394296715	-1.123021495	1.07E-28	8.16E-28
LINC01606	2.060511101	0.214272976	-3.265480419	7.24E-42	2.76E-39
GPC5	4.265916853	0.074618444	-5.837179765	5.28E-43	2.94E-40
IFNGR2	18.42842153	39.67874578	1.10643392	8.05E-35	1.67E-33
AC104984.4	3.682573955	0.462925451	-2.99186271	3.54E-38	2.23E-36
CSF2RB	1.354506286	3.549222509	1.389735934	3.25E-23	1.53E-22
NLRP2	1.264827701	0.395416881	-1.677494501	7.00E-21	2.75E-20
AC010245.2	0.416246463	0.93672544	1.170188232	3.39E-23	1.59E-22
AC005776.2	0.069657548	0.330542771	2.246485364	2.49E-20	9.36E-20
FABP6	0.390912925	19.45977802	5.637504156	1.50E-38	1.05E-36
BMP7	3.334839914	0.378357432	-3.139795818	1.94E-37	9.23E-36
LAIR2	0.048505092	0.790285676	4.026166139	6.80E-29	5.34E-28
SLC13A4	0.093340113	0.239749801	1.360960507	1.80E-09	3.36E-09
AC104966.1	0.067775142	0.703967032	3.376679731	6.35E-24	3.16E-23
AC087301.1	0.131414892	0.425057155	1.693528074	5.88E-09	1.06E-08
AC015911.1	0.074056732	0.228242682	1.623865806	0.000131577	0.000179659
IL15RA	2.055387417	5.678963155	1.466217201	4.48E-30	4.03E-29
C17orf53	0.259770076	0.757906064	1.544783801	1.04E-32	1.41E-31
MRGPRF	5.654163	1.223246113	-2.208598776	7.92E-33	1.09E-31
AC093001.1	0.011143787	0.965969566	6.437666232	2.11E-10	4.15E-10
AC113383.1	0.342325802	0.799473533	1.223680236	1.53E-06	2.36E-06
AC015911.8	0.118244872	0.948182238	3.003386754	2.69E-28	1.98E-27
SNHG17	1.621331265	3.353464144	1.048473289	5.03E-21	2.00E-20
RNU6-1278P	0.041532471	0.254458618	2.615119447	1.16E-08	2.06E-08
REEP4	3.077310931	6.712879508	1.125261527	3.90E-30	3.55E-29
HASPIN	0.085646454	0.28481299	1.733549527	1.96E-28	1.46E-27
COL26A1	4.210523056	0.156993271	-4.745224834	2.74E-39	2.70E-37
STAT4	0.389246365	1.453216963	1.900494638	1.72E-32	2.24E-31
STX16-NPEP	0.07910886	0.336039759	2.086720745	8.26E-17	2.43E-16
TMIGD3	0.498164241	3.882011731	2.962111105	1.11E-36	3.97E-35
AC048341.2	0.78654094	2.522626732	1.681332983	1.06E-11	2.26E-11
AL390728.6	2.31611475	6.033090978	1.381190606	3.64E-22	1.58E-21
MCCC1	16.67542453	8.025507583	-1.055058945	4.19E-31	4.31E-30
LINC02499	1.062363146	0.093727066	-3.50266747	1.53E-13	3.66E-13

Table S1. The differentially expressed genes(DEGs) were screened out in TCGA

ID	conMean	treatMean	logFC	pValue	FDR
SRL	1.448605024	0.393006492	-1.882039235	8.67E-30	7.50E-29
SLFN12L	0.085869018	0.590092278	2.780730987	8.39E-31	8.30E-30
NCF1C	0.360334308	1.750387695	2.280266577	9.19E-31	9.04E-30
APOL2	11.78700781	30.35294725	1.364639079	4.11E-32	4.97E-31
TSPAN4	7.397576917	17.02767571	1.202756822	6.56E-33	9.19E-32
SEMA6A	2.831858922	10.95869285	1.952254424	2.56E-29	2.11E-28
CLDN19	32.00252953	0.627481467	-5.672469283	2.32E-41	6.92E-39
IGHV3-49	3.019982567	17.28818116	2.517173969	6.01E-06	8.96E-06
GNG7	8.1336065	3.088619511	-1.39693304	1.68E-32	2.20E-31
AC108727.1	0.202179873	0.470956516	1.219954477	5.45E-07	8.69E-07
U73169.1	0.058882449	0.285203073	2.276079934	4.15E-15	1.09E-14
ZNF26	0.403563214	0.814042617	1.012309652	1.00E-22	4.54E-22
CRYBB2	0.091891097	0.315006788	1.77738592	8.84E-19	2.98E-18
RNU6-1048P	0.287291072	0.902975745	1.652174075	3.70E-12	8.12E-12
LCN1	0.004005765	0.39145799	6.610636044	9.49E-23	4.30E-22
MIR4697HG	0.330250903	0.700477594	1.084776398	8.21E-05	0.000113411
LINC02521	0.030766358	0.227647061	2.887373252	2.97E-29	2.43E-28
TMPRSS13	0.579745579	0.172266754	-1.750775613	0.000337109	0.000447306
KLRB1	1.471052933	4.314373181	1.552301811	7.10E-23	3.25E-22
CAPN8	0.652763759	0.231308873	-1.496740354	2.46E-17	7.51E-17
IGLV4-60	0.986003858	7.428199202	2.913347308	0.000295687	0.000394199
GAPDHP72	0.112608161	0.267646092	1.249015198	8.93E-17	2.63E-16
PPIAP45	0.039650163	0.468260028	3.561911182	1.17E-13	2.81E-13
AC020779.2	1.299145707	0.313116204	-2.05279317	5.32E-27	3.43E-26
AL031123.3	0.686247687	0.160048114	-2.100223702	2.40E-33	3.64E-32
NGF	1.724507074	7.614480068	2.142561483	7.05E-25	3.80E-24
LINC00926	0.143547305	0.576711203	2.006322805	2.77E-22	1.21E-21
DPY19L1P1	0.587988553	1.280747853	1.123126499	3.18E-18	1.03E-17
CYP3A5	1.208858242	5.412248331	2.162582961	1.17E-21	4.87E-21
SCNN1D	0.205090881	1.118036754	2.446632362	1.79E-20	6.84E-20
LGALS3	40.40707125	98.89604017	1.291304969	1.07E-16	3.13E-16
RPS12P26	0.250946852	0.57096358	1.186016877	2.22E-11	4.64E-11
RN7SKP150	0.130307209	0.532791252	2.031653492	5.65E-15	1.48E-14
LINC01714	0.190397275	0.573805656	1.591549355	1.37E-11	2.90E-11
AC007906.2	25.22155967	1.575783928	-4.000515862	3.09E-39	2.92E-37
FGG	4.807253771	32.03921351	2.736553864	2.60E-05	3.72E-05
CAV1	14.6949154	67.82712973	2.206545389	5.37E-35	1.17E-33
CBLN1	0.100580264	0.436741893	2.118433682	0.000583109	0.000761452
SLC22A12	60.82427463	15.81901693	-1.942987264	5.00E-10	9.59E-10
ELFN1	3.560235581	1.768097686	-1.009774723	4.29E-14	1.06E-13
AL162586.1	0.582168761	1.360771309	1.224915296	1.08E-09	2.03E-09
RASGRP3	2.125646417	4.549844282	1.097915533	1.03E-25	5.97E-25
KLHL6-AS1	0.429875629	1.067467862	1.312201408	1.53E-06	2.37E-06
SLC19A2	9.186015076	4.131322657	-1.152835412	1.70E-29	1.43E-28
AC092681.1	0.421549465	0.888384906	1.075482952	1.02E-10	2.04E-10
AL078587.1	0.073165646	0.277954854	1.925612263	6.17E-10	1.18E-09
AL162171.1	2.443813018	0.76742304	-1.671039922	9.84E-29	7.57E-28
AC097504.2	0.646768563	0.254349588	-1.3464368	5.17E-27	3.35E-26
LINC00472	4.818639603	1.329253511	-1.858009626	2.14E-35	5.13E-34
AC105105.3	0.027002818	0.318164884	3.558592716	1.40E-24	7.40E-24
AFMID	15.65068176	7.680142191	-1.027020578	9.13E-26	5.32E-25
GAS6-DT	2.265056582	0.900256654	-1.331138827	8.73E-25	4.67E-24
SOSTDC1	15.92105641	2.23066456	-2.835390579	9.17E-37	3.43E-35
RNU6-610P	0.184218368	0.705299338	1.936818768	1.10E-10	2.20E-10
FGFBP2	0.560145814	2.112985156	1.915408297	1.00E-22	4.54E-22

Table S1. The differentially expressed genes(DEGs) were screened out in TCGA

ID	conMean	treatMean	logFC	pValue	FDR
AC138207.4	0.65609839	1.428084035	1.12209679	3.91E-17	1.18E-16
XK	0.965144899	0.257089546	-1.908474607	6.38E-32	7.48E-31
GGT8P	0.301965661	1.791896263	2.569030716	1.93E-20	7.34E-20
AC092301.1	0.06719418	0.241983787	1.848502211	3.75E-15	9.93E-15
AC009041.2	0.184570915	0.634588221	1.781645514	0.002127931	0.002669379
MS4A4E	0.081544727	0.773823091	3.24634029	4.90E-31	4.99E-30
TM6SF1	1.025916514	2.093374738	1.02891726	2.85E-13	6.71E-13
AC069528.2	0.442256324	0.980710235	1.148944163	4.30E-16	1.21E-15
AHSA2P	1.340136279	5.993290927	2.160968691	1.88E-25	1.06E-24
PP7080	48.5093381	8.981723355	-2.433198303	7.00E-32	8.15E-31
MSR1	1.976159253	8.485425345	2.102287765	9.49E-28	6.54E-27
AC008740.1	0.056313335	0.302825295	2.426937224	5.84E-13	1.35E-12
LINC01543	6.51959216	0.063067521	-6.691740678	3.21E-39	3.02E-37
TRBV6-5	0.180384492	1.651042643	3.194230164	1.19E-23	5.80E-23
SNORA11	0.460773097	1.487719417	1.690974071	3.53E-06	5.35E-06
AL020997.2	0.09257984	0.327903317	1.824500519	0.002620063	0.003263863
CTXND1	1.876877788	0.446013579	-2.073175174	1.91E-30	1.81E-29
CLDN11	2.237101722	0.395328977	-2.500505246	5.48E-32	6.49E-31
AC091185.1	0.171909205	0.846735722	2.30026496	7.20E-24	3.55E-23
DIRC3	0.148503618	0.480643752	1.694469902	5.26E-17	1.57E-16
EGFL7	12.60895651	28.23157113	1.162860533	1.43E-13	3.42E-13
FAM229A	0.437422	0.98307228	1.168271711	1.12E-07	1.87E-07
FGD5-AS1	45.0959475	20.72309911	-1.121758021	2.81E-37	1.26E-35
SMIM10	4.311140625	8.940358961	1.052263135	3.78E-21	1.52E-20
SIGLEC22P	0.064944098	0.249275523	1.940470895	2.13E-19	7.51E-19
LINC00482	0.270330208	0.674345988	1.318766255	0.000133891	0.000182722
CA10	3.692737373	0.116661506	-4.984290152	8.91E-42	3.16E-39
TTYH3	11.75810811	46.1347723	1.972198588	2.26E-32	2.87E-31
CALM2P2	0.513299054	1.435009617	1.4831889	3.87E-16	1.09E-15
TMEM30B	16.97602308	2.146878929	-2.983185783	2.64E-36	8.13E-35
GOLGA8B	0.635754207	2.620307422	2.043195074	5.06E-13	1.17E-12
GOT2	55.53744931	25.76515548	-1.108039621	5.42E-35	1.18E-33
AGMAT	68.35658834	13.51153143	-2.338889192	5.88E-08	9.95E-08
AP000786.1	0.150443752	0.378929197	1.332704109	2.95E-07	4.77E-07
AC009087.1	0.079819463	0.221357744	1.47156737	7.38E-09	1.32E-08
SAMD3	0.082423135	0.433972173	2.396481297	8.06E-33	1.11E-31
CHFR	0.655053721	1.440495051	1.136879572	5.46E-35	1.19E-33
TMEM213	69.45706511	7.87924815	-3.139991574	1.31E-36	4.58E-35
RHEBP2	0.556261535	0.245103682	-1.18237119	1.85E-12	4.14E-12
ADGRL4	15.22727414	30.97867482	1.024617725	2.04E-14	5.15E-14
ANO5	2.884860404	0.697612012	-2.048004723	9.30E-34	1.54E-32
AL080317.2	0.18743386	0.589897072	1.65408165	4.83E-15	1.27E-14
MYO10	10.53886717	4.8397286	-1.122721746	2.44E-29	2.01E-28
AC009336.1	0.459099329	0.211975598	-1.11490813	1.86E-17	5.71E-17
NRP1	19.33172615	41.3433067	1.096683319	1.34E-21	5.57E-21
OXCT1	31.02574981	12.88907579	-1.267317258	4.91E-33	7.05E-32
PROS1	16.68525179	37.34381309	1.162295787	2.36E-19	8.32E-19
AL445991.1	0.094638441	0.216689734	1.195132583	2.19E-06	3.36E-06
CLECL1	0.115265646	0.580856705	2.333219711	7.76E-27	4.94E-26
AC011411.1	0.037638989	0.337060441	3.16270752	1.12E-27	7.67E-27
AC106795.2	0.507669308	0.180153648	-1.494661178	1.29E-11	2.73E-11
AC069257.2	0.18276953	0.416919546	1.189743435	4.51E-05	6.35E-05
SLC5A2	8.089921186	0.329811965	-4.616410003	1.19E-19	4.29E-19
POLR2J3	0.177234375	0.356859782	1.009698876	2.73E-15	7.31E-15
GPX1P1	0.428772665	0.884323793	1.044361772	3.65E-06	5.51E-06

Table S1. The differentially expressed genes(DEGs) were screened out in TCGA

ID	conMean	treatMean	logFC	pValue	FDR
RPL37	37.75943	75.98261772	1.008832429	1.36E-31	1.50E-30
SFRP1	99.57998058	2.681934537	-5.214509806	1.10E-40	2.35E-38
CFLAR-AS1	0.078687864	0.562187093	2.83683728	7.19E-27	4.59E-26
AC073174.1	2.555823851	0.995639231	-1.360093425	1.32E-07	2.18E-07
SNORA31	0.548961778	2.763544343	2.331742154	8.60E-16	2.37E-15
COL4A6	2.555318328	0.127328038	-4.326880983	1.11E-40	2.35E-38
AC007114.1	4.509993963	1.88359235	-1.259638734	6.96E-30	6.12E-29
UGT2A1	1.680503923	0.072063182	-4.543487739	2.89E-27	1.92E-26
KIF18B	0.106537277	0.703703389	2.723609142	8.05E-35	1.67E-33
RNU6-1011P	0.126873626	0.511155543	2.010370162	3.24E-15	8.62E-15
GABARAPL1	84.36010986	28.69902076	-1.555559462	3.00E-37	1.33E-35
AC007038.1	0.221807149	0.624189924	1.492679206	6.21E-14	1.52E-13
MSS51	0.217666512	0.622219774	1.515304779	2.93E-15	7.81E-15
MYOSLID	0.086680165	0.292671303	1.755507491	2.04E-15	5.50E-15
PCK1	149.2913056	22.33713424	-2.740614137	3.76E-19	1.30E-18
CYTL1	0.620975596	1.974365518	1.668780626	1.38E-15	3.77E-15
RIT1	9.737691042	21.42323593	1.13752478	6.34E-29	5.00E-28
RNA5SP268	0.068886808	0.26795282	1.959679361	0.00027885	0.000372331
CDK3	0.06523874	0.357515974	2.454206888	1.70E-22	7.53E-22
AL049840.4	3.189331681	6.913197302	1.116098959	3.82E-22	1.65E-21
ZNF676	0.781153727	0.340394534	-1.198398623	8.99E-25	4.81E-24
AFF3	0.633669914	1.781880415	1.491597094	5.42E-17	1.62E-16
RHOXF1	0.137017054	0.447243591	1.706705335	1.04E-10	2.09E-10
TEDC2	0.215685665	0.432510506	1.003804887	1.57E-11	3.32E-11
DACT2	1.428570785	0.620313304	-1.20350355	2.17E-20	8.21E-20
MIR3682	1.249310669	2.604344735	1.059788149	1.29E-06	2.00E-06
AP002884.1	0.432109079	2.446632403	2.501329909	2.13E-33	3.26E-32
GNLY	0.490162003	4.338098701	3.145732321	4.28E-37	1.79E-35
LINC01234	0.038619992	0.504905611	3.708593934	6.98E-17	2.07E-16
AL355338.1	5.936277153	2.443675706	-1.280505609	2.04E-32	2.61E-31
INO80B	1.118413067	2.379996801	1.089506513	1.73E-24	9.02E-24
TPSD1	0.250199256	0.643603476	1.363094618	8.54E-09	1.53E-08
AL928654.4	0.216114068	0.485244939	1.16692018	3.78E-24	1.91E-23
EZH2	0.504125022	1.857130958	1.881222083	4.69E-36	1.34E-34
SPATA17	0.782042833	0.362412883	-1.109613384	1.10E-25	6.38E-25
AC126773.1	0.049273107	0.237396165	2.268424285	2.93E-08	5.05E-08
CES4A	0.604520013	7.942975039	3.715817464	1.76E-34	3.36E-33
AC011933.1	0.069031232	0.27919662	2.015960346	4.27E-19	1.47E-18
LINC02100	0.063519158	0.873416948	3.781406821	1.55E-23	7.47E-23
WFDC10B	0.018047441	0.232847369	3.689518393	2.86E-12	6.33E-12
CORO1C	12.86157771	27.06598174	1.073413092	1.04E-29	9.00E-29
CATSPER3	0.374226772	0.779965712	1.059497931	4.32E-16	1.22E-15
IL22RA1	0.865452788	2.586410077	1.579424009	1.95E-15	5.26E-15
AMH	0.060116315	0.39390047	2.712002652	5.55E-17	1.65E-16
COL11A2	0.12661353	0.289906598	1.19515659	5.32E-09	9.61E-09
MMP11	0.922479864	3.658903457	1.987822025	1.11E-29	9.58E-29
LRMP	1.988999953	4.940085501	1.31249277	1.14E-17	3.55E-17
AC012170.2	0.338887017	0.771023781	1.185970992	5.77E-09	1.04E-08
SLC7A9	16.0912293	6.584554066	-1.289116903	0.015371292	0.018047571
IL4I1	1.567318124	6.66819712	2.088998715	2.42E-27	1.61E-26
AP001033.1	0.99297916	0.249925188	-1.990267134	1.63E-35	4.04E-34
BUB1	0.248782564	1.069230272	2.103615307	3.99E-33	5.84E-32
AC009951.1	0.010020769	0.232969802	4.53907783	5.67E-30	5.04E-29
RGL4	0.096268601	0.375564126	1.963922034	7.93E-30	6.89E-29
TAP1	9.824433889	37.89533981	1.947574261	5.31E-39	4.45E-37

Table S1. The differentially expressed genes(DEGs) were screened out in TCGA

ID	conMean	treatMean	logFC	pValue	FDR
IGKV1D-8	0.557545693	1.837839128	1.720848539	5.76E-06	8.60E-06
IL32	21.53573967	83.82719765	1.960685526	2.32E-28	1.71E-27
DANCR	19.82004889	5.901720696	-1.74775297	6.43E-38	3.64E-36
GPX2	1.981453477	0.833502518	-1.249300632	4.76E-28	3.39E-27
LNX1	7.000729417	2.436167202	-1.522892093	3.59E-32	4.38E-31
AC093162.2	0.473383946	1.429102835	1.594027046	1.83E-18	6.02E-18
CA1	0.064220997	0.365426958	2.508466096	1.77E-08	3.10E-08
AC099535.1	0.107050883	0.3676388	1.779992343	2.25E-10	4.41E-10
DLEU2	0.300367226	0.752263636	1.324510946	8.57E-28	5.94E-27
AP003392.2	0.848771507	3.005051687	1.823941675	1.78E-15	4.82E-15
CABYR	0.166246082	0.468725657	1.495423424	1.06E-23	5.16E-23
AL158834.2	0.063918798	0.280866533	2.135572532	4.32E-22	1.86E-21
PFKP	28.33611743	110.7615584	1.966743257	2.81E-33	4.20E-32
AC109583.2	1.348084036	0.621200352	-1.11777988	4.19E-19	1.45E-18
AC012065.3	0.690362021	1.770718193	1.358909623	3.91E-09	7.12E-09
ATP5PBP5	2.554570235	1.001515944	-1.350895212	9.89E-33	1.35E-31
RGS20	0.049943709	0.262410345	2.393449724	8.21E-19	2.77E-18
IL24	0.398831635	0.874172873	1.132138761	6.65E-19	2.26E-18
LPAR6	5.298160875	12.60508219	1.250441969	6.71E-23	3.08E-22
MALAT1	15.83500685	71.62380438	2.17732166	3.77E-10	7.29E-10
AQP3	181.3012126	38.36542064	-2.240510097	1.11E-34	2.23E-33
INHBB	1.785995579	25.1763071	3.817266267	5.40E-38	3.17E-36
RNU6-1099P	0.241185069	0.782082548	1.697180295	8.89E-11	1.79E-10
APOLD1	9.551160456	38.6446188	2.016519601	3.21E-20	1.20E-19
CD3D	1.618134958	12.11799203	2.904746818	4.82E-30	4.33E-29
GMPR	18.9570153	3.60522437	-2.394570883	3.39E-35	7.72E-34
CD160	0.097055096	0.368600621	1.925182628	2.67E-32	3.34E-31
SPI1	3.241952369	16.63906764	2.359639795	1.76E-35	4.33E-34
WNT11	1.353755155	0.465916315	-1.538824076	1.14E-18	3.82E-18
AL031846.2	0.165472565	0.609876724	1.88192562	2.26E-19	7.96E-19
SCD5	30.30366839	9.379611275	-1.69189241	2.98E-31	3.13E-30
AF186192.2	1.21758524	0.421162667	-1.531573313	2.55E-30	2.37E-29
ZNF248	1.897117433	3.803699527	1.0035943	4.71E-23	2.18E-22
TMPRSS11CI	0.06110075	0.259198268	2.084794075	5.12E-13	1.19E-12
FAM19A5	0.619465019	1.696229581	1.453236728	7.27E-11	1.47E-10
P4HA2	5.700932	14.17300312	1.313875785	6.12E-25	3.32E-24
CLEC18B	2.434091234	8.341671672	1.776953285	3.02E-11	6.25E-11
AC004930.1	0.025189392	0.316404311	3.650881045	6.34E-13	1.46E-12
FTH1P4	0.166639783	0.406865732	1.287819915	4.87E-12	1.06E-11
AL121761.1	0.388079228	0.179796497	-1.109986297	4.47E-19	1.54E-18
DNASE2B	0.037669388	0.283850006	2.913664269	4.36E-20	1.62E-19
PAPPA	4.588428132	0.526282964	-3.12408941	1.67E-38	1.16E-36
AC026333.4	0.077933936	0.257530006	1.724416956	7.47E-15	1.94E-14
CHRDL1	7.544807333	2.171027519	-1.797106047	9.32E-29	7.20E-28
IGFBP3	50.72550458	562.2928074	3.470538377	2.16E-35	5.17E-34
AC097658.1	0.16121303	0.480004161	1.574078555	1.94E-20	7.40E-20
CD38	0.36920732	1.385397938	1.907797369	7.46E-18	2.35E-17
KLHL6	0.219051042	1.392989421	2.668845319	1.07E-35	2.79E-34
CD4	6.587828292	23.21248573	1.817026166	2.33E-30	2.17E-29
RPL32P1	0.102028774	0.723311807	2.825641621	1.46E-21	6.05E-21
AL355388.2	0.092308192	0.315543922	1.77331024	8.94E-14	2.16E-13
ASB9P1	0.098385201	0.384565615	1.966716551	4.85E-13	1.13E-12
PTPRO	11.84453077	1.362434221	-3.119962564	2.77E-08	4.79E-08
CNKSR1	2.830565743	0.468329354	-2.59549506	7.64E-35	1.60E-33
HACD3	33.5541705	12.20431418	-1.459100867	1.71E-36	5.65E-35

Table S1. The differentially expressed genes(DEGs) were screened out in TCGA

ID	conMean	treatMean	logFC	pValue	FDR
IL10RA	1.7733518	8.547340509	2.268996829	1.42E-31	1.56E-30
AC010536.2	0.082191889	0.518917916	2.658438417	1.15E-11	2.45E-11
AL590560.1	0.511989417	1.731293808	1.757664681	1.08E-10	2.16E-10
AC024145.1	0.14571822	0.535562285	1.877873091	1.25E-14	3.20E-14
IGKV1-9	6.249610452	34.40638203	2.460838023	3.77E-05	5.33E-05
SFRP2	6.892486494	26.20857179	1.926942297	0.018758414	0.021895806
RARRES3	34.38991556	80.1438001	1.220605345	1.57E-15	4.26E-15
LINC02019	0.149048483	0.35662173	1.258612918	4.05E-17	1.22E-16
ZNF503	9.434193916	2.475242842	-1.930329183	3.26E-37	1.42E-35
KCNJ14	0.164486031	0.415851364	1.338102899	2.46E-23	1.17E-22
FILIP1	0.974215339	2.606160067	1.419613092	5.37E-20	1.98E-19
AC023154.1	1.457783421	0.036428063	-5.322582286	2.12E-31	2.27E-30
ABI3	5.4650145	11.62106794	1.088445425	1.53E-23	7.41E-23
ANKRD10-IT	2.534112981	7.590593428	1.582731832	1.90E-20	7.24E-20
CRHR2	0.096275014	0.259108678	1.428323996	1.15E-07	1.91E-07
PIDD1	0.896995229	2.380530926	1.408111154	3.52E-23	1.65E-22
LOXL3	0.6874365	2.082144719	1.598771986	8.57E-28	5.94E-27
CPNE7	0.244287468	1.211761063	2.310453496	3.78E-11	7.78E-11
BCAM	151.2481679	46.54557323	-1.700201797	9.51E-37	3.52E-35
GZMB	0.760945961	4.539312802	2.576607999	3.66E-32	4.45E-31
WSCD2	0.783080446	0.160998506	-2.282113219	5.93E-37	2.35E-35
CD6	0.379998834	1.976623131	2.378970932	1.88E-31	2.04E-30
RNA5SP296	0.103245517	0.382537958	1.889523766	3.37E-07	5.44E-07
DHDH	9.627835494	4.437280615	-1.117535699	0.001773267	0.002237834
COBLL1	21.57975581	4.087285029	-2.40046378	4.53E-39	3.92E-37
AL353804.2	0.036084652	0.296991746	3.04096559	2.15E-16	6.17E-16
AC022400.7	1.110377342	2.818306235	1.343778349	8.96E-23	4.07E-22
ABCB1	22.41781958	6.986940544	-1.681913196	5.76E-35	1.24E-33
RPS6KL1	0.309419328	0.708671434	1.195553579	1.57E-11	3.30E-11
IGHV1OR15-:	0.195240142	0.90479879	2.212347293	0.000291806	0.000389175
AP005233.2	0.028870837	4.031178858	7.125445033	3.61E-32	4.40E-31
AF111169.3	0.075761938	0.455529806	2.588000311	4.35E-25	2.38E-24
AL645940.1	0.101613925	0.317535696	1.643820669	1.13E-11	2.39E-11
PKIB	0.732018478	1.492922548	1.02818735	4.24E-08	7.24E-08
PRR11	0.432956225	1.614095739	1.898433082	1.69E-30	1.61E-29
SULT2B1	3.078149876	0.324451164	-3.245990234	6.37E-13	1.47E-12
ABCA4	5.704451366	0.223627705	-4.672917303	4.49E-38	2.70E-36
AFAP1-AS1	0.028427279	0.416487594	3.872925585	6.57E-17	1.95E-16
AC104072.1	2.087981359	0.10882948	-4.26196752	5.45E-59	1.87E-55
SNORD12C	0.067410644	0.317382473	2.235174136	3.87E-10	7.47E-10
IGHJ3P	0.269995585	2.851605053	3.400766462	2.86E-06	4.35E-06
UBXN11	2.845217014	7.217950686	1.343050591	9.68E-24	4.74E-23
AC067817.2	0.250253489	0.993010851	1.988419302	3.68E-11	7.57E-11
AC022706.1	0.445055972	0.917695168	1.044028225	1.15E-14	2.95E-14
PHYKPL	2.974751264	9.525280338	1.678992512	1.08E-36	3.89E-35
AC073130.2	0.091287256	0.344391647	1.915564774	1.81E-23	8.71E-23
AC022784.1	0.253861588	0.844935468	1.734799047	2.61E-16	7.43E-16
PLEKHB1	6.76544531	1.277988454	-2.404310095	6.65E-36	1.84E-34
RUNX2	0.500835638	1.385548304	1.46804788	8.29E-22	3.50E-21
CHST9	0.848224842	1.996529543	1.234975779	0.000435715	0.000573856
FAM83B	3.061280484	0.266432183	-3.522294969	9.03E-39	6.89E-37
ADCY10P1	0.327390858	0.755088947	1.205632561	5.24E-07	8.37E-07
PRODH2	35.03860194	7.812471513	-2.165094283	0.000158108	0.000214549
GPM6B	3.772448464	0.875595194	-2.107165251	1.18E-34	2.35E-33
AC012181.1	0.453947644	1.343302473	1.565186374	5.35E-17	1.60E-16

Table S1. The differentially expressed genes(DEGs) were screened out in TCGA

ID	conMean	treatMean	logFC	pValue	FDR
TAZ	2.512156556	5.38509528	1.100045496	2.19E-28	1.63E-27
AC092542.1	0.172227987	0.657488916	1.932646974	1.28E-15	3.49E-15
LAYN	2.395805758	5.741435042	1.260900431	2.68E-20	1.01E-19
ENPP1	2.860093124	1.355302349	-1.077447389	5.50E-23	2.54E-22
BHLHB9	2.394038931	1.161768147	-1.043124432	1.09E-35	2.84E-34
C1orf195	0.079158108	0.405713685	2.357652933	0.000103589	0.000142284
P3H1	2.595312167	6.425397578	1.307877648	1.04E-33	1.70E-32
NBPF15	1.261630086	3.07368901	1.284682234	1.93E-28	1.44E-27
H1F0	182.7320093	48.2787236	-1.920269935	1.58E-19	5.64E-19
DGKD	1.214062043	5.383360917	2.148665001	4.24E-37	1.78E-35
AL353801.1	5.664125478	1.296056012	-2.127725157	3.69E-29	2.98E-28
TNN	0.181198397	0.380027381	1.068533175	0.002067913	0.002596462
AC132872.4	0.068951152	0.350134175	2.344261325	2.03E-23	9.72E-23
BTC	4.092454764	1.157579386	-1.821855334	2.26E-36	7.14E-35
MIR26B	0.122728698	0.395360472	1.687696004	2.25E-07	3.67E-07
TPSAB1	1.572556854	5.045338535	1.681838898	2.21E-20	8.37E-20
HIST3H2A	1.193807828	3.426505392	1.521167339	0.004143083	0.005087545
SGK2	13.74532426	4.641130054	-1.566392911	4.26E-32	5.13E-31
HSPD1P4	0.128003241	0.256017972	1.000064748	2.88E-10	5.61E-10
AC019226.1	0.137448829	0.424590731	1.627178263	2.39E-11	4.98E-11
AOAH	1.455888357	6.794139932	2.222391204	7.55E-30	6.58E-29
NINL	6.687759208	2.922379792	-1.194379222	3.66E-31	3.80E-30
STAG3L5P	0.671310943	2.993066756	2.156571391	9.82E-20	3.56E-19
AC090772.4	0.679775266	1.389749302	1.031694883	6.10E-09	1.10E-08
AP001001.1	0.122395941	0.325127129	1.409448231	3.53E-15	9.38E-15
AC122688.3	0.131794809	0.292732006	1.15128694	6.53E-09	1.17E-08
PODNL1	0.132711477	0.763149409	2.523672395	1.62E-08	2.84E-08
XIST	0.997847339	3.059420493	1.616367389	4.48E-08	7.64E-08
MSTN	0.111334463	0.308371399	1.469768729	5.41E-09	9.78E-09
EEF1DP5	0.176288459	0.382802403	1.118661852	7.64E-05	0.000105966
GK	9.87483969	3.711115972	-1.411904242	1.11E-08	1.97E-08
TRBV30	0.110564428	0.62400682	2.496674495	9.14E-16	2.52E-15
CNN2P9	0.123444055	0.260015253	1.0747389	3.12E-15	8.30E-15
FOXC2	2.489140742	5.962520783	1.260274584	1.56E-11	3.29E-11
SLC41A2	3.536377875	8.208456311	1.214838493	2.03E-31	2.19E-30
BEX2	28.04341903	13.75273846	-1.027943334	5.73E-25	3.11E-24
CENPH	0.820401667	2.090512855	1.349454587	1.97E-32	2.54E-31
AC016405.1	0.047758963	0.28203702	2.562041116	5.29E-11	1.08E-10
AL139349.1	0.288177374	1.652000911	2.519185511	6.87E-17	2.03E-16
MIAT	0.033592374	0.734769957	4.451086975	1.54E-34	2.99E-33
CCDC13	0.561429665	0.208314667	-1.430340873	7.55E-32	8.72E-31
CGNL1	51.16667194	11.82991647	-2.112764511	2.70E-38	1.76E-36
OR52N4	0.069167246	0.420757039	2.60482649	3.42E-20	1.28E-19
GRB7	11.75244738	5.090433985	-1.207100657	8.66E-29	6.72E-28
HSPE1P18	0.054941676	0.315441312	2.521398793	5.70E-14	1.40E-13
TNC	32.0911616	15.00162227	-1.09705749	7.32E-07	1.16E-06
CRTAM	0.140274249	1.197757133	3.094013307	2.14E-32	2.74E-31
ACCS	1.497528861	3.738437803	1.319851723	2.75E-20	1.04E-19
SCARNA9	1.137593669	4.280203761	1.911694137	5.07E-08	8.62E-08
HLA-V	0.259283128	1.137347471	2.133072846	6.05E-24	3.02E-23
AC068580.1	0.693650822	1.722327894	1.312078317	3.58E-13	8.37E-13
AC119396.2	0.191575567	0.474303943	1.307898282	4.11E-07	6.60E-07
NRK	5.482930404	0.191085479	-4.842657106	2.34E-40	3.95E-38
CALB2	0.097860397	0.377642218	1.94822301	6.93E-19	2.35E-18
SNORD46	0.221810124	1.18370783	2.415915911	6.21E-16	1.73E-15

Table S1. The differentially expressed genes(DEGs) were screened out in TCGA

ID	conMean	treatMean	logFC	pValue	FDR
CDH16	346.7096125	64.79555573	-2.419761067	3.44E-41	9.70E-39
SLC17A9	0.202426168	2.049075086	3.339505141	1.32E-34	2.60E-33
MUC1	62.08031639	20.48334161	-1.599684817	2.05E-31	2.20E-30
RF00569	0.310338035	0.722297459	1.218752573	1.18E-06	1.84E-06
SNORA71B	0.209083912	0.889761927	2.089337311	1.96E-17	6.03E-17
AP005131.7	0.25014345	0.524083239	1.067040294	1.64E-07	2.70E-07
AC110285.6	0.073989429	0.267158638	1.852305587	9.70E-07	1.52E-06
ITIH1	0.013430357	0.840533927	5.967736519	3.21E-12	7.07E-12
AL731533.2	0.979157867	4.530346454	2.210007998	8.21E-32	9.39E-31
SPACA9	5.895429194	2.648956562	-1.154172661	4.33E-32	5.19E-31
PANK1	7.949893347	2.990223588	-1.410682143	4.50E-28	3.23E-27
ZNF83	3.586138097	10.22595921	1.511733226	2.46E-29	2.03E-28
PRMT6	14.29197143	5.905813857	-1.274997145	1.43E-39	1.65E-37
ATP6V1H	21.75309986	9.566379163	-1.185176124	3.84E-37	1.64E-35
TUBA3E	0.265976945	1.287764595	2.275495789	2.54E-21	1.04E-20
AC025271.4	0.866756792	0.178520735	-2.279535586	1.05E-35	2.76E-34
IGLV2-5	0.083196887	0.284356473	1.773099187	0.002923973	0.003630149
MMP14	23.03693632	62.71335094	1.444823743	1.19E-28	9.08E-28
CH25H	2.768572289	1.085744478	-1.350457577	1.26E-09	2.37E-09
LUM	71.24277354	28.76824695	-1.308266373	4.92E-18	1.57E-17
CXCL10	3.230415582	35.63265339	3.463408238	1.08E-31	1.21E-30
HOXD3	1.527709192	0.717585459	-1.090147383	8.17E-23	3.72E-22
RFX8	0.05793485	0.649467406	3.48675378	6.13E-36	1.71E-34
CROCCP3	0.243826423	0.678686399	1.476890625	1.64E-15	4.45E-15
MAPRE2	10.41575403	21.20408949	1.02557525	1.78E-27	1.20E-26
CX3CL1	20.38310458	41.15719871	1.013770984	3.02E-18	9.80E-18
TRGC2	0.210222527	1.067557081	2.34432403	1.49E-25	8.51E-25
RNU6-762P	0.234840984	0.748848148	1.672988991	2.91E-11	6.03E-11
HOXA13	0.07170623	0.386841028	2.431570428	8.23E-12	1.77E-11
STRA6	2.406388614	0.153884146	-3.966953138	2.12E-23	1.01E-22
PLCXD3	2.719047508	0.783007491	-1.796003344	1.04E-27	7.17E-27
GTF2IP14	0.23996966	0.497630581	1.052223131	2.99E-08	5.16E-08
TAP2	3.191248139	7.1862553	1.171119398	4.42E-29	3.55E-28
CD40	6.298331111	22.51214435	1.837661976	6.24E-36	1.74E-34
AGAP13P	0.131014963	0.571561446	2.125177015	6.01E-14	1.47E-13
MYBL1	0.341752438	1.169402929	1.774748578	6.01E-32	7.08E-31
FMR1-IT1	0.277187352	0.778214173	1.489305823	1.35E-11	2.85E-11
AC093827.3	0.255533635	0.730213015	1.514804185	7.48E-21	2.93E-20
AC020658.5	0.136235651	0.421622932	1.629849046	8.67E-17	2.55E-16
CFB	2.074003074	5.07234635	1.290235227	5.15E-16	1.44E-15
RNU6-1098P	0.206832596	0.83792283	2.018353817	1.06E-12	2.42E-12
ZBTB32	0.074897747	0.271962501	1.860413514	5.70E-23	2.63E-22
ENKUR	0.193718139	0.558118736	1.526613034	2.44E-15	6.54E-15
DARS-AS1	0.113055018	1.029901889	3.18740998	1.51E-37	7.48E-36
AL513164.1	0.013057602	0.430836864	5.044179809	3.15E-12	6.93E-12
AC104237.2	3.063654486	0.149280169	-4.359159176	3.58E-48	3.35E-45
PLVAP	92.60878361	358.9141602	1.954417903	1.04E-25	6.01E-25
MASP1	0.967176061	4.030233341	2.059012928	4.09E-19	1.41E-18
MATN2	14.61173775	6.742851655	-1.115697002	1.14E-25	6.56E-25
TRAF3IP2	3.322183208	6.941959833	1.063211382	6.09E-29	4.81E-28
SYT7	6.772723254	0.571535449	-3.566821157	6.20E-38	3.55E-36
MIR6753	0.047639953	0.394986168	3.051558239	5.20E-15	1.36E-14
AC004492.1	0.445941253	0.918408612	1.042282505	4.23E-12	9.24E-12
RUNX1	1.330867568	4.475056271	1.749538805	1.61E-27	1.09E-26
PCDHGA5	0.202829452	0.43161648	1.089482794	0.003700817	0.004562421

Table S1. The differentially expressed genes(DEGs) were screened out in TCGA

ID	conMean	treatMean	logFC	pValue	FDR
AC012531.1	0.218904982	0.463267426	1.081540457	1.10E-06	1.72E-06
NFATC2	1.54546936	3.506266326	1.181890531	8.23E-23	3.74E-22
CXorf36	2.925778058	10.8789871	1.894651999	1.41E-27	9.62E-27
AL353699.1	0.925549902	0.316665739	-1.547349987	8.29E-43	4.49E-40
AC138466.1	0.511514067	0.218870144	-1.224698744	1.40E-14	3.56E-14
AC092376.2	0.173537563	0.496127797	1.515463819	3.64E-22	1.58E-21
SPC25	0.338747939	1.2127816	1.840035695	4.00E-28	2.88E-27
TMED10P2	1.248036803	0.290014011	-2.10546597	4.58E-28	3.28E-27
FAIM2	0.630381083	0.202041075	-1.641575621	2.46E-17	7.52E-17
PIMREG	0.161342184	0.779358344	2.272163135	2.81E-32	3.49E-31
HAR1A	0.104966869	0.27141485	1.370565618	1.04E-12	2.35E-12
JAK3	0.925142496	4.953011168	2.420558374	4.17E-33	6.08E-32
TLR7	0.653760812	2.853060323	2.125675444	1.31E-24	6.92E-24
IL3RA	4.988365139	13.59181659	1.446099313	3.31E-28	2.40E-27
FABP5	1.390030232	5.110482824	1.878343339	3.53E-28	2.56E-27
ZNF841	1.416332813	3.094095951	1.127357624	1.89E-20	7.20E-20
AC074124.1	0.103218206	0.722236819	2.806774507	1.96E-12	4.37E-12
AL162727.2	0.121847813	0.335702403	1.46210251	4.40E-17	1.32E-16
AC016773.1	0.107833654	0.677946824	2.652364619	1.23E-20	4.73E-20
AUH	23.52458606	10.78687134	-1.124892856	2.04E-32	2.61E-31
LCMT1-AS1	0.41879998	0.174382402	-1.264006916	1.73E-22	7.69E-22
TRBJ2-7	0.354102063	2.373454182	2.744751048	1.10E-19	3.97E-19
TUBA3D	1.026352915	8.410392006	3.034646153	2.79E-32	3.47E-31
GIMAP4	16.13566226	40.81144483	1.338720995	2.76E-27	1.83E-26
C4A	3.652500701	12.22123267	1.742433353	2.33E-19	8.21E-19
IGLV6-57	4.468005686	20.71576499	2.21302617	2.28E-06	3.49E-06
AC005034.2	0.10046658	0.236128884	1.232858856	7.18E-07	1.14E-06
NOG	0.237738849	1.549986074	2.704805674	0.003555884	0.004389528
PATL2	0.094733664	0.707778679	2.901349218	7.12E-35	1.50E-33
EVI2A	1.999889547	7.42991654	1.893425682	1.07E-28	8.16E-28
HLX	0.745428161	4.359822848	2.548128287	1.10E-35	2.85E-34
ERVK9-11	0.70537211	2.369300617	1.748004822	8.26E-17	2.43E-16
AC245884.12	0.392623813	0.948369941	1.272302262	6.63E-06	9.86E-06
FLNB-AS1	0.175709722	0.408250946	1.216262208	9.90E-07	1.55E-06
TAC4	0.101514647	0.264759268	1.382993292	3.67E-11	7.57E-11
MTHFD2	3.049140875	6.126585107	1.006680349	1.55E-17	4.81E-17
HRH1	1.60164341	3.260223493	1.025417885	9.01E-16	2.49E-15
MIR199A1	0.029208118	0.324302979	3.472900967	1.85E-10	3.65E-10
LINC02310	0.008784105	0.256274742	4.866652233	5.06E-28	3.60E-27
GABBR1	1.150887806	2.58000423	1.164626232	2.15E-07	3.52E-07
TNK2	1.355112267	3.610297541	1.413705362	7.42E-32	8.58E-31
CLEC2D	0.407064292	2.439841623	2.583458925	5.72E-37	2.29E-35
SNRPGP14	0.267718019	0.658904502	1.299355141	1.91E-08	3.33E-08
AL138899.2	0.076412959	0.231853502	1.601324287	1.20E-06	1.88E-06
CCDC81	0.087436416	0.224665158	1.361470228	2.15E-21	8.81E-21
ARHGAP30	1.908123682	7.969489049	2.062332543	1.15E-31	1.28E-30
AC006148.2	0.015891718	0.322356575	4.342310366	8.02E-19	2.71E-18
AC004540.2	6.333620961	2.140098806	-1.565353125	1.30E-27	8.85E-27
MIR1972-1	0.097426021	0.355342958	1.866833062	2.27E-06	3.48E-06
CDH3	4.301000457	0.906115368	-2.246905631	1.17E-35	3.01E-34
LINC02362	0.032315737	0.251135043	2.958154555	8.55E-21	3.34E-20
VWA2	1.127260921	0.148274917	-2.926475015	5.71E-35	1.24E-33
AC004466.3	0.166598781	0.482635368	1.534555796	7.54E-07	1.19E-06
AC008991.2	0.02153308	0.409407659	4.248911476	3.46E-13	8.10E-13
AC007497.1	0.072972847	0.271837423	1.897312429	3.19E-17	9.67E-17

Table S1. The differentially expressed genes(DEGs) were screened out in TCGA

ID	conMean	treatMean	logFC	pValue	FDR
AC103591.2	0.330474105	0.79709639	1.270216963	1.70E-14	4.31E-14
AC026250.1	0.156297453	0.51963389	1.733201256	8.78E-27	5.56E-26
SIPA1	5.759921125	12.31063644	1.095784388	2.04E-29	1.70E-28
LIMD2	1.263923631	5.080647671	2.007103126	8.39E-31	8.30E-30
PFKFB3	101.8757353	41.65925882	-1.290101396	0.039439136	0.044843041
SIGLEC14	0.703643475	2.200836907	1.645135711	2.17E-19	7.67E-19
RALBP1	39.63920847	14.36988403	-1.463879736	2.29E-38	1.52E-36
CYGB	3.984707111	16.16402176	2.020240608	2.69E-29	2.21E-28
SCG2	0.206756901	3.342029928	4.014717263	1.19E-27	8.15E-27
LRRC37A7P	0.803054989	2.524471362	1.652410626	8.73E-05	0.00012049
CD8B	0.386198107	4.244911035	3.45832132	2.84E-29	2.33E-28
HNRNPCP7	0.306213483	0.651829275	1.089956342	1.95E-13	4.62E-13
AL117335.1	0.307360112	2.085682196	2.762517492	3.56E-32	4.35E-31
IGHM	27.09973814	110.5711665	2.028624408	2.61E-09	4.82E-09
CHEK2	0.716620594	1.652296421	1.205191119	2.16E-28	1.60E-27
FAM13A-AS1	0.216505404	1.241791942	2.519948536	1.42E-29	1.21E-28
TMEM131L	1.654800676	3.664675921	1.14702817	4.22E-31	4.34E-30
TRAV13-2	0.121282795	0.650105494	2.422298941	4.92E-19	1.69E-18
TCIM	121.0886589	26.42338456	-2.196176572	7.02E-30	6.15E-29
SPRY1	12.62340775	32.46900275	1.362961649	3.26E-19	1.14E-18
ADAMTSL4	0.748160844	3.312091799	2.146322291	1.58E-29	1.33E-28
AC116407.4	0.070262868	0.287690981	2.03368562	8.05E-28	5.60E-27
YEATS2-AS1	0.080667792	0.454780835	2.495106785	4.10E-30	3.71E-29
TRAV6	0.068594009	0.376544184	2.456664669	9.46E-16	2.61E-15
FAM184A	1.213793374	0.429882782	-1.497507618	3.97E-30	3.60E-29
KDF1	8.565026236	2.013979033	-2.088409001	4.69E-37	1.94E-35
ALOX12B	0.090868373	0.354813187	1.965209486	1.93E-16	5.55E-16
RPL4P3	0.066248092	0.364626201	2.460467421	1.35E-17	4.20E-17
DES	21.21334589	7.238267128	-1.551255933	2.96E-11	6.13E-11
EEF1B2P6	0.513079303	1.082180747	1.076687745	8.02E-16	2.22E-15
SH3BGRL3	43.17038736	91.00906628	1.075968235	7.83E-23	3.57E-22
ARAP1-AS2	0.034522458	0.259971451	2.912746129	2.66E-23	1.26E-22
NTRK2	7.674335042	1.867513918	-2.038922755	1.74E-28	1.31E-27
PALM	15.80622343	5.191854036	-1.606170978	7.06E-35	1.49E-33
TNXB	2.262375688	1.055668946	-1.09968104	3.84E-17	1.16E-16
ADH1B	20.46521623	3.096128932	-2.724636454	7.11E-34	1.20E-32
OTOGL	1.261174361	0.249957505	-2.335012997	2.67E-05	3.81E-05
CD300LB	0.244598136	0.797307115	1.704722131	2.24E-25	1.26E-24
RGS18	0.687485483	2.176571798	1.662656455	4.05E-23	1.89E-22
E2F1	0.613877008	2.711799959	2.143229217	5.71E-35	1.24E-33
TMEM132A	2.294615254	5.101729312	1.152734082	1.37E-13	3.28E-13
AC091563.1	12.35485101	5.590588135	-1.144005645	1.69E-32	2.21E-31
PRKCQ	8.453836333	2.651547517	-1.67277158	4.39E-26	2.63E-25
KAZN	4.954413361	2.341622419	-1.081205778	5.58E-29	4.42E-28
TLR5	4.342319604	1.913259175	-1.182433597	1.39E-20	5.34E-20
GAS6-AS1	0.111491808	2.010099004	4.172256946	2.68E-39	2.67E-37
MIR5581	0.479895863	0.994037734	1.050579244	0.000121394	0.000166075
CEP55	0.350289878	1.757562239	2.326954574	9.63E-34	1.58E-32
GATA3-AS1	1.868654362	0.074523803	-4.648154642	1.15E-42	5.90E-40
AC092325.1	1.648129599	0.056590993	-4.864113425	1.03E-21	4.33E-21
IGLV3-27	0.951204104	4.225916517	2.151437422	0.000463089	0.000608739
AC131212.3	0.123488337	0.255878551	1.051084428	6.55E-09	1.18E-08
AP000919.4	0.11305397	0.268124583	1.245891842	5.06E-12	1.10E-11
BRWD1-AS2	1.017552271	0.331309407	-1.618851835	4.43E-28	3.18E-27
AC091390.1	0.169001031	1.517106222	3.166218146	3.60E-35	8.16E-34

Table S1. The differentially expressed genes(DEGs) were screened out in TCGA

ID	conMean	treatMean	logFC	pValue	FDR
VAMP1	1.208645467	2.450813733	1.019869721	9.19E-08	1.54E-07
SNRNP70	14.58716831	29.42471599	1.012328639	1.19E-17	3.72E-17
AC114730.3	0.105112435	0.500254576	2.250729112	1.02E-14	2.62E-14
LINC00894	0.117141069	0.545007525	2.218029179	2.86E-15	7.64E-15
DTHD1	0.025331771	0.25359307	3.323495464	4.26E-28	3.06E-27
AP002340.1	0.120917516	0.31045154	1.360344835	2.27E-08	3.94E-08
AC009133.1	0.80922559	2.517896361	1.637605053	4.33E-32	5.19E-31
HAVCR2	6.370026603	23.52073779	1.884562012	1.68E-18	5.56E-18
EXO1	0.161270118	0.525297646	1.703655979	7.07E-30	6.20E-29
AL121782.1	0.072199328	0.25322588	1.810367554	5.62E-15	1.47E-14
RHEX	4.348334465	17.28271285	1.990794874	9.06E-22	3.81E-21
CSDC2	9.100150464	3.710531283	-1.294264629	4.61E-15	1.21E-14
AK3	41.77613681	15.12056843	-1.466166711	1.50E-39	1.70E-37
AC132192.2	0.393124334	0.861511963	1.131885163	8.01E-06	1.19E-05
DISC1	0.326601612	1.055304684	1.692055773	5.87E-33	8.29E-32
LINC02586	0.944605584	0.410852761	-1.201090603	8.47E-23	3.85E-22
SPHK2	4.412089446	1.96998958	-1.16327404	1.29E-34	2.56E-33
PRELID3A	0.239516872	0.742347295	1.631967	1.29E-30	1.24E-29
STUM	3.30958114	1.531752562	-1.111465377	1.09E-15	2.99E-15
AC116667.1	0.225576628	0.594042856	1.396949418	1.63E-13	3.89E-13
AL138831.3	0.143119701	0.536279925	1.905763974	2.07E-15	5.57E-15
GULOP	0.122635273	0.345777852	1.495471469	3.38E-07	5.45E-07
AC103982.1	0.078885638	0.268765557	1.768513701	5.36E-13	1.24E-12
GAPDHP59	0.063612714	0.229796802	1.85297167	8.97E-08	1.50E-07
RIPPLY1	1.584640428	0.223548539	-2.825495493	1.54E-31	1.69E-30
PPFIA4	0.369549845	3.517686723	3.250786133	2.44E-29	2.01E-28
STK33	1.609813536	0.511475146	-1.654157548	4.82E-31	4.92E-30
HOXB-AS1	0.841338688	2.117972234	1.331925086	4.21E-18	1.35E-17
AC092338.1	0.111578252	0.428301333	1.940570306	1.49E-13	3.56E-13
HLA-DRB9	0.309947769	0.898673851	1.535772503	1.80E-11	3.79E-11
GUCY1B1	8.465010681	19.57593569	1.209497473	3.37E-29	2.74E-28
FHOD1	2.949295986	7.160100064	1.279609134	6.27E-32	7.36E-31
HDC	0.093007408	0.349956859	1.911759548	1.06E-17	3.30E-17
AC079907.1	0.148504083	0.461424425	1.635591781	3.38E-16	9.59E-16
HOXA7	3.380404974	1.194312178	-1.501016104	2.41E-30	2.24E-29
AC010531.5	0.043424322	0.244724432	2.49458291	4.56E-18	1.46E-17
PDCL3P5	0.114453043	0.350743695	1.615661349	4.89E-18	1.56E-17
EDDM13	0.115661677	0.239641417	1.050966348	0.000348296	0.000461793
HPD	114.4395138	3.977336003	-4.846639018	1.39E-24	7.31E-24
HEYL	7.11452725	22.30896922	1.648784056	2.43E-20	9.15E-20
AC122134.1	0.023159963	0.57588524	4.636076514	1.05E-33	1.71E-32
IGLJ2	0.114384091	0.39731721	1.796404877	0.00885332	0.01060832
LINC02453	0.537580933	0.236877769	-1.18233916	1.74E-27	1.18E-26
AP001010.1	0.038194448	0.306582946	3.004842584	2.03E-24	1.05E-23
MDK	7.52182584	24.04585735	1.676633558	3.42E-08	5.88E-08
MYO1G	0.345630548	2.198040497	2.668915327	8.42E-35	1.74E-33
GPR182	0.806947868	0.156122974	-2.369792624	2.29E-35	5.46E-34
C10orf55	0.939172528	0.194072759	-2.274792578	5.44E-25	2.96E-24
RNU6-1095P	0.172261533	0.487243038	1.500040999	6.60E-09	1.19E-08
RPL13P12	8.107457819	20.78481329	1.35820827	5.55E-11	1.13E-10
BCKDHB	10.61940158	4.431452988	-1.260850757	1.10E-35	2.85E-34
AP001767.3	0.207632478	0.562414852	1.437602568	6.03E-12	1.30E-11
PIGHP1	0.136174679	0.319477761	1.230257047	1.98E-11	4.14E-11
RAPGEFL1	0.331838028	1.127817051	1.76498193	1.68E-29	1.42E-28
AC064801.1	0.73152387	0.276336511	-1.40447875	6.00E-24	3.00E-23

Table S1. The differentially expressed genes(DEGs) were screened out in TCGA

ID	conMean	treatMean	logFC	pValue	FDR
BIRC3	5.138881764	25.90594016	2.333756578	1.64E-29	1.38E-28
PMEPA1	8.418645319	25.88378275	1.620388466	1.25E-24	6.63E-24
AC099811.4	0.039752076	0.252464404	2.666977876	2.65E-10	5.17E-10
VIP	0.100782698	0.450943659	2.161699212	9.95E-14	2.40E-13
AL731563.1	0.020923057	0.263556299	3.654945635	5.90E-14	1.45E-13
IDH2	133.58927	61.13909096	-1.127637127	7.68E-26	4.50E-25
AC007611.1	0.137334498	0.328374288	1.257647093	4.88E-26	2.91E-25
LRRC7	0.084792912	0.250492213	1.562750188	1.04E-08	1.85E-08
PDXK	21.94630433	10.24707435	-1.098765952	1.12E-21	4.68E-21
IGLV5-52	0.050711024	0.3164373	2.641548368	4.58E-09	8.30E-09
AP000692.1	0.192446729	0.716232717	1.895969272	2.00E-18	6.56E-18
IGLV4-3	0.073914308	0.297659233	2.00973609	0.009128675	0.010927446
RPL21P54	0.113647491	0.229109209	1.011469611	7.19E-06	1.07E-05
MIR509-2	0.020975217	0.358844878	4.09660271	1.32E-13	3.16E-13
PARP15	0.095127862	0.638959801	2.74778531	4.82E-31	4.92E-30
AC010319.1	0.095278534	0.388062927	2.0260675	2.99E-28	2.18E-27
SYTL4	3.770526444	1.686548787	-1.160691916	1.32E-23	6.41E-23
CCDC18	0.258530607	0.637633664	1.302392711	5.29E-22	2.26E-21
TMSB10	602.5835472	2548.010179	2.080137853	1.48E-35	3.71E-34
CROT	8.556267792	3.563531557	-1.263673936	2.54E-39	2.57E-37
HLA-DRB1	205.239071	640.0057864	1.640779549	4.50E-28	3.23E-27
ENGASE	1.805922322	3.818675755	1.080336585	5.75E-14	1.41E-13
SEMA3B	11.76148754	4.609254218	-1.351465292	1.93E-28	1.44E-27
AL391994.1	0.078117904	0.262832	1.750415786	2.31E-09	4.28E-09
BST2	54.71206903	134.9449007	1.302439442	7.78E-23	3.54E-22
PALM3	15.46477183	4.164686495	-1.892705745	2.40E-31	2.56E-30
C2orf15	2.981314942	0.819515768	-1.863105175	6.14E-38	3.54E-36
CD8A	0.902661944	11.31597051	3.648030728	7.87E-32	9.04E-31
AC007342.5	0.902256864	0.155843806	-2.533437397	3.76E-36	1.10E-34
P2RY14	0.934705558	2.074994699	1.150523773	3.09E-13	7.24E-13
PSMB8	23.5900901	72.09384719	1.61169521	1.60E-39	1.79E-37
TRAV14DV4	0.124019138	0.70335574	2.503691783	4.07E-19	1.41E-18
LINC01954	0.746078342	0.35486099	-1.072073142	0.000188356	0.000254337
LRRC66	0.203381476	0.811747103	1.996842043	3.03E-22	1.32E-21
AC009120.1	0.730694297	2.358117531	1.690295773	1.61E-16	4.66E-16
LINC01336	0.702346083	0.304701819	-1.204783984	1.47E-25	8.42E-25
LAPTM5	17.44166918	101.6070442	2.542390406	1.45E-36	5.01E-35
RAB11FIP1P1	0.322021564	0.902723143	1.487126294	4.37E-13	1.02E-12
SSPN	3.265431806	7.386656117	1.177647635	1.32E-22	5.91E-22
AC010463.3	0.11836407	0.314820465	1.411298114	7.37E-11	1.49E-10
KCTD13	1.461181443	3.00140151	1.038500991	1.74E-29	1.46E-28
AC018638.1	1.077628308	3.025687846	1.489403501	2.50E-19	8.79E-19
AC090527.3	0.098437373	0.221189905	1.168007479	4.18E-05	5.88E-05
RNU6-10P	0.036993042	0.311285262	3.072911412	2.46E-11	5.13E-11
AL121899.1	0.289443002	0.790484306	1.449457542	1.87E-11	3.91E-11
RPL23AP18	0.157185741	0.320771113	1.029073877	1.07E-07	1.78E-07
AC092279.2	0.170190622	0.439172291	1.367635491	1.40E-07	2.32E-07
MCCD1	21.57575429	0.998619203	-4.433332538	1.78E-40	3.33E-38
GPR20	0.28617994	0.728449682	1.347906772	4.43E-08	7.56E-08
AL132780.1	1.342400154	0.406507441	-1.723461121	3.87E-25	2.13E-24
VWFP1	0.13309167	0.854219307	2.682186226	6.04E-17	1.79E-16
OCLM	0.130457288	0.449684878	1.785336822	1.11E-15	3.05E-15
AC104758.2	0.103819454	0.330996036	1.672737134	9.82E-16	2.70E-15
ADGRG3	0.114641721	0.371275757	1.695358945	3.21E-11	6.64E-11
FOXM1	0.435162837	2.359437653	2.438815788	4.56E-34	8.00E-33

Table S1. The differentially expressed genes(DEGs) were screened out in TCGA

ID	conMean	treatMean	logFC	pValue	FDR
AC019257.1	0.414670791	2.352645847	2.504245828	7.20E-24	3.56E-23
SUZ12P1	0.82686225	1.668476903	1.012812805	1.76E-14	4.45E-14
ETV4	1.055346785	0.421475562	-1.324196254	4.04E-26	2.43E-25
IRS1	5.109214208	2.455880879	-1.056860838	3.36E-25	1.86E-24
BDKRB2	8.286683708	3.59522943	-1.204711018	2.28E-25	1.28E-24
DSCAML1	0.991171853	2.51063886	1.340847397	7.54E-17	2.23E-16
HLA-U	0.988192049	3.252757761	1.718800032	2.72E-14	6.81E-14
CLMP	2.752997482	1.304185427	-1.077854287	1.43E-23	6.92E-23
KCNH3	0.711739521	0.181763144	-1.969289648	1.17E-34	2.33E-33
LMBR1L	2.073119292	5.576572156	1.427575455	1.29E-34	2.56E-33
IFNWP19	0.882933873	0.330456723	-1.417844042	8.17E-18	2.57E-17
ASAP1	4.152569333	8.760192432	1.076958305	1.14E-25	6.56E-25
CARMN	0.107672238	0.409489687	1.9271808	5.49E-12	1.19E-11
AC002070.1	0.834902158	2.041992126	1.290298259	1.31E-23	6.37E-23
TNFSF9	0.555273832	6.020362831	3.438579124	6.92E-34	1.17E-32
MIR148A	0.054979621	0.234255497	2.091114042	1.73E-06	2.67E-06
AL157935.2	0.091951719	0.416358534	2.178877951	7.17E-20	2.62E-19
EBI3	0.982246128	4.88358894	2.31378529	1.62E-33	2.53E-32
B4GALNT1	0.19358754	2.863771179	3.886858222	2.96E-28	2.17E-27
NUDT10	1.582994853	0.253464574	-2.64280054	2.03E-36	6.53E-35
ATG9B	0.112389562	0.869842456	2.952246071	2.52E-29	2.07E-28
AC124312.5	3.761218303	1.705030134	-1.141402807	5.38E-26	3.20E-25
PPIAP53	0.34722864	0.793384634	1.192134507	1.52E-11	3.21E-11
AC093110.1	0.407161811	1.089928141	1.420558862	1.34E-13	3.21E-13
GK-AS1	0.18814117	0.639210314	1.764475109	2.78E-14	6.96E-14
DPP6	1.305551346	0.131778975	-3.308467081	6.18E-33	8.69E-32
LDHAP4	1.612061846	4.581599986	1.506944411	2.42E-17	7.39E-17
FAM220CP	0.510819982	0.185548202	-1.461020942	0.000675886	0.000878592
RPS19	72.31161458	194.8100333	1.429768688	2.36E-35	5.57E-34
PRKD1	8.566402708	4.184863745	-1.033508849	3.98E-31	4.11E-30
AP001148.1	1.171779296	0.575731105	-1.0252338	8.79E-25	4.71E-24
AP002954.1	0.107101301	0.779962553	2.864428852	1.54E-24	8.07E-24
IGHGP	4.404423918	22.86642407	2.376205532	2.81E-06	4.29E-06
AC010680.4	0.109834749	0.328268772	1.579542957	1.29E-10	2.57E-10
AC010240.2	0.104761453	0.278495293	1.410544969	0.000264042	0.000353226
LGALS1	59.06596569	252.1866685	2.09409303	3.70E-34	6.63E-33
RPS11P5	0.959456965	1.934884773	1.01195765	2.29E-18	7.50E-18
AC027601.1	0.052105447	0.28295912	2.441087541	1.49E-24	7.85E-24
RSPO3	2.295701982	0.89682058	-1.35604408	1.68E-15	4.55E-15
NCBP2-AS1	0.067544097	0.234796234	1.797507666	5.07E-14	1.25E-13
KMT5C	0.617078629	1.503385227	1.284688495	1.70E-24	8.89E-24
CCDC160	4.88782461	0.94864699	-2.365249281	1.97E-37	9.30E-36
AL096712.1	0.059367579	0.252821631	2.090372708	0.000411272	0.000542565
SDHAP1	1.284660964	3.27369155	1.34953073	2.73E-15	7.31E-15
AC018804.1	0.118054274	0.476517166	2.013077917	2.14E-11	4.47E-11
AC092119.2	0.153225191	0.659499182	2.10571737	8.33E-15	2.15E-14
AL157394.1	0.378047322	1.050794883	1.474842339	6.14E-29	4.85E-28
RND2	1.044272865	0.513228335	-1.024826006	4.54E-23	2.11E-22
AL359075.1	0.149573527	0.38383898	1.359646373	7.37E-05	0.000102296
AC104046.1	0.074177068	0.250893757	1.758031418	5.06E-11	1.03E-10
AL035405.1	0.026875136	0.274541183	3.352678622	0.001970605	0.002478819
TCTEX1D4	0.126088543	0.268279875	1.089301642	3.91E-09	7.12E-09
SLC17A4	0.937143787	12.5323201	3.741239295	9.89E-21	3.84E-20
1-Mar	0.712775113	2.191787105	1.620588802	1.33E-22	5.95E-22
SEC14L1P1	0.206899766	0.42635942	1.043138119	5.44E-18	1.73E-17

Table S1. The differentially expressed genes(DEGs) were screened out in TCGA

ID	conMean	treatMean	logFC	pValue	FDR
AC009084.1	0.064694354	2.012670838	4.959327615	1.71E-26	1.06E-25
GABRP	2.220298322	0.482186992	-2.203088894	3.31E-29	2.69E-28
CPEB3	3.246758369	1.293468214	-1.327755419	1.74E-34	3.34E-33
PDE1B	0.777354383	2.45399052	1.658485321	3.03E-28	2.21E-27
LINC02568	1.679222575	0.177566721	-3.241360339	6.99E-38	3.89E-36
AL360091.1	0.128205817	0.319316424	1.316525041	8.21E-05	0.000113502
AC010480.1	0.013211619	0.268645435	4.345824127	1.94E-07	3.18E-07
AL138787.2	0.087626931	0.23796214	1.441285826	6.37E-07	1.01E-06
RNA5SP37	0.413256671	0.855860018	1.050336743	1.10E-05	1.61E-05
LRRC4B	0.529021099	1.069738782	1.015861382	2.08E-11	4.35E-11
ANGPT2	2.2648326	19.27621162	3.089345219	9.42E-36	2.50E-34
SNORA28	0.13995554	1.242826388	3.150584279	0.000154848	0.00021036
FATE1	0.164149668	1.919932372	3.547971753	1.20E-37	6.14E-36
DSCC1	0.594283	1.191201707	1.00319571	6.50E-25	3.51E-24
AC010615.2	0.102957282	0.28510723	1.469458754	1.84E-20	7.02E-20
F2RL3	1.866981751	9.903045003	2.407164369	4.80E-22	2.06E-21
NADSYN1	1.993024972	4.211279179	1.079298732	1.29E-33	2.06E-32
TEX41	0.328871483	0.857510939	1.382631161	0.000921537	0.001188239
AC096541.1	0.132821227	0.50128275	1.916138863	2.46E-24	1.27E-23
IGKV1-6	3.616319605	13.51034496	1.90147042	0.000454082	0.000597166
LRRC39	0.199670994	0.867277457	2.118868844	7.73E-22	3.27E-21
ECI2	30.015659	14.56816692	-1.042895985	2.68E-30	2.48E-29
SNAP25	0.645310559	2.606078364	2.013814929	2.12E-19	7.48E-19
SNX33	5.5353615	14.05979369	1.344825981	1.66E-34	3.19E-33
NAPEPLD	8.277413306	3.729551504	-1.150177845	8.64E-35	1.78E-33
AL136528.1	0.040002573	0.366775542	3.196732726	1.62E-15	4.40E-15
Z84484.1	0.129546329	0.455585185	1.814252696	5.74E-19	1.96E-18
IGHV1OR21-	0.076519418	0.391076033	2.353551311	0.026750421	0.030839829
BNIP3P22	0.053897708	0.54748761	3.344530485	4.22E-13	9.83E-13
AP001160.1	0.194224708	0.655605872	1.755102036	4.58E-16	1.29E-15
PDIA2	0.03504275	0.332661282	3.246866049	0.00505491	0.006164218
SLC26A4	3.11408057	0.318452459	-3.289656354	1.36E-11	2.88E-11
AL360181.2	0.624783126	2.160390777	1.7898649	2.09E-18	6.85E-18
GLIS1	1.138620787	4.359423592	1.936850049	1.83E-24	9.55E-24
MRO	6.735794965	0.91192082	-2.884867756	4.83E-20	1.79E-19
RPS15AP1	1.551451488	3.112718314	1.00455644	1.13E-16	3.30E-16
LRRC43	1.262936783	0.449585923	-1.490113656	2.88E-30	2.66E-29
EP400P1	0.333783281	0.699528111	1.06747034	1.81E-11	3.81E-11
RNU6-623P	0.076443899	0.35260354	2.205573679	1.91E-11	4.01E-11
AL031658.1	0.081078745	0.291021863	1.843731877	1.25E-13	3.00E-13
AL109615.2	0.013303012	0.393285501	4.885752198	2.58E-33	3.88E-32
LYPD8	1.189275474	0.440668566	-1.432317032	9.52E-23	4.31E-22
CLCNKB	102.1311302	8.54066354	-3.579930706	8.32E-38	4.52E-36
RPL17P34	0.118132588	0.252009673	1.09307211	1.24E-10	2.48E-10
AC105020.5	0.289827802	1.055407806	1.864532659	2.15E-21	8.81E-21
LINC00475	0.058809384	0.796684891	3.759890921	3.25E-33	4.81E-32
AC006160.1	0.112001637	0.393050196	1.811193751	1.99E-17	6.11E-17
AC073218.1	1.673591173	16.69444367	3.318348965	1.78E-29	1.49E-28
AL033397.2	0.065993166	0.291547895	2.143344378	7.60E-18	2.40E-17
PLA2G4A	6.517388921	2.156617862	-1.595523526	8.22E-26	4.81E-25
FGFBP1	0.808438407	0.129361457	-2.64373003	2.90E-39	2.80E-37
AL080317.1	1.512871275	10.98623205	2.860335526	1.87E-30	1.77E-29
PLAG1	0.776258156	0.245020357	-1.663634903	1.43E-33	2.26E-32
AC111000.2	2.205802839	9.93726236	2.171544611	2.70E-15	7.22E-15
CBFA2T3	0.342484748	0.719063949	1.070080339	1.55E-15	4.21E-15

Table S1. The differentially expressed genes(DEGs) were screened out in TCGA

ID	conMean	treatMean	logFC	pValue	FDR
P2RY12	0.478947898	1.637549988	1.773598319	3.12E-17	9.48E-17
HIGD1B	2.076005817	6.529603948	1.653185002	2.31E-18	7.55E-18
HDAC11	9.083866431	4.278490318	-1.086204667	1.38E-29	1.17E-28
TRIM36	0.162370509	0.394504827	1.28075333	2.24E-21	9.16E-21
AL354751.2	0.011080791	0.284266973	4.681113772	5.95E-17	1.77E-16
PDGFD	6.480145444	24.03746681	1.891186766	1.07E-23	5.23E-23
HOXD1	0.936789015	0.410660962	-1.189776349	1.42E-24	7.47E-24
SLC48A1	18.46886875	6.686247807	-1.465826771	2.89E-39	2.80E-37
AC007326.1	0.045200322	5.410881725	6.903386843	1.68E-37	8.15E-36
PAEP	0.010618538	6.658253526	9.292414907	2.82E-13	6.64E-13
UBA52P5	0.191854102	0.553214852	1.527830275	7.74E-13	1.77E-12
SNORD124	0.107708433	0.583803879	2.438352584	2.88E-14	7.20E-14
MIR1302-3	0.225390879	0.460290774	1.030116393	0.009432898	0.011276536
RPL9P18	0.126469896	0.256815733	1.021939569	1.47E-08	2.59E-08
GABRQ	0.17993072	0.488755025	1.441670018	2.10E-14	5.29E-14
AC007016.1	0.059794261	0.393515102	2.71834008	6.23E-19	2.12E-18
RPL18	68.24917944	147.9483398	1.116209902	1.91E-31	2.07E-30
SFTA1P	0.055659013	1.74793422	4.972891763	5.13E-40	7.48E-38
ACPP	12.7747708	0.272862307	-5.54898048	3.10E-42	1.36E-39
ICAM3	0.250033743	1.014098726	2.020003403	5.31E-28	3.77E-27
RUBCNL	0.258648188	0.74376568	1.523857094	2.66E-21	1.08E-20
CIITA	1.230969225	3.498989709	1.507143727	2.29E-21	9.35E-21
TSPYL5	12.62530863	4.062507463	-1.635876286	2.32E-31	2.48E-30
ABCA8	2.512803188	0.663691116	-1.92071381	1.07E-19	3.86E-19
BICDL1	29.6328196	7.271615195	-2.026848146	1.33E-32	1.77E-31
AL596325.2	0.317326248	0.875754048	1.46455889	8.41E-09	1.50E-08
AL031777.1	0.107208707	0.403054286	1.910552081	1.78E-11	3.74E-11
CHL1	6.325989149	0.617426192	-3.35695249	1.08E-39	1.34E-37
RNA5SP25	0.030514588	0.331795965	3.442725329	2.57E-08	4.45E-08
LINC02535	2.784083629	0.99265732	-1.487834878	0.000396608	0.000523796
POU5F1	1.138772681	12.5255037	3.459316929	2.74E-35	6.40E-34
AC007490.1	1.750346536	0.577197223	-1.600504313	4.64E-28	3.32E-27
ARHGAP22	0.187419152	1.230492509	2.714895583	2.16E-38	1.45E-36
AIF1L	235.4903638	25.14391291	-3.227386941	1.85E-40	3.40E-38
SNX20	0.342532793	1.902300351	2.473431029	2.11E-31	2.27E-30
TSPAN33	77.48068181	25.92135175	-1.579695695	5.68E-33	8.03E-32
AC104837.2	0.02900484	0.237613179	3.0342493	3.06E-17	9.30E-17
FMO5	7.16317225	1.359321053	-2.397712393	1.80E-37	8.69E-36
AP002761.4	0.838323701	0.34021073	-1.301078774	3.62E-23	1.69E-22
INSRR	1.049377112	0.086549548	-3.59986314	2.06E-31	2.22E-30
IGHV3-64	0.367105422	2.127335659	2.534781359	8.66E-06	1.28E-05
TNNI1	1.875642287	0.119198764	-3.975943533	1.71E-39	1.90E-37
YBX3	12.44101939	35.18160207	1.499716479	6.36E-36	1.77E-34
AC243562.1	0.107661392	0.219705857	1.029072344	0.00236882	0.002961816
LINC01159	2.26319711	0.447639246	-2.337953807	1.28E-31	1.42E-30
CNDP1	0.5200034	0.231286482	-1.16884011	1.94E-10	3.81E-10
GPR85	0.203972361	0.567073716	1.475162613	1.08E-19	3.91E-19
RASSF10	3.562484723	0.418156987	-3.090767252	1.32E-38	9.64E-37
AC099343.2	0.198281827	0.426932124	1.106454264	0.002173948	0.002724946
AC124944.2	0.107699309	0.818837219	2.926567687	2.21E-25	1.24E-24
RNU6-1005P	0.094249676	0.392754078	2.059066693	1.38E-08	2.43E-08
NTNG1	1.948527505	0.09022643	-4.432690382	8.71E-33	1.19E-31
CLEC4A	1.019567549	3.370871741	1.725164375	2.33E-30	2.17E-29
MIR4677	0.090377385	0.399194349	2.143057584	1.43E-08	2.51E-08
IL15	0.639172143	1.312437882	1.037972703	4.94E-23	2.29E-22

Table S1. The differentially expressed genes(DEGs) were screened out in TCGA

ID	conMean	treatMean	logFC	pValue	FDR
CXXC4	0.403872228	0.84019481	1.056824931	2.70E-08	4.68E-08
AC008033.3	0.134335614	0.963922257	2.843074965	1.73E-16	5.00E-16
RNU1-106P	0.08387602	1.43105331	4.092675203	4.90E-38	2.92E-36
RN7SKP70	0.440695764	1.528459802	1.794223674	2.31E-17	7.08E-17
ZBTB40-IT1	0.110619764	0.368959642	1.737853841	8.30E-13	1.90E-12
SLC34A1	38.70892885	1.961465431	-4.302662571	3.35E-14	8.34E-14
PRPH2	0.658782775	0.280344736	-1.232600851	2.20E-23	1.05E-22
AC112243.1	2.510130556	0.00456142	-9.104063777	2.89E-57	7.44E-54
HLA-DRB5	82.19951569	212.7735843	1.372117253	1.20E-14	3.07E-14
SRGAP3	1.62807294	0.296547477	-2.456830332	1.92E-38	1.31E-36
AL137847.1	1.070414007	0.494232317	-1.114907646	6.19E-23	2.85E-22
AC007365.1	0.194745243	0.408367477	1.068279889	7.43E-12	1.60E-11
AC009704.2	0.150205429	0.940764571	2.646896768	1.69E-20	6.47E-20
OXGR1	2.743162946	0.375384016	-2.869401197	9.61E-37	3.54E-35
RNA5SP39	0.087777898	0.342041206	1.962240512	9.95E-08	1.66E-07
LNCSRLR	0.26932953	1.394997038	2.372817735	1.37E-27	9.33E-27
NPIPB4	0.102648719	0.427041755	2.056661514	2.56E-26	1.57E-25
PKHD1	9.156717458	4.03398231	-1.18262575	2.80E-26	1.71E-25
AL137026.1	0.062430838	0.473538851	2.923152049	2.90E-09	5.33E-09
AC116407.2	0.225808339	0.84806892	1.909082748	2.59E-24	1.33E-23
HSD3B7	6.777910042	31.08186301	2.197160585	9.71E-32	1.10E-30
AL357874.1	0.067580107	0.228662368	1.758548421	2.96E-18	9.61E-18
CTSE	0.268338036	1.576072224	2.554210179	4.03E-07	6.47E-07
GATA2	9.248065569	3.141012732	-1.557921836	2.79E-30	2.58E-29
COL5A1	3.042497317	12.15200668	1.997866672	2.37E-18	7.74E-18
SLC8A1	4.233672193	1.735050851	-1.286931622	1.47E-06	2.28E-06
PAIP1P1	0.099654756	0.353393898	1.82626657	1.30E-16	3.78E-16
LINC00942	0.035330316	0.551311682	3.963889617	0.009517375	0.011375542
AC098934.1	1.56642688	0.336224979	-2.219978613	2.29E-15	6.15E-15
NPEPL1	1.134531339	3.173114866	1.483803287	1.90E-20	7.24E-20
GCGR	5.430422043	0.7829299	-2.79410928	2.20E-36	6.96E-35
TREM1	0.2352162	1.03368845	2.135742099	3.77E-17	1.14E-16
AC118755.1	0.035304736	0.340843746	3.271176879	1.21E-05	1.76E-05
AC011297.1	2.367640214	0.196664734	-3.589639682	2.49E-14	6.25E-14
ARHGAP26	2.184870306	4.630859985	1.083732494	8.75E-24	4.29E-23
TMEM52B	64.95063176	1.942802689	-5.063132265	4.86E-36	1.39E-34
CDKL1	3.044271851	1.058873901	-1.523566404	7.00E-32	8.15E-31
AL513008.1	0.042307088	0.424146007	3.32558969	1.59E-11	3.34E-11
REG1A	5.419557857	37.31737496	2.783600443	2.82E-10	5.51E-10
AL365356.4	0.085151695	0.766997604	3.171114923	3.05E-32	3.76E-31
RPLP0P2	0.501794691	0.182038315	-1.46285507	6.26E-17	1.86E-16
LRRC37A6P	1.007007514	0.234723356	-2.101041138	1.16E-33	1.87E-32
MAP3K14	2.977203307	7.857882948	1.400182934	1.32E-26	8.24E-26
NCF2	2.062473179	6.994414958	1.761828031	8.44E-28	5.86E-27
MIR4701	0.224520956	0.768787907	1.775735539	4.75E-07	7.60E-07
MAL	329.7434364	29.54487768	-3.480364011	1.91E-39	2.03E-37
PITPNM3	0.833350431	0.306625001	-1.442447954	1.96E-27	1.31E-26
ARRB2	5.009289611	14.29244323	1.512574632	6.92E-40	9.19E-38
AL136380.1	0.1362943	0.803176303	2.558991476	1.53E-17	4.74E-17
LINC02432	1.300241627	0.18074666	-2.846738854	4.60E-34	8.05E-33
AC084117.1	0.236447428	2.19738922	3.216199081	2.69E-27	1.79E-26
FTCD	20.1618415	9.054115931	-1.15498173	7.69E-05	0.000106559
TMEM74B	0.221149585	2.937309341	3.731400768	8.63E-41	2.02E-38
TMEM92	0.430332932	2.821656251	2.713017087	2.97E-16	8.44E-16
DNAJB13	0.087978912	1.182662321	3.748736642	4.92E-18	1.57E-17

Table S1. The differentially expressed genes(DEGs) were screened out in TCGA

ID	conMean	treatMean	logFC	pValue	FDR
PDGFRL	0.789984438	1.8177989	1.202296467	0.001513956	0.001922964
CMTM3	5.356410861	15.09815297	1.495033537	1.47E-29	1.24E-28
AC026369.3	0.028990475	0.419224266	3.854071335	1.62E-28	1.22E-27
AC078906.1	0.016378799	0.341561031	4.382241881	5.99E-32	7.07E-31
CCND2	4.796099144	10.94468323	1.190296812	1.74E-19	6.19E-19
SULT1C2P2	0.074004006	0.25183722	1.766816253	1.42E-05	2.06E-05
TACC3	1.065821556	2.898830202	1.443504914	3.01E-29	2.46E-28
LINC01014	0.576123304	0.166149918	-1.793892037	7.05E-25	3.80E-24
RAC2	4.125681333	17.26656379	2.065276704	3.53E-29	2.86E-28
GNGT1	0.128110243	0.613884308	2.260580965	2.64E-05	3.77E-05
SELP	7.175079697	3.318397114	-1.112508312	5.64E-21	2.23E-20
EIF3KP1	0.16355123	0.361919573	1.145926525	2.09E-08	3.65E-08
AL161719.1	0.753366417	0.191812089	-1.973658076	2.93E-19	1.02E-18
AC005253.1	0.241345153	0.82706465	1.776902254	4.04E-20	1.50E-19
DNM1	1.331132006	3.704657155	1.476686388	1.22E-11	2.59E-11
RAP2B	4.460961625	10.1787358	1.190131747	2.09E-30	1.97E-29
AL355803.1	0.97642176	2.494071213	1.352926307	2.59E-16	7.39E-16
AL354836.1	1.377484261	4.828557104	1.809556304	5.64E-18	1.80E-17
GBP2	6.270769403	24.73639289	1.979920766	9.52E-35	1.94E-33
AC007406.1	0.398160096	1.270501995	1.673978097	2.41E-05	3.46E-05
GGT6	29.37386549	0.964887929	-4.928027937	3.39E-40	5.29E-38
AC009120.3	0.490426906	1.394818293	1.507967155	1.20E-12	2.71E-12
TMEM238L	6.459414075	0.046910823	-7.105338689	4.59E-41	1.23E-38
SYN2	0.660790483	0.138703664	-2.252187015	1.15E-26	7.21E-26
NEDD4L	13.27887514	6.341286256	-1.066285531	4.40E-32	5.27E-31
GP6	0.066037686	0.314418408	2.251324202	7.95E-21	3.11E-20
GPBAR1	1.869285747	0.574612455	-1.701825952	7.98E-06	1.18E-05
TRAV1-1	0.037999211	0.228868729	2.590478984	7.94E-14	1.93E-13
NOTCH4	4.496523375	13.7228948	1.609702972	4.29E-23	2.00E-22
AL137784.2	0.129461135	0.28759737	1.151531426	1.66E-10	3.29E-10
ST6GAL1	62.30450014	13.3692088	-2.220422282	5.32E-37	2.16E-35
OPHN1	3.431152375	1.086067134	-1.659579912	9.86E-37	3.61E-35
RN7SL521P	0.140917267	0.416038009	1.561866934	0.001034527	0.001329266
LINC02446	0.0577345	0.658960558	3.512686529	6.62E-28	4.66E-27
AP000873.4	0.13111748	0.506492122	1.949679793	4.73E-18	1.51E-17
IGLV2-18	1.164087098	5.19661036	2.158371884	6.29E-05	8.76E-05
AP002518.2	0.03086171	0.2307015	2.902137473	7.59E-12	1.63E-11
BMP8A	0.213770845	0.581148867	1.442842664	3.12E-15	8.30E-15
UBE2CP2	0.127933602	0.338817309	1.405112339	3.71E-13	8.66E-13
FHOD3	8.254974528	3.482104278	-1.245304356	2.56E-32	3.23E-31
MLKL	1.190642764	4.617439406	1.955352412	3.94E-37	1.67E-35
CXorf21	0.672212811	2.154950442	1.680664749	7.91E-24	3.90E-23
FANCI	0.739965921	1.814149772	1.293762832	1.75E-31	1.90E-30
CEBPA	1.50495391	4.269424801	1.504322411	1.49E-18	4.95E-18
LRFN1	0.263529652	0.605289303	1.199659555	1.73E-21	7.13E-21
AC005393.1	0.201999569	0.436024734	1.110057764	4.26E-09	7.74E-09
ESRP1	7.651884918	0.827468709	-3.209038514	1.72E-36	5.66E-35
PLD4	0.382278107	1.878126168	2.296599498	7.56E-29	5.89E-28
RNU6-1165P	0.199747332	0.588708759	1.559377859	2.50E-08	4.33E-08
FOLH1	3.650400113	7.396661172	1.018819587	2.73E-11	5.66E-11
XKR9	0.184435708	0.406128732	1.138819096	1.69E-06	2.61E-06
MIR548AN	0.081081994	0.693958818	3.09739657	5.78E-17	1.72E-16
ZNF90	0.410863332	0.855399356	1.05793954	5.72E-08	9.69E-08
TMC4	26.97228763	6.029732434	-2.161311997	1.48E-32	1.95E-31
MEIS3	0.561016217	1.495021549	1.4140519	4.68E-19	1.61E-18

Table S1. The differentially expressed genes(DEGs) were screened out in TCGA

ID	conMean	treatMean	logFC	pValue	FDR
BCAT1	0.887504932	2.402357403	1.436623758	1.69E-17	5.23E-17
AC009159.2	0.089243674	0.732248935	3.036512374	8.60E-28	5.97E-27
RNA5SP40	0.04927061	0.63292346	3.683231808	2.72E-10	5.30E-10
AC008735.2	0.60931559	3.237210055	2.409489424	7.61E-20	2.78E-19
RNU5B-4P	0.554499381	1.261793643	1.186218234	5.14E-12	1.12E-11
LINC00426	0.065963634	0.407551855	2.627240844	6.09E-28	4.30E-27
IGKV6D-21	0.40939284	1.818111987	2.15088329	0.001771069	0.002235608
APCDD1L-D	1.075267417	0.332487014	-1.693325601	1.02E-32	1.39E-31
SEPT4	2.167871206	4.889803678	1.173497495	1.55E-18	5.14E-18
NPHS2	70.15892911	0.552262563	-6.989128583	5.62E-27	3.62E-26
RASA3	3.363749694	9.005959812	1.420809685	2.32E-31	2.48E-30
AC132938.5	0.093328151	0.379528953	2.023825734	1.30E-15	3.56E-15
AC104699.1	0.05716606	0.29767485	2.380506583	1.75E-08	3.06E-08
ENOX1	2.132766885	0.588526596	-1.857546766	5.22E-26	3.11E-25
EPN3	5.188636457	0.331475622	-3.968380778	2.56E-39	2.58E-37
SEMA3F	11.20445658	24.62551571	1.136081258	6.10E-24	3.04E-23
GAS5	14.87308425	48.29328388	1.699118716	7.95E-34	1.33E-32
MELTF	7.190304461	1.172231897	-2.616794861	1.96E-13	4.65E-13
S100A2	44.94602435	3.644959574	-3.62421879	4.13E-39	3.64E-37
FAM124A	2.279548493	1.056733526	-1.109136478	1.30E-33	2.07E-32
PSTPIP1	0.267624642	1.873409795	2.807383647	8.31E-34	1.39E-32
AL365330.1	0.731326394	1.494122578	1.030711175	8.46E-11	1.70E-10
TNFRSF18	0.155185162	1.163310576	2.906173788	2.65E-32	3.32E-31
GTF2IP9	0.33922041	0.691288415	1.027064774	5.28E-06	7.91E-06
TUBAL3	2.369958628	0.162240092	-3.868659597	3.17E-27	2.09E-26
CYP4B1	0.883420445	0.298382399	-1.565937787	6.80E-18	2.15E-17
ZNF204P	9.887552153	4.534189526	-1.124768703	4.63E-27	3.01E-26
DGCR10	0.155184998	1.988954659	3.679949391	7.53E-35	1.58E-33
AC253536.3	0.40278369	0.844068359	1.06735458	0.000392266	0.000518156
AC093908.1	0.106874047	0.431701506	2.014122566	2.51E-28	1.85E-27
ARHGAP15	0.680641636	2.127802768	1.644397118	4.05E-29	3.26E-28
ALG13-AS1	0.229273251	1.059664213	2.20846722	3.30E-21	1.33E-20
AC083967.1	0.011282092	0.610006114	5.756717163	3.79E-29	3.05E-28
Z98257.1	0.009198857	0.225992518	4.618676608	1.38E-19	4.94E-19
SLAMF1	0.249723053	0.722332122	1.53233332	1.17E-17	3.65E-17
ARHGEF38	1.129058856	0.41547599	-1.442283683	3.17E-27	2.09E-26
AC004837.2	0.080245995	0.270365897	1.752411882	1.10E-12	2.50E-12
CD200R1	0.227560597	1.144087666	2.329874925	1.09E-22	4.92E-22
AC135048.4	0.154308769	1.205756734	2.966046909	1.22E-33	1.95E-32
DMRT2	11.78343835	3.130501005	-1.912295102	1.40E-35	3.53E-34
CLEC17A	0.080542217	0.234000124	1.538692216	5.32E-11	1.08E-10
SLC31A1P1	0.10344062	0.228725314	1.144813219	0.000119986	0.000164182
COL4A5	6.233219764	1.269149205	-2.296115892	3.94E-39	3.51E-37
LINC00284	2.061569184	0.335522709	-2.619260557	4.86E-33	7.00E-32
MYH14	9.389518944	2.638070607	-1.831568067	3.28E-32	4.02E-31
DDX39BP1	0.061970447	0.277506452	2.162869031	2.44E-11	5.08E-11
PIP4K2C	32.97064292	12.32394639	-1.419717708	1.01E-40	2.23E-38
AP001107.4	0.200963108	0.762355744	1.923533694	5.62E-13	1.30E-12
RNA5SP298	0.079860484	0.375795519	2.234394149	2.64E-10	5.16E-10
AC093724.1	0.697388461	1.421040974	1.026913754	5.63E-12	1.22E-11
MFSD3	11.96106775	5.551290562	-1.10745107	5.66E-27	3.65E-26
AC012404.2	0.034536514	0.49043401	3.827864671	2.12E-13	5.03E-13
PCDHB14	1.168998431	3.112251861	1.412685823	1.15E-22	5.19E-22
AL035071.2	0.128561259	0.2689395	1.064825698	0.002466291	0.0030792
MB	0.109955377	0.235326235	1.097744011	0.018615508	0.021736494

Table S1. The differentially expressed genes(DEGs) were screened out in TCGA

ID	conMean	treatMean	logFC	pValue	FDR
AC084880.3	0.135912057	1.608491711	3.564963148	5.16E-30	4.61E-29
Z99289.1	0.03301156	0.351164036	3.411101863	3.31E-30	3.03E-29
AKR7A2P1	0.035126332	0.336592543	3.260378383	4.92E-30	4.41E-29
AL845552.1	0.028714454	0.230873458	3.007253305	7.62E-06	1.13E-05
RNA5SP449	0.049439244	0.238119728	2.267958553	3.78E-11	7.78E-11
AC135178.5	0.300388687	0.712719715	1.246504354	5.99E-22	2.55E-21
RHD	0.109485998	0.284789574	1.379149955	9.13E-10	1.73E-09
AC112694.2	0.112285837	0.295929546	1.398077783	8.38E-10	1.59E-09
ABCG1	5.000240292	12.12078588	1.277413911	5.52E-28	3.91E-27
IGHG2	53.540109	210.2353181	1.973313072	5.58E-06	8.34E-06
SNORA2A	0.230062549	1.081580297	2.23304272	2.94E-13	6.91E-13
ALAS1	28.54401931	13.93552561	-1.034421078	1.16E-33	1.87E-32
PDCL3P4	0.888970474	1.889042138	1.087447476	0.000403074	0.000532125
AC025287.3	0.256912254	0.615612468	1.260746749	5.68E-11	1.16E-10
LINC00982	13.26859927	0.316471523	-5.389796579	2.97E-40	4.71E-38
AC004865.2	0.122290623	0.491641994	2.007294365	3.81E-29	3.07E-28
AC135178.1	0.070889522	0.296774186	2.065721312	4.47E-12	9.76E-12
WSB1	8.174707139	17.08986734	1.063902246	5.34E-11	1.09E-10
TRDC	0.536539903	2.540657403	2.243444473	1.04E-31	1.17E-30
AC012170.3	0.157508627	0.353434671	1.166012723	8.27E-07	1.30E-06
NKAPL	0.965093628	0.456012362	-1.081595975	3.72E-25	2.05E-24
KSR1	2.708142889	10.44603089	1.947579108	2.59E-28	1.91E-27
ETNK2	20.55196175	6.529046657	-1.654331854	1.01E-21	4.24E-21
CHN2	2.039346169	0.69482304	-1.553389188	1.24E-36	4.35E-35
AC009159.3	1.631306993	3.993357697	1.291573998	1.95E-13	4.62E-13
MIR3941	0.10197068	0.785412746	2.94529662	1.87E-13	4.43E-13
AL049539.1	0.044403363	0.23688956	2.41547375	1.08E-07	1.81E-07
AC087276.1	0.195804961	0.833166315	2.089187193	2.06E-16	5.90E-16
SLC16A5	22.0304321	2.609375861	-3.077721118	3.94E-37	1.67E-35
TBX15	0.210030784	1.786312855	3.088312072	1.32E-32	1.76E-31
LINC01612	3.150561376	0.338202929	-3.219647854	2.61E-46	1.85E-43
SCN2A	0.997240903	0.117666152	-3.083242689	2.81E-37	1.26E-35
TRIM52	1.508779863	3.318789658	1.13727487	1.92E-23	9.20E-23
AMZ1	0.02425658	0.369250299	3.928151014	1.54E-33	2.42E-32
LHX1-DT	5.958081772	0.343124838	-4.118042455	6.11E-41	1.48E-38
YPEL4	0.09611978	0.604062004	2.651791398	1.90E-37	9.05E-36
HNF4A-AS1	2.914970828	0.144329283	-4.336045503	2.24E-20	8.48E-20
TCF21	10.25957853	0.917941382	-3.482425626	1.15E-39	1.38E-37
MIR6503	0.071031215	0.835919587	3.556839093	3.86E-19	1.33E-18
COL3A1	46.07860072	112.8471836	1.292201601	1.52E-12	3.42E-12
LINC02041	0.352964463	4.161978755	3.559674756	2.33E-29	1.93E-28
BCL2L10	2.550475111	0.771822101	-1.724425762	2.94E-30	2.71E-29
LOXL2	3.709400017	23.0460262	2.63526025	7.85E-33	1.08E-31
SLC18A3	0.002540888	1.374411798	9.079266102	9.38E-09	1.67E-08
MESP1	0.360661401	0.850691748	1.237991428	2.72E-18	8.84E-18
SLC22A4	2.625763261	5.739698215	1.128238035	3.96E-12	8.67E-12
TAC1	0.879882936	0.111907125	-2.975009697	7.19E-33	9.97E-32
NAT2	0.900692763	0.270082497	-1.737634922	3.30E-29	2.69E-28
APOC1P1	0.018793974	0.307021313	4.029996764	1.33E-33	2.11E-32
P4HB	77.15507111	201.3146886	1.383619552	1.51E-35	3.78E-34
PTOV1-AS2	0.642868247	2.771483232	2.10806328	1.53E-18	5.07E-18
LCK	2.048330546	4.955535296	1.274592359	8.55E-16	2.36E-15
AC104031.1	1.315596706	6.273398718	2.253529958	2.77E-18	9.00E-18
AL512791.1	0.66579777	1.915916058	1.52487841	3.98E-13	9.28E-13
FJX1	2.309058068	6.492966056	1.491573213	1.30E-27	8.85E-27

Table S1. The differentially expressed genes(DEGs) were screened out in TCGA

ID	conMean	treatMean	logFC	pValue	FDR
RPS20P14	0.884776075	2.288692258	1.371139209	6.25E-16	1.74E-15
LY6E-DT	0.24588886	1.076474862	2.130236351	1.38E-14	3.52E-14
AC023669.1	0.304971577	1.779069967	2.544376552	0.002890134	0.003589436
AL445673.1	0.174220522	0.739341759	2.08532683	2.97E-19	1.04E-18
FCGR3A	5.324274978	54.78629213	3.363157979	1.15E-37	5.91E-36
AC145207.8	0.097559027	0.244886773	1.327767573	2.10E-08	3.66E-08
MAFA-AS1	0.038338823	0.273866274	2.836593657	0.000268762	0.000359349
NR2E1	0.035360978	0.719879866	4.347526103	1.26E-05	1.83E-05
LDHD	27.38050605	5.644840111	-2.278144488	1.43E-31	1.57E-30
PEPD	84.34766029	23.9005466	-1.819304437	3.68E-21	1.48E-20
DIO1	26.5740953	1.008427804	-4.71984087	2.88E-21	1.17E-20
AC011899.3	0.041002606	0.317017231	2.950773755	7.39E-32	8.57E-31
RPL10	137.9935456	282.6638575	1.034486638	8.93E-33	1.22E-31
AC023509.3	0.146996536	0.752397199	2.355712318	1.70E-24	8.89E-24
AP000873.2	0.325321008	0.664423958	1.030240104	6.83E-12	1.47E-11
PLAGL1	1.836169739	4.418175673	1.266751353	2.69E-21	1.09E-20
RNU6-930P	0.083243149	0.33827271	2.022783354	2.92E-07	4.73E-07
ASPDH	12.4909683	1.884029867	-2.728991578	5.80E-10	1.11E-09
MCF2L-AS1	2.411878111	0.498237863	-2.275250433	1.59E-36	5.35E-35
LINC02274	4.878736272	13.7714662	1.497102763	6.17E-13	1.42E-12
PDP2	4.253299083	1.498487312	-1.505075438	1.43E-36	4.93E-35
USP46	4.265539917	1.750047916	-1.285333941	1.79E-39	1.93E-37
NKG7	2.946645176	33.89984614	3.524133473	1.55E-36	5.26E-35
TMEM82	6.406256995	2.962212287	-1.11280664	1.59E-05	2.31E-05
LINC01152	0.080504212	0.571609066	2.827892625	2.70E-25	1.51E-24
SNORA22B	0.0578702	0.234645921	2.019592843	2.82E-06	4.29E-06
LINC00216	0.123240616	0.294806175	1.258288942	0.003633931	0.004482378
AC080112.3	0.108886245	0.452154788	2.05399502	6.28E-24	3.12E-23
RRN3P2	0.173627052	0.530757282	1.61206052	1.28E-20	4.94E-20
KLF5	10.33221908	3.659709134	-1.497349244	1.07E-27	7.33E-27
TNNI2	0.132532006	0.703935369	2.409102169	5.02E-32	5.97E-31
AL161669.3	0.768424278	2.13739052	1.475875519	5.44E-07	8.67E-07
AC244197.3	0.107981339	0.42859881	1.988845829	6.17E-16	1.72E-15
INPP5D	1.973086658	6.462947246	1.711737895	7.42E-34	1.25E-32
RSPO4	0.056528742	0.341165889	2.593416921	3.51E-08	6.04E-08
AMFR	61.35922611	22.50680849	-1.446918793	4.49E-38	2.70E-36
AL031714.1	0.188507858	0.798453436	2.08258361	1.83E-24	9.55E-24
FSTL3	11.03094181	36.62563896	1.731297957	6.63E-20	2.43E-19
SHBG	0.962381983	0.206551441	-2.220108506	5.84E-27	3.75E-26
WNT9A	0.420453181	0.880848393	1.066948571	0.000556335	0.000727458
POLR2KP1	0.138386897	0.353586711	1.35335671	1.68E-06	2.60E-06
LILRA4	0.119213843	0.687380617	2.527557403	1.37E-25	7.89E-25
SUOX	15.40417578	7.440068766	-1.04993363	6.43E-38	3.64E-36
AC011383.1	0.038250756	0.406493653	3.409672654	1.20E-23	5.86E-23
OTOAP1	0.027380156	0.238963714	3.125589016	1.23E-38	9.07E-37
AC005899.7	0.089888873	0.373835774	2.056190189	4.24E-15	1.12E-14
PLAC9	3.733137958	9.161709919	1.29522806	6.99E-14	1.71E-13
AL359399.1	0.040462567	0.400934604	3.308707183	4.61E-19	1.59E-18
TRAV27	0.080017135	0.826501118	3.368635894	1.54E-17	4.78E-17
WNT6	0.223415152	0.502545601	1.169527481	0.00376003	0.004633206
SNORA5A	0.278544208	1.907501918	2.775706286	9.86E-13	2.24E-12
TNKS2-AS1	0.964664955	0.316663156	-1.607078936	5.05E-23	2.34E-22
KLC3	1.091151011	0.262438015	-2.055802159	5.76E-30	5.12E-29
KCNJ5	5.987828892	2.247310529	-1.413833509	0.001127625	0.001444831
AL157791.2	0.123332945	0.450409971	1.868680537	2.73E-15	7.30E-15

Table S1. The differentially expressed genes(DEGs) were screened out in TCGA

ID	conMean	treatMean	logFC	pValue	FDR
EME1	0.118293792	0.502080546	2.085544463	2.01E-34	3.79E-33
CBLN3	1.152428208	3.602153913	1.644182947	6.46E-28	4.55E-27
TVP23A	0.150986453	0.38927777	1.366380849	5.27E-21	2.09E-20
ACHE	0.622196647	1.519225254	1.287893266	0.003934003	0.004837729
PRTN3	0.040807546	0.25283187	2.631270478	1.24E-10	2.47E-10
AC018638.5	2.744867347	8.85276631	1.68939191	9.82E-24	4.81E-23
BMPER	0.146831769	0.405863646	1.466830976	9.00E-05	0.000123989
SYT1	1.069950865	0.224506289	-2.25271678	5.06E-34	8.78E-33
AC246787.1	1.136922089	3.321617631	1.546752614	2.35E-20	8.86E-20
AC118344.1	0.153617001	0.307350088	1.000545004	0.000155205	0.000210782
RN7SL128P	0.100056471	1.120135266	3.484786587	0.039525055	0.04493825
CORO1A	2.703767621	14.76266884	2.448910492	2.51E-33	3.79E-32
CDON	0.620468379	3.000772979	2.273904584	2.82E-29	2.31E-28
CFL1P5	0.187136035	0.387265893	1.049237058	1.05E-10	2.10E-10
LAMA5-AS1	0.102876616	0.812123599	2.98078422	4.16E-30	3.77E-29
C1QB	23.21477589	179.5414103	2.951201374	2.69E-35	6.31E-34
RPS20P4	0.042325676	0.271253861	2.680038667	2.38E-19	8.36E-19
TRAV26-2	0.057277341	0.388472446	2.761775838	3.69E-15	9.78E-15
AC060780.3	0.782240319	2.151177374	1.459442681	3.89E-10	7.51E-10
SERPINH1	17.48710889	50.73820463	1.53678068	3.99E-33	5.84E-32
ADGRE1	0.169917055	1.136049951	2.741123696	6.51E-33	9.12E-32
KIF14	0.084313681	0.312318041	1.88917725	3.36E-32	4.12E-31
P2RY13	1.04058252	3.482637241	1.742788828	4.61E-23	2.14E-22
PIK3R6	0.137901625	1.443178438	3.387538325	3.59E-39	3.28E-37
IGLV5-45	1.532065772	5.745637537	1.90698875	1.51E-05	2.20E-05
MORF4L2-AS	0.154217845	0.312001953	1.016585343	1.18E-05	1.72E-05
AC111186.1	0.083900485	0.23360152	1.477298598	2.31E-06	3.53E-06
CXCR6	0.308036263	2.060846095	2.742064663	1.85E-31	2.01E-30
AC104126.1	1.162663196	5.412947297	2.218981108	3.68E-07	5.92E-07
DGKZ	3.085506722	7.622944624	1.304840958	1.20E-34	2.39E-33
KISS1R	0.164233087	6.056842695	5.204749229	1.57E-33	2.47E-32
AC092653.1	0.136184582	0.387262806	1.507749571	7.96E-18	2.51E-17
ECSCR	4.376643861	10.48656868	1.260645791	1.59E-21	6.58E-21
TMEM155	0.017928872	0.655659625	5.19259043	5.20E-39	4.37E-37
SAPCD1-AS1	0.924251056	0.364679832	-1.341654371	1.09E-30	1.06E-29
PSMB9	5.288747181	24.81906221	2.230450688	1.07E-37	5.57E-36
AC006064.1	0.039086055	0.223274369	2.514091754	3.13E-19	1.09E-18
PCED1B-AS1	0.477538906	3.126144024	2.710694064	7.88E-36	2.14E-34
EMCN	30.37034835	14.68963719	-1.047864693	1.42E-19	5.09E-19
ANGPTL1	7.418793986	1.196912248	-2.631867294	3.95E-36	1.15E-34
SLC25A5-AS	1.906289936	0.465586068	-2.033647768	7.05E-37	2.72E-35
AC090589.3	0.409017473	1.357323426	1.730530151	4.99E-20	1.85E-19
ZC3HAV1L	2.044624458	4.776817133	1.224213765	4.77E-20	1.77E-19
AC090709.1	2.120159188	0.009765957	-7.7621955	4.17E-67	4.30E-63
AC138409.2	0.083065689	0.266853049	1.683720914	1.46E-17	4.53E-17
AL671883.2	1.153151556	2.704006295	1.229516376	2.47E-25	1.38E-24
AC124944.1	0.034606377	0.274790708	2.98922342	3.30E-24	1.68E-23
NPIPP1	0.558762067	2.295663486	2.03860519	4.42E-27	2.88E-26
RNU4ATAC1	0.018827601	0.380893536	4.338466685	1.73E-11	3.63E-11
AC010300.1	0.081179677	0.217180591	1.419704681	1.89E-07	3.10E-07
TIMM9P2	0.089014021	0.37777668	2.085429143	4.31E-07	6.91E-07
LDHB	428.8609028	146.1951428	-1.552614419	8.55E-39	6.62E-37
ORAI3	5.373079931	12.97450976	1.271858823	6.75E-34	1.14E-32
DEGS1	24.54561431	58.28496078	1.2476584	3.85E-31	3.98E-30
LIPA	19.79203097	44.87582433	1.181018777	1.51E-23	7.32E-23

Table S1. The differentially expressed genes(DEGs) were screened out in TCGA

ID	conMean	treatMean	logFC	pValue	FDR
TMCC1-AS1	0.216668486	0.745242156	1.782219952	1.48E-34	2.88E-33
SCUBE3	1.705700753	0.823103538	-1.051218739	9.07E-12	1.94E-11
AC114495.2	0.206934065	0.666247321	1.686886671	7.14E-13	1.64E-12
MIR3174	0.059164632	0.395588056	2.741191958	2.50E-07	4.06E-07
BCL7A	7.701682	3.780897663	-1.026444755	1.03E-30	1.01E-29
KCNV1	0.072285454	0.234676175	1.698894117	0.042799267	0.048537585
AC233280.2	0.080840612	0.271078857	1.745560451	8.27E-10	1.57E-09
RPL29P14	0.096331396	0.54347966	2.496148067	2.02E-10	3.98E-10
AC020917.4	0.249400595	1.092883681	2.131603048	2.57E-23	1.22E-22
MAGI2-AS3	5.701242833	1.921800221	-1.568818081	3.07E-26	1.86E-25
STEAP4	2.051316785	5.061920532	1.303134554	1.32E-10	2.63E-10
DDX12P	0.107296925	0.762302203	2.828754317	1.63E-32	2.15E-31
HLA-DQA2	7.124018247	39.21800228	2.460752932	6.66E-14	1.63E-13
LINC01150	0.228776632	1.151939418	2.332053242	1.24E-29	1.06E-28
AL031848.1	0.119280874	0.400093448	1.745974263	0.000576926	0.000753617
PLPPR4	0.213911511	0.428574051	1.002530387	0.003301897	0.004084568
AC009950.1	0.130414333	0.356219699	1.449664864	6.21E-27	3.99E-26
EPM2A	3.451112939	1.354741728	-1.349043849	3.09E-39	2.92E-37
TRBJ2-1	0.223418438	1.614231899	2.853027696	6.50E-22	2.77E-21
LINC01546	0.026377838	0.231946237	3.136392198	2.03E-31	2.19E-30
AP001830.1	0.702669326	1.554427449	1.145465455	7.62E-15	1.98E-14
INO80E	3.410822972	8.020481561	1.233568982	3.16E-36	9.44E-35
AL365356.3	0.230216358	0.677928585	1.558142954	7.62E-15	1.98E-14
CSF1	7.164363264	14.4531964	1.012478193	4.67E-22	2.01E-21
B2M	435.5191014	1104.585561	1.342697274	2.53E-33	3.82E-32
OIP5	0.289743232	0.861085187	1.571381008	3.47E-32	4.25E-31
GPRIN1	0.307153246	1.369553214	2.156674787	2.44E-31	2.60E-30
PYHIN1	0.172445116	1.405146101	3.026510971	5.03E-33	7.22E-32
LINC01736	0.061913901	0.420851079	2.764974551	7.35E-22	3.11E-21
LINC02195	0.027041274	0.297716564	3.460704474	4.15E-20	1.54E-19
ACKR2	0.926836596	0.190462646	-2.282806932	4.50E-35	1.00E-33
RN7SL208P	0.165518838	0.400838903	1.276027109	0.00088854	0.001147273
PRRG1	7.809160319	3.171903763	-1.299818431	2.04E-30	1.92E-29
SMG1P7	0.11726164	0.488570014	2.058834181	1.85E-22	8.17E-22
AL008707.1	0.123474995	0.415506353	1.750651621	2.14E-11	4.47E-11
ANO1	2.607134011	9.385392956	1.847952409	5.24E-25	2.86E-24
AC004908.3	0.274564273	0.732766579	1.416209796	3.19E-10	6.20E-10
TH2LCRR	0.05910671	0.250700743	2.08457245	9.11E-17	2.68E-16
AC073325.2	0.300996484	1.025238014	1.768140337	2.58E-11	5.36E-11
Z83843.1	0.54655055	1.609296565	1.558003373	1.02E-12	2.31E-12
TMEM86A	8.604149361	3.279287343	-1.391650248	6.71E-36	1.85E-34
LLGL2	13.42304108	5.685389038	-1.239380583	3.39E-35	7.72E-34
RAD51AP1	0.613055693	1.525074871	1.314790025	9.45E-29	7.30E-28
HLA-DMB	8.848303875	18.98307058	1.101240534	1.11E-16	3.24E-16
HLA-DRA	343.1340806	1147.741158	1.741952989	6.26E-28	4.41E-27
AP000797.3	0.009056442	0.252962234	4.803833867	9.08E-20	3.30E-19
AC010326.4	1.019886433	2.505785876	1.296854625	7.96E-19	2.69E-18
TRAV17	0.140349866	0.945829806	2.752552917	6.60E-21	2.60E-20
AC004585.1	0.089846222	0.922131088	3.359442114	2.00E-31	2.16E-30
LENG8	7.999182653	24.47658098	1.613477549	1.01E-16	2.97E-16
PCDHB6	0.535168546	1.872915526	1.807220601	1.89E-09	3.52E-09
BMPR1B-DT	1.455253724	0.054169588	-4.747643773	4.55E-52	7.80E-49
HNRNPA1P1[illegible]	0.085082094	0.256667066	1.592970745	2.91E-07	4.71E-07
LAMP3	0.755555239	1.67583864	1.149274105	9.88E-17	2.90E-16
IGLV1-41	0.180827749	1.282299729	2.826045532	0.000190525	0.000257182

Table S1. The differentially expressed genes(DEGs) were screened out in TCGA

ID	conMean	treatMean	logFC	pValue	FDR
MMP1	2.865098558	6.066956861	1.082388285	0.001389254	0.001768834
RF00404	0.167904951	0.493307882	1.554843566	1.13E-07	1.88E-07
SIGLEC1	0.553293826	3.309869657	2.580656675	3.08E-31	3.23E-30
MIR140	0.80691875	5.594120875	2.793416109	3.60E-19	1.25E-18
RASL10A	0.088502374	0.379535054	2.100445088	1.45E-25	8.33E-25
GBP1	6.002254292	21.85772452	1.864566872	8.03E-26	4.70E-25
RN7SL180P	0.077846777	0.392133036	2.332633972	0.004151887	0.005098053
MFNG	3.649244458	8.467584873	1.214352744	2.87E-28	2.10E-27
CD27	0.729040837	7.389494292	3.341404101	1.74E-29	1.46E-28
AC098614.4	0.604291846	2.253037619	1.898554023	7.35E-16	2.04E-15
EDIL3	10.83350471	23.76740427	1.13348431	2.00E-09	3.73E-09
BTBD16	0.132070097	1.538103079	3.541776433	1.99E-33	3.06E-32
LINC02157	0.149540575	0.369755269	1.306033722	2.60E-06	3.97E-06
LINC01702	2.552884988	0.345894001	-2.883726646	9.95E-19	3.34E-18
SH2B3	7.154943556	17.78482048	1.313634121	9.56E-28	6.59E-27
ATP1B1	828.4410625	321.4619011	-1.365751301	2.04E-34	3.85E-33
LINC01857	0.149180115	1.176315933	2.979148435	1.11E-26	7.00E-26
AL162430.2	0.068273418	0.307635553	2.171826349	9.24E-18	2.90E-17
LYPD6	1.175642684	0.178839566	-2.716711788	6.53E-36	1.81E-34
RPLP0	162.7625281	331.219276	1.025018042	5.91E-30	5.25E-29
SNHG3	1.434006997	2.874319313	1.003168278	1.78E-08	3.11E-08
ITPR1-DT	1.062180918	0.462842719	-1.198435586	2.39E-19	8.42E-19
FPR3	3.044746508	14.24515296	2.226077087	3.11E-28	2.26E-27
GIT2	2.02658	5.125554665	1.338661008	1.05E-29	9.07E-29
HLA-DQB2	2.422724475	17.12781795	2.821639118	7.47E-25	4.02E-24
GPAT3	31.91128811	5.607160768	-2.508724505	3.30E-35	7.56E-34
MS4A7	2.808058072	15.56794507	2.470933848	3.87E-34	6.91E-33
CDC45	0.204142215	0.98382515	2.268827387	2.08E-35	5.01E-34
UBA52P8	0.386495455	1.13251334	1.551004691	4.62E-11	9.45E-11
AC005332.7	0.130386864	0.394477518	1.597144549	6.28E-17	1.86E-16
AC012404.1	0.017146455	0.499150403	4.863492343	1.40E-08	2.46E-08
SNORA68B	0.071197547	0.35232786	2.307019126	4.23E-11	8.67E-11
PRELID2	0.421487728	1.795662	2.090953284	2.08E-35	5.01E-34
BMP6	14.4672776	3.283360442	-2.139548426	2.46E-19	8.63E-19
AKAP5	1.415611447	0.567259281	-1.319345121	2.16E-19	7.62E-19
AC068790.5	0.149478973	0.657821977	2.137754652	5.98E-22	2.55E-21
AC017100.1	1.253366303	0.622589019	-1.009456074	8.88E-22	3.74E-21
IL17RE	2.600160454	0.951753036	-1.449941482	3.77E-29	3.04E-28
AC007991.4	0.011748735	0.384423637	5.032119694	4.48E-29	3.59E-28
LINC02449	0.256072913	0.53877942	1.073140086	2.26E-08	3.92E-08
ITGAM	1.19621866	4.879095226	2.028132514	1.47E-30	1.41E-29
STEAP1B	0.063848706	0.297272238	2.219055453	2.01E-22	8.86E-22
CDC20	0.978308635	3.118393183	1.672441262	4.00E-27	2.61E-26
WDR27	0.547834221	1.42734045	1.381518196	8.97E-13	2.05E-12
AP001931.1	0.21892878	0.554793892	1.341490284	4.80E-09	8.70E-09
FERMT3	2.426102363	10.57932314	2.124535	1.48E-34	2.88E-33
AL583832.1	0.0779928	0.261079871	1.743078383	2.30E-11	4.80E-11
FGF9	9.149162983	1.075261352	-3.088952401	1.56E-36	5.28E-35
LINC01474	0.130085024	0.514798811	1.984553843	1.96E-16	5.64E-16
RNU6-678P	0.107503768	0.403428302	1.907925077	2.16E-08	3.76E-08
AC115284.2	0.628201907	0.192564751	-1.705884684	4.37E-30	3.94E-29
SLC13A3	72.00659971	2.805283593	-4.681910613	9.40E-21	3.66E-20
CRB2	3.43696713	0.492738815	-2.802241027	8.96E-10	1.70E-09
AP005212.2	0.059479456	0.265345937	2.157411114	8.92E-11	1.79E-10
CEACAM21	0.333127012	1.227125435	1.88113848	4.50E-28	3.23E-27

Table S1. The differentially expressed genes(DEGs) were screened out in TCGA

ID	conMean	treatMean	logFC	pValue	FDR
C2orf92	0.109701749	0.474382284	2.11246361	8.69E-24	4.27E-23
BNIP3P27	0.080116751	0.393242457	2.29524327	2.71E-09	4.99E-09
AL354892.3	0.166186519	0.372114016	1.162941373	3.18E-09	5.82E-09
AC007998.3	0.525834906	1.234404285	1.231133156	1.92E-06	2.95E-06
AP000769.1	0.885670976	2.821652489	1.671697571	6.02E-28	4.25E-27
EAF2	5.138787586	2.525312864	-1.024965883	1.69E-08	2.96E-08
AC000123.1	0.786116856	1.860508384	1.242881203	1.99E-25	1.12E-24
MAMDC2	2.72457525	0.693518452	-1.974025165	4.98E-30	4.46E-29
AC022400.8	0.233741993	0.798530661	1.772430856	1.48E-29	1.25E-28
IL20RB	0.672176993	10.24814299	3.930377536	6.27E-37	2.47E-35
AL138767.3	0.643366061	0.25212342	-1.351509693	8.79E-21	3.43E-20
AC144548.1	0.08271539	0.250557785	1.598915674	3.59E-13	8.39E-13
AC010463.2	0.027957635	0.460038065	4.040439016	6.05E-21	2.39E-20
SP140	0.248055484	0.866994532	1.805360042	4.89E-21	1.95E-20
ASPM	0.137410003	0.653812804	2.250390605	6.79E-33	9.49E-32
CST6	1.902074173	0.670825225	-1.503564663	2.22E-17	6.80E-17
MFSD13B	0.139604007	0.412493468	1.563030921	5.64E-10	1.08E-09
ARHGEF39	0.150836148	1.005209646	2.736442298	1.10E-40	2.35E-38
AC023137.1	0.120572822	0.309129108	1.358304754	6.08E-06	9.07E-06
AC126603.1	0.546273005	1.885903042	1.787561468	5.83E-10	1.11E-09
NUDT1	1.930520633	4.451537321	1.205313677	6.51E-33	9.12E-32
LINC01510	8.574224659	2.084540285	-2.040276965	3.14E-09	5.75E-09
AC107983.1	1.103371115	2.849277135	1.368677834	1.96E-16	5.64E-16
RAB37	0.712586286	2.73703049	1.941474882	1.38E-32	1.83E-31
HPN-AS1	0.212642214	0.700171954	1.719281241	4.27E-08	7.29E-08
CD84	0.537409615	3.845515907	2.839083121	1.66E-32	2.18E-31
ADPRHL1	0.690697993	0.328923464	-1.070303103	3.59E-27	2.36E-26
AL158827.2	0.1112767	0.250458544	1.170420282	5.24E-09	9.47E-09
CAND2	1.834939486	0.525710641	-1.803391644	1.01E-36	3.69E-35
AL138721.1	0.137966413	0.632580631	2.196932287	6.66E-10	1.27E-09
GALNT3	8.438114076	1.431968301	-2.558921035	4.02E-36	1.17E-34
ATP5PDP4	0.343024114	0.714617179	1.058860597	2.20E-06	3.37E-06
AL513365.2	0.13790796	0.404823175	1.553586154	1.08E-14	2.77E-14
PDCD1	0.257014739	2.666049357	3.374780489	3.86E-26	2.32E-25
AC009154.1	0.04623392	0.332456688	2.846142807	6.95E-09	1.25E-08
AC010525.1	0.065975193	0.276679929	2.068222414	1.26E-08	2.22E-08
HS6ST2	10.61975078	0.1425965	-6.218667533	5.22E-41	1.34E-38
CYP4F2	12.57277723	0.40139887	-4.969122998	4.28E-17	1.29E-16
LY6G5B	0.177001851	0.967895761	2.451087234	8.46E-22	3.57E-21
PTP4A3	5.701081667	20.8782656	1.872694296	2.77E-34	5.06E-33
BX640514.2	0.309585969	1.326625909	2.099349613	3.01E-28	2.20E-27
SEMA6A-AS1	0.144724342	0.740373344	2.354945358	1.72E-27	1.16E-26
AC244517.6	0.12025112	0.250257365	1.0573622	0.000995126	0.001279999
AC012066.1	0.033962885	0.232640698	2.776072594	1.55E-19	5.52E-19
ZDHHC3	13.64500906	6.803601453	-1.004002815	3.11E-33	4.62E-32
IGBP1-AS1	0.096276568	0.377840418	1.97252042	7.08E-17	2.09E-16
AC010761.3	0.182288929	0.653050153	1.840966849	7.63E-14	1.86E-13
CD3E	1.760655164	11.22003117	2.671892403	8.19E-29	6.36E-28
FBXL16	4.41547229	13.12623379	1.571813369	7.45E-15	1.93E-14
BASP1P1	0.269657855	2.569696038	3.252395754	0.000140368	0.000191195
AC092142.1	0.419777938	0.19470789	-1.108314998	1.20E-20	4.64E-20
AC034236.1	5.562373296	12.58526132	1.177962699	2.33E-15	6.25E-15
IFI16	7.022676042	21.35546167	1.604512297	2.81E-32	3.49E-31
AC145285.1	0.110376674	0.250343765	1.1814752	1.98E-09	3.68E-09
AP000763.3	0.034723134	0.245492232	2.821708286	7.35E-18	2.32E-17

Table S1. The differentially expressed genes(DEGs) were screened out in TCGA

ID	conMean	treatMean	logFC	pValue	FDR
CUZD1	0.168090419	0.419018925	1.317777907	3.68E-17	1.11E-16
AL109615.1	0.054547336	7.016056543	7.007007833	5.72E-36	1.62E-34
MLIP	0.069581502	0.312347902	2.166378109	1.86E-27	1.25E-26
CLEC18C	0.668626969	2.335237522	1.804295846	7.79E-09	1.40E-08
AC018816.2	0.297625349	0.795238856	1.417890842	0.00033641	0.000446494
COL6A1	27.51836869	59.21488368	1.105564895	5.42E-17	1.62E-16
AC103691.1	0.388933929	1.332946557	1.777021939	8.26E-16	2.28E-15
RPL7P50	0.027957464	0.247761711	3.147647836	3.29E-29	2.68E-28
SYPL2	5.765026083	2.331310243	-1.306186129	2.19E-31	2.34E-30
MIR1291	0.088031036	0.245066687	1.477090231	5.70E-07	9.08E-07
AC253576.2	0.224225358	0.510799307	1.187807122	3.60E-05	5.09E-05
HIST1H2BK	15.60831514	33.25068243	1.091069139	4.03E-11	8.27E-11
PGBD5	0.704718219	2.431164767	1.786529254	5.22E-15	1.37E-14
CHAC1	4.404742008	1.083240905	-2.023703397	8.91E-28	6.17E-27
KCNAB1	0.861697503	2.429327894	1.49530382	2.15E-20	8.16E-20
ANKAR	0.335285046	0.723529482	1.109663666	2.17E-24	1.12E-23
CSRNP3	1.294016257	0.431942902	-1.582943219	1.60E-34	3.09E-33
ZNF826P	3.213913482	9.879239535	1.620068901	1.32E-16	3.86E-16
SPAG4	1.464010193	21.43590853	3.872032062	1.83E-40	3.40E-38
ZKSCAN2-D	0.240281043	0.857670623	1.835700873	1.06E-16	3.09E-16
AP001094.2	0.090901885	0.643657052	2.823910088	2.00E-32	2.58E-31
FAM57A	3.705001542	11.03845636	1.574992389	3.59E-29	2.90E-28
LINC01114	0.103882963	0.423587035	2.027699371	0.001621037	0.002053416
RPL31P61	0.083182249	0.384420382	2.208337225	6.21E-14	1.52E-13
LBX2-AS1	3.087290622	7.877656231	1.351425164	3.70E-28	2.67E-27
AC010271.1	0.131659221	0.297014407	1.173724351	4.16E-06	6.27E-06
SRGAP2D	0.303261356	0.676881759	1.158342168	2.66E-11	5.53E-11
SNORA2C	0.125369134	2.253001896	4.16759442	3.83E-10	7.40E-10
LINC01402	0.190616266	0.514413559	1.432257436	1.06E-08	1.87E-08
ALPK2	2.429858385	9.829275626	2.016212866	6.94E-25	3.75E-24
Z97056.1	0.0709241	0.689246686	3.280672579	1.78E-20	6.79E-20
ALS2CL	4.751228111	1.461386593	-1.700962596	2.84E-27	1.89E-26
AC123768.2	0.096441687	0.264401358	1.455000793	7.14E-14	1.74E-13
BNIPL	0.829635176	0.283248702	-1.55040772	9.41E-23	4.27E-22
LILRB4	0.450543951	3.973962832	3.140838624	1.48E-36	5.08E-35
PNRC2P1	2.187064062	1.077260869	-1.021627825	6.80E-07	1.08E-06
EBF2	0.318784504	2.134153921	2.743010823	1.95E-31	2.10E-30
IGLV7-43	1.955734998	8.688353114	2.15137184	1.33E-05	1.94E-05
TSSC2	0.187553409	0.509938103	1.443020655	1.54E-17	4.75E-17
ENO2	5.282017392	47.76765384	3.176873062	8.85E-37	3.34E-35
GIMAP7	13.68628575	32.19384067	1.234053724	4.41E-20	1.64E-19
CHDH	16.15601893	6.838296663	-1.240362824	8.88E-22	3.74E-21
C16orf89	16.52573969	2.027019195	-3.027283193	1.01E-33	1.65E-32
AL022238.3	0.099236905	0.379808316	1.936322852	3.04E-14	7.60E-14
DPY19L2P2	0.567186582	1.911702269	1.752962546	6.54E-21	2.57E-20
CD70	1.815810682	36.13037075	4.31452636	9.13E-36	2.45E-34
FGFR1OP	0.808658618	2.164913137	1.42070645	2.74E-26	1.67E-25
CDCA7L	1.556091649	5.368508037	1.786594171	1.38E-29	1.17E-28
RNU6-807P	0.15691013	0.533173607	1.764666873	3.24E-08	5.57E-08
AC021851.1	0.139902516	0.314945156	1.170678717	5.95E-15	1.55E-14
RASSF8	11.52839654	4.091519974	-1.494483066	5.67E-40	7.89E-38
AC007292.2	0.124418503	0.317284974	1.350578152	3.92E-24	1.98E-23
MN1	0.757331717	1.531350656	1.015807421	3.33E-07	5.37E-07
NXNL2	1.215935082	2.751500472	1.17815237	4.06E-12	8.88E-12
AC137055.1	0.05719823	0.24151816	2.078089273	2.76E-07	4.47E-07

Table S1. The differentially expressed genes(DEGs) were screened out in TCGA

ID	conMean	treatMean	logFC	pValue	FDR
PPP1R36	1.478023525	0.259821455	-2.508076759	3.42E-30	3.13E-29
NHSL1	5.619309792	2.410254312	-1.221207561	2.58E-33	3.88E-32
STARD4-AS1	0.414538776	1.478126912	1.83419118	3.24E-24	1.65E-23
RAB24	2.272892013	7.24519473	1.672495324	5.39E-33	7.70E-32
PLIN2	26.37278014	208.070087	2.979948003	2.63E-31	2.78E-30
TRAJ18	0.023256135	0.364358804	3.96967661	1.30E-11	2.76E-11
TOMM20P2	0.141794125	0.675107046	2.251318518	4.55E-23	2.11E-22
AL512652.1	0.070394588	0.255079651	1.85741139	8.37E-14	2.03E-13
LINC00173	0.193862867	1.96901178	3.344363347	7.19E-27	4.59E-26
HS6ST1	33.10929818	7.272424258	-2.186728159	2.30E-36	7.23E-35
FAM177B	0.113337707	0.417375716	1.88071874	2.49E-25	1.39E-24
AP003392.1	0.517678371	1.759650235	1.765160746	2.38E-14	5.98E-14
ACAP1	0.646466929	2.365560149	1.871533371	1.08E-32	1.46E-31
AL117209.1	0.194589444	0.608899265	1.64577012	3.34E-11	6.89E-11
NLRP3P1	0.004716156	0.603159632	6.998784646	3.07E-34	5.58E-33
AC012618.2	0.114513475	0.2729143	1.252930623	3.91E-07	6.28E-07
HECW1	1.49880001	0.121962045	-3.619303741	1.40E-12	3.15E-12
SCARB1	2.450124358	34.56733916	3.818482674	1.22E-39	1.44E-37
CASS4	0.50878411	1.223410827	1.26578343	4.95E-17	1.48E-16
PTHLH	0.312913128	14.43224251	5.527389487	6.30E-36	1.75E-34
CTNNAL1	20.82053774	7.819279	-1.412899839	9.64E-38	5.14E-36
GABRR2	0.138140918	0.366496312	1.40765796	1.09E-09	2.05E-09
RNU6-1053P	0.406908222	1.325296027	1.703539308	6.40E-12	1.38E-11
GAPLINC	0.085080507	0.369521722	2.118758632	7.04E-28	4.93E-27
UBA52P3	0.132378857	0.432655967	1.708547585	1.25E-09	2.35E-09
THEMIS2	2.093788319	8.922891776	2.091395748	2.43E-33	3.67E-32
SH3RF3	1.379779321	3.12676427	1.180232913	1.23E-21	5.14E-21
IGHV3-23	15.06798929	75.58112064	2.326538996	9.28E-06	1.37E-05
RNU1-67P	0.046360858	0.885557967	4.255607573	3.00E-16	8.53E-16
RNU6-564P	0.051815713	0.293268255	2.500759361	4.97E-11	1.01E-10
PFN1P6	0.17861499	1.02713464	2.523700244	5.89E-25	3.19E-24
RNU6ATAC2	0.071486968	0.273367648	1.935090347	5.64E-05	7.88E-05
GYPC	9.83688675	19.97347257	1.021811482	1.74E-26	1.07E-25
DEFB1	841.20163	207.3787319	-2.020183702	1.15E-34	2.29E-33
ADORA2A	0.104148646	0.286872569	1.461765938	2.02E-22	8.92E-22
RPL37P23	0.250534523	0.581062338	1.213683531	1.39E-15	3.80E-15
AL357060.1	0.132084163	0.704163061	2.414452053	1.07E-39	1.33E-37
IGLC2	44.5174044	228.6131896	2.360467257	4.17E-09	7.58E-09
IGKV1-16	3.66623047	19.33284086	2.398684264	4.61E-06	6.93E-06
PRR35	5.78884519	0.039927477	-7.179749839	7.00E-47	5.76E-44
NR5A2	0.501486453	1.219608861	1.282135903	8.90E-20	3.23E-19
FER1L6	1.134488088	0.164301779	-2.787621455	1.37E-39	1.61E-37
MYOZ1	2.525932714	0.942535402	-1.422197496	6.60E-31	6.62E-30
MIR374B	0.123784107	0.50486769	2.028079257	2.88E-06	4.38E-06
AC027644.2	0.133021748	0.355909853	1.419849733	9.92E-05	0.000136463
MIR6512	0.196620936	0.427774178	1.121432451	0.000179312	0.00024257
AC093616.1	0.170339962	0.701585016	2.042201004	6.50E-29	5.12E-28
MYH10	31.39399342	9.501357385	-1.724283016	3.65E-36	1.07E-34
IBSP	0.021935604	0.827301317	5.237066521	1.23E-21	5.10E-21
RPS8	174.3955486	350.7467918	1.008066692	1.59E-29	1.34E-28
LPXN	3.950287222	9.938450486	1.331063383	8.55E-33	1.17E-31
RDH5	0.857347276	2.117810726	1.304622053	3.38E-17	1.02E-16
AC009533.1	0.258111078	1.297927696	2.330146049	3.49E-23	1.63E-22
ATP12A	4.346723687	0.029698188	-7.193409661	1.79E-43	1.05E-40
AC124016.2	0.558243788	1.207655684	1.113241988	3.08E-10	5.98E-10

Table S1. The differentially expressed genes(DEGs) were screened out in TCGA

ID	conMean	treatMean	logFC	pValue	FDR
CHRNA1	0.009914517	0.424061477	5.418587067	8.61E-31	8.50E-30
AL033519.2	0.193275437	0.396249857	1.035752118	4.56E-12	9.94E-12
RN7SL558P	0.047308392	0.364864947	2.947194538	6.92E-14	1.69E-13
MIR1285-1	0.279502017	1.018080886	1.864921589	2.48E-12	5.50E-12
AC025171.2	0.447331908	1.245729831	1.477573641	4.99E-26	2.97E-25
AQP4	1.710380198	0.702657326	-1.283423867	1.09E-13	2.64E-13
AL157871.2	0.09581481	0.533825767	2.478048377	5.99E-16	1.67E-15
AC110015.1	0.044357726	0.27691196	2.642170049	5.82E-23	2.68E-22
TBX3	6.350419642	1.790014118	-1.826880964	3.36E-33	4.97E-32
TRIM60P18	0.850068285	1.965041381	1.208909053	8.96E-23	4.07E-22
AC114760.2	0.0396378	0.24355636	2.619306869	9.84E-15	2.53E-14
SLC45A2	0.05818203	0.260007386	2.159907077	3.97E-11	8.16E-11
RNU6ATAC2	0.237891463	0.602951861	1.341739327	3.99E-07	6.42E-07
AC007619.1	0.200898358	0.447548944	1.155579693	3.62E-07	5.83E-07
AC079322.1	0.100086147	0.429485207	2.101366135	2.01E-21	8.25E-21
DEPP1	27.14535215	129.6864656	2.256250819	7.03E-33	9.77E-32
LINC02027	3.339307601	1.436699321	-1.216790834	4.03E-07	6.48E-07
CAPN15	2.82624294	7.042157595	1.31713203	1.92E-34	3.65E-33
SCARNA5	0.200124987	52.75781533	8.042339704	1.61E-07	2.66E-07
AC108463.2	0.294182831	1.337661305	2.184927916	1.13E-32	1.53E-31
ARHGDIB	40.47512806	127.8264013	1.659078291	5.71E-35	1.24E-33
CYTOR	1.49133694	5.177977986	1.795782587	3.00E-30	2.76E-29
IL34	1.862572189	4.495850981	1.271297875	9.75E-13	2.22E-12
AC133644.2	0.168096263	1.568148095	3.221702259	5.92E-15	1.55E-14
FLT4	4.349304556	9.914092772	1.188696023	3.22E-16	9.14E-16
RASD2	0.602515363	4.563982581	2.921223353	2.57E-36	7.96E-35
FO393401.1	0.074205779	0.410289448	2.467038598	2.40E-33	3.64E-32
TAS2R20	0.0960179	0.400112361	2.059029916	1.90E-23	9.14E-23
AC129492.1	0.275619812	0.698306046	1.341179872	1.81E-09	3.37E-09
SNORA74B	0.124356876	10.28181924	6.369465468	7.90E-06	1.17E-05
GALNT14	23.56257244	51.17547492	1.118955532	1.43E-25	8.22E-25
RNA5SP108	0.09527334	0.387099108	2.022558507	5.99E-13	1.38E-12
BIRC6-AS2	0.040059487	0.222211266	2.471716126	1.73E-09	3.23E-09
LINC00885	0.746540432	0.157909883	-2.241118931	1.68E-36	5.56E-35
HNRNPA1P5	0.083392509	0.256022707	1.618282072	2.73E-15	7.30E-15
AC015727.1	0.11786545	0.403973364	1.777119293	1.10E-12	2.48E-12
WFDC2	115.4656713	50.32470087	-1.198125395	1.26E-22	5.63E-22
GATA5	0.860232943	0.201383656	-2.094780781	4.35E-22	1.88E-21
AC093496.1	3.372014013	0.366625623	-3.201231009	1.12E-25	6.50E-25
FLRT1	2.268199999	0.126036994	-4.169628703	1.09E-37	5.63E-36
WDR54	5.021784181	11.07302974	1.140778085	1.06E-28	8.11E-28
ELMO1	1.445801311	5.114068108	1.82260207	1.01E-33	1.65E-32
IGHV6-1	0.342533854	2.469780651	2.850064429	3.39E-06	5.14E-06
SLC4A1	48.75202	4.206311193	-3.534834504	5.81E-38	3.37E-36
ITGA6-AS1	0.155973861	1.231354903	2.980870456	1.15E-35	2.96E-34
GLOD5	3.269687926	0.62183194	-2.394556319	9.28E-34	1.54E-32
PDE8B	3.406938569	1.637623751	-1.056872003	4.57E-21	1.82E-20
AC005696.3	0.085639204	0.268126081	1.646568273	2.50E-09	4.62E-09
AC112484.3	0.162960469	0.368869838	1.178589784	0.001446536	0.001840282
AC010976.1	0.072017193	0.241902787	1.748014117	2.63E-15	7.04E-15
SOWAHA	5.387074083	0.615433943	-3.129825984	1.31E-40	2.58E-38
AGXT2	32.33866855	14.88963982	-1.118951426	0.005501978	0.006694331
SKA1	0.134348722	0.577037356	2.102682122	5.82E-34	1.00E-32
MUC13	7.263092983	1.336993377	-2.44159173	3.06E-09	5.62E-09
LINC02532	9.270650042	19.12120944	1.044431372	1.07E-15	2.95E-15

Table S1. The differentially expressed genes(DEGs) were screened out in TCGA

ID	conMean	treatMean	logFC	pValue	FDR
AL022718.1	0.112709721	0.264979666	1.233269707	0.007274962	0.008774278
MTMR9LP	1.2376124	3.398717789	1.457431017	3.44E-08	5.91E-08
IGLL5	2.897181959	19.21974518	2.729867007	9.41E-08	1.57E-07
GABRA2	2.319220942	0.083128469	-4.802153821	2.33E-40	3.95E-38
NTM	0.671986411	2.240970853	1.737619921	1.39E-09	2.61E-09
AC007406.4	0.163163813	0.469915929	1.526081549	0.001455587	0.001851339
JCHAIN	33.24663349	82.48492357	1.310922188	1.98E-05	2.86E-05
IGKV2-28	0.094094636	1.795693071	4.254284486	5.46E-05	7.63E-05
LCN1P1	0.070331777	0.21768309	1.629980763	9.62E-11	1.93E-10
BCAN	0.156655338	0.616614545	1.976774987	5.38E-26	3.20E-25
AC004967.1	2.409503804	4.835612784	1.004962645	1.42E-20	5.45E-20
TWSG1	30.57066375	13.42510519	-1.187214484	2.10E-33	3.21E-32
RTN4R	0.46910271	0.974716441	1.055078746	4.14E-12	9.06E-12
AC099684.2	1.919925497	0.485309291	-1.984073941	1.38E-15	3.76E-15
C16orf45	7.648834736	3.668634243	-1.0599969	4.74E-33	6.84E-32
LY86	1.905317428	10.66029259	2.484143758	1.00E-34	2.04E-33
SNORA23	0.133256569	8.742214109	6.035720153	0.010499647	0.012499522
AC027644.1	0.615532269	1.597440549	1.375855843	1.97E-11	4.14E-11
ERBB2	35.63789492	12.91565293	-1.464291542	2.79E-37	1.26E-35
SLAMF7	0.648377985	4.704244136	2.859055922	7.07E-30	6.20E-29
AL031123.1	10.58834317	0.906174057	-3.54654486	1.09E-39	1.34E-37
SLC43A2	18.68026072	8.808725565	-1.084509379	9.68E-29	7.47E-28
EVI2B	3.330214731	13.03070022	1.968227502	1.87E-28	1.40E-27
PTCRA	0.030712418	0.231175395	2.912093842	4.22E-33	6.15E-32
LINC00342	0.591508676	3.152218238	2.413896187	6.71E-27	4.29E-26
FAM110A	2.005331454	4.387516887	1.129563964	1.37E-28	1.04E-27
LINC01770	0.433212715	1.039776138	1.263125461	5.51E-12	1.20E-11
ZNF197-AS1	0.674898457	0.252895598	-1.416128533	2.08E-29	1.72E-28
AC005674.2	0.268052131	0.76060599	1.504635696	2.70E-11	5.61E-11
RPL41P2	0.938775388	2.107045371	1.166369456	7.04E-12	1.52E-11
CPAMD8	5.930039749	0.854294691	-2.795236053	4.62E-39	3.95E-37
ASIP	0.185070003	0.561546097	1.601333385	3.50E-05	4.96E-05
RGS10	6.044428903	18.56216488	1.61868704	4.70E-31	4.80E-30
PTPN13	16.96199097	4.817310848	-1.816005596	6.83E-39	5.41E-37
RNU6-196P	0.074188073	0.292009846	1.976757843	6.29E-07	9.98E-07
AC004024.1	0.709044819	0.314317142	-1.17365587	1.90E-26	1.17E-25
AL645939.3	0.314076355	0.920294123	1.550979679	2.13E-15	5.74E-15
IBA57-DT	0.121534298	0.337348916	1.472878015	1.02E-11	2.18E-11
KIF2C	0.530609532	1.461630604	1.461856249	3.37E-29	2.74E-28
CRYZL2P-SE	0.100537364	0.337087303	1.745390514	9.08E-20	3.30E-19
AC087163.3	0.077597036	0.280426138	1.853547371	6.20E-17	1.84E-16
AC002511.2	0.210363614	0.688537418	1.710649872	0.015746071	0.018469696
RPL3P4	12.01027469	30.57338526	1.348007156	1.56E-12	3.49E-12
NELL2	0.258981066	0.693991913	1.422072226	1.59E-09	2.97E-09
APOBEC3D	0.729074669	2.993356202	2.037625483	6.35E-35	1.36E-33
GPR65	0.624472513	2.379821988	1.930143688	2.25E-28	1.66E-27
OGDHL	64.02705629	16.169257	-1.985428296	1.51E-30	1.44E-29
LIN52	5.398752042	2.588698076	-1.060399246	1.31E-33	2.09E-32
AC091959.1	0.028744511	0.25847031	3.168640195	4.12E-11	8.45E-11
SNORD117	0.180261794	0.927368069	2.363048396	1.05E-13	2.52E-13
RNU6-5P	0.119976649	0.377392757	1.653313096	1.45E-06	2.25E-06
MRAP2	2.947445291	0.286314374	-3.363793027	5.77E-39	4.77E-37
AVPR2	7.926016222	0.725834401	-3.44888358	5.62E-39	4.66E-37
AC068987.3	0.112252142	0.266406022	1.246883718	3.92E-06	5.92E-06
IQCA1-AS1	0.151347304	0.339075011	1.163741489	8.94E-07	1.40E-06

Table S1. The differentially expressed genes(DEGs) were screened out in TCGA

ID	conMean	treatMean	logFC	pValue	FDR
IGLV3-10	4.086395064	36.00517493	3.139303683	1.93E-05	2.78E-05
RPS19P3	0.207425185	0.728064602	1.811475395	8.05E-25	4.32E-24
LHFPL2	1.99220945	7.892943931	1.986194168	6.03E-33	8.49E-32
AC110799.1	0.027079654	0.302324918	3.480818679	4.61E-26	2.75E-25
IL12RB2	0.18194062	1.165041818	2.678842156	0.004842922	0.005916572
AC093484.4	0.161109073	0.557765226	1.791620251	6.04E-13	1.39E-12
AP003555.3	0.088634251	0.232956736	1.394125831	3.49E-08	6.00E-08
LHFPL3	0.74458489	0.289936234	-1.360700698	3.95E-25	2.17E-24
CCNO	1.368027039	0.564324878	-1.27749889	2.07E-18	6.79E-18
AP002360.3	0.333514189	1.383087376	2.052072255	5.50E-18	1.75E-17
PURPL	0.064544492	0.483354356	2.904715345	6.28E-06	9.36E-06
TLR6	0.222823465	0.765516281	1.780531891	1.17E-26	7.32E-26
SYTL5	0.89612689	0.287677011	-1.639253093	6.08E-31	6.13E-30
IGHV1-3	0.223389594	1.822157704	3.028013936	1.16E-05	1.69E-05
TRBV7-6	0.097191294	0.779281389	3.003245367	4.79E-17	1.44E-16
NOS2	0.59413861	1.204800056	1.019922293	0.002423956	0.003028364
AL359092.1	0.047416596	0.27266807	2.523681764	6.15E-05	8.58E-05
PROM2	28.94209932	2.420898455	-3.5795551	1.99E-39	2.10E-37
TRBV13	0.06753949	0.642841843	3.250660639	6.13E-15	1.60E-14
ADAMTS8	0.766713247	0.327941721	-1.225247655	1.02E-31	1.15E-30
ANKRD13B	0.53353215	1.438103468	1.430520364	2.50E-22	1.10E-21
MIR3653	0.434865843	1.142599617	1.393677651	2.10E-13	4.99E-13
AC073957.3	0.336633615	0.794259792	1.238431727	2.20E-07	3.60E-07
INF2	6.799292792	17.0901412	1.329707715	3.03E-28	2.21E-27
HIST1H3B	0.020438769	0.540657458	4.725334617	7.74E-08	1.30E-07
AP003484.1	0.016846087	0.290150652	4.106316712	1.37E-17	4.25E-17
SNAI3	0.424890299	1.060103251	1.319042477	5.28E-27	3.41E-26
RAP2C-AS1	1.250433854	0.554320113	-1.173637483	1.23E-33	1.96E-32
CDHR5	21.29616201	46.34265821	1.121747348	2.67E-08	4.62E-08
AC245100.2	0.111980422	0.287562343	1.360628238	4.50E-08	7.67E-08
LHFPL3-AS2	8.787694431	3.668862503	-1.260151867	1.08E-23	5.27E-23
FMO4	13.0881996	5.233469072	-1.322427177	7.17E-20	2.62E-19
MIR6509	0.153123243	0.540397172	1.819326833	1.66E-07	2.74E-07
IGHV4-59	5.931061566	31.93591725	2.428817635	2.74E-05	3.91E-05
CEBPB	9.480237	24.14280419	1.348598224	2.01E-18	6.60E-18
SNORA9B	0.102304151	0.418342557	2.031820087	2.96E-12	6.54E-12
SLC6A3	0.593973177	42.62205684	6.165058623	1.96E-31	2.12E-30
DNM3	1.451464525	0.642009932	-1.17684179	1.74E-25	9.90E-25
AC135068.2	0.076430972	0.308627933	2.013639357	4.62E-05	6.50E-05
DPYS	36.99005904	14.36359881	-1.36472034	0.002107752	0.002644709
KLK7	4.020145442	0.074967631	-5.744836073	4.42E-24	2.22E-23
ETNPPL	1.252024841	0.215209912	-2.540446754	9.20E-21	3.58E-20
MIR659	0.977247269	0.487216918	-1.004159417	1.08E-13	2.61E-13
MAP9	4.146226181	1.723186217	-1.266720204	4.91E-33	7.05E-32
MTOR	9.448338153	4.229550758	-1.159556164	8.30E-37	3.16E-35
RGS14	4.573026472	11.91387454	1.381421496	6.75E-26	3.97E-25
CLEC12A	0.197688489	1.434497766	2.859244951	1.03E-31	1.16E-30
MIR499A	0.045923037	0.403001614	3.133495651	1.28E-14	3.27E-14
FCER1G	9.167727556	53.05744517	2.532919132	2.30E-36	7.23E-35
AL355309.1	0.24496819	0.653729057	1.416098399	0.001225949	0.001567594
RPL12P10	0.056432751	0.444107923	2.976305725	1.34E-17	4.15E-17
SORD2P	1.637897763	0.511294105	-1.679620008	3.43E-25	1.90E-24
AC010719.1	0.158760966	0.634456567	1.998665162	2.70E-12	5.97E-12
RPRM	1.424327524	0.141879374	-3.327544156	4.57E-37	1.90E-35
AC103740.1	0.107899959	0.505373526	2.227655769	1.13E-27	7.73E-27

Table S1. The differentially expressed genes(DEGs) were screened out in TCGA

ID	conMean	treatMean	logFC	pValue	FDR
AC015712.2	0.232121878	0.480619563	1.050012864	6.73E-15	1.75E-14
FAM153A	0.061901243	0.614541707	3.311470636	4.01E-09	7.30E-09
SYN1	0.339540918	0.751360369	1.145919577	0.000125471	0.000171584
MAPK4	2.663208699	0.163049901	-4.029780013	2.19E-38	1.46E-36
IGKV3-15	5.805712656	39.27893908	2.758210888	1.23E-06	1.92E-06
ENPP2	23.147985	70.05449092	1.597590925	4.69E-11	9.59E-11
FRMD7	1.402549824	0.10776412	-3.702103197	1.06E-43	6.40E-41
RPL21P136	0.027152176	0.258807825	3.252741518	5.50E-15	1.44E-14
AC005532.2	0.05299806	0.448296202	3.080440823	1.83E-17	5.64E-17
PDHB	21.86855007	9.711039788	-1.171159887	3.87E-37	1.65E-35
CBR1	56.00014792	27.86296553	-1.007081822	1.83E-29	1.53E-28
AL663070.1	0.147402869	0.325801973	1.144230734	1.93E-07	3.17E-07
AC008543.3	0.060493478	0.221476893	1.872304682	2.84E-05	4.04E-05
SIRPA	15.50138138	45.14401342	1.542137899	9.24E-33	1.26E-31
GRAMD1A	4.960974681	12.30073406	1.310048913	4.40E-37	1.84E-35
TMPRSS6	0.069317915	0.295344512	2.091098637	5.06E-13	1.17E-12
AL021407.3	0.041406063	0.224180169	2.436744734	6.74E-19	2.29E-18
AC090772.3	0.244647465	0.507035922	1.051383627	1.22E-12	2.75E-12
AC011498.6	0.140205536	0.650710007	2.214471425	1.51E-22	6.72E-22
DENND3	1.898102049	3.835897821	1.015006732	4.29E-25	2.35E-24
BATF3	0.34201229	1.501586129	2.134367154	6.23E-31	6.26E-30
AC136475.3	0.856646009	13.48917833	3.976959498	5.75E-18	1.83E-17
AL158163.1	0.162386515	0.537603478	1.727110637	1.97E-21	8.09E-21
AC007342.9	0.848837257	0.142547386	-2.574046397	2.75E-37	1.25E-35
AC084859.1	1.019802024	0.412992872	-1.304100317	8.92E-22	3.76E-21
AC025449.1	1.291535333	2.709148305	1.068752259	2.12E-21	8.70E-21
RNU6-237P	0.053055301	0.302142807	2.509661778	6.22E-09	1.12E-08
RPL30P7	0.100184982	0.879925891	3.134715758	3.72E-14	9.25E-14
SLC7A8	56.28882894	4.899947328	-3.522010488	1.81E-35	4.42E-34
FSCN1	9.285839792	27.24454454	1.552863078	2.27E-27	1.52E-26
LINC01589	1.196982837	0.192172886	-2.63892576	9.18E-20	3.33E-19
LINC02298	0.89206821	1.923469693	1.108485166	3.67E-16	1.04E-15
MYDGF	22.95190583	53.27986178	1.214976386	5.62E-37	2.26E-35
ALB	54.80645898	21.52170558	-1.348553511	5.03E-16	1.41E-15
MIR6746	0.061490035	0.416908791	2.761307266	2.51E-09	4.64E-09
IGKC	69.86566755	432.7235856	2.630790172	4.55E-08	7.76E-08
ETV7	0.654395974	4.278064482	2.708722451	8.20E-33	1.13E-31
IKBIP	3.95600775	13.7519296	1.797516907	1.92E-36	6.25E-35
NAPSB	2.800682515	9.098204151	1.699803358	4.08E-17	1.23E-16
PRR16	0.459714799	1.570134612	1.772077237	7.81E-25	4.20E-24
AC006064.3	0.165555254	1.109641934	2.744709517	6.22E-32	7.30E-31
AC084357.2	0.185891434	0.379629496	1.030131793	6.95E-09	1.25E-08
SNORD123	0.150153979	1.047172675	2.801984744	3.74E-16	1.06E-15
U62317.1	0.174245529	1.083324874	2.636272412	1.10E-30	1.07E-29
AC018682.1	0.076464423	0.295166162	1.948666777	1.41E-16	4.11E-16
UGT1A8	0.090910173	0.299302478	1.719090572	0.029940156	0.034372828
RNU6-315P	0.061335146	0.360041621	2.553377795	5.44E-06	8.13E-06
AC244517.1	0.222550897	0.519900129	1.224099199	7.51E-05	0.000104124
AC000123.2	0.562960053	1.481750112	1.396197708	1.96E-27	1.31E-26
AC026992.1	1.142241219	0.471733738	-1.27582266	3.93E-19	1.36E-18
PGM5	7.027221042	2.987323218	-1.234100939	1.34E-28	1.02E-27
IRF8	2.671762379	5.705327584	1.094518021	5.28E-13	1.22E-12
RASSF9	2.348302858	0.888559558	-1.402078099	3.18E-29	2.60E-28
STK10	2.866339944	6.655285731	1.215290885	1.93E-31	2.09E-30
PBLD	37.69821967	16.90097934	-1.157389546	4.98E-13	1.16E-12

Table S1. The differentially expressed genes(DEGs) were screened out in TCGA

ID	conMean	treatMean	logFC	pValue	FDR
RNA5SP78	0.079488159	0.377395489	2.2472653	2.10E-10	4.13E-10
NRG3	0.320035543	1.272436077	1.99128914	1.12E-29	9.65E-29
KCNJ13	3.301384003	0.639376054	-2.368334338	1.52E-32	2.00E-31
WBP1LP2	0.352951472	0.839924287	1.250789448	1.68E-29	1.42E-28
JSRP1	0.140938841	0.75687628	2.42498824	2.27E-13	5.38E-13
AC006441.1	0.071102076	0.244456241	1.781612657	8.52E-10	1.61E-09
VWF	18.45550564	116.5560549	2.658900782	1.43E-32	1.89E-31
APOBEC3G	1.122554896	5.688964697	2.34138013	1.89E-35	4.60E-34
SLC18A2	0.146345756	0.616439357	2.074578071	5.65E-08	9.58E-08
ZNF331	7.267121222	3.18999819	-1.187828367	6.58E-20	2.41E-19
TRAV8-2	1.889770391	0.677133581	-1.480698583	2.97E-06	4.52E-06
FABP1	26.40938491	1.126210216	-4.551502653	3.06E-18	9.91E-18
RN7SKP275	0.133887075	0.74990386	2.485688951	2.35E-18	7.70E-18
RN7SL663P	0.082667577	0.335876811	2.022538689	6.79E-14	1.66E-13
AC010186.3	0.686134993	1.376526571	1.004468107	1.03E-19	3.72E-19
IGKV6-21	1.03289805	3.527769219	1.772058322	9.15E-05	0.00012603
CYP2D7	0.061098965	0.365046052	2.578858628	1.39E-16	4.05E-16
GRM1	1.026526231	0.095565601	-3.425135278	1.58E-37	7.73E-36
SEMA6D	9.514390299	1.26975477	-2.905561315	1.35E-37	6.79E-36
OPRL1	0.421510908	0.844054275	1.001765804	5.25E-19	1.80E-18
ASF1B	0.718953047	3.162062183	2.136896278	2.21E-33	3.36E-32
AL590369.1	0.607736082	0.289828453	-1.068245715	9.39E-15	2.42E-14
PRDM16	4.665680464	0.34676225	-3.750068752	2.58E-40	4.22E-38
CTGF	190.8310664	83.9272039	-1.185085632	1.30E-19	4.66E-19
NAP1L6	0.529346378	0.189558375	-1.48156986	4.26E-29	3.42E-28
RETN	0.080380331	0.512194119	2.671776271	1.36E-19	4.88E-19
C10orf99	0.340605909	20.61363852	5.919351906	2.12E-23	1.01E-22
AC012409.3	0.499784776	1.057593746	1.081406691	1.45E-06	2.25E-06
LINC02048	0.02410485	1.285380424	5.736728126	3.02E-36	9.10E-35
MBOAT2	6.588774389	2.229418146	-1.563342896	8.02E-36	2.17E-34
PTPRH	0.135557302	0.785517081	2.53473982	0.016108318	0.018879543
CLSTN2	4.477918213	0.906867522	-2.303864461	3.52E-36	1.04E-34
FCRL6	0.193703582	1.134865804	2.550599173	1.21E-32	1.63E-31
PAQR8	7.767148889	3.03719733	-1.354644476	1.40E-14	3.57E-14
ADORA1	3.49396714	1.625010184	-1.104417281	1.96E-14	4.96E-14
FAM81A	3.655935089	0.581940543	-2.651296791	4.01E-39	3.56E-37
LINC02511	0.043964082	0.603882625	3.779870903	7.14E-05	9.92E-05
PHACTR1	3.1670587	1.487433118	-1.090318808	2.97E-17	9.04E-17
PGA5	0.086921574	0.444431012	2.354173295	3.53E-09	6.45E-09
YBX2	0.280298438	0.698579402	1.317460402	0.009985376	0.011910024
SCPEP1	36.81709557	16.03379685	-1.199259719	2.01E-36	6.48E-35
ELF5	11.67010111	0.047323242	-7.94605242	4.78E-41	1.26E-38
DPEP2	0.361325213	1.798371289	2.315321076	5.88E-37	2.35E-35
MT3	0.906485847	8.786108145	3.276867857	1.05E-05	1.54E-05
AL356356.1	0.133109945	0.801461282	2.590014468	2.64E-21	1.07E-20
AC124067.2	0.123269369	0.278042418	1.173490644	0.000391386	0.000517026
AF064858.2	0.910952535	3.877872546	2.089817598	3.33E-08	5.73E-08
AC007038.2	0.503581722	1.409964141	1.485360646	5.12E-16	1.44E-15
DNAH1	0.413578786	1.120771762	1.438258421	2.29E-16	6.55E-16
MIR6757	0.214901508	0.939099809	2.127602889	9.04E-10	1.71E-09
AL033384.2	0.172763993	0.514625301	1.574719823	1.79E-16	5.17E-16
TRAV5	0.092755032	0.600441044	2.694525143	1.14E-18	3.82E-18
SH3BP1	0.672830315	2.112300025	1.650500151	4.20E-30	3.79E-29
AL358852.1	0.580712997	0.221528504	-1.390332983	1.24E-24	6.59E-24
AC073912.2	0.050980679	0.277327436	2.443567861	1.59E-13	3.78E-13

Table S1. The differentially expressed genes(DEGs) were screened out in TCGA

ID	conMean	treatMean	logFC	pValue	FDR
CDCA5	0.471204965	1.399565671	1.570552537	7.99E-30	6.94E-29
ANKRD2	10.63591439	1.067385606	-3.316790703	1.30E-35	3.30E-34
CENPE	0.141142898	0.437663557	1.632665726	1.36E-28	1.04E-27
ARHGAP42	2.805044653	7.038345746	1.327212649	5.95E-22	2.54E-21
RPS20	132.8400724	268.0015859	1.012551124	2.04E-28	1.52E-27
CPN2	6.425652558	2.704812305	-1.24831449	7.15E-14	1.74E-13
ITLN1	2.467006215	0.196633673	-3.649179045	2.05E-36	6.58E-35
GPR141	0.126524831	0.660689293	2.384551415	1.56E-27	1.06E-26
ATP11A	8.018090472	23.31906001	1.540179033	1.06E-21	4.44E-21
AC060766.6	0.235000933	0.530846354	1.175627865	2.54E-12	5.63E-12
MIR221	0.346671575	0.865591995	1.320117608	0.022265232	0.025857209
SUCLG2	53.0390475	21.86739409	-1.278273561	1.34E-36	4.67E-35
IGKV1OR22-	0.077996608	0.289275261	1.890959661	0.003839613	0.004725607
LINC01929	0.041636436	0.486620094	3.546877407	3.06E-27	2.03E-26
CDS1	15.01953874	5.613819195	-1.419786002	1.22E-32	1.64E-31
BAG1	20.13346401	8.229862317	-1.290655213	9.52E-35	1.94E-33
RNU6V	0.123982349	0.352383267	1.507010681	1.42E-05	2.06E-05
PDE6G	1.806525897	0.815106971	-1.148156627	1.02E-19	3.70E-19
PXK	8.147173806	3.829444073	-1.089164717	7.71E-35	1.60E-33
LINC02542	0.199480918	0.653409974	1.711737726	8.58E-06	1.27E-05
IGF2BP2	2.893530682	1.413791169	-1.033261907	6.90E-23	3.16E-22
SEMA6A-AS:	0.140508804	0.43234198	1.621512397	1.63E-18	5.40E-18
AC005912.1	6.574553694	19.68909981	1.582432285	2.29E-18	7.50E-18
AL450384.2	0.302826407	1.05262366	1.797426808	2.02E-20	7.67E-20
AC005840.2	0.190084825	0.793156273	2.060961783	1.82E-22	8.07E-22
DUTP6	0.305189135	0.838865185	1.458735372	2.50E-21	1.02E-20
AC106782.5	0.193050133	0.495313209	1.35936555	3.21E-11	6.64E-11
FAM171A1	34.25282844	7.709377679	-2.151536812	3.94E-39	3.51E-37
LRBA	13.47082047	6.000199505	-1.166755348	1.87E-32	2.42E-31
PREX2	1.238045343	5.347232305	2.110728201	7.27E-20	2.66E-19
UGT8	24.84969878	7.249328525	-1.777309088	9.39E-32	1.06E-30
IGHV2-5	1.626936919	3.448719093	1.083902308	0.00297793	0.003695355
TRBV5-5	0.044206428	0.312789636	2.822864647	8.54E-16	2.36E-15
KCNK3	3.464236917	14.98458001	2.112869145	1.20E-18	3.99E-18
AC008764.8	0.129767693	0.510344489	1.975540159	3.45E-20	1.29E-19
PTPN22	0.326500886	1.408671588	2.109176494	1.28E-28	9.74E-28
AL353152.1	2.991248158	0.170473751	-4.133126088	2.50E-39	2.56E-37
LRRC37A5P	0.479404452	0.231079468	-1.052854226	1.58E-19	5.64E-19
LINC00271	0.905164494	0.280097088	-1.692253011	1.89E-36	6.15E-35
CSAG1	0.039104925	0.276996601	2.824446068	3.81E-20	1.42E-19
ADRA2C	8.260074147	2.971283164	-1.475068631	2.05E-24	1.06E-23
LINC00462	0.339391906	15.43996781	5.507573775	8.14E-35	1.69E-33
HLA-J	1.269080317	6.961896228	2.455696934	2.81E-31	2.97E-30
EPO	0.962355036	8.855615117	3.201951379	9.26E-09	1.65E-08
CEL	3.982085128	0.537627863	-2.888844249	5.23E-35	1.15E-33
IGLV3-9	1.652075363	8.349854986	2.337471642	2.18E-05	3.13E-05
TFAP2A	2.712707357	0.655883323	-2.048222322	7.17E-34	1.21E-32
SDHAP3	2.647056157	15.78444264	2.576042612	7.12E-35	1.50E-33
HK2	1.589258743	17.0547609	3.423748599	2.37E-36	7.41E-35
AL390728.4	3.023424819	7.849904157	1.376491334	1.31E-19	4.69E-19
AP000894.4	3.705652086	1.14408092	-1.69553834	4.01E-32	4.85E-31
SCN4B	1.765081297	6.988504366	1.985249099	4.80E-22	2.06E-21
VDR	10.8537089	3.359952172	-1.691675519	2.80E-23	1.32E-22
GJB1	20.40892603	7.54861327	-1.434916725	1.01E-25	5.84E-25
NPY6R	3.339575505	8.212190567	1.298102374	1.32E-10	2.63E-10

Table S1. The differentially expressed genes(DEGs) were screened out in TCGA

ID	conMean	treatMean	logFC	pValue	FDR
PCCA	19.67942326	7.331791955	-1.424450187	1.57E-32	2.06E-31
LY96	4.158937982	13.22779512	1.669285529	3.92E-25	2.16E-24
AL356364.1	1.430962038	0.107294211	-3.73734125	3.18E-18	1.03E-17
IGHV4-55	0.661658323	1.828390464	1.466415886	0.000327645	0.000435338
SCARA3	14.22936106	6.179813773	-1.203235613	2.06E-07	3.38E-07
SNORA66	0.432772066	1.605951953	1.891749444	3.75E-14	9.32E-14
SUCLA2	26.78668	13.29967367	-1.010124934	2.86E-36	8.70E-35
SCX	0.266227499	0.677651223	1.347883335	8.73E-05	0.00012049
AP003352.1	0.45892139	1.335152679	1.540685771	2.28E-24	1.18E-23
GPC3	48.85896707	3.517973163	-3.795806982	1.90E-40	3.47E-38
AL137186.2	0.188834699	0.90609508	2.262538559	1.61E-37	7.86E-36
AC144831.1	3.273022317	0.553122434	-2.564952677	6.58E-35	1.40E-33
AQP2	790.8741995	1.563995998	-8.9820676	2.13E-43	1.22E-40
CHMP4BP1	0.199940934	0.541711471	1.437950771	1.21E-06	1.89E-06
ZNF346-IT1	0.137309065	0.362430455	1.400277314	4.18E-08	7.15E-08
SLC2A12	3.539748913	0.332891282	-3.410524036	1.12E-39	1.36E-37
AFAP1L2	17.40745456	4.801922818	-1.858021137	1.51E-31	1.66E-30
TRAV8-4	0.152096451	0.656695569	2.110238227	1.80E-16	5.20E-16
AC090970.1	0.044208799	0.285882767	2.693018213	0.011194352	0.013301935
ADCY2	0.056779558	0.351482859	2.630010804	2.85E-06	4.34E-06
CLK2	5.044888958	10.60115238	1.071326679	1.78E-21	7.33E-21
TRBV10-2	0.041812886	0.420734824	3.330891707	1.44E-14	3.66E-14
AC004975.1	0.150611888	0.429303681	1.51116289	2.31E-08	4.01E-08
CSTA	0.574392105	2.820911127	2.296053391	6.88E-32	8.03E-31
OLFM4	7.463201099	0.545578076	-3.773936983	1.02E-36	3.70E-35
AL158152.2	0.587771692	1.261249666	1.101526105	1.01E-15	2.78E-15
IGHV5-51	11.71673263	56.41247575	2.267443943	6.29E-05	8.77E-05
LINC02121	11.54835293	0.039456535	-8.193207106	2.36E-65	1.62E-61
RNVU1-15	0.081934051	0.411806368	2.329431085	4.17E-08	7.12E-08
IKZF1	0.657617358	2.852471677	2.116892276	1.07E-28	8.16E-28
WNT5A	2.707965281	1.072318992	-1.336475101	4.91E-13	1.14E-12
SLC25A4	27.37052386	12.12265987	-1.174916768	7.24E-32	8.40E-31
RNU1-61P	0.195670623	0.421823108	1.108210957	0.001965107	0.002472204
AC008267.2	0.327395847	0.679511337	1.053461602	1.06E-08	1.88E-08
CBWD4P	0.160200301	0.443237488	1.468203051	5.30E-13	1.23E-12
HIF1A-AS2	0.328371353	6.148215444	4.226767543	1.10E-35	2.85E-34
IGHV1-46	2.743081623	15.71182552	2.517981356	3.83E-06	5.78E-06
AC006480.2	0.23559057	0.62539894	1.408494979	7.04E-09	1.26E-08
IFNG	0.033548735	0.770130505	4.520772651	6.90E-30	6.08E-29
C7	197.4646453	20.64507448	-3.257724844	3.10E-36	9.31E-35
RPL35AP32	0.068402578	0.395546606	2.531725079	1.58E-16	4.58E-16
HHLA2	2.198480475	19.55388748	3.152876831	8.08E-24	3.98E-23
RHOA-IT1	0.121514385	0.425607626	1.808396889	2.02E-12	4.50E-12
AL138724.1	0.329561771	0.890932339	1.434766973	7.12E-32	8.27E-31
ALDH5A1	13.0155959	6.345243701	-1.036493885	4.30E-30	3.88E-29
AC004067.1	0.180212229	0.58833131	1.706931899	2.16E-25	1.22E-24
LINC01213	0.634512162	0.210699821	-1.590458729	1.28E-36	4.48E-35
SNORD93	0.077320328	0.630326737	3.027180201	4.08E-14	1.01E-13
AC138466.4	0.053067813	0.222320006	2.066728784	0.016758906	0.019618603
AQP5	1.125694435	0.172471152	-2.706388289	3.63E-38	2.27E-36
ACTRT3	2.281913744	0.660792448	-1.787975156	1.29E-35	3.28E-34
Z98200.1	0.066444658	0.221594925	1.73769971	3.25E-12	7.16E-12
MCUB	1.083694832	3.357785803	1.631551651	1.60E-27	1.08E-26
RN7SL34P	0.08672696	0.253937275	1.549919737	2.06E-08	3.59E-08
AL353803.1	0.333955309	1.670054013	2.322167809	2.48E-08	4.30E-08

Table S1. The differentially expressed genes(DEGs) were screened out in TCGA

ID	conMean	treatMean	logFC	pValue	FDR
TACSTD2	177.3748228	15.51523219	-3.515044051	8.40E-38	4.55E-36
AMY2B	0.407151139	1.425070527	1.807396977	2.31E-14	5.79E-14
TENT5B	5.932483567	0.979166188	-2.599010554	1.41E-29	1.20E-28
ENTPD2	2.156894928	5.178703673	1.263635111	1.33E-11	2.81E-11
HTRA3	1.515763208	3.125201386	1.043904765	1.20E-07	2.00E-07
GREM1	2.026311349	0.361915718	-2.485130195	7.46E-07	1.18E-06
MIOX	203.7872566	43.78182319	-2.2186599	2.88E-06	4.39E-06
AC026356.2	0.211542978	0.870792292	2.041377843	3.78E-28	2.74E-27
PRSS53	0.054941561	0.64802855	3.560087575	1.19E-33	1.90E-32
PLEKHO1	2.423417603	8.300898816	1.776224528	1.53E-32	2.02E-31
ALDOB	1633.284657	63.12515953	-4.693417314	3.15E-20	1.18E-19
LIG1	2.114498583	4.383243438	1.051683212	6.49E-32	7.59E-31
TRAC	4.743952097	20.71046861	2.12619885	4.54E-23	2.11E-22
ABAT	20.19248172	2.561271717	-2.978886016	2.05E-25	1.16E-24
CASZ1	1.809800976	0.449121786	-2.010652441	4.64E-37	1.93E-35
VAX2	0.143273485	0.331031527	1.208196988	8.89E-05	0.000122546
EPOP	0.444973932	0.965302177	1.117259812	3.80E-16	1.07E-15
SNHG26	0.169398213	0.703647143	2.054433488	1.99E-34	3.77E-33
AL022068.1	0.53255401	0.214820159	-1.309798461	2.97E-13	6.97E-13
UPK1A	1.058490106	0.167192147	-2.662428789	6.60E-11	1.33E-10
ADAM28	0.9447953	2.418892919	1.356273212	4.92E-21	1.96E-20
TMEM91	1.316735075	17.13261506	3.701708364	3.30E-36	9.81E-35
DOCK8	3.243174861	7.619177777	1.232228507	3.91E-27	2.55E-26
AC243829.4	0.009532238	0.255826956	4.746209536	1.09E-33	1.78E-32
VSIG1	0.073522565	1.1310414	3.943320824	4.20E-34	7.44E-33
PEAK3	0.177523565	0.486136274	1.453350243	9.64E-22	4.04E-21
AC018644.1	0.064440033	0.281097144	2.125039654	3.43E-15	9.11E-15
LINC01985	0.096907303	0.224212132	1.210187049	3.32E-06	5.03E-06
RASGRP2	0.901607515	1.940854199	1.106120297	9.59E-17	2.82E-16
PFN2	50.62574597	23.62441845	-1.099592446	9.88E-34	1.62E-32
EWSAT1	0.747417201	0.223222462	-1.743431561	4.74E-27	3.08E-26
DDIT4	32.32598861	145.0282952	2.165568002	2.24E-31	2.40E-30
SLC6A17	1.308381495	0.187985077	-2.799093218	2.00E-36	6.44E-35
DUSP15	4.627770774	1.908322564	-1.278012355	2.85E-34	5.18E-33
AC026691.1	0.852002926	0.327005342	-1.381544183	5.39E-24	2.70E-23
AC138150.2	0.191527303	0.503070358	1.393210114	1.60E-19	5.71E-19
IGHV4-4	0.596255098	2.784694064	2.223517237	0.000209519	0.000282045
AJ009632.2	0.249405544	0.718947628	1.527393146	3.26E-06	4.94E-06
LINC00892	0.094824289	0.259676442	1.45338658	1.16E-16	3.39E-16
AC010809.2	0.181627526	0.419377934	1.207268091	1.50E-08	2.64E-08
DPH6-DT	1.391908185	0.523195944	-1.411640788	3.23E-29	2.64E-28
CCDC127	2.233429819	4.614903889	1.04703968	3.94E-35	8.82E-34
AC233266.2	0.111956039	0.251464734	1.167423731	0.007636607	0.009194296
AC245140.2	0.335995371	0.813549486	1.275788748	4.56E-26	2.73E-25
TRBV7-3	0.406110911	0.945351092	1.218976441	7.44E-05	0.000103171
MIR4326	0.097745146	0.525520423	2.426649868	7.19E-09	1.29E-08
KIAA0895L	1.434815147	6.152614144	2.100334637	1.72E-30	1.63E-29
ADAMTS15	6.290947799	1.576558163	-1.996498995	1.60E-24	8.35E-24
FRZB	14.50754547	45.83801213	1.659741029	7.56E-13	1.73E-12
MIR3609	0.386636506	18.58307157	5.586867312	0.00018316	0.000247565
SPON2	4.512449071	14.75412186	1.709135502	5.89E-17	1.75E-16
COL4A3	9.259925542	2.441788936	-1.923062091	3.33E-35	7.61E-34
TMEM173	9.211673472	19.02865531	1.046638437	6.75E-23	3.09E-22
RN7SL834P	0.239019111	1.24867322	2.385198092	6.38E-23	2.93E-22
GCNT3	8.368454717	2.369634876	-1.820296466	0.019747801	0.023023259

Table S1. The differentially expressed genes(DEGs) were screened out in TCGA

ID	conMean	treatMean	logFC	pValue	FDR
CILP2	0.062836322	0.391584235	2.639652031	0.00459175	0.005615728
CD74	419.7033403	1411.712739	1.750004703	1.32E-30	1.27E-29
AL591845.1	0.967887443	2.545007486	1.39475871	2.79E-08	4.82E-08
CWH43	5.494279625	1.088503335	-2.335584509	2.69E-37	1.23E-35
TMEM45B	18.58362124	1.20224281	-3.950231452	1.09E-41	3.81E-39
AC019257.2	0.106578481	0.231703642	1.120364543	0.002039955	0.002563234
LSAMP	1.256063177	0.22611327	-2.473791466	4.16E-35	9.29E-34
AC004832.5	0.212312167	0.727070708	1.775908626	9.21E-23	4.18E-22
NMUR1	0.568766867	1.22920387	1.111814885	1.89E-13	4.48E-13
AL021707.3	0.332287322	0.895784617	1.430720641	9.77E-17	2.87E-16
ITGB6	23.15368819	3.738364266	-2.630762964	2.16E-32	2.76E-31
POU3F4	0.835144528	0.156478307	-2.416063221	5.73E-49	6.55E-46
AC087284.1	0.018044317	0.253644979	3.813194183	4.37E-19	1.51E-18
HOXB6	16.81898631	3.883185867	-2.114778088	1.94E-34	3.68E-33
BAIAP2L2	5.249974524	12.44906719	1.245655318	5.64E-10	1.08E-09
AL513218.1	0.142617511	0.595938404	2.063012096	4.51E-15	1.19E-14
CCR2	1.050009944	2.717554543	1.371906001	4.03E-15	1.06E-14
AC007344.1	0.050753194	0.281360993	2.470851807	0.000442379	0.000582259
AC015911.7	0.162196999	1.552927104	3.259171074	9.95E-35	2.02E-33
CD1D	0.860631723	4.193998054	2.284858267	3.95E-36	1.15E-34
CYP2A6	0.019672342	0.837936339	5.412599995	6.48E-07	1.03E-06
CD163	4.77661625	17.76826151	1.89524165	1.13E-18	3.78E-18
DCAF11	23.36091185	10.64946329	-1.133315864	4.52E-40	6.84E-38
LINC01315	1.054023569	2.570068371	1.285899611	1.28E-11	2.72E-11
RN7SKP173	0.052262833	0.307925944	2.55872618	4.76E-16	1.34E-15
MFAP3L	17.05089861	5.216904484	-1.708581849	6.56E-33	9.19E-32
RTCA-AS1	5.171181863	1.699710851	-1.605204702	4.96E-35	1.09E-33
RPS3AP38	0.08204843	0.315895997	1.944902027	3.24E-14	8.07E-14
AC084824.1	0.796875654	1.646602804	1.047066062	1.75E-24	9.15E-24
SNORD15B	0.174419887	6.66530525	5.256034503	1.26E-11	2.67E-11
FBLN5	41.056555	12.51781969	-1.713629277	7.81E-32	8.98E-31
CRYZP1	0.109813916	0.297399656	1.437342088	3.76E-09	6.85E-09
AC016876.2	0.147796569	0.590297167	1.997828644	2.94E-35	6.83E-34
HOXA4	2.482873028	5.882780154	1.244487637	5.36E-22	2.29E-21
PRKCA	8.382534028	3.659701811	-1.19566033	9.88E-32	1.11E-30
IGKV2-24	2.562533482	15.65721317	2.61118469	5.47E-05	7.64E-05
DOC2GP	0.068241907	0.271246848	1.990876504	2.11E-14	5.31E-14
AC114939.1	0.127972185	0.439929126	1.781440849	6.48E-14	1.58E-13
COL14A1	16.21072846	6.853839199	-1.241964673	3.05E-14	7.62E-14
FAM3B	6.902502356	0.430629103	-4.002601748	2.02E-40	3.58E-38
ST3GAL6-AS	2.028861533	0.556074906	-1.867319269	1.75E-31	1.90E-30
IGHV3-30	6.065975761	54.654816	3.171536989	2.24E-07	3.65E-07
SMAD9-IT1	0.12419663	0.409204243	1.720195078	1.89E-10	3.73E-10
AC000120.1	0.060980182	0.313825537	2.363550393	5.11E-17	1.53E-16
SMIM24	172.2983811	70.91944676	-1.280655959	5.81E-08	9.84E-08
HIST1H2BL	0.044490352	0.352915892	2.987759994	5.08E-19	1.74E-18
HIBADH	88.20252319	41.82693738	-1.076387561	1.03E-31	1.16E-30
AKNA	1.692198029	4.736547065	1.484937311	4.42E-29	3.55E-28
CSF2RA	0.940421503	3.654180793	1.958168582	1.91E-30	1.81E-29
LILRA5	0.773811113	1.84427015	1.252996646	9.27E-18	2.91E-17
AC073575.2	0.140452414	0.399035432	1.506435432	1.12E-18	3.75E-18
SNORD6	0.775640914	2.907899137	1.906516418	4.72E-19	1.62E-18
TMPRSS2	21.22980094	1.59799953	-3.731751955	2.14E-37	1.00E-35
TMCC1	3.925239806	13.59209978	1.791915648	1.41E-35	3.55E-34
CASP4	3.514193972	8.346382179	1.247957157	3.45E-34	6.23E-33

Table S1. The differentially expressed genes(DEGs) were screened out in TCGA

ID	conMean	treatMean	logFC	pValue	FDR
LINP1	0.165611812	2.033279224	3.617930871	2.73E-36	8.38E-35
RN7SL566P	0.134847849	0.352473227	1.386181169	9.03E-07	1.42E-06
RNF166	2.037512922	5.059567166	1.312204762	9.34E-36	2.49E-34
GALNT5	0.217060197	0.759312934	1.806599385	0.018455801	0.021552355
HLA-L	0.832115805	2.433738542	1.548317961	2.84E-21	1.15E-20
TRBV10-3	0.188714398	0.79042779	2.066429173	1.05E-15	2.90E-15
MIR3153	0.059164126	0.30774623	2.378946601	3.24E-07	5.23E-07
LINC01138	0.624317926	1.388918321	1.153608964	1.65E-20	6.33E-20
KCNA3	0.197772511	0.532086814	1.427819735	8.36E-14	2.03E-13
CACNB1	0.281033504	0.705075235	1.327035072	6.93E-18	2.19E-17
CTBP1-AS	0.113337408	0.457503607	2.013159007	2.28E-23	1.08E-22
AC073869.3	0.052973365	0.240861785	2.184866461	1.02E-16	3.00E-16
AC008147.3	0.081314695	0.433241285	2.413582734	0.000590401	0.000770485
PRR15L	21.49308224	4.877756824	-2.139582648	7.34E-33	1.02E-31
CD276	6.087684542	13.8735561	1.188372123	7.40E-33	1.02E-31
GTSF1	0.089810436	0.238277443	1.407687388	8.11E-19	2.74E-18
RAB7B	0.426023451	1.749203007	2.037692982	2.58E-33	3.88E-32
AC025580.3	0.051429403	0.798072769	3.955854993	3.67E-32	4.46E-31
PTPRE	1.181018826	3.802451735	1.686897973	2.11E-33	3.23E-32
LINC00379	7.239985654	0.343279104	-4.398532892	5.85E-41	1.45E-38
AC053513.1	0.216829319	0.652253033	1.5888719	1.20E-12	2.72E-12
HLA-P	0.020674745	0.229237775	3.470903328	3.83E-27	2.50E-26
RASL11B	16.23551784	0.813254989	-4.319301823	1.18E-40	2.42E-38
CXCL11	0.435641105	5.137675006	3.55990364	1.24E-30	1.20E-29
NLGN3	0.626640136	0.266193127	-1.235163854	8.75E-24	4.29E-23
ZNF337	0.439783172	1.10558381	1.329944088	4.56E-24	2.30E-23
AL590666.1	0.102628046	0.255259568	1.314539994	2.03E-06	3.12E-06
GOLGA6L9	0.129306125	0.329485848	1.34942589	2.23E-13	5.27E-13
KDELC1	3.154761333	7.42597692	1.235049966	1.61E-28	1.21E-27
EXOSC5	5.796388361	14.18738413	1.291382446	2.89E-35	6.73E-34
DUSP10	1.257036978	3.303967931	1.394172594	3.88E-28	2.80E-27
AC021146.12	0.643607603	0.184672742	-1.801210432	2.27E-22	1.00E-21
EPS8L1	4.630310065	1.510631278	-1.615957242	6.65E-24	3.30E-23
AL589745.1	0.527170553	1.399829763	1.408909698	2.68E-09	4.95E-09
CCNI2	1.595631393	0.210784715	-2.920285254	7.43E-39	5.86E-37
FAM86GP	0.292229765	0.715025707	1.290891979	6.44E-15	1.68E-14
POMGNT2	12.50049179	5.663017618	-1.142341931	1.01E-35	2.67E-34
AL137013.1	0.187494947	0.44461794	1.24571445	1.60E-15	4.33E-15
AL158071.2	0.444675091	0.935840117	1.073510483	3.99E-06	6.01E-06
MIR7111	0.133812167	0.815199782	2.606944369	3.38E-16	9.57E-16
AXIN2	1.076281854	0.492308295	-1.128421982	4.51E-23	2.10E-22
PARVG	0.340106073	2.536222293	2.898624527	4.53E-38	2.72E-36
AC010761.6	0.529955447	0.231180551	-1.196851048	3.16E-18	1.02E-17
AC004264.1	0.332652392	0.970038299	1.544026302	2.25E-09	4.18E-09
SCGN	1.127248955	30.33762812	4.750230218	7.96E-20	2.90E-19
AC245884.8	0.222197161	1.305749995	2.554966411	1.03E-21	4.32E-21
PITRM1-AS1	0.087117755	0.360992507	2.050930216	2.19E-34	4.11E-33
PRUNE2	6.921025583	17.81166695	1.363764799	6.01E-15	1.57E-14
SLC47A2	7.855981212	0.541198923	-3.859560607	2.40E-29	1.98E-28
RPL11P3	0.186554939	0.555282818	1.573622203	2.14E-15	5.77E-15
AC138207.5	0.884341435	4.936060112	2.480684572	6.34E-39	5.14E-37
VMP1	13.10452178	32.49707459	1.310245145	9.60E-25	5.13E-24
SIT1	0.534095041	3.558756068	2.736204653	9.12E-28	6.31E-27
MEFV	0.116192453	0.458298683	1.979771777	1.86E-29	1.55E-28
NUAK2	29.82674314	6.831295251	-2.126375402	8.27E-33	1.14E-31

Table S1. The differentially expressed genes(DEGs) were screened out in TCGA

ID	conMean	treatMean	logFC	pValue	FDR
C6orf132	3.048809383	1.083022139	-1.493183219	1.18E-15	3.24E-15
MTCO2P12	21.64867976	9.295948898	-1.219605002	9.11E-09	1.62E-08
AC022509.2	0.845565649	6.118481481	2.855184968	4.78E-27	3.10E-26
AL645924.2	0.044946693	0.289508133	2.687316993	8.87E-15	2.29E-14
EIF4EP1	0.108532114	0.417804919	1.944707483	7.53E-14	1.83E-13
ARL4C	8.339481043	24.57319224	1.559055772	2.40E-17	7.34E-17
AC023818.1	0.112477131	0.38616023	1.779567891	4.71E-19	1.62E-18
IL2RG	3.815911381	13.90997645	1.866020405	2.53E-23	1.20E-22
AL391244.3	0.419782008	1.092350205	1.379723215	9.00E-22	3.79E-21
LINC00987	0.77125789	1.550870182	1.00779268	3.34E-18	1.08E-17
LINC02600	1.396893071	0.637764007	-1.131127006	7.14E-19	2.42E-18
AL139275.2	0.793856698	0.141351525	-2.489591152	1.60E-36	5.37E-35
NPIPB5	0.066270527	0.511438065	2.948120246	3.01E-23	1.42E-22
ZNF581	5.687644333	11.79290736	1.052016279	6.50E-31	6.52E-30
RNA5SP151	0.087270578	0.289398741	1.729491379	5.71E-07	9.08E-07
AC124947.2	0.478847612	0.192314059	-1.316102372	2.60E-16	7.43E-16
AL512656.1	0.088754352	0.217758277	1.294837795	1.20E-05	1.75E-05
TRBV12-4	0.138878832	0.845183387	2.60543769	1.30E-18	4.34E-18
MMP16	0.077422438	0.560872813	2.856850009	1.52E-27	1.03E-26
AL359095.1	0.032485727	0.2842669	3.129368232	2.70E-06	4.12E-06
SLAMF6	0.625194848	3.704744733	2.566996346	8.80E-29	6.82E-28
AC010618.3	0.270740151	0.618489669	1.191840644	4.93E-13	1.14E-12
IGKV1-39	0.174729916	1.194486311	2.773191775	0.010642987	0.012665037
RN7SL145P	0.131045891	0.373845723	1.512370909	8.86E-09	1.58E-08
AXL	8.205070653	24.92379701	1.60293621	4.57E-29	3.66E-28
LENG8-AS1	1.052020947	3.302748556	1.650503708	3.37E-22	1.46E-21
AL162274.2	0.752120361	1.582992774	1.073619212	1.05E-14	2.69E-14
AL132989.1	0.684699374	1.931372104	1.496083548	3.45E-13	8.07E-13
PPIEL	0.417853021	1.089029516	1.381975584	3.66E-13	8.54E-13
AL109976.1	0.754934557	0.184213344	-2.034974012	1.31E-13	3.15E-13
AC007919.1	0.172674255	0.588945029	1.770079986	5.48E-05	7.66E-05
GPR37	0.517171718	1.65630418	1.679252361	0.000381906	0.000504989
BCL11B	0.334573893	1.048570882	1.648027608	6.76E-21	2.66E-20
RPS2P7	0.26910057	0.70203299	1.383393382	1.02E-22	4.63E-22
ITGB1-DT	0.074538423	0.298452208	2.001443732	3.27E-19	1.14E-18
PLAC8	0.304165923	1.103140788	1.858686492	2.02E-22	8.92E-22
AREL1	9.949713139	4.373315148	-1.185927617	1.48E-37	7.38E-36
CCM2	3.021729278	6.501671091	1.105436162	2.50E-37	1.15E-35
THRB	6.16222875	1.629610372	-1.91892517	6.43E-38	3.64E-36
CD19	0.134982263	0.29865017	1.145686696	0.001834211	0.002312618
AC023794.2	1.396663994	0.46428762	-1.588894264	6.92E-28	4.85E-27
CDH5	12.22966257	29.40506861	1.265680258	4.06E-19	1.40E-18
ASGR1	0.176208928	0.524056897	1.572436434	1.07E-22	4.82E-22
SH2D5	0.09833076	0.537879154	2.451567382	1.70E-26	1.05E-25
AC067852.3	0.167249728	0.609204143	1.864921886	6.88E-18	2.18E-17
LINC02384	0.864156558	4.964735538	2.522352258	7.27E-16	2.02E-15
AP000347.2	0.106717594	0.265008959	1.312243091	1.80E-09	3.35E-09
TPX2	0.917095694	4.347690937	2.245105203	8.60E-34	1.43E-32
GK-IT1	0.043120873	0.257425832	2.577698555	3.06E-09	5.61E-09
AL137244.1	0.103611778	0.22863303	1.141845825	9.17E-06	1.35E-05
PLA1A	7.496634472	23.00606604	1.617699344	1.25E-24	6.63E-24
SLITRK5	0.034597019	0.377350013	3.447183667	1.90E-12	4.24E-12
RNU6-216P	0.092053288	0.39483602	2.100712458	2.61E-10	5.11E-10
ATP2B1-AS1	1.031560103	0.475862957	-1.116209821	8.78E-27	5.56E-26
PTP4A2P2	0.265044656	2.190796107	3.047147867	1.83E-33	2.83E-32

Table S1. The differentially expressed genes(DEGs) were screened out in TCGA

ID	conMean	treatMean	logFC	pValue	FDR
TXLNB	0.101656461	0.372347149	1.872946406	1.17E-23	5.69E-23
C1QL1	2.116853707	26.62247187	3.652651055	3.12E-18	1.01E-17
KCTD1	6.309072724	1.589934088	-1.988461022	1.39E-35	3.53E-34
AL442125.1	0.102533363	0.242228472	1.24027504	2.92E-14	7.30E-14
SUCLG2-AS1	1.044462781	0.356145849	-1.552221002	4.52E-34	7.94E-33
GUCA1B	0.246989361	0.557863668	1.175463698	5.26E-11	1.07E-10
CHST11	2.084835428	7.156856489	1.779392546	1.13E-32	1.53E-31
RNY3P16	0.206052521	1.402531039	2.76694868	2.79E-24	1.43E-23
GRAMD1C	6.268162222	2.420786875	-1.372566447	4.54E-33	6.58E-32
PLPPR5	0.019206032	1.661342626	6.434646341	8.05E-25	4.32E-24
DNAJC3-DT	3.549417015	1.396473157	-1.345794242	1.95E-28	1.45E-27
ADAMTSL2	9.837698514	4.030193252	-1.287471824	3.65E-27	2.39E-26
AC015813.1	0.572767729	1.860718595	1.699837771	2.02E-16	5.80E-16
LINC01431	0.864626436	0.376580774	-1.199117603	3.11E-23	1.46E-22
RPL7L1P9	0.023536097	0.308625371	3.712909682	2.28E-19	8.02E-19
PPM1N	0.189829642	0.412476436	1.119606421	2.63E-12	5.83E-12
TRAF3IP3	0.35790943	1.356799832	1.922541437	1.83E-25	1.04E-24
EIF4EBP3	6.625957875	16.00946801	1.272724432	1.46E-29	1.23E-28
CXCL2	2.548180188	7.809354085	1.61573593	8.04E-08	1.35E-07
CRACR2A	0.08711407	0.402706571	2.208751361	1.11E-33	1.79E-32
MEIS3P2	0.849971935	0.390244623	-1.123036451	6.31E-18	2.00E-17
MEOX2	0.515356927	1.306766375	1.342357369	0.001389256	0.001768834
PTGFR	2.835108582	0.926759906	-1.613136453	6.90E-30	6.08E-29
CD248	8.726147347	29.91070497	1.777245174	1.82E-23	8.77E-23
GDF7	2.248676313	0.819640679	-1.456012514	1.48E-32	1.95E-31
LINC01322	0.201254498	0.553553287	1.459701177	3.48E-10	6.74E-10
PALD1	2.068786331	8.220696736	1.990476025	4.77E-36	1.37E-34
RNF149	8.161234681	20.52890357	1.330797243	3.45E-36	1.02E-34
APOBEC3C	5.735219014	24.61184262	2.101432187	5.44E-32	6.44E-31
ADGRL1	4.835866639	2.355932501	-1.037476253	7.67E-23	3.50E-22
ATG12	2.466337486	5.002604325	1.020309121	4.17E-36	1.21E-34
PCDH7	1.27740015	0.486410964	-1.392962873	6.50E-21	2.56E-20
ALDH1A2	6.188338667	1.911341814	-1.694966347	3.22E-32	3.95E-31
GTF2IP20	0.852427918	2.208304935	1.373289652	3.14E-13	7.35E-13
GRIK3	0.091674858	3.17478796	5.11399031	1.95E-37	9.26E-36
RNU4-62P	0.346408164	2.113860407	2.609335271	9.51E-15	2.45E-14
ISG15	13.7994606	40.46061088	1.551906227	8.66E-26	5.06E-25
TBXAS1	1.432715124	5.04850144	1.817103435	8.93E-36	2.40E-34
SHISA2	4.035382526	0.752719941	-2.422520341	1.87E-35	4.57E-34
CCR4	0.37777026	1.049633981	1.474305297	4.56E-13	1.06E-12
AC083798.1	0.181335494	0.487192299	1.425829987	0.000612976	0.000799034
HLA-K	1.95583844	5.756490098	1.557402223	1.12E-12	2.54E-12
SYT12	0.1624993	0.616284338	1.923162625	1.85E-09	3.45E-09
AL109741.2	0.069847908	0.268675661	1.943576814	1.70E-13	4.05E-13
LPAR5	0.543109325	2.480662166	2.191410734	1.38E-32	1.83E-31
SNORA54	0.151229023	12.473909	6.366034789	0.022391863	0.02599547
SLC5A12	37.13157583	13.53986359	-1.455433339	0.000411843	0.000543248
RNU6-125P	0.10269131	0.352868366	1.780816004	3.28E-08	5.64E-08
LRRN1	0.641438011	0.244892487	-1.389161334	4.97E-34	8.63E-33
AC068790.7	0.110991305	0.381369551	1.780742999	1.92E-15	5.18E-15
LINC02356	0.114659569	0.233014964	1.023065846	7.21E-06	1.07E-05
RNU6-45P	0.073800738	0.220939649	1.581945206	3.00E-06	4.56E-06
IGLVI-70	0.060746185	0.581720875	3.259461374	0.0039776	0.004889005
FSIP2-AS1	0.074798642	0.282715429	1.918266634	1.93E-14	4.87E-14
MYH7B	0.16935475	0.400947274	1.243364077	3.02E-08	5.20E-08

Table S1. The differentially expressed genes(DEGs) were screened out in TCGA

ID	conMean	treatMean	logFC	pValue	FDR
DOK7	1.273456439	0.235278725	-2.436306835	1.60E-30	1.53E-29
PIK3IP1-AS1	0.16646616	0.460649791	1.46844143	8.03E-12	1.72E-11
AC008013.1	0.02449986	0.282473929	3.527272289	2.00E-24	1.04E-23
RNU6-1206P	0.078823548	0.263090354	1.738859767	0.000106	0.000145519
AC011462.4	0.384346072	1.666939175	2.116723632	2.50E-18	8.18E-18
AC018738.1	11.31228107	23.67082709	1.065220245	1.39E-14	3.55E-14
CYSTM1	108.2925346	52.29856764	-1.050090451	1.04E-30	1.02E-29
GMFG	6.790638792	24.41895476	1.846382248	2.94E-35	6.83E-34
SLC25A5	313.8113806	143.5702395	-1.128140948	3.48E-33	5.13E-32
TRNP1	10.33964227	2.56999227	-2.008350346	5.07E-31	5.15E-30
GABPB1-AS1	0.45966771	1.176202694	1.355473471	2.55E-09	4.70E-09
ARHGEF6	3.482305297	8.000884894	1.200116879	2.19E-28	1.63E-27
VSIG8	1.052724628	0.156703924	-2.748014891	6.15E-15	1.60E-14
MIR7851	0.058426904	0.32337507	2.468503715	4.20E-07	6.74E-07
ATRNL1	1.41614039	0.36887733	-1.940751262	5.60E-31	5.66E-30
SCOC	19.77360851	9.669348494	-1.032085583	6.69E-35	1.43E-33
RIPOR2	1.185723388	2.471663038	1.059714585	1.81E-15	4.90E-15
AL139280.1	9.836985751	0.089441661	-6.781125519	1.18E-42	5.90E-40
AC044849.1	0.096537564	0.312691086	1.69557576	1.10E-16	3.23E-16
SLC15A3	2.414404156	7.561034736	1.646916488	2.07E-32	2.65E-31
AC007953.1	0.425381453	2.477238388	2.54190367	2.78E-15	7.41E-15
EVA1C	2.4942814	5.489176606	1.13796552	1.15E-22	5.16E-22
LINC02167	0.01304284	0.359030193	4.782775235	0.000887442	0.001145999
BRCC3	9.277266306	4.483319082	-1.049132574	1.23E-36	4.33E-35
KIAA0319	0.051204146	0.378006195	2.884077335	1.99E-23	9.52E-23
SH3PXD2B	1.633679643	4.718738435	1.530276098	3.75E-25	2.07E-24
FREM1	4.800607591	0.465917184	-3.365071566	1.35E-38	9.77E-37
MIR553	0.187885165	0.608221677	1.694746074	0.000184605	0.000249469
DUSP5P1	0.033008682	0.561697978	4.088877161	2.35E-34	4.35E-33
NRBF2P5	0.066555866	0.300421489	2.174350283	1.72E-19	6.10E-19
MARVELD1	6.114439847	13.19401756	1.109591687	5.36E-22	2.29E-21
SKIDA1	0.627480321	0.279658307	-1.165905026	4.37E-24	2.20E-23
HS3ST3B1	2.009527624	0.841999608	-1.254964943	1.52E-30	1.45E-29
PABPC1P3	0.296712468	0.681397956	1.19943207	9.39E-19	3.16E-18
TEX15	0.010777577	0.606018266	5.813256484	2.19E-13	5.18E-13
SMIM25	0.257030096	1.518804929	2.562927387	6.13E-33	8.63E-32
TRMT1	4.00538875	9.001299178	1.168190971	7.66E-38	4.22E-36
MIR589	0.189078804	0.4399576	1.218376848	9.60E-09	1.71E-08
HRAT92	0.244673787	0.531951025	1.120433884	1.10E-05	1.62E-05
BRIP1	0.119980233	0.433330819	1.852672104	7.61E-32	8.78E-31
CCBE1	1.732462516	0.489675237	-1.822926992	7.61E-30	6.63E-29
ALOX5	2.980667235	12.6033022	2.08009456	3.05E-25	1.69E-24
IGHV4-39	10.18009334	58.14495383	2.513903202	0.000176906	0.000239455
LINC02351	0.567380842	5.740939918	3.338897614	9.82E-08	1.64E-07
SNORA33	1.123508455	3.743426416	1.736348415	2.50E-11	5.20E-11
IGFLR1	0.362848168	1.149624678	1.663725047	4.00E-23	1.86E-22
AL450998.2	0.095521077	0.332924604	1.801304484	8.38E-17	2.47E-16
FTLP15	0.033773816	0.646487475	4.258645338	3.82E-22	1.66E-21
HIST1H4I	1.982742971	4.298443565	1.116316696	1.30E-11	2.76E-11
AC087683.2	0.179873357	0.367402406	1.030379563	0.002219625	0.002780846
AC023024.2	0.391276198	3.680516616	3.233649027	9.74E-31	9.58E-30
AC010998.2	0.070062366	0.221218608	1.658761126	3.84E-10	7.42E-10
RN7SKP271	0.077143906	0.24679902	1.677712567	5.85E-06	8.72E-06
IGLV3-12	0.107528232	0.512671375	2.253318853	3.58E-08	6.15E-08
SYNE4	2.431315445	0.48135982	-2.336549458	5.86E-35	1.26E-33

Table S1. The differentially expressed genes(DEGs) were screened out in TCGA

ID	conMean	treatMean	logFC	pValue	FDR
AL133410.1	0.245266753	0.527486407	1.104782231	1.29E-05	1.88E-05
AC025171.4	0.167365533	0.980219953	2.550103063	2.23E-27	1.49E-26
AC022182.2	0.10863025	0.259309966	1.255251753	9.00E-17	2.65E-16
CAB39L	9.514609514	3.04389965	-1.644223651	3.13E-35	7.19E-34
RSPH9	0.443295907	1.000483355	1.174355218	5.34E-18	1.70E-17
SLC5A1	2.409237749	11.15656198	2.211243839	1.46E-05	2.12E-05
CR1	1.106808736	0.290158861	-1.931491042	5.77E-10	1.10E-09
ATP6V1G2	0.972420825	0.464731309	-1.065183948	1.96E-25	1.11E-24
AC110285.2	0.562439891	1.814949524	1.6901586	1.44E-05	2.09E-05
RNU6-407P	0.073867338	0.456753948	2.628408716	1.50E-09	2.81E-09
AL031705.1	0.052757477	0.221974166	2.072944304	3.04E-13	7.13E-13
AF131215.7	0.582491901	0.177725418	-1.71258796	5.42E-28	3.85E-27
AC005785.2	0.184874462	0.757598718	2.03488794	1.06E-14	2.73E-14
LTB	3.023133071	6.293113535	1.057729487	3.69E-08	6.34E-08
OSMR	9.402279542	26.62434045	1.501663308	1.12E-25	6.47E-25
BNIP3P16	0.084456721	0.238324091	1.49664066	0.00052375	0.000686245
GSDMA	0.174058689	0.478996959	1.460442657	1.34E-09	2.52E-09
SFXN5	3.497616972	1.479127488	-1.241625901	7.38E-35	1.55E-33
CAMKK1	0.824798469	2.284343874	1.469666282	2.84E-32	3.51E-31
AL928654.2	0.738447469	2.173388958	1.557379186	1.36E-11	2.88E-11
PLA2R1	10.68592537	1.316313958	-3.021136309	1.84E-18	6.05E-18
CD79A	1.308517064	4.445079049	1.764276337	1.02E-09	1.92E-09
LINC02577	0.007771186	0.485712515	5.965824067	1.09E-16	3.19E-16
BTBD11	2.219668311	0.779528753	-1.509669965	5.20E-25	2.84E-24
REV3L-IT1	0.07923046	0.229012348	1.531298307	0.024309171	0.028140556
ZNF320	3.640963903	8.058748898	1.146235443	2.41E-19	8.47E-19
SLC9A4	12.84169881	0.161819483	-6.310306935	1.39E-42	6.65E-40
ZFAS1	14.12676968	45.20327922	1.677995828	5.87E-38	3.39E-36
NRIR	0.057481411	0.321269818	2.48261807	1.20E-20	4.66E-20
AC017048.3	0.341540392	1.200181064	1.813123959	3.32E-15	8.82E-15
KLHL3	2.635180489	0.834973952	-1.658098681	6.96E-30	6.12E-29
AC068858.1	0.004890102	0.336092614	6.102850342	1.17E-32	1.58E-31
AC020594.1	0.211982826	0.613809802	1.533844297	2.93E-12	6.47E-12
TFPI	9.58249375	20.08668751	1.067766612	8.01E-15	2.07E-14
DBT	8.216476389	2.868898877	-1.518022714	1.14E-40	2.39E-38
INKA1	1.97034924	4.941061567	1.326369665	2.06E-20	7.81E-20
FKBP9P1	0.406839473	1.969950795	2.275628029	9.61E-06	1.41E-05
AC078993.1	0.075040845	0.306914151	2.032087194	1.28E-07	2.12E-07
NR1I3	1.192470162	0.217708335	-2.453484618	2.63E-32	3.30E-31
EME2	0.541334508	2.03330192	1.90923219	2.23E-19	7.86E-19
SNHG12	0.598911894	4.772296704	2.994268052	2.39E-37	1.10E-35
PCDHB16	1.583781643	3.232568051	1.029307298	2.87E-05	4.09E-05
ATP5MC1P3	0.057498489	0.230933085	2.005878923	1.06E-07	1.76E-07
SLC6A8	25.86596617	81.88765431	1.662590881	2.95E-24	1.51E-23
AP000347.1	0.134307988	0.346059731	1.365475961	1.12E-09	2.10E-09
CNNM2	2.685589222	1.339853339	-1.003163561	6.02E-27	3.87E-26
NEAT1	11.05923219	34.95294304	1.660162708	2.89E-09	5.31E-09
AC124854.1	1.089440548	5.830667031	2.420073469	5.35E-12	1.16E-11
AL139123.1	0.082770263	0.360858653	2.124249398	1.48E-14	3.77E-14
CA4	10.62316754	4.044865668	-1.393050306	1.46E-20	5.62E-20
RPL36AP26	0.056592634	0.226051329	1.9979642	1.15E-08	2.03E-08
SNORA77	0.117443957	0.703215212	2.581993792	4.51E-19	1.55E-18
AC011352.1	0.04359626	1.920661092	5.461254773	4.77E-31	4.87E-30
CYTIP	2.064335346	4.931662954	1.256396853	2.22E-16	6.37E-16
NT5DC3	1.112540356	5.33735589	2.262267544	4.59E-31	4.70E-30

Table S1. The differentially expressed genes(DEGs) were screened out in TCGA

ID	conMean	treatMean	logFC	pValue	FDR
LINC01018	1.2876631	0.257171604	-2.323951921	1.66E-32	2.18E-31
ZEB2-AS1	0.19662036	0.484196453	1.30017979	3.30E-18	1.07E-17
LRG1	2.489034007	5.43212172	1.125929866	0.000357884	0.000474077
RNU6-1223P	0.105880317	0.546848944	2.368707949	9.59E-12	2.05E-11
DACH1	6.507152597	1.307835817	-2.314844947	2.23E-35	5.34E-34
CD200	4.497830722	14.55637593	1.694349945	1.41E-30	1.36E-29
DGCR9	0.208328068	3.437848526	4.04457685	1.50E-37	7.43E-36
LMO7	10.97207676	4.721159131	-1.216623605	9.04E-31	8.90E-30
AC069209.1	0.055201966	0.479997948	3.120236695	5.11E-37	2.08E-35
AC007387.1	0.199805525	0.709832488	1.828882132	1.96E-22	8.65E-22
AC116345.1	0.347109887	1.978532634	2.510966497	5.36E-07	8.54E-07
RHOBTB3	14.44933147	4.872251806	-1.568342144	5.55E-31	5.62E-30
SLC7A7	32.90210566	15.28204536	-1.106342268	0.016778568	0.019640502
AKR1C2	3.012281661	1.251670804	-1.267001499	0.000733587	0.000951435
HUNK	4.698769292	1.440625397	-1.705587691	5.34E-32	6.34E-31
DOCK10	1.270712457	3.489792394	1.457503607	1.57E-26	9.76E-26
EFHD2	10.82617878	29.13925687	1.428439968	7.06E-35	1.49E-33
AC046143.1	0.399306842	0.881937974	1.143179403	5.04E-22	2.16E-21
BHMT	162.9281698	71.68400267	-1.184512962	0.006948433	0.00839373
IGLV2-11	10.21579816	39.00749926	1.932949584	2.11E-06	3.24E-06
RPL37P6	0.39315398	1.227342303	1.642371303	3.21E-16	9.11E-16
LINC01843	0.734497359	2.935287205	1.998672463	5.23E-23	2.42E-22
AC008771.1	3.743810764	1.154918865	-1.69671601	2.86E-38	1.85E-36
KCNN1	0.048862429	0.965840504	4.304987469	1.80E-28	1.35E-27
PLAUR	1.890214832	5.432327378	1.523020213	2.41E-21	9.86E-21
TST	39.88242282	18.93979191	-1.074332575	1.85E-16	5.33E-16
EFNA3	0.495254386	2.558876447	2.369268832	3.67E-34	6.58E-33
AP003721.4	0.732516388	0.358155059	-1.032276715	1.18E-22	5.30E-22
UCHL1	34.07244613	8.608046433	-1.98484776	4.98E-31	5.07E-30
ATP6V1A	73.65001917	32.1654159	-1.195175558	3.77E-35	8.46E-34
Z93241.1	0.051342081	0.281070073	2.452716186	2.29E-11	4.79E-11
AC006272.2	0.156066015	0.563393263	1.851985905	1.72E-17	5.32E-17
ALDH6A1	74.57451519	9.606474867	-2.956603663	7.95E-34	1.33E-32
AL024508.1	0.128651828	0.289249157	1.168840803	4.34E-06	6.53E-06
AC007878.1	0.124091059	0.587720868	2.243731957	6.22E-15	1.62E-14
MIR1249	0.134043078	0.735103214	2.455250105	1.61E-18	5.33E-18
AC156455.1	0.231366171	1.954357258	3.078444376	1.22E-32	1.64E-31
TNFSF11	0.055632971	0.250587558	2.171302735	5.90E-05	8.23E-05
AGR3	1.37164739	0.33103386	-2.050858957	3.20E-29	2.61E-28
AC008735.1	0.061577444	0.371671466	2.593554034	1.34E-18	4.46E-18
PLEKHN1	0.216677001	1.178643091	2.443508983	4.40E-31	4.51E-30
TP73	0.029289927	0.245782348	3.06890478	7.64E-35	1.60E-33
C7orf61	0.071046797	0.265264539	1.90059032	2.11E-21	8.64E-21
HSD17B7P2	0.575905651	1.397143087	1.278575396	3.83E-13	8.95E-13
FGD2	0.269128156	1.603339915	2.57471508	1.81E-34	3.45E-33
AC008105.3	0.051765831	0.825717928	3.995576987	4.20E-33	6.12E-32
AL928921.1	0.665300456	0.229312225	-1.536692752	1.13E-36	4.05E-35
PAFAH2	13.64466783	6.629820497	-1.041295559	2.89E-39	2.80E-37
ASPHD1	0.907101764	5.709440909	2.654013164	2.66E-30	2.46E-29
SCARNA21	0.281411142	5.568094176	4.306432259	0.000125481	0.000171586
WDFY3-AS2	5.034594472	2.270668448	-1.14875851	1.95E-28	1.45E-27
GAPDHP63	0.15671585	0.459136792	1.550772946	6.94E-25	3.75E-24
RNU6-548P	0.050078837	0.266062457	2.409491987	9.30E-07	1.46E-06
RN7SKP74	0.110412243	0.313906981	1.507436954	3.36E-05	4.77E-05
ATP8B3	0.162560979	1.714730632	3.39892906	9.01E-37	3.38E-35

Table S1. The differentially expressed genes(DEGs) were screened out in TCGA

ID	conMean	treatMean	logFC	pValue	FDR
ASS1	292.4275089	46.38766543	-2.656265887	2.32E-15	6.22E-15
AC244035.1	0.071883294	0.658871218	3.196268071	6.72E-26	3.96E-25
SSC4D	3.103315003	0.626701394	-2.307960039	3.63E-38	2.27E-36
AC092902.2	0.149906984	0.378056975	1.334536072	8.01E-15	2.07E-14
CXCL9	2.611677277	32.86933179	3.653693587	4.37E-32	5.23E-31
AC007098.1	0.106151122	0.572507396	2.431174709	2.41E-28	1.78E-27
HIST1H2BC	2.682778747	5.801798546	1.112772124	3.83E-06	5.78E-06
ITM2BP1	0.035137487	0.255483193	2.862145483	2.41E-16	6.91E-16
FXYD1	0.634122951	2.165892981	1.77212746	0.020860929	0.024271543
ARG1	0.056402044	0.281505714	2.319344853	1.47E-12	3.31E-12
MIR514B	0.390470938	1.31106583	1.747453048	8.17E-11	1.65E-10
CCDC74B	0.535560888	1.273610003	1.249801064	3.86E-10	7.45E-10
LIMS1-AS1	0.113409345	0.549407112	2.276336056	4.25E-16	1.20E-15
LINC02303	2.518065288	0.642811841	-1.96984728	1.08E-36	3.89E-35
HMCN1	0.851387547	2.06073792	1.275273144	1.06E-15	2.91E-15
NHLRC4	6.565288281	0.657599657	-3.31957691	8.36E-40	1.08E-37
MIR6859-1	0.086435225	0.523918746	2.599651803	6.30E-11	1.28E-10
RCN3	4.469268711	12.36701907	1.468387104	5.89E-19	2.01E-18
IL12RB1	0.281273986	1.74229676	2.63094234	1.39E-36	4.81E-35
B3GALT2	0.695039127	0.243777024	-1.51153204	2.57E-27	1.71E-26
AC092535.4	0.684499673	4.749027837	2.794510456	1.10E-23	5.38E-23
CARD14	0.069239688	0.427836773	2.627389363	6.29E-20	2.31E-19
SLC5A3	38.45969168	10.00933542	-1.942001008	8.73E-26	5.09E-25
AC138028.4	0.342256766	1.258070924	1.878062287	2.75E-17	8.37E-17
RNF207	0.845602467	1.913290193	1.178004216	3.19E-08	5.50E-08
COL18A1-AS	0.808831245	0.213232132	-1.923413873	3.71E-30	3.38E-29
AC015977.1	0.034084068	0.943823735	4.791348034	4.80E-30	4.31E-29
GAPT	0.476238973	2.054795962	2.109237551	1.11E-26	7.00E-26
OR2A4	0.011538265	1.428506527	6.951937514	3.52E-35	8.01E-34
BDNF	0.143576146	0.633485553	2.141495643	5.45E-13	1.26E-12
KCNE4	2.292917635	9.532058827	2.055603323	2.47E-28	1.82E-27
LINC00685	0.452585078	1.358990851	1.586274821	2.52E-13	5.94E-13
AL031058.1	1.43643786	0.615755112	-1.222066979	8.68E-28	6.01E-27
IL10RB	9.550411444	19.31456255	1.01605421	2.80E-34	5.10E-33
AC018553.1	0.626430707	3.968911411	2.663516523	1.57E-32	2.06E-31
AC008115.3	0.325154265	1.159394281	1.834175022	5.75E-24	2.88E-23
LDHC	0.570020052	0.208934469	-1.447962146	1.40E-13	3.35E-13
KLRG2	5.777790892	0.161630276	-5.159748619	6.74E-40	9.01E-38
AL513190.1	0.110530533	0.331572858	1.584880959	5.47E-14	1.34E-13
GTF2IP13	0.624502972	1.5948146	1.352608376	1.79E-16	5.18E-16
HSPB8	19.22729801	86.76589821	2.173972091	4.14E-34	7.36E-33
PHEX	0.158928018	0.389262882	1.292371299	2.98E-08	5.14E-08
AL513329.1	0.058947871	0.24972618	2.082835473	1.45E-15	3.94E-15
MELK	0.259265315	1.328792969	2.357615229	1.74E-34	3.34E-33
CHI3L2	0.442306416	2.096467856	2.244842634	8.60E-16	2.37E-15
AC084824.3	0.189594878	0.532344545	1.4894403	3.43E-18	1.11E-17
AP000553.2	0.095149677	0.420388802	2.143453576	2.72E-10	5.31E-10
IGKV3D-11	0.44319516	1.917625878	2.113307251	7.49E-05	0.000103913
APLNR	7.656364378	20.39366017	1.413389331	1.84E-12	4.12E-12
TMCC2	1.490492729	0.710378529	-1.069129456	9.39E-19	3.16E-18
AL117381.1	0.074774261	0.503164811	2.750417376	2.70E-14	6.77E-14
AC008759.3	0.320423252	0.797216234	1.314992246	1.29E-11	2.73E-11
AL138826.1	12.45973602	4.761008831	-1.387934293	1.99E-06	3.06E-06
CYP21A1P	0.265406118	1.358733308	2.355988781	2.23E-20	8.43E-20
MIR3939	0.170483807	0.517949658	1.603177171	1.36E-05	1.98E-05

Table S1. The differentially expressed genes(DEGs) were screened out in TCGA

ID	conMean	treatMean	logFC	pValue	FDR
KIAA1324	0.127750022	0.454819651	1.831971046	3.03E-17	9.21E-17
CD300C	0.445935792	2.184710382	2.292534136	1.01E-31	1.14E-30
Z98751.2	0.054203376	1.249112384	4.526376771	9.78E-28	6.74E-27
AC147067.2	0.238913819	1.252577816	2.390338023	4.78E-19	1.64E-18
HIST2H2BC	0.604960483	1.839094015	1.60408242	9.70E-27	6.13E-26
FCGR2B	0.396042087	1.586283661	2.001925122	8.50E-24	4.18E-23
PNCK	0.692857538	24.79488998	5.16134027	1.34E-37	6.75E-36
AC073115.1	0.0405357	2.249831888	5.794480338	3.97E-32	4.81E-31
AC008537.3	0.248763385	0.501302878	1.010908362	5.52E-11	1.12E-10
CLEC9A	0.242871092	0.68352274	1.492798556	8.56E-13	1.95E-12
SHROOM3	7.386548546	1.997940346	-1.886386897	3.57E-35	8.11E-34
RN7SL608P	0.362101949	1.33118521	1.878243463	1.63E-16	4.72E-16
RNU6-90P	0.062784584	0.347213339	2.467340091	5.96E-11	1.21E-10
CCND1	36.32078097	183.1363816	2.334051294	5.57E-34	9.60E-33
CTD-2297D1(	0.956468354	0.243550613	-1.97349562	1.16E-27	7.98E-27
RAB15	5.731899764	2.799890486	-1.033642982	9.92E-29	7.63E-28
CATSPER2	0.194370723	0.448502123	1.206303888	3.73E-09	6.80E-09
IGHV1-24	5.155594012	33.68942038	2.708085042	0.0007009	0.000910246
ZNF189	8.239923389	16.84151373	1.031318986	3.31E-11	6.83E-11
MSC-AS1	1.220437258	3.699326263	1.599864417	5.20E-25	2.84E-24
BBC3	1.081136615	3.449776983	1.673954262	1.24E-34	2.47E-33
AC073052.1	0.086026184	0.230901671	1.424430859	1.23E-06	1.91E-06
AC026992.2	1.618297154	0.567803464	-1.511012986	8.47E-26	4.95E-25
PITPNC1	3.452344069	6.959672922	1.011443252	1.27E-22	5.71E-22
CCSER1	1.167511879	0.218842165	-2.41547459	2.54E-39	2.57E-37
AC133919.2	0.076362801	0.228109528	1.578784793	0.00568634	0.00690966
AL138847.2	1.412018289	0.397092841	-1.830210517	8.66E-26	5.06E-25
MALL	0.49131175	1.589124379	1.6935214	1.39E-20	5.34E-20
MIRLET7BH(	0.171123053	0.448607394	1.390419277	3.96E-10	7.65E-10
AL157838.1	0.225540769	0.557560922	1.305741211	2.69E-11	5.58E-11
C4B	3.488472213	11.63988239	1.738409234	1.01E-19	3.67E-19
AGER	0.735303564	3.212644635	2.127349524	5.81E-26	3.44E-25
PCDHGB2	0.412111081	1.312982891	1.671742958	0.003480266	0.004299533
AL354733.3	0.578836821	1.504160766	1.377730168	1.09E-12	2.46E-12
AC066613.1	0.195761845	0.597887761	1.610775071	3.26E-17	9.87E-17
AC011472.2	0.118748808	0.401684633	1.758150244	6.77E-24	3.35E-23
SLFN13	1.118439989	5.096339485	2.187973535	1.02E-36	3.71E-35
CXCL12	37.1814979	16.05792324	-1.21129957	6.88E-22	2.92E-21
TMEM140	19.64592444	52.5895233	1.420545364	5.76E-35	1.24E-33
MCM5	3.383897639	7.062441181	1.061481019	7.06E-32	8.21E-31
KCNJ1	103.415914	1.338793899	-6.271380518	4.86E-41	1.27E-38
DIRAS2	0.909225106	7.364273278	3.017833739	7.44E-29	5.81E-28
RNU7-45P	0.457226825	2.16802047	2.245396426	1.05E-11	2.25E-11
ATP2A1	0.081020136	0.436369754	2.429198701	5.20E-21	2.06E-20
HOXB5	7.3413065	1.866719336	-1.975531803	1.75E-32	2.27E-31
RN7SL473P	0.070248248	0.39675855	2.497727161	5.06E-09	9.16E-09
CCR10	0.194860055	0.623353335	1.677611777	1.94E-22	8.57E-22
AC010973.1	0.092602892	0.22571864	1.28539641	2.14E-06	3.28E-06
RN7SL277P	0.012010486	0.289828269	4.592831938	4.23E-08	7.22E-08
SCARNA13	0.393948544	11.2979006	4.841903701	7.21E-09	1.29E-08
FOLR3	3.915703364	0.365672528	-3.420647327	2.43E-37	1.12E-35
AC016355.1	1.293010511	0.589404338	-1.133404419	1.21E-22	5.44E-22
FCN1	1.199971981	2.567401155	1.097308015	1.68E-11	3.53E-11
AC006441.4	1.586704021	0.082427006	-4.26677214	1.17E-14	2.99E-14
ZDHHC8P1	0.836111299	0.409034214	-1.031473477	4.23E-12	9.24E-12

Table S1. The differentially expressed genes(DEGs) were screened out in TCGA

ID	conMean	treatMean	logFC	pValue	FDR
SMPD5	0.083065513	0.236910553	1.512020932	2.63E-22	1.15E-21
TEN1-CDK3	0.108263607	0.451365744	2.059748567	7.41E-24	3.66E-23
SCNN1A	70.01062319	8.597278019	-3.025621983	1.81E-37	8.73E-36
OR2AT1P	0.004534392	0.251415915	5.793023197	1.04E-08	1.84E-08
LRRC75B	4.068962664	11.58559773	1.509599531	1.71E-22	7.59E-22
SLC22A7	11.08029853	1.266998231	-3.128510336	1.26E-15	3.45E-15
LINC00944	0.07658033	1.148177627	3.906228156	2.46E-32	3.12E-31
RNU6-796P	0.206706668	3.148393893	3.928959213	2.83E-31	2.99E-30
AC132812.1	1.430879772	5.73534493	2.002977797	1.44E-22	6.41E-22
MOXD1	14.11447025	2.802109149	-2.332589924	4.43E-28	3.18E-27
TRBJ2-3	0.189229992	1.843410977	3.284165078	8.53E-25	4.57E-24
EPB41L5	11.42652749	3.443815509	-1.730307274	6.42E-41	1.54E-38
AP000907.2	0.099392701	0.406925167	2.033551696	2.52E-13	5.94E-13
TMEM44	1.504340865	5.175391077	1.782536382	6.83E-31	6.83E-30
LINC02036	0.082439276	0.476345565	2.530604813	1.06E-21	4.41E-21
GFAP	0.145218033	0.371593389	1.355504216	0.035658299	0.040710534
AC060766.4	1.104377467	2.472203598	1.162564204	9.15E-23	4.15E-22
AC068282.1	0.367757645	0.837085792	1.186620156	2.38E-10	4.67E-10
LINC00882	0.706365326	0.269856108	-1.388224181	2.74E-13	6.44E-13
IGKV1D-17	0.20194485	0.553245967	1.453959673	0.001886806	0.002375875
SYP	1.469080383	0.496358527	-1.565458855	3.84E-37	1.64E-35
INSL3	0.166406688	0.447177734	1.426134936	5.98E-11	1.21E-10
POU5F1P3	0.05484929	0.344005176	2.648885415	2.28E-26	1.40E-25
HSPA7	0.288952251	4.601849173	3.993310684	6.18E-35	1.33E-33
RPL23AP32	0.140070192	0.669752515	2.257478118	4.44E-09	8.05E-09
ANKRD36	0.146064745	0.519656781	1.830951077	1.11E-19	4.01E-19
AL096701.3	0.087898713	0.305176833	1.795731505	2.57E-16	7.35E-16
MIR7856	0.324548774	0.777467354	1.260346792	0.000302773	0.000403306
HIST1H2BH	0.137485524	0.841267551	2.613284973	6.63E-13	1.52E-12
C1R	27.82714464	57.15088376	1.038282928	1.52E-05	2.21E-05
RPL10P9	4.381459824	15.3122633	1.805204007	1.24E-12	2.79E-12
OAS1	4.888371375	13.44938515	1.460114423	8.67E-30	7.50E-29
AC040160.1	0.11782695	0.513147147	2.122703027	1.99E-33	3.06E-32
AC011290.2	0.191914794	0.68217528	1.829676549	1.51E-18	5.01E-18
UNC93A	0.87529782	0.387584995	-1.175261257	1.75E-14	4.43E-14
AP001628.1	0.081387428	0.347057296	2.092295996	1.03E-11	2.19E-11
AL358216.1	0.096081257	0.499237269	2.377398713	3.58E-08	6.14E-08
GLDN	0.203987774	0.7916184	1.95632246	0.03100904	0.035554361
SIGLEC12	0.06434534	0.395776131	2.620777027	1.16E-17	3.62E-17
CCDC113	1.725929068	0.736996347	-1.2276438	6.46E-22	2.75E-21
RASL12	2.92217495	6.975140349	1.255179692	1.98E-20	7.53E-20
TRIB3	2.151184735	18.04057482	3.06804198	9.85E-36	2.61E-34
BMP1	2.151834175	7.837272629	1.864784779	2.28E-33	3.48E-32
AC012435.2	0.125944949	0.272725323	1.114655396	1.14E-08	2.02E-08
IGKV3D-20	1.856870892	6.156037195	1.72912844	0.000420348	0.000554184
AC025884.1	0.148498748	0.501695188	1.756360331	1.03E-12	2.35E-12
AC004832.4	0.091147509	0.419442037	2.202196315	2.73E-19	9.57E-19
CLASRP	2.541471667	5.93973332	1.224734012	5.51E-24	2.76E-23
TRBJ2-4	0.030405794	0.38169232	3.649991968	8.92E-13	2.03E-12
GAL3ST4	0.732551153	5.532738686	2.916992377	1.95E-38	1.32E-36
IGHV3OR16-[illegible]	0.103796313	0.492636136	2.246767254	9.77E-05	0.000134353
TLL1	0.834454313	2.536952502	1.604191541	1.12E-14	2.87E-14
PREX1	5.333059722	14.09112526	1.401751435	4.91E-29	3.92E-28
CGAS	0.579969432	1.547722707	1.416098251	7.09E-29	5.56E-28
LRRK2	6.140615806	13.11213786	1.094447681	9.90E-13	2.25E-12

Table S1. The differentially expressed genes(DEGs) were screened out in TCGA

ID	conMean	treatMean	logFC	pValue	FDR
E2F8	0.086575633	0.531694488	2.618564581	2.29E-33	3.49E-32
GM2A	10.97664703	28.08748678	1.35549011	5.97E-36	1.67E-34
GUCA2B	0.057989515	3.516992214	5.922406262	8.30E-23	3.77E-22
TMIGD2	0.099493346	0.454141846	2.190471026	5.03E-29	4.00E-28
BCL11A	0.160436965	0.475625296	1.567818863	1.73E-15	4.68E-15
ATG16L2	0.670101101	3.480230744	2.376732278	5.17E-32	6.14E-31
GBP5	0.402860415	4.777350086	3.567858643	7.89E-34	1.32E-32
AGBL2	0.187472221	0.540246643	1.526941362	2.65E-18	8.63E-18
GGTA1P	1.365545429	5.234507678	1.938576543	8.28E-32	9.45E-31
GNB3	0.086716699	0.239588608	1.466177562	0.004864243	0.005941053
AC099687.1	0.136221711	0.27556808	1.016452124	0.001397868	0.001779686
HNRNPA1P2'	0.045625098	0.228187521	2.322320347	5.17E-19	1.77E-18
RAD54L	0.174777705	0.998978966	2.514933138	5.97E-34	1.02E-32
LPIN3	3.327830181	9.995920641	1.586757633	1.88E-23	9.02E-23
AC015987.1	0.119984786	0.292664956	1.28639852	8.83E-07	1.39E-06
MCHR1	0.087334375	4.565749915	5.708158423	1.85E-25	1.05E-24
SPIB	0.110725189	0.28049537	1.340993502	2.77E-11	5.74E-11
RNU6-953P	0.018421721	0.49044612	4.734614911	2.99E-14	7.48E-14
ESM1	8.264372046	103.5572073	3.647378951	2.79E-37	1.26E-35
IGLC3	27.01878303	171.6655449	2.667565904	1.29E-08	2.28E-08
RNU6-595P	0.160492825	0.488815254	1.606780508	4.85E-06	7.27E-06
EGFR-AS1	0.162631198	5.553533335	5.093730003	3.10E-27	2.05E-26
PCK2	50.91182556	10.95643502	-2.216222344	1.11E-09	2.09E-09
EIF1P6	0.189538861	0.684150464	1.851819979	1.98E-10	3.91E-10
AC092834.1	0.242454634	0.890098725	1.87625053	1.01E-07	1.69E-07
AP4B1-AS1	0.070945853	0.245309291	1.789811615	3.36E-15	8.91E-15
EPB41L4B	3.736502288	0.31595678	-3.563889277	1.09E-38	8.09E-37
APOBEC3H	0.215014276	1.599652516	2.895254191	3.68E-36	1.08E-34
RALYL	3.662808025	0.072170966	-5.665387711	5.02E-42	2.03E-39
NDUFB8P2	0.094187408	0.230861562	1.293421881	7.21E-09	1.29E-08
PAH	37.24339022	5.445535652	-2.773838534	5.88E-12	1.27E-11
GGH	14.24268686	5.517472686	-1.368141847	1.86E-15	5.04E-15
LGALS9	2.135658654	9.548851391	2.160646128	5.08E-36	1.45E-34
TRBV7-4	0.033955945	0.282215242	3.055059823	8.58E-14	2.08E-13
PCLAF	0.335500439	1.545367802	2.203563686	9.52E-35	1.94E-33
AC093525.8	0.355696656	0.906666104	1.349923938	1.83E-10	3.61E-10
NHP2P1	0.098099588	0.221249291	1.17335384	4.62E-05	6.50E-05
ACTG1P3	0.111938717	0.320383975	1.517092878	9.44E-22	3.97E-21
LBX2	0.120267261	0.506846629	2.075305285	3.60E-31	3.74E-30
AC010326.3	1.614871008	3.366571839	1.05986132	2.50E-16	7.14E-16
DLX1	0.029448873	0.269472099	3.19385156	1.34E-33	2.12E-32
RNU6-100P	0.187231295	0.58801782	1.651038281	3.91E-10	7.55E-10
JUP	94.19718042	45.92781022	-1.036315877	1.16E-30	1.12E-29
GSTO2	2.900973017	0.828437673	-1.80807181	1.97E-33	3.04E-32
GLRB	2.664312121	1.134932246	-1.231156929	8.22E-21	3.21E-20
AC010973.2	0.170749117	0.775083363	2.182473371	4.60E-24	2.31E-23
LINC02526	0.05494887	1.075609227	4.29092041	1.33E-32	1.77E-31
AL451064.2	0.097109848	0.233696517	1.266946724	1.31E-05	1.92E-05
AC100803.3	0.062379176	0.517254557	3.051738052	7.77E-16	2.15E-15
INPP5J	12.69138305	3.056462871	-2.05391435	3.99E-36	1.16E-34
PLEKHG2	1.843376396	6.261068511	1.764058205	1.22E-32	1.64E-31
RPL18P10	0.034062344	0.28931865	3.086409715	4.85E-20	1.80E-19
OLFML2A	3.795180365	25.06617713	2.72350163	7.85E-33	1.08E-31
CNTN3	1.863694217	0.564183385	-1.723929086	9.38E-34	1.54E-32
AC069549.1	0.082428281	0.460471051	2.48189915	6.82E-14	1.66E-13

Table S1. The differentially expressed genes(DEGs) were screened out in TCGA

ID	conMean	treatMean	logFC	pValue	FDR
MIR6769A	0.084377388	0.374794181	2.151170228	1.43E-08	2.52E-08
CYP4A22	8.167694856	2.459057687	-1.731823389	1.63E-07	2.69E-07
AC087854.1	0.060331085	0.225822624	1.904216597	1.55E-13	3.69E-13
SIM1	10.17692438	1.235908632	-3.041657625	5.30E-38	3.13E-36
MIR643	0.05137386	0.333085742	2.696787227	4.31E-09	7.84E-09
PLA2G16	20.04850131	42.23984463	1.075110134	4.40E-28	3.16E-27
GNA15	0.868479335	2.909155838	1.744037155	1.10E-28	8.42E-28
AP000346.2	0.40175776	1.076007716	1.42129063	2.89E-08	4.98E-08
TRPV2	1.881719088	7.448069868	1.984815335	9.86E-37	3.61E-35
AC007786.1	0.494151987	0.235284511	-1.070548493	3.71E-08	6.36E-08
IGHV3-33	4.261851907	20.08033199	2.236230754	5.44E-06	8.13E-06
SNORA16B	0.268873851	0.557573619	1.052232848	0.004859514	0.005936138
GOLGA2P5	0.582708539	1.390029895	1.254269554	8.05E-10	1.53E-09
MXRA8	9.013715097	21.06605582	1.224726469	3.10E-06	4.71E-06
MCM10	0.085175469	0.404863489	2.248925649	1.70E-34	3.27E-33
MIR29B2CH(	0.276192233	0.651519178	1.238134897	2.20E-05	3.16E-05
AL365181.2	0.130982067	1.030728596	2.9762233	2.91E-27	1.93E-26
AC112722.1	0.090853519	0.328100664	1.852524204	5.18E-15	1.36E-14
AC006159.2	1.363246646	0.514752449	-1.405095913	1.04E-06	1.64E-06
ZMIZ1-AS1	0.141731074	0.284793205	1.006758623	1.36E-06	2.11E-06
RNU6-226P	0.02020744	0.437624079	4.436733633	4.31E-18	1.38E-17
CCDC186	7.462750125	3.09973346	-1.267563215	6.80E-38	3.81E-36
IFITM10	1.040091779	2.652543968	1.350665825	6.94E-11	1.40E-10
AL391056.1	0.074862035	0.314505309	2.070778201	1.59E-10	3.15E-10
GRB14	10.79335464	4.168165002	-1.372659038	2.16E-30	2.03E-29
RPS15AP16	0.089178683	0.294800287	1.724967136	3.60E-08	6.18E-08
MMP17	0.102019445	0.408242739	2.000583072	1.12E-07	1.86E-07
CLRN3	12.60649473	36.52594955	1.534754593	2.63E-18	8.58E-18
FKBP11	1.354539188	5.232706036	1.949755081	1.28E-34	2.54E-33
RNY1P13	0.133820204	0.605588657	2.178042236	3.72E-11	7.66E-11
RPS15P5	0.167779309	0.389988864	1.216868121	2.19E-08	3.80E-08
AC015660.4	0.025210355	0.23617157	3.22774694	1.73E-26	1.07E-25
IFI27	12.47682479	33.68974983	1.433058884	2.60E-21	1.06E-20
CNIH2	0.074387138	0.288520877	1.955550609	1.10E-27	7.56E-27
SNRPN	49.15339014	20.8401164	-1.237927588	1.22E-38	9.02E-37
DLX5	0.13884758	1.46357556	3.397923289	4.90E-36	1.40E-34
NLRC3	0.686245461	1.779143488	1.374386262	8.05E-28	5.60E-27
WLS	43.93294708	16.04694347	-1.453004753	6.23E-39	5.08E-37
PYGO1	1.047223397	0.333787794	-1.64956613	3.08E-28	2.25E-27
RNU2-52P	0.087830817	0.683435523	2.960006112	1.76E-25	9.99E-25
MIR6774	0.076657988	0.497287856	2.697573171	9.90E-10	1.87E-09
AC018638.4	2.373337549	5.986953344	1.334904725	7.81E-19	2.64E-18
SNORD14E	1.042713769	3.311364663	1.667082711	0.002177275	0.002728951
INSYN1-AS1	1.006203196	0.272172674	-1.886327545	4.63E-38	2.77E-36
MIR6739	0.142965758	0.585424809	2.033814237	2.31E-10	4.52E-10
HAPLN1	0.244811734	2.782122133	3.50644114	1.39E-30	1.33E-29
TIGIT	0.162190975	1.226441911	2.918713456	5.67E-29	4.49E-28
MIR548AA2	0.197358683	1.553989385	2.977084748	1.55E-14	3.96E-14
TXNP6	0.145196381	0.323713696	1.156712914	0.001276512	0.001630122
MGARP	0.743015113	6.110280274	3.039775097	5.93E-08	1.00E-07
AL139125.2	0.044896302	0.424510114	3.24113039	2.84E-37	1.27E-35
DRICH1	0.177231052	0.38812511	1.130890378	0.000669908	0.000871041
CCDC57	0.937289574	1.955805057	1.061195839	9.16E-12	1.96E-11
PARD3B	4.884027903	1.869769577	-1.385210953	2.52E-37	1.16E-35
DGKI	0.384588485	1.220309835	1.665860021	0.007375358	0.008891718

Table S1. The differentially expressed genes(DEGs) were screened out in TCGA

ID	conMean	treatMean	logFC	pValue	FDR
RNU6-485P	1.109776618	3.058514369	1.462561742	2.94E-12	6.49E-12
TCEAL2	6.656771994	0.792506831	-3.070327479	2.69E-36	8.27E-35
IGKV1-17	4.290085233	22.96411421	2.42030292	2.85E-05	4.06E-05
RF02119	0.123521104	0.737539559	2.577962878	4.15E-05	5.85E-05
ANGPTL4	11.07169999	340.8253171	4.944083844	1.58E-36	5.32E-35
AC108673.3	1.135737843	2.338143563	1.041733652	2.45E-10	4.79E-10
SLC13A2	18.49248723	0.775387295	-4.575878374	2.76E-37	1.25E-35
AC116914.2	0.236940207	1.374503062	2.536315183	1.69E-22	7.51E-22
CARMIL3	0.182647461	0.44081546	1.271113128	1.56E-06	2.40E-06
HPCAL1	10.36381203	37.4564472	1.85365931	2.83E-31	2.99E-30
TOLLIP-AS1	2.175561569	1.055290324	-1.043747889	6.66E-16	1.85E-15
FAM78B	0.308671312	0.811937931	1.39529804	1.46E-16	4.23E-16
NOD2	0.166210027	0.927548648	2.480415529	2.52E-31	2.68E-30
CORO6	0.093570706	0.407864287	2.123960347	3.06E-11	6.34E-11
MTFR2	0.176809933	0.497462755	1.492389187	6.04E-29	4.78E-28
SOWAHD	0.537704422	1.103217165	1.036831567	1.27E-12	2.86E-12
AC010201.1	0.285731236	0.685727333	1.262976268	4.87E-07	7.78E-07
AC007728.2	0.057291831	0.301434979	2.395445492	5.75E-29	4.55E-28
RPL18A	37.21040181	79.90811203	1.102636	6.21E-30	5.49E-29
GPR171	0.414092141	1.356570128	1.711939902	2.10E-20	7.96E-20
CYP2D8P	0.034565439	0.249633869	2.852411511	2.87E-22	1.26E-21
TNFRSF25	0.642991257	2.448286157	1.928901165	1.19E-19	4.27E-19
CARD16	1.644555668	6.222953559	1.919901636	2.03E-34	3.82E-33
RNU6-757P	0.033595706	0.276248462	3.039617698	4.01E-10	7.73E-10
AC012368.1	0.659458854	1.997190781	1.5986176	2.81E-16	8.01E-16
GRIA4	0.134677913	1.78667502	3.729692075	8.53E-06	1.26E-05
SLC16A7	9.963696342	2.461580264	-2.017096272	1.74E-35	4.30E-34
KIAA1522	27.55112875	10.28176591	-1.422023355	3.53E-37	1.53E-35
PCDHB10	0.623858614	2.063701843	1.725943539	9.68E-29	7.47E-28
VTCN1	17.16196353	1.604722277	-3.418819081	5.20E-38	3.09E-36
AP001107.1	0.113967598	0.286788425	1.33136309	1.38E-16	4.01E-16
AL035661.1	29.66427933	2.555782522	-3.53688975	1.54E-36	5.24E-35
AC073476.1	0.244196016	0.519466041	1.088989776	1.08E-05	1.58E-05
ESRRB	12.01685323	0.186421239	-6.010349107	3.15E-41	9.02E-39
PSMD6-AS2	0.151897706	0.310682803	1.032342307	6.43E-06	9.56E-06
RPL21P44	0.087672888	0.288016798	1.715950279	2.71E-13	6.37E-13
F12	0.653473489	2.130439221	1.70495028	3.18E-09	5.82E-09
PARVB	2.8279185	5.831700192	1.04417601	9.82E-20	3.56E-19
RAB3C	0.099666209	0.266920395	1.421233183	5.35E-12	1.16E-11
TRIM59	0.395178513	0.8902319	1.171676694	7.43E-30	6.49E-29
HLA-W	0.17061659	0.419000128	1.296192753	5.75E-17	1.71E-16
AL122125.1	0.05372958	0.398730479	2.891625425	1.22E-22	5.48E-22
RSRP1	3.185366208	6.733553978	1.079908919	1.53E-08	2.68E-08
SH3D21	1.316743254	3.715853175	1.496719429	1.06E-23	5.19E-23
KCNIP1	1.127209279	0.451201788	-1.320910702	8.55E-28	5.94E-27
AC067945.3	0.046420783	0.295643446	2.671015532	3.57E-25	1.97E-24
AC069499.1	0.158084682	0.980618694	2.632994688	6.65E-27	4.26E-26
VWA7	4.781047306	1.634967343	-1.548064861	1.65E-36	5.50E-35
REEP6	9.289523868	1.158690633	-3.003109231	6.13E-36	1.71E-34
INHBE	0.114432542	1.186352414	3.373963352	7.23E-18	2.29E-17
SCARNA6	0.146265926	18.81642477	7.007255006	2.80E-19	9.78E-19
NR1H3	4.204288542	8.723172943	1.052991307	5.41E-30	4.81E-29
LINC02084	0.147411539	0.832738078	2.49801334	9.70E-27	6.13E-26
AC113410.1	0.212526332	0.531048694	1.321202554	5.17E-09	9.35E-09
PLOD2	13.39686915	44.16285257	1.72093748	1.73E-28	1.30E-27

Table S1. The differentially expressed genes(DEGs) were screened out in TCGA

ID	conMean	treatMean	logFC	pValue	FDR
MTFP1	2.706267047	8.233979576	1.605285663	4.44E-34	7.83E-33
SNORD116-1	0.11945121	0.447828392	1.906524534	0.01138963	0.013520712
THEMIS	0.240537162	1.168348983	2.280139563	1.55E-22	6.91E-22
IGHV3-38	0.087050101	0.509610854	2.54947813	3.37E-05	4.77E-05
HIST1H2AD	0.309245493	1.089917931	1.817395033	0.000185212	0.000250272
CCR8	0.036456592	0.258426905	2.825504683	3.29E-22	1.43E-21
AL031847.1	1.477750957	0.610162816	-1.276136987	4.73E-26	2.82E-25
AC010616.1	0.029979616	0.227607406	2.924493704	2.96E-26	1.80E-25
NRP2	1.891662182	6.209033214	1.714714178	5.06E-28	3.60E-27
CCDC14	1.422956832	3.208672999	1.173084874	4.55E-20	1.69E-19
SHISAL2A	0.125346351	0.443073601	1.821626369	6.65E-26	3.92E-25
IGHV3OR15-	0.108057597	0.650552249	2.589864426	0.000694209	0.000901842
DNAJC12	4.682203842	1.89868067	-1.302190458	1.44E-06	2.24E-06
SLC27A4	15.74103956	7.662653496	-1.038614847	1.58E-33	2.47E-32
AC004890.1	0.46476678	0.975070881	1.069000143	4.73E-13	1.10E-12
ANKRD36B	0.108212981	0.300703552	1.474468344	6.12E-15	1.60E-14
CLUL1	1.971198492	0.177300591	-3.474803806	2.37E-40	3.97E-38
RNU7-140P	0.336236857	0.988752	1.556130832	6.30E-08	1.07E-07
EYA2	2.492329667	1.022485108	-1.285415078	1.62E-18	5.36E-18
FTH1P1	0.097531	0.283504754	1.539440171	6.35E-09	1.14E-08
GVQW3	0.204888864	0.43462935	1.084944028	2.19E-12	4.87E-12
CYCSP40	0.068038035	0.393371421	2.531478763	7.02E-10	1.34E-09
TCHH	0.108706385	0.293732227	1.434064886	4.35E-09	7.90E-09
GPHN	7.626959444	3.526872305	-1.112718686	2.94E-28	2.15E-27
AC093388.1	0.616402349	1.551980007	1.332165709	3.89E-26	2.34E-25
S1PR5	0.299206805	1.603826287	2.422302997	4.29E-32	5.16E-31
FP671120.4	0.378018616	0.843376641	1.157719779	0.002796022	0.003475068
TCF19	3.333770403	8.416378659	1.336044865	5.13E-21	2.04E-20
FAM107A	17.70996376	6.201400776	-1.513895226	4.85E-34	8.44E-33
HSD17B3	0.084911115	0.735941346	3.115565469	5.09E-24	2.55E-23
CRHBP	13.7814243	0.877572875	-3.973062252	9.08E-33	1.24E-31
AC068620.2	0.194321181	0.484051145	1.31671633	4.62E-09	8.38E-09
AL355075.2	0.189104686	0.720110146	1.929032477	1.15E-13	2.77E-13
LAMC1-AS1	0.227818218	0.601305107	1.40021409	5.07E-14	1.25E-13
AC147067.1	0.54967441	1.194309481	1.119527509	9.27E-18	2.91E-17
AC015845.2	1.708304026	0.836162865	-1.030708876	1.83E-31	1.99E-30
AC026369.2	0.439149282	5.670779166	3.690763625	1.27E-37	6.41E-36
CYP1A1	0.93614846	0.110894701	-3.077546904	4.58E-28	3.28E-27
SLFN11	3.083823514	10.18932426	1.72426627	1.35E-29	1.15E-28
LAD1	24.96643559	3.950002032	-2.660064565	7.81E-38	4.29E-36
DIRAS3	4.383876575	1.240958533	-1.820752273	3.48E-33	5.13E-32
TBC1D3L	0.051281753	0.239612384	2.224184994	4.32E-14	1.07E-13
LINC02154	0.023894145	0.33760518	3.820607981	1.13E-16	3.29E-16
AP001458.1	0.07706929	0.522679203	2.761697745	8.78E-14	2.13E-13
IGHV3-73	1.720516429	20.65981033	3.585913436	0.001871801	0.002357991
RN7SL364P	0.057771776	0.525579516	3.18547231	4.03E-16	1.14E-15
C6orf223	1.300641026	15.03410088	3.530943847	1.71E-30	1.62E-29
AC058791.1	0.041256254	0.429342839	3.379445387	0.000454747	0.000597988
RPS27AP12	0.107556965	0.220663632	1.036747922	4.41E-05	6.20E-05
AC002044.1	0.077712297	0.555233319	2.836879338	4.49E-12	9.79E-12
ZDHHC20P1	0.100378211	0.420559926	2.066865243	2.12E-25	1.20E-24
AC007390.2	0.228447058	0.470540232	1.042458216	4.31E-11	8.82E-11
LINC00844	5.424967486	2.395979172	-1.178999124	3.17E-14	7.91E-14
AC068790.3	0.146629954	0.547016098	1.899403435	5.38E-21	2.13E-20
C3orf67	0.17337756	0.551913801	1.670525773	2.06E-28	1.53E-27

Table S1. The differentially expressed genes(DEGs) were screened out in TCGA

ID	conMean	treatMean	logFC	pValue	FDR
AC104843.1	0.087905748	0.223813847	1.348269898	1.03E-10	2.06E-10
IGHV3-66	1.470538824	7.11600166	2.274721973	9.28E-05	0.000127874
UGT2B27P	0.066864581	0.270220892	2.014825119	1.26E-05	1.84E-05
IGHV3-15	8.033585604	37.07454247	2.206312939	2.80E-05	3.99E-05
PPIL6	1.925452394	0.954048985	-1.013062207	3.29E-31	3.44E-30
NLRC4	0.380052118	1.354477894	1.833467669	1.00E-31	1.13E-30
ADGRE4P	0.022553551	0.416372826	4.206449401	1.01E-35	2.66E-34
AL590428.1	0.08662498	0.375642895	2.116506799	5.60E-27	3.61E-26
AFP	0.73710173	0.147390728	-2.32221797	4.05E-09	7.38E-09
BX255925.1	0.260464784	0.788156688	1.597394143	6.32E-19	2.15E-18
PCDHGB7	1.576728686	3.884412815	1.3007621	0.016941709	0.019828089
ASB9	4.991026403	2.440245805	-1.032310058	1.62E-21	6.72E-21
FASLG	0.139840728	1.632608074	3.545321997	3.64E-35	8.23E-34
IGHV3-35	0.150863298	0.560083236	1.892399377	0.000404838	0.000534386
AC125611.1	0.066061357	0.476373837	2.85021568	3.37E-11	6.96E-11
AC021549.1	0.508591118	0.244658088	-1.055739294	0.001688019	0.002135619
PLIN5	1.409475043	0.492997434	-1.515505891	2.50E-29	2.06E-28
PKD1P6-NPIP	0.151196594	0.462560815	1.613217411	2.94E-25	1.63E-24
AC078899.2	0.047805215	0.25193646	2.397820016	8.95E-09	1.60E-08
LINC01270	0.061485005	0.224580755	1.868927804	7.15E-29	5.59E-28
AC073869.1	1.653146038	4.566101295	1.465748688	1.33E-22	5.95E-22
AL359220.1	0.091878044	0.227215479	1.306269066	3.82E-12	8.38E-12
SLC35F3	0.33807858	0.786698256	1.218451773	0.042240454	0.047924968
CCDC88B	0.381502054	2.277299562	2.57756135	8.93E-37	3.37E-35
DOK3	0.498618026	3.108907502	2.640400747	5.02E-39	4.24E-37
RN7SL444P	0.044043787	0.230355425	2.386851157	1.31E-08	2.31E-08
AC006435.2	0.201304351	0.937788223	2.219883808	2.41E-15	6.47E-15
PELI2	5.099335528	2.226575202	-1.195482929	5.37E-35	1.17E-33
TAL1	0.886836775	1.828429014	1.043864116	5.62E-10	1.08E-09
LSMEM1	0.239030119	0.881811679	1.883278166	3.83E-23	1.79E-22
AC090695.1	0.089218926	0.235541307	1.400558406	1.24E-11	2.63E-11
NTS	1.858870504	0.837367851	-1.150492836	0.00104296	0.001339768
FAM198B	5.807211528	13.6407845	1.232009128	7.84E-16	2.17E-15
AP000229.1	0.783941404	0.373593158	-1.069277791	6.12E-23	2.82E-22
SNORA69	0.038124078	0.243211054	2.673434439	1.02E-10	2.05E-10
TGFB1	14.84169029	43.80108268	1.561311124	8.28E-32	9.45E-31
SH2B2	1.132430458	2.796845824	1.304378269	5.11E-29	4.06E-28
AC008737.1	0.105066975	0.532859648	2.342446315	6.62E-16	1.84E-15
AC245595.1	0.382981501	1.086455469	1.504282432	1.28E-16	3.73E-16
TPBGL	0.118207265	0.58470706	2.306395304	1.26E-13	3.04E-13
SLA2	0.277966546	2.135034307	2.941276083	1.46E-34	2.86E-33
MIR4635	0.494761926	2.55712854	2.369718293	1.17E-11	2.48E-11
CD48	1.921758003	7.55138923	1.974315405	5.84E-27	3.75E-26
LAMA2	2.915384071	1.03766252	-1.490348645	1.40E-21	5.80E-21
ATP1A3	0.105380447	0.529069591	2.327850293	8.22E-14	2.00E-13
MIR320E	0.214871935	1.545851342	2.846852624	5.78E-23	2.66E-22
PDE7A	2.547677325	5.983789267	1.231876803	4.09E-22	1.77E-21
AC104986.1	0.136306116	0.282758593	1.052720571	6.79E-06	1.01E-05
PSORS1C2	0.028610678	0.396213274	3.791651622	9.93E-31	9.75E-30
AC104076.1	0.597276423	0.189041662	-1.659694559	2.31E-18	7.57E-18
HLA-DQA1	8.914367764	42.15292427	2.241428333	2.25E-28	1.66E-27
C20orf202	0.278351811	0.714462339	1.359948495	5.44E-25	2.96E-24
AP003717.1	0.018590934	0.61040162	5.037087644	3.04E-26	1.85E-25
AC021146.1	0.048532821	0.70549143	3.861595921	6.25E-18	1.98E-17
AC090578.1	0.00251411	0.256400251	6.672205893	8.22E-10	1.56E-09

Table S1. The differentially expressed genes(DEGs) were screened out in TCGA

ID	conMean	treatMean	logFC	pValue	FDR
AL359921.1	0.201957473	0.551824854	1.450158909	2.66E-17	8.13E-17
LINC01522	0.056872193	0.311726293	2.454484503	5.99E-12	1.30E-11
SNORA63C	0.127585462	1.45115945	3.507670191	1.89E-06	2.91E-06
ACTA2-AS1	0.422263699	0.946602767	1.164614912	1.22E-09	2.30E-09
APOBEC3A	0.196572769	0.449295121	1.192599918	1.89E-12	4.21E-12
MTMR11	4.119823125	13.10169104	1.669098728	6.55E-27	4.20E-26
C6orf163	0.135513895	0.296971456	1.13188348	2.88E-09	5.29E-09
AC015660.1	0.568763003	0.215263554	-1.401723543	6.30E-20	2.31E-19
AC242376.2	0.544661729	1.473717071	1.436027174	1.49E-07	2.46E-07
AC120498.1	0.051367242	0.24354079	2.245242904	1.76E-07	2.90E-07
PHKG1	0.124915627	0.494667456	1.985505018	4.45E-30	4.00E-29
SNHG5	16.30645065	35.24465544	1.11196171	8.76E-19	2.95E-18
NTNG2	0.131784685	0.440321129	1.740373358	1.85E-15	5.01E-15
AL023653.1	0.087700191	0.540457459	2.623529174	6.89E-31	6.89E-30
AGMO	1.726082529	3.492752007	1.016862766	4.19E-08	7.15E-08
VWA1	13.89072419	34.44114168	1.310011151	8.94E-22	3.76E-21
SLC38A4	3.795252662	1.237788183	-1.61643148	7.64E-27	4.87E-26
AC136475.9	0.140316014	0.367980649	1.390950232	5.36E-06	8.01E-06
KCNE3	5.540530222	25.48950462	2.201807383	5.53E-33	7.87E-32
AP000350.6	0.104887799	0.216718207	1.046973496	0.001511412	0.00191997
MECOM	26.06859747	4.414945809	-2.561845331	2.68E-40	4.35E-38
AL355916.1	0.144584171	0.424047121	1.552314974	6.72E-12	1.45E-11
TRAV29DV5	0.162362257	1.008920985	2.635524988	2.90E-19	1.01E-18
HES4	1.831551906	9.072631117	2.308454414	7.02E-30	6.15E-29
AC079848.1	1.393802986	0.390118514	-1.83704228	3.51E-35	7.99E-34
SPINK1	11.97785933	5.791864319	-1.048270384	3.55E-15	9.43E-15
LINC01587	0.25374638	2.348123509	3.21004915	6.02E-36	1.69E-34
LINC01311	0.167618835	0.481194399	1.521435576	1.17E-14	3.00E-14
NRARP	2.5812195	8.156627695	1.659919973	3.44E-22	1.49E-21
AC093281.2	0.004856362	0.236493717	5.605782093	2.88E-21	1.17E-20
AL590094.1	0.187699391	0.898105391	2.258460783	3.48E-29	2.82E-28
AC004816.1	1.608546747	0.515132742	-1.642741719	4.64E-34	8.11E-33
SLC25A37	3.379612208	6.881191739	1.025800729	3.24E-17	9.81E-17
LURAP1L	3.888376556	8.004429092	1.041630569	1.49E-16	4.33E-16
AC012618.1	0.304502912	0.636235329	1.063104462	1.28E-07	2.13E-07
MBOAT4	0.062224595	0.334017476	2.424366738	1.36E-35	3.45E-34
ADGRE2	0.426565821	1.514130619	1.82764939	2.91E-27	1.93E-26
NLRP11	0.155951383	0.328998202	1.076983355	0.000508529	0.000666813
CYCSP24	0.0981778	0.254341126	1.373296007	4.32E-10	8.32E-10
PINLYP	1.677479965	0.822805646	-1.027671937	2.70E-23	1.28E-22
BACH2	0.484030686	0.230260188	-1.071833522	2.02E-21	8.30E-21
HIST1H4J	0.050470749	0.244289287	2.275071192	2.22E-17	6.80E-17
AC135279.1	0.053482805	0.232248698	2.118523477	2.77E-15	7.40E-15
GDF3	2.173530581	0.470366337	-2.208183676	1.58E-06	2.44E-06
TNFRSF9	0.093367998	1.623367798	4.119917946	6.71E-31	6.72E-30
UBBP1	0.413612079	0.860385185	1.056704363	3.18E-05	4.52E-05
CTXN3	12.25457444	1.280067765	-3.259028294	5.53E-26	3.28E-25
PDCD4-AS1	1.183000264	2.892163312	1.289698624	2.61E-31	2.76E-30
TNXA	0.069539539	0.531437018	2.933993321	1.92E-18	6.33E-18
FOLR2	6.466904875	17.41474502	1.429162055	1.85E-16	5.33E-16
RPS23P6	0.213341581	0.81017558	1.925069424	2.58E-07	4.19E-07
AL162742.1	0.091800562	0.246667773	1.425994344	1.63E-05	2.37E-05
MIR200CHG	2.760586858	0.476473598	-2.534506816	1.27E-41	4.16E-39
FOXA3	1.737423934	0.228240284	-2.928324466	2.19E-34	4.11E-33
TRBJ2-2	0.152181082	0.851993732	2.485053793	2.71E-14	6.78E-14

Table S1. The differentially expressed genes(DEGs) were screened out in TCGA

ID	conMean	treatMean	logFC	pValue	FDR
HSPB2	1.841623653	4.257944284	1.209178804	2.95E-16	8.39E-16
PROCR	9.636000347	28.80449262	1.579787495	2.03E-29	1.68E-28
FCRL3	0.075300144	0.560407206	2.895750971	2.37E-24	1.22E-23
AL117379.1	0.36918492	1.101465494	1.57700877	3.60E-14	8.94E-14
CDK5R1	0.419956603	0.907916837	1.112319906	1.48E-22	6.59E-22
RNA5SP122	0.242232572	0.693711523	1.517942976	2.09E-07	3.42E-07
AL355796.1	0.038769555	0.369023084	3.250714981	1.45E-12	3.25E-12
AC019197.1	2.144548209	0.331330829	-2.694329396	2.06E-35	4.98E-34
ERAP2	6.136530688	12.91520588	1.073575484	1.61E-07	2.66E-07
SYNGR1	7.219574208	2.76912741	-1.382482318	1.58E-24	8.29E-24
EDNRA	3.748537333	8.923995553	1.251362024	4.11E-18	1.32E-17
MPP7	10.34143214	1.38458039	-2.900915267	2.14E-38	1.44E-36
AP000851.2	0.576450513	0.186025476	-1.63169655	0.028759507	0.033065323
MKRN2OS	0.391666953	0.182883715	-1.098700793	3.86E-17	1.16E-16
HIST1H4K	0.116288736	0.313935711	1.432757787	2.42E-05	3.47E-05
RPL9P32	0.091462624	0.304001418	1.732823834	4.61E-13	1.07E-12
AL645608.7	0.422838843	1.821948996	2.107302755	4.11E-18	1.32E-17
ASB15	1.518333427	0.047306114	-5.004318171	7.72E-31	7.67E-30
F11	9.220758745	0.457322088	-4.333602963	4.62E-39	3.95E-37
SNORD59A	0.233576367	0.61247053	1.390746119	3.99E-06	6.01E-06
TPD52L1	7.446355847	2.146230678	-1.794729415	1.87E-32	2.42E-31
TCL6	1.450816965	0.236554236	-2.616622618	5.37E-20	1.98E-19
IGKV1-5	22.94765016	75.37975577	1.715830692	0.00030553	0.000406846
AC009318.4	0.186690033	0.440233933	1.237625446	2.14E-05	3.08E-05
UPK3BL1	0.17193814	0.363960453	1.081892094	0.012751803	0.015077769
GAS1	6.467211603	1.508430201	-2.100095874	2.50E-36	7.76E-35
AC069234.4	0.109205922	0.447202577	2.033877406	1.45E-25	8.32E-25
AC007000.3	0.085740961	0.309708885	1.852856272	1.03E-08	1.83E-08
KCNQ1	22.97739263	6.04657698	-1.92602454	3.14E-37	1.38E-35
AC096720.1	0.13269401	0.45714294	1.784542096	1.50E-10	2.98E-10
AC018450.1	0.106235756	0.367007395	1.78853972	6.44E-19	2.19E-18
AC092123.1	0.197116847	0.471718366	1.258874687	1.88E-07	3.09E-07
CDH1	53.0348044	18.82839958	-1.494029072	5.83E-31	5.89E-30
ISM2	0.67842872	0.221270855	-1.616383808	1.46E-27	9.93E-27
AL359504.2	0.218444153	0.583593899	1.417700308	3.07E-10	5.97E-10
AC091959.2	0.022174546	0.284801162	3.682978552	5.14E-15	1.35E-14
PITX1	0.054145341	0.472970545	3.126841237	7.64E-06	1.13E-05
IGHV3-47	0.125190355	0.443708721	1.825489495	5.84E-06	8.71E-06
ARHGAP25	1.824978474	6.30563266	1.78876168	9.52E-35	1.94E-33
MMP9	0.839206088	13.7903335	4.038488393	2.51E-25	1.40E-24
NID1	16.64737272	43.57029422	1.388050345	5.35E-23	2.47E-22
C1QTNF7	1.878741599	0.636098711	-1.562444084	3.57E-30	3.26E-29
SLC12A9-AS	0.246444155	0.724152224	1.555032235	1.68E-18	5.56E-18
AL031670.1	0.274296832	0.710435169	1.372965043	1.41E-11	2.97E-11
INTS6L	0.546731767	1.918682509	1.811210896	8.93E-33	1.22E-31
STARD9	0.373205973	1.107848869	1.569717104	2.62E-24	1.35E-23
AC023669.2	0.277617916	1.903775793	2.777691001	0.000737398	0.000956319
TSPAN6	27.33934792	11.27682915	-1.277617369	6.74E-37	2.61E-35
Z99129.1	0.13418389	0.357815735	1.415005355	1.65E-06	2.55E-06
AL354760.1	0.153249355	0.376223902	1.295710506	2.75E-11	5.70E-11
AC018638.6	0.134719301	0.31382091	1.219984929	0.000306772	0.000408474
HECW2	1.392989002	3.988837624	1.517784529	4.34E-18	1.39E-17
TREH	4.600384207	0.972181992	-2.242456039	2.55E-18	8.33E-18
GDPD5	0.506761117	1.322098542	1.383451975	8.00E-27	5.09E-26
DWORF	1.079489333	0.23901996	-2.175145981	0.002332512	0.002917305

Table S1. The differentially expressed genes(DEGs) were screened out in TCGA

ID	conMean	treatMean	logFC	pValue	FDR
SH3BP5-AS1	0.638036868	1.65920607	1.378781382	2.75E-13	6.47E-13
AC012254.5	0.124196448	0.470878556	1.92273111	4.75E-13	1.11E-12
SAP30L-AS1	0.154534974	0.361450494	1.225864678	5.90E-11	1.20E-10
PRRG2	7.308763661	0.954691029	-2.936521576	3.22E-38	2.05E-36
FABP7	1.387057525	84.19427879	5.923622676	5.27E-37	2.14E-35
AC026304.1	0.829610072	0.375193743	-1.14479764	7.20E-23	3.29E-22
PF4	0.194015017	0.417496776	1.105596737	0.000247616	0.000331853
ESRP2	6.509789903	2.227626022	-1.547103932	2.08E-35	5.01E-34
NEU4	0.574721458	0.186410772	-1.624377687	2.46E-21	1.01E-20
RPS2P46	2.798286172	7.069824541	1.337130901	4.70E-22	2.02E-21
UBBP5	0.130713044	0.396349174	1.600368847	2.05E-09	3.80E-09
FAM222A	5.420689728	0.751000875	-2.851589937	3.79E-39	3.43E-37
HCP5B	0.073319582	0.22414191	1.612141958	7.93E-08	1.33E-07
FKBP10	7.593420875	51.42166518	2.759554451	9.08E-33	1.24E-31
CKAP2L	0.160201086	0.623318241	1.960085006	5.26E-32	6.24E-31
CENPF	0.358114201	1.24944129	1.802791479	2.55E-30	2.37E-29
FXYD5	8.884865472	29.48373252	1.730497338	2.20E-30	2.06E-29
PACRG	3.786141279	1.598265931	-1.244220772	4.65E-36	1.34E-34
SCOCP1	0.11129943	0.230743545	1.051844081	3.27E-05	4.64E-05
AC104564.3	0.177312788	0.780916822	2.138872305	7.16E-18	2.26E-17
TNFSF15	1.742586521	0.749936981	-1.216389015	2.42E-24	1.25E-23
MAP4K1	0.514946375	2.758239705	2.421253734	7.55E-32	8.72E-31
PGM5-AS1	0.803920373	0.291178654	-1.465148016	2.52E-22	1.11E-21
SLC6A12	24.70419853	10.77733745	-1.196755448	4.05E-06	6.11E-06
CDH9	0.730981499	0.17186065	-2.088595639	1.93E-15	5.21E-15
NCF1B	0.169723001	0.818902808	2.270510142	1.01E-30	9.95E-30
TRADD	5.898320153	16.3220214	1.468443702	2.18E-36	6.93E-35
TRGV5	0.128248552	0.435064862	1.762287971	4.56E-15	1.20E-14
SELENBP1	34.06001181	10.23236685	-1.734939042	7.53E-36	2.06E-34
KRT81	0.036959353	0.422861431	3.516173567	2.80E-28	2.05E-27
DRD4	0.160799212	0.651045903	2.01749893	1.40E-23	6.78E-23
DIRAS1	2.707172581	0.517510289	-2.387127409	1.41E-34	2.77E-33
AL603756.1	0.103969939	0.264940797	1.349503552	1.75E-08	3.07E-08
CORO7	0.567474408	1.642614053	1.533366309	1.24E-36	4.35E-35
AC021087.3	0.123776637	1.83910556	3.893193352	1.72E-07	2.83E-07
PNMA8A	13.29497021	3.630071438	-1.872810699	1.24E-32	1.67E-31
CA9	2.243136175	136.0519951	5.922497097	5.35E-38	3.16E-36
AC015961.2	0.192925075	0.497627079	1.367024326	1.05E-09	1.98E-09
GPR176	2.462149375	6.736636576	1.452110181	7.12E-26	4.19E-25
ADGRF1	10.86227787	0.589864301	-4.202799767	1.44E-38	1.01E-36
VWA5A	9.1151085	4.026091521	-1.178879861	2.27E-29	1.88E-28
AC114803.1	0.088562714	2.628324025	4.891299902	3.59E-37	1.55E-35
AL162411.1	1.294092243	0.621019402	-1.059230209	1.99E-20	7.57E-20
RNU6-510P	0.626875832	1.270246842	1.018857262	0.000336516	0.000446606
RPS7P3	0.073770041	0.239179528	1.696986958	6.83E-14	1.67E-13
AC090948.3	0.271798197	0.596721784	1.134522561	3.30E-07	5.33E-07
SHC2	2.000505413	4.997053687	1.320713186	1.94E-17	5.96E-17
RPL13	76.12893514	162.5812871	1.094644411	5.45E-29	4.32E-28
ZNF710-AS1	14.8738353	5.864820571	-1.342617827	6.37E-24	3.17E-23
HLA-E	232.7418903	492.7907821	1.082244371	6.66E-31	6.67E-30
CCL4	1.353069597	8.732162588	2.690102944	1.84E-32	2.39E-31
AL132639.2	0.952499796	0.368891673	-1.368521558	1.36E-28	1.04E-27
LIPH	9.909148458	0.766007959	-3.693329798	9.04E-39	6.89E-37
SSTR5	1.622891918	0.109789717	-3.88575208	4.63E-32	5.53E-31
MICALL2	0.797501278	3.332508266	2.063049718	6.45E-33	9.06E-32

Table S1. The differentially expressed genes(DEGs) were screened out in TCGA

ID	conMean	treatMean	logFC	pValue	FDR
FDCSP	0.06956089	8.940201974	7.00588722	7.68E-05	0.000106372
PNPLA3	1.083143219	0.381052559	-1.507162107	0.002809059	0.00349106
AL137024.1	0.099194831	0.247691933	1.320210039	0.000603152	0.000786676
AC093788.1	0.138315563	0.584969991	2.080399126	3.62E-19	1.25E-18
AC016705.2	3.459214514	1.275122663	-1.439808445	7.08E-27	4.52E-26
DNAJC6	3.658590569	1.395100298	-1.390919128	1.18E-29	1.01E-28
SNORA5C	0.569443038	2.315415503	2.02364767	2.73E-20	1.03E-19
HIST1H2BF	0.087292578	0.700054641	3.003536632	4.53E-08	7.73E-08
AC137834.2	0.010499838	0.46886002	5.48071834	1.70E-37	8.24E-36
SHANK3	4.975534	12.22530062	1.296946664	1.31E-19	4.69E-19
LILRA2	0.253274353	0.957236218	1.918173988	1.90E-28	1.42E-27
DTX1	5.941631389	1.060120694	-2.48663058	7.74E-36	2.10E-34
ZFHX2-AS1	0.067244723	0.255484777	1.925744366	4.35E-16	1.22E-15
PLG	29.10647671	3.352723678	-3.117934724	2.10E-18	6.90E-18
AC009090.1	0.253848084	0.813872623	1.680837652	3.89E-14	9.65E-14
MIR4477B	0.507853347	1.751291947	1.785935751	2.97E-19	1.03E-18
FOXCUT	3.003648866	0.225931326	-3.732759946	4.94E-40	7.29E-38
AC011472.5	0.130876388	0.438008902	1.742755353	3.58E-10	6.93E-10
MTCP1	0.330582161	3.209401257	3.279223393	1.25E-39	1.47E-37
AP001094.1	0.299947793	0.66963881	1.158671729	3.12E-08	5.38E-08
THOC6	6.824269972	14.37514652	1.074830034	2.26E-32	2.87E-31
HMGCS2	108.9225706	17.51671484	-2.6364988	2.58E-31	2.74E-30
LINC01252	0.097695051	0.25132915	1.363220616	6.12E-08	1.03E-07
SNORD7	0.223123361	0.73174902	1.713507336	1.37E-08	2.42E-08
AL034399.2	0.010136973	0.898284903	6.469474247	5.25E-35	1.15E-33
BTN2A2	1.998506625	4.647615228	1.217568279	6.42E-30	5.66E-29
RPL9P7	0.327324488	0.732398103	1.161906516	2.88E-15	7.68E-15
TRBV15	0.047038241	0.521969299	3.472058939	3.47E-18	1.12E-17
CDH13	3.096009276	9.338292442	1.592748975	1.73E-21	7.13E-21
SCRN1	29.37056186	14.17747243	-1.050770514	7.20E-23	3.29E-22
AL590764.1	0.153930179	0.753471698	2.291277215	3.26E-31	3.42E-30
AC026368.1	0.106269404	0.230563843	1.117439996	0.000541861	0.000709433
AC008267.3	0.293571544	0.947909182	1.691036707	2.29E-17	7.00E-17
NME8	0.041484689	0.295523026	2.832619658	1.01E-34	2.05E-33
IGSF21	0.346311228	1.041709338	1.588811719	2.06E-20	7.81E-20
TRBV21-1	0.02927303	0.257227868	3.135402971	9.27E-13	2.11E-12
RPL12P12	0.220631822	0.477125509	1.112727933	4.95E-17	1.48E-16
WNK4	19.53498876	1.153112157	-4.082455678	1.26E-40	2.53E-38
C14orf132	3.878621611	1.421125187	-1.448510389	6.14E-24	3.06E-23
AL022328.1	0.488094669	1.278296115	1.388989173	3.64E-15	9.64E-15
ZNF276	1.012265892	2.546885825	1.331145994	3.04E-24	1.55E-23
HPSE2	0.909455705	0.169838211	-2.420842295	3.26E-26	1.98E-25
AC115618.3	4.138494569	10.00917151	1.274144593	3.16E-34	5.74E-33
PLOD3	8.622099708	22.42286348	1.378859376	2.37E-37	1.10E-35
PAQR6	0.238329471	1.478289197	2.632899262	4.06E-22	1.76E-21
AC116351.1	0.526054251	1.512762039	1.523901571	0.000264044	0.000353226
AC100778.2	0.152945383	0.423324776	1.468748375	1.10E-10	2.19E-10
AP002812.1	0.076203802	0.397663656	2.383613828	2.56E-18	8.35E-18
SRSF9P1	0.130479737	0.380115444	1.542611862	1.66E-19	5.93E-19
MIR186	1.394609789	3.311316781	1.247543521	4.40E-07	7.05E-07
IL21R	0.180143879	1.136034379	2.656784954	2.61E-32	3.28E-31
CCDC154	0.074344323	0.372737856	2.325866862	1.93E-12	4.31E-12
MT1F	47.98363982	10.68370914	-2.167129993	4.46E-20	1.66E-19
PKD2L1	0.03692825	0.350477956	3.246526889	2.87E-26	1.75E-25
AC024580.1	0.271210764	0.601097275	1.14818404	3.57E-11	7.36E-11

Table S1. The differentially expressed genes(DEGs) were screened out in TCGA

ID	conMean	treatMean	logFC	pValue	FDR
ZHX1-C8orf7(	0.183201485	0.376193149	1.038042378	4.09E-22	1.77E-21
AP001793.1	0.144976211	0.344154296	1.247239326	8.56E-11	1.72E-10
IGLV5-37	0.266608137	0.842445289	1.659862183	0.004241025	0.00520223
DCXR	64.1071605	14.67242986	-2.127377698	6.79E-33	9.49E-32
AL731571.1	0.207636123	0.638208813	1.619971076	1.56E-21	6.45E-21
CHKB-CPT1E	0.072762262	0.246321006	1.759277361	1.73E-11	3.65E-11
AC079466.1	0.079746909	6.708832791	6.394489381	1.30E-25	7.49E-25
PCCB	12.71099814	3.964180799	-1.680982655	7.18E-37	2.77E-35
C22orf34	0.182433978	0.616539348	1.756818517	3.48E-27	2.29E-26
MOGAT3	0.397957991	1.956139507	2.297321212	1.01E-11	2.16E-11
WNT7B	3.936383163	0.245372546	-4.003824914	6.86E-27	4.39E-26
SNORD17	1.085952229	72.1082438	6.05313166	1.56E-09	2.92E-09
AC018868.1	0.194105699	0.463105292	1.254497769	1.12E-14	2.87E-14
PLAC8L1	0.085234942	0.547450151	2.683210717	2.32E-28	1.71E-27
TMSB4Y	1.793387248	0.580927978	-1.626255825	3.33E-10	6.46E-10
RNU6-945P	0.183928468	0.422648164	1.200312387	9.83E-07	1.54E-06
REG1B	0.037606154	0.94952685	4.658168127	2.35E-10	4.60E-10
TRAV25	0.071733493	0.478806804	2.738724876	1.34E-16	3.89E-16
EHBP1L1	3.579166972	9.832540615	1.457940392	7.60E-31	7.57E-30
AC025171.3	0.089514831	0.295542209	1.723165548	1.28E-18	4.26E-18
SLC29A2	7.048646268	1.796930508	-1.971811589	1.01E-32	1.37E-31
AC005306.1	0.060826949	0.360572816	2.567508089	2.68E-18	8.72E-18
AGT	23.32483383	50.60252513	1.117342576	9.34E-09	1.66E-08
AC092118.2	0.199200985	0.560169679	1.491639116	1.05E-09	1.97E-09
AC087045.3	4.052397141	0.087205346	-5.538215175	1.77E-41	5.35E-39
BTBD19	0.959842457	3.711220214	1.951024075	6.71E-29	5.27E-28
MIR4435-2H(	1.056913754	4.043424605	1.935720055	5.52E-37	2.23E-35
CALCRL	10.04338083	20.5130162	1.030294643	7.70E-12	1.66E-11
SLCO4A1-AS	0.119330044	0.501380484	2.070948521	4.42E-05	6.23E-05
PLCG1-AS1	0.094879833	0.335665901	1.822852613	2.19E-22	9.66E-22
CADPS2	17.98879646	6.013922565	-1.580720469	1.01E-38	7.54E-37
LINCR-0001	0.137810995	0.504949755	1.873448843	1.54E-15	4.19E-15
ALOX15B	0.078100228	1.511289479	4.274309451	2.52E-34	4.64E-33
LINC00174	0.635956639	1.856050674	1.545235792	1.25E-17	3.88E-17
CLEC1A	1.267956868	3.094716956	1.287301796	3.39E-21	1.37E-20
SLC16A6	0.550368194	2.03508022	1.886616661	1.75E-22	7.74E-22
DOC2A	0.077140151	5.516229564	6.160056725	1.25E-37	6.32E-36
AC012254.1	0.094205753	0.304175832	1.69101846	1.23E-07	2.04E-07
GPX3	2285.433584	774.8427263	-1.56049248	5.82E-05	8.13E-05
AC007406.2	2.072276318	8.050849708	1.957924672	4.38E-18	1.41E-17
AL354989.1	0.067013994	0.218914904	1.707835875	2.68E-11	5.57E-11
LINC00941	0.083075172	0.27625519	1.733512294	0.000566667	0.000740639
SRD5A3-AS1	0.097738523	0.246246003	1.333101104	7.86E-30	6.83E-29
NEBL	13.3968191	4.789705375	-1.483881671	5.81E-32	6.87E-31
COL18A1-AS	0.056908514	0.366294149	2.686286246	0.000231323	0.000310664
LINC01426	0.747948715	4.168572739	2.478542252	7.71E-19	2.61E-18
MS4A4A	2.608754557	10.09454606	1.952142915	8.38E-27	5.32E-26
AC027607.1	0.160421123	0.489209562	1.608588486	5.02E-19	1.72E-18
AC010531.3	0.102831911	0.260090783	1.338727238	2.07E-07	3.39E-07
AC092667.1	0.042215338	0.264775802	2.648932107	7.30E-22	3.10E-21
RPLP1P6	1.121982719	2.382621246	1.086499175	2.38E-19	8.37E-19
CCR1	2.950080943	6.545957196	1.14984963	1.20E-11	2.55E-11
AC078820.1	0.086721327	0.226312053	1.38385468	0.021260062	0.024720556
AL136084.3	0.483262762	1.155168865	1.257224026	3.97E-08	6.80E-08
RIN3	1.736247782	4.692865695	1.434496321	9.64E-33	1.31E-31

Table S1. The differentially expressed genes(DEGs) were screened out in TCGA

ID	conMean	treatMean	logFC	pValue	FDR
AC025627.2	0.115567273	0.27567049	1.25421193	6.32E-05	8.81E-05
IFI44	5.393094931	16.26535588	1.592617055	5.90E-29	4.66E-28
PRTG	0.597444692	0.151856139	-1.976099934	3.70E-35	8.35E-34
TMEM178A	11.01851029	1.18546425	-3.216405123	7.12E-40	9.39E-38
PTH1R	73.50216802	8.079874382	-3.185382036	3.31E-25	1.83E-24
TNFRSF13C	0.141724726	0.325606696	1.200038895	2.02E-08	3.52E-08
AC022167.2	0.26350701	0.659988537	1.324599626	3.27E-26	1.98E-25
AC091887.1	0.039846947	0.239376263	2.586739002	5.46E-23	2.52E-22
TRAV36DV7	0.061838876	0.289964705	2.22929131	2.69E-16	7.67E-16
PKD1P6	0.2754947	0.752809118	1.450259537	9.86E-21	3.83E-20
SLC25A27	1.089425683	2.35259271	1.110683793	0.000760576	0.000985508
S1PR4	0.727896708	2.586876839	1.829405723	2.23E-29	1.85E-28
HCAR1	1.33583617	0.413246465	-1.692668698	5.60E-31	5.66E-30
PSAT1	44.95856672	7.969221281	-2.496085381	4.15E-24	2.09E-23
MGAT3	2.258322908	5.634608378	1.319063556	8.92E-09	1.59E-08
PROM1	24.19362538	10.57581297	-1.193858403	1.63E-19	5.82E-19
WDFY4	0.376538008	1.32030667	1.810005663	8.41E-23	3.82E-22
AC020951.1	0.107455066	0.233379902	1.11894682	0.006962952	0.008409703
CPNE6	1.886353055	0.022183501	-6.409968829	2.26E-15	6.06E-15
CAMTA1-DT	1.149879181	0.364071883	-1.659187053	1.48E-26	9.21E-26
MRPS6	34.24203228	10.80997837	-1.663404692	5.23E-21	2.08E-20
OGFR	6.041739389	12.50979341	1.050022105	1.29E-33	2.06E-32
TRBV27	0.081946518	0.658507945	3.006446299	6.31E-17	1.87E-16
IGLV3-16	0.238380436	3.446111105	3.853631475	0.000351268	0.000465644
AL359880.1	0.070448354	0.241110444	1.775056241	6.25E-06	9.31E-06
AC079760.2	0.11451777	4.718793375	5.364774613	4.96E-35	1.09E-33
AC068299.1	0.013518085	0.316513021	4.549302177	5.32E-33	7.60E-32
NOL3	3.572675417	38.74501045	3.43893378	9.81E-40	1.24E-37
MCEMP1	0.074701727	0.334087127	2.161010899	1.16E-17	3.61E-17
CCDC17	0.160879947	0.388786141	1.272992277	2.62E-07	4.26E-07
RPS26P8	0.119268952	0.286712543	1.265386488	0.000714109	0.000926875
AC127502.2	0.607823922	2.179395073	1.842202385	6.78E-26	3.99E-25
RPL29P24	0.099923503	0.293993226	1.556886963	9.65E-13	2.20E-12
B3GNTL1	0.288435572	0.731718047	1.343038742	3.72E-32	4.51E-31
CR936218.1	0.259280711	1.093931576	2.076935713	9.57E-22	4.02E-21
NPY1R	15.03992332	6.678034831	-1.171301688	5.71E-24	2.86E-23
RUFY4	0.008875577	0.282116978	4.990308735	8.92E-35	1.83E-33
CCDC88A	1.66092669	3.352990926	1.013460182	1.78E-24	9.28E-24
AL109936.2	0.824016286	1.775648282	1.107601086	5.19E-12	1.13E-11
ADAMTS16	3.34860144	0.546467058	-2.615352236	9.12E-31	8.97E-30
PTCH2	0.181356082	0.512816451	1.499617412	1.87E-20	7.15E-20
AL008723.1	0.090383249	0.279535169	1.628902485	0.001781734	0.002248243
FHL1	17.83232988	57.64560407	1.692715385	4.44E-20	1.65E-19
RASL11A	22.55621031	10.6515464	-1.082461803	1.83E-26	1.13E-25
AC010883.1	0.578507076	1.50989883	1.384045374	1.65E-12	3.71E-12
FERMT1	1.645701214	0.68215302	-1.270535125	1.38E-13	3.30E-13
TRAJ3	0.056552988	0.553283985	3.290345022	2.17E-09	4.02E-09
IGF2BP3	0.04052223	0.327299929	3.013827801	3.38E-17	1.02E-16
SAA4	0.030838515	0.655397765	4.409565532	8.03E-06	1.19E-05
AC022306.2	1.085303486	2.254416747	1.054655711	4.18E-20	1.55E-19
ISG20	0.41655104	2.125562903	2.351279771	2.86E-36	8.70E-35
AC093424.1	0.109950796	0.305131446	1.472572818	1.39E-08	2.45E-08
B4GALNT2	6.037468302	0.450305995	-3.74496612	3.08E-34	5.58E-33
ITGAX	0.539689098	5.246232732	3.281081361	2.83E-38	1.84E-36
ERICH4	5.257066422	0.457508877	-3.522386322	6.41E-16	1.79E-15

Table S1. The differentially expressed genes(DEGs) were screened out in TCGA

ID	conMean	treatMean	logFC	pValue	FDR
MIR17HG	0.08218488	0.229677489	1.482664555	0.000565182	0.000738745
PDE6B	1.590094147	3.186809617	1.003000646	3.49E-13	8.16E-13
RAB33A	0.35543965	1.55095771	2.125482818	4.83E-28	3.44E-27
IGKJ5	0.511897653	4.008572135	2.96916114	1.19E-06	1.85E-06
SLC13A1	19.274907	8.515903804	-1.178492341	5.28E-07	8.42E-07
AC009119.3	0.062631439	0.314896319	2.329917963	1.53E-16	4.42E-16
IGHJ3	1.827186476	10.7568777	2.557563597	1.56E-06	2.42E-06
AC103706.1	0.269835801	0.755660515	1.485656467	7.65E-18	2.41E-17
MUC3A	0.199117942	2.076072848	3.382161936	3.45E-32	4.22E-31
DGKZP1	0.179193184	0.358648188	1.001053582	8.29E-23	3.77E-22
AC004148.2	0.510152588	1.863830991	1.869270313	1.47E-17	4.56E-17
DGCR5	0.418540462	11.01395087	4.717821168	2.97E-36	8.96E-35
AC012615.6	0.180508467	0.822616013	2.188152643	7.62E-23	3.47E-22
MZT2A	3.393943589	9.956934727	1.552739087	2.35E-30	2.19E-29
AC022210.1	0.148512025	0.366649645	1.30382239	1.74E-18	5.73E-18
AL513327.1	0.253826551	1.05298526	2.052570348	1.35E-24	7.11E-24
RNA5SP425	0.036669985	0.333501968	3.185023684	1.12E-10	2.24E-10
CCNA2	0.615271843	2.401520603	1.964652309	3.98E-35	8.89E-34
TFAP2C	1.775898371	0.379051297	-2.228084016	2.65E-24	1.36E-23
CREB5	1.370992689	3.91888113	1.515220935	6.59E-25	3.56E-24
MIR503HG	1.62844239	3.264438536	1.003342196	0.004237092	0.005197714
PPARA	8.637702278	4.296827626	-1.007375689	5.64E-25	3.07E-24
GRID1	0.243957778	0.673372682	1.464773714	5.80E-12	1.26E-11
MEX3B	0.32677398	0.741375235	1.181910815	2.04E-16	5.87E-16
AC006023.2	0.075974745	0.253109062	1.736167334	0.000407948	0.000538421
PLEK	2.499327369	12.07705911	2.272657399	3.13E-30	2.87E-29
MIR4489	0.277436693	0.957139095	1.786569991	3.83E-10	7.41E-10
TCIRG1	4.765599194	13.28861379	1.479461092	1.02E-30	1.00E-29
LRRC14B	0.076099054	0.822630841	3.43429473	2.12E-06	3.26E-06
GRIK1	0.047772063	0.238876709	2.32202711	3.68E-05	5.20E-05
AL669983.1	0.06099654	0.342146574	2.487815199	1.99E-10	3.92E-10
SCN9A	0.645444863	1.735984281	1.427388122	3.48E-09	6.36E-09
AL356740.1	0.896657788	0.167994777	-2.416141103	3.00E-36	9.05E-35
AC108676.1	0.076567014	0.409667281	2.419657777	7.04E-28	4.93E-27
AC080023.1	0.246310941	0.527745956	1.099362907	5.01E-13	1.16E-12
AC079610.2	0.729658632	0.309381111	-1.237836544	2.74E-19	9.59E-19
CACNA2D4	0.299653483	0.982678717	1.713424666	4.87E-29	3.89E-28
OVOL2	1.245520458	0.1128671	-3.464051803	1.27E-38	9.33E-37
PSTPIP2	1.109967185	2.430500159	1.130736204	3.32E-20	1.24E-19
GCDH	8.152330028	3.993787816	-1.02945477	1.44E-26	9.01E-26
GZMK	1.119425467	11.76101515	3.393182213	3.73E-27	2.44E-26
AC009961.2	0.012566478	0.321483385	4.677091902	1.41E-10	2.80E-10
AP000892.2	0.140978721	0.285750376	1.019277977	3.60E-05	5.09E-05
ANKRD53	0.331531142	0.663520321	1.000996257	1.15E-11	2.46E-11
PPP1R13L	3.480957736	10.55501862	1.600372921	6.26E-30	5.53E-29
AFG3L1P	0.546364406	1.148194493	1.071431638	5.51E-12	1.20E-11
AC004846.1	0.693133468	0.314771852	-1.138826644	1.40E-29	1.19E-28
SAXO2	0.412849423	0.202788733	-1.025638191	1.25E-19	4.49E-19
BTN3A2	4.651531708	18.77493027	2.013029783	2.74E-35	6.40E-34
RNY4P25	0.114837461	0.525508829	2.194121662	7.41E-05	0.000102826
AC006378.2	0.064806349	0.224302831	1.791240758	3.92E-17	1.18E-16
AEN	2.457039503	6.439466189	1.390020046	2.01E-36	6.48E-35
CDH24	1.021140424	2.213403899	1.116085462	6.80E-23	3.12E-22
CDKN2C	2.615416444	7.829170889	1.581818853	1.87E-32	2.42E-31
RNF212B	2.278186778	0.136962508	-4.0560331	7.85E-31	7.80E-30

Table S1. The differentially expressed genes(DEGs) were screened out in TCGA

ID	conMean	treatMean	logFC	pValue	FDR
IGLV5-48	0.112185317	0.493044874	2.13583509	0.004539569	0.005554868
COL23A1	2.064496081	61.90506148	4.906195787	1.04E-34	2.10E-33
AICDA	0.012507122	0.376703428	4.912607391	8.82E-35	1.81E-33
CD36	3.561980571	16.4489814	2.207246696	2.55E-23	1.21E-22
MPP2	1.067437387	0.274902997	-1.957156906	2.54E-32	3.21E-31
CLEC18A	0.509953973	2.317215841	2.183953488	3.99E-14	9.88E-14
FOXC2-AS1	0.099378763	0.724179465	2.865337772	9.22E-13	2.10E-12
OSCAR	0.427156025	2.537181639	2.570391769	5.36E-36	1.52E-34
RNU2-11P	0.159922159	1.404027851	3.134129794	4.15E-21	1.66E-20
DCSTAMP	0.029995388	0.265781414	3.147427614	6.69E-28	4.70E-27
AC133065.3	0.952575899	2.643840798	1.472729354	5.89E-17	1.75E-16
EIF4EBP1	9.736554972	41.00997178	2.074491443	5.29E-34	9.15E-33
TBX21	0.179919735	1.026242273	2.511946014	2.06E-34	3.88E-33
OLFML2B	3.230107157	20.55591299	2.669899519	8.24E-34	1.38E-32
AC087222.1	0.103054198	0.659191437	2.677294231	1.79E-21	7.40E-21
CENPT	2.265614597	5.171412889	1.190656029	1.22E-24	6.45E-24
PCSK6	0.974560968	7.786660459	2.99818037	2.09E-28	1.55E-27
RFC2	5.968517278	12.75512434	1.095632481	1.53E-37	7.51E-36
SNORD9	0.107317568	0.847625966	2.981541516	0.000136872	0.000186617
CRIP1	0.368523833	1.357788786	1.881429245	7.15E-25	3.85E-24
RTEL1	0.120362321	0.274666435	1.190296786	1.19E-22	5.34E-22
AC068790.2	0.1267637	0.46818075	1.884923939	1.51E-18	5.01E-18
AL132712.2	0.408532998	1.083164251	1.406727514	4.95E-09	8.95E-09
AC108134.3	0.476816533	1.23918014	1.377879762	4.65E-11	9.50E-11
LINC00900	0.10549299	0.414216534	1.973238009	2.36E-25	1.33E-24
UST	3.268293092	1.337158139	-1.289367271	1.15E-28	8.81E-28
CRYBB1	0.252800557	0.704587479	1.478779195	3.86E-23	1.80E-22
GNAI1	23.59326063	10.08993414	-1.225458057	1.09E-25	6.33E-25
RPL22P19	0.024782544	0.30236259	3.608883429	1.79E-22	7.91E-22
AC010336.2	0.754044821	0.249261096	-1.596992551	1.16E-10	2.32E-10
IGHV3-11	5.841501167	21.9016924	1.906631285	7.33E-05	0.000101761
IGKV2D-40	0.591343743	2.611844381	2.143000037	0.009066488	0.010856163
AC120036.3	0.814874019	0.372008639	-1.13124091	1.27E-20	4.89E-20
RNU6-892P	0.166769624	0.569524875	1.771902325	2.53E-07	4.12E-07
TACR1	0.832519025	0.314857087	-1.402786099	2.86E-23	1.35E-22
NIM1K	0.164269292	0.476549306	1.536562677	4.31E-11	8.82E-11
PRR7	0.341700545	1.745356908	2.352717628	1.51E-30	1.44E-29
AL035404.2	0.034081686	0.261890205	2.941893497	2.94E-11	6.10E-11
AC005899.6	0.329949503	1.153752238	1.806016296	2.64E-15	7.07E-15
RNU6-1136P	0.183656647	0.560314547	1.609225833	1.18E-11	2.52E-11
AC244197.2	0.120918853	0.664160648	2.45749305	1.09E-12	2.47E-12
SEM1P1	1.400167967	0.131233199	-3.415395268	1.98E-05	2.85E-05
RERGL	5.761676472	1.992618295	-1.531823279	5.36E-25	2.92E-24
CNTD2	3.80168081	0.137763559	-4.786371182	8.87E-27	5.62E-26
AP006621.2	0.441335212	1.854161364	2.070820041	3.74E-14	9.28E-14
SLC25A10	9.414016619	4.596092748	-1.034402488	9.34E-09	1.66E-08
SLPI	166.7490311	48.27262438	-1.788401211	1.44E-18	4.78E-18
LIN37	0.663995044	1.335982335	1.008656553	4.18E-34	7.41E-33
AC141557.1	0.212571125	0.972681117	2.194021273	9.27E-17	2.72E-16
AL139351.1	0.088745039	0.63526481	2.839619728	1.69E-18	5.58E-18
C6orf52	1.326797647	0.374138231	-1.82630506	9.09E-37	3.40E-35
AL162724.2	0.207476886	1.892004637	3.188893097	9.77E-34	1.60E-32
TYRP1	5.27942938	0.141356967	-5.222967112	1.50E-39	1.70E-37
DPYSL3	6.055980132	13.39626318	1.145398247	2.11E-11	4.41E-11
AL161937.2	0.056829079	0.354454687	2.640899957	7.05E-32	8.21E-31

Table S1. The differentially expressed genes(DEGs) were screened out in TCGA

ID	conMean	treatMean	logFC	pValue	FDR
PADI2	14.23608738	2.525784916	-2.494748996	5.58E-29	4.42E-28
IFITM4P	0.111226415	0.712468944	2.679327677	5.84E-26	3.46E-25
FECH	25.27341774	6.544823334	-1.949194619	3.09E-39	2.92E-37
ADA	1.034734726	4.739863256	2.195584485	5.15E-34	8.92E-33
SNX10	13.96596378	32.87792731	1.235204215	1.47E-25	8.39E-25
ANO9	0.981751083	3.274819326	1.737986124	4.30E-10	8.29E-10
AC005154.1	0.140944599	0.438417121	1.637175946	6.72E-09	1.21E-08
RNU6-853P	0.162539635	0.392163182	1.270662536	0.00172991	0.0021862
B4GALNT4	0.09727171	0.875462438	3.169953089	3.95E-07	6.35E-07
AL365361.1	0.314556736	1.027701595	1.708029262	1.42E-20	5.48E-20
SLC9A5	0.11545852	0.402574641	1.801881665	2.65E-27	1.76E-26
GIMAP5	0.433065458	1.159739565	1.421143854	1.62E-19	5.78E-19
CRYL1	133.7763104	64.89023218	-1.043749428	9.96E-24	4.88E-23
RN7SL49P	0.085435089	0.28497207	1.737919896	2.98E-08	5.15E-08
AC087588.2	0.090828032	0.278339228	1.615634724	2.84E-06	4.32E-06
AC004069.1	0.193290274	0.751973518	1.959912811	9.13E-26	5.32E-25
RNA5SP195	0.085769956	0.29987931	1.805837699	5.96E-05	8.32E-05
HMGB3P24	0.220103711	0.509998323	1.212309032	1.81E-14	4.59E-14
PHYHD1	13.34341421	2.781405091	-2.262242076	6.44E-37	2.51E-35
ATP2C2	0.589952918	0.152278652	-1.953886121	4.48E-34	7.89E-33
NSG1	1.000402027	0.427832944	-1.225460402	1.12E-05	1.64E-05
ABHD14A	9.483436306	4.657996353	-1.025700402	7.15E-29	5.59E-28
AC087752.2	0.043375357	0.247698112	2.513635335	4.62E-15	1.22E-14
AL590644.1	0.007711228	1.502525555	7.606213181	1.46E-36	5.03E-35
VSIG4	5.905771228	18.19610974	1.623432663	1.08E-16	3.16E-16
SHC1	17.57345944	37.06370639	1.076608936	1.14E-31	1.27E-30
CRYM	8.631071331	3.93842758	-1.131919897	5.64E-07	8.98E-07
IGHG3	12.89760276	121.8557209	3.239999137	6.32E-08	1.07E-07
CCDC150	0.32424327	0.760027399	1.228974796	1.84E-09	3.42E-09
AP003110.1	0.669024443	0.187988877	-1.831411622	4.45E-10	8.56E-10
PER2	3.173807708	7.14373388	1.170463616	1.58E-22	7.05E-22
AC084018.1	0.99577225	2.827534184	1.50565675	2.43E-09	4.49E-09
AC079684.1	0.171040217	0.581492446	1.765424861	6.02E-17	1.79E-16
ENPP7P7	0.300459119	0.927455427	1.62610924	9.86E-10	1.86E-09
EPSTI1	2.863071139	6.304459988	1.138809283	1.30E-18	4.33E-18
IGLV2-34	0.226060286	0.478530828	1.081904303	0.001673113	0.002117282
MYO3B	5.068082386	0.368135252	-3.783132167	1.94E-38	1.32E-36
AC138932.5	0.194844928	0.569217787	1.546654366	1.64E-13	3.91E-13
LAPTM4B	59.73287806	28.61708752	-1.061648385	6.01E-30	5.32E-29
NPM1P25	5.999087301	1.742020769	-1.783981202	5.05E-35	1.11E-33
AC006566.1	0.174338961	0.510535798	1.550117109	3.98E-16	1.12E-15
UBA52P6	0.114347293	0.480916806	2.072365132	4.51E-29	3.61E-28
NALT1	0.153686553	0.641720519	2.061954171	2.63E-12	5.83E-12
AC025265.1	0.078608932	1.250925062	3.992158307	3.70E-34	6.63E-33
AC073651.1	0.016418257	0.411436632	4.647297396	4.39E-05	6.17E-05
AL133523.1	0.472838	0.215613069	-1.132901358	1.69E-13	4.02E-13
SCARNA10	0.214070386	20.62631466	6.590257033	0.01369097	0.016148364
GSG1L2	0.004601747	7.758228835	10.71932983	9.38E-19	3.16E-18
AC005332.5	0.789173261	1.897862519	1.265961507	5.79E-27	3.73E-26
AC009084.2	0.040651844	2.242469683	5.785623888	1.35E-36	4.71E-35
AC069120.1	1.115246096	0.178333828	-2.6447098	3.72E-27	2.44E-26
TIMM8AP1	0.252828591	0.593797339	1.231811007	0.000511519	0.000670603
AL078459.1	0.081824285	0.282804933	1.789206287	1.31E-14	3.35E-14
AC117503.4	0.127779634	0.402620444	1.655762513	3.97E-15	1.05E-14
MIR3671	0.551157319	1.961680023	1.83155366	1.03E-08	1.82E-08

Table S1. The differentially expressed genes(DEGs) were screened out in TCGA

ID	conMean	treatMean	logFC	pValue	FDR
CDHR1	0.211467849	3.991554242	4.238440375	7.71E-19	2.61E-18
AC004076.2	0.09897778	0.277680497	1.488249256	4.48E-21	1.79E-20
NAT8L	11.04399282	0.394484636	-4.807148933	1.42E-39	1.65E-37
RNU6-890P	0.099586661	0.330989262	1.732759999	0.002508242	0.00312892
PYCR1	1.066275435	3.393914528	1.670370077	2.67E-11	5.55E-11
CYP2J2	1.437270426	52.21073534	5.182943038	1.17E-30	1.13E-29
TRBJ2-2P	0.265883929	1.528393628	2.523147664	4.37E-19	1.51E-18
AC032044.1	0.070081025	0.229369918	1.710580413	1.13E-12	2.55E-12
MIR6728	0.049133807	0.5571913	3.503384803	1.34E-18	4.47E-18
BTG1P1	0.058069398	0.228322871	1.975225389	3.35E-17	1.01E-16
AC004890.3	0.049373396	0.258951777	2.390877674	2.34E-17	7.16E-17
AC027020.2	0.394557798	0.836579964	1.08426679	6.32E-06	9.41E-06
SAPCD1	0.072301114	0.596161888	3.043614375	2.29E-19	8.06E-19
HNRNPH1P1	0.118700071	0.2641168	1.153855271	1.65E-06	2.54E-06
C1orf226	5.661988175	0.800242463	-2.822799647	3.90E-39	3.51E-37
MARCKS	22.78024789	48.25415192	1.082869635	1.56E-26	9.69E-26
RNU6-1157P	0.152090689	0.636464629	2.065148508	2.24E-13	5.30E-13
IGHV1-2	11.49579373	26.4907642	1.204383382	0.000434522	0.000572394
LYPD3	1.201011788	0.480951579	-1.320286751	2.31E-25	1.30E-24
IL7	0.703906974	1.626606139	1.20840828	1.69E-24	8.84E-24
DEFB131E	0.009131719	0.276969319	4.922695937	4.91E-35	1.09E-33
DUSP26	1.479492803	0.232901021	-2.667313809	2.26E-36	7.13E-35
WASHC5-AS	0.111216664	0.285971626	1.362499038	2.05E-11	4.29E-11
PM20D1	1.996239577	0.299158411	-2.738303342	7.74E-12	1.66E-11
PCDHGC3	2.834770899	6.221170971	1.133954011	1.20E-18	3.99E-18
AL161935.1	0.041822578	0.285633522	2.771811422	2.26E-24	1.17E-23
AC100826.1	0.100752212	0.44072568	2.129069449	1.89E-06	2.91E-06
HLA-G	1.676810196	24.47187207	3.867333171	2.77E-32	3.44E-31
TENM1	0.358796028	2.105451036	2.552893502	9.48E-27	6.00E-26
ANXA4	21.65618764	78.2592556	1.853482095	1.87E-34	3.56E-33
RIOX2	4.013628333	1.700767779	-1.238720857	1.58E-35	3.93E-34
ZNF793-AS1	1.437710857	0.651347356	-1.142274534	9.76E-29	7.51E-28
ALDOC	6.832748431	32.67437267	2.257621622	4.42E-25	2.42E-24
DOCK4	2.353753925	4.745518742	1.011602299	3.51E-21	1.41E-20
AC243964.2	2.639445889	0.727702807	-1.858813809	7.81E-25	4.20E-24
AP001160.4	0.155973569	0.542680096	1.798800421	1.73E-10	3.43E-10
AC135050.3	0.160602104	1.482246647	3.206222835	1.68E-30	1.60E-29
AC002401.1	4.430612702	0.86628547	-2.354591795	2.17E-07	3.55E-07
ARMC2-AS1	0.128187831	0.328476553	1.357531086	9.19E-06	1.36E-05
SNORD89	0.673311726	4.50720654	2.742887066	4.78E-27	3.10E-26
RNF138P1	0.088036688	0.434767778	2.304068252	3.91E-13	9.13E-13
MIR126	0.357106199	2.802709243	2.972397003	2.10E-15	5.66E-15
PARM1	31.90076421	14.97769543	-1.090775328	6.51E-24	3.23E-23
AL139407.1	0.181116747	0.489115411	1.433254974	1.01E-11	2.15E-11
IGLV3-25	10.55332712	46.96425583	2.153865244	2.14E-07	3.49E-07
AC006077.2	0.363484036	0.768493069	1.080140245	8.17E-06	1.21E-05
AC122719.1	0.022557583	0.325929803	3.852876856	1.48E-16	4.30E-16
PNMA3	0.854318747	2.389100988	1.483621492	0.007743492	0.009315357
VCAN	7.009032463	25.42183894	1.858781183	6.47E-18	2.05E-17
RNU4ATAC1	0.471305239	1.631738128	1.791675918	1.10E-13	2.65E-13
IGHD	3.47977236	7.861654315	1.175839998	0.001315795	0.001678622
AC009996.1	0.121245898	0.369511548	1.607683516	2.82E-10	5.51E-10
TRAV35	0.067022355	0.318884614	2.250320207	3.01E-15	8.03E-15
TDRD6	0.080143275	0.30214995	1.914611342	1.20E-24	6.36E-24
SNHG15	1.780567522	5.903480523	1.729228631	3.30E-34	5.97E-33

Table S1. The differentially expressed genes(DEGs) were screened out in TCGA

ID	conMean	treatMean	logFC	pValue	FDR
HTR6	0.030121315	1.066320661	5.145712768	2.31E-30	2.16E-29
CALML3	3.394824496	0.073922323	-5.521183095	3.63E-16	1.03E-15
MELTF-AS1	0.693801615	1.426135092	1.039515546	2.13E-07	3.48E-07
AC091729.2	0.093200067	0.495761222	2.411242539	1.39E-08	2.45E-08
CLIC5	22.71876015	1.727578613	-3.717060837	3.10E-24	1.58E-23
ADAMTS4	2.251445369	8.919761138	1.986153605	6.43E-23	2.95E-22
KRT8P46	0.091718781	0.243367236	1.40784587	1.06E-18	3.55E-18
DAG1	33.34842403	14.99587457	-1.153052921	9.55E-38	5.12E-36
IGHV3-20	0.342937209	1.811452692	2.401130777	9.87E-06	1.45E-05
ANXA9	21.50598792	5.687017928	-1.918994148	1.19E-40	2.42E-38
MAP6	3.296785568	0.64103857	-2.362576985	2.92E-40	4.66E-38
ARHGAP45	2.681118772	7.69766429	1.521585622	1.41E-32	1.88E-31
CD96	0.392553479	2.23494666	2.509279283	3.84E-30	3.49E-29
AC004687.1	0.090107372	0.986754326	3.452973887	5.85E-29	4.63E-28
AC069281.2	0.371931094	0.765229455	1.040857041	6.78E-09	1.22E-08
AC148476.1	0.211363621	1.766309352	3.062939044	1.70E-20	6.50E-20
IGKV1-8	0.532781266	2.333930189	2.131146148	5.49E-05	7.68E-05
ANOS1	0.71460719	1.433146347	1.003963604	9.22E-15	2.38E-14
LINC01638	0.060197012	1.138491507	4.241287833	7.30E-23	3.34E-22
KDR	18.6239401	43.08507936	1.210030018	1.98E-12	4.42E-12
TMEM191A	0.305177442	0.653621366	1.098806821	6.41E-24	3.19E-23
CFAP221	2.431400757	0.908318767	-1.420517112	3.90E-30	3.55E-29
CCL5	5.452020633	51.86301717	3.249843211	5.05E-35	1.11E-33
LDLR	6.403849768	2.576844187	-1.313334158	9.73E-21	3.78E-20
ZNF831	0.072928143	0.362103727	2.311855468	8.23E-25	4.41E-24
ADRB1	0.785908845	0.294305843	-1.417045805	6.18E-34	1.06E-32
PSCA	2.625123626	0.990275588	-1.406483385	1.35E-23	6.57E-23
AC106047.1	0.69513783	0.284471958	-1.289012613	8.43E-20	3.07E-19
AKAP3	1.564919536	0.316153421	-2.307391743	3.62E-39	3.30E-37
ZBP1	0.070055515	0.526008935	2.908516772	1.37E-31	1.51E-30
C2	1.981194317	8.994835323	2.182726481	2.57E-19	9.01E-19
SNORA3B	0.100526771	0.603037111	2.58466703	4.94E-13	1.15E-12
CHD3	20.54570439	10.24434228	-1.004009431	3.07E-26	1.86E-25
RHEBL1	0.216199959	0.831478964	1.943313517	3.65E-36	1.07E-34
TRBV2	0.182627847	1.268133092	2.7957275	8.58E-22	3.62E-21
AL355376.2	1.697040675	0.185066188	-3.1969079	2.07E-13	4.91E-13
SCCPDH	53.96174569	20.90225703	-1.368278288	3.83E-21	1.54E-20
LILRA1	0.314534836	0.921088995	1.550120743	2.00E-24	1.04E-23
PPT2-EGFL8	0.098078938	0.231728214	1.240418443	5.99E-14	1.47E-13
LINC01948	1.777017847	0.613197798	-1.535033749	4.10E-26	2.46E-25
CNGA1	3.16827715	0.5471117	-2.533791229	4.37E-32	5.23E-31
VAV1	0.70746699	3.586581249	2.341874574	5.87E-34	1.01E-32
VEGFA	10.79031619	125.7155178	3.542353694	2.71E-37	1.23E-35
TRAV41	0.09234061	0.563372793	2.609052729	2.93E-21	1.19E-20
AC027271.1	0.160882491	0.402100897	1.32155023	1.41E-14	3.59E-14
PTGDS	37.2462633	15.73044518	-1.243536195	3.82E-14	9.48E-14
FCGBP	6.326709439	2.775545239	-1.188684132	1.67E-09	3.12E-09
CAMK1D	1.475041436	3.637081591	1.302025808	1.62E-29	1.36E-28
SLC17A2	0.179394519	1.838151896	3.35704827	1.19E-22	5.37E-22
MIR548AR	0.137205813	0.379202972	1.46662867	2.99E-05	4.25E-05
SORL1	18.35745235	8.767072029	-1.066198849	1.01E-21	4.24E-21
AGXT	3.632917127	0.822009876	-2.143900824	1.16E-12	2.63E-12
LINC00551	1.443060012	0.125846744	-3.519391497	1.62E-36	5.42E-35
APOC1	2.677430229	88.20676067	5.041968351	9.59E-41	2.16E-38
CHODL	1.624665236	0.511380237	-1.667674168	4.22E-27	2.75E-26

Table S1. The differentially expressed genes(DEGs) were screened out in TCGA

ID	conMean	treatMean	logFC	pValue	FDR
AZGP1	41.87893129	12.40667375	-1.755108248	2.04E-06	3.13E-06
AL353807.4	0.290060575	0.75605027	1.382127946	1.38E-07	2.28E-07
RPS20P10	0.087128626	0.215382959	1.305685417	8.90E-07	1.40E-06
FANCA	0.205336984	0.652863696	1.66878832	8.37E-28	5.82E-27
ACADM	47.14106	17.04715657	-1.467453078	2.02E-33	3.11E-32
AP001178.2	0.101110398	0.493745211	2.287835391	4.20E-12	9.17E-12
EPHA1	2.791812264	0.945351179	-1.562279665	9.19E-35	1.88E-33
ADRA1B	2.114406457	5.774293738	1.449391763	4.88E-23	2.26E-22
RPS24P17	4.402992474	0.255194408	-4.108815758	1.05E-27	7.22E-27
BVES	1.552126725	0.636864777	-1.285187364	2.43E-09	4.49E-09
XRCC3	0.362753863	1.010706636	1.478301426	3.87E-25	2.13E-24
HCG4P8	0.009524735	0.285907676	4.907726524	1.00E-26	6.33E-26
DEPDC1	0.125705224	0.487340381	1.95488516	4.67E-32	5.57E-31
TMEM56	4.093651819	1.829511066	-1.161930259	3.26E-29	2.66E-28
MISP	5.671915125	1.779461104	-1.672395546	6.67E-28	4.69E-27
AC009237.3	0.818004244	0.293099899	-1.480715857	2.55E-18	8.33E-18
ZNF667-AS1	6.669844874	2.486440702	-1.423571182	4.46E-33	6.47E-32
SULT1C2P1	0.255639436	0.563692343	1.140797558	0.002009453	0.002525833
LINC02313	0.166041423	0.547806964	1.72212441	6.21E-13	1.43E-12
AL391121.1	4.156416625	1.432769015	-1.536534229	1.12E-35	2.89E-34
GLRX5	21.86946911	10.06061614	-1.120199537	4.82E-36	1.38E-34
CDRT15P1	0.168703113	0.354445044	1.071075367	2.31E-08	4.02E-08
AL606760.1	1.255715813	0.370449339	-1.761161833	6.58E-39	5.27E-37
ATP6V1G3	18.67342761	5.808373219	-1.684780704	4.22E-44	2.63E-41
AP000696.1	3.178654573	0.055359656	-5.84343746	4.71E-42	1.98E-39
NOVA2	0.873929133	1.992309239	1.188853392	7.06E-18	2.23E-17
DLK2	0.198237225	1.013021194	2.353364553	8.73E-29	6.77E-28
AC087762.1	1.789442771	0.256268173	-2.803784182	3.66E-34	6.58E-33
AC090510.2	0.083297584	0.438557994	2.396421075	5.35E-25	2.92E-24
RN7SL838P	0.040962958	0.370421021	3.17677417	7.37E-08	1.24E-07
AC126118.1	0.105057362	0.341438131	1.700446919	2.42E-11	5.04E-11
IGKV1-33	0.068910751	0.536434485	2.960601003	0.00294533	0.003656002
XCR1	0.212447169	0.545847427	1.361393634	1.55E-09	2.90E-09
FRY	5.308358569	2.408725971	-1.139995551	3.75E-28	2.72E-27
RNF157-AS1	1.457353044	0.327546108	-2.153580504	1.15E-30	1.11E-29
MIR25	0.760879156	3.101571785	2.027260271	2.47E-17	7.56E-17
RPL36A	7.488435861	17.41447553	1.21755071	1.26E-33	2.01E-32
PRELP	16.86733319	8.212714025	-1.038300927	1.21E-13	2.91E-13
FIBCD1	0.045066528	0.926502771	4.361667066	6.71E-18	2.13E-17
ORM2	0.076579212	2.804391432	5.194591116	7.94E-13	1.82E-12
SASH3	1.770754143	10.05557653	2.505559977	5.97E-35	1.28E-33
PARD3-AS1	0.513022164	1.148093167	1.162146661	5.76E-09	1.04E-08
PRR15	14.4380112	0.873984751	-4.04612011	1.31E-40	2.58E-38
AKR1C1	8.402919547	3.821344945	-1.136810178	1.97E-08	3.44E-08
BX293995.1	0.001821509	0.287108271	7.300317268	2.26E-21	9.24E-21
HMGN1P8	0.090095302	0.299487444	1.73297174	1.71E-07	2.82E-07
RNA5SP203	0.137116472	0.466449908	1.766320269	6.54E-09	1.18E-08
FOXQ1	9.416483194	4.09015053	-1.203034413	3.48E-16	9.85E-16
RNU6-584P	0.130060679	0.368300766	1.501699535	9.34E-06	1.38E-05
TLR2	2.401574331	7.391895057	1.621963813	4.19E-27	2.73E-26
AL592431.1	0.028590686	0.405737472	3.826929395	5.93E-21	2.34E-20
CLK1	8.275146917	19.01665938	1.200407002	1.31E-18	4.38E-18
FOXF2	0.115490241	0.373069307	1.691672721	2.67E-18	8.68E-18
CD69	1.38589119	3.280271299	1.243001147	2.21E-16	6.33E-16
AC138150.1	0.152174906	0.324670405	1.093245409	0.001695939	0.002145375

Table S1. The differentially expressed genes(DEGs) were screened out in TCGA

ID	conMean	treatMean	logFC	pValue	FDR
SPON1-AS1	0.090233769	0.264997037	1.554236878	1.65E-05	2.39E-05
SH2D2A	0.393381143	2.600390207	2.724728417	4.40E-31	4.51E-30
MEST	7.929202094	3.564572498	-1.153446633	5.78E-23	2.66E-22
KL	53.4211564	18.70037734	-1.514343825	3.60E-18	1.16E-17
COL8A1	3.147452988	12.4832125	1.987732519	3.06E-24	1.56E-23
AL031602.1	0.124185362	0.301356923	1.278978082	0.005427508	0.006606452
FBLN7	0.327401933	1.513740814	2.208983461	5.27E-21	2.09E-20
DPT	10.11693416	2.111837764	-2.260201248	2.48E-23	1.18E-22
EPHA6	0.134316144	0.356306061	1.407484305	0.002756833	0.003428224
LINC02381	30.88938061	6.50874254	-2.246660189	4.90E-37	2.01E-35
DUX4L50	0.411858074	1.119098283	1.442117569	2.51E-21	1.02E-20
SFTA2	2.895263353	0.515823432	-2.488745366	1.45E-33	2.29E-32
NINJ2	0.398763239	1.284402884	1.687493488	7.26E-29	5.68E-28
ADD2	0.053915945	0.35032464	2.699908555	2.99E-22	1.31E-21
AC092645.1	0.354928091	1.014702885	1.515458686	3.74E-09	6.83E-09
IGHV1-69D	5.593177516	17.24997021	1.624853846	0.001680769	0.002126708
ARMC12	0.089948121	0.371689272	2.046932003	1.72E-19	6.11E-19
SCD	11.16170549	90.01779866	3.0116528	3.02E-35	6.99E-34
GMNC	1.494253624	0.132791998	-3.492184927	5.15E-17	1.54E-16
SUSD1	10.41714931	3.834843108	-1.441721072	1.69E-32	2.21E-31
AC022960.1	0.056336854	0.312443023	2.471442222	4.97E-09	8.99E-09
AGAP2	0.173813083	1.13906475	2.712241179	3.03E-39	2.92E-37
AL049840.5	0.282955951	0.865366204	1.6127333	2.81E-13	6.61E-13
DERL3	0.658600557	2.965654707	2.170874997	2.06E-23	9.86E-23
AC010487.2	0.181014186	0.798013912	2.140311137	5.98E-17	1.78E-16
AC007637.1	2.486030985	1.11299636	-1.159395403	1.88E-21	7.72E-21
SLC47A1P1	0.083782365	0.379165453	2.178109016	6.91E-16	1.92E-15
IGLV2-14	23.7326758	98.57276775	2.054314365	4.44E-06	6.67E-06
P4HA3	0.305583323	2.089957349	2.773835782	6.55E-29	5.16E-28
C3orf86	0.758859821	0.225647594	-1.749762014	6.16E-28	4.35E-27
AC007036.1	0.141953239	0.300129694	1.080170291	0.004693524	0.005737458
NIPAL1	4.809330513	0.496577127	-3.275746361	2.37E-38	1.57E-36
AL008723.3	0.046784342	0.287412023	2.619022758	2.29E-14	5.77E-14
SCGB3A2	0.016306443	0.698494462	5.420734615	1.53E-27	1.04E-26
IGLV2-8	4.389469195	17.26615716	1.97582863	6.16E-07	9.79E-07
ACTG1P14	0.11636481	0.266377463	1.194817198	2.19E-12	4.87E-12
YJEFN3	0.272044113	1.205023804	2.147149135	1.07E-13	2.58E-13
GAP43	0.092897367	0.251906327	1.439177747	1.97E-09	3.66E-09
FOXF1	0.901184725	2.377155022	1.399341224	4.16E-18	1.34E-17
HDAC10	0.607280494	1.769920006	1.543249221	2.83E-26	1.72E-25
PLXNC1	1.1945283	3.515213769	1.557171392	2.64E-23	1.25E-22
AC005180.2	0.283015561	0.571910798	1.014908768	8.13E-08	1.36E-07
AC011468.1	0.622300789	1.65878468	1.414442648	3.06E-24	1.56E-23
IGLV3-21	12.38973266	56.10132689	2.178889836	5.09E-06	7.63E-06
AL133215.1	0.073067412	0.222244491	1.604847648	1.66E-09	3.09E-09
PRF1	1.677410115	10.782119	2.684333371	6.58E-35	1.40E-33
TMEM233	0.613281449	2.56744766	2.06571365	1.28E-29	1.09E-28
IRX2	8.608547688	0.266990544	-5.010909314	1.31E-41	4.16E-39
TRAV8-3	0.223816011	0.979909809	2.130335726	1.91E-16	5.49E-16
AGAP9	0.659345135	1.575940362	1.257107192	8.44E-09	1.51E-08
ITGB2	4.560106314	24.18347272	2.406882064	3.25E-34	5.87E-33
MME	44.25642935	13.73022218	-1.688532087	5.23E-06	7.83E-06
S100A9	18.61200506	39.07580521	1.070042118	2.79E-10	5.44E-10
PLOD1	22.33061819	56.13843988	1.32996578	2.72E-31	2.88E-30
GCNA	0.131661904	0.398376327	1.59729395	5.04E-17	1.51E-16

Table S1. The differentially expressed genes(DEGs) were screened out in TCGA

ID	conMean	treatMean	logFC	pValue	FDR
LINC01268	0.417985647	2.241825967	2.423148979	9.01E-29	6.97E-28
LINC01607	0.626987187	0.249280033	-1.330668632	2.97E-20	1.11E-19
RNF165	0.253882234	0.681702759	1.42498338	3.92E-13	9.14E-13
MIR4782	0.16437444	0.383038563	1.220503663	5.32E-05	7.45E-05
INE1	0.721406906	1.498679534	1.054806783	8.66E-11	1.74E-10
LIX1	5.394239722	1.579757224	-1.771716773	1.23E-20	4.75E-20
BID	2.75022775	5.892018811	1.099210941	1.10E-35	2.85E-34
AC002128.1	0.20441707	0.666121531	1.704269738	6.57E-12	1.42E-11
MIR497HG	0.298492424	0.81925111	1.456611409	5.71E-22	2.44E-21
FAM153C	0.028358289	1.114719651	5.296766622	5.24E-27	3.38E-26
MAPK15	1.030271925	3.960718847	1.942737129	5.53E-13	1.28E-12
AC005104.1	0.290904015	1.230954214	2.081161986	1.63E-19	5.80E-19
FAM57B	0.006646536	0.236835249	5.155137319	2.68E-30	2.48E-29
AC112491.1	0.359300245	1.373930144	1.93504683	3.83E-21	1.54E-20
RPS20P22	0.11999109	0.28187575	1.232132092	4.20E-05	5.92E-05
AC009041.4	0.198565477	0.431808615	1.12077721	5.20E-16	1.46E-15
GTF2IP23	0.657663858	1.902858313	1.532745849	3.95E-14	9.77E-14
AC010333.2	0.091907648	0.228273823	1.312508616	0.015290585	0.017959982
NUPR1	11.25090435	28.31621185	1.331587304	3.33E-14	8.30E-14
MIR210	0.255814454	3.803167669	3.894031859	5.89E-30	5.23E-29
SERPINA1	106.3144874	333.446596	1.649117516	7.83E-14	1.91E-13
ADAM8	0.588298344	2.56805713	2.126057416	8.44E-28	5.86E-27
AC145098.1	0.201499502	1.140631816	2.500985002	5.77E-34	9.93E-33
FDXR	2.955498444	7.781235188	1.396597725	5.67E-32	6.70E-31
UNC13D	0.448942081	2.060304782	2.198256536	1.66E-33	2.60E-32
AL590999.1	0.100026162	0.680252753	2.765693495	4.65E-19	1.60E-18
OASL	1.068942564	3.167356973	1.567095137	7.52E-24	3.71E-23
DTX2P1	0.214173535	0.671395063	1.648381706	1.76E-27	1.18E-26
UGT2B7	235.8441965	102.7707512	-1.198404373	0.022276879	0.025867816
LAIR1	0.892522766	5.657179956	2.664122191	2.97E-36	8.96E-35
PSMG3-AS1	2.237111832	0.938160688	-1.253730424	7.25E-30	6.34E-29
PECAM1	38.35099306	91.37525312	1.252539562	3.13E-21	1.26E-20
AC129507.2	3.994117367	1.917423659	-1.058707588	3.00E-21	1.21E-20
AC025627.1	0.300878241	1.372503166	2.189557797	1.03E-12	2.35E-12
MYOC	0.582438392	1.198950681	1.041594955	3.23E-05	4.59E-05
LST1	1.79740901	8.571814986	2.253681972	9.86E-35	2.01E-33
AL021068.1	1.84244905	0.321199044	-2.520085222	1.21E-37	6.18E-36
ITGAD	0.021203334	0.600487968	4.823772315	1.71E-39	1.90E-37
XPNPEP2	59.53000315	1.464139854	-5.345491699	2.88E-24	1.47E-23
AC008610.1	0.648958053	1.7859403	1.460486722	1.03E-11	2.20E-11
HOXA11-AS	0.80562704	0.315004117	-1.354741423	1.05E-27	7.22E-27
CD86	1.224751815	5.447647688	2.153143974	1.68E-30	1.60E-29
AC018638.8	1.696416133	3.533608455	1.058652081	4.46E-10	8.58E-10
TRAV1-2	0.166123721	0.76676314	2.206522893	1.26E-16	3.68E-16
WISP2	0.139120158	1.440516521	3.372182828	3.89E-14	9.65E-14
SLC30A2	8.328763157	0.800618674	-3.378915096	3.18E-30	2.92E-29
AC005839.1	0.2154785	0.530010609	1.29847731	4.54E-21	1.81E-20
GAPDH	522.868625	1428.527476	1.450008377	7.25E-37	2.78E-35
MORN5	0.81603014	0.39418697	-1.049742352	7.72E-21	3.02E-20
ST14	37.08638458	14.5993315	-1.344987322	2.05E-26	1.26E-25
ERO1A	6.665221556	18.30561546	1.457561542	1.04E-35	2.74E-34
MPP5	12.08307018	4.76055887	-1.34378422	1.01E-37	5.30E-36
ZNF426-DT	1.810307133	0.798110749	-1.181573623	8.82E-22	3.72E-21
AP006296.1	0.583085258	0.161696565	-1.850417816	1.15E-29	9.89E-29
DDX47	0.147418555	0.473119744	1.682287248	1.44E-24	7.57E-24

Table S1. The differentially expressed genes(DEGs) were screened out in TCGA

ID	conMean	treatMean	logFC	pValue	FDR
HIST2H2BD	0.134587179	0.276242957	1.037396702	3.94E-08	6.75E-08
CBLN4	0.384914935	1.655152095	2.104352239	8.78E-11	1.77E-10
AC006270.1	0.035903486	0.378536648	3.398237156	2.08E-09	3.86E-09
GTSE1-DT	0.813555493	0.290259252	-1.486898701	1.57E-26	9.76E-26
SLAMF8	1.087885257	7.058921058	2.697921289	2.11E-31	2.27E-30
MKLN1-AS	2.763880447	1.275506148	-1.11562536	1.94E-23	9.33E-23
TMEM179	0.080882284	0.877063473	3.438785605	6.81E-16	1.89E-15
CENPM	0.391057447	1.765151577	2.174339612	8.69E-36	2.34E-34
MIR3164	0.141361582	0.673699875	2.252715942	3.14E-11	6.50E-11
HLA-DQB1-A	0.995988793	4.980997234	2.322233196	6.05E-24	3.02E-23
AC073611.1	0.303039913	1.439374415	2.24786219	1.96E-35	4.74E-34
EMB	1.590882528	4.852979378	1.60904342	6.90E-24	3.41E-23
SLC2A1-AS1	0.255366381	0.606771646	1.248585066	2.57E-17	7.84E-17
TTC4P1	0.015392382	0.436816111	4.826737692	6.09E-24	3.04E-23
PPIAP72	0.066204868	0.222016041	1.745654701	7.04E-13	1.62E-12
MYL5	1.239646678	2.933956057	1.242918279	1.41E-25	8.10E-25
HNRNPA3P9	0.109791826	0.250439642	1.189692294	6.67E-08	1.13E-07
MNDA	2.405670243	8.574885096	1.83367844	3.07E-26	1.86E-25
AC131212.2	0.124790647	0.437859981	1.810959789	1.82E-13	4.32E-13
CMTM2	0.161999704	0.368444491	1.185456106	1.31E-11	2.77E-11
SLC1A3	0.5760844	3.952321389	2.77834817	3.19E-32	3.92E-31
AP003419.3	0.174521697	0.562227973	1.687748827	8.50E-12	1.82E-11
ATP5F1B	521.4805528	259.2292114	-1.008385154	2.70E-32	3.37E-31
AL513477.2	0.355740914	0.839156655	1.238113252	3.71E-11	7.64E-11
AC103739.1	0.092515742	0.249590346	1.431791369	1.05E-09	1.98E-09
KAT2A	5.348032903	11.88696787	1.152300513	1.06E-17	3.32E-17
AC010469.1	0.09448154	0.234176036	1.309489058	0.000235574	0.000316209
TNIP1	36.42815028	75.60517377	1.053431223	2.61E-30	2.42E-29
SNORD72	0.106631656	0.35935019	1.752754656	1.96E-05	2.82E-05
GLTPD2	6.746808654	3.294859115	-1.033988468	3.78E-07	6.08E-07
TAF1D	2.674472222	7.676171724	1.521132767	3.84E-38	2.38E-36
CIB4	0.204928942	1.183853107	2.530294427	5.19E-18	1.66E-17
AC079779.2	0.537962561	1.575286866	1.550036895	0.007791627	0.009371073
AL021707.6	0.457953759	1.920309812	2.06806525	1.75E-18	5.76E-18
C17orf107	1.043981781	2.347476805	1.169014369	6.48E-14	1.58E-13
AC011939.2	0.008456496	0.240638367	4.830662878	7.92E-11	1.60E-10
AC079145.1	0.783475566	0.317123217	-1.304844779	1.76E-18	5.80E-18
SCARNA20	0.093194376	0.421784071	2.178189811	3.67E-08	6.30E-08
LINC01886	0.307132188	1.617644497	2.396962965	1.58E-14	4.02E-14
AL592546.1	0.840954661	0.119799743	-2.811403206	3.73E-35	8.40E-34
MAL2	94.40825665	13.64566843	-2.790469972	3.03E-37	1.34E-35
CYP21A2	0.115831797	0.658639805	2.507458363	3.12E-22	1.36E-21
HSPA8P15	0.078928468	0.29046177	1.879730643	2.41E-16	6.90E-16
TP53I11	9.189451056	19.54063697	1.088426908	6.23E-18	1.98E-17
AC093583.1	7.450630268	2.086020688	-1.836609006	1.21E-22	5.44E-22
NANOS1	1.425184593	0.462028822	-1.625094035	2.46E-21	1.01E-20
AC027796.2	0.089327744	0.288656987	1.692175909	7.10E-09	1.27E-08
GOLGA6L7	0.015724672	0.272924737	4.117401362	0.008119664	0.009754778
IDUA	1.524896753	3.599673327	1.239154422	3.72E-24	1.89E-23
ASAP2	8.773921986	4.02941523	-1.122651396	1.92E-29	1.60E-28
ZMYND12	4.208118525	1.681946015	-1.32304394	7.66E-31	7.62E-30
HMGB3P32	0.037206387	0.228376937	2.617794766	6.99E-20	2.56E-19
CFP	0.347637119	0.871758225	1.326345938	5.97E-20	2.19E-19
AKIRIN1	32.9478725	15.62639632	-1.076200202	1.45E-34	2.84E-33
GPR155	6.365621819	3.074384805	-1.050003698	9.44E-22	3.97E-21

Table S1. The differentially expressed genes(DEGs) were screened out in TCGA

ID	conMean	treatMean	logFC	pValue	FDR
VGLL3	1.725942197	0.679618587	-1.344586934	2.63E-18	8.58E-18
AC026471.3	0.916604604	0.190983233	-2.262853549	3.50E-19	1.22E-18
ODF2-AS1	0.060157594	0.223793034	1.895346354	4.07E-11	8.35E-11
YES1P1	0.033598984	0.260090488	2.952524128	3.06E-24	1.56E-23
FAM71F2	0.136669188	0.336417564	1.299565009	3.71E-07	5.97E-07
BDH2P1	0.416468143	0.203678631	-1.031911518	3.08E-13	7.21E-13
CKM	0.902833927	0.129967094	-2.796314232	1.68E-16	4.85E-16
CLEC5A	0.340400289	1.420781834	2.061380874	3.36E-25	1.86E-24
CDC6	0.273359045	1.075979973	1.976782209	1.10E-33	1.78E-32
PHGDH	15.55599317	6.076919757	-1.356058359	6.45E-26	3.81E-25
AL139421.1	0.145582998	0.412085328	1.501101215	8.77E-12	1.88E-11
TMEM72	62.32633997	7.618779573	-3.032210174	9.26E-37	3.45E-35
RNU6-312P	0.263787836	1.222036765	2.211837746	2.39E-15	6.41E-15
WDR62	0.108813111	0.315398072	1.535321439	1.52E-28	1.15E-27
RGS1	3.688435003	32.59565004	3.143598729	7.71E-35	1.60E-33
GXYLT2	0.665322289	2.543195282	1.934516975	1.43E-10	2.84E-10
SIPA1L2	3.187093342	7.282658391	1.192223901	4.73E-24	2.38E-23
RNU6ATAC1	0.201079619	0.710020398	1.820093608	3.63E-05	5.14E-05
PLK2	5.418387389	25.39042822	2.228349279	2.44E-35	5.76E-34
RNA5SP18	0.147594589	1.75606293	3.572632808	2.70E-29	2.22E-28
AC008555.5	0.616108581	1.60007634	1.376884205	2.86E-18	9.27E-18
ANGPTL8	0.207657269	3.07074218	3.886311108	1.84E-08	3.22E-08
RNU6-387P	0.169787466	0.510991274	1.589568691	8.43E-08	1.41E-07
LDHAP3	0.205788991	0.725188683	1.817190604	1.44E-16	4.17E-16
AC120498.2	0.036224045	0.244667768	2.755804487	1.06E-10	2.12E-10
SLC38A3	1.429770997	0.437433077	-1.708649871	4.21E-31	4.34E-30
AGAP4	0.232678751	0.511758074	1.137122495	6.62E-14	1.62E-13
LINC02188	0.288385679	4.111824487	3.833707255	3.96E-33	5.80E-32
LINC02487	0.073158506	0.242901513	1.731273964	6.11E-21	2.41E-20
IQGAP2	13.63318647	6.589664084	-1.048845972	1.97E-12	4.39E-12
AC009185.1	0.869134477	0.265513657	-1.710793345	1.49E-22	6.63E-22
THSD7A	10.1749424	3.184596181	-1.675838278	2.49E-35	5.86E-34
RNF43	3.682605844	0.636477177	-2.532546304	5.50E-38	3.22E-36
AC100812.1	0.104792781	0.264963836	1.338256134	0.001312864	0.001674985
HLA-A	258.0919347	835.7994612	1.69527177	4.97E-38	2.96E-36
GDF6	0.37890828	4.544392649	3.584166923	2.78E-29	2.27E-28
PRRX2	0.448125566	1.025560219	1.194437267	4.36E-05	6.14E-05
AC079313.1	0.050808629	0.403234257	2.988472755	3.32E-23	1.56E-22
PPP1R3C	7.713945653	29.83253044	1.951345467	1.92E-32	2.48E-31
MAP3K9	1.292951718	0.409639468	-1.658241774	3.13E-36	9.37E-35
AC034243.1	1.247529015	0.437180451	-1.512772574	3.07E-16	8.71E-16
SLCO2B1	3.712007903	11.62793469	1.647323186	3.01E-22	1.32E-21
AF064858.3	0.393570098	3.733111896	3.245686231	4.08E-17	1.23E-16
PPP1R3G	0.450707474	2.008353376	2.15574986	8.05E-35	1.67E-33
SUCLA2-AS1	0.831175993	0.367382985	-1.177869173	8.57E-28	5.94E-27
AL021707.8	0.398045527	1.502743392	1.916593322	1.98E-16	5.70E-16
EFNA1	21.27078333	50.18426558	1.238361939	9.48E-26	5.51E-25
PRRT3	1.363433739	0.558356262	-1.287986749	5.32E-31	5.40E-30
AL391832.3	0.098967349	0.230033231	1.216817749	1.39E-16	4.04E-16
SLC14A1	13.07680767	1.642118798	-2.993379984	3.05E-27	2.02E-26
KCNH6	2.296244342	0.619206005	-1.890784798	0.000275594	0.000368078
C1QTNF1-AS	0.115380541	0.494735175	2.100256545	7.26E-07	1.15E-06
LRRC19	17.56810768	4.554621854	-1.947555617	7.21E-33	1.00E-31
AC004923.4	0.157851053	0.649031048	2.039723608	3.03E-22	1.32E-21
IL18RAP	0.202747075	0.690481198	1.767921031	3.10E-24	1.58E-23

Table S1. The differentially expressed genes(DEGs) were screened out in TCGA

ID	conMean	treatMean	logFC	pValue	FDR
IGKV1D-16	0.38308698	1.78084432	2.216817506	4.62E-05	6.50E-05
TSPAN8	25.3138838	1.662685825	-3.928341378	7.50E-39	5.87E-37
MIR6819	0.131684028	1.098284751	3.060099875	9.24E-17	2.71E-16
CTHRC1	1.958374904	16.23718885	3.051572999	1.44E-27	9.76E-27
SLC25A15	4.449283264	2.080018394	-1.096976665	1.30E-32	1.73E-31
SLC9B2	0.6084479	1.311727146	1.108262015	1.01E-18	3.38E-18
ADCY8	0.108427298	1.002621743	3.208977506	0.002645	0.003293533
OXNAD1	3.333847335	1.525834259	-1.127589781	2.81E-32	3.49E-31
PSMB8-AS1	3.218310639	9.132174165	1.504654789	3.14E-37	1.38E-35
RNU6ATAC1	0.106469904	0.290015174	1.445682709	0.00161349	0.002044218
LINC01485	0.914876842	0.224060733	-2.029687706	3.48E-30	3.19E-29
HSF4	0.362248841	19.79881145	5.772288941	4.21E-41	1.14E-38
SMIM3	14.50620486	31.31809397	1.110326284	3.40E-05	4.81E-05
AC010422.2	0.079124815	0.265622299	1.747174149	1.59E-11	3.35E-11
RNA5SP311	0.31301377	0.794904848	1.344556053	4.90E-06	7.34E-06
TMEM86B	0.293071884	0.69513487	1.246038347	1.08E-18	3.62E-18
OMG	0.037640447	1.127563107	4.904780599	1.83E-25	1.04E-24
AC011481.1	0.20753463	0.776333208	1.903323909	2.38E-11	4.96E-11
PDLIM7	4.963670014	9.946592747	1.0027952	4.24E-18	1.36E-17
MIR23C	0.026604671	0.886837322	5.058918027	1.41E-16	4.10E-16
GAPDHP65	0.493681739	1.111621388	1.171012311	9.09E-17	2.67E-16
ZNF205	3.701642111	7.600154861	1.037863399	1.20E-31	1.33E-30
TEX11	0.167128398	1.922829313	3.524201906	2.49E-21	1.02E-20
GBAP1	0.408327738	0.906165487	1.150046971	3.81E-17	1.15E-16
ZGLP1	0.148044303	0.356489216	1.26782946	4.48E-14	1.10E-13
TBC1D30	1.277251519	0.593503084	-1.105715221	4.98E-31	5.07E-30
AC138393.3	0.186656787	0.389095668	1.059736951	0.001242173	0.001587847
PAGE2B	0.009714654	0.597116308	5.941705605	1.08E-09	2.03E-09
AL162741.1	0.046637788	0.230315141	2.304037977	1.56E-20	6.00E-20
AC139887.1	0.42619266	0.957318528	1.167493286	3.46E-10	6.70E-10
WNT10A	1.258375029	0.532367063	-1.241068725	5.79E-18	1.84E-17
STAB1	4.505997458	14.95877809	1.731073928	2.67E-29	2.19E-28
CD93	21.28892224	45.23346658	1.08728765	1.38E-14	3.52E-14
AL033397.1	0.541005382	0.223970083	-1.272336907	1.03E-08	1.84E-08
FAM193B	1.966080549	8.585823902	2.126634154	7.19E-30	6.29E-29
AC139100.1	0.207473974	0.417210952	1.007846656	2.78E-08	4.81E-08
MCAM	15.03714426	63.34914728	2.074794592	1.48E-31	1.62E-30
Z84485.1	0.19281286	0.493649144	1.356284746	1.18E-09	2.21E-09
LTA	0.091767198	0.577242079	2.653126016	1.06E-27	7.30E-27
AC039056.2	1.148164435	0.572155555	-1.004849936	3.18E-08	5.48E-08
TRAV12-1	0.194002807	1.310076449	2.755501573	7.45E-20	2.72E-19
SPC24	0.29440191	1.416040924	2.266004024	1.13E-33	1.82E-32
ZDHHC15	1.221313929	0.50516936	-1.273595038	3.51E-30	3.21E-29
BEST1	0.245153189	0.912996022	1.896925047	1.20E-32	1.62E-31
GNRH1	0.178043116	1.28296754	2.849186111	1.65E-31	1.80E-30
ENPP3	3.092035128	78.62046653	4.66827631	3.06E-32	3.77E-31
SLC6A1	0.37253083	1.828547538	2.295266404	3.26E-28	2.37E-27
CSPG4P10	0.113999015	0.249664344	1.130968435	5.40E-12	1.17E-11
RNU6-549P	0.03189019	0.392262146	3.620633507	3.60E-09	6.57E-09
DLGAP5	0.248132319	0.993828229	2.001886866	2.98E-32	3.68E-31
WNK1	34.29279611	12.25156867	-1.48493906	1.14E-34	2.28E-33
RHBDF2	2.104422932	8.122403312	1.948481989	1.08E-37	5.60E-36
RNU6-824P	0.122884462	0.322448376	1.391765701	0.001658263	0.002099134
VAT1L	6.471405132	1.085896058	-2.575192982	6.01E-32	7.08E-31
GP2	3.012645749	0.025188552	-6.902119031	1.11E-42	5.86E-40

Table S1. The differentially expressed genes(DEGs) were screened out in TCGA

ID	conMean	treatMean	logFC	pValue	FDR
TNFRSF14-A!	0.585105108	1.91843143	1.713159481	2.19E-21	8.98E-21
AP005131.5	0.01885711	0.277348897	3.878521507	3.78E-14	9.39E-14
RNU7-84P	0.184455976	1.164476448	2.658333023	1.68E-06	2.60E-06
CLEC2B	1.543810914	7.493377704	2.27912011	7.08E-36	1.94E-34
RPS29P16	0.269950593	0.738530936	1.451962971	3.49E-13	8.16E-13
VWCE	0.467453959	3.153475373	2.754046485	4.32E-23	2.01E-22
WNK3	0.755702029	0.281478157	-1.424794527	1.05E-33	1.71E-32
SLC22A6	89.75103601	12.76920908	-2.813259424	2.08E-10	4.10E-10
GPSM3	4.883909375	18.68207295	1.935546209	2.53E-35	5.95E-34
FCGR2C	0.161353472	1.876844382	3.540012511	1.99E-34	3.77E-33
LINC01077	0.195286124	6.408140665	5.03624447	4.11E-36	1.19E-34
MSRA	19.17340599	8.286897354	-1.210202684	2.01E-08	3.50E-08
OVCH2	3.986696969	0.19003316	-4.390870859	7.66E-42	2.82E-39
PRR36	1.141640168	0.419455619	-1.444517922	7.92E-28	5.52E-27
AC127502.1	0.13812623	0.351445354	1.347313069	1.62E-19	5.78E-19
ARHGAP4	3.639885756	7.800654666	1.099702037	1.13E-19	4.09E-19
CSAD	1.590289622	4.768674093	1.584298656	1.24E-13	2.99E-13
PLXND1	9.233672639	28.41906014	1.621882353	6.88E-31	6.89E-30
AC019171.1	0.455062892	0.222421468	-1.0327699	6.46E-22	2.75E-21
USP46-AS1	4.355235583	1.58024621	-1.462601404	3.25E-38	2.06E-36
AL592114.3	0.831015308	0.287067181	-1.53348665	4.35E-22	1.87E-21
TFAP2B	6.527445387	0.126779197	-5.686128545	3.64E-41	1.01E-38
NCF4	1.735199821	7.074388123	2.027503563	2.00E-32	2.58E-31
PRLR	5.089004043	1.480109057	-1.781679857	5.65E-17	1.68E-16
MIR509-1	0.018223036	0.317926874	4.124859723	2.86E-13	6.71E-13
AC022784.5	0.237053066	0.78364534	1.72499082	7.66E-15	1.99E-14
AL049838.1	9.722105714	0.849197715	-3.517096428	9.90E-40	1.24E-37
AL390208.1	0.176572623	0.473033455	1.421680547	2.12E-13	5.03E-13
AC008906.1	0.438708328	1.025164652	1.224521642	5.75E-17	1.71E-16
AL512274.1	0.598685306	0.288258902	-1.054432698	2.33E-11	4.87E-11
PKNOX2	0.785185504	0.268871452	-1.546116957	2.07E-27	1.39E-26
PNPLA7	0.564264138	1.717209593	1.605623572	3.18E-16	9.03E-16
TRBV4-1	0.123017573	1.354294019	3.460604661	1.01E-21	4.23E-21
KLHL2P1	0.183310527	0.735908388	2.00523654	2.74E-28	2.01E-27
TRAJ35	0.43073961	0.187077841	-1.203177312	1.11E-06	1.73E-06
MUC20-OT1	0.875010683	2.629490607	1.587410807	5.48E-14	1.34E-13
CFAP73	0.475466897	0.172819063	-1.460082536	1.56E-10	3.10E-10
EGFL8	0.203743336	0.776781758	1.930756449	1.69E-13	4.02E-13
GRIP1	1.363728893	0.548100122	-1.315045508	8.11E-28	5.65E-27
LINC00861	0.089858191	0.728956025	3.020109864	1.93E-31	2.09E-30
SAT2	60.59576681	28.08924389	-1.109199221	5.14E-22	2.21E-21
TNFRSF1B	8.299252042	22.28444455	1.424983775	6.71E-29	5.27E-28
ROPN1L	0.27458781	0.649811253	1.242753148	3.55E-19	1.23E-18
NUGGC	2.032220332	0.87120925	-1.22196565	4.50E-07	7.21E-07
PAQR7	24.04133847	7.993242147	-1.588664519	1.20E-41	4.10E-39
AC022400.4	0.111495531	0.293373839	1.395754337	3.69E-07	5.95E-07
AC012651.1	0.152596748	0.542478263	1.829841114	6.26E-15	1.63E-14
SLC15A2	6.131489517	0.749574899	-3.032093041	7.75E-40	1.01E-37
EGLN3-AS1	0.010421138	0.280675426	4.751317974	2.40E-23	1.14E-22
AL390879.1	0.297520518	0.801580617	1.429858459	7.05E-23	3.23E-22
OR51E2	0.253898779	1.216764734	2.260724882	4.56E-24	2.30E-23
BCO1	1.194116639	3.607608036	1.595098836	1.91E-20	7.29E-20
N4BP2L2-IT2	0.146033703	0.772945436	2.404065209	1.96E-27	1.31E-26
HTRA4	0.026596515	0.773371221	4.861851933	8.95E-39	6.89E-37
AC007216.4	0.146962967	0.409994786	1.480152908	7.05E-14	1.72E-13

Table S1. The differentially expressed genes(DEGs) were screened out in TCGA

ID	conMean	treatMean	logFC	pValue	FDR
CACNA1F	0.062106309	0.302419122	2.283737639	5.93E-18	1.89E-17
RPL26P6	0.531248247	1.310747938	1.302932196	8.55E-16	2.36E-15
C19orf38	0.843918336	2.018322889	1.257981689	4.51E-22	1.94E-21
GINS2	0.527853749	1.929898306	1.870314663	2.85E-34	5.18E-33
TAS2R31	0.092076458	0.252236324	1.45387181	5.88E-08	9.95E-08
AQP9	0.912599834	4.128871543	2.177693238	1.69E-16	4.89E-16
IGHV3-71	0.149036959	0.508639373	1.770972998	0.006594405	0.007976828
HIST1H2AE	1.329814042	4.227189301	1.668474204	2.40E-06	3.67E-06
NPHP3	0.546880407	1.24187734	1.183225406	1.11E-21	4.62E-21
ANGPTL3	11.02284145	3.314820816	-1.733493375	2.96E-23	1.40E-22
AC003984.1	0.236368805	1.190645968	2.332632946	2.89E-11	5.98E-11
C11orf86	0.377785484	1.855436468	2.296119427	6.17E-05	8.60E-05
RAB11FIP4	3.896452251	1.002758543	-1.95818688	1.88E-39	2.00E-37
RNU6-638P	0.185216857	0.659679576	1.832550033	0.000142388	0.000193856
AC009974.1	0.149144972	0.553979095	1.893116198	3.59E-17	1.09E-16
MZB1	1.137554077	5.015647031	2.140500694	3.30E-08	5.68E-08
SNORD94	0.613890572	9.947254898	4.018245028	2.81E-19	9.83E-19
SLC25A35	3.426801074	1.130561837	-1.599822542	9.09E-37	3.40E-35
RASAL3	0.619171098	4.023561188	2.700062937	8.02E-38	4.39E-36
AC016722.2	0.087689023	0.25058467	1.514830002	1.58E-14	4.02E-14
NMB	4.496406361	26.26324022	2.54620059	1.59E-31	1.74E-30
COLGALT1	8.855732306	29.15959154	1.719286993	4.73E-37	1.95E-35
AP000844.2	0.026764018	0.587040146	4.455092555	1.45E-16	4.20E-16
TSHR	0.046037775	0.251863624	2.451752769	2.01E-23	9.66E-23
SEL1L3	6.965037833	22.26376404	1.676494429	1.79E-28	1.34E-27
LINC01789	0.237053931	2.248996411	3.245994138	4.17E-15	1.10E-14
OGFR-AS1	0.148351809	0.385462182	1.377566803	3.70E-15	9.80E-15
HK3	0.300916402	1.835402138	2.608661545	6.24E-34	1.06E-32
GAPDHP60	0.12034672	0.311475617	1.371922411	1.40E-19	5.00E-19
ABTB2	9.307763861	2.96728645	-1.649290404	7.03E-33	9.77E-32
MIR378J	0.356172236	0.84780507	1.251157533	1.60E-07	2.64E-07
AC090164.2	0.014076668	0.261995536	4.218164453	5.84E-23	2.69E-22
NPY5R	1.490346287	0.283573824	-2.393851313	8.53E-37	3.24E-35
SNORD13E	0.138778947	0.324206264	1.224123244	0.0031541	0.003907842
GPR4	4.896542583	18.8594624	1.945453216	4.68E-28	3.34E-27
SNHG4	0.220817588	0.506311716	1.197170784	1.52E-12	3.41E-12
RNU6-689P	0.201123263	0.622534452	1.630073723	6.33E-09	1.14E-08
AL031429.1	1.186410315	0.402810612	-1.558429451	1.86E-27	1.25E-26
NPNT	36.50304207	9.270894031	-1.977236324	1.14E-30	1.11E-29
Z95152.1	0.093286801	0.25916546	1.474128585	2.77E-06	4.22E-06
AF064858.1	0.218331442	1.656994642	2.923977124	1.01E-20	3.92E-20
AK7	2.556986644	0.904119979	-1.499858486	1.27E-31	1.41E-30
DEF6	1.186172283	5.79958666	2.289636516	8.38E-34	1.40E-32
F2	0.225922261	2.47904949	3.455888738	3.47E-12	7.63E-12
AC112220.2	2.461206015	1.167221631	-1.076286899	6.85E-33	9.56E-32
C19orf33	8.927035264	47.67551722	2.41699556	2.81E-20	1.06E-19
RNU6-969P	0.066501437	0.249659715	1.908505624	0.00212271	0.002663316
AC078864.1	1.264192232	9.301077437	2.879181993	8.13E-27	5.17E-26
RNU1-22P	0.067135909	0.220395214	1.714936366	5.99E-07	9.51E-07
AL021707.1	0.103823628	0.347080997	1.741137579	2.82E-14	7.06E-14
AP005131.2	0.084327603	0.345269043	2.033644142	7.64E-17	2.26E-16
AC063965.1	0.111036948	0.705899427	2.668422829	2.30E-17	7.06E-17
CD82	25.91812381	9.414445216	-1.461013301	6.32E-16	1.76E-15
AC025423.1	0.058959054	0.264217215	2.16393919	2.28E-09	4.22E-09
AC106028.3	0.127531214	0.269299096	1.078358983	5.42E-10	1.04E-09

Table S1. The differentially expressed genes(DEGs) were screened out in TCGA

ID	conMean	treatMean	logFC	pValue	FDR
FAM169A	2.59635319	0.288669016	-3.168998478	1.41E-38	1.01E-36
BHLHE41	5.679504139	38.34068989	2.755039414	1.19E-35	3.05E-34
CSNK1E	7.571308292	16.32324537	1.108313403	1.42E-33	2.25E-32
CYP26B1	4.758004643	1.56253881	-1.606464656	1.39E-25	7.98E-25
AC105020.1	0.103422968	1.258347583	3.604901961	3.08E-35	7.11E-34
COL27A1	1.674292196	5.849796132	1.804835019	1.22E-25	7.05E-25
AC008764.6	0.158822782	0.479355328	1.593677599	8.50E-20	3.09E-19
PTPRC	2.958021914	10.97800215	1.891910882	1.70E-24	8.89E-24
AC016582.3	1.895043181	0.929078405	-1.028358466	1.03E-19	3.74E-19
TMEM207	10.33718361	0.036153342	-8.159498463	6.98E-58	2.05E-54
TUBB2B	6.639621743	1.075469071	-2.626135018	1.77E-35	4.36E-34
AL049555.1	3.540425111	0.370257527	-3.257321632	5.29E-36	1.50E-34
AC027559.1	0.523698049	1.824900066	1.801010328	2.74E-28	2.01E-27
AC099548.2	0.119738382	0.386389367	1.690169716	1.27E-29	1.08E-28
TRBV3-1	0.200870173	1.049109697	2.384830274	2.68E-19	9.37E-19
AC005041.3	0.172677064	0.583222112	1.755968947	1.55E-31	1.70E-30
UGT2B11	0.125563487	0.286072059	1.187961595	0.00137201	0.001748387
CCL23	1.164984526	0.425394819	-1.453436424	3.04E-14	7.58E-14
AC104534.1	0.252252502	0.950528673	1.913861567	1.81E-15	4.90E-15
SIX1	0.08427676	0.452755343	2.425524915	9.31E-32	1.06E-30
LMX1B	1.775058821	0.10747216	-4.045831939	2.51E-23	1.19E-22
TMEM14EP	0.015075859	0.30137928	4.321268081	1.12E-05	1.65E-05
LZTS1	1.570720407	8.352466607	2.41077591	1.22E-32	1.64E-31
ANXA3	8.501242389	2.065835753	-2.04094814	1.24E-31	1.38E-30
NUF2	0.191138972	0.74719425	1.96686139	1.24E-33	1.98E-32
GALNT16	1.788341536	0.875946169	-1.029708171	1.67E-18	5.53E-18
AC002553.1	0.484196907	1.375797734	1.506602616	2.07E-18	6.81E-18
AC117498.2	1.43587889	0.329297942	-2.12446867	4.95E-35	1.09E-33
AL645608.1	0.101781727	0.217419661	1.095003836	6.22E-06	9.26E-06
CYP51A1P2	0.061836636	0.237276212	1.940033724	3.17E-22	1.38E-21
CCNL2	3.985122583	14.25868958	1.839145392	7.27E-20	2.66E-19
RPS15AP6	0.026806154	0.229122486	3.095482888	4.91E-12	1.07E-11
BNIP3	18.27417681	54.27520146	1.570486757	5.11E-30	4.57E-29
AC087482.1	3.536526086	14.45827188	2.031490317	1.04E-21	4.36E-21
AL360181.1	0.225065234	0.599871432	1.414310109	1.05E-06	1.65E-06
AL161909.1	0.216490828	0.438656755	1.01878658	1.24E-07	2.06E-07
AC104463.2	0.134375838	0.44707214	1.734233888	1.44E-10	2.85E-10
UBE2C	0.822440413	6.01320007	2.870149899	7.78E-35	1.62E-33
AL031717.1	0.122615147	0.591304273	2.269763488	2.41E-19	8.46E-19
NPTN-IT1	0.198570018	0.607257592	1.612660817	3.49E-12	7.67E-12
VN1R84P	0.112471969	0.246153115	1.129990514	0.010264816	0.012230571
AC007376.2	0.111342407	0.267628801	1.265230198	5.10E-11	1.04E-10
MCTP2	0.857558938	2.128782752	1.311720994	1.21E-28	9.23E-28
AC105118.1	0.022909954	0.282846104	3.62597083	4.69E-07	7.50E-07
AC018766.1	0.122396923	0.295118461	1.269726876	7.59E-05	0.000105223
HIST1H2AM	0.086024004	0.588016089	2.773044447	4.55E-21	1.81E-20
AC025459.1	0.093038716	0.279603475	1.587479195	2.20E-08	3.83E-08
SLC43A3	2.669088651	7.836844108	1.553925575	3.97E-28	2.86E-27
RNU6-834P	0.153989256	0.377288884	1.292839903	4.27E-05	6.02E-05
GPR82	0.120444171	0.526660157	2.128507751	7.59E-25	4.08E-24
AL049612.1	0.125218466	0.326142816	1.38105652	5.98E-07	9.50E-07
FBXL6	1.656425131	3.557525683	1.102801176	6.55E-24	3.25E-23
RPL21P123	0.030359801	0.234279826	2.947998495	8.11E-13	1.86E-12
SLC6A1-AS1	0.062080255	0.38010067	2.614175168	4.09E-19	1.41E-18
AP005899.1	0.151139008	0.820445992	2.440532312	3.40E-27	2.24E-26

Table S1. The differentially expressed genes(DEGs) were screened out in TCGA

ID	conMean	treatMean	logFC	pValue	FDR
AC136428.1	0.102453534	0.415324522	2.019269304	0.007261453	0.008758497
ALDOA	158.1132872	333.2219371	1.07552477	2.79E-32	3.47E-31
RNU6-882P	0.30552245	0.755742728	1.306616798	2.63E-06	4.01E-06
LINCMD1	0.122562657	0.538764447	2.136135178	7.07E-12	1.52E-11
AC011815.3	0.325129649	1.117828123	1.781611348	1.91E-18	6.28E-18
SLC36A2	16.97225293	0.714666882	-4.569763338	1.16E-17	3.61E-17
TRBV6-6	0.108635302	0.675095226	2.635598024	3.66E-19	1.27E-18
LINC00989	0.268249996	0.67486527	1.331021362	5.72E-14	1.40E-13
OSM	0.525026197	1.919826635	1.870514724	9.11E-15	2.35E-14
MACROD1	14.06170969	6.90449152	-1.026164939	1.43E-21	5.91E-21
AC011466.1	0.095968483	0.243341932	1.342352343	1.00E-10	2.01E-10
L2HGDH	4.625866342	1.803637072	-1.358814514	1.76E-30	1.67E-29
CLDN4	49.91557688	21.58078602	-1.209742688	1.08E-13	2.61E-13
AL121987.2	0.068106159	0.234777903	1.785439457	9.19E-22	3.86E-21
RNU6-130P	0.103222025	0.37507676	1.86143504	0.000607808	0.000792598
ZNF692	1.508794296	5.382348191	1.834839597	1.82E-26	1.12E-25
ANK1	0.244951047	1.192060163	2.282891686	6.57E-13	1.51E-12
AC108474.1	0.045911949	0.288005497	2.649154757	4.38E-07	7.03E-07
DCDC2B	0.060301009	0.23508126	1.962905484	2.37E-13	5.60E-13
4-Mar	0.054037708	0.36762737	2.7662058	6.87E-29	5.39E-28
UTAT33	0.156211549	0.399221669	1.35368891	6.00E-12	1.30E-11
CECR7	0.747218885	0.273513895	-1.449916793	4.65E-18	1.49E-17
NXPH4	0.631326212	11.81941047	4.226628618	1.77E-36	5.83E-35
JAML	0.704410566	2.284856423	1.697615058	2.38E-25	1.33E-24
SPDYA	0.075396623	0.249306077	1.725346245	1.64E-17	5.07E-17
PLEKHG3	7.362809597	3.651941473	-1.011592749	1.03E-29	8.86E-29
GABRE	0.390434599	3.042250423	2.961986102	1.14E-24	6.05E-24
LINC00884	0.441169488	0.889932169	1.012362364	9.65E-13	2.20E-12
AC007406.3	0.325605199	3.523161651	3.435675028	2.81E-37	1.26E-35
CD44	7.405462972	19.1090518	1.367594356	2.19E-18	7.19E-18
ILDR1	10.87732019	2.614332423	-2.056808667	2.18E-37	1.02E-35
ARMH1	0.172167801	0.35179813	1.030932458	4.12E-07	6.61E-07
AP000866.6	0.174191518	0.522762081	1.58548012	1.32E-10	2.63E-10
DUSP4	0.711342711	2.673226358	1.909965309	2.20E-29	1.82E-28
AC233728.1	0.234389245	0.505538677	1.108915098	1.53E-06	2.37E-06
MFSD4A	81.5697549	2.037774673	-5.322967878	3.52E-39	3.26E-37
IGHV2-26	1.17714612	7.622778442	2.69502353	7.49E-05	0.000103947
GLB1L	7.009834222	15.96044265	1.187048433	2.89E-19	1.01E-18
AC027601.2	0.223624711	0.666671988	1.575897493	1.32E-15	3.61E-15
HIST1H2AI	0.129501403	1.287822075	3.313893652	2.88E-23	1.36E-22
FMNL3	3.082178417	7.601080847	1.302254203	9.17E-30	7.93E-29
AC138956.2	0.50321061	1.650622201	1.713775705	7.23E-17	2.14E-16
RPL10P12	0.079287135	0.376699495	2.248255394	2.87E-13	6.74E-13
HMGA1P2	0.190026786	0.429516857	1.176511962	1.36E-07	2.25E-07
M1AP	0.922131493	0.271860063	-1.762108257	5.25E-15	1.38E-14
SCNN1G	63.76658065	2.217129484	-4.846035588	2.39E-41	7.04E-39
GPD1L	18.81618064	6.675052335	-1.495122762	1.23E-35	3.15E-34
ANKRD19P	0.219765113	0.482913197	1.135801513	1.81E-21	7.47E-21
CCL20	3.115283676	17.81231427	2.515439526	1.17E-18	3.89E-18
ITPRIPL1	0.168101055	0.588772803	1.808382251	1.99E-32	2.56E-31
ANO7	0.301949409	0.67476031	1.160068269	4.23E-28	3.04E-27
ATP1A1	684.389025	98.81102022	-2.792072767	7.64E-42	2.82E-39
WDR72	25.78748292	7.343593324	-1.812112888	1.36E-29	1.16E-28
MT1H	93.20017893	4.531718349	-4.362202621	1.49E-28	1.13E-27
SLC9A2	4.189831609	0.463075474	-3.177573007	9.79E-37	3.59E-35

Table S1. The differentially expressed genes(DEGs) were screened out in TCGA

ID	conMean	treatMean	logFC	pValue	FDR
SCARA5	0.903456468	0.242767964	-1.895877037	3.98E-16	1.12E-15
PPM1H	9.595256125	2.545904381	-1.914143081	2.21E-35	5.30E-34
RNA5SP82	0.455530874	1.507539396	1.726574963	3.85E-14	9.54E-14
AC020915.2	0.373869023	0.957978934	1.35746099	2.89E-11	5.99E-11
AC112496.1	0.192407958	0.58044059	1.592979942	1.88E-11	3.95E-11
NECTIN4	2.119452682	0.550493021	-1.944895577	2.69E-33	4.04E-32
ALDH4A1	68.44591693	16.63498608	-2.040743823	5.93E-19	2.02E-18
CLEC4E	0.929858017	2.397608152	1.366513546	2.07E-12	4.62E-12
TSPAN18	8.240333736	21.20830382	1.363854569	4.21E-21	1.69E-20
AC108471.1	0.02158927	0.256179994	3.568771465	5.41E-06	8.09E-06
KCNK17	0.065368599	0.594725957	3.185555357	3.56E-15	9.44E-15
LDHAP7	1.107217825	2.863830586	1.371007076	7.58E-10	1.44E-09
BTK	0.541992096	2.527741105	2.221504991	3.60E-33	5.30E-32
SEMA3B-AS1	6.09514734	0.821448215	-2.891419562	1.08E-36	3.89E-35
ADAMTS12	0.347625869	0.882727793	1.344433175	3.96E-10	7.65E-10
TLN2	11.85082897	5.583016619	-1.085871224	3.92E-26	2.36E-25
FNDC5	0.774011932	0.291229421	-1.410199697	1.04E-29	8.93E-29
LINC02294	8.955562275	2.121126305	-2.077953484	2.01E-11	4.20E-11
SLC16A10	1.837978289	0.648523139	-1.50288977	3.01E-28	2.20E-27
CD44-AS1	0.069181285	0.302843995	2.130121082	4.75E-10	9.13E-10
SNORD63	0.362167521	1.54759488	2.095298784	6.49E-14	1.59E-13
RNA5SP345	0.090715111	0.224013528	1.304171071	0.000100288	0.000137897
TPI1P2	1.226154872	0.423937875	-1.532216445	5.68E-33	8.03E-32
AC010618.2	0.09451756	0.433380676	2.196980538	1.73E-15	4.69E-15
ATP5MFP4	0.045101258	0.321704044	2.834494488	5.30E-15	1.39E-14
AL365277.1	0.092717326	0.27327864	1.559461828	2.47E-06	3.77E-06
RNY1P4	0.077964669	0.240111821	1.622814031	0.000638855	0.000831663
B3GNT10	2.398240667	1.015292497	-1.240081027	6.94E-31	6.93E-30
ZNF395	9.711918181	61.92087754	2.672597745	9.34E-36	2.49E-34
AC006272.1	0.036270779	0.25145554	2.793423683	1.37E-24	7.24E-24
SCGB1B2P	0.42333294	1.035865268	1.290971712	4.10E-16	1.16E-15
IGHV5-78	0.360377915	0.818904725	1.184185012	0.001326956	0.00169244
RPL10AP6	0.870664519	1.959570828	1.170348881	2.71E-17	8.27E-17
PCDH17	1.581885469	6.474528895	2.033130071	5.07E-26	3.02E-25
MEGF6	1.356934558	3.394784563	1.322968877	1.11E-14	2.86E-14
ZAP70	0.24077723	1.853038361	2.944121876	4.11E-34	7.30E-33
AC022113.1	0.084365464	0.237215212	1.491472091	2.47E-09	4.57E-09
MIR4257	0.038086649	0.396021823	3.378222681	5.07E-12	1.10E-11
TRBV6-1	0.15718123	0.85341478	2.440818147	6.70E-20	2.46E-19
SLC9A9	1.806797042	6.365325386	1.816799808	5.19E-28	3.68E-27
MYH3	0.168088951	0.469186646	1.480937055	3.96E-13	9.23E-13
MYO1F	0.810584683	4.694881575	2.534053948	1.29E-36	4.52E-35
ADCY7	0.313740974	1.251110423	1.995563267	1.74E-24	9.08E-24
CYP3A4	0.6968827	0.275412893	-1.339319743	1.47E-05	2.14E-05
ANKRD45	1.180298568	0.486159791	-1.279649369	7.97E-26	4.67E-25
AC100830.2	0.236398046	0.99661949	2.075824676	1.22E-17	3.80E-17
AC119396.1	0.134364075	0.319927322	1.251596744	1.79E-13	4.25E-13
NSUN5P2	0.188970961	0.475010263	1.329794134	1.47E-08	2.58E-08
ACOT11	4.084562972	1.04262638	-1.969959458	1.24E-36	4.35E-35
IGHV3-63	0.150805915	0.391706833	1.377081284	0.00205299	0.002578511
AC136604.2	0.075976137	0.364630913	2.262818607	1.94E-22	8.57E-22
RN7SL481P	0.11636316	0.494376254	2.086975073	2.44E-15	6.55E-15
SSBP3-AS1	0.110795454	0.257985453	1.21939103	2.55E-10	4.99E-10
AC007349.1	0.181622348	0.514545983	1.502358279	0.000144688	0.000196921
AC005703.6	0.084068749	0.288222238	1.777540136	2.55E-30	2.37E-29

Table S1. The differentially expressed genes(DEGs) were screened out in TCGA

ID	conMean	treatMean	logFC	pValue	FDR
C19orf18	0.34716265	0.710780652	1.033792671	3.58E-08	6.14E-08
EMP3	12.27501342	39.12582339	1.672396512	3.42E-32	4.18E-31
DEPTOR	31.59801042	10.66494454	-1.566957258	4.61E-40	6.87E-38
AC239859.6	0.662986087	2.607382667	1.975551833	6.32E-17	1.87E-16
AC011479.2	0.052126083	0.2685893	2.365324482	5.12E-18	1.64E-17
IGLV7-46	2.072737913	30.89589622	3.897805611	1.36E-06	2.11E-06
AL391427.1	0.192073715	0.391172674	1.026145489	1.43E-05	2.08E-05
AC232271.1	0.33276802	0.906635768	1.446006283	4.86E-14	1.20E-13
AC133134.1	0.119263263	0.321258513	1.429584967	5.78E-12	1.25E-11
ST6GALNAC	0.168335711	0.505284119	1.585753577	1.91E-16	5.50E-16
AC097372.3	0.143939511	0.33151334	1.203604264	9.92E-05	0.000136409
RNU6-652P	0.307088585	1.120865049	1.867885796	3.43E-10	6.66E-10
NACAP8	1.336140896	0.446838355	-1.580247216	3.05E-24	1.56E-23
AL450384.1	0.081069558	0.349933559	2.109848855	3.21E-15	8.53E-15
S100A12	0.65134735	1.634756885	1.327577085	1.50E-08	2.64E-08
SIGLEC8	0.134383141	2.851051209	4.407069888	4.56E-40	6.86E-38
FTH1P10	0.164690203	0.351921696	1.095499722	4.06E-13	9.47E-13
AD000864.1	0.08821762	0.337801391	1.937036524	1.51E-30	1.44E-29
ABHD11-AS1	0.579551177	3.120356187	2.428702754	2.61E-25	1.46E-24
CXCR2	0.247007653	0.498153889	1.012035743	1.48E-09	2.77E-09
NRM	4.844230958	10.11481593	1.06213051	1.34E-26	8.36E-26
AC148477.4	5.974009204	0.803677613	-2.89401066	2.37E-34	4.39E-33
SPRY4	9.079185681	22.37826269	1.301463227	1.22E-21	5.07E-21
HSD17B7	1.106739867	3.497367602	1.659953279	2.48E-32	3.13E-31
RPL17P50	1.064843056	3.543465949	1.734520375	1.14E-35	2.94E-34
LINC00924	6.000021076	2.21591726	-1.437063555	6.24E-22	2.66E-21
AL158166.1	0.192879231	0.553920296	1.521980595	2.90E-21	1.17E-20
BSND	15.37960198	1.253583121	-3.616888602	9.80E-40	1.24E-37
LAMA4	2.583336629	16.39142372	2.665633613	1.56E-36	5.28E-35
LCP1	9.375988889	33.71652484	1.84641308	1.98E-26	1.22E-25
AC131391.1	0.059004207	0.234061949	1.988000682	0.00913695	0.010935445
PCDH12	5.11562576	11.78776364	1.204307407	5.76E-16	1.61E-15
FAM118A	1.212160097	2.649837352	1.128323553	6.13E-20	2.25E-19
AC147651.3	0.555321331	2.88380415	2.376578471	3.13E-35	7.19E-34
AC108673.2	0.391170011	0.909587234	1.217416236	2.44E-23	1.16E-22
CXorf65	0.032662397	0.371218888	3.506567554	5.73E-26	3.39E-25
IGKV1OR2-6	0.204450282	0.831447206	2.02387461	0.00564961	0.006867461
AL139807.1	0.092183212	0.297407349	1.689864362	0.000420652	0.000554548
RNU6-339P	0.278813563	4.039082535	3.856654975	1.36E-30	1.31E-29
ABCB4	0.195752971	0.864833787	2.14338868	2.19E-18	7.19E-18
LINC01788	1.625362352	0.428770051	-1.922485337	2.63E-26	1.61E-25
FAM78A	0.573603294	3.025175948	2.398893839	1.88E-37	8.99E-36
EGR2	9.535756329	4.33014458	-1.138932174	7.85E-05	0.000108689
SH3BGR	6.136889264	2.724504117	-1.17151388	2.43E-36	7.58E-35
AC004847.1	0.11487841	0.917831515	2.998121655	2.53E-33	3.82E-32
SCARNA22	0.03139024	0.473814912	3.91593566	1.73E-10	3.42E-10
AC018638.2	4.187503931	11.82688941	1.497908231	3.39E-21	1.37E-20
FAM49A	1.325616379	3.935572954	1.56991035	5.67E-29	4.49E-28
FOXS1	1.948901399	5.715997555	1.552344198	1.21E-15	3.32E-15
IGHV4-34	4.934541898	27.00094745	2.452021969	9.99E-06	1.47E-05
HCG4B	0.435287967	1.790990774	2.040715863	9.38E-20	3.40E-19
AC009053.3	1.313678207	3.613814907	1.459910693	1.67E-11	3.52E-11
AL591468.1	0.014541152	0.282896719	4.28206202	1.82E-22	8.08E-22
AC026462.3	2.202735956	0.421615756	-2.385295881	6.99E-34	1.18E-32

Table S2. The immune-related DEGs were screened out in TCGA

ID	conMean	treatMean	logFC	pValue	FDR
AZGP1	41.87893129	12.40667375	-1.755108248	2.04E-06	3.13E-06
B2M	435.5191014	1104.585561	1.342697274	2.53E-33	3.82E-32
CD1A	0.039832605	0.278854276	2.807489666	3.80E-14	9.43E-14
CD1D	0.860631723	4.193998054	2.284858267	3.95E-36	1.15E-34
CD4	6.587828292	23.21248573	1.817026166	2.33E-30	2.17E-29
CD8A	0.902661944	11.31597051	3.648030728	7.87E-32	9.04E-31
CD8B	0.386198107	4.244911035	3.45832132	2.84E-29	2.33E-28
CD74	419.7033403	1411.712739	1.750004703	1.32E-30	1.27E-29
CTSE	0.268338036	1.576072224	2.554210179	4.03E-07	6.47E-07
CTSS	10.84665618	41.53150929	1.936955946	2.94E-29	2.40E-28
FCER1G	9.167727556	53.05744517	2.532919132	2.30E-36	7.23E-35
HLA-A	258.0919347	835.7994612	1.69527177	4.97E-38	2.96E-36
HLA-B	290.8432708	1113.972107	1.937399279	1.20E-39	1.42E-37
HLA-C	265.9387185	677.5405253	1.349213403	1.28E-32	1.71E-31
HLA-DMA	20.48890526	57.94869739	1.499933329	3.00E-30	2.76E-29
HLA-DMB	8.848303875	18.98307058	1.101240534	1.11E-16	3.24E-16
HLA-DOA	8.590774556	23.0765818	1.425569424	3.07E-14	7.66E-14
HLA-DOB	0.636122774	2.937564404	2.20724334	2.20E-20	8.32E-20
HLA-DPA1	33.19723619	121.3011518	1.869458208	1.23E-27	8.40E-27
HLA-DPB1	44.02344333	186.7294374	2.084605486	7.30E-32	8.46E-31
HLA-DQA1	8.914367764	42.15292427	2.241428333	2.25E-28	1.66E-27
HLA-DQA2	7.124018247	39.21800228	2.460752932	6.66E-14	1.63E-13
HLA-DQB1	14.35841368	71.73125957	2.320705595	4.98E-30	4.46E-29
HLA-DRA	343.1340806	1147.741158	1.741952989	6.26E-28	4.41E-27
HLA-DRB1	205.239071	640.0057864	1.640779549	4.50E-28	3.23E-27
HLA-DRB5	82.19951569	212.7735843	1.372117253	1.20E-14	3.07E-14
HLA-E	232.7418903	492.7907821	1.082244371	6.66E-31	6.67E-30
HLA-F	6.785350208	37.5298872	2.46754477	6.29E-40	8.58E-38
HLA-G	1.676810196	24.47187207	3.867333171	2.77E-32	3.44E-31
HLA-H	17.05623982	57.10324799	1.743273179	2.33E-32	2.96E-31
HSPA2	52.34832846	3.354695578	-3.963889711	2.22E-40	3.84E-38
HSPA6	0.654532543	2.747032921	2.069337371	2.90E-31	3.06E-30
ICAM1	11.38388336	29.8352402	1.390024607	7.81E-25	4.20E-24
IFNG	0.033548735	0.770130505	4.520772651	6.90E-30	6.08E-29
KIR2DL4	0.071827739	0.384075366	2.418776434	3.36E-28	2.44E-27
KLRC1	0.061161691	0.233621995	1.933475905	1.68E-20	6.45E-20
KLRD1	0.084454012	0.438419828	2.37607518	1.76E-36	5.78E-35
LTA	0.091767198	0.577242079	2.653126016	1.06E-27	7.30E-27
CIITA	1.230969225	3.498989709	1.507143727	2.29E-21	9.35E-21
MICB	0.94848986	2.373876505	1.323540629	6.65E-29	5.23E-28
PSMB8	23.5900901	72.09384719	1.61169521	1.60E-39	1.79E-37
RELB	4.108267917	8.908311619	1.116621757	1.70E-26	1.05E-25
TAP1	9.824433889	37.89533981	1.947574261	5.31E-39	4.45E-37
TAP2	3.191248139	7.1862553	1.171119398	4.42E-29	3.55E-28
TAPBP	27.74980472	84.23953798	1.602019905	2.87E-37	1.28E-35
IFI30	0.192021464	0.970552	2.337537931	3.39E-26	2.05E-25

Table S2. The immune-related DEGs were screened out in TCGA

ID	conMean	treatMean	logFC	pValue	FDR
PROCR	9.636000347	28.80449262	1.579787495	2.03E-29	1.68E-28
ERAP2	6.136530688	12.91520588	1.073575484	1.61E-07	2.66E-07
ULBP2	0.562564232	1.14788055	1.028882787	9.77E-17	2.87E-16
RAET1E	0.890929207	0.255746995	-1.800593513	2.61E-16	7.43E-16
PDIA2	0.03504275	0.332661282	3.246866049	0.00505491	0.006164218
HAMP	0.042262231	0.565979605	3.74330925	2.84E-29	2.33E-28
SLPI	166.7490311	48.27262438	-1.788401211	1.44E-18	4.78E-18
CXCL10	3.230415582	35.63265339	3.463408238	1.08E-31	1.21E-30
CXCL9	2.611677277	32.86933179	3.653693587	4.37E-32	5.23E-31
CXCL5	0.211824868	5.253037935	4.632208128	1.39E-10	2.77E-10
CXCL11	0.435641105	5.137675006	3.55990364	1.24E-30	1.20E-29
CXCL12	37.1814979	16.05792324	-1.21129957	6.88E-22	2.92E-21
CXCL13	0.265823874	5.028927296	4.241708112	1.86E-26	1.14E-25
CXCL2	2.548180188	7.809354085	1.61573593	8.04E-08	1.35E-07
PF4	0.194015017	0.417496776	1.105596737	0.000247616	0.000331853
XCL1	0.196233068	1.000537621	2.350135332	2.24E-23	1.07E-22
DEFB1	841.20163	207.3787319	-2.020183702	1.15E-34	2.29E-33
TMSB10	602.5835472	2548.010179	2.080137853	1.48E-35	3.71E-34
LCN2	8.599858558	3.208513648	-1.422407812	8.73E-21	3.41E-20
LCN1	0.004005765	0.39145799	6.610636044	9.49E-23	4.30E-22
S100A9	18.61200506	39.07580521	1.070042118	2.79E-10	5.44E-10
S100A8	3.659235781	10.48543314	1.518772175	2.03E-15	5.48E-15
S100A12	0.65134735	1.634756885	1.327577085	1.50E-08	2.64E-08
CCR10	0.194860055	0.623353335	1.677611777	1.94E-22	8.57E-22
PTGDS	37.2462633	15.73044518	-1.243536195	3.82E-14	9.48E-14
S100A2	44.94602435	3.644959574	-3.62421879	4.13E-39	3.64E-37
S100A5	0.95986352	0.320581968	-1.582136007	1.95E-28	1.45E-27
LMBR1L	2.073119292	5.576572156	1.427575455	1.29E-34	2.56E-33
TMSB4Y	1.793387248	0.580927978	-1.626255825	3.33E-10	6.46E-10
S100A14	12.84185961	2.839144884	-2.177325754	5.27E-35	1.15E-33
ZC3HAV1L	2.044624458	4.776817133	1.224213765	4.77E-20	1.77E-19
WFIKKN1	0.320360779	0.771844669	1.268613005	1.40E-05	2.04E-05
WFDC2	115.4656713	50.32470087	-1.198125395	1.26E-22	5.63E-22
TGFB1	14.84169029	43.80108268	1.561311124	8.28E-32	9.45E-31
PF4V1	0.720211486	2.991160317	2.054212723	8.05E-10	1.53E-09
MMP9	0.839206088	13.7903335	4.038488393	2.51E-25	1.40E-24
APOBEC3G	1.122554896	5.688964697	2.34138013	1.89E-35	4.60E-34
FABP6	0.390912925	19.45977802	5.637504156	1.50E-38	1.05E-36
NOD2	0.166210027	0.927548648	2.480415529	2.52E-31	2.68E-30
TLR2	2.401574331	7.391895057	1.621963813	4.19E-27	2.73E-26
PLAU	72.7867979	15.84399196	-2.199740923	1.85E-25	1.05E-24
PAEP	0.010618538	6.658253526	9.292414907	2.82E-13	6.64E-13
NOX4	13.94177603	5.867594815	-1.248573201	0.003417254	0.004223461
FABP7	1.387057525	84.19427879	5.923622676	5.27E-37	2.14E-35
FABP5	1.390030232	5.110482824	1.878343339	3.53E-28	2.56E-27
OASL	1.068942564	3.167356973	1.567095137	7.52E-24	3.71E-23

Table S2. The immune-related DEGs were screened out in TCGA

ID	conMean	treatMean	logFC	pValue	FDR
RBP2	2.037824387	0.139397015	-3.869758154	4.08E-36	1.18E-34
CETP	2.257560383	5.432347509	1.266811196	2.15E-18	7.06E-18
C8G	0.339359283	1.155214376	1.767275217	3.66E-09	6.69E-09
APOD	10.8744237	3.910502721	-1.475512954	8.00E-23	3.64E-22
ORM2	0.076579212	2.804391432	5.194591116	7.94E-13	1.82E-12
ORM1	0.132598528	3.962466698	4.90126214	0.000614203	0.000800583
PRTN3	0.040807546	0.25283187	2.631270478	1.24E-10	2.47E-10
PML	4.127491986	9.57467159	1.21395759	3.33E-36	9.89E-35
AEN	2.457039503	6.439466189	1.390020046	2.01E-36	6.48E-35
CYBB	5.948485569	21.40530795	1.847374268	1.71E-20	6.53E-20
ISG20	0.41655104	2.125562903	2.351279771	2.86E-36	8.70E-35
TLR3	5.539796903	16.01348697	1.531382501	3.44E-24	1.74E-23
IDO1	1.222919186	12.36748266	3.338150901	2.43E-36	7.58E-35
GDF15	79.55718229	34.44815336	-1.207565533	1.94E-16	5.58E-16
CCL20	3.115283676	17.81231427	2.515439526	1.17E-18	3.89E-18
IRF1	5.463305597	12.01400087	1.136870644	3.27E-20	1.22E-19
IL15	0.639172143	1.312437882	1.037972703	4.94E-23	2.29E-22
APOBEC3F	1.222887569	2.648046756	1.114636825	3.68E-30	3.36E-29
RARRES3	34.38991556	80.1438001	1.220605345	1.57E-15	4.26E-15
CHIT1	0.077507872	2.431162175	4.971159477	1.23E-21	5.10E-21
CD40	6.298331111	22.51214435	1.837661976	6.24E-36	1.74E-34
TLR7	0.653760812	2.853060323	2.125675444	1.31E-24	6.92E-24
VEGFA	10.79031619	125.7155178	3.542353694	2.71E-37	1.23E-35
ISG15	13.7994606	40.46061088	1.551906227	8.66E-26	5.06E-25
TNFAIP3	6.156998153	15.3819319	1.320937669	3.51E-25	1.94E-24
TFR2	0.048947392	0.754157325	3.945561616	5.32E-29	4.22E-28
LYZ	20.62648308	114.1078376	2.467828128	1.72E-24	8.96E-24
CCL5	5.452020633	51.86301717	3.249843211	5.05E-35	1.11E-33
KLKB1	0.646815801	1.766200451	1.449222259	3.31E-08	5.70E-08
TLR8	0.398471467	1.794289803	2.170864597	2.55E-23	1.21E-22
GNLY	0.490162003	4.338098701	3.145732321	4.28E-37	1.79E-35
APOM	38.40334376	16.86765048	-1.186972899	0.00079868	0.001033839
BST2	54.71206903	134.9449007	1.302439442	7.78E-23	3.54E-22
BPHL	10.42599381	3.653792286	-1.512718382	1.49E-21	6.15E-21
PDGFRA	7.540041465	1.449602308	-2.3789153	9.55E-32	1.08E-30
GNAI1	23.59326063	10.08993414	-1.225458057	1.09E-25	6.33E-25
WNT5A	2.707965281	1.072318992	-1.336475101	4.91E-13	1.14E-12
NOS2	0.59413861	1.204800056	1.019922293	0.002423956	0.003028364
MSR1	1.976159253	8.485425345	2.102287765	9.49E-28	6.54E-27
DLL4	3.828581707	20.8556227	2.445554437	7.87E-32	9.04E-31
SLC11A1	0.304216049	1.604221745	2.398705405	1.83E-33	2.83E-32
TMEM173	9.211673472	19.02865531	1.046638437	6.75E-23	3.09E-22
DES	21.21334589	7.238267128	-1.551255933	2.96E-11	6.13E-11
TNFRSF10B	7.282567361	20.89170975	1.520411519	2.66E-37	1.22E-35
CCL4	1.353069597	8.732162588	2.690102944	1.84E-32	2.39E-31
APOBEC3H	0.215014276	1.599652516	2.895254191	3.68E-36	1.08E-34

Table S2. The immune-related DEGs were screened out in TCGA

ID	conMean	treatMean	logFC	pValue	FDR
TMPRSS6	0.069317915	0.295344512	2.091098637	5.06E-13	1.17E-12
SPINK5	0.21438913	0.944449288	2.139241573	1.19E-07	1.97E-07
MARCO	0.513380503	2.577398728	2.327815329	7.27E-11	1.47E-10
TNFSF11	0.055632971	0.250587558	2.171302735	5.90E-05	8.23E-05
KNG1	288.1662501	2.998896111	-6.586326023	1.30E-41	4.16E-39
KLRK1	0.029883865	0.326691405	3.450489826	7.38E-29	5.76E-28
CLDN4	49.91557688	21.58078602	-1.209742688	1.08E-13	2.61E-13
CCL28	2.527795814	7.263620979	1.522808991	1.43E-15	3.90E-15
IRF7	3.246384853	10.86632612	1.742958307	1.53E-29	1.29E-28
LTB4R	0.519578019	3.04305765	2.550109361	4.49E-38	2.70E-36
APOBEC3A	0.196572769	0.449295121	1.192599918	1.89E-12	4.21E-12
IL7R	2.286850316	5.217229036	1.189921831	6.31E-14	1.54E-13
IFNAR2	2.667835708	5.754906838	1.109122749	2.94E-29	2.40E-28
SYTL1	0.372823383	1.091240262	1.549404527	6.52E-11	1.32E-10
APOBEC3C	5.735219014	24.61184262	2.101432187	5.44E-32	6.44E-31
PTGS2	5.727144401	2.5058454	-1.19251857	2.04E-14	5.15E-14
CD40LG	0.375650774	1.065211627	1.503676102	2.32E-18	7.59E-18
CD14	18.80520831	62.83544718	1.740446365	1.94E-27	1.31E-26
MASP1	0.967176061	4.030233341	2.059012928	4.09E-19	1.41E-18
PROC	5.261353549	1.422607443	-1.886896381	2.92E-21	1.18E-20
HRG	10.93005619	2.354339139	-2.21490676	7.84E-42	2.83E-39
NDRG1	92.62849931	239.8847846	1.372813608	1.15E-26	7.21E-26
IRF9	0.563746139	1.906369183	1.757709983	2.67E-19	9.36E-19
TRIM22	6.872980139	18.10588291	1.397450834	3.65E-27	2.39E-26
HMOX1	24.87096883	128.4014111	2.368126444	1.97E-32	2.54E-31
BACH2	0.484030686	0.230260188	-1.071833522	2.02E-21	8.30E-21
PIK3CG	0.760539903	1.849939691	1.282382391	1.84E-14	4.65E-14
PDCD1	0.257014739	2.666049357	3.374780489	3.86E-26	2.32E-25
ARG2	37.12929571	9.539183227	-1.960620301	2.69E-27	1.79E-26
AQP9	0.912599834	4.128871543	2.177693238	1.69E-16	4.89E-16
FASLG	0.139840728	1.632608074	3.545321997	3.64E-35	8.23E-34
BIRC5	0.401040877	2.170156096	2.435977619	9.38E-34	1.54E-32
VIM	109.0103189	639.5199219	2.552524595	4.86E-37	2.00E-35
VCAM1	29.0877245	103.6318576	1.832985227	8.23E-22	3.48E-21
GFAP	0.145218033	0.371593389	1.355504216	0.035658299	0.040710534
GBP2	6.270769403	24.73639289	1.979920766	9.52E-35	1.94E-33
ALB	54.80645898	21.52170558	-1.348553511	5.03E-16	1.41E-15
OAS1	4.888371375	13.44938515	1.460114423	8.67E-30	7.50E-29
AGER	0.735303564	3.212644635	2.127349524	5.81E-26	3.44E-25
NOS1	1.580688498	0.08064948	-4.292744047	1.54E-29	1.30E-28
ACO1	25.11522347	12.09053945	-1.054683497	1.86E-24	9.68E-24
CCL18	0.240432572	12.12416365	5.656109063	1.08E-30	1.06E-29
CCL22	0.319994012	0.673709132	1.074080948	5.63E-12	1.22E-11
CCL4L2	1.021498395	6.129015295	2.584968368	9.51E-22	3.99E-21
CCR7	0.523290493	1.614489439	1.625394051	1.00E-19	3.62E-19
CCR8	0.036456592	0.258426905	2.825504683	3.29E-22	1.43E-21

Table S2. The immune-related DEGs were screened out in TCGA

ID	conMean	treatMean	logFC	pValue	FDR
CCL3	1.57382809	5.668468198	1.848680963	8.77E-18	2.75E-17
CCL11	2.321754935	0.411467152	-2.496366534	4.10E-28	2.95E-27
CCR5	0.782307784	5.954670484	2.92821345	9.31E-32	1.06E-30
CCL23	1.164984526	0.425394819	-1.453436424	3.04E-14	7.58E-14
CCL3L1	0.782775898	2.583180855	1.722477412	1.35E-10	2.68E-10
CCR1	2.950080943	6.545957196	1.14984963	1.20E-11	2.55E-11
XCL2	0.332617371	1.964680643	2.562359401	1.73E-27	1.17E-26
CXCR4	13.70918847	94.82623863	2.790143139	4.33E-39	3.76E-37
CXCR6	0.308036263	2.060846095	2.742064663	1.85E-31	2.01E-30
CCR4	0.37777026	1.049633981	1.474305297	4.56E-13	1.06E-12
FAM19A5	0.619465019	1.696229581	1.453236728	7.27E-11	1.47E-10
CDH1	53.0348044	18.82839958	-1.494029072	5.83E-31	5.89E-30
IL10	0.200087059	0.50553959	1.337196218	3.56E-12	7.82E-12
FGR	1.999401647	5.741111935	1.521761869	1.98E-28	1.47E-27
MIF	23.23782518	51.43787337	1.146355944	1.44E-20	5.55E-20
CRP	0.039230023	4.083626221	6.701748816	5.53E-10	1.06E-09
PTGDR	0.125898207	0.600976753	2.255051445	7.67E-30	6.68E-29
CD86	1.224751815	5.447647688	2.153143974	1.68E-30	1.60E-29
HCK	2.050127786	8.59168049	2.067226506	3.51E-33	5.17E-32
VDR	10.8537089	3.359952172	-1.691675519	2.80E-23	1.32E-22
RNASE2	0.376079281	2.019287882	2.424737872	1.50E-26	9.35E-26
CD79A	1.308517064	4.445079049	1.764276337	1.02E-09	1.92E-09
BTK	0.541992096	2.527741105	2.221504991	3.60E-33	5.30E-32
BLNK	9.552324958	4.287332507	-1.155771605	2.01E-29	1.67E-28
VAV3	27.17348351	10.44541001	-1.379330401	3.61E-35	8.17E-34
VAV1	0.70746699	3.586581249	2.341874574	5.87E-34	1.01E-32
RAC2	4.125681333	17.26656379	2.065276704	3.53E-29	2.86E-28
NFATC2	1.54546936	3.506266326	1.181890531	8.23E-23	3.74E-22
CARD11	0.949591299	4.033692469	2.086722476	6.80E-24	3.36E-23
NFKBIE	6.618898931	15.23765777	1.202978012	2.66E-26	1.62E-25
CD19	0.134982263	0.29865017	1.145686696	0.001834211	0.002312618
CR2	2.50528243	0.183293138	-3.77274857	1.14E-28	8.71E-28
PIK3R5	0.361425004	2.584320304	2.838016668	1.00E-37	5.28E-36
INPP5D	1.973086658	6.462947246	1.711737895	7.42E-34	1.25E-32
CD22	0.342610763	0.811314312	1.243690464	2.79E-10	5.44E-10
CD72	0.362359888	2.408438665	2.732603018	1.21E-36	4.31E-35
LILRB3	0.191575925	1.056059909	2.462703506	3.19E-35	7.31E-34
FCGR2B	0.396042087	1.586283661	2.001925122	8.50E-24	4.18E-23
RASGRP3	2.125646417	4.549844282	1.097915533	1.03E-25	5.97E-25
IGHA1	110.5818066	382.7663462	1.791349943	1.13E-08	2.00E-08
IGHA2	13.1693102	57.92937063	2.137115211	1.60E-08	2.81E-08
IGHD	3.47977236	7.861654315	1.175839998	0.001315795	0.001678622
IGHE	0.09999659	0.353360308	1.821189196	0.002436529	0.003043702
IGHG1	45.43358152	363.3513878	2.999534474	4.06E-07	6.52E-07
IGHG2	53.540109	210.2353181	1.973313072	5.58E-06	8.34E-06
IGHG3	12.89760276	121.8557209	3.239999137	6.32E-08	1.07E-07

Table S2. The immune-related DEGs were screened out in TCGA

ID	conMean	treatMean	logFC	pValue	FDR
IGHG4	25.72455506	57.58469046	1.162539182	0.001218361	0.001558182
IGHJ2	0.929418008	5.149062181	2.469910189	0.000138464	0.000188725
IGHJ3	1.827186476	10.7568777	2.557563597	1.56E-06	2.42E-06
IGHM	27.09973814	110.5711665	2.028624408	2.61E-09	4.82E-09
IGHV1-18	8.956996947	55.70513736	2.636723366	2.05E-06	3.15E-06
IGHV1-2	11.49579373	26.4907642	1.204383382	0.000434522	0.000572394
IGHV1-24	5.155594012	33.68942038	2.708085042	0.0007009	0.000910246
IGHV1-3	0.223389594	1.822157704	3.028013936	1.16E-05	1.69E-05
IGHV1-45	0.275815979	1.177627542	2.094105374	0.000269995	0.000360928
IGHV1-46	2.743081623	15.71182552	2.517981356	3.83E-06	5.78E-06
IGHV1-58	0.889514594	3.047616159	1.776591029	0.024968837	0.028874819
IGHV1-69	1.61452826	9.229171318	2.515088421	7.28E-05	0.000101045
IGHV2-26	1.17714612	7.622778442	2.69502353	7.49E-05	0.000103947
IGHV2-5	1.626936919	3.448719093	1.083902308	0.00297793	0.003695355
IGHV2-70	1.656294636	12.97103667	2.969262547	0.000891782	0.001150881
IGHV3-11	5.841501167	21.9016924	1.906631285	7.33E-05	0.000101761
IGHV3-15	8.033585604	37.07454247	2.206312939	2.80E-05	3.99E-05
IGHV3-20	0.342937209	1.811452692	2.401130777	9.87E-06	1.45E-05
IGHV3-21	4.432468157	31.22557114	2.816545788	2.22E-06	3.40E-06
IGHV3-23	15.06798929	75.58112064	2.326538996	9.28E-06	1.37E-05
IGHV3-30	6.065975761	54.654816	3.171536989	2.24E-07	3.65E-07
IGHV3-33	4.261851907	20.08033199	2.236230754	5.44E-06	8.13E-06
IGHV3-35	0.150863298	0.560083236	1.892399377	0.000404838	0.000534386
IGHV3-38	0.087050101	0.509610854	2.54947813	3.37E-05	4.77E-05
IGHV3-43	1.233913283	4.604851501	1.899913624	8.90E-05	0.000122652
IGHV3-48	1.24586632	9.322350761	2.903544519	1.91E-06	2.94E-06
IGHV3-49	3.019982567	17.28818116	2.517173969	6.01E-06	8.96E-06
IGHV3-53	2.274194994	10.78057039	2.245005647	0.000946178	0.0012194
IGHV3-64	0.367105422	2.127335659	2.534781359	8.66E-06	1.28E-05
IGHV3-66	1.470538824	7.11600166	2.274721973	9.28E-05	0.000127874
IGHV3-7	0.800938598	2.448886886	1.612362587	0.002168932	0.002718824
IGHV3-72	1.79127637	5.567548948	1.636054394	0.00260664	0.003247927
IGHV3-73	1.720516429	20.65981033	3.585913436	0.001871801	0.002357991
IGHV4-28	0.940814095	5.01157033	2.41328115	0.000101513	0.000139507
IGHV4-31	2.635738927	15.81837258	2.585321798	6.65E-05	9.25E-05
IGHV4-34	4.934541898	27.00094745	2.452021969	9.99E-06	1.47E-05
IGHV4-39	10.18009334	58.14495383	2.513903202	0.000176906	0.000239455
IGHV4-4	0.596255098	2.784694064	2.223517237	0.000209519	0.000282045
IGHV4-59	5.931061566	31.93591725	2.428817635	2.74E-05	3.91E-05
IGHV4-61	0.880962227	3.013112678	1.774102557	0.001377879	0.001755214
IGHV5-51	11.71673263	56.41247575	2.267443943	6.29E-05	8.77E-05
IGHV6-1	0.342533854	2.469780651	2.850064429	3.39E-06	5.14E-06
IGKC	69.86566755	432.7235856	2.630790172	4.55E-08	7.76E-08
IGKJ5	0.511897653	4.008572135	2.96916114	1.19E-06	1.85E-06
IGKV1-12	0.736419297	1.767910023	1.263445516	0.000522011	0.000684054
IGKV1-16	3.66623047	19.33284086	2.398684264	4.61E-06	6.93E-06

Table S2. The immune-related DEGs were screened out in TCGA

ID	conMean	treatMean	logFC	pValue	FDR
IGKV1-17	4.290085233	22.96411421	2.42030292	2.85E-05	4.06E-05
IGKV1-27	3.305946873	12.32980267	1.899014265	8.85E-05	0.000122034
IGKV1-33	0.068910751	0.536434485	2.960601003	0.00294533	0.003656002
IGKV1-39	0.174729916	1.194486311	2.773191775	0.010642987	0.012665037
IGKV1-5	22.94765016	75.37975577	1.715830692	0.00030553	0.000406846
IGKV1-6	3.616319605	13.51034496	1.90147042	0.000454082	0.000597166
IGKV1-8	0.532781266	2.333930189	2.131146148	5.49E-05	7.68E-05
IGKV1-9	6.249610452	34.40638203	2.460838023	3.77E-05	5.33E-05
IGKV1D-12	0.143719423	0.569367598	1.986105347	0.031252317	0.035821328
IGKV1D-13	0.561049656	1.47929134	1.398705846	0.004004301	0.004920355
IGKV1D-16	0.38308698	1.78084432	2.216817506	4.62E-05	6.50E-05
IGKV1D-17	0.20194485	0.553245967	1.453959673	0.001886806	0.002375875
IGKV1D-39	0.06654096	0.620356125	3.220782062	0.000371867	0.000492062
IGKV1D-42	0.118195626	0.339532339	1.522372348	0.024474073	0.028328116
IGKV1D-43	0.095152802	0.647564283	2.766705364	0.009653354	0.011530707
IGKV1D-8	0.557545693	1.837839128	1.720848539	5.76E-06	8.60E-06
IGKV2-24	2.562533482	15.65721317	2.61118469	5.47E-05	7.64E-05
IGKV2-28	0.094094636	1.795693071	4.254284486	5.46E-05	7.63E-05
IGKV2-30	0.783076669	1.979691728	1.338050325	0.000794108	0.001028116
IGKV2D-24	0.15442559	0.568533003	1.88033226	0.000181221	0.000245071
IGKV2D-29	1.152520884	6.769730069	2.554305417	6.51E-06	9.69E-06
IGKV2D-30	0.068049783	0.267724693	1.976087753	0.008832521	0.010584014
IGKV2D-40	0.591343743	2.611844381	2.143000037	0.009066488	0.010856163
IGKV3-11	16.25485653	79.24572673	2.285462318	1.83E-06	2.82E-06
IGKV3-15	5.805712656	39.27893908	2.758210888	1.23E-06	1.92E-06
IGKV3-20	36.32660977	160.4613739	2.14312742	3.99E-05	5.62E-05
IGKV3-7	0.517787288	1.660191768	1.680918443	2.58E-06	3.94E-06
IGKV3D-11	0.44319516	1.917625878	2.113307251	7.49E-05	0.000103913
IGKV3D-15	0.737091759	2.109140634	1.516739162	0.002103146	0.002639253
IGKV3D-20	1.856870892	6.156037195	1.72912844	0.000420348	0.000554184
IGKV4-1	22.3848872	97.57261536	2.123951251	4.39E-06	6.60E-06
IGKV5-2	0.547297696	5.30014141	3.275633164	0.000453944	0.000597023
IGKV6-21	1.03289805	3.527769219	1.772058322	9.15E-05	0.00012603
IGKV6D-21	0.40939284	1.818111987	2.15088329	0.001771069	0.002235608
IGLC2	44.5174044	228.6131896	2.360467257	4.17E-09	7.58E-09
IGLC3	27.01878303	171.6655449	2.667565904	1.29E-08	2.28E-08
IGLC6	0.119058536	0.601370793	2.336583745	3.22E-05	4.57E-05
IGLC7	0.960335685	6.227441905	2.697028965	7.99E-06	1.18E-05
IGLJ2	0.114384091	0.39731721	1.796404877	0.00885332	0.01060832
IGLV1-36	1.152643858	4.21611198	1.870966366	0.000194511	0.000262442
IGLV1-40	13.55344794	177.9843242	3.715018364	3.31E-07	5.35E-07
IGLV1-44	11.27860858	48.7341877	2.111345102	2.07E-06	3.17E-06
IGLV1-47	6.388097263	36.16840818	2.50127192	5.42E-07	8.63E-07
IGLV1-50	0.15383399	0.667502541	2.117399024	0.000311541	0.000414641
IGLV1-51	14.28230035	57.20029646	2.001794261	8.59E-08	1.44E-07
IGLV10-54	1.894357916	7.256751497	1.93761493	9.55E-05	0.000131496

Table S2. The immune-related DEGs were screened out in TCGA

ID	conMean	treatMean	logFC	pValue	FDR
IGLV2-11	10.21579816	39.00749926	1.932949584	2.11E-06	3.24E-06
IGLV2-14	23.7326758	98.57276775	2.054314365	4.44E-06	6.67E-06
IGLV2-18	1.164087098	5.19661036	2.158371884	6.29E-05	8.76E-05
IGLV2-23	14.18486224	75.80777859	2.417993751	1.19E-06	1.85E-06
IGLV2-8	4.389469195	17.26615716	1.97582863	6.16E-07	9.79E-07
IGLV3-1	10.99084213	38.42420146	1.805713347	5.28E-07	8.42E-07
IGLV3-10	4.086395064	36.00517493	3.139303683	1.93E-05	2.78E-05
IGLV3-12	0.107528232	0.512671375	2.253318853	3.58E-08	6.15E-08
IGLV3-16	0.238380436	3.446111105	3.853631475	0.000351268	0.000465644
IGLV3-19	14.18982428	123.4008551	3.120423763	7.61E-07	1.20E-06
IGLV3-21	12.38973266	56.10132689	2.178889836	5.09E-06	7.63E-06
IGLV3-25	10.55332712	46.96425583	2.153865244	2.14E-07	3.49E-07
IGLV3-27	0.951204104	4.225916517	2.151437422	0.000463089	0.000608739
IGLV3-9	1.652075363	8.349854986	2.337471642	2.18E-05	3.13E-05
IGLV4-3	0.073914308	0.297659233	2.00973609	0.009128675	0.010927446
IGLV4-60	0.986003858	7.428199202	2.913347308	0.000295687	0.000394199
IGLV4-69	4.600324245	36.05767816	2.970499046	0.000373714	0.000494411
IGLV5-37	0.266608137	0.842445289	1.659862183	0.004241025	0.00520223
IGLV5-45	1.532065772	5.745637537	1.90698875	1.51E-05	2.20E-05
IGLV5-48	0.112185317	0.493044874	2.13583509	0.004539569	0.005554868
IGLV5-52	0.050711024	0.3164373	2.641548368	4.58E-09	8.30E-09
IGLV6-57	4.468005686	20.71576499	2.21302617	2.28E-06	3.49E-06
IGLV7-43	1.955734998	8.688353114	2.15137184	1.33E-05	1.94E-05
IGLV7-46	2.072737913	30.89589622	3.897805611	1.36E-06	2.11E-06
IGLV8-61	4.347945808	20.09799394	2.208645644	2.13E-05	3.06E-05
IGLV9-49	0.825740822	24.64058859	4.899203879	1.07E-06	1.67E-06
C3	22.99807194	235.7467393	3.357652995	2.34E-33	3.56E-32
C5	1.98548505	0.974544859	-1.026690998	2.91E-32	3.60E-31
CKLF	2.729053529	5.792647343	1.085822144	2.45E-30	2.28E-29
CX3CL1	20.38310458	41.15719871	1.013770984	3.02E-18	9.80E-18
EDN1	9.967852014	34.23102498	1.779949937	4.86E-18	1.55E-17
SEMA3A	0.189572838	0.41963461	1.146381405	0.002418253	0.003021606
SEMA3B	11.76148754	4.609254218	-1.351465292	1.93E-28	1.44E-27
SEMA3F	11.20445658	24.62551571	1.136081258	6.10E-24	3.04E-23
SEMA3G	18.17338861	6.023849073	-1.593069921	5.15E-30	4.60E-29
SEMA4A	9.113818778	3.051167014	-1.578694532	9.06E-22	3.81E-21
SEMA5B	2.082418883	27.6203172	3.729397684	2.77E-32	3.44E-31
SEMA6A	2.831858922	10.95869285	1.952254424	2.56E-29	2.11E-28
SEMA6B	2.768146275	8.425802233	1.605893874	1.53E-25	8.75E-25
SEMA6D	9.514390299	1.26975477	-2.905561315	1.35E-37	6.79E-36
SLIT2	4.282229282	1.86475961	-1.199372382	9.51E-14	2.30E-13
TNC	32.0911616	15.00162227	-1.09705749	7.32E-07	1.16E-06
TYMP	4.304598149	33.13173079	2.944263104	4.69E-37	1.94E-35
C5AR1	3.605503958	9.359148153	1.376176299	3.16E-23	1.49E-22
CCRL2	0.87396559	2.426985188	1.47351692	3.50E-29	2.84E-28
CX3CR1	2.027509138	6.70631854	1.725812594	1.66E-17	5.13E-17

Table S2. The immune-related DEGs were screened out in TCGA

ID	conMean	treatMean	logFC	pValue	FDR
CXCR3	0.371623525	3.382527768	3.18618804	3.52E-31	3.66E-30
CYSLTR1	0.476301081	1.090941784	1.195628388	7.06E-18	2.23E-17
EDNRA	3.748537333	8.923995553	1.251362024	4.11E-18	1.32E-17
FPR1	1.842032599	5.525870812	1.584903241	4.13E-17	1.24E-16
FPR2	0.163058047	0.412641538	1.339503416	1.95E-11	4.09E-11
LTB4R2	0.168818613	0.802658086	2.249311586	9.46E-34	1.56E-32
PLAUR	1.890214832	5.432327378	1.523020213	2.41E-21	9.86E-21
PLXNA3	1.441355619	3.711254294	1.364480528	2.58E-29	2.12E-28
PLXNA4	1.030777932	0.183567644	-2.48934986	4.03E-33	5.89E-32
PLXNB1	19.20313639	7.242743757	-1.406733721	5.13E-36	1.46E-34
PLXNB3	0.12341833	0.500386446	2.019486028	8.29E-15	2.14E-14
PLXNC1	1.1945283	3.515213769	1.557171392	2.64E-23	1.25E-22
PLXND1	9.233672639	28.41906014	1.621882353	6.88E-31	6.89E-30
PTAFR	1.975882161	5.364454696	1.440934618	2.14E-19	7.57E-19
XCR1	0.212447169	0.545847427	1.361393634	1.55E-09	2.90E-09
ADM	12.07183515	80.42273438	2.735958378	4.95E-37	2.02E-35
AGT	23.32483383	50.60252513	1.117342576	9.34E-09	1.66E-08
AMH	0.060116315	0.39390047	2.712002652	5.55E-17	1.65E-16
ANGPTL7	0.444157062	0.212461096	-1.063871234	2.38E-13	5.63E-13
APLN	6.064477011	33.99312623	2.486787907	5.53E-33	7.87E-32
BDNF	0.143576146	0.633485553	2.141495643	5.45E-13	1.26E-12
BMP1	2.151834175	7.837272629	1.864784779	2.28E-33	3.48E-32
BMP5	0.843819914	0.349110677	-1.273250655	9.95E-07	1.56E-06
BMP6	14.4672776	3.283360442	-2.139548426	2.46E-19	8.63E-19
BMP7	3.334839914	0.378357432	-3.139795818	1.94E-37	9.23E-36
BMP8A	0.213770845	0.581148867	1.442842664	3.12E-15	8.30E-15
BMP8B	0.629849776	1.481873568	1.234342682	3.49E-09	6.39E-09
BTC	4.092454764	1.157579386	-1.821855334	2.26E-36	7.14E-35
CALCA	8.634989161	1.194736171	-2.853502298	2.69E-38	1.76E-36
CAT	123.5289486	51.07843203	-1.274063028	2.08E-33	3.19E-32
CD70	1.815810682	36.13037075	4.31452636	9.13E-36	2.45E-34
CHGB	7.505851206	0.56755816	-3.725175547	2.27E-39	2.34E-37
CMTM2	0.161999704	0.368444491	1.185456106	1.31E-11	2.77E-11
CMTM3	5.356410861	15.09815297	1.495033537	1.47E-29	1.24E-28
CMTM4	27.75082024	7.570558575	-1.874058759	2.03E-37	9.54E-36
CSF1	7.164363264	14.4531964	1.012478193	4.67E-22	2.01E-21
CTF1	7.684911203	3.378299894	-1.185731187	1.26E-26	7.89E-26
CTGF	190.8310664	83.9272039	-1.185085632	1.30E-19	4.66E-19
EBI3	0.982246128	4.88358894	2.31378529	1.62E-33	2.53E-32
EGF	74.50253505	1.589919098	-5.550264254	3.88E-38	2.40E-36
EPO	0.962355036	8.855615117	3.201951379	9.26E-09	1.65E-08
ESM1	8.264372046	103.5572073	3.647378951	2.79E-37	1.26E-35
FAM3B	6.902502356	0.430629103	-4.002601748	2.02E-40	3.58E-38
FGF1	26.41248142	1.479408148	-4.158127816	3.23E-31	3.40E-30
FGF14	0.145720211	0.318787333	1.12939332	0.031064472	0.035611968
FGF7	1.747524878	0.800011909	-1.127219613	6.85E-17	2.03E-16

Table S2. The immune-related DEGs were screened out in TCGA

ID	conMean	treatMean	logFC	pValue	FDR
FGF9	9.149162983	1.075261352	-3.088952401	1.56E-36	5.28E-35
GDF3	2.173530581	0.470366337	-2.208183676	1.58E-06	2.44E-06
GDF6	0.37890828	4.544392649	3.584166923	2.78E-29	2.27E-28
GDF7	2.248676313	0.819640679	-1.456012514	1.48E-32	1.95E-31
GMFG	6.790638792	24.41895476	1.846382248	2.94E-35	6.83E-34
GNRH1	0.178043116	1.28296754	2.849186111	1.65E-31	1.80E-30
GREM1	2.026311349	0.361915718	-2.485130195	7.46E-07	1.18E-06
IL11	1.442833319	0.134805611	-3.419952193	6.57E-30	5.79E-29
IL16	0.589597089	2.210966514	1.906875869	8.63E-32	9.84E-31
IL24	0.398831635	0.874172873	1.132138761	6.65E-19	2.26E-18
IL32	21.53573967	83.82719765	1.960685526	2.32E-28	1.71E-27
IL34	1.862572189	4.495850981	1.271297875	9.75E-13	2.22E-12
IL7	0.703906974	1.626606139	1.20840828	1.69E-24	8.84E-24
INHBB	1.785995579	25.1763071	3.817266267	5.40E-38	3.17E-36
INHBE	0.114432542	1.186352414	3.373963352	7.23E-18	2.29E-17
INSL3	0.166406688	0.447177734	1.426134936	5.98E-11	1.21E-10
JAG2	2.464757322	7.855815598	1.672315458	2.89E-27	1.92E-26
KITLG	21.52851915	6.954415999	-1.630247813	3.34E-31	3.50E-30
KL	53.4211564	18.70037734	-1.514343825	3.60E-18	1.16E-17
LTB	3.023133071	6.293113535	1.057729487	3.69E-08	6.34E-08
MDK	7.52182584	24.04585735	1.676633558	3.42E-08	5.88E-08
MSTN	0.111334463	0.308371399	1.469768729	5.41E-09	9.78E-09
NGF	1.724507074	7.614480068	2.142561483	7.05E-25	3.80E-24
NMB	4.496406361	26.26324022	2.54620059	1.59E-31	1.74E-30
NRG3	0.320035543	1.272436077	1.99128914	1.12E-29	9.65E-29
NTS	1.858870504	0.837367851	-1.150492836	0.00104296	0.001339768
OGN	3.634924457	1.646940747	-1.142136731	6.32E-16	1.76E-15
OSM	0.525026197	1.919826635	1.870514724	9.11E-15	2.35E-14
PDGFD	6.480145444	24.03746681	1.891186766	1.07E-23	5.23E-23
PDGFRL	0.789984438	1.8177989	1.202296467	0.001513956	0.001922964
PGF	1.895384371	29.09362359	3.940140642	1.71E-35	4.23E-34
PMCH	0.011450176	0.323610897	4.820818461	5.80E-27	3.73E-26
PTHLH	0.312913128	14.43224251	5.527389487	6.30E-36	1.75E-34
REG1A	5.419557857	37.31737496	2.783600443	2.82E-10	5.51E-10
RETN	0.080380331	0.512194119	2.671776271	1.36E-19	4.88E-19
SCG2	0.206756901	3.342029928	4.014717263	1.19E-27	8.15E-27
SCGB3A1	0.2821962	0.891585871	1.659675191	2.78E-13	6.54E-13
STC2	1.485266486	23.58730573	3.989216928	4.29E-38	2.62E-36
TAC1	0.879882936	0.111907125	-2.975009697	7.19E-33	9.97E-32
TDGF1	4.941875346	0.500182846	-3.304531135	2.11E-36	6.72E-35
TGFA	10.33891817	32.95195043	1.672278627	4.01E-25	2.21E-24
TNFRSF11B	30.39567186	13.77588677	-1.141720719	4.21E-21	1.69E-20
TNFSF13B	1.005474456	5.701772058	2.503533935	1.57E-31	1.71E-30
TNFSF14	0.114871977	1.55979764	3.763260072	4.76E-34	8.30E-33
TNFSF15	1.742586521	0.749936981	-1.216389015	2.42E-24	1.25E-23
TNFSF8	0.475100429	1.746131819	1.877858061	1.89E-25	1.07E-24

Table S2. The immune-related DEGs were screened out in TCGA

ID	conMean	treatMean	logFC	pValue	FDR
TNFSF9	0.555273832	6.020362831	3.438579124	6.92E-34	1.17E-32
UCN	0.219488887	1.32043169	2.588789867	5.39E-33	7.70E-32
VEGFC	3.189453496	6.994936381	1.133001693	1.55E-17	4.81E-17
VIP	0.100782698	0.450943659	2.161699212	9.95E-14	2.40E-13
ADRB1	0.785908845	0.294305843	-1.417045805	6.18E-34	1.06E-32
ANGPTL1	7.418793986	1.196912248	-2.631867294	3.95E-36	1.15E-34
ANGPTL2	9.848470819	25.9743834	1.399117863	6.80E-18	2.15E-17
ANGPTL3	11.02284145	3.314820816	-1.733493375	2.96E-23	1.40E-22
ANGPTL4	11.07169999	340.8253171	4.944083844	1.58E-36	5.32E-35
APLNR	7.656364378	20.39366017	1.413389331	1.84E-12	4.12E-12
AVPR1B	0.132594746	1.831644103	3.788043694	3.26E-26	1.98E-25
AVPR2	7.926016222	0.725834401	-3.44888358	5.62E-39	4.66E-37
BMPR1B	6.925853413	1.525887457	-2.182343294	1.89E-36	6.15E-35
C3AR1	3.346978871	12.57674676	1.909827438	5.02E-28	3.58E-27
CALCRL	10.04338083	20.5130162	1.030294643	7.70E-12	1.66E-11
CNTFR	1.18102445	0.46929796	-1.331462739	1.14E-20	4.43E-20
CRHR2	0.096275014	0.259108678	1.428323996	1.15E-07	1.91E-07
CSF1R	7.452381472	23.2148752	1.639276095	1.55E-24	8.13E-24
CSF2RA	0.940421503	3.654180793	1.958168582	1.91E-30	1.81E-29
CSF2RB	1.354506286	3.549222509	1.389735934	3.25E-23	1.53E-22
CSF3R	0.361273153	2.497306914	2.789211182	4.99E-36	1.42E-34
EGFR	10.23830246	28.39874681	1.471850735	8.44E-24	4.15E-23
ESRRB	12.01685323	0.186421239	-6.010349107	3.15E-41	9.02E-39
ESRRG	9.957923889	1.152864747	-3.110621721	5.61E-41	1.41E-38
FGFR2	11.88838857	4.92208734	-1.272211013	9.34E-28	6.45E-27
FLT1	11.79856785	45.57086422	1.94949998	7.10E-25	3.83E-24
FLT4	4.349304556	9.914092772	1.188696023	3.22E-16	9.14E-16
GCGR	5.430422043	0.7829299	-2.79410928	2.20E-36	6.96E-35
IFNGR2	18.42842153	39.67874578	1.10643392	8.05E-35	1.67E-33
IL10RA	1.7733518	8.547340509	2.268996829	1.42E-31	1.56E-30
IL10RB	9.550411444	19.31456255	1.01605421	2.80E-34	5.10E-33
IL12RB1	0.281273986	1.74229676	2.63094234	1.39E-36	4.81E-35
IL12RB2	0.18194062	1.165041818	2.678842156	0.004842922	0.005916572
IL13RA2	2.898230885	1.347450712	-1.104940028	2.07E-08	3.61E-08
IL15RA	2.055387417	5.678963155	1.466217201	4.48E-30	4.03E-29
IL17RE	2.600160454	0.951753036	-1.449941482	3.77E-29	3.04E-28
IL18R1	1.058179985	2.621489478	1.308801721	1.73E-20	6.62E-20
IL18RAP	0.202747075	0.690481198	1.767921031	3.10E-24	1.58E-23
IL1RAP	0.788852025	1.612824186	1.031762573	1.46E-15	3.99E-15
IL1RL1	8.227516492	2.075214184	-1.987196768	1.62E-22	7.19E-22
IL20RB	0.672176993	10.24814299	3.930377536	6.27E-37	2.47E-35
IL21R	0.180143879	1.136034379	2.656784954	2.61E-32	3.28E-31
IL22RA1	0.865452788	2.586410077	1.579424009	1.95E-15	5.26E-15
IL2RA	0.334480075	1.620337851	2.276302479	9.76E-19	3.28E-18
IL2RB	0.763775293	6.259662302	3.034864672	4.52E-37	1.88E-35
IL2RG	3.815911381	13.90997645	1.866020405	2.53E-23	1.20E-22

Table S2. The immune-related DEGs were screened out in TCGA

ID	conMean	treatMean	logFC	pValue	FDR
IL3RA	4.988365139	13.59181659	1.446099313	3.31E-28	2.40E-27
IL4R	4.881285833	13.64068417	1.482582868	6.75E-35	1.44E-33
INSR	15.25112535	38.0406436	1.318625955	2.49E-25	1.39E-24
KDR	18.6239401	43.08507936	1.210030018	1.98E-12	4.42E-12
LGR6	0.632048805	0.210063396	-1.589211175	7.28E-19	2.47E-18
MC1R	0.264908213	0.799431296	1.59348148	5.41E-20	1.99E-19
MCHR1	0.087334375	4.565749915	5.708158423	1.85E-25	1.05E-24
MET	20.76852771	46.29848048	1.156565898	1.96E-24	1.02E-23
NGFR	2.313789089	8.467775684	1.871725689	9.76E-19	3.28E-18
NR0B2	12.87589489	1.486644849	-3.114540763	9.70E-38	5.16E-36
NR1H3	4.204288542	8.723172943	1.052991307	5.41E-30	4.81E-29
NR1I3	1.192470162	0.217708335	-2.453484618	2.63E-32	3.30E-31
NR2E1	0.035360978	0.719879866	4.347526103	1.26E-05	1.83E-05
NR3C2	11.14803619	2.762160559	-2.012920503	1.39E-37	6.95E-36
NR4A2	12.34924099	5.541268422	-1.156134215	3.53E-08	6.07E-08
NR4A3	7.697664926	3.264947522	-1.23736107	7.83E-09	1.40E-08
NR5A2	0.501486453	1.219608861	1.282135903	8.90E-20	3.23E-19
NRP1	19.33172615	41.3433067	1.096683319	1.34E-21	5.57E-21
NRP2	1.891662182	6.209033214	1.714714178	5.06E-28	3.60E-27
OGFR	6.041739389	12.50979341	1.050022105	1.29E-33	2.06E-32
OPRL1	0.421510908	0.844054275	1.001765804	5.25E-19	1.80E-18
OSMR	9.402279542	26.62434045	1.501663308	1.12E-25	6.47E-25
PPARA	8.637702278	4.296827626	-1.007375689	5.64E-25	3.07E-24
PRLR	5.089004043	1.480109057	-1.781679857	5.65E-17	1.68E-16
PTGER1	9.081212405	0.265394724	-5.096673324	9.60E-41	2.16E-38
PTGER3	36.22309219	8.233594074	-2.137315479	1.06E-33	1.73E-32
PTGFR	2.835108582	0.926759906	-1.613136453	6.90E-30	6.08E-29
PTH1R	73.50216802	8.079874382	-3.185382036	3.31E-25	1.83E-24
RARA	7.262338653	15.19483995	1.065075367	8.71E-27	5.52E-26
SCTR	4.773160885	1.749431184	-1.448059049	4.01E-14	9.93E-14
SDC3	8.28777425	17.34777311	1.065693868	1.44E-23	6.97E-23
SORT1	37.95197806	13.23821091	-1.51946692	9.04E-39	6.89E-37
SSTR5	1.622891918	0.109789717	-3.88575208	4.63E-32	5.53E-31
TACR1	0.832519025	0.314857087	-1.402786099	2.86E-23	1.35E-22
TEK	22.50718069	8.632241494	-1.382578221	3.85E-32	4.66E-31
TGFBR3	15.92464507	5.356685893	-1.571848612	1.99E-34	3.77E-33
THRB	6.16222875	1.629610372	-1.91892517	6.43E-38	3.64E-36
TNFRSF13C	0.141724726	0.325606696	1.200038895	2.02E-08	3.52E-08
TNFRSF14	5.158948625	23.1763763	2.167506029	1.00E-38	7.50E-37
TNFRSF17	0.223611994	0.678031667	1.600355082	1.98E-07	3.24E-07
TNFRSF18	0.155185162	1.163310576	2.906173788	2.65E-32	3.32E-31
TNFRSF1B	8.299252042	22.28444455	1.424983775	6.71E-29	5.27E-28
TNFRSF25	0.642991257	2.448286157	1.928901165	1.19E-19	4.27E-19
TNFRSF4	0.570296188	5.561031829	3.285569301	6.99E-38	3.89E-36
TNFRSF9	0.093367998	1.623367798	4.119917946	6.71E-31	6.72E-30
TSHR	0.046037775	0.251863624	2.451752769	2.01E-23	9.66E-23

Table S2. The immune-related DEGs were screened out in TCGA

ID	conMean	treatMean	logFC	pValue	FDR
TUBB3	0.089289797	0.249026016	1.47972923	8.29E-09	1.48E-08
ITGAL	1.221017113	7.198869878	2.559687022	1.35E-33	2.13E-32
ITGB2	4.560106314	24.18347272	2.406882064	3.25E-34	5.87E-33
TYROBP	15.28945163	96.42195363	2.656824997	1.62E-36	5.42E-35
LCK	2.048330546	4.955535296	1.274592359	8.55E-16	2.36E-15
FCGR3A	5.324274978	54.78629213	3.363157979	1.15E-37	5.91E-36
FCGR3B	0.343330288	0.977381146	1.509324139	5.29E-07	8.44E-07
NCR1	0.050670735	0.327064827	2.690351947	8.14E-32	9.33E-31
NCR3	0.203123655	0.730691176	1.846903528	4.46E-26	2.67E-25
CD247	0.498860564	3.068987214	2.621054103	1.52E-34	2.95E-33
ZAP70	0.24077723	1.853038361	2.944121876	4.11E-34	7.30E-33
LCP2	1.60631535	7.624742511	2.246933469	1.79E-36	5.86E-35
LAT	0.025849011	0.275403271	3.413364728	3.03E-29	2.48E-28
SH3BP2	4.034804486	13.43978336	1.735939211	1.37E-34	2.68E-33
SHC2	2.000505413	4.997053687	1.320713186	1.94E-17	5.96E-17
SHC3	0.59571092	0.209852883	-1.505234119	1.71E-25	9.76E-25
SHC1	17.57345944	37.06370639	1.076608936	1.14E-31	1.27E-30
HCST	2.506624654	9.894549697	1.98088807	1.59E-27	1.08E-26
CD48	1.921758003	7.55138923	1.974315405	5.84E-27	3.75E-26
CD244	0.212198227	1.08118021	2.3491225	1.61E-32	2.11E-31
PRKCA	8.382534028	3.659701811	-1.19566033	9.88E-32	1.11E-30
SH2D1B	0.235735878	0.56113457	1.251175452	1.94E-17	5.96E-17
SH2D1A	0.376502135	2.149816285	2.513483427	9.19E-27	5.82E-26
FAS	4.339167014	10.13104214	1.223292565	1.32E-26	8.24E-26
GZMB	0.760945961	4.539312802	2.576607999	3.66E-32	4.45E-31
PRF1	1.677410115	10.782119	2.684333371	6.58E-35	1.40E-33
BID	2.75022775	5.892018811	1.099210941	1.10E-35	2.85E-34
CD3D	1.618134958	12.11799203	2.904746818	4.82E-30	4.33E-29
CD3E	1.760655164	11.22003117	2.671892403	8.19E-29	6.36E-28
CD3G	0.354834962	2.149054049	2.598481698	6.83E-28	4.79E-27
PTPRC	2.958021914	10.97800215	1.891910882	1.70E-24	8.89E-24
ITK	0.260597012	1.165764617	2.161384071	3.29E-26	1.99E-25
GRAP2	0.204742758	0.68192207	1.735794455	3.09E-25	1.72E-24
CD28	0.401188881	1.092552612	1.445349226	1.22E-16	3.56E-16
ICOS	0.068278474	0.728396481	3.415221227	1.60E-31	1.75E-30
MAP3K14	2.977203307	7.857882948	1.400182934	1.32E-26	8.24E-26
CTLA4	0.114287353	0.907691279	2.98953593	1.86E-27	1.25E-26
CBLC	4.100831594	0.988518325	-2.052576883	3.19E-34	5.78E-33
PDK1	1.321960119	5.058250405	1.935959804	7.51E-35	1.57E-33
PRKCQ	8.453836333	2.651547517	-1.67277158	4.39E-26	2.63E-25
TRAC	4.743952097	20.71046861	2.12619885	4.54E-23	2.11E-22
TRAJ1	0.021891544	0.437100626	4.319519799	9.74E-13	2.22E-12
TRAJ3	0.056552988	0.553283985	3.290345022	2.17E-09	4.02E-09
TRAJ18	0.023256135	0.364358804	3.96967661	1.30E-11	2.76E-11
TRAJ35	0.43073961	0.187077841	-1.203177312	1.11E-06	1.73E-06
TRAV1-1	0.037999211	0.228868729	2.590478984	7.94E-14	1.93E-13

Table S2. The immune-related DEGs were screened out in TCGA

ID	conMean	treatMean	logFC	pValue	FDR
TRAV1-2	0.166123721	0.76676314	2.206522893	1.26E-16	3.68E-16
TRAV2	0.119579648	0.705460227	2.56059288	1.16E-19	4.18E-19
TRAV3	0.116137831	0.748555373	2.688271044	5.58E-19	1.91E-18
TRAV4	0.155301446	1.013204457	2.705782163	1.48E-21	6.13E-21
TRAV5	0.092755032	0.600441044	2.694525143	1.14E-18	3.82E-18
TRAV8-1	0.078945269	0.38536212	2.287290054	1.50E-13	3.58E-13
TRAV8-2	1.889770391	0.677133581	-1.480698583	2.97E-06	4.52E-06
TRAV8-3	0.223816011	0.979909809	2.130335726	1.91E-16	5.49E-16
TRAV8-4	0.152096451	0.656695569	2.110238227	1.80E-16	5.20E-16
TRAV8-6	0.15330211	0.827524867	2.43242511	1.64E-19	5.83E-19
TRAV9-2	0.147797347	0.720451599	2.285281142	4.12E-18	1.33E-17
TRAV10	0.048122876	0.335127569	2.799915593	8.40E-15	2.17E-14
TRAV12-1	0.194002807	1.310076449	2.755501573	7.45E-20	2.72E-19
TRAV12-2	0.218301273	1.35452236	2.633391757	5.45E-19	1.86E-18
TRAV12-3	0.169048066	1.006449563	2.573769456	3.13E-19	1.09E-18
TRAV13-1	0.276330233	1.49609232	2.436733886	3.73E-19	1.29E-18
TRAV13-2	0.121282795	0.650105494	2.422298941	4.92E-19	1.69E-18
TRAV14DV4	0.124019138	0.70335574	2.503691783	4.07E-19	1.41E-18
TRAV16	0.12231083	0.661238388	2.43461833	4.26E-19	1.47E-18
TRAV17	0.140349866	0.945829806	2.752552917	6.60E-21	2.60E-20
TRAV19	0.158428723	1.430488225	3.174601803	4.24E-25	2.33E-24
TRAV20	0.092756593	0.475867866	2.359039294	9.24E-14	2.24E-13
TRAV21	0.168999613	1.258429278	2.896532294	1.19E-21	4.95E-21
TRAV22	0.118260256	0.360959194	1.60987044	1.05E-07	1.74E-07
TRAV23DV6	0.112269687	0.428993963	1.933988895	3.94E-15	1.04E-14
TRAV24	0.068604758	0.681325397	3.311963443	3.45E-16	9.77E-16
TRAV25	0.071733493	0.478806804	2.738724876	1.34E-16	3.89E-16
TRAV26-1	0.078861069	0.468924188	2.571969524	7.88E-21	3.08E-20
TRAV26-2	0.057277341	0.388472446	2.761775838	3.69E-15	9.78E-15
TRAV27	0.080017135	0.826501118	3.368635894	1.54E-17	4.78E-17
TRAV29DV5	0.162362257	1.008920985	2.635524988	2.90E-19	1.01E-18
TRAV35	0.067022355	0.318884614	2.250320207	3.01E-15	8.03E-15
TRAV36DV7	0.061838876	0.289964705	2.22929131	2.69E-16	7.67E-16
TRAV38-1	0.050240006	0.288100635	2.5196643	1.08E-12	2.44E-12
TRAV41	0.09234061	0.563372793	2.609052729	2.93E-21	1.19E-20
TRBC1	0.107523008	0.793897945	2.884308154	5.32E-31	5.40E-30
TRBC2	2.576459104	18.20364254	2.820765564	5.03E-29	4.00E-28
TRBJ2-1	0.223418438	1.614231899	2.853027696	6.50E-22	2.77E-21
TRBJ2-2	0.152181082	0.851993732	2.485053793	2.71E-14	6.78E-14
TRBJ2-3	0.189229992	1.843410977	3.284165078	8.53E-25	4.57E-24
TRBJ2-4	0.030405794	0.38169232	3.649991968	8.92E-13	2.03E-12
TRBJ2-7	0.354102063	2.373454182	2.744751048	1.10E-19	3.97E-19
TRBV2	0.182627847	1.268133092	2.7957275	8.58E-22	3.62E-21
TRBV3-1	0.200870173	1.049109697	2.384830274	2.68E-19	9.37E-19
TRBV4-1	0.123017573	1.354294019	3.460604661	1.01E-21	4.23E-21
TRBV4-2	0.117070998	1.417207799	3.59759569	1.48E-21	6.13E-21

Table S2. The immune-related DEGs were screened out in TCGA

ID	conMean	treatMean	logFC	pValue	FDR
TRBV5-1	0.240400225	1.300121923	2.43513677	2.86E-21	1.16E-20
TRBV5-4	0.135794445	0.662669958	2.286866049	6.29E-18	2.00E-17
TRBV5-5	0.044206428	0.312789636	2.822864647	8.54E-16	2.36E-15
TRBV5-6	0.114617708	0.736621355	2.684093268	1.11E-19	4.00E-19
TRBV6-1	0.15718123	0.85341478	2.440818147	6.70E-20	2.46E-19
TRBV6-5	0.180384492	1.651042643	3.194230164	1.19E-23	5.80E-23
TRBV6-6	0.108635302	0.675095226	2.635598024	3.66E-19	1.27E-18
TRBV7-3	0.406110911	0.945351092	1.218976441	7.44E-05	0.000103171
TRBV7-4	0.033955945	0.282215242	3.055059823	8.58E-14	2.08E-13
TRBV7-6	0.097191294	0.779281389	3.003245367	4.79E-17	1.44E-16
TRBV7-9	0.379669498	2.827487652	2.896704718	1.59E-23	7.65E-23
TRBV9	0.190927867	1.639053492	3.10176335	5.33E-23	2.46E-22
TRBV10-2	0.041812886	0.420734824	3.330891707	1.44E-14	3.66E-14
TRBV10-3	0.188714398	0.79042779	2.066429173	1.05E-15	2.90E-15
TRBV11-2	0.139708356	0.863098204	2.627106406	5.48E-19	1.88E-18
TRBV12-3	0.085789612	0.472627531	2.461828806	6.04E-17	1.79E-16
TRBV12-4	0.138878832	0.845183387	2.60543769	1.30E-18	4.34E-18
TRBV13	0.06753949	0.642841843	3.250660639	6.13E-15	1.60E-14
TRBV14	0.083606474	0.464931186	2.475330631	8.50E-17	2.50E-16
TRBV15	0.047038241	0.521969299	3.472058939	3.47E-18	1.12E-17
TRBV18	0.158751505	0.818320916	2.365896461	1.42E-18	4.73E-18
TRBV19	0.319424665	2.130600632	2.73771257	1.23E-22	5.51E-22
TRBV20-1	1.141252884	3.140158488	1.46021887	4.18E-22	1.80E-21
TRBV24-1	0.099810743	0.523539388	2.391031074	6.28E-19	2.14E-18
TRBV27	0.081946518	0.658507945	3.006446299	6.31E-17	1.87E-16
TRBV28	1.116786232	8.664480758	2.955760234	2.11E-30	1.98E-29
TRBV29-1	0.375992816	2.167340172	2.527148604	5.79E-21	2.29E-20
TRBV30	0.110564428	0.62400682	2.496674495	9.14E-16	2.52E-15
TRDC	0.536539903	2.540657403	2.243444473	1.04E-31	1.17E-30
TRDV1	0.137387798	0.746249974	2.441405095	6.49E-20	2.38E-19
TRGV5	0.128248552	0.435064862	1.762287971	4.56E-15	1.20E-14
TRGV4	0.064596539	0.307302827	2.25013227	1.03E-20	3.98E-20
TRGV3	0.136668263	0.466451619	1.771049186	5.45E-18	1.74E-17
TRGV2	0.052945686	0.356171188	2.749985772	4.09E-25	2.25E-24
TRGJP2	0.115550072	0.363523907	1.653532084	4.30E-06	6.47E-06
TRGC2	0.210222527	1.067557081	2.34432403	1.49E-25	8.51E-25
TRAV6	0.068594009	0.376544184	2.456664669	9.46E-16	2.61E-15

Table S3. Gene ontology analysis of prognostic-associated immune genes.

ONTOLOGY	ID	Description	GeneRatio	BgRatio	pvalue	p.adjust	qvalue	geneID	Count
BP	GO:0043410	positive regulation of MAPK cascade	11/35	499/17913	1.60E-09	1.82E-06	1.06E-06	AGER/CX3CL1/IL11/KL/TGFA/FLT1/INSR/KDR/NRP1/TEK/SHC1	11
BP	GO:0045785	positive regulation of cell adhesion	10/35	388/17913	2.29E-09	1.82E-06	1.06E-06	XCL1/AGER/VAV3/CX3CL1/EBI3/MDK/KDR/NRP1/TEK/TNFRSF13C	10
BP	GO:0050900	leukocyte migration	10/35	467/17913	1.35E-08	5.23E-06	3.05E-06	XCL1/AGER/VAV3/IGKV1-12/CX3CL1/MDK/PDGFD/FLT1/TEK/SHC1	10
BP	GO:0018108	peptidyl-tyrosine phosphorylation	9/35	346/17913	1.53E-08	5.23E-06	3.05E-06	IL11/PDGFD/TGFA/FLT1/INSR/KDR/NRP1/TEK/SHC1	9
BP	GO:0018212	peptidyl-tyrosine modification	9/35	349/17913	1.65E-08	5.23E-06	3.05E-06	IL11/PDGFD/TGFA/FLT1/INSR/KDR/NRP1/TEK/SHC1	9
BP	GO:0060326	cell chemotaxis	8/35	253/17913	2.40E-08	6.34E-06	3.70E-06	XCL1/VAV3/CX3CL1/MDK/PDGFD/FLT1/KDR/NRP1	8
BP	GO:0097529	myeloid leukocyte migration	7/35	170/17913	3.29E-08	7.46E-06	4.35E-06	XCL1/AGER/VAV3/CX3CL1/MDK/PDGFD/FLT1	7
BP	GO:0070661	leukocyte proliferation	8/35	270/17913	3.97E-08	7.88E-06	4.59E-06	XCL1/AGER/VAV3/CD19/CX3CL1/EBI3/IL34/TNFRSF13C	8
BP	GO:0050921	positive regulation of chemotaxis	6/35	130/17913	1.78E-07	3.13E-05	1.82E-05	XCL1/AGER/MDK/PDGFD/KDR/NRP1	6
BP	GO:1904018	positive regulation of vasculature development	7/35	232/17913	2.76E-07	4.37E-05	2.55E-05	CX3CL1/MDK/PDGFD/FLT1/KDR/NRP1/TEK	7
BP	GO:0043552	positive regulation of phosphatidylinositol 3-kinase activity	4/35	32/17913	4.22E-07	6.09E-05	3.55E-05	VAV3/CD19/FLT1/TEK	4
BP	GO:0090218	positive regulation of lipid kinase activity	4/35	36/17913	6.88E-07	8.90E-05	5.18E-05	VAV3/CD19/FLT1/TEK	4
BP	GO:0043491	protein kinase B signaling	7/35	268/17913	7.29E-07	8.90E-05	5.18E-05	CD19/CX3CL1/KL/TGFA/INSR/KDR/TEK	7
BP	GO:0051897	positive regulation of protein kinase B signaling	6/35	173/17913	9.57E-07	0.000108442	6.32E-05	CD19/CX3CL1/KL/TGFA/INSR/TEK	6
BP	GO:0050920	regulation of chemotaxis	6/35	186/17913	1.46E-06	0.000144819	8.44E-05	XCL1/AGER/MDK/PDGFD/KDR/NRP1	6

Table S3. Gene ontology analysis of prognostic-associated immune genes.

ONTOLOGY	ID	Description	GeneRatio	BgRatio	pvalue	p.adjust	qvalue	geneID	Count
BP	GO:0070374	positive regulation of ERK1 and ERK2 cascade	6/35	186/17913	1.46E-06	0.000144819	8.44E-05	AGER/CX3CL1/KDR/NRP1/TEK/SHC1	6
BP	GO:0032103	positive regulation of response to external stimulus	7/35	315/17913	2.14E-06	0.000186577	0.000108724	XCL1/AGER/CX3CL1/MDK/PDGFD/KDR/NRP1	7
BP	GO:0010811	positive regulation of cell-substrate adhesion	5/35	110/17913	2.23E-06	0.000186577	0.000108724	CX3CL1/MDK/KDR/NRP1/TEK	5
BP	GO:1903727	positive regulation of phospholipid metabolic process	4/35	48/17913	2.24E-06	0.000186577	0.000108724	VAV3/CD19/FLT1/TEK	4
BP	GO:0045766	positive regulation of angiogenesis	6/35	203/17913	2.43E-06	0.000192536	0.000112196	CX3CL1/MDK/FLT1/KDR/NRP1/TEK	6
BP	GO:0001954	positive regulation of cell-matrix adhesion	4/35	51/17913	2.86E-06	0.000215901	0.000125812	CX3CL1/KDR/NRP1/TEK	4
BP	GO:0002693	positive regulation of cellular extravasation	3/35	15/17913	3.06E-06	0.000220564	0.000128529	AGER/MDK/PDGFD	3
BP	GO:0043551	regulation of phosphatidylinositol 3-kinase activity	4/35	53/17913	3.34E-06	0.000230349	0.000134231	VAV3/CD19/FLT1/TEK	4
BP	GO:0002694	regulation of leukocyte activation	8/35	492/17913	3.75E-06	0.000247656	0.000144316	XCL1/AGER/VAV3/CD19/CX3CL1/EBI3/MDK/TNFRSF13C	8
BP	GO:0050671	positive regulation of lymphocyte proliferation	5/35	125/17913	4.19E-06	0.000265855	0.000154922	XCL1/AGER/VAV3/EBI3/TNFRSF13C	5
BP	GO:0032946	positive regulation of mononuclear cell proliferation	5/35	126/17913	4.36E-06	0.000265855	0.000154922	XCL1/AGER/VAV3/EBI3/TNFRSF13C	5
BP	GO:0045123	cellular extravasation	4/35	59/17913	5.15E-06	0.000302458	0.000176251	AGER/CX3CL1/MDK/PDGFD	4
BP	GO:0070665	positive regulation of leukocyte proliferation	5/35	134/17913	5.89E-06	0.000332265	0.000193621	XCL1/AGER/VAV3/EBI3/TNFRSF13C	5
BP	GO:0050678	regulation of epithelial cell proliferation	7/35	369/17913	6.08E-06	0.000332265	0.000193621	MDK/TGFA/FLT1/KDR/NRP1/TEK/TGFBR3	7
BP	GO:0043550	regulation of lipid kinase activity	4/35	62/17913	6.28E-06	0.000332265	0.000193621	VAV3/CD19/FLT1/TEK	4
BP	GO:0051896	regulation of protein kinase B signaling	6/35	241/17913	6.52E-06	0.00033376	0.000194492	CD19/CX3CL1/KL/TGFA/INSR/TEK	6
BP	GO:0046651	lymphocyte proliferation	6/35	244/17913	7.00E-06	0.000347058	0.000202241	XCL1/AGER/VAV3/CD19/EBI3/TNFRSF13C	6
BP	GO:0032943	mononuclear cell proliferation	6/35	246/17913	7.34E-06	0.000352629	0.000205488	XCL1/AGER/VAV3/CD19/EBI3/TNFRSF13C	6
BP	GO:0022409	positive regulation of cell-cell adhesion	6/35	251/17913	8.23E-06	0.000383964	0.000223748	XCL1/AGER/CX3CL1/EBI3/MDK/TNFRSF13C	6

Table S3. Gene ontology analysis of prognostic-associated immune genes.

ONTOLOGY	ID	Description	GeneRatio	BgRatio	pvalue	p.adjust	qvalue	geneID	Count
BP	GO:0050918	positive chemotaxis	4/35	67/17913	8.58E-06	0.000388614	0.000226457	AGER/CX3CL1/KDR/NRP1	4
BP	GO:0071674	mononuclear cell migration	4/35	68/17913	9.10E-06	0.000400877	0.000233603	AGER/MDK/PDGFD/FLT1	4
BP	GO:0051251	positive regulation of lymphocyte activation	6/35	258/17913	9.63E-06	0.000412777	0.000240538	XCL1/AGER/VAV3/EBI3/MDK/TNFRSF13C	6
BP	GO:0051249	regulation of lymphocyte activation	7/35	401/17913	1.05E-05	0.00043664	0.000254443	XCL1/AGER/VAV3/CD19/EBI3/MDK/TNFRSF13C	7
BP	GO:0070372	regulation of ERK1 and ERK2 cascade	6/35	271/17913	1.27E-05	0.000517963	0.000301833	AGER/CX3CL1/KDR/NRP1/TEK/SHC1	6
BP	GO:0050673	epithelial cell proliferation	7/35	425/17913	1.52E-05	0.000576242	0.000335793	MDK/TGFA/FLT1/KDR/NRP1/TEK/TGFBR3	7
BP	GO:1901342	regulation of vasculature development	7/35	425/17913	1.52E-05	0.000576242	0.000335793	CX3CL1/MDK/PDGFD/FLT1/KDR/NRP1/TEK	7
BP	GO:0051894	positive regulation of focal adhesion assembly	3/35	25/17913	1.53E-05	0.000576242	0.000335793	KDR/NRP1/TEK	3
BP	GO:0002691	regulation of cellular extravasation	3/35	26/17913	1.72E-05	0.000607163	0.000353812	AGER/MDK/PDGFD	3
BP	GO:0061437	renal system vasculature development	3/35	26/17913	1.72E-05	0.000607163	0.000353812	PDGFD/NRP1/TEK	3
BP	GO:0061440	kidney vasculature development	3/35	26/17913	1.72E-05	0.000607163	0.000353812	PDGFD/NRP1/TEK	3
BP	GO:0070371	ERK1 and ERK2 cascade	6/35	289/17913	1.83E-05	0.000631397	0.000367934	AGER/CX3CL1/KDR/NRP1/TEK/SHC1	6
BP	GO:0001936	regulation of endothelial cell proliferation	5/35	170/17913	1.87E-05	0.000631397	0.000367934	MDK/FLT1/KDR/NRP1/TEK	5
BP	GO:0002460	adaptive immune response based on somatic recombination of immune receptors built from immunoglobulin superfamily domains	6/35	295/17913	2.06E-05	0.000680248	0.000396401	XCL1/AGER/CD19/IGKV1-12/EBI3/TNFRSF13C	6
BP	GO:0031532	actin cytoskeleton reorganization	4/35	85/17913	2.21E-05	0.000715229	0.000416786	MDK/NRP1/TEK/SHC1	4
BP	GO:1903725	regulation of phospholipid metabolic process	4/35	86/17913	2.31E-05	0.000734099	0.000427782	VAV3/CD19/FLT1/TEK	4
BP	GO:0002696	positive regulation of leukocyte activation	6/35	305/17913	2.48E-05	0.000772455	0.000450133	XCL1/AGER/VAV3/EBI3/MDK/TNFRSF13C	6
BP	GO:0042102	positive regulation of T cell proliferation	4/35	90/17913	2.77E-05	0.000840882	0.000490007	XCL1/AGER/EBI3/TNFRSF13C	4
BP	GO:0001935	endothelial cell proliferation	5/35	185/17913	2.81E-05	0.000840882	0.000490007	MDK/FLT1/KDR/NRP1/TEK	5
BP	GO:1903393	positive regulation of adherens junction organization	3/35	31/17913	2.96E-05	0.000868904	0.000506337	KDR/NRP1/TEK	3
BP	GO:0030595	leukocyte chemotaxis	5/35	188/17913	3.04E-05	0.00087526	0.000510041	XCL1/VAV3/CX3CL1/MDK/FLT1	5

Table S3. Gene ontology analysis of prognostic-associated immune genes.

ONTOLOGY	ID	Description	GeneRatio	BgRatio	pvalue	p.adjust	qvalue	geneID	Count
BP	GO:0048010	vascular endothelial growth factor receptor signaling pathway	4/35	93/17913	3.15E-05	0.000889138	0.000518128	VAV3/FLT1/KDR/NRP1	4
BP	GO:0050867	positive regulation of cell activation	6/35	319/17913	3.20E-05	0.000889138	0.000518128	XCL1/AGER/VAV3/EBI3/ MDK/TNFRSF13C	6
BP	GO:0050727	regulation of inflammatory response	7/35	479/17913	3.29E-05	0.000898763	0.000523737	XCL1/AGER/CD19/IGKV1 -12/CX3CL1/MDK/TEK	7
BP	GO:1901890	positive regulation of cell junction assembly	3/35	33/17913	3.58E-05	0.000962709	0.000561	KDR/NRP1/TEK	3
BP	GO:0050870	positive regulation of T cell activation	5/35	198/17913	3.89E-05	0.001002884	0.000584411	XCL1/AGER/EBI3/MDK/T NFRSF13C	5
BP	GO:2000249	regulation of actin cytoskeleton reorganization	3/35	34/17913	3.92E-05	0.001002884	0.000584411	MDK/NRP1/TEK	3
BP	GO:0050670	regulation of lymphocyte proliferation	5/35	199/17913	3.98E-05	0.001002884	0.000584411	XCL1/AGER/VAV3/EBI3/ TNFRSF13C	5
BP	GO:0050679	positive regulation of epithelial cell proliferation	5/35	199/17913	3.98E-05	0.001002884	0.000584411	MDK/TGFA/KDR/NRP1/T EK	5
BP	GO:0032944	regulation of mononuclear cell proliferation	5/35	200/17913	4.08E-05	0.001011112	0.000589206	XCL1/AGER/VAV3/EBI3/ TNFRSF13C	5
BP	GO:0010810	regulation of cell-substrate adhesion	5/35	201/17913	4.18E-05	0.001019526	0.000594109	CX3CL1/MDK/KDR/NRP1 /TEK	5
BP	GO:0001938	positive regulation of endothelial cell proliferation	4/35	107/17913	5.46E-05	0.001312788	0.000765001	MDK/KDR/NRP1/TEK	4
BP	GO:0070663	regulation of leukocyte proliferation	5/35	214/17913	5.63E-05	0.001313186	0.000765234	XCL1/AGER/VAV3/EBI3/ TNFRSF13C	5
BP	GO:1903039	positive regulation of leukocyte cell-cell adhesion	5/35	214/17913	5.63E-05	0.001313186	0.000765234	XCL1/AGER/EBI3/MDK/T NFRSF13C	5
BP	GO:1904019	epithelial cell apoptotic process	4/35	111/17913	6.30E-05	0.001435161	0.000836312	MDK/KDR/TEK/BID	4
BP	GO:0038084	vascular endothelial growth factor signaling pathway	3/35	40/17913	6.42E-05	0.001435161	0.000836312	FLT1/KDR/NRP1	3
BP	GO:0051281	positive regulation of release of sequestered calcium ion into cytosol	3/35	40/17913	6.42E-05	0.001435161	0.000836312	XCL1/CD19/CX3CL1	3
BP	GO:0001952	regulation of cell-matrix adhesion	4/35	117/17913	7.74E-05	0.001705023	0.000993569	CX3CL1/KDR/NRP1/TEK	4
BP	GO:0002687	positive regulation of leukocyte migration	4/35	121/17913	8.82E-05	0.001890865	0.001101865	XCL1/AGER/MDK/PDGF D	4
BP	GO:0045765	regulation of angiogenesis	6/35	383/17913	8.82E-05	0.001890865	0.001101865	CX3CL1/MDK/FLT1/KDR /NRP1/TEK	6
BP	GO:0071675	regulation of mononuclear cell migration	3/35	45/17913	9.17E-05	0.001938281	0.001129495	AGER/MDK/PDGFD	3
BP	GO:0022407	regulation of cell-cell adhesion	6/35	389/17913	9.61E-05	0.001991093	0.00116027	XCL1/AGER/CX3CL1/EBI 3/MDK/TNFRSF13C	6
BP	GO:0045687	positive regulation of glial cell differentiation	3/35	46/17913	9.79E-05	0.001991093	0.00116027	AGER/IL34/MDK	3

Table S3. Gene ontology analysis of prognostic-associated immune genes.

ONTOLOGY	ID	Description	GeneRatio	BgRatio	pvalue	p.adjust	qvalue	geneID	Count
BP	GO:1904036	negative regulation of epithelial cell apoptotic process	3/35	46/17913	9.79E-05	0.001991093	0.00116027	MDK/KDR/TEK	3
BP	GO:0014911	positive regulation of smooth muscle cell migration	3/35	47/17913	0.000104459	0.002097118	0.001222055	MDK/PDGFD/NRP1	3
BP	GO:0050730	regulation of peptidyl-tyrosine phosphorylation	5/35	245/17913	0.000106675	0.002114831	0.001232376	IL11/PDGFD/TGFA/NRP1/SHC1	5
BP	GO:0043406	positive regulation of MAP kinase activity	5/35	250/17913	0.000117288	0.002296526	0.001338255	AGER/TGFA/FLT1/INSR/SHC1	5
BP	GO:0010524	positive regulation of calcium ion transport into cytosol	3/35	54/17913	0.000158301	0.003061774	0.001784189	XCL1/CD19/CX3CL1	3
BP	GO:0071670	smooth muscle cell chemotaxis	2/35	10/17913	0.000165264	0.003157939	0.001840227	MDK/PDGFD	2
BP	GO:0045834	positive regulation of lipid metabolic process	4/35	144/17913	0.000172781	0.003262273	0.001901026	VAV3/CD19/FLT1/TEK	4
BP	GO:0042129	regulation of T cell proliferation	4/35	149/17913	0.000196972	0.003675272	0.002141693	XCL1/AGER/EBI3/TNFRSF13C	4
BP	GO:0001819	positive regulation of cytokine production	6/35	446/17913	0.000202558	0.003735551	0.00217682	XCL1/AGER/CX3CL1/EBI3/MDK/TNFRSF13C	6
BP	GO:0042063	gliogenesis	5/35	285/17913	0.000216107	0.003904605	0.002275332	NDRG1/AGER/CX3CL1/IL34/MDK	5
BP	GO:0030593	neutrophil chemotaxis	3/35	60/17913	0.000216649	0.003904605	0.002275332	XCL1/VAV3/MDK	3
BP	GO:0051893	regulation of focal adhesion assembly	3/35	61/17913	0.00022755	0.004009944	0.002336716	KDR/NRP1/TEK	3
BP	GO:0090109	regulation of cell-substrate junction assembly	3/35	61/17913	0.00022755	0.004009944	0.002336716	KDR/NRP1/TEK	3
BP	GO:0001906	cell killing	4/35	156/17913	0.000234829	0.004092727	0.002384957	ULBP2/HAMP/XCL1/AGER	4
BP	GO:0045078	positive regulation of interferon-gamma biosynthetic process	2/35	12/17913	0.000241793	0.004168299	0.002428995	EBI3/TNFRSF13C	2
BP	GO:0002449	lymphocyte mediated immunity	5/35	293/17913	0.000245664	0.0041895	0.002441349	ULBP2/XCL1/AGER/CD19/IGKV1-12	5
BP	GO:0042108	positive regulation of cytokine biosynthetic process	3/35	63/17913	0.000250406	0.004224938	0.002462	AGER/EBI3/TNFRSF13C	3
BP	GO:1903037	regulation of leukocyte cell-cell adhesion	5/35	295/17913	0.000253513	0.004232332	0.002466309	XCL1/AGER/EBI3/MDK/TNFRSF13C	5
BP	GO:0035924	cellular response to vascular endothelial growth factor stimulus	3/35	65/17913	0.000274698	0.004538238	0.00264457	FLT1/KDR/NRP1	3
BP	GO:0060347	heart trabecula formation	2/35	13/17913	0.000285405	0.004618895	0.002691571	TEK/TGFBR3	2
BP	GO:0090594	inflammatory response to wounding	2/35	13/17913	0.000285405	0.004618895	0.002691571	AGER/MDK	2
BP	GO:1903391	regulation of adherens junction organization	3/35	67/17913	0.000300464	0.004813491	0.002804968	KDR/NRP1/TEK	3
BP	GO:0050863	regulation of T cell activation	5/35	307/17913	0.000304699	0.004832528	0.002816061	XCL1/AGER/EBI3/MDK/TNFRSF13C	5
BP	GO:0050728	negative regulation of inflammatory response	4/35	170/17913	0.000325864	0.005117033	0.002981851	AGER/CX3CL1/MDK/TEK	4

Table S3. Gene ontology analysis of prognostic-associated immune genes.

ONTOLOGY	ID	Description	GeneRatio	BgRatio	pvalue	p.adjust	qvalue	geneID	Count
BP	GO:0002523	leukocyte migration involved in inflammatory response	2/35	14/17913	0.000332564	0.005171037	0.003013321	CX3CL1/MDK	2
BP	GO:1901652	response to peptide	6/35	491/17913	0.000339637	0.005214878	0.003038868	AGER/KL/INSR/TEK/TGFBR3/SHC1	6
BP	GO:1904427	positive regulation of calcium ion transmembrane transport	3/35	70/17913	0.000341959	0.005214878	0.003038868	XCL1/CD19/CX3CL1	3
BP	GO:0042100	B cell proliferation	3/35	71/17913	0.000356569	0.005385885	0.003138519	VAV3/CD19/TNFRSF13C	3
BP	GO:1990266	neutrophil migration	3/35	72/17913	0.000371575	0.005475902	0.003190975	XCL1/VAV3/MDK	3
BP	GO:0031589	cell-substrate adhesion	5/35	322/17913	0.000379323	0.005475902	0.003190975	CX3CL1/MDK/KDR/NRP1/TEK	5
BP	GO:0043405	regulation of MAP kinase activity	5/35	322/17913	0.000379323	0.005475902	0.003190975	AGER/TGFA/FLT1/INSR/SHC1	5
BP	GO:0071902	positive regulation of protein serine/threonine kinase activity	5/35	322/17913	0.000379323	0.005475902	0.003190975	AGER/TGFA/FLT1/INSR/SHC1	5
BP	GO:0042098	T cell proliferation	4/35	177/17913	0.000379791	0.005475902	0.003190975	XCL1/AGER/EBI3/TNFRSF13C	4
BP	GO:0048638	regulation of developmental growth	5/35	325/17913	0.000395775	0.005654952	0.003295313	HAMP/SEMA3G/INSR/NRP1/TGFBR3	5
BP	GO:0007159	leukocyte cell-cell adhesion	5/35	328/17913	0.000412764	0.005845027	0.003406075	XCL1/AGER/EBI3/MDK/TNFRSF13C	5
BP	GO:0048639	positive regulation of developmental growth	4/35	183/17913	0.000430867	0.005981274	0.00348547	HAMP/INSR/NRP1/TGFBR3	4
BP	GO:0014015	positive regulation of gliogenesis	3/35	76/17913	0.000435654	0.005981274	0.00348547	AGER/IL34/MDK	3
BP	GO:0045072	regulation of interferon-gamma biosynthetic process	2/35	16/17913	0.00043747	0.005981274	0.00348547	EBI3/TNFRSF13C	2
BP	GO:2000251	positive regulation of actin cytoskeleton reorganization	2/35	16/17913	0.00043747	0.005981274	0.00348547	NRP1/TEK	2
BP	GO:0045685	regulation of glial cell differentiation	3/35	77/17913	0.00045271	0.006136742	0.003576066	AGER/IL34/MDK	3
BP	GO:0002685	regulation of leukocyte migration	4/35	186/17913	0.000458165	0.006158053	0.003588485	XCL1/AGER/MDK/PDGFD	4
BP	GO:0051279	regulation of release of sequestered calcium ion into cytosol	3/35	78/17913	0.00047019	0.006266568	0.00365172	XCL1/CD19/CX3CL1	3
BP	GO:0042095	interferon-gamma biosynthetic process	2/35	17/17913	0.000495192	0.006544781	0.003813844	EBI3/TNFRSF13C	2
BP	GO:0032102	negative regulation of response to external stimulus	5/35	343/17913	0.000506141	0.006634208	0.003865955	AGER/CX3CL1/MDK/NRP1/TEK	5
BP	GO:0007045	cell-substrate adherens junction assembly	3/35	81/17913	0.000525213	0.006717648	0.003914578	KDR/NRP1/TEK	3
BP	GO:0014068	positive regulation of phosphatidylinositol 3-kinase signaling	3/35	81/17913	0.000525213	0.006717648	0.003914578	FLT1/KDR/TEK	3
BP	GO:0048041	focal adhesion assembly	3/35	81/17913	0.000525213	0.006717648	0.003914578	KDR/NRP1/TEK	3
BP	GO:0038083	peptidyl-tyrosine autophosphorylation	2/35	18/17913	0.000556407	0.00694852	0.004049114	INSR/KDR	2

Table S3. Gene ontology analysis of prognostic-associated immune genes.

ONTOLOGY	ID	Description	GeneRatio	BgRatio	pvalue	p.adjust	qvalue	geneID	Count
BP	GO:0046641	positive regulation of alpha-beta T cell proliferation	2/35	18/17913	0.000556407	0.00694852	0.004049114	XCL1/EBI3	2
BP	GO:0060977	coronary vasculature morphogenesis	2/35	18/17913	0.000556407	0.00694852	0.004049114	NRP1/TGFBR3	2
BP	GO:0051781	positive regulation of cell division	3/35	84/17913	0.000584204	0.007182535	0.004185482	MDK/PDGFD/TGFA	3
BP	GO:0071621	granulocyte chemotaxis	3/35	84/17913	0.000584204	0.007182535	0.004185482	XCL1/VAV3/MDK	3
BP	GO:0034764	positive regulation of transmembrane transport	4/35	199/17913	0.000590889	0.007208847	0.004200815	XCL1/CD19/CX3CL1/INSR	4
BP	GO:0014910	regulation of smooth muscle cell migration	3/35	85/17913	0.000604768	0.007321854	0.004266667	MDK/PDGFD/NRP1	3
BP	GO:0034446	substrate adhesion-dependent cell spreading	3/35	86/17913	0.000625791	0.007518975	0.004381536	MDK/NRP1/TEK	3
BP	GO:1904035	regulation of epithelial cell apoptotic process	3/35	88/17913	0.000669226	0.007980396	0.00465042	MDK/KDR/TEK	3
BP	GO:0060216	definitive hemopoiesis	2/35	20/17913	0.00068927	0.008097648	0.004718746	TEK/TGFBR3	2
BP	GO:0071636	positive regulation of transforming growth factor beta production	2/35	20/17913	0.00068927	0.008097648	0.004718746	XCL1/CX3CL1	2
BP	GO:0034333	adherens junction assembly	3/35	90/17913	0.000714542	0.008212053	0.004785414	KDR/NRP1/TEK	3
BP	GO:0046634	regulation of alpha-beta T cell activation	3/35	90/17913	0.000714542	0.008212053	0.004785414	XCL1/AGER/EBI3	3
BP	GO:1901888	regulation of cell junction assembly	3/35	90/17913	0.000714542	0.008212053	0.004785414	KDR/NRP1/TEK	3
BP	GO:0046777	protein autophosphorylation	4/35	210/17913	0.000722875	0.008248055	0.004806393	FLT1/INSR/KDR/TEK	4
BP	GO:0014909	smooth muscle cell migration	3/35	91/17913	0.000737915	0.008359523	0.004871349	MDK/PDGFD/NRP1	3
BP	GO:0010001	glial cell differentiation	4/35	212/17913	0.000748941	0.00842426	0.004909073	NDRG1/AGER/IL34/MDK	4
BP	GO:0007160	cell-matrix adhesion	4/35	214/17913	0.000775667	0.008663437	0.005048449	CX3CL1/KDR/NRP1/TEK	4
BP	GO:0032649	regulation of interferon-gamma production	3/35	93/17913	0.000786113	0.008693047	0.005065704	XCL1/EBI3/TNFRSF13C	3
BP	GO:0042113	B cell activation	4/35	215/17913	0.00078928	0.008693047	0.005065704	VAV3/CD19/IL11/TNFRSF13C	4
BP	GO:0048714	positive regulation of oligodendrocyte differentiation	2/35	22/17913	0.000835954	0.009080983	0.005291765	IL34/MDK	2
BP	GO:0050927	positive regulation of positive chemotaxis	2/35	22/17913	0.000835954	0.009080983	0.005291765	AGER/KDR	2
BP	GO:0007044	cell-substrate junction assembly	3/35	96/17913	0.000862096	0.009301253	0.005420124	KDR/NRP1/TEK	3
BP	GO:0001909	leukocyte mediated cytotoxicity	3/35	97/17913	0.000888423	0.00952053	0.005547903	ULBP2/XCL1/AGER	3
BP	GO:0030517	negative regulation of axon extension	2/35	23/17913	0.000914447	0.009549968	0.005565058	SEMA3G/NRP1	2
BP	GO:0050926	regulation of positive chemotaxis	2/35	23/17913	0.000914447	0.009549968	0.005565058	AGER/KDR	2
BP	GO:0071677	positive regulation of mononuclear cell migration	2/35	23/17913	0.000914447	0.009549968	0.005565058	AGER/PDGFD	2
BP	GO:0010522	regulation of calcium ion transport into cytosol	3/35	98/17913	0.000915255	0.009549968	0.005565058	XCL1/CD19/CX3CL1	3

Table S3. Gene ontology analysis of prognostic-associated immune genes.

ONTOLOGY	ID	Description	GeneRatio	BgRatio	pvalue	p.adjust	qvalue	geneID	Count
BP	GO:0097530	granulocyte migration	3/35	100/17913	0.000970454	0.010001397	0.005828119	XCL1/VAV3/MDK	3
BP	GO:0001914	regulation of T cell mediated cytotoxicity	2/35	24/17913	0.000996356	0.010001397	0.005828119	XCL1/AGER	2
BP	GO:0030325	adrenal gland development	2/35	24/17913	0.000996356	0.010001397	0.005828119	MDK/INSR	2
BP	GO:0060343	trabecula formation	2/35	24/17913	0.000996356	0.010001397	0.005828119	TEK/TGFBR3	2
BP	GO:0072012	glomerulus vasculature development	2/35	24/17913	0.000996356	0.010001397	0.005828119	PDGFD/TEK	2
BP	GO:0090023	positive regulation of neutrophil chemotaxis	2/35	24/17913	0.000996356	0.010001397	0.005828119	XCL1/MDK	2
BP	GO:0014812	muscle cell migration	3/35	103/17913	0.001057142	0.010489275	0.00611242	MDK/PDGFD/NRP1	3
BP	GO:0043434	response to peptide hormone	5/35	404/17913	0.001058187	0.010489275	0.00611242	KL/INSR/TEK/TGFBR3/SHC1	5
BP	GO:0007176	regulation of epidermal growth factor-activated receptor activity	2/35	25/17913	0.001081669	0.010655445	0.006209252	TGFA/SHC1	2
BP	GO:0032609	interferon-gamma production	3/35	105/17913	0.001117572	0.010941165	0.00637575	XCL1/EBI3/TNFRSF13C	3
BP	GO:0031348	negative regulation of defense response	4/35	237/17913	0.001133469	0.011028724	0.006426773	AGER/CX3CL1/MDK/TEK	4
BP	GO:0051402	neuron apoptotic process	4/35	238/17913	0.001151254	0.011133464	0.006487809	CX3CL1/MDK/NRP1/BID	4
BP	GO:0071624	positive regulation of granulocyte chemotaxis	2/35	26/17913	0.001170372	0.01124976	0.006555578	XCL1/MDK	2
BP	GO:0046640	regulation of alpha-beta T cell proliferation	2/35	27/17913	0.001262454	0.011983785	0.006983316	XCL1/EBI3	2
BP	GO:1902624	positive regulation of neutrophil migration	2/35	27/17913	0.001262454	0.011983785	0.006983316	XCL1/MDK	2
BP	GO:0042035	regulation of cytokine biosynthetic process	3/35	110/17913	0.001278088	0.011983785	0.006983316	AGER/EBI3/TNFRSF13C	3
BP	GO:0050864	regulation of B cell activation	3/35	110/17913	0.001278088	0.011983785	0.006983316	VAV3/CD19/TNFRSF13C	3
BP	GO:0048732	gland development	5/35	422/17913	0.001284517	0.011983785	0.006983316	HAMP/MDK/INSR/NRP1/TGFBR3	5
BP	GO:0021782	glial cell development	3/35	111/17913	0.00131184	0.01216712	0.007090152	NDRG1/AGER/MDK	3
BP	GO:0051209	release of sequestered calcium ion into cytosol	3/35	112/17913	0.001346148	0.012340988	0.00719147	XCL1/CD19/CX3CL1	3
BP	GO:0051283	negative regulation of sequestering of calcium ion	3/35	112/17913	0.001346148	0.012340988	0.00719147	XCL1/CD19/CX3CL1	3
BP	GO:0003007	heart morphogenesis	4/35	249/17913	0.001360041	0.012396693	0.007223931	INSR/NRP1/TEK/TGFBR3	4
BP	GO:0051282	regulation of sequestering of calcium ion	3/35	114/17913	0.001416452	0.0128371	0.007480569	XCL1/CD19/CX3CL1	3
BP	GO:0014066	regulation of phosphatidylinositol 3-kinase signaling	3/35	117/17913	0.001526175	0.013675217	0.007968966	FLT1/KDR/TEK	3
BP	GO:0051208	sequestering of calcium ion	3/35	117/17913	0.001526175	0.013675217	0.007968966	XCL1/CD19/CX3CL1	3
BP	GO:0001569	branching involved in blood vessel morphogenesis	2/35	30/17913	0.001558838	0.013735094	0.008003858	MDK/NRP1	2

Table S3. Gene ontology analysis of prognostic-associated immune genes.

ONTOLOGY	ID	Description	GeneRatio	BgRatio	pvalue	p.adjust	qvalue	geneID	Count
BP	GO:0046633	alpha-beta T cell proliferation	2/35	30/17913	0.001558838	0.013735094	0.008003858	XCL1/EBI3	2
BP	GO:0090022	regulation of neutrophil chemotaxis	2/35	30/17913	0.001558838	0.013735094	0.008003858	XCL1/MDK	2
BP	GO:0042110	T cell activation	5/35	443/17913	0.001591931	0.013949184	0.008128615	XCL1/AGER/EBI3/MDK/TNFRSF13C	5
BP	GO:0051928	positive regulation of calcium ion transport	3/35	119/17913	0.001602209	0.013962103	0.008136143	XCL1/CD19/CX3CL1	3
BP	GO:0042089	cytokine biosynthetic process	3/35	120/17913	0.001641101	0.01419132	0.008269715	AGER/EBI3/TNFRSF13C	3
BP	GO:0035767	endothelial cell chemotaxis	2/35	31/17913	0.001664304	0.01419132	0.008269715	KDR/NRP1	2
BP	GO:0061036	positive regulation of cartilage development	2/35	31/17913	0.001664304	0.01419132	0.008269715	BMP1/MDK	2
BP	GO:1903978	regulation of microglial cell activation	2/35	31/17913	0.001664304	0.01419132	0.008269715	AGER/CX3CL1	2
BP	GO:0042107	cytokine metabolic process	3/35	121/17913	0.001680581	0.014253485	0.00830594	AGER/EBI3/TNFRSF13C	3
BP	GO:0045927	positive regulation of growth	4/35	266/17913	0.001732859	0.014618696	0.008518759	HAMP/INSR/NRP1/TGFBR3	4
BP	GO:0061384	heart trabecula morphogenesis	2/35	32/17913	0.001773084	0.014878894	0.008670385	TEK/TGFBR3	2
BP	GO:0050769	positive regulation of neurogenesis	5/35	455/17913	0.001790316	0.014944425	0.008708572	AGER/CX3CL1/IL34/MDK/NRP1	5
BP	GO:0001913	T cell mediated cytotoxicity	2/35	33/17913	0.001885166	0.015505965	0.009035798	XCL1/AGER	2
BP	GO:2000352	negative regulation of endothelial cell apoptotic process	2/35	33/17913	0.001885166	0.015505965	0.009035798	KDR/TEK	2
BP	GO:0097553	calcium ion transmembrane import into cytosol	3/35	126/17913	0.001886918	0.015505965	0.009035798	XCL1/CD19/CX3CL1	3
BP	GO:0008286	insulin receptor signaling pathway	3/35	128/17913	0.001973681	0.016052608	0.009354344	KL/INSR/SHC1	3
BP	GO:0014013	regulation of gliogenesis	3/35	128/17913	0.001973681	0.016052608	0.009354344	AGER/IL34/MDK	3
BP	GO:0032735	positive regulation of interleukin-12 production	2/35	34/17913	0.002000537	0.016083012	0.009372061	AGER/MDK	2
BP	GO:1902622	regulation of neutrophil migration	2/35	34/17913	0.002000537	0.016083012	0.009372061	XCL1/MDK	2
BP	GO:0003206	cardiac chamber morphogenesis	3/35	129/17913	0.002017982	0.016083012	0.009372061	NRP1/TEK/TGFBR3	3
BP	GO:0010595	positive regulation of endothelial cell migration	3/35	129/17913	0.002017982	0.016083012	0.009372061	KDR/NRP1/TEK	3
BP	GO:0001889	liver development	3/35	130/17913	0.002062899	0.016358792	0.009532767	HAMP/MDK/TGFBR3	3
BP	GO:0048246	macrophage chemotaxis	2/35	35/17913	0.002119185	0.016721529	0.009744144	CX3CL1/MDK	2
BP	GO:0046631	alpha-beta T cell activation	3/35	133/17913	0.002201382	0.017198976	0.010022367	XCL1/AGER/EBI3	3
BP	GO:0061008	hepaticobiliary system development	3/35	133/17913	0.002201382	0.017198976	0.010022367	HAMP/MDK/TGFBR3	3
BP	GO:0071900	regulation of protein serine/threonine kinase activity	5/35	479/17913	0.002241018	0.017229911	0.010040394	AGER/TGFA/FLT1/INSR/SHC1	5
BP	GO:0071634	regulation of transforming growth factor beta production	2/35	36/17913	0.002241097	0.017229911	0.010040394	XCL1/CX3CL1	2

Table S3. Gene ontology analysis of prognostic-associated immune genes.

ONTOLOGY	ID	Description	GeneRatio	BgRatio	pvalue	p.adjust	qvalue	geneID	Count
BP	GO:1900026	positive regulation of substrate adhesion-dependent cell spreading	2/35	36/17913	0.002241097	0.017229911	0.010040394	MDK/NRP1	2
BP	GO:0034332	adherens junction organization	3/35	134/17913	0.002248797	0.017229911	0.010040394	KDR/NRP1/TEK	3
BP	GO:0002822	regulation of adaptive immune response based on somatic recombination of immune receptors built from immunoglobulin superfamily domains	3/35	135/17913	0.002296843	0.017513427	0.010205608	XCL1/AGER/TNFRSF13C	3
BP	GO:0002521	leukocyte differentiation	5/35	485/17913	0.002365608	0.017951453	0.010460859	AGER/CD19/IL11/IL34/MDK	5
BP	GO:0014065	phosphatidylinositol 3-kinase signaling	3/35	138/17913	0.002444802	0.018464074	0.010759579	FLT1/KDR/TEK	3
BP	GO:0071604	transforming growth factor beta production	2/35	38/17913	0.002494664	0.018575291	0.010824388	XCL1/CX3CL1	2
BP	GO:0090050	positive regulation of cell migration involved in sprouting angiogenesis	2/35	38/17913	0.002494664	0.018575291	0.010824388	KDR/NRP1	2
BP	GO:2001222	regulation of neuron migration	2/35	38/17913	0.002494664	0.018575291	0.010824388	CX3CL1/MDK	2
BP	GO:1904064	positive regulation of cation transmembrane transport	3/35	140/17913	0.002546653	0.018873795	0.010998336	XCL1/CD19/CX3CL1	3
BP	GO:0032956	regulation of actin cytoskeleton organization	4/35	297/17913	0.002586517	0.019080072	0.011118539	CX3CL1/MDK/NRP1/TEK	4
BP	GO:0030890	positive regulation of B cell proliferation	2/35	39/17913	0.002626294	0.019194942	0.011185477	VAV3/TNFRSF13C	2
BP	GO:0048713	regulation of oligodendrocyte differentiation	2/35	39/17913	0.002626294	0.019194942	0.011185477	IL34/MDK	2
BP	GO:1902107	positive regulation of leukocyte differentiation	3/35	143/17913	0.002704306	0.019674447	0.0114649	AGER/IL34/MDK	3
BP	GO:1903169	regulation of calcium ion transmembrane transport	3/35	144/17913	0.002758168	0.01997468	0.011639855	XCL1/CD19/CX3CL1	3
BP	GO:0060402	calcium ion transport into cytosol	3/35	145/17913	0.002812691	0.020276944	0.011815993	XCL1/CD19/CX3CL1	3
BP	GO:0043524	negative regulation of neuron apoptotic process	3/35	146/17913	0.002867876	0.02058123	0.01199331	CX3CL1/MDK/NRP1	3
BP	GO:0000187	activation of MAPK activity	3/35	147/17913	0.002923727	0.02088753	0.0121718	TGFA/INSR/SHC1	3
BP	GO:0042088	T-helper 1 type immune response	2/35	42/17913	0.003040422	0.021623808	0.012600852	XCL1/EBI3	2
BP	GO:0002819	regulation of adaptive immune response	3/35	150/17913	0.003095302	0.021915842	0.012771029	XCL1/AGER/TNFRSF13C	3
BP	GO:2001236	regulation of extrinsic apoptotic signaling pathway	3/35	151/17913	0.003153842	0.022231084	0.01295473	CX3CL1/NRP1/BID	3
BP	GO:0001764	neuron migration	3/35	152/17913	0.003213062	0.022448969	0.013081698	CX3CL1/MDK/NRP1	3
BP	GO:0034767	positive regulation of ion transmembrane transport	3/35	152/17913	0.003213062	0.022448969	0.013081698	XCL1/CD19/CX3CL1	3
BP	GO:0050729	positive regulation of inflammatory response	3/35	153/17913	0.003272963	0.022767188	0.013267134	AGER/CX3CL1/MDK	3
BP	GO:0007568	aging	4/35	318/17913	0.003306762	0.022887475	0.013337229	HAMP/AGER/CX3CL1/KL	4
BP	GO:0071622	regulation of granulocyte chemotaxis	2/35	44/17913	0.003332416	0.022887475	0.013337229	XCL1/MDK	2
BP	GO:0008361	regulation of cell size	3/35	154/17913	0.003333548	0.022887475	0.013337229	VAV3/SEMA3G/NRP1	3

Table S3. Gene ontology analysis of prognostic-associated immune genes.

ONTOLOGY	ID	Description	GeneRatio	BgRatio	pvalue	p.adjust	qvalue	geneID	Count
BP	GO:0060401	cytosolic calcium ion transport	3/35	157/17913	0.003519432	0.024059565	0.014020242	XCL1/CD19/CX3CL1	3
BP	GO:0099173	postsynapse organization	3/35	158/17913	0.003582778	0.024387495	0.014211336	NDRG1/INSR/NRP1	3
BP	GO:0048015	phosphatidylinositol-mediated signaling	3/35	160/17913	0.003711563	0.025156146	0.014659253	FLT1/KDR/TEK	3
BP	GO:0001101	response to acid chemical	4/35	330/17913	0.003774546	0.025474171	0.014844576	HAMP/PDGFD/KDR/TGFBR3	4
BP	GO:0061383	trabecula morphogenesis	2/35	47/17913	0.003794026	0.025497138	0.014857959	TEK/TGFBR3	2
BP	GO:0051302	regulation of cell division	3/35	162/17913	0.003843152	0.02571831	0.014986843	MDK/PDGFD/TGFA	3
BP	GO:0002695	negative regulation of leukocyte activation	3/35	163/17913	0.003910005	0.025807713	0.015038941	XCL1/CX3CL1/MDK	3
BP	GO:0048017	inositol lipid-mediated signaling	3/35	163/17913	0.003910005	0.025807713	0.015038941	FLT1/KDR/TEK	3
BP	GO:0032653	regulation of interleukin-10 production	2/35	48/17913	0.003954145	0.025807713	0.015038941	XCL1/AGER	2
BP	GO:0045840	positive regulation of mitotic nuclear division	2/35	48/17913	0.003954145	0.025807713	0.015038941	TGFA/INSR	2
BP	GO:0101023	vascular endothelial cell proliferation	2/35	48/17913	0.003954145	0.025807713	0.015038941	MDK/FLT1	2
BP	GO:1905562	regulation of vascular endothelial cell proliferation	2/35	48/17913	0.003954145	0.025807713	0.015038941	MDK/FLT1	2
BP	GO:0035272	exocrine system development	2/35	49/17913	0.00411737	0.026653666	0.015531903	INSR/NRP1	2
BP	GO:0055023	positive regulation of cardiac muscle tissue growth	2/35	49/17913	0.00411737	0.026653666	0.015531903	HAMP/TGFBR3	2
BP	GO:0032970	regulation of actin filament-based process	4/35	341/17913	0.004241288	0.026853466	0.015648333	CX3CL1/MDK/NRP1/TEK	4
BP	GO:0001774	microglial cell activation	2/35	50/17913	0.004283687	0.026853466	0.015648333	AGER/CX3CL1	2
BP	GO:0002269	leukocyte activation involved in inflammatory response	2/35	50/17913	0.004283687	0.026853466	0.015648333	AGER/CX3CL1	2
BP	GO:0014009	glial cell proliferation	2/35	50/17913	0.004283687	0.026853466	0.015648333	CX3CL1/IL34	2
BP	GO:0050771	negative regulation of axonogenesis	2/35	50/17913	0.004283687	0.026853466	0.015648333	SEMA3G/NRP1	2
BP	GO:0060976	coronary vasculature development	2/35	50/17913	0.004283687	0.026853466	0.015648333	NRP1/TGFBR3	2
BP	GO:1905517	macrophage migration	2/35	50/17913	0.004283687	0.026853466	0.015648333	CX3CL1/MDK	2
BP	GO:2000179	positive regulation of neural precursor cell proliferation	2/35	50/17913	0.004283687	0.026853466	0.015648333	CX3CL1/MDK	2
BP	GO:0038095	Fc-epsilon receptor signaling pathway	3/35	169/17913	0.00432608	0.027012454	0.01574098	VAV3/IGKV1-12/SHC1	3
BP	GO:0003205	cardiac chamber development	3/35	170/17913	0.00439794	0.027353465	0.015939697	NRP1/TEK/TGFBR3	3
BP	GO:0032613	interleukin-10 production	2/35	51/17913	0.004453084	0.027374387	0.015951889	XCL1/AGER	2
BP	GO:0150077	regulation of neuroinflammatory response	2/35	51/17913	0.004453084	0.027374387	0.015951889	AGER/CX3CL1	2
BP	GO:1900024	regulation of substrate adhesion-dependent cell spreading	2/35	51/17913	0.004453084	0.027374387	0.015951889	MDK/NRP1	2
BP	GO:0016064	immunoglobulin mediated immune response	3/35	171/17913	0.004470525	0.027375492	0.015952533	XCL1/CD19/IGKV1-12	3

Table S3. Gene ontology analysis of prognostic-associated immune genes.

ONTOLOGY	ID	Description	GeneRatio	BgRatio	pvalue	p.adjust	qvalue	geneID	Count
BP	GO:0070997	neuron death	4/35	347/17913	0.004511697	0.027521353	0.016037531	CX3CL1/MDK/NRP1/BID	4
BP	GO:0010634	positive regulation of epithelial cell migration	3/35	173/17913	0.004617876	0.027954011	0.016289654	KDR/NRP1/TEK	3
BP	GO:0019724	B cell mediated immunity	3/35	173/17913	0.004617876	0.027954011	0.016289654	XCL1/CD19/IGKV1-12	3
BP	GO:0032655	regulation of interleukin-12 production	2/35	53/17913	0.004801074	0.028733976	0.016744163	AGER/MDK	2
BP	GO:0050853	B cell receptor signaling pathway	2/35	53/17913	0.004801074	0.028733976	0.016744163	VAV3/CD19	2
BP	GO:0060421	positive regulation of heart growth	2/35	53/17913	0.004801074	0.028733976	0.016744163	HAMP/TGFBR3	2
BP	GO:0001666	response to hypoxia	4/35	354/17913	0.004841672	0.028868012	0.01682227	NDRG1/AGER/TEK/TGFBR3	4
BP	GO:0032615	interleukin-12 production	2/35	55/17913	0.005161244	0.030658174	0.017865452	AGER/MDK	2
BP	GO:0002040	sprouting angiogenesis	3/35	181/17913	0.00523668	0.030990206	0.018058937	KDR/NRP1/TEK	3
BP	GO:1903708	positive regulation of hemopoiesis	3/35	182/17913	0.005317373	0.031350756	0.018269041	AGER/IL34/MDK	3
BP	GO:1901653	cellular response to peptide	4/35	364/17913	0.005340818	0.031372363	0.018281632	AGER/KL/INSR/SHC1	4
BP	GO:0036293	response to decreased oxygen levels	4/35	365/17913	0.005392563	0.031559425	0.018390639	NDRG1/AGER/TEK/TGFBR3	4
BP	GO:0050731	positive regulation of peptidyl-tyrosine phosphorylation	3/35	184/17913	0.005481008	0.031959111	0.018623548	IL11/TGFA/NRP1	3
BP	GO:0030888	regulation of B cell proliferation	2/35	57/17913	0.005533498	0.032146987	0.018733029	VAV3/TNFRSF13C	2
BP	GO:0032233	positive regulation of actin filament bundle assembly	2/35	58/17913	0.005724127	0.033058664	0.019264291	CX3CL1/NRP1	2
BP	GO:0050866	negative regulation of cell activation	3/35	187/17913	0.005732114	0.033058664	0.019264291	XCL1/CX3CL1/MDK	3
BP	GO:0061900	glial cell activation	2/35	59/17913	0.005917742	0.033760934	0.019673525	AGER/CX3CL1	2
BP	GO:2000351	regulation of endothelial cell apoptotic process	2/35	59/17913	0.005917742	0.033760934	0.019673525	KDR/TEK	2
BP	GO:2000514	regulation of CD4-positive, alpha-beta T cell activation	2/35	59/17913	0.005917742	0.033760934	0.019673525	XCL1/AGER	2
BP	GO:0051785	positive regulation of nuclear division	2/35	60/17913	0.006114332	0.034757457	0.020254229	TGFA/INSR	2
BP	GO:0002709	regulation of T cell mediated immunity	2/35	61/17913	0.006313884	0.035509999	0.020692759	XCL1/AGER	2
BP	GO:0032835	glomerulus development	2/35	61/17913	0.006313884	0.035509999	0.020692759	PDGFD/TEK	2
BP	GO:0046635	positive regulation of alpha-beta T cell activation	2/35	61/17913	0.006313884	0.035509999	0.020692759	XCL1/EBI3	2
BP	GO:0031099	regeneration	3/35	194/17913	0.006344677	0.035557096	0.020720203	HAMP/MDK/TGFBR3	3
BP	GO:0032729	positive regulation of interferon-gamma production	2/35	62/17913	0.006516387	0.036390807	0.021206032	EBI3/TNFRSF13C	2
BP	GO:0032869	cellular response to insulin stimulus	3/35	197/17913	0.006618743	0.036832725	0.021463551	KL/INSR/SHC1	3
BP	GO:0001503	ossification	4/35	388/17913	0.006677587	0.036901232	0.021503472	BMP1/KL/MDK/TEK	4
BP	GO:0055074	calcium ion homeostasis	4/35	388/17913	0.006677587	0.036901232	0.021503472	XCL1/CD19/CX3CL1/KL	4

Table S3. Gene ontology analysis of prognostic-associated immune genes.

ONTOLOGY	ID	Description	GeneRatio	BgRatio	pvalue	p.adjust	qvalue	geneID	Count
BP	GO:0055025	positive regulation of cardiac muscle tissue development	2/35	63/17913	0.006721829	0.036975651	0.021546838	HAMP/TGFBR3	2
BP	GO:0070482	response to oxygen levels	4/35	389/17913	0.006737682	0.036975651	0.021546838	NDRG1/AGER/TEK/TGFBR3	4
BP	GO:0048762	mesenchymal cell differentiation	3/35	199/17913	0.006805335	0.037090245	0.021613616	MDK/NRP1/TGFBR3	3
BP	GO:0071229	cellular response to acid chemical	3/35	199/17913	0.006805335	0.037090245	0.021613616	HAMP/PDGFD/KDR	3
BP	GO:0001910	regulation of leukocyte mediated cytotoxicity	2/35	64/17913	0.006930198	0.03751295	0.021859939	XCL1/AGER	2
BP	GO:0046622	positive regulation of organ growth	2/35	64/17913	0.006930198	0.03751295	0.021859939	HAMP/TGFBR3	2
BP	GO:0050707	regulation of cytokine secretion	3/35	201/17913	0.006995047	0.037735187	0.021989443	AGER/CX3CL1/MDK	3
BP	GO:0050808	synapse organization	4/35	394/17913	0.00704357	0.037868145	0.022066922	NDRG1/CX3CL1/INSR/NRP1	4
BP	GO:0072577	endothelial cell apoptotic process	2/35	65/17913	0.007141484	0.038264843	0.02229809	KDR/TEK	2
BP	GO:0043030	regulation of macrophage activation	2/35	66/17913	0.007355674	0.039017055	0.022736427	AGER/CX3CL1	2
BP	GO:0061035	regulation of cartilage development	2/35	66/17913	0.007355674	0.039017055	0.022736427	BMP1/MDK	2
BP	GO:1903672	positive regulation of sprouting angiogenesis	2/35	66/17913	0.007355674	0.039017055	0.022736427	KDR/NRP1	2
BP	GO:0051495	positive regulation of cytoskeleton organization	3/35	205/17913	0.007383886	0.039036145	0.022747551	CX3CL1/NRP1/TEK	3
BP	GO:0006644	phospholipid metabolic process	4/35	402/17913	0.007551998	0.039660494	0.023111378	VAV3/CD19/FLT1/TEK	4
BP	GO:0019216	regulation of lipid metabolic process	4/35	402/17913	0.007551998	0.039660494	0.023111378	VAV3/CD19/FLT1/TEK	4
BP	GO:1901215	negative regulation of neuron death	3/35	207/17913	0.007583038	0.039692075	0.023129781	CX3CL1/MDK/NRP1	3
BP	GO:0043523	regulation of neuron apoptotic process	3/35	210/17913	0.007887717	0.041151048	0.02397997	CX3CL1/MDK/NRP1	3
BP	GO:0097191	extrinsic apoptotic signaling pathway	3/35	213/17913	0.008199571	0.042575328	0.024809941	CX3CL1/NRP1/BID	3
BP	GO:0072507	divalent inorganic cation homeostasis	4/35	412/17913	0.008221057	0.042575328	0.024809941	XCL1/CD19/CX3CL1/KL	4
BP	GO:0050871	positive regulation of B cell activation	2/35	70/17913	0.008241252	0.042575328	0.024809941	VAV3/TNFRSF13C	2
BP	GO:0034329	cell junction assembly	3/35	214/17913	0.008305125	0.042765999	0.02492105	KDR/NRP1/TEK	3
BP	GO:0046578	regulation of Ras protein signal transduction	3/35	215/17913	0.008411481	0.043173491	0.025158509	VAV3/NRP1/SHC1	3
BP	GO:0048588	developmental cell growth	3/35	216/17913	0.008518642	0.04358247	0.025396833	HAMP/SEMA3G/NRP1	3
BP	GO:0051881	regulation of mitochondrial membrane potential	2/35	73/17913	0.008935374	0.045523069	0.026527679	KDR/BID	2
BP	GO:0002526	acute inflammatory response	3/35	220/17913	0.008955358	0.045523069	0.026527679	HAMP/CD19/IGKV1-12	3
BP	GO:2001234	negative regulation of apoptotic signaling pathway	3/35	223/17913	0.009291409	0.047080428	0.0274352	CX3CL1/NRP1/BID	3
BP	GO:0031100	animal organ regeneration	2/35	75/17913	0.009412208	0.047540644	0.027703382	HAMP/TGFBR3	2
BP	GO:0022604	regulation of cell morphogenesis	4/35	430/17913	0.009521898	0.047942001	0.027937265	SEMA3G/MDK/KDR/NRP1	4

Table S3. Gene ontology analysis of prognostic-associated immune genes.

ONTOLOGY	ID	Description	GeneRatio	BgRatio	pvalue	p.adjust	qvalue	geneID	Count
BP	GO:0030516	regulation of axon extension	2/35	76/17913	0.009654818	0.048152645	0.028060014	SEMA3G/NRP1	2
BP	GO:0048844	artery morphogenesis	2/35	76/17913	0.009654818	0.048152645	0.028060014	MDK/NRP1	2
BP	GO:0150076	neuroinflammatory response	2/35	76/17913	0.009654818	0.048152645	0.028060014	AGER/CX3CL1	2
BP	GO:0002768	immune response-regulating cell surface receptor signaling pathway	4/35	434/17913	0.00982823	0.048863867	0.028474464	VAV3/CD19/IGKV1-12/SHC1	4
BP	GO:0060411	cardiac septum morphogenesis	2/35	77/17913	0.009900207	0.049067903	0.028593362	NRP1/TGFBR3	2
BP	GO:0050663	cytokine secretion	3/35	230/17913	0.010104114	0.049830675	0.029037853	AGER/CX3CL1/MDK	3
BP	GO:0001570	vasculogenesis	2/35	78/17913	0.010148366	0.049830675	0.029037853	KDR/TGFBR3	2
BP	GO:0010771	negative regulation of cell morphogenesis involved in differentiation	2/35	78/17913	0.010148366	0.049830675	0.029037853	SEMA3G/NRP1	2
MF	GO:0048018	receptor ligand activity	12/33	397/16969	5.19E-12	5.97E-10	3.61E-10	HAMP/XCL1/CX3CL1/BMP1/EBI3/IL11/IL34/KL/MDK/PDGFD/PMCH/TGFA	12
MF	GO:0030545	receptor regulator activity	12/33	425/16969	1.15E-11	6.62E-10	4.00E-10	HAMP/XCL1/CX3CL1/BMP1/EBI3/IL11/IL34/KL/MDK/PDGFD/PMCH/TGFA	12
MF	GO:0019838	growth factor binding	8/33	121/16969	6.33E-11	2.43E-09	1.47E-09	KL/FLT1/INSR/KDR/NRP1/TEK/TGFBR3/SHC1	8
MF	GO:0005126	cytokine receptor binding	7/33	210/16969	1.31E-07	3.54E-06	2.14E-06	XCL1/CX3CL1/IL11/IL34/TGFBR3/SHC1/BID	7
MF	GO:0019199	transmembrane receptor protein kinase activity	5/33	65/16969	1.54E-07	3.54E-06	2.14E-06	FLT1/INSR/KDR/NRP1/TGFBR3	5
MF	GO:0008083	growth factor activity	6/33	147/16969	3.48E-07	6.68E-06	4.03E-06	BMP1/IL11/IL34/MDK/PDGFD/TGFA	6
MF	GO:0004714	transmembrane receptor protein tyrosine kinase activity	4/33	49/16969	2.36E-06	3.88E-05	2.34E-05	FLT1/INSR/KDR/NRP1	4
MF	GO:0004713	protein tyrosine kinase activity	5/33	120/16969	3.29E-06	4.74E-05	2.86E-05	FLT1/INSR/KDR/NRP1/TEK	5
MF	GO:0005125	cytokine activity	5/33	154/16969	1.12E-05	0.000142489	8.61E-05	XCL1/CX3CL1/BMP1/EBI3/IL34	5
MF	GO:0005154	epidermal growth factor receptor binding	3/33	29/16969	2.37E-05	0.000272034	0.000164341	VAV3/TGFA/SHC1	3
MF	GO:0070851	growth factor receptor binding	4/33	121/16969	8.58E-05	0.00089674	0.000541738	VAV3/IL11/TGFA/SHC1	4
MF	GO:0005159	insulin-like growth factor receptor binding	2/33	15/16969	0.000379044	0.003632506	0.002194466	INSR/SHC1	2
MF	GO:0017134	fibroblast growth factor binding	2/33	21/16969	0.000752574	0.006657385	0.004021852	KL/TGFBR3	2

Table S3. Gene ontology analysis of prognostic-associated immune genes.

ONTOLOGY	ID	Description	GeneRatio	BgRatio	pvalue	p.adjust	qvalue	geneID	Count
MF	GO:0005179	hormone activity	3/33	103/16969	0.001038077	0.008527065	0.005151362	HAMP/KL/PMCH	3
MF	GO:0008009	chemokine activity	2/33	30/16969	0.001541939	0.011821533	0.007141613	XCL1/CX3CL1	2
MF	GO:0015026	coreceptor activity	2/33	40/16969	0.002731481	0.019430977	0.011738622	NRP1/TGFBR3	2
MF	GO:0008201	heparin binding	3/33	148/16969	0.002928243	0.019430977	0.011738622	MDK/NRP1/TGFBR3	3
MF	GO:0003823	antigen binding	3/33	150/16969	0.00304137	0.019430977	0.011738622	IGKV1-12/IGLV4-69/IGLV9-49	3
MF	GO:0042379	chemokine receptor binding	2/33	47/16969	0.003753538	0.022718781	0.013724847	XCL1/CX3CL1	2
MF	GO:0005539	glycosaminoglycan binding	3/33	199/16969	0.006691015	0.038473336	0.023242473	MDK/NRP1/TGFBR3	3
MF	GO:0001540	amyloid-beta binding	2/33	71/16969	0.008381408	0.045898188	0.027727967	AGER/INSR	2
MF	GO:1901681	sulfur compound binding	3/33	221/16969	0.008916765	0.046610364	0.028158206	MDK/NRP1/TGFBR3	3

Table S4. KEGG pathway enrichment of prognostic-associated immune genes

ID	Description	GeneRatio	BgRatio	pvalue	p.adjust	qvalue	geneID	Count
hsa04014	Ras signaling pathway	7/25	232/7946	5.07E-06	0.00051197	0.00040552	PDGFD/TGFA/FLT1/INSR/KDR/TEK/SHC1	7
hsa04015	Rap1 signaling pathway	6/25	210/7946	3.69E-05	0.00186351	0.00147605	VAV3/PDGFD/FLT1/INSR/KDR/TEK	6
hsa04151	PI3K-Akt signaling pathway	7/25	354/7946	7.86E-05	0.0024666	0.00195375	CD19/PDGFD/TGFA/FLT1/INSR/KDR/TEK	7
hsa01521	EGFR tyrosine kinase inhibitor resistance	4/25	79/7946	9.77E-05	0.0024666	0.00195375	PDGFD/TGFA/KDR/SHC1	4
hsa05323	Rheumatoid arthritis	4/25	93/7946	0.00018428	0.00292264	0.00231496	CXCL2/IL11/FLT1/TEK	4
hsa04060	Cytokine-cytokine receptor interaction	6/25	294/7946	0.00023807	0.00292264	0.00231496	CXCL2/XCL1/CX3CL1/IL11/IL34/TNFRSF13C	6
hsa04010	MAPK signaling pathway	6/25	295/7946	0.00024251	0.00292264	0.00231496	PDGFD/TGFA/FLT1/INSR/KDR/TEK	6
hsa04061	Viral protein interaction with cytokine and cytokine receptor	4/25	100/7946	0.00024384	0.00292264	0.00231496	CXCL2/XCL1/CX3CL1/IL34	4
hsa04062	Chemokine signaling pathway	5/25	189/7946	0.00026043	0.00292264	0.00231496	CXCL2/XCL1/VAV3/CX3CL1/SHC1	5
hsa04510	Focal adhesion	5/25	199/7946	0.00033085	0.00334154	0.00264676	VAV3/PDGFD/FLT1/KDR/SHC1	5
hsa04650	Natural killer cell mediated cytotoxicity	4/25	131/7946	0.00068225	0.00626427	0.00496179	ULBP2/VAV3/SHC1/BID	4
hsa04215	Apoptosis - multiple species	2/25	32/7946	0.00444911	0.03601982	0.02853055	BIRC5/BID	2
hsa04066	HIF-1 signaling pathway	3/25	109/7946	0.00463621	0.03601982	0.02853055	FLT1/INSR/TEK	3
hsa05340	Primary immunodeficiency	2/25	38/7946	0.00623356	0.04497072	0.03562037	CD19/TNFRSF13C	2
